# Supplementary material for: Single-cell genomics of multiple uncultured stramenopiles reveals underestimated functional diversity across oceans
Source: Nat Commun. 2018 Jan 22;9:310. doi: 10.1038/s41467-017-02235-3 (PMC5778133; doi:10.1038/s41467-017-02235-3)
Supplement: Supplementary file 1 — Supplementary Information [file 41467_2017_2235_MOESM1_ESM.pdf]

Supplementary Information

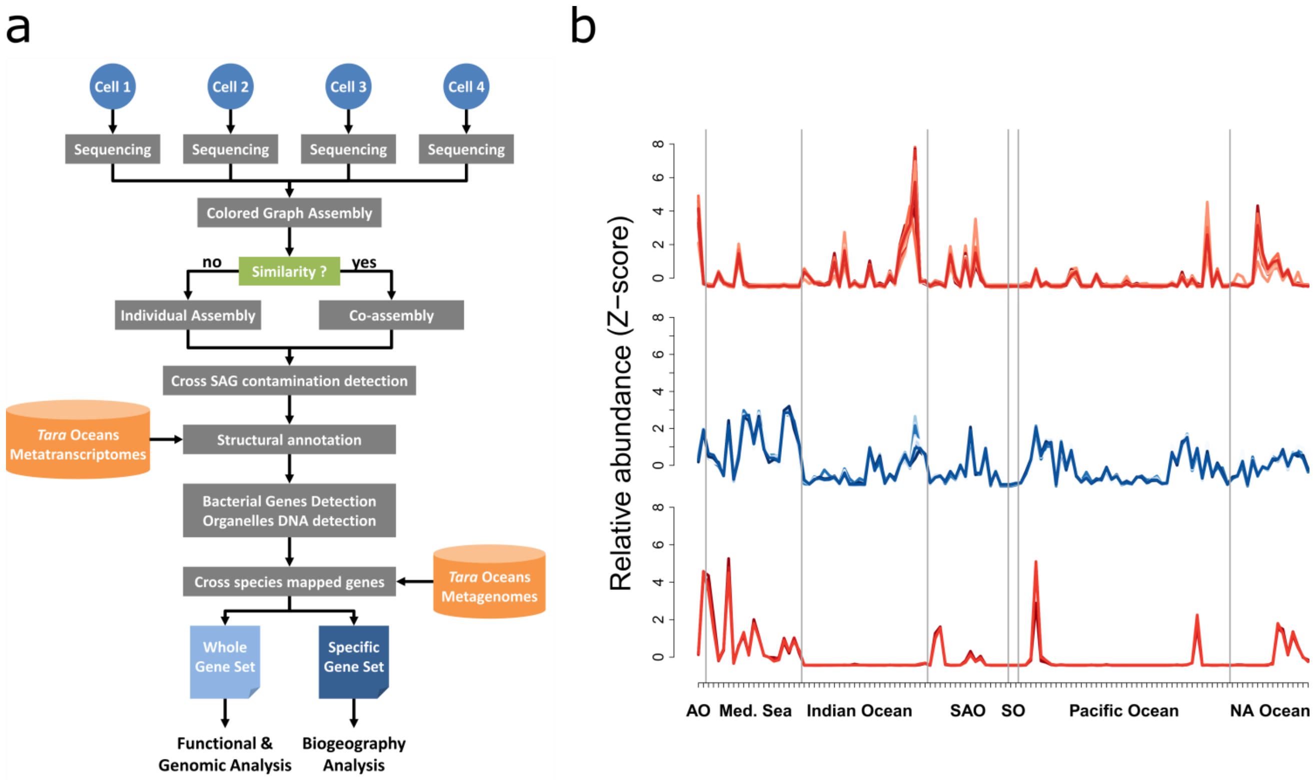

Supplementary Figure 1. Schematic pipeline for single-cell genome assembly, cleaning and annotation. a. The assembly process was optimized to account for multiple cells putatively originating from the same species. We used metatranscriptomes from *Tara* Oceans to improve gene detection accuracy. We exploited metagenomic fragment recruitment results to filter out assembled genomic regions that likely correspond to other species and highly conserved genes that can be mapped by reads from other species. **b**. Detection of foreign contigs in the MAST4-A1 assembly by fragment recruitment analysis. The x-axis represents *Tara* Oceans metagenomes from the 0.8-5 µm size fraction (AO: Atlantic Ocean, Med. Sea: Mediterranean Sea, SAO: South Atlantic Ocean, SO: Southern Ocean, NA Ocean: North Atlantic Ocean). The y-axis represents the z-score of the abundance of mapped metagenomic reads for each metagenome. The central (blue) graph shows the typical pattern obtained for the 20 longest contigs of MAST-4A1 as an example. The red graphs show two subsets of

contigs from the MAST-4A1 assembly that were rejected by the filter because of a signature statistically deviating from the MAST4-A1 signature. Two different patterns are shown: the upper red plot is a subset of contigs taxonomically assigned to *Bathycoccus prasinos*, whereas the lower corresponds to contigs assigned to *Prochlorococcus* MED4.

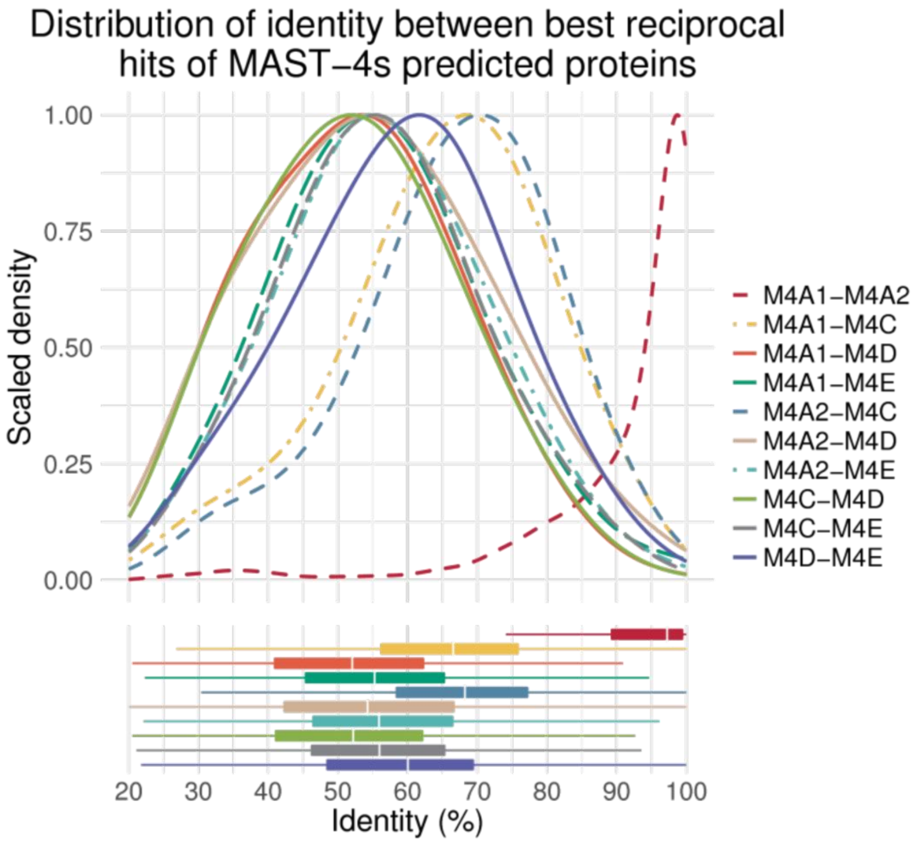

Supplementary Figure 2: Distribution of identity between best reciprocal hits of MAST-4 predicted proteins. All-versus-all comparisons of the distribution of identity between MAST-4 predicted proteomes. Except the 2 assemblies of MAST-4 A that came from nearly identical genomes, the median identity between MAST-4 orthologs ranges from 53% to 68%. Median identity with the previously sequenced MAST-4 D ranges from 53% to 60%.

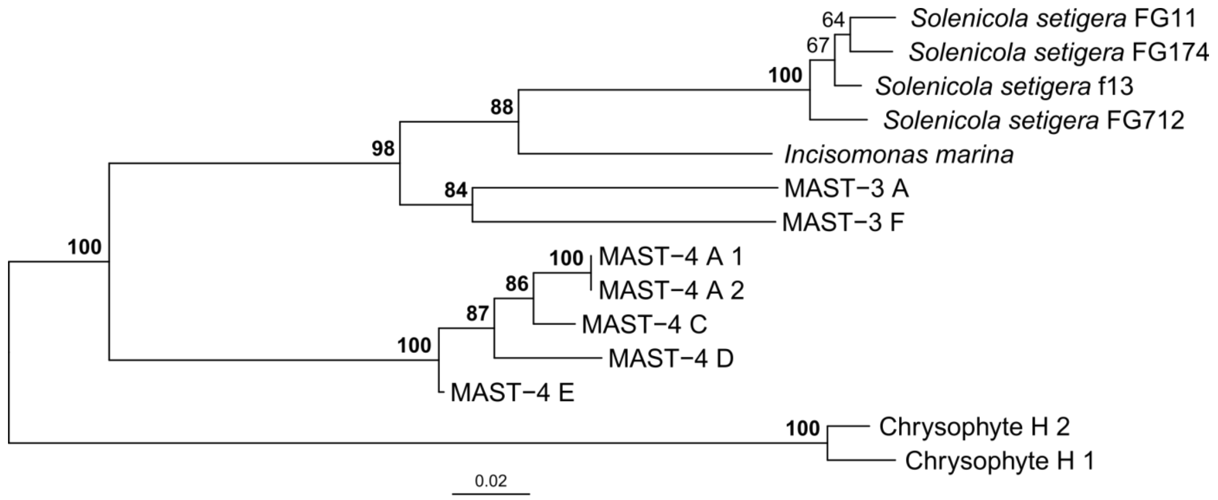

Supplementary Figure 3. Maximum-likelihood phylogenetic tree inferred from the 18S rDNA sequences of the SAGs and close taxa. Bootstrap values are indicated for each node and the scale bar represents the expected number of substitutions per site.

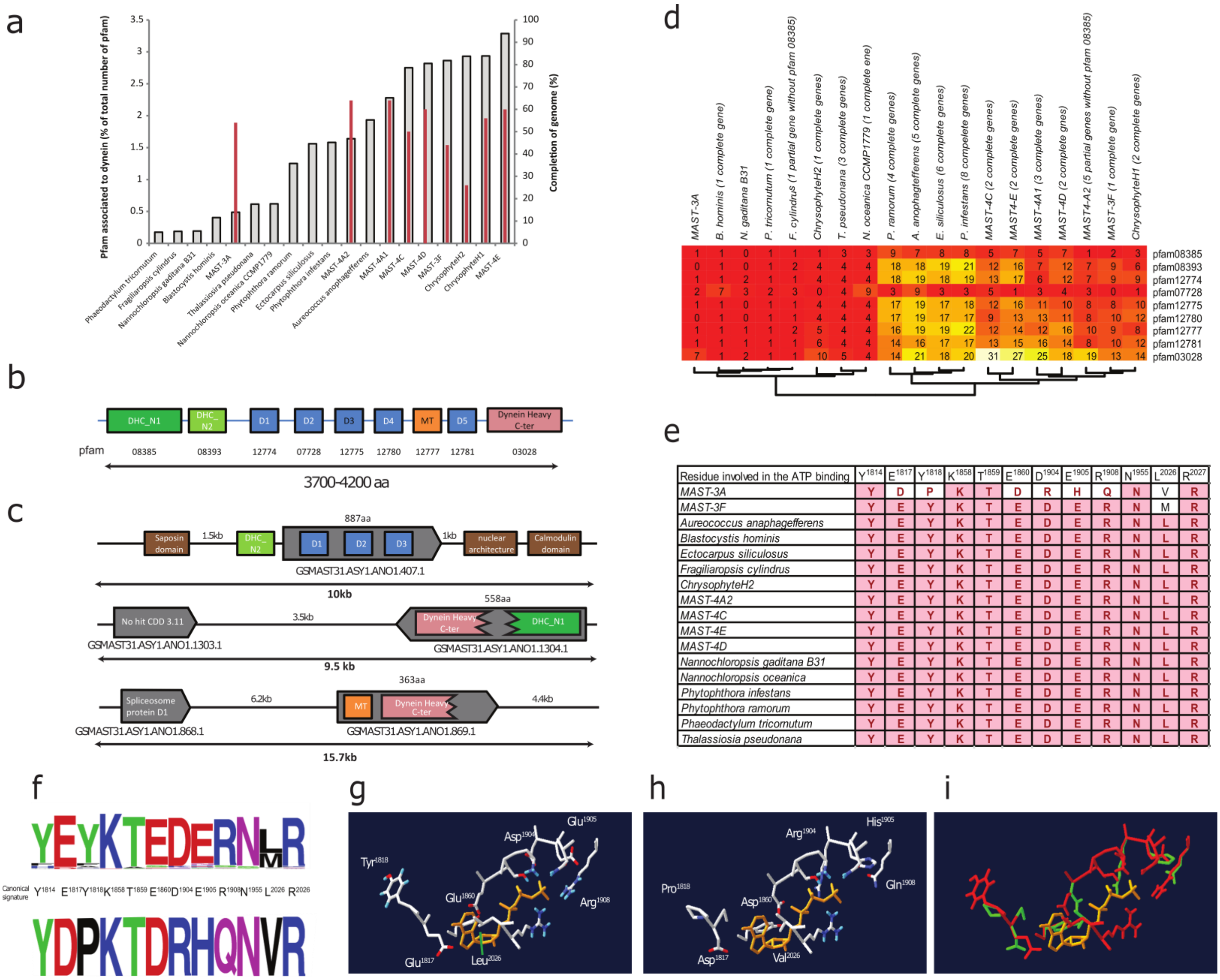

Supplementary Figure 4. Dynein heavy chains encoded in the co-assembled genomes.

a. Number of Pfam domains associated with dynein heavy chain (DHC) protein genes per genome (grey bars) for the heterotrophic lineages and in genomes from a range of reference organisms including from marine environments. The estimated genome completion of each co-assembled genome calculated from BUSCO output is indicated by red bars. The MAST-3A genome has particularly few DHC domains. **b.** Canonical DHC gene structure. Nine Pfam domains were detected for DHCs. The ATP binding and hydrolysis occur in the D1 module (associated with pfam12774). **c.** Genomic structure in MAST-3A of genes encoding proteins

with similarities to the DHC-associated Pfam. Grey boxes indicate genes predicted by GAZE. The colored boxes indicate the domain found by CDD search analysis after a six-frame scaffold sequence translation. Broken boxes indicate truncated domains. All regions contain incomplete genes, and remnant sequences are indicative of pseudogenization. **d.** Heatmap of Pfam domains associated with dynein heavy chains in stramenopiles. The number of genes per organism with all Pfam domains is indicated next to the name of the organism. Hierarchical clustering was performed between organisms. **e.** Alignment of residue involved in ATP binding. For each organism, the protein with the highest conservation of the canonical residues was selected and used for alignment. Red indicates the residue conservation in the corresponding protein. **f.** Conserved canonical signature of the DHC D1 module (conserved residues are implied in ATP binding) in all proteins with similarities to the Pfam12774 motif in SAG organisms (upper panel). Sequence of the same residues in the only protein from MAST-3A with similarities to the Pfam12774 domain (lower panel). Nature and position of the residues shown in the structural models (**g-i**) are indicated in the middle. **g.** Three-dimensional structure of residues contacting ATP (indicated in orange) with a residue that corresponds to the canonical signature. **h.** Three-dimensional structure of the residue found in a MAST-3A protein with Pfam12774 signature. **i.** Comparison between the canonical residue (red) and MAST-3A residue (green). Modified residues are more distant from ATP, which indicates decreased affinity.

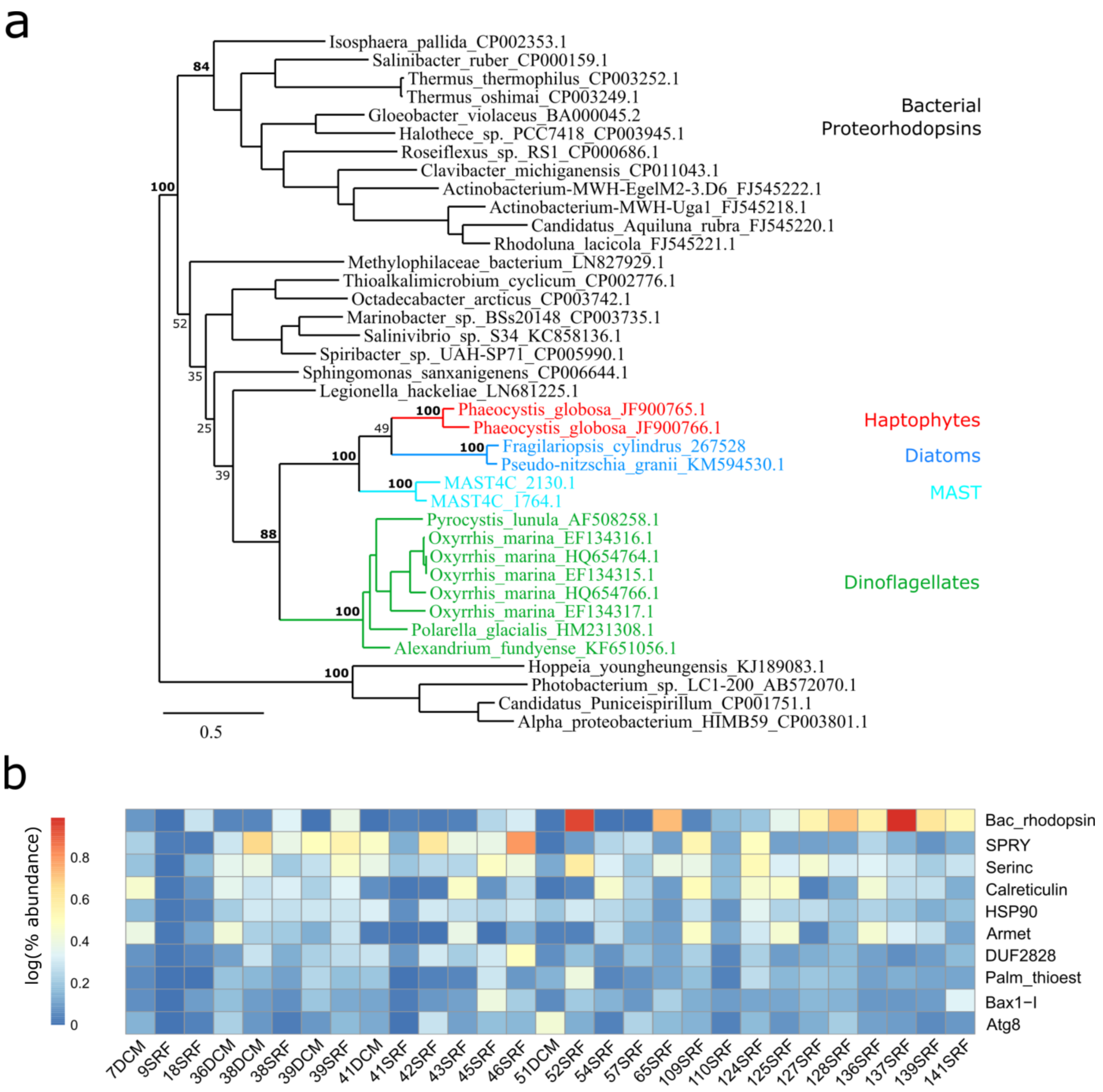

c

```
MAST-4C_1764    MMASTIFYWMMVSNVKPRYRSALTITGLVTFIAAYHYFRIFNSWVEAYRYPTPGGSSKTTIGNPELTGKPFNDAYRYMDWLLTVPLLLIEIIFVMDLKPE
MAST-4C_2130    MMASTIFYWMMVSNVKPKFRSALTITGLVTFIAAYHYFRIFNSWVEAYKYPVPGGSSKTTIGNPELTGKPFNDAYRYMDWLLTVPLLLIEIVFVMELKPE
                *****************.:*****************************.**.***************************************:***:****

MAST-4C_1764    ETSKKAWQLGASAALMIILGYPGELILEADKLSTRWVYWALAMIPFLFVVYTLLVGLAGATRNETPEVASAIRYAQWMTVLSWCTYPIVYIIPMFGAKGS
MAST-4C_2130    ETSKKAWQLGASAALMICLGYPGELILEADKLSTRWVYWALAMIPFLFVVYTLLVGLAGALRDESPEIASSIRTAQWMTVISWCTYPIVYIIPMFGAKGA
                ***************** ****************************************** *:*:**:**:** ******:******************:

MAST-4C_1764    NAVVGIQVGYCIADVISKCGVGFVIYNITARKSAQSSDKDGYNPIQN
MAST-4C_2130    NAVVGIQLGYCIADVISKCGVGFIIYNITAKKSAL-TDKDGYRAVQ-
                *******:***************:******.***  :*****..:*
```

Supplementary Figure 5. The proteorhodopsin gene candidates in MAST-4C are typical of eukaryotic sequences and represent some of the most expressed transcripts in different environments.

a. Phylogenetic tree of proteorhodopsins based on the tBLASTN best hits of the MAST4C_1764.1 gene against the nr-nuc database. The two MAST-4C and *Phaeocystis globosa* proteorhodopsins were added manually. Proteorhodopsin sequences were aligned with MUSCLE 3.7 and the maximum likelihood tree was constructed with 100 bootstraps. Bootstrap values of important nodes are reported and bootstrap values > 80 are in bold. **b**. Heatmap of the log relative metatranscriptomic RPKM values (in log percentages) for the 10 most expressed – on average – Pfam domains in the *Tara* Oceans samples of the 0.8-5µm size fraction where more than 50% of MAST-4 C genes are expressed. The proteorhodopsin candidates (two genes) constitute the most expressed category in these samples. **c**. MUSCLE alignment of the two MAST-4 C proteorhodopsins. Residues implicated in the proton-pump function are colored in red.

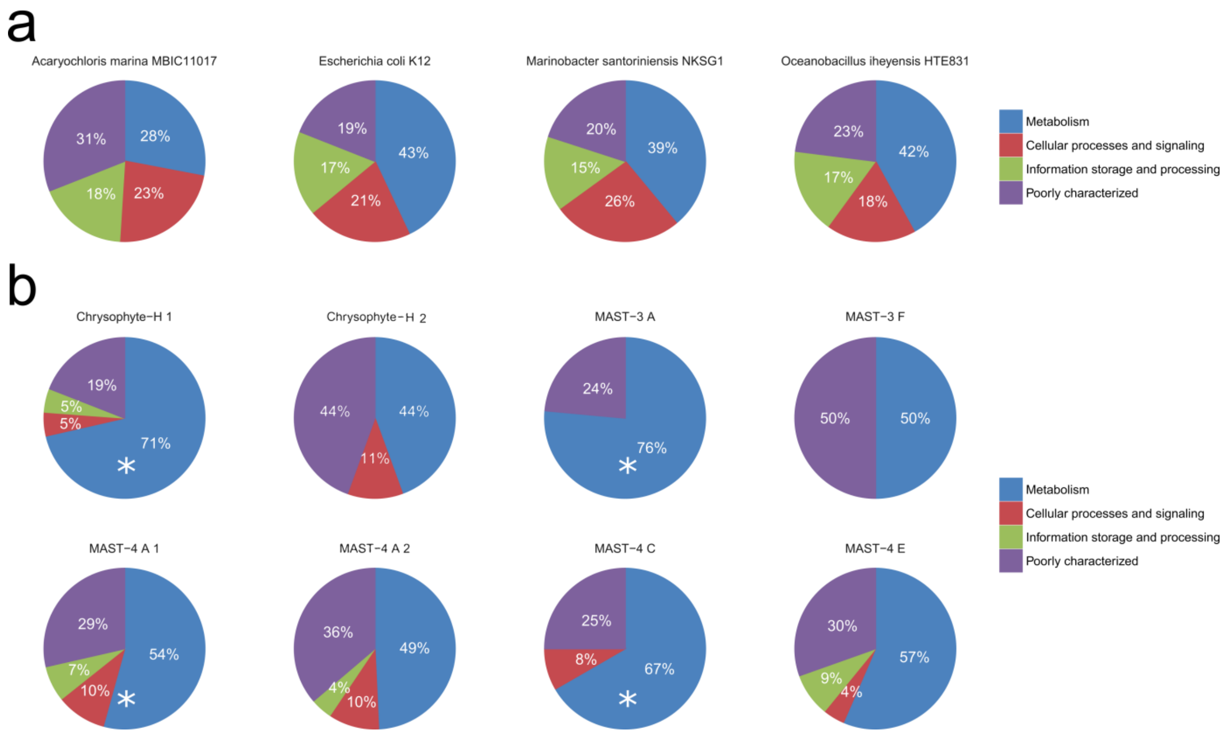

Supplementary Figure 6. Relative proportions and functions of putative Horizontally Transferred Genes. a. Fraction of four reference genomes annotated according to different COG categories (blue: metabolism; red: cellular processes and signaling; green: information storage and processing; purple: poorly characterized). **b.** Horizontally transferred genes annotated according to different COG categories. COG categories linked to metabolism (blue) that are significantly over-represented (one sided Chi-squared test p-value < 0.05) in the MAST and chrysophyte genomes compared to bacterial genomes are annotated with a star (*).

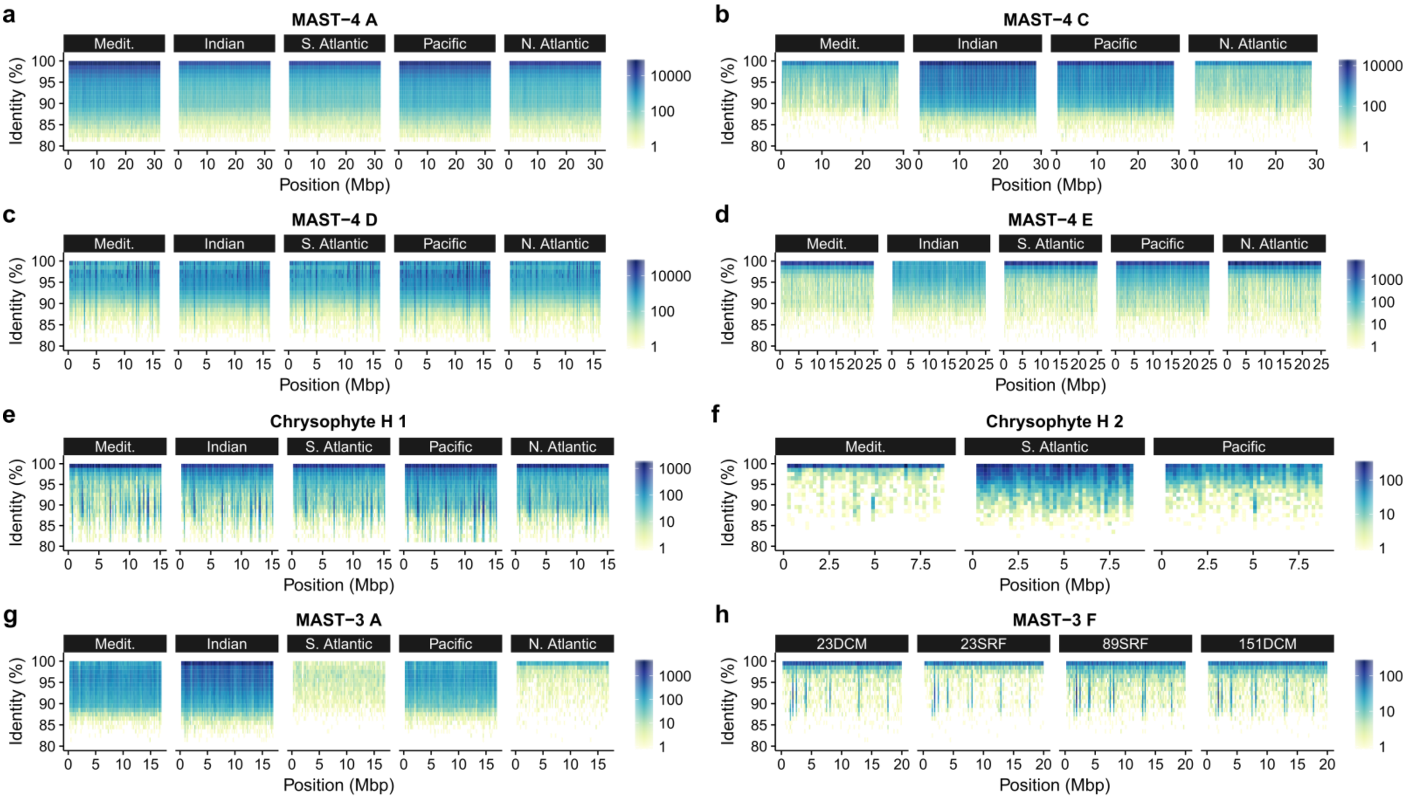

Supplementary Figure 7. Fragment recruitment plots with *Tara* Oceans metagenomic samples grouped by ocean regions. The x-axis corresponds to the matching positions of metagenomics reads on arbitrarily concatenated scaffolds, and the y-axis shows the identity level of alignments. The 2D space is binned (200 kbp on the x-axis and 1% identity on the y-axis) to improve readability. Colour scale represents the density of reads recruited by bins. Bins that contained highly conserved genes were removed from this representation. For the same SAG lineage, the centre of identity distributions may vary among regions (for MAST-3A, MAST-4E, and Chrysophyte H2), or be relatively constant (for MAST-4C, Chrysophyte H1, MAST-4A, and MAST-3F). The mean identity value reflects the genetic distance of the assemblies from the genetically closest abundant genome in the considered region; this is more precisely reported for each metagenomic sample on a world map in Figure 2.

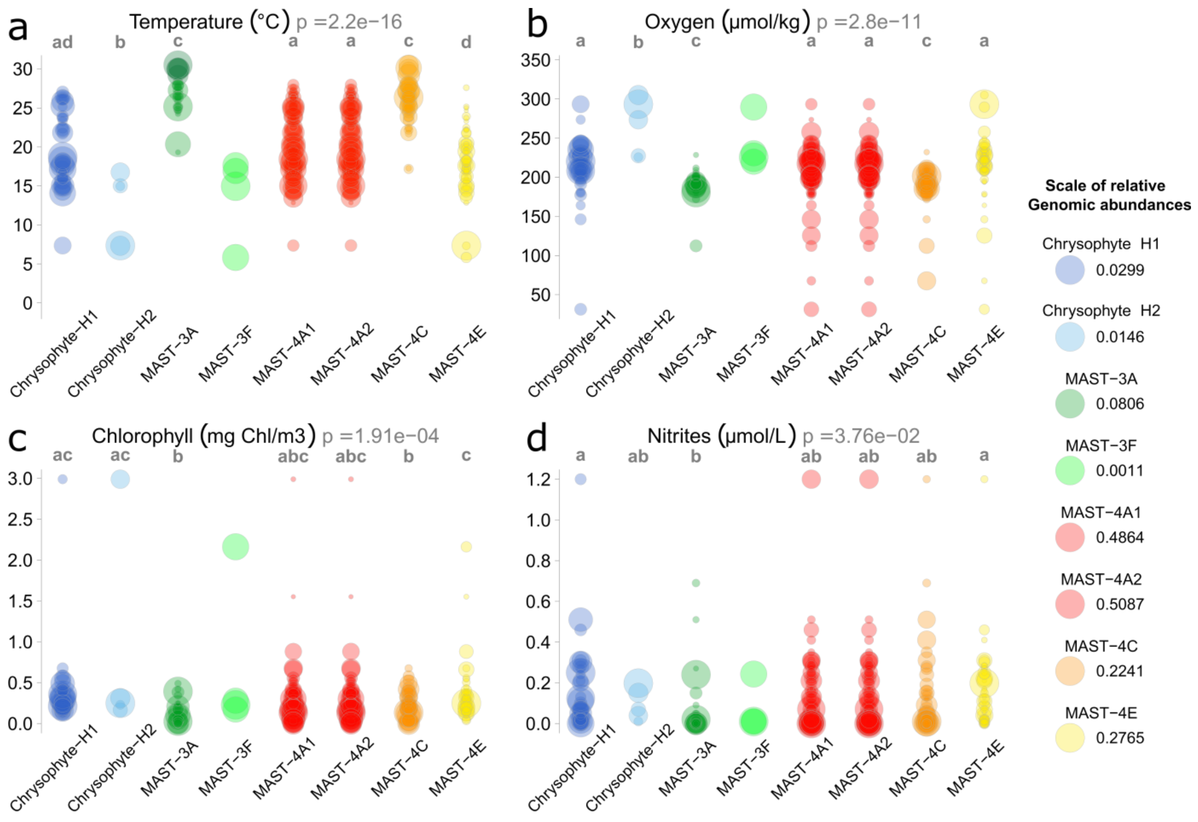

Supplementary Figure 8. Abundance plots based on several contextual parameters measured at each sampling site. Each parameter is indicated above the corresponding graph. The x-axis corresponds to each lineage, and the y-axis shows the value of the parameter (unit indicated above each figure). Circle size corresponds to relative co-assembled genome abundance at a particular station/depth measured by metagenomic read mapping. This is normalized on the figure, and each correspondence between circle size and relative abundance is indicated on the right. Kruskall-Wallis probabilities are indicated in grey close to parameter names. Lineage statistical classes were computed on parameters (when Kruskall-Wallis probabilities $< 10^{-2}$). Temperature is the highest discriminant parameter; groupings included MAST-4A1 and MAST-4A2 (class a), and MAST-3A and MAST-4C (class c), but MAST-4E1 was an independent class (class d). Chrysophyte H1 (class ad) is present in a range of temperatures that corresponds to both classes a and d of MAST-4A and MAST-4E. Chrysophyte H2 is independently classified (class b).

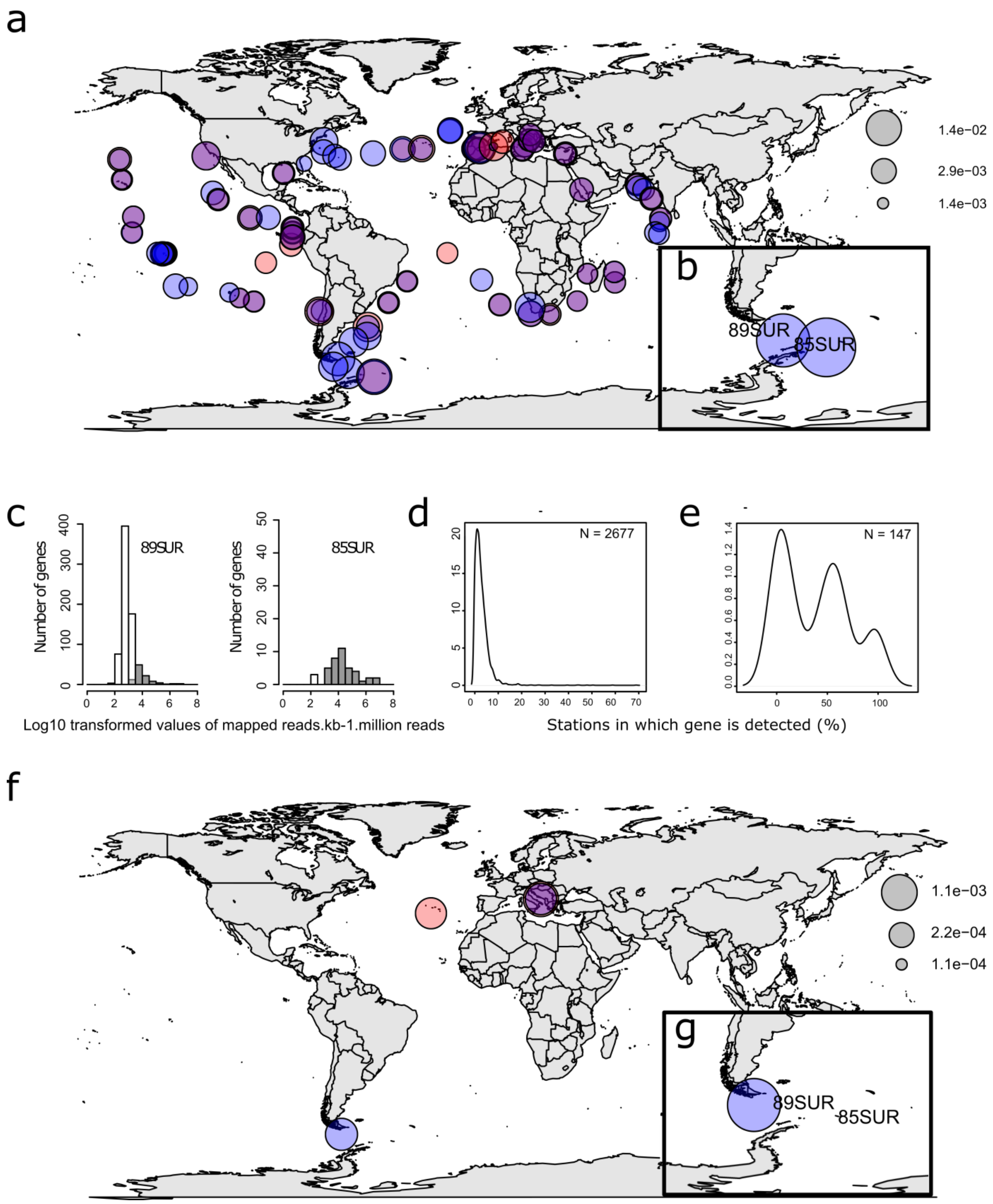

Supplementary Figure 9. Importance of detecting specifically-matching genes to study heterotrophic protist biogeography. a. Relative abundance of MAST-3F calculated from the initial whole gene dataset (cleaned for organelle scaffolds). Circle size is proportional to the relative organismal abundance (scale indicated by the grey circles). Blue circles indicate surface stations and red circles indicate DCM stations. This organism was apparently ubiquitously distributed. These results were inconsistent with distributions observed by V9

sequencing and may be evidence of cross-mapping. **b.** Close-up of relative MAST-3F abundances from stations 85SUR and 89SUR. **c.** Histogram plot of metagenomic RPKM values of inlier (white bar) and outlier (grey bar) gene dataset (x-axis : log10-transformed values of metagenomic RPKM, y-axis: number of genes). **d.** Inlier and **e.** Outlier datasets of occurrence of genes per station was calculated for each SAG lineage. The plot represents the density of genes detected (metagenomic RPKM >0) in a specific percentage of stations (x-axis: percentage of station positive for the gene). In this example, the MAST-3F genes were detected in 3 to 5% of the *Tara* Oceans stations (**d**). However, genes from the outlier data set showed a different occurrence pattern; some were detected in all stations or about 50% of the stations. The MAST-3F distribution shown with the inlier dataset is more discrete, with two major locations, in Mediterranean Sea (where MAST-3F was sampled) and in South Atlantic Ocean (**f**). **g.** The use of the inlier dataset showed that the entire signal in station 85 was due to non-specific matches.

Pfam	Ectocarpus	Chrysophyte H 1	Chrysophyte H 2	MAST-3 A	MAST-3 F	MAST-4 A 1	MAST-4 A 2	MAST-4 C	MAST-4 E	MAST-4 D	Domain
pfam00069	2.99	3.01	2.97	3.77	3.54	3.24	2.99	3.5	3.42	3.06	Protein kinase domain
pfam12796	3.58	1.29	1.49	0.63	1.42	3.41	3.18	1.3	1.65	4.06	Ankyrin repeats (3 copies)
pfam00225	0.58	1.56	1.13	0.77	1.87	1.31	1.21	1.18	1.49	0.95	Kinesin motor domain
pfam00271	0.75	1.21	1.34	1.13	1.46	0.65	0.83	0.74	0.78	0.47	Helicase conserved C−terminal domain
pfam13499	0.4	0.63	0.5	0.17	0.42	0.84	0.72	0.79	0.89	1.55	EF−hand domain pair
pfam00071	0.56	0.55	0.85	0.77	0.83	0.44	0.5	0.66	0.84	0.53	Ras family
pfam00270	0.53	0.94	0.78	0.9	1.29	0.44	0.53	0.49	0.63	0.45	DEAD/DEAH box helicase
pfam00226	0.47	0.51	0.35	0.57	0.54	0.58	0.5	0.39	0.57	0.68	DnaJ domain
pfam03028	0.19	0.55	0.71	0.17	0.5	0.54	0.42	0.71	0.71	0.47	Dynein heavy chain and region D6 of dynein motor
pfam00443	0.37	0.39	0.64	0.47	0.46	0.3	0.4	0.47	0.5	0.53	Ubiquitin carboxyl−terminal hydrolase
pfam00415	0.31	0.23	0.28	0.2	0.42	0.42	0.5	0.39	0.5	0.61	Regulator of chromosome condensation (RCC1) repeat
pfam00076	0.53	0.43	0.14	0.47	0.5	0.33	0.31	0.37	0.37	0.47	RNA recognition motif. (a.k.a. RRM. RBD. or RNP domain)
pfam00632	0.2	0.27	0.28	0.37	0.62	0.42	0.42	0.42	0.42	0.24	HECT−domain (ubiquitin−transferase)
pfam00112	0.09	0.27	0.57	0.43	0.08	0.35	0.42	0.54	0.47	0.26	Papain family cysteine protease
pfam00884	0.1	0.2	0.28	0.53	0.04	0.49	0.7	0.42	0.47	0.32	Sulfatase
pfam12781	0.18	0.43	0.42		0.42	0.35	0.18	0.3	0.39	0.37	ATP−binding dynein motor region D5
pfam00027	0.21	0.43	0.35	0.13	0.29	0.35	0.31	0.52	0.6	0.82	Cyclic nucleotide−binding domain
pfam00085	0.43	0.31	0.21	0.33	0.12	0.4	0.35	0.27	0.26	0.45	Thioredoxin
pfam00378	0.13	0.31	0.14	0.4	0.08	0.68	0.44	0.44	0.26	0.32	Enoyl−CoA hydratase/isomerase family
pfam12774	0.19	0.31	0.28	0.03	0.37	0.14	0.15	0.32	0.44	0.32	Hydrolytic ATP binding site of dynein motor region D1
pfam12777	0.2	0.31	0.35	0.03	0.37	0.26	0.22	0.27	0.37	0.42	Microtubule−binding stalk of dynein motor
pfam00063	0.26	0.39	0.28	0.57	0.29	0.3	0.2	0.42	0.21	0.11	Myosin head (motor domain)
pfam12780	0.18	0.43	0.28		0.42	0.28	0.18	0.2	0.34	0.29	P−loop containing dynein motor region D4
pfam12775	0.18	0.35	0.28	0.03	0.33	0.23	0.18	0.27	0.42	0.26	P−loop containing dynein motor region D3
pfam03133	0.21	0.31	0.21	0.17	0.42	0.21	0.31	0.49	0.42	0.21	Tubulin−tyrosine ligase family
pfam00171	0.08	0.23	0.28	0.37	0.29	0.26	0.22	0.27	0.26	0.11	Aldehyde dehydrogenase family
pfam08393	0.2	0.23	0.28		0.37	0.16	0.13	0.3	0.42	0.32	Dynein heavy chain. N−terminal region 2
pfam00240	0.19	0.2	0.35	0.1	0.12	0.56	0.35	0.05	0.29	0.37	Ubiquitin family
pfam07714	0.57	0.12	0.21	0.23	0.08	0.3	0.33	0.1	0.13	0.29	Protein tyrosine kinase
pfam13561	0.14	0.04		0.3	0.08	0.42	0.4	0.47	0.31	0.11	Enoyl−(Acyl carrier protein) reductase
pfam00520	0.13	0.27	0.35	0.07	0.08	0.21	0.2	0.22	0.44	0.26	Ion transport protein
pfam00648	0.09	0.27	0.28	0.17	0.12	0.16	0.22	0.32	0.16	0.26	Calpain family cysteine protease
pfam13424	0.78	0.23	0.14			0.37	0.33	0.1	0.08	0.45	Tetratricopeptide repeat
pfam06602	0.04	0.16	0.21	0.17	0.21	0.23	0.18	0.22	0.16	0.05	Myotubularin−like phosphatase domain
pfam00107	0.05	0.04	0.14	0.13	0.04	0.33	0.35	0.2	0.21	0.18	Zinc−binding dehydrogenase
pfam01926	0.22	0.35	0.28	0.17	0.5	0.14	0.15	0.1	0.16	0.08	50S ribosome−binding GTPase
pfam01363	0.22	0.12	0.21	0.23	0.04	0.16	0.13	0.15	0.13	0.32	FYVE zinc finger
pfam14580	0.15	0.12	0.07	0.13	0.12	0.16	0.13	0.27	0.21	0.26	Leucine−rich repeat
pfam00628	0.24	0.04	0.14	0.07	0.17	0.12	0.13	0.12	0.24	0.18	PHD−finger
pfam13855	0.8	0.27	0.21	0.07	0.12	0.14	0.13	0.07	0.13	0.32	Leucine rich repeat
pfam00135	0.03	0.27	0.21	0.13	0.04	0.19	0.15	0.12	0.08	0.08	Carboxylesterase family
pfam00933	0.01			0.17		0.12	0.13	0.17	0.21	0.16	Glycosyl hydrolase family 3 N terminal domain
pfam08016	0.1					0.14	0.15	0.17	0.18	0.5	Polycystin cation channel
pfam00118	0.13	0.16	0.42	0.13	0.25	0.12	0.02	0.02	0.1	0.08	TCP−1/cpn60 chaperonin family
pfam00026	0.06	0.08	0.21	0.13	0.04	0.07	0.11	0.12	0.18	0.21	Eukaryotic aspartyl protease
pfam00295		0.04		0.1	0.04	0.16	0.2	0.17	0.24	0.13	Glycosyl hydrolases family 28
pfam00169	0.19	0.16		0.1	0.04	0.09	0.04	0.12	0.21	0.32	PH domain
pfam13637	0.13	0.04			0.21	0.35	0.29		0.1	0.11	Ankyrin repeats (many copies)
pfam03060	0.01	0.04	0.07	0.13		0.19	0.31	0.25	0.16	0.03	Nitronate monooxygenase
pfam00083	0.18	0.2	0.35	0.07	0.12	0.05	0.04	0.02	0.08	0.21	Sugar (and other) transporter
pfam08385	0.08	0.12	0.07	0.03	0.08	0.12	0.02	0.12	0.18	0.18	Dynein heavy chain. N−terminal region 1
pfam01408	0.09	0.08		0.1	0.04	0.14	0.13	0.12	0.13	0.03	Oxidoreductase family. NAD−binding Rossmann fold
pfam13540	0.07	0.04				0.12	0.15	0.12	0.18	0.16	Regulator of chromosome condensation (RCC1) repeat
pfam13833	0.09		0.07	0.03	0.04	0.09	0.18	0.15	0.16	0.16	EF−hand domain pair
pfam03016	0.12	0.12	0.07	0.2		0.05	0.07	0.07	0.13	0.11	Exostosin family
pfam01915	0.01			0.17		0.12	0.13	0.07	0.21	0.11	Glycosyl hydrolase family 3 C−terminal domain
pfam00211	0.05			0.1	0.12	0.37	0.35	0.1	0.08	0.08	Adenylate and Guanylate cyclase catalytic domain
pfam01485	0.02	0.16	0.07	0.03	0.08	0.09	0.09	0.07	0.1	0.11	IBR domain
pfam02515		0.04		0.17	0.04	0.44	0.37	0.15	0.08	0.08	CoA−transferase family III
pfam12237	0.02		0.07	0.07	0.04	0.09	0.11	0.12	0.1	0.08	Phosphorylated CTD interacting factor 1 WW domain
pfam00728		0.08	0.07	0.03		0.07	0.15	0.17	0.24	0.08	Glycosyl hydrolase family 20. catalytic domain
pfam02065				0.13		0.07	0.09	0.07	0.16	0.13	Melibiase
pfam00583	0.08	0.04	0.07	0.1		0.07	0.09	0.02	0.03	0.16	Acetyltransferase (GNAT) family
pfam09286	0.01			0.03		0.19	0.15	0.2	0.21	0.11	Pro−kumamolisin. activation domain
pfam00144	0.01			0.1		0.3	0.15	0.07	0.16	0.05	Beta−lactamase
pfam01391					0.04	0.26	0.15	0.07	0.1	0.18	Collagen triple helix repeat (20 copies)
pfam11527	0.03	0.04	0.07			0.07	0.07	0.05	0.08	0.18	The ARF−like 2 binding protein BART
pfam03151	0.17	0.23	0.21	0.03		0.02	0.07	0.05	0.03	0.05	Triose−phosphate Transporter family
pfam01434	0.05	0.08	0.14	0.07	0.04	0.05	0.04	0.02	0.05	0.03	Peptidase family M41
pfam11028		0.04	0.14	0.07	0.29	0.05		0.05	0.08	0.03	Protein of unknown function (DUF2723)
pfam00066	0.02	0.04			0.04	0.84	0.46	0.25	0.18	0.05	LNR domain
pfam01074	0.01	0.04		0.03		0.12	0.13	0.07	0.13	0.05	Glycosyl hydrolases family 38 N−terminal domain
pfam03382		0.16	0.21	0.67	0.08	0.05	0.04		0.03		Mycoplasma protein of unknown function. DUF285
pfam00488	0.08	0.08	0.07	0.03	0.12		0.04	0.02	0.03	0.03	MutS domain V
pfam00620	0.03			0.03	0.04	0.12	0.07	0.02	0.13	0.11	RhoGAP domain
pfam01764	0.22	0.04	0.07	0.03		0.07	0.07	0.02	0.03	0.03	Lipase (class 3)
pfam13088		0.04		0.13		0.02	0.02	0.05	0.08	0.08	BNR repeat−like domain
pfam01833	0.02	0.04				0.12	0.2		0.13	0.18	IPT/TIG domain
pfam05592				0.03		0.14	0.11	0.1	0.05	0.03	Bacterial alpha−L−rhamnosidase
pfam12848	0.07	0.08		0.03			0.07		0.05	0.03	ABC transporter

Supplementary Table 1. Most abundant Pfam domains annotated in the SAG co-assembled genomes and in the *Ectocarpus siliculosus* genome (as an example of photosynthetic stramenopile) and MAST-4D. Red and orange indicate the most abundant Pfam domains, and grey indicates complete absence of the domain in the annotated proteins. The values represent percentages of total Pfam domains.

	GH (number)	GH (% of gene models)	Potentially secreted	Putative algal cell wall degrading enzymes	Associated algal DNA (number of cells positive / total number of cells)
**MAST-4A1**	91	1.1%	39	8	Micromonas (1/4)
**MAST-4A2**	101	1.1%	44	10	Micromonas, Bathycoccus (1/5)
**MAST-4C**	73	1.3%	38	7	none
**MAST-4E**	98	2.1%	67	9	Pelagomonas (1/9)
**MAST-3A**	76	2.3%	33	3	none
**MAST-3F**	13	0.5%	2	1	none
**ChrysophyteH1**	23	0.8%	12	1	none
**ChrysophyteH2**	7	0.4%	3	0	none

Supplementary Table 2. Distribution of glycoside hydrolases (GHs) and algal DNA in the SAG lineages.

	Category 1	Category 2	Category 3	False Positives	Ambiguous	Total
**MAST-3A**	6	1	10	3	2	**22**
**MAST-3F**	1	1	1	1	0	**4**
**MAST-4 A1**	32	15	27	17	7	**98**
**MAST-4 A2**	25	22	23	25	10	**105**
**MAST-4C**	4	16	4	11	3	**38**
**MAST-4E**	2	10	12	7	0	**31**
**Chrysophyte-H1**	5	2	15	13	2	**37**
**Chrysophyte-H2**	2	2	5	1	0	**10**
**Total**	**77** (22.3%)	**69** (20.0%)	**97** (28.1%)	**78** (18.6%)	**24** (6.4%)	**345 (100%)**

Supplementary Table 3. Summary of the validation of putative horizontal gene transfers using a tree-based method. Putative HGTs were classified in three categories, from the most recent events (category 1) to the less recent ones (category 3). Candidate HGTs that were branching with other eukaryotic proteins were considered as False Positives. Ambiguous cases result from poor alignments or insufficient matches.

	Antibiotic and stress resistance	Carbohydrate metabolism	Lipid metabolism	Nitrogen containing molecules	Proteolytic enzymes (peptidase/protease)	Transport
**MAST-4A1**	11	17	3	10	7	2
**MAST-4A2**	8	11	0	6	5	3
**MAST-4C**	6	7	0	3	0	2
**MAST-4E**	2	9	0	1	0	1
**MAST-3A**	1	2	4	2	1	0
**MAST-3F**	0	1	0	0	0	0
**ChrysophyteH1**	3	2	0	2	0	1
**ChrysophyteH2**	1	0	1	0	1	0

Supplementary Table 4. Main categories of HGT enzymes involved in metabolism.

Name	Scientific Name	Taxon ID	Accession numbers
MAST-4 A 1	Stramenopiles sp. TOSAG23-1	1735742	ERR1198936<br>ERR1198938<br>ERR1198948<br>ERR1198949<br>ERR1198925<br>ERR1198954
MAST-4 A 2	Stramenopiles sp. TOSAG23-2	1735743	ERR1138643<br>ERR1138644<br>ERR1138645<br>ERR1138646
MAST-4 C	Stramenopiles sp. TOSAG41-1	1735744	ERR1198926<br>ERR1198940<br>ERR1198945<br>ERR1198955
MAST-4 E	Stramenopiles sp. TOSAG23-3	1735745	ERR1189844<br>ERR1189846<br>ERR1189847<br>ERR1189854<br>ERR1198927<br>ERR1198928<br>ERR1198941<br>ERR1198946<br>ERR1198950
MAST-3 A	Stramenopiles sp. TOSAG41-2	1735746	ERR1198931<br>ERR1198953<br>ERR1198930<br>ERR1198943
MAST-3 F	Stramenopiles sp. TOSAG23-6	1735747	ERR1189848<br>ERR1189852
Chrysophyte H 1	Chrysophyceae sp. TOSAG23-4	1735748	ERR1189849<br>ERR1189855<br>ERR1198924<br>ERR1198933<br>ERR1198934<br>ERR1198937<br>ERR1198951<br>ERR1198956
Chrysophyte H 2	Chrysophyceae sp. TOSAG23-5	1735749	ERR1198929<br>ERR1198935<br>ERR1198944

Supplementary Table 5. Scientifc name, taxon ID and accession numbers at the European Nucleotide Archive of each Single-cell Amplified Genome.

Genome	Number of models	Calibrated on
**ChrysophyteH1**	428	Chrysophyte-H1
**ChrysophyteH2**	243	Chrysophyte-H2
**MAST-3A**	111	MAST-3A
**MAST-3F**	111	MAST-3A
**MAST-4A1**	370	MAST-4A1
**MAST-4A2**	393	MAST-4A2
**MAST-4C**	226	MAST-4C
**MAST-4E**	188	MAST-4E

Supplementary Table 6. Number of complete models used to train SNAP and source of calibration.
