# Supplementary material for: Single-cell genomics of multiple uncultured stramenopiles reveals underestimated functional diversity across oceans
Source: Nat Commun. 2018 Jan 22;9:310. doi: 10.1038/s41467-017-02235-3 (PMC5778133; doi:10.1038/s41467-017-02235-3)
Supplement: Supplementary file 3 — Description of Additional Supplementary Files [file 41467_2017_2235_MOESM3_ESM.pdf]

Description of Additional Supplementary Files

File Name: Supplementary Data 1
Description: Methodological context for metagenomic samples.

File Name: Supplementary Data 2
Description: Environmental parameters measured for each metagenomic sample.

File Name: Supplementary Data 3
Description: Name and accession number of metagenomic samples

File Name: Supplementary Data 4
Description: Phylogenetic trees of horizontal gene transfers candidates
