# Supplementary material for: Single-cell genomics of multiple uncultured stramenopiles reveals underestimated functional diversity across oceans
Source: Nat Commun. 2018 Jan 22;9:310. doi: 10.1038/s41467-017-02235-3 (PMC5778133; doi:10.1038/s41467-017-02235-3)
Supplement: Supplementary file 7 — Supplementary Data 4 [file 41467_2017_2235_MOESM7_ESM.pdf]

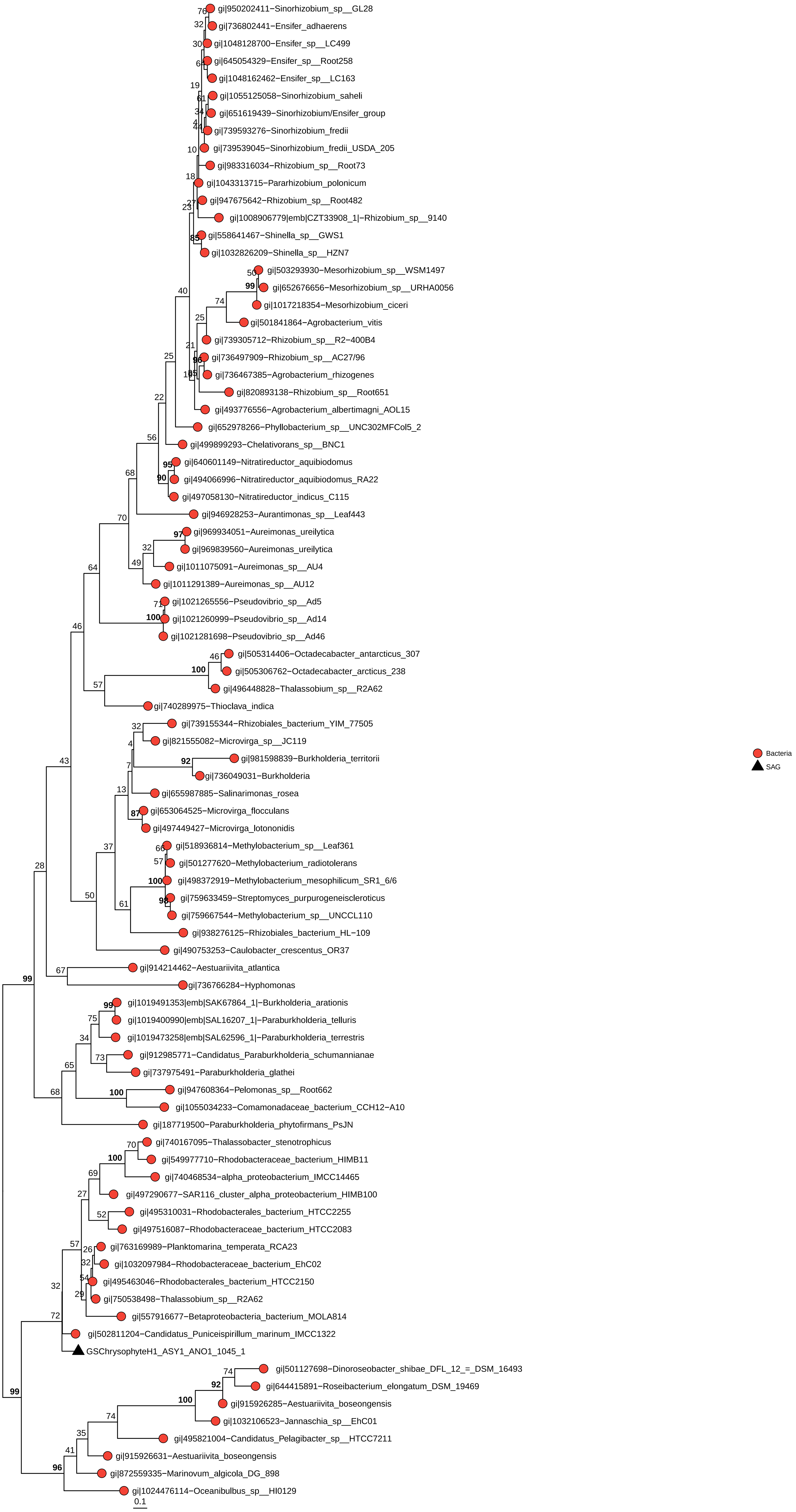
gi|950202411−Sinorhizobium_sp__GL28
gi|736802441−Ensifer_adhaerens
gi|1048128700−Ensifer_sp__LC499
gi|645054329−Ensifer_sp__Root258
gi|1048162462−Ensifer_sp__LC163
gi|1055125058−Sinorhizobium_saheli
gi|651619439−Sinorhizobium/Ensifer_group
gi|739593276−Sinorhizobium_fredii
gi|739539045−Sinorhizobium_fredii_USDA_205
gi|983316034−Rhizobium_sp__Root73
gi|1043313715−Pararhizobium_polonicum
gi|947675642−Rhizobium_sp__Root482
gi|1008906779|emb|CZT33908_1|−Rhizobium_sp__9140
gi|558641467−Shinella_sp__GWS1
gi|1032826209−Shinella_sp__HZN7
gi|503293930−Mesorhizobium_sp__WSM1497
gi|652676656−Mesorhizobium_sp__URHA0056
gi|1017218354−Mesorhizobium_ciceri
gi|501841864−Agrobacterium_vitis
gi|739305712−Rhizobium_sp__R2−400B4
gi|736497909−Rhizobium_sp__AC27/96
gi|736467385−Agrobacterium_rhizogenes
gi|820893138−Rhizobium_sp__Root651
gi|493776556−Agrobacterium_albertimagni_AOL15
gi|652978266−Phyllobacterium_sp__UNC302MFCol5_2
gi|499899293−Chelativorans_sp__BNC1
gi|640601149−Nitratireductor_aquibiodomus
gi|494066996−Nitratireductor_aquibiodomus_RA22
gi|497058130−Nitratireductor_indicus_C115
gi|946928253−Aurantimonas_sp__Leaf443
gi|969934051−Aureimonas_ureilytica
gi|969839560−Aureimonas_ureilytica
gi|1011075091−Aureimonas_sp__AU4
gi|1011291389−Aureimonas_sp__AU12
gi|1021265556−Pseudovibrio_sp__Ad5
gi|1021260999−Pseudovibrio_sp__Ad14
gi|1021281698−Pseudovibrio_sp__Ad46
gi|505314406−Octadecabacter_antarcticus_307
gi|505306762−Octadecabacter_arcticus_238
gi|496448828−Thalassobium_sp__R2A62
gi|740289975−Thioclava_indica
gi|739155344−Rhizobiales_bacterium_YIM_77505
gi|821555082−Microvirga_sp__JC119
gi|981598839−Burkholderia_territorii
gi|736049031−Burkholderia
gi|655987885−Salinarimonas_rosea
gi|653064525−Microvirga_flocculans
gi|497449427−Microvirga_lotononidis
gi|518936814−Methylobacterium_sp__Leaf361
gi|501277620−Methylobacterium_radiotolerans
gi|498372919−Methylobacterium_mesophilicum_SR1_6/6
gi|759633459−Streptomyces_purpurogeneiscleroticus
gi|759667544−Methylobacterium_sp__UNCCL110
gi|938276125−Rhizobiales_bacterium_HL−109
gi|490753253−Caulobacter_crescentus_OR37
gi|914214462−Aestuariivita_atlantica
gi|736766284−Hyphomonas
gi|1019491353|emb|SAK67864_1|−Burkholderia_arationis
gi|1019400990|emb|SAL16207_1|−Paraburkholderia_telluris
gi|1019473258|emb|SAL62596_1|−Paraburkholderia_terrestris
gi|912985771−Candidatus_Paraburkholderia_schumannianae
gi|737975491−Paraburkholderia_glathei
gi|947608364−Pelomonas_sp__Root662
gi|1055034233−Comamonadaceae_bacterium_CCH12−A10
gi|187719500−Paraburkholderia_phytofirmans_PsJN
gi|740167095−Thalassobacter_stenotrophicus
gi|549977710−Rhodobacteraceae_bacterium_HIMB11
gi|740468534−alpha_proteobacterium_IMCC14465
gi|497290677−SAR116_cluster_alpha_proteobacterium_HIMB100
gi|495310031−Rhodobacterales_bacterium_HTCC2255
gi|497516087−Rhodobacteraceae_bacterium_HTCC2083
gi|763169989−Planktomarina_temperata_RCA23
gi|1032097984−Rhodobacteraceae_bacterium_EhC02
gi|495463046−Rhodobacterales_bacterium_HTCC2150
gi|750538498−Thalassobium_sp__R2A62
gi|557916677−Betaproteobacteria_bacterium_MOLA814
gi|502811204−Candidatus_Puniceispirillum_marinum_IMCC1322
GSChrysophyteH1_ASY1_ANO1_1045_1
gi|501127698−Dinoroseobacter_shibae_DFL_12_=_DSM_16493
gi|644415891−Roseibacterium_elongatum_DSM_19469
gi|915926285−Aestuariivita_boseongensis
gi|1032106523−Jannaschia_sp__EhC01
gi|495821004−Candidatus_Pelagibacter_sp__HTCC7211
gi|915926631−Aestuariivita_boseongensis
gi|872559335−Marinovum_algicola_DG_898
gi|1024476114−Oceanibulbus_sp__HI0129
0.1
Bacteria
SAG

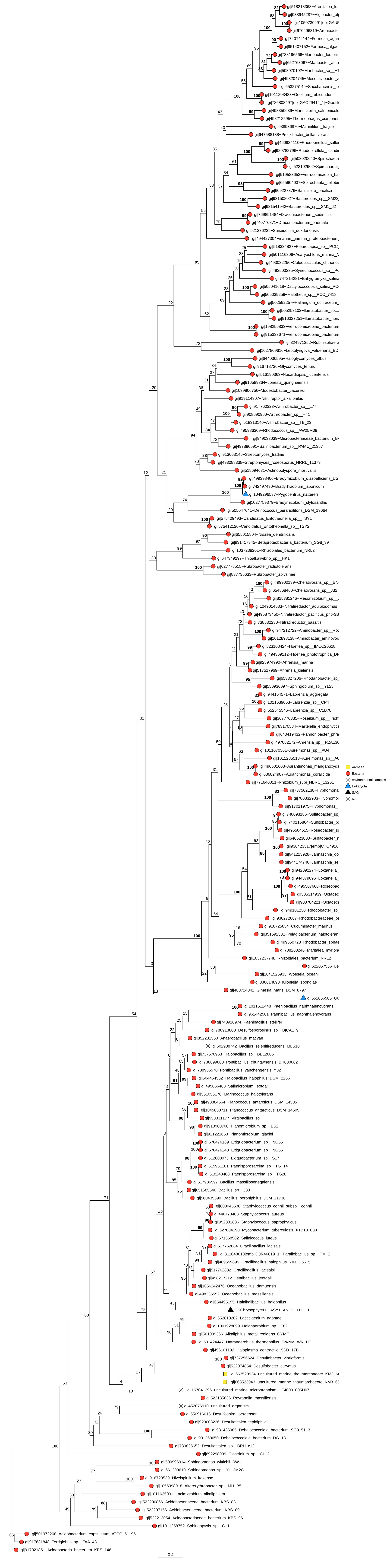
Archaea
Bacteria
environmental samples
Eukaryota
SAG
NA
0.4

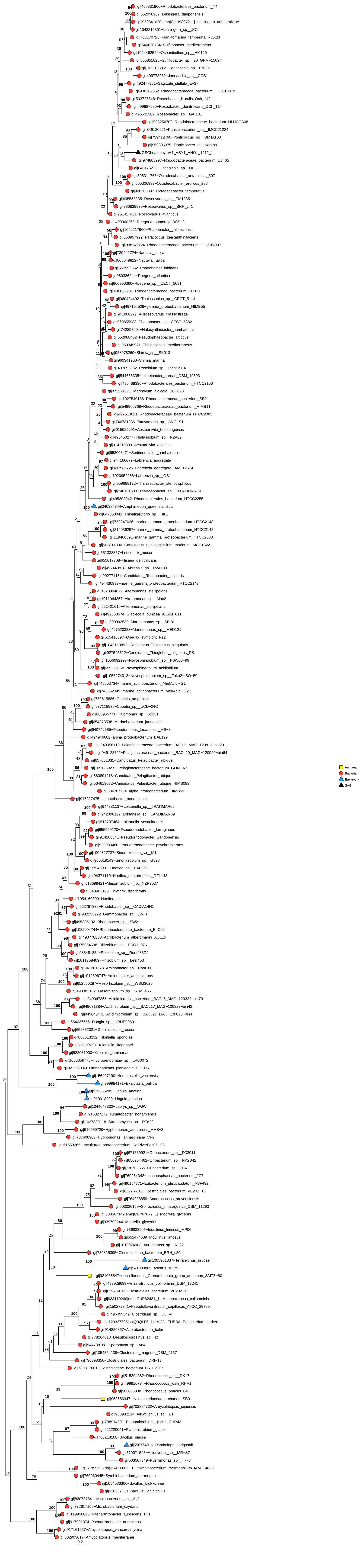
Archaea
Bacteria
Eukaryota
SAG
0.2

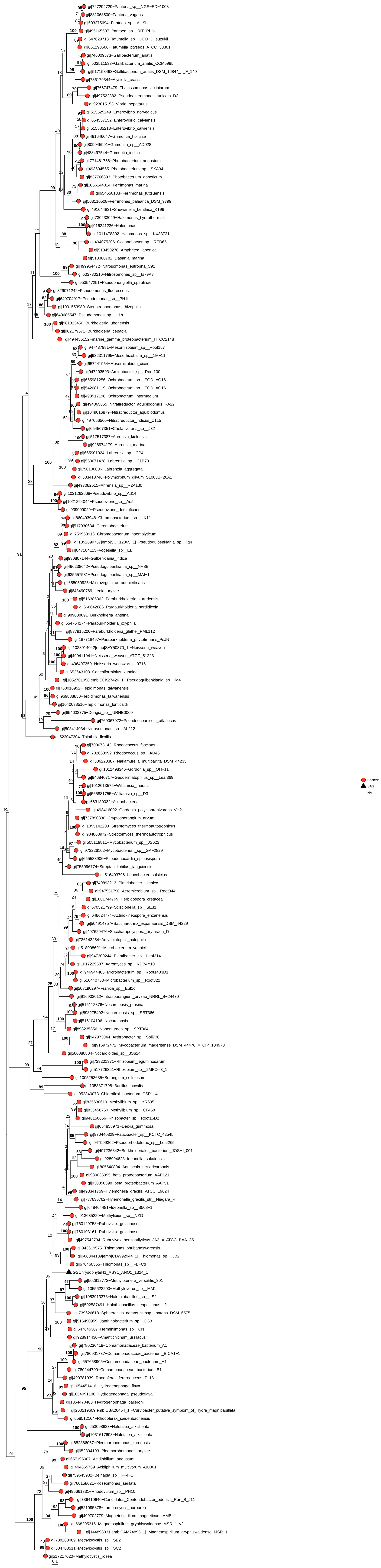

gi|727294729~Pantoea_sp__NGS-ED-1003
gi|881068500~Pantoea_vagans
gi|503275694~Pantoea_sp__At-9b
gi|495165507~Pantoea_sp__RIT-PI-b
gi|647629718~Tatumella_sp__UCD-D_suzukii
gi|661298566~Tatumella_ptyseos_ATCC_33301
gi|746009573~Gallibacterium_anatis
gi|503511533~Gallibacterium_anatis_CCM5995
gi|517158493~Gallibacterium_anatis_DSM_16844_=_F_149
gi|736179344~Alysiella_crassa
gi|766747479~Thalassomonas_actiniarum
gi|497522382~Pseudoalteromonas_tunicata_D2
gi|923015153~Vibrio_hepatarius
gi|515525249~Enterovibrio_norvegicus
gi|654557152~Enterovibrio_calviensis
gi|515585218~Enterovibrio_calviensis
gi|491646047~Grimontia_hollisae
gi|809045991~Grimontia_sp__AD028
gi|488497544~Grimontia_indica
gi|771461756~Photobacterium_angustum
gi|493694565~Photobacterium_sp__SKA34
gi|837766893~Photobacterium_aphoticum
gi|1056144014~Ferrimonas_marina
gi|654650133~Ferrimonas_futtsuensis
gi|503110508~Ferrimonas_balearica_DSM_9799
gi|491644831~Shewanella_benthica_KT99
gi|730433049~Halomonas_hydrothermalis
gi|916241236~Halomonas
gi|1011478302~Halomonas_sp__KX33721
gi|494075206~Oceanobacter_sp__RED65
gi|518450276~Amphritea_japonica
gi|518360782~Dasania_marina
gi|499954472~Nitrosomonas_eutropha_C91
gi|503730210~Nitrosomonas_sp__Is79A3
gi|953547251~Pseudohongiella_spirulinae
gi|829071242~Pseudomonas_fluorescens
gi|640704017~Pseudomonas_sp__PH1b
gi|1001553980~Stenotrophomonas_rhizophila
gi|640685547~Pseudomonas_sp__H1h
gi|981823450~Burkholderia_ubonensis
gi|982179571~Burkholderia_cepacia
gi|494435152~marine_gamma_proteobacterium_HTCC2148
gi|947437981~Mesorhizobium_sp__Root157
gi|932311795~Mesorhizobium_sp__1M-11
gi|657241954~Mesorhizobium_ciceri
gi|947203593~Aminobacter_sp__Root100
gi|665981256~Ochrobactrum_sp__EGD-AQ16
gi|542081119~Ochrobactrum_sp__EGD-AQ16
gi|493512198~Ochrobactrum_intermedium
gi|494065855~Nitratireductor_aquibiodomus_RA22
gi|1049016879~Nitratireductor_aquibiodomus
gi|497056560~Nitratireductor_indicus_C115
gi|654567351~Chelativorans_sp__J32
gi|517517387~Ahrensia_kielensis
gi|928974179~Ahrensia_marina
gi|665901924~Labrenzia_sp__CP4
gi|550671438~Labrenzia_sp__C1B70
gi|750136006~Labrenzia_aggregata
gi|503418740~Polymorphum_gilvum_SL003B-26A1
gi|497082515~Ahrensia_sp__R2A130
gi|1021262668~Pseudovibrio_sp__Ad14
gi|1021264044~Pseudovibrio_sp__Ad5
gi|939009029~Pseudovibrio_denitrificans
gi|860403948~Chromobacterium_sp__LK11
gi|517930634~Chromobacterium
gi|759953913~Chromobacterium_haemolyticum
gi|1052699757|emb|SCK12065_1|~Pseudogulbenkiania_sp__lig4
gi|847184115~Vogesella_sp__EB
gi|930807144~Gulbenkiania_indica
gi|496238642~Pseudogulbenkiania_sp__NH8B
gi|635657581~Pseudogulbenkiania_sp__MAI-1
gi|655050925~Microvirgula_aerodenitrificans
gi|648490769~Leeia_oryzae
gi|516385362~Paraburkholderia_kururiensis
gi|666642686~Paraburkholderia_sordidicola
gi|989088091~Burkholderia_anthina
gi|654764274~Paraburkholderia_oxyphila
gi|837915200~Paraburkholderia_glathei_PML112
gi|187718497~Paraburkholderia_phytofirmans_PsJN
gi|1028914042|emb|SAY50870_1|~Neisseria_weaveri
gi|490411941~Neisseria_weaveri_ATCC_51223
gi|496407359~Neisseria_wadsworthii_9715
gi|652643108~Conchiformibius_kuhniae
gi|1052701958|emb|SCK27426_1|~Pseudogulbenkiania_sp__lig4
gi|760016952~Tepidimonas_taiwanensis
gi|969888850~Tepidimonas_taiwanensis
gi|1040038510~Tepidimonas_fonticaldi
gi|654633775~Dongia_sp__URHE0060
gi|760067972~Pseudooceanicola_atlanticus
gi|503414034~Nitrosomonas_sp__AL212
gi|522047304~Thiothrix_flexilis
gi|700673142~Rhodococcus_fascians
gi|702668992~Rhodococcus_sp__AD45
gi|506228387~Nakamurella_multipartita_DSM_44233
gi|1011498346~Gordonia_sp__QH-11
gi|946840717~Geodermatophilus_sp__Leaf369
gi|1012013575~Williamsia_muralis
gi|565881755~Williamsia_sp__D3
gi|663130032~Actinobacteria
gi|493416002~Gordonia_polyisoprenivorans_VH2
gi|737890830~Cryptosporangium_arvum
gi|1055142203~Streptomyces_thermoautotrophicus
gi|984863972~Streptomyces_thermoautotrophicus
gi|505119811~Mycobacterium_sp__JS623
gi|973226102~Mycobacterium_sp__GA-2829
gi|655588906~Pseudonocardia_spinosispora
gi|755096774~Streptacidiphilus_jiangxiensis
gi|516403796~Leucobacter_salsicius
gi|740893213~Pimelobacter_simplex
gi|947551790~Aeromicrobium_sp__Root344
gi|1001744759~Herbidospora_cretacea
gi|670521799~Sciscionella_sp__SE31
gi|648624774~Actinokineospora_enzanensis
gi|504914757~Saccharothrix_espanaensis_DSM_44229
gi|497629476~Saccharopolyspora_erythraea_D
gi|736143254~Amycolatopsis_halophila
gi|518008691~Microbacterium_yannicii
gi|947309244~Plantibacter_sp__Leaf314
gi|1017229587~Agromyces_sp__NDB4Y10
gi|946944465~Microbacterium_sp__Root1433D1
gi|516440753~Microbacterium_sp__Root322
gi|503190297~Frankia_sp__EuI1c
gi|916903012~Intrasporangium_oryzae_NRRL_B-24470
gi|516112876~Nocardiopsis_prasina
gi|898275402~Nocardiopsis_sp__SBT366
gi|516104196~Nocardiopsis
gi|898235856~Nonomuraea_sp__SBT364
gi|947973044~Arthrobacter_sp__Soil736
gi|916972472~Mycobacterium_mageritense_DSM_44476_=_CIP_104973
gi|500080804~Nocardioides_sp__JS614
gi|739201371~Rhizobium_leguminosarum
gi|517726351~Rhizobium_sp__2MFCol3_1
gi|1005253635~Sorangium_cellulosum
gi|1053871798~Bacillus_novalis
gi|952340073~Chloroflexi_bacterium_CSP1-4
gi|835630619~Methylibium_sp__YR605
gi|835458760~Methylibium_sp__CF468
gi|948150656~Rhizobacter_sp__Root16D2
gi|654858971~Derxia_gummosa
gi|970440329~Paucibacter_sp__KCTC_42545
gi|947999362~Pseudorhodoferax_sp__Leaf265
gi|497236342~Burkholderiales_bacterium_JOSHI_001
gi|928994623~Ideonella_sakaiensis
gi|805540804~Aquincola_tertiaricarbonis
gi|930035995~beta_proteobacterium_AAP121
gi|930050398~beta_proteobacterium_AAP51
gi|493341759~Hylemonella_gracilis_ATCC_19624
gi|737636762~Hylemonella_gracilis_str__Niagara_R
gi|648404481~Ideonella_sp__B508-1
gi|913635220~Methylibium_sp__NZG
gi|760129758~Rubrivivax_gelatinosus
gi|760103161~Rubrivivax_gelatinosus
gi|497542734~Rubrivivax_benzoatilyticus_JA2_=_ATCC_BAA-35
gi|943619575~Thiomonas_bhubaneswarensis
gi|668344109|emb|CDW92944_1|~Thiomonas_sp__CB2
gi|670460565~Thiomonas_sp__FB-Cd
GSChrysophyteH1_ASY1_ANO1_1324_1
gi|502912772~Methyloversatilis_versatilis_301
gi|1055623200~Methyloversatilis_sp__MM1
gi|1053913373~Halothiobacillus_sp__LS2
gi|502587491~Halothiobacillus_neapolitanus_c2
gi|739626618~Sphaerotilus_natans_subsp__natans_DSM_6575
gi|516490959~Janthinobacterium_sp__CG3
gi|647645307~Herminiimonas_sp__CN
gi|928914430~Amantichitinum_ursilacus
gi|780236418~Comamonadaceae_bacterium_A1
gi|780901737~Comamonadaceae_bacterium_BICA1-1
gi|647658906~Comamonadaceae_bacterium_H1
gi|780244700~Comamonadaceae_bacterium_B1
gi|499781939~Rhodoferax_ferrireducens_T118
gi|1054451416~Hydrogenophaga_flava
gi|1054091108~Hydrogenophaga_pseudoflava
gi|1054470483~Hydrogenophaga_palleronii
gi|260219609|emb|CBA26454_1|~Curvibacter_putative_symbiont_of_Hydra_magnipapillata
gi|658512164~Rhodoferax_saidenbachensis
gi|653098683~Halotalea_alkalilenta
gi|1031617698~Halotalea_alkalilenta
gi|652386067~Pleomorphomonas_koreensis
gi|652394193~Pleomorphomonas_oryzae
gi|657195007~Acidiphilium_angustum
gi|494665769~Acidiphilium_multivorum_AIU301
gi|759645932~Belnapia_sp__F-4-1
gi|760158621~Roseomonas_aerilata
gi|495661331~Rhodovulum_sp__PH10
gi|736410640~Candidatus_Contendobacter_odensis_Run_B_J11
gi|521995878~Lamprocystis_purpurea
gi|499702779~Magnetospirillum_magneticum_AMB-1
gi|568205316~Magnetospirillum_gryphiswaldense_MSR-1_v2
gi|144898031|emb|CAM74895_1|~Magnetospirillum_gryphiswaldense_MSR-1
gi|738288089~Methylocystis_sp__SB2
gi|504703511~Methylocystis_sp__SC2
gi|517217020~Methylocystis_rosea
0.1
Bacteria
SAG
NA

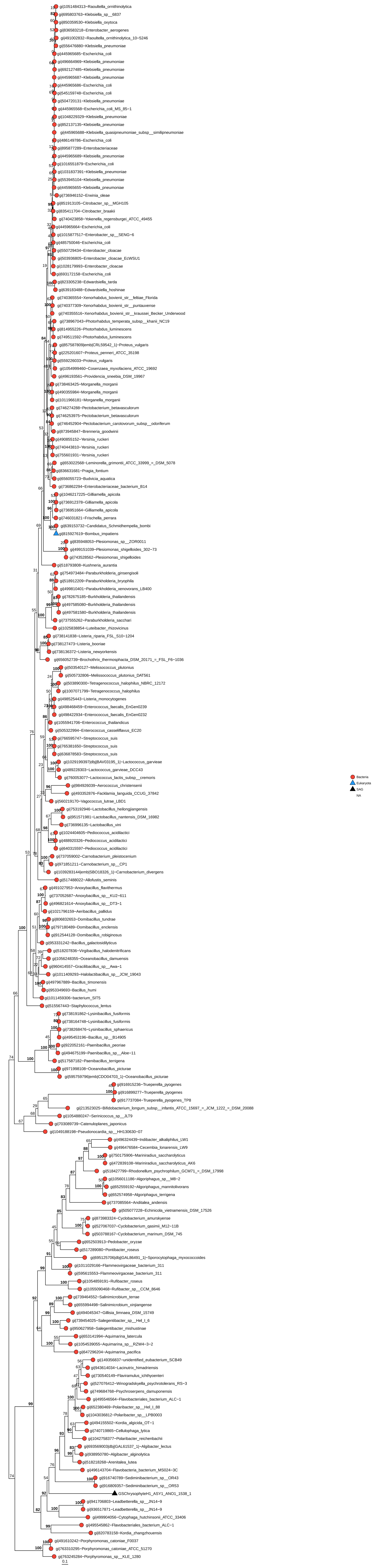

Bacteria
Eukaryota
SAG
NA
0.1

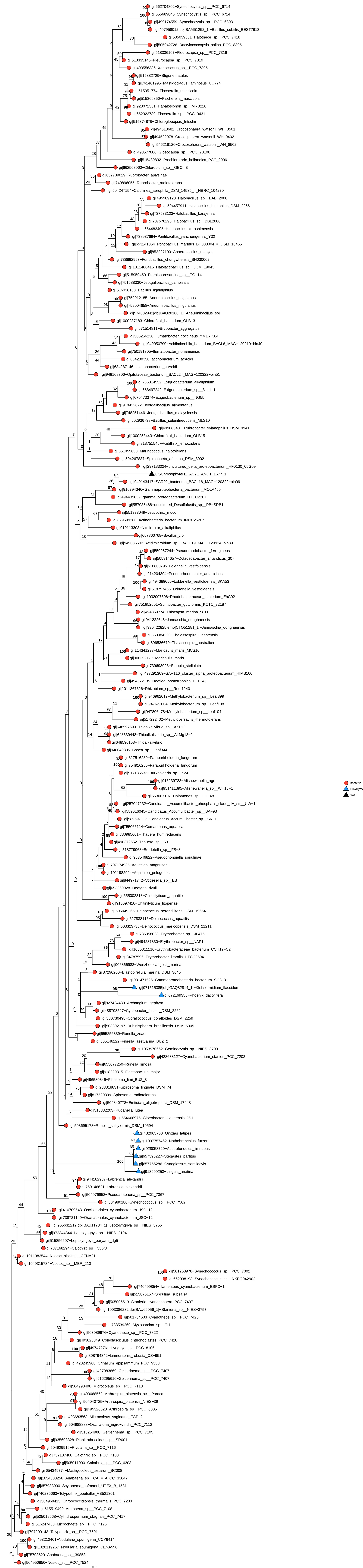
Bacteria
Eukaryota
SAG
0.2

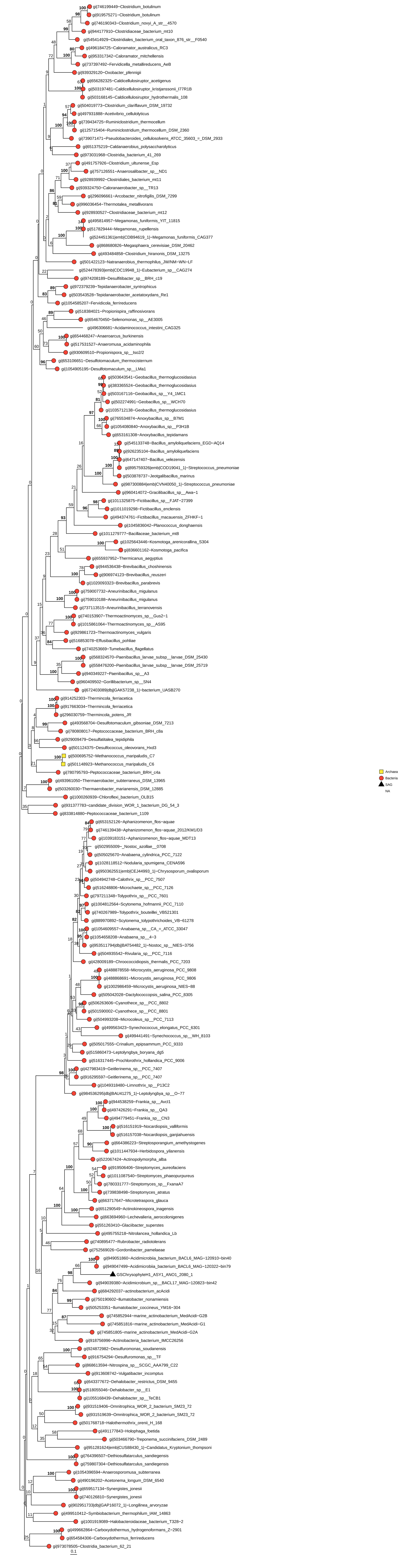
Archaea
Bacteria
SAG
NA
0.1

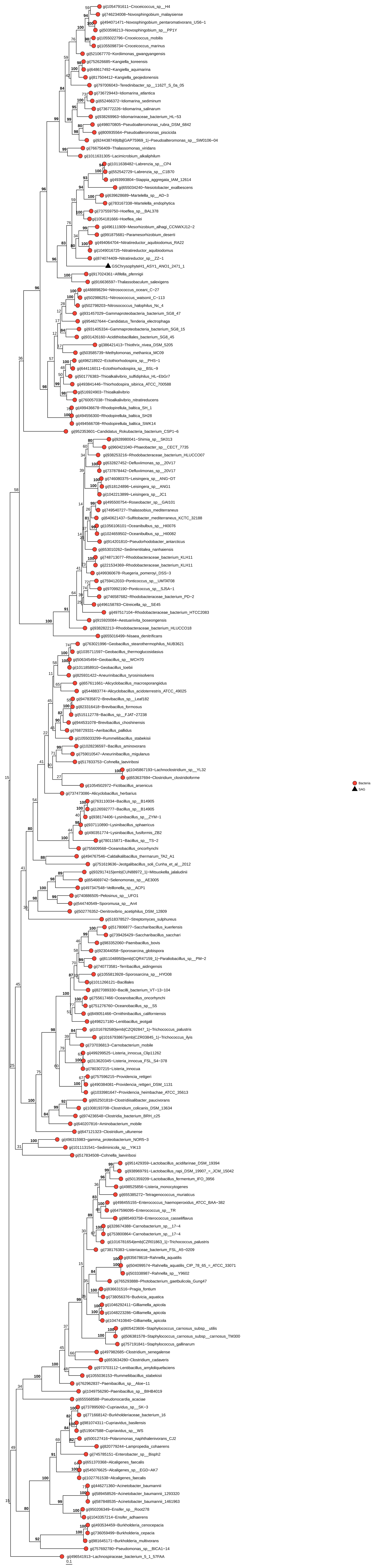
gi|1054791611~Croceicoccus_sp__H4
gi|746234008~Novosphingobium_malaysiense
gi|494071471~Novosphingobium_pentaromativorans_US6~1
gi|503598213~Novosphingobium_sp__PP1Y
gi|1055022796~Croceicoccus_mobilis
gi|1055098734~Croceicoccus_marinus
gi|521067770~Kordiimonas_gwangyangensis
gi|752626685~Kangiella_koreensis
gi|648617492~Kangiella_aquimarina
gi|817504412~Kangiella_geojedonensis
gi|797006043~Teredinibacter_sp__1162T_S_0a_05
gi|736729443~Idiomarina_atlantica
gi|652466372~Idiomarina_sediminum
gi|736772226~Idiomarina_salinarum
gi|938269963~Idiomarinaceae_bacterium_HL~53
gi|498070805~Pseudoalteromonas_rubra_DSM_6842
gi|800935564~Pseudoalteromonas_piscicida
gi|924438749|dbj|GAP75969_1|~Pseudoalteromonas_sp__SW0106~04
gi|766756409~Thalassomonas_viridans
gi|1011631305~Lacimicrobium_alkaliphilum
gi|1011638482~Labrenzia_sp__CP4
gi|552542729~Labrenzia_sp__C1B70
gi|493993804~Stappia_aggregata_IAM_12614
gi|655034240~Nesiotobacter_exalbescens
gi|639628689~Martelella_sp__AD~3
gi|783167338~Martelella_endophytica
gi|737559750~Hoeflea_sp__BAL378
gi|1054181066~Hoeflea_olei
gi|496111909~Mesorhizobium_alhagi_CCNWXJ12~2
gi|991875681~Paramesorhizobium_deserti
gi|494064704~Nitratireductor_aquibiodomus_RA22
gi|1049016725~Nitratireductor_aquibiodomus
gi|874074409~Nitratireductor_sp__ZZ~1
GSChrysophyteH1_ASY1_ANO1_2471_1
gi|917024361~Afifella_pfennigii
gi|916636597~Thalassobaculum_salexigens
gi|488898294~Nitrosococcus_oceani_C~27
gi|502986251~Nitrosococcus_watsonii_C~113
gi|502798203~Nitrosococcus_halophilus_Nc_4
gi|931457029~Gammaproteobacteria_bacterium_SG8_47
gi|954627644~Candidatus_Tenderia_electrophaga
gi|931405334~Gammaproteobacteria_bacterium_SG8_15
gi|931426160~Acidithiobacillales_bacterium_SG8_45
gi|386421413~Thiothrix_nivea_DSM_5205
gi|503585739~Methylomonas_methanica_MC09
gi|496218922~Ectothiorhodospira_sp__PHS~1
gi|644116011~Ectothiorhodospira_sp__BSL~9
gi|501776383~Thioalkalivibrio_sulfidiphilus_HL~EbGr7
gi|493841446~Thiorhodospira_sibirica_ATCC_700588
gi|516924903~Thioalkalivibrio
gi|760057038~Thioalkalivibrio_nitratireducens
gi|494436678~Rhodopirellula_baltica_SH_1
gi|494556300~Rhodopirellula_baltica_SH28
gi|494566708~Rhodopirellula_baltica_SWK14
gi|952353601~Candidatus_Rokubacteria_bacterium_CSP1~6
gi|928980041~Shimia_sp__SK013
gi|960421040~Phaeobacter_sp__CECT_7735
gi|938253216~Rhodobacteraceae_bacterium_HLUCCO07
gi|632827452~Defluviimonas_sp__20V17
gi|737878442~Defluviimonas_sp__20V17
gi|746080375~Leisingera_sp__ANG~DT
gi|518124896~Leisingera_sp__ANG1
gi|1042213899~Leisingera_sp__JC1
gi|495500754~Roseobacter_sp__GAI101
gi|749540727~Thalassobius_mediterraneus
gi|640621437~Sulfitobacter_mediterraneus_KCTC_32188
gi|1056106101~Oceanibulbus_sp__HI0076
gi|1024659502~Oceanibulbus_sp__HI0082
gi|914201810~Pseudorhodobacter_antarcticus
gi|653010262~Sedimentitalea_nanhaiensis
gi|748713077~Rhodobacteraceae_bacterium_KLH11
gi|221534369~Rhodobacteraceae_bacterium_KLH11
gi|499360678~Ruegeria_pomeroyi_DSS~3
gi|759412033~Ponticoccus_sp__UMTAT08
gi|970992190~Ponticoccus_sp__SJ5A~1
gi|746587682~Rhodobacteraceae_bacterium_PD~2
gi|496158783~Citreicella_sp__SE45
gi|497517104~Rhodobacteraceae_bacterium_HTCC2083
gi|915920084~Aestuariivita_boseongensis
gi|938282213~Rhodobacteraceae_bacterium_HLUCCO18
gi|655016499~Nisaea_denitrificans
gi|763021996~Geobacillus_stearothermophilus_NUB3621
gi|1035711597~Geobacillus_thermoglucosidasius
gi|506345494~Geobacillus_sp__WCH70
gi|1011858910~Geobacillus_toebii
gi|825931422~Aneurinibacillus_tyrosinisolvens
gi|657611661~Alicyclobacillus_macrosporangiidus
gi|544883774~Alicyclobacillus_acidoterrestris_ATCC_49025
gi|947835872~Brevibacillus_sp__Leaf182
gi|823316418~Brevibacillus_formosus
gi|515112778~Bacillus_sp__FJAT~27238
gi|944531078~Brevibacillus_choshinensis
gi|768729331~Aeribacillus_pallidus
gi|1055033299~Rummeliibacillus_stabekisii
gi|1028236597~Bacillus_aminovorans
gi|759010547~Aneurinibacillus_migulanus
gi|517833753~Cohnella_laeviribosi
gi|1045867193~Lachnoclostridium_sp__YL32
gi|653637694~Clostridium_clostridioforme
gi|1054502972~Ficticibacillus_arsenicus
gi|737473086~Alicyclobacillus_herbarius
gi|763110034~Bacillus_sp__B14905
gi|126592777~Bacillus_sp__B14905
gi|938174406~Lysinibacillus_sp__ZYM~1
gi|937110890~Lysinibacillus_sphaericus
gi|490351774~Lysinibacillus_fusiformis_ZB2
gi|780175871~Bacillus_sp__TS~2
gi|755609568~Oceanobacillus_oncorhynchi
gi|494767546~Caldalkalibacillus_thermarum_TA2_A1
gi|751619636~Jeotgalibacillus_soli_Cunha_et_al__2012
gi|932917415|emb|CUN88972_1|~Mitsuokella_jalaludinii
gi|654669742~Selenomonas_sp__AE3005
gi|497347548~Veillonella_sp__ACP1
gi|740886505~Pelosinus_sp__UFO1
gi|544740549~Sporomusa_sp__An4
gi|502776352~Denitrovibrio_acetiphilus_DSM_12809
gi|518378527~Streptomyces_sulphureus
gi|517806877~Saccharibacillus_kuerlensis
gi|739426429~Saccharibacillus_sacchari
gi|983352060~Paenibacillus_bovis
gi|923044058~Sporosarcina_globispora
gi|811048950|emb|CQR47159_1|~Paralioobacillus_sp__PM~2
gi|740773581~Terribacillus_aidingensis
gi|1055813928~Sporosarcina_sp__HYO08
gi|1011266121~Bacillales
gi|827089330~Bacilli_bacterium_VT~13~104
gi|755617466~Oceanobacillus_oncorhynchi
gi|751276760~Oceanobacillus_sp__S5
gi|849051466~Ornithinibacillus_californiensis
gi|498217180~Lentibacillus_jeotgali
gi|1016782580|emb|CZQ92847_1|~Trichococcus_palustris
gi|1016793867|emb|CZR03845_1|~Trichococcus_ilyis
gi|737036813~Carnobacterium_mobile
gi|499299525~Listeria_innocua_Clip11262
gi|313620345~Listeria_innocua_FSL_S4~378
gi|780307215~Listeria_innocua
gi|757596215~Providencia_rettgeri
gi|490384081~Providencia_rettgeri_DSM_1131
gi|1033981647~Providencia_heimbachae_ATCC_35613
gi|652501818~Clostridiisalibacter_paucivorans
gi|1008193708~Clostridium_colicanis_DSM_13634
gi|974236548~Clostridia_bacterium_BRH_c25
gi|640207816~Aminobacterium_mobile
gi|647121323~Clostridium_ultunense
gi|496315983~gamma_proteobacterium_NOR5~3
gi|1011131541~Sediminicola_sp__YIK13
gi|517834508~Cohnella_laeviribosi
gi|951429359~Lactobacillus_acidifarinae_DSM_19394
gi|938969791~Lactobacillus_rapi_DSM_19907_=_JCM_15042
gi|501359209~Lactobacillus_fermentum_IFO_3956
gi|498525856~Listeria_monocytogenes
gi|655385272~Tetragenococcus_muriaticus
gi|498455155~Enterococcus_haemoperoxidus_ATCC_BAA~382
gi|647596095~Enterococcus_sp__TR
gi|985493758~Enterococcus_casseliflavus
gi|328674388~Carnobacterium_sp__17~4
gi|753800864~Carnobacterium_sp__17~4
gi|1016781654|emb|CZR01863_1|~Trichococcus_palustris
gi|738176383~Listeriaceae_bacterium_FSL_A5~0209
gi|835678618~Rahnella_aquatilis
gi|504099574~Rahnella_aquatilis_CIP_78_65_=_ATCC_33071
gi|503338987~Rahnella_sp__Y9602
gi|765293880~Photobacterium_gaetbulicola_Gung47
gi|836631516~Pragia_fontium
gi|738056376~Budvicia_aquatica
gi|1046292411~Gilliamella_apicola
gi|1048223286~Gilliamella_apicola
gi|1047410840~Gilliamella_apicola
gi|805423606~Staphylococcus_carnosus_subsp__utilis
gi|506381578~Staphylococcus_carnosus_subsp__carnosus_TM300
gi|757191841~Staphylococcus_gallinarum
gi|497982685~Clostridium_senegalense
gi|653634280~Clostridium_cadaveris
gi|737033112~Lentibacillus_amyloliquefaciens
gi|1055036153~Rummeliibacillus_stabekisii
gi|762962837~Paenibacillus_sp__Aloe~11
gi|1049756290~Paenibacillus_sp__BIHB4019
gi|655568588~Pseudonocardia_acaciae
gi|737895092~Cupriavidus_sp__SK~3
gi|771668142~Burkholderiaceae_bacterium_16
gi|981074311~Cupriavidus_basilensis
gi|519047588~Cupriavidus_sp__WS
gi|500127416~Polaromonas_naphthalenivorans_CJ2
gi|827792444~Lampropedia_cohaerens
gi|745785151~Enterobacter_sp__Bisph2
gi|651370368~Alcaligenes_faecalis
gi|545076625~Alcaligenes_sp__EGD~AK7
gi|1027761538~Alcaligenes_faecalis
gi|446271360~Acinetobacter_baumannii
gi|589458526~Acinetobacter_baumannii_1293320
gi|587848535~Acinetobacter_baumannii_1461963
gi|950206349~Ensifer_sp__Root78
gi|1043357214~Ensifer_adhaerens
gi|493534459~Burkholderia_cenocepacia
gi|736059499~Burkholderia_cepacia
gi|981645171~Burkholderia_multivorans
gi|757692780~Pseudomonas_sp__BICA1~14
gi|496541913~Lachnospiraceae_bacterium_5_1_57FAA
0.1
Bacteria
SAG

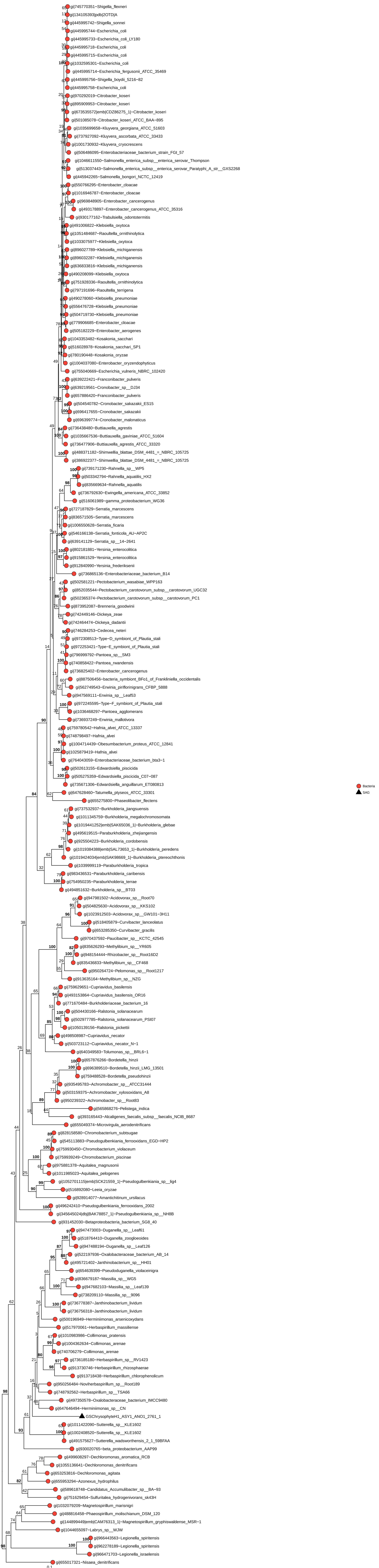
gi|745770351−Shigella_flexneri
gi|134105393|pdb|2OTD|A
gi|445995742−Shigella_sonnei
gi|445995744−Escherichia_coli
gi|445995733−Escherichia_coli_LY180
gi|445995718−Escherichia_coli
gi|445995715−Escherichia_coli
gi|1032595301−Escherichia_coli
gi|445995714−Escherichia_fergusonii_ATCC_35469
gi|445995756−Shigella_boydii_5216−82
gi|445995758−Escherichia_coli
gi|970292019−Citrobacter_koseri
gi|895909953−Citrobacter_koseri
gi|673535572|emb|CDZ86275_1|−Citrobacter_koseri
gi|501085078−Citrobacter_koseri_ATCC_BAA−895
gi|1035699658−Kluyvera_georgiana_ATCC_51603
gi|737927092−Kluyvera_ascorbata_ATCC_33433
gi|1001730932−Kluyvera_cryocrescens
gi|506486095−Enterobacteriaceae_bacterium_strain_FGI_57
gi|1046611550−Salmonella_enterica_subsp__enterica_serovar_Thompson
gi|513037443−Salmonella_enterica_subsp__enterica_serovar_Paratyphi_A_str__GXS2268
gi|445942265−Salmonella_bongori_NCTC_12419
gi|550766295−Enterobacter_cloacae
gi|1016946787−Enterobacter_cloacae
gi|969848905−Enterobacter_cancerogenus
gi|493178897−Enterobacter_cancerogenus_ATCC_35316
gi|930177162−Trabulsiella_odontotermitis
gi|491006822−Klebsiella_oxytoca
gi|1051484687−Raoultella_ornithinolytica
gi|1033075977−Klebsiella_oxytoca
gi|896027789−Klebsiella_michiganensis
gi|896032287−Klebsiella_michiganensis
gi|636833816−Klebsiella_michiganensis
gi|490208099−Klebsiella_oxytoca
gi|751928336−Raoultella_ornithinolytica
gi|797191696−Raoultella_terrigena
gi|490278060−Klebsiella_pneumoniae
gi|556476728−Klebsiella_pneumoniae
gi|504719730−Klebsiella_pneumoniae
gi|779906685−Enterobacter_cloacae
gi|505182229−Enterobacter_aerogenes
gi|1043353482−Kosakonia_sacchari
gi|516028978−Kosakonia_sacchari_SP1
gi|780190448−Kosakonia_oryzae
gi|1004037080−Enterobacter_oryzendophyticus
gi|755040669−Escherichia_vulneris_NBRC_102420
gi|639222421−Franconibacter_pulveris
gi|639219561−Cronobacter_sp__DJ34
gi|657886420−Franconibacter_pulveris
gi|504540782−Cronobacter_sakazakii_ES15
gi|696417655−Cronobacter_sakazakii
gi|696399774−Cronobacter_malonaticus
gi|736438480−Buttiauxella_agrestis
gi|1035667536−Buttiauxella_gaviniae_ATCC_51604
gi|736477906−Buttiauxella_agrestis_ATCC_33320
gi|488371182−Shimwellia_blattae_DSM_4481_=_NBRC_105725
gi|386922377−Shimwellia_blattae_DSM_4481_=_NBRC_105725
gi|739171230−Rahnella_sp__WP5
gi|503342794−Rahnella_aquatilis_HX2
gi|835669634−Rahnella_aquatilis
gi|736792630−Ewingella_americana_ATCC_33852
gi|516061989−gamma_proteobacterium_WG36
gi|727187829−Serratia_marcescens
gi|836571505−Serratia_marcescens
gi|1006550628−Serratia_ficaria
gi|546166138−Serratia_fonticola_AU−AP2C
gi|639141129−Serratia_sp__14−2641
gi|802181881−Yersinia_enterocolitica
gi|915861529−Yersinia_enterocolitica
gi|912840990−Yersinia_frederiksenii
gi|736865136−Enterobacteriaceae_bacterium_B14
gi|502581221−Pectobacterium_wasabiae_WPP163
gi|852035544−Pectobacterium_carotovorum_subsp__carotovorum_UGC32
gi|502365374−Pectobacterium_carotovorum_subsp__carotovorum_PC1
gi|873952087−Brenneria_goodwinii
gi|742449146−Dickeya_zeae
gi|742464474−Dickeya_dadantii
gi|746284253−Cedecea_neteri
gi|972308513−Type−D_symbiont_of_Plautia_stali
gi|972253421−Type−E_symbiont_of_Plautia_stali
gi|796999792−Pantoea_sp__SM3
gi|740858422−Pantoea_rwandensis
gi|736825402−Enterobacter_cancerogenus
gi|887506456−bacteria_symbiont_BFo1_of_Frankliniella_occidentalis
gi|562749543−Erwinia_piriflorinigrans_CFBP_5888
gi|947569111−Erwinia_sp__Leaf53
gi|972245595−Type−F_symbiont_of_Plautia_stali
gi|1036468297−Pantoea_agglomerans
gi|736937249−Erwinia_mallotivora
gi|759780542−Hafnia_alvei_ATCC_13337
gi|748798497−Hafnia_alvei
gi|1004714439−Obesumbacterium_proteus_ATCC_12841
gi|1025879419−Hafnia_alvei
gi|764043059−Enterobacteriaceae_bacterium_bta3−1
gi|502613155−Edwardsiella_piscicida
gi|505275359−Edwardsiella_piscicida_C07−087
gi|735671306−Edwardsiella_anguillarum_ET080813
gi|647628460−Tatumella_ptyseos_ATCC_33301
gi|655275800−Phaseolibacter_flectens
gi|737532937−Burkholderia_jiangsuensis
gi|1011345759−Burkholderia_megalochromosomata
gi|1019441252|emb|SAK65036_1|−Burkholderia_glebae
gi|495619515−Paraburkholderia_zhejiangensis
gi|925504223−Burkholderia_cordobensis
gi|1019384388|emb|SAL73653_1|−Burkholderia_peredens
gi|1019424034|emb|SAK98669_1|−Burkholderia_ptereochthonis
gi|1039999119−Paraburkholderia_tropica
gi|983436531−Paraburkholderia_caribensis
gi|754950235−Paraburkholderia_terrae
gi|494851632−Burkholderia_sp__BT03
gi|947981502−Acidovorax_sp__Root70
gi|504825630−Acidovorax_sp__KKS102
gi|1023912503−Acidovorax_sp__GW101−3H11
gi|518405879−Curvibacter_lanceolatus
gi|653285350−Curvibacter_gracilis
gi|970437592−Paucibacter_sp__KCTC_42545
gi|835626293−Methylibium_sp__YR605
gi|948154444−Rhizobacter_sp__Root16D2
gi|835436833−Methylibium_sp__CF468
gi|950264724−Pelomonas_sp__Root1217
gi|913635164−Methylibium_sp__NZG
gi|759629651−Cupriavidus_basilensis
gi|493153864−Cupriavidus_basilensis_OR16
gi|771670484−Burkholderiaceae_bacterium_16
gi|504430166−Ralstonia_solanacearum
gi|502977785−Ralstonia_solanacearum_PSI07
gi|1050139156−Ralstonia_pickettii
gi|498508987−Cupriavidus_necator
gi|503723112−Cupriavidus_necator_N−1
gi|640349583−Tolumonas_sp__BRL6−1
gi|657876266−Bordetella_hinzii
gi|696389510−Bordetella_hinzii_LMG_13501
gi|759488528−Bordetella_pseudohinzii
gi|935495783−Achromobacter_sp__ATCC31444
gi|503159375−Achromobacter_xylosoxidans_A8
gi|950239322−Achromobacter_sp__Root83
gi|565868276−Pelistega_indica
gi|393165443−Alcaligenes_faecalis_subsp__faecalis_NCIB_8687
gi|655049374−Microvirgula_aerodenitrificans
gi|828158580−Chromobacterium_subtsugae
gi|545113883−Pseudogulbenkiania_ferrooxidans_EGD−HP2
gi|759930450−Chromobacterium_violaceum
gi|759939249−Chromobacterium_piscinae
gi|975881378−Aquitalea_magnusonii
gi|1011985023−Aquitalea_pelogenes
gi|1052701115|emb|SCK21559_1|−Pseudogulbenkiania_sp__lig4
gi|516892080−Leeia_oryzae
gi|928914077−Amantichitinum_ursilacus
gi|496242410−Pseudogulbenkiania_ferrooxidans_2002
gi|345645024|dbj|BAK78857_1|−Pseudogulbenkiania_sp__NH8B
gi|931452030−Betaproteobacteria_bacterium_SG8_40
gi|947473003−Duganella_sp__Leaf61
gi|518764410−Duganella_zoogloeoides
gi|947488194−Duganella_sp__Leaf126
gi|522197936−Oxalobacteraceae_bacterium_AB_14
gi|495721402−Janthinobacterium_sp__HH01
gi|654639399−Pseudoduganella_violaceinigra
gi|836679187−Massilia_sp__WG5
gi|947682103−Massilia_sp__Leaf139
gi|738209110−Massilia_sp__9096
gi|736778387−Janthinobacterium_lividum
gi|736756318−Janthinobacterium_lividum
gi|500196949−Herminiimonas_arsenicoxydans
gi|517970061−Herbaspirillum_massiliense
gi|1010983986−Collimonas_pratensis
gi|1004362634−Collimonas_arenae
gi|740706279−Collimonas_arenae
gi|736185180−Herbaspirillum_sp__RV1423
gi|913730746−Herbaspirillum_rhizosphaerae
gi|913718438−Herbaspirillum_chlorophenolicum
gi|950256484−Noviherbaspirillum_sp__Root189
gi|748792562−Herbaspirillum_sp__TSA66
gi|497350578−Oxalobacteraceae_bacterium_IMCC9480
gi|647646494−Herminiimonas_sp__CN
GSChrysophyteH1_ASY1_ANO1_2761_1
gi|1011422090−Sutterella_sp__KLE1602
gi|1002408520−Sutterella_sp__KLE1602
gi|491575627−Sutterella_wadsworthensis_2_1_59BFAA
gi|930020765−beta_proteobacterium_AAP99
gi|499608297−Dechloromonas_aromatica_RCB
gi|1055136641−Dechloromonas_denitrificans
gi|653253816−Dechloromonas_agitata
gi|655953294−Azonexus_hydrophilus
gi|589618748−Candidatus_Accumulibacter_sp__BA−93
gi|751629454−Sulfuritalea_hydrogenivorans_sk43H
gi|1032079209−Magnetospirillum_marisnigri
gi|488816458−Phaeospirillum_molischianum_DSM_120
gi|144899449|emb|CAM76313_1|−Magnetospirillum_gryphiswaldense_MSR−1
gi|1044655097−Labrys_sp__WJW
gi|966443563−Legionella_spiritensis
gi|962278189−Legionella_spiritensis
gi|966471703−Legionella_israelensis
gi|655017321−Nisaea_denitrificans
0.1
Bacteria
SAG

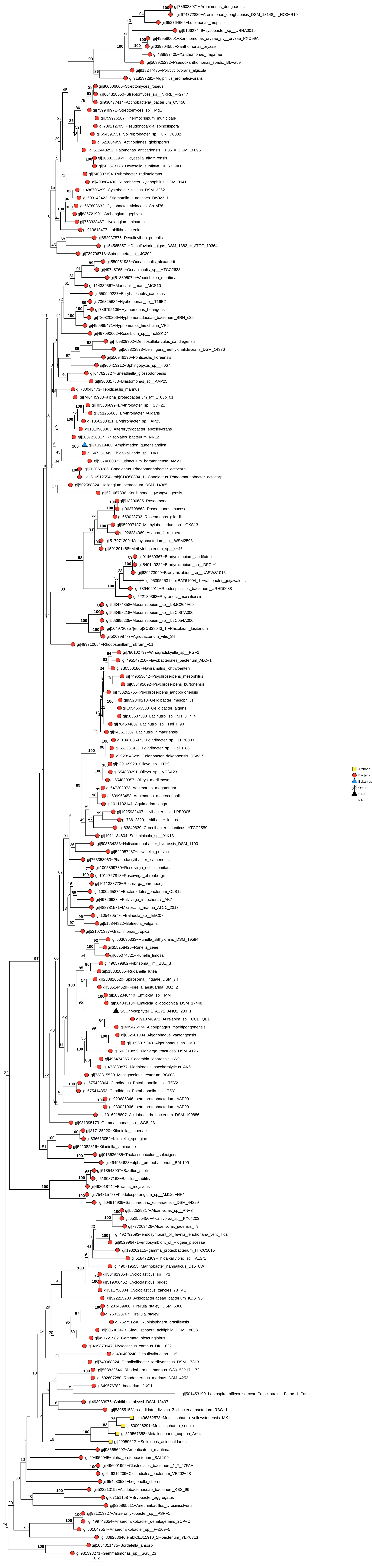

gi|736088071~Arenimonas_donghaensis
gi|674772830~Arenimonas_donghaensis_DSM_18148_=_HO3=R19
gi|652764665~Luteimonas_mephitis
gi|916627449~Lysobacter_sp__URHA0019
gi|499580001~Xanthomonas_oryzae_pv__oryzae_PXO99A
gi|639804555~Xanthomonas_oryzae
gi|488897405~Xanthomonas_fragariae
gi|503925232~Pseudoxanthomonas_spadix_BD=a59
gi|918247435~Polycyclovorans_algicola
gi|918237281~Algiphilus_aromaticivorans
gi|860606006~Streptomyces_roseus
gi|664328550~Streptomyces_sp__NRRL_F=2747
gi|930477414~Actinobacteria_bacterium_OV450
gi|739949971~Streptomyces_sp__Mg1
gi|759975287~Thermocrispum_municipale
gi|739212705~Pseudonocardia_spinosispora
gi|654591531~Solirubrobacter_sp__URHD0082
gi|522004959~Actinoplanes_globisporus
gi|512440252~Halomonas_anticariensis_FP35_=_DSM_16096
gi|1033135969~Hoyosella_altamirensis
gi|503573173~Hoyosella_subflava_DQS3=9A1
gi|740897194~Rubrobacter_radiotolerans
gi|499884430~Rubrobacter_xylanophilus_DSM_9941
gi|488706299~Cystobacter_fuscus_DSM_2262
gi|503142422~Stigmatella_aurantiaca_DW4/3=1
gi|667803632~Cystobacter_violaceus_Cb_vi76
gi|836721901~Archangium_gephyra
gi|763333467~Hyalangium_minutum
gi|913618477~Labilithrix_luteola
gi|652937576~Desulfovibrio_putealis
gi|545653571~Desulfovibrio_gigas_DSM_1382_=_ATCC_19364
gi|739706718~Spirochaeta_sp__JC202
gi|550951986~Oceanicaulis_alexandrii
gi|497487654~Oceanicaulis_sp__HTCC2633
gi|518805074~Woodsholea_maritima
gi|114339567~Maricaulis_maris_MCS10
gi|550949227~Euryhalocaulis_caribicus
gi|736825684~Hyphomonas_sp__T16B2
gi|736795106~Hyphomonas_beringensis
gi|780820206~Hyphomonadaceae_bacterium_BRH_c29
gi|499965471~Hyphomonas_hirschiana_VP5
gi|497090602~Roseibium_sp__TrichSKD4
gi|759809302~Dethiosulfatarculus_sandiegensis
gi|568323873~Leisingera_methylohalidivorans_DSM_14336
gi|550946190~Ponticaulis_koreensis
gi|966413212~Sphingopyxis_sp__H067
gi|647625727~Sneathiella_glossodoripedis
gi|930031788~Blastomonas_sp__AAP25
gi|780043473~Tepidicaulis_marinus
gi|740445983~alpha_proteobacterium_Mf_1_05b_01
gi|493886899~Erythrobacter_sp__SD=21
gi|751255663~Erythrobacter_vulgaris
gi|1056203421~Erythrobacter_sp__AP23
gi|1010968363~Altererythrobacter_epoxidivorans
gi|1037238017~Rhizobiales_bacterium_NRL2
gi|761919480~Amphimedon_queenslandica
gi|647351349~Thioalkalivibrio_sp__HK1
gi|557406087~Lutibaculum_baratangense_AMV1
gi|763069286~Candidatus_Phaeomarinobacter_ectocarpi
gi|610512554|emb|CDO58894_1|~Candidatus_Phaeomarinobacter_ectocarpi
gi|502588824~Haliangium_ochraceum_DSM_14365
gi|521067336~Kordiimonas_gwangyangensis
gi|518290685~Roseomonas
gi|963708868~Roseomonas_mucosa
gi|653028793~Roseomonas_gilardii
gi|959937137~Methylobacterium_sp__GXS13
gi|926284069~Asanoa_ferruginea
gi|517071209~Methylobacterium_sp__WSM2598
gi|501291488~Methylobacterium_sp__4=46
gi|914639367~Bradyrhizobium_viridifuturi
gi|540140222~Bradyrhizobium_sp__DFCI=1
gi|639273949~Bradyrhizobium_sp__UASWS1016
gi|953952531|dbj|BAT61004_1|~Variibacter_gotjawalensis
gi|739402911~Rhodospirillales_bacterium_URHD0088
gi|522189368~Reyranella_massiliensis
gi|563474858~Mesorhizobium_sp__LSJC264A00
gi|563458218~Mesorhizobium_sp__L2C067A000
gi|563995235~Mesorhizobium_sp__L2C054A000
gi|1049720357|emb|SCB38043_1|~Rhizobium_lusitanum
gi|506398777~Agrobacterium_vitis_S4
gi|499710054~Rhodospirillum_rubrum_F11
gi|780102797~Winogradskyella_sp__PG=2
gi|495547210~Flavobacteriales_bacterium_ALC=1
gi|730550186~Flaviramulus_ichthyoenteri
gi|749653642~Psychroserpens_mesophilus
gi|655492092~Psychroserpens_burtonensis
gi|730262755~Psychroserpens_jangbogonensis
gi|652849218~Gelidibacter_mesophilus
gi|1054663500~Gelidibacter_algens
gi|503637300~Lacinutrix_sp__5H=3=7=4
gi|764504607~Lacinutrix_sp__Hel_I_90
gi|943613307~Lacinutrix_himadriensis
gi|1043036473~Polaribacter_sp__LPB0003
gi|652381432~Polaribacter_sp__Hel_I_88
gi|928948289~Polaribacter_dokdonensis_DSW=5
gi|939165923~Olleya_sp__ITB9
gi|654838291~Olleya_sp__VCSA23
gi|654830357~Olleya_marilimosa
gi|647202073~Aquimarina_megaterium
gi|639968453~Aquimarina_macrocephali
gi|1011132141~Aquimarina_longa
gi|1025932467~Ulvibacter_sp__LPB0005
gi|736128291~Altibacter_lentus
gi|83849639~Croceibacter_atlanticus_HTCC2559
gi|1011134604~Sediminicola_sp__YIK13
gi|503534283~Haliscomenobacter_hydrossis_DSM_1100
gi|522057487~Lewinella_persica
gi|763358063~Phaeodactylibacter_xiamenensis
gi|1005899780~Roseivirga_echinicomitans
gi|1011767818~Roseivirga_ehrenbergii
gi|1011388778~Roseivirga_ehrenbergii
gi|1000265874~Bacteroidetes_bacterium_OLB12
gi|497266334~Fulvivirga_imtechensis_AK7
gi|488781571~Microscilla_marina_ATCC_23134
gi|1054305776~Balneola_sp__EhC07
gi|516844822~Balneola_vulgaris
gi|521071397~Gracilimonas_tropica
gi|503695333~Runella_slithyformis_DSM_19594
gi|655258425~Runella_zeae
gi|655074821~Runella_limosa
gi|496579802~Fibrisoma_limi_BUZ_3
gi|518831856~Rudanella_lutea
gi|283816620~Spirosoma_linguale_DSM_74
gi|505144629~Fibrella_aestuarina_BUZ_2
gi|1032340440~Emticicia_sp__MM
gi|504843184~Emticicia_oligotrophica_DSM_17448
GSChrysophyteH1_ASY1_ANO1_283_1
gi|918740972~Aureispira_sp__CCB=QB1
gi|495476974~Algoriphagus_machipongonensis
gi|652561004~Algoriphagus_vanfongensis
gi|1056015348~Algoriphagus_sp__M8=2
gi|503219899~Marivirga_tractuosa_DSM_4126
gi|496474355~Cecembia_lonarensis_LW9
gi|472839877~Mariniradius_saccharolyticus_AK6
gi|738315520~Mastigocoleus_testarum_BC008
gi|575423664~Candidatus_Entotheonella_sp__TSY2
gi|575414852~Candidatus_Entotheonella_sp__TSY1
gi|929685346~beta_proteobacterium_AAP99
gi|930021966~beta_proteobacterium_AAP99
gi|1016918807~Acidobacteria_bacterium_DSM_100886
gi|931395173~Gemmatimonas_sp__SG8_23
gi|817135220~Kiloniella_litopenaei
gi|836613052~Kiloniella_spongiae
gi|522082816~Kiloniella_laminariae
gi|916636985~Thalassobaculum_salexigens
gi|494954623~alpha_proteobacterium_BAL199
gi|518543007~Bacillus_subtilis
gi|518087168~Bacillus_subtilis
gi|498018746~Bacillus_mojavensis
gi|754815777~Kibdelosporangium_sp__MJ126=NF4
gi|504914939~Saccharothrix_espanaensis_DSM_44229
gi|655529817~Alcanivorax_sp__PN=3
gi|652555456~Alcanivorax_sp__KX64203
gi|737263426~Alcanivorax_jadensis_T9
gi|492792593~endosymbiont_of_Tevnia_jerichonana_vent_Tica
gi|952996471~endosymbiont_of_Ridgeia_piscesae
gi|198262115~gamma_proteobacterium_HTCC5015
gi|518472369~Thioalkalivibrio_sp__ALSr1
gi|490719555~Marinobacter_nanhaiticus_D15=8W
gi|504819054~Cycloclasticus_sp__P1
gi|519006452~Cycloclasticus_pugetii
gi|511756804~Cycloclasticus_zancles_78=ME
gi|522215208~Acidobacteriaceae_bacterium_KBS_96
gi|283439980~Pirellula_staleyi_DSM_6068
gi|763323767~Pirellula_staleyi
gi|752751240~Rubinisphaera_brasiliensis
gi|505062473~Singulisphaera_acidiphila_DSM_18658
gi|497721582~Gemmata_obscuriglobus
gi|498870947~Myxococcus_xanthus_DK_1622
gi|496400240~Desulfovibrio_sp__U5L
gi|749068824~Geoalkalibacter_ferrihydriticus_DSM_17813
gi|503832646~Rhodothermus_marinus_SG0_5JP17=172
gi|502607280~Rhodothermus_marinus_DSM_4252
gi|649576782~bacterium_JKG1
gi|501453190~Leptospira_biflexa_serovar_Patoc_strain__Patoc_1_Paris_
gi|493983976~Caldithrix_abyssi_DSM_13497
gi|530551531~candidate_division_Zixibacteria_bacterium_RBG=1
gi|496362578~Metallosphaera_yellowstonensis_MK1
gi|500926291~Metallosphaera_sedula
gi|329567358~Metallosphaera_cuprina_Ar=4
gi|499596221~Sulfolobus_acidocaldarius
gi|935656202~Ardenticatena_maritima
gi|494954945~alpha_proteobacterium_BAL199
gi|496001996~Clostridiales_bacterium_1_7_47FAA
gi|646316209~Clostridiales_bacterium_VE202=28
gi|654930535~Legionella_cherrii
gi|522213192~Acidobacteriaceae_bacterium_KBS_96
gi|671511587~Bryobacter_aggregatus
gi|825865511~Aneurinibacillus_tyrosinisolvens
gi|981213327~Anaeromyxobacter_sp__PSR=1
gi|499742654~Anaeromyxobacter_dehalogenans_2CP=C
gi|501047557~Anaeromyxobacter_sp__Fw109=5
gi|809268646|emb|CEJ11910_1|~bacterium_YEK0313
gi|1054011475~Bordetella_ansorpii
gi|931393271~Gemmatimonas_sp__SG8_23
0.2
Archaea
Bacteria
Eukaryota
Other
SAG
NA

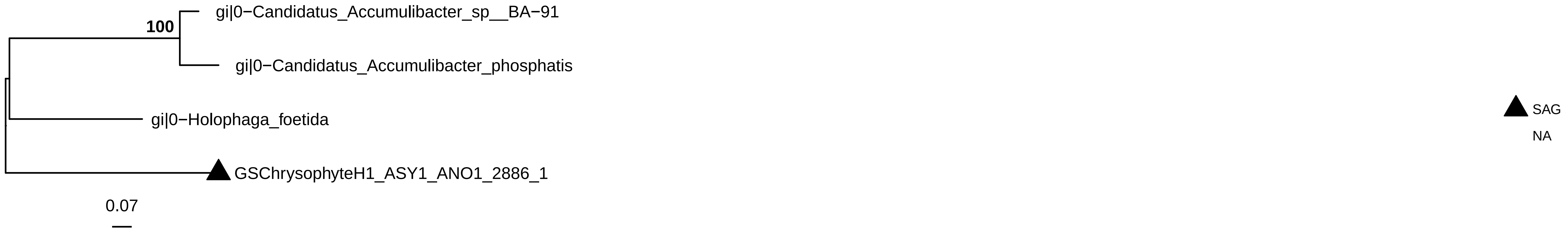
100
gi|0−Candidatus_Accumulibacter_sp__BA−91
gi|0−Candidatus_Accumulibacter_phosphatis
gi|0−Holophaga_foetida
GSChrysophyteH1_ASY1_ANO1_2886_1
0.07
SAG
NA

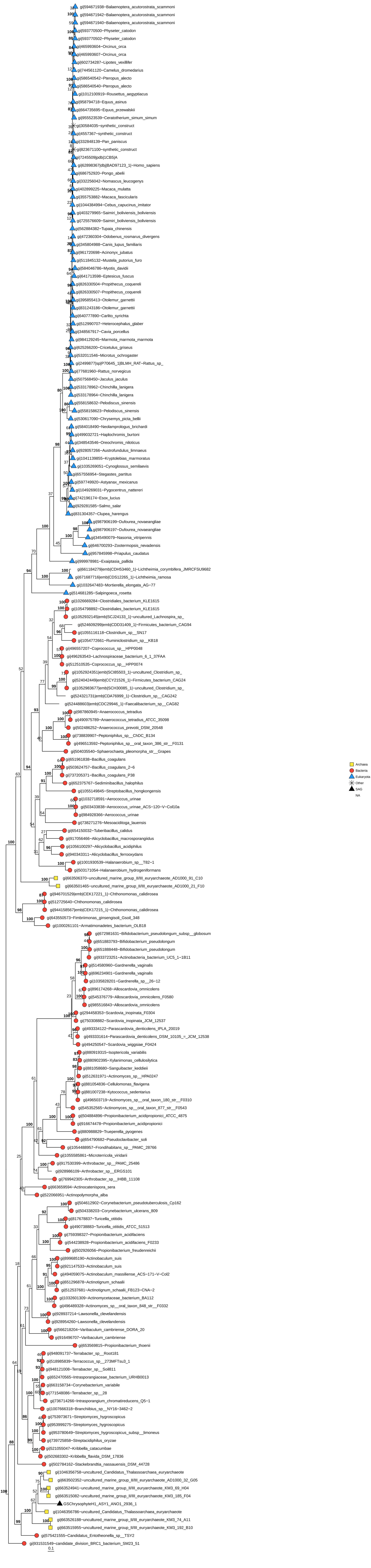

Archaea
Bacteria
Eukaryota
Other
SAG
NA
0.1

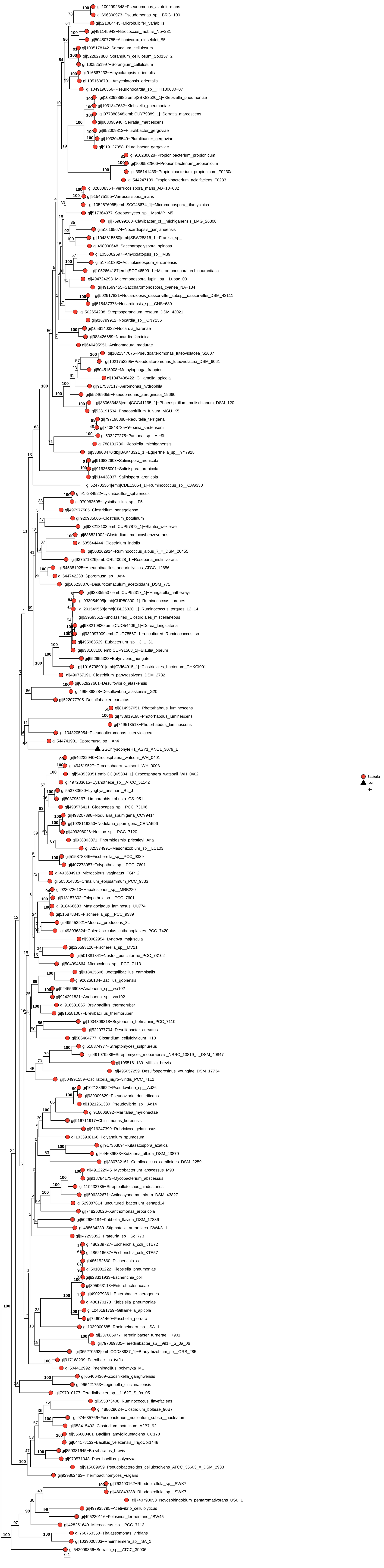

gi|1002992348~Pseudomonas_azotoformans
gi|696300973~Pseudomonas_sp__BRG~100
gi|521084445~Microbulbifer_variabilis
gi|491145943~Nitrococcus_mobilis_Nb~231
gi|504807755~Alcanivorax_dieselolei_B5
gi|1005178142~Sorangium_cellulosum
gi|522827880~Sorangium_cellulosum_So0157~2
gi|1005251997~Sorangium_cellulosum
gi|916567233~Amycolatopsis_orientalis
gi|1051606701~Amycolatopsis_orientalis
gi|1049190366~Pseudonocardia_sp__HH130630~07
gi|1030988985|emb|SBK83520_1|~Klebsiella_pneumoniae
gi|1031847632~Klebsiella_pneumoniae
gi|977888548|emb|CUY79389_1|~Serratia_marcescens
gi|983098940~Serratia_marcescens
gi|852009812~Pluralibacter_gergoviae
gi|1033048549~Pluralibacter_gergoviae
gi|919127058~Pluralibacter_gergoviae
gi|916280028~Propionibacterium_propionicum
gi|1006532806~Propionibacterium_propionicum
gi|395141439~Propionibacterium_propionicum_F0230a
gi|544247109~Propionibacterium_acidifaciens_F0233
gi|328808354~Verrucosispora_maris_AB~18~032
gi|915475155~Verrucosispora_maris
gi|1052676065|emb|SCG48674_1|~Micromonospora_rifamycinica
gi|517364977~Streptomyces_sp__MspMP~M5
gi|759899260~Clavibacter_cf__michiganensis_LMG_26808
gi|516165674~Nocardiopsis_ganjiahuensis
gi|1043615550|emb|SBW28816_1|~Frankia_sp_
gi|498000648~Saccharopolyspora_spinosa
gi|1056062697~Amycolatopsis_sp__M39
gi|517510390~Actinokineospora_enzanensis
gi|1052664187|emb|SCG46599_1|~Micromonospora_echinaurantiaca
gi|494724293~Micromonospora_lupini_str__Lupac_08
gi|491599455~Saccharomonospora_cyanea_NA~134
gi|502917821~Nocardiopsis_dassonvillei_subsp__dassonvillei_DSM_43111
gi|518437378~Nocardiopsis_sp__CNS~639
gi|502654208~Streptosporangium_roseum_DSM_43021
gi|916799912~Nocardia_sp__CNY236
gi|1056140332~Nocardia_harenae
gi|983426689~Nocardia_farcinica
gi|640495951~Actinomadura_madurae
gi|1021347675~Pseudoalteromonas_luteoviolacea_S2607
gi|1021752295~Pseudoalteromonas_luteoviolacea_DSM_6061
gi|504515908~Methylophaga_frappieri
gi|1047408422~Gilliamella_apicola
gi|917537117~Aeromonas_hydrophila
gi|552469655~Pseudomonas_aeruginosa_19660
gi|380683483|emb|CCG41195_1|~Phaeospirillum_molischianum_DSM_120
gi|528191534~Phaeospirillum_fulvum_MGU~K5
gi|797198388~Raoultella_terrigena
gi|740848735~Yersinia_kristensenii
gi|503277275~Pantoea_sp__At~9b
gi|788191736~Klebsiella_michiganensis
gi|338903470|dbj|BAK43321_1|~Eggerthella_sp__YY7918
gi|916832603~Salinispora_arenicola
gi|916365001~Salinispora_arenicola
gi|914438037~Salinispora_arenicola
gi|524705364|emb|CDE13054_1|~Ruminococcus_sp__CAG330
gi|917284922~Lysinibacillus_sphaericus
gi|970962695~Lysinibacillus_sp__F5
gi|497977505~Clostridium_senegalense
gi|920935006~Clostridium_botulinum
gi|933213103|emb|CUP97872_1|~Blautia_wexlerae
gi|636821002~Clostridium_methoxybenzovorans
gi|635644444~Clostridium_indolis
gi|503262914~Ruminococcus_albus_7_=_DSM_20455
gi|937571826|emb|CRL40028_1|~Roseburia_inulinivorans
gi|545381925~Aneurinibacillus_aneurinilyticus_ATCC_12856
gi|544742238~Sporomusa_sp__An4
gi|506238376~Desulfotomaculum_acetoxidans_DSM_771
gi|933359537|emb|CUP92317_1|~Hungatella_hathewayi
gi|933054905|emb|CUP80300_1|~Ruminococcus_torques
gi|291549558|emb|CBL25820_1|~Ruminococcus_torques_L2~14
gi|639693512~unclassified_Clostridiales_miscellaneous
gi|933210820|emb|CUO54406_1|~Dorea_longicatena
gi|932997009|emb|CUO78567_1|~uncultured_Ruminococcus_sp_
gi|495963529~Eubacterium_sp__3_1_31
gi|933168100|emb|CUP91568_1|~Blautia_obeum
gi|652955328~Butyrivibrio_hungatei
gi|1016798901|emb|CVI64915_1|~Clostridiales_bacterium_CHKCI001
gi|490757191~Clostridium_papyrosolvens_DSM_2782
gi|652927601~Desulfovibrio_alaskensis
gi|499686828~Desulfovibrio_alaskensis_G20
gi|522077705~Desulfobacter_curvatus
gi|814957051~Photorhabdus_luminescens
gi|738919198~Photorhabdus_luminescens
gi|749513513~Photorhabdus_luminescens
gi|1048205954~Pseudoalteromonas_luteoviolacea
gi|544741901~Sporomusa_sp__An4
GSChrysophyteH1_ASY1_ANO1_3079_1
gi|546232940~Crocosphaera_watsonii_WH_0401
gi|494519527~Crocosphaera_watsonii_WH_0003
gi|543539351|emb|CCQ65304_1|~Crocosphaera_watsonii_WH_0402
gi|497233615~Cyanothece_sp__ATCC_51142
gi|553733680~Lyngbya_aestuarii_BL_J
gi|808795197~Limnoraphis_robusta_CS~951
gi|493576411~Gloeocapsa_sp__PCC_73106
gi|493207398~Nodularia_spumigena_CCY9414
gi|1028119250~Nodularia_spumigena_CENA596
gi|493306026~Nostoc_sp__PCC_7120
gi|938303071~Phormidesmis_priestleyi_Ana
gi|825374991~Mesorhizobium_sp__LC103
gi|515878346~Fischerella_sp__PCC_9339
gi|407273057~Tolypothrix_sp__PCC_7601
gi|493684918~Microcoleus_vaginatus_FGP~2
gi|505014305~Crinalium_epipsammum_PCC_9333
gi|923072610~Hapalosiphon_sp__MRB220
gi|918157302~Tolypothrix_sp__PCC_7601
gi|918466603~Mastigocladus_laminosus_UU774
gi|515878345~Fischerella_sp__PCC_9339
gi|495453921~Moorea_producens_3L
gi|493036824~Coleofasciculus_chthonoplastes_PCC_7420
gi|50082954~Lyngbya_majuscula
gi|225593120~Fischerella_sp__MV11
gi|501381341~Nostoc_punctiforme_PCC_73102
gi|504994664~Microcoleus_sp__PCC_7113
gi|918425596~Jeotgalibacillus_campisalis
gi|926266134~Bacillus_gobiensis
gi|924656903~Anabaena_sp__wa102
gi|924291831~Anabaena_sp__wa102
gi|916581065~Brevibacillus_thermoruber
gi|916581067~Brevibacillus_thermoruber
gi|1004809318~Scytonema_hofmannii_PCC_7110
gi|522077704~Desulfobacter_curvatus
gi|506404777~Clostridium_cellulolyticum_H10
gi|518374977~Streptomyces_sulphureus
gi|491079286~Streptomyces_mobaraensis_NBRC_13819_=_DSM_40847
gi|1055161189~Millisia_brevis
gi|495057259~Desulfosporosinus_youngiae_DSM_17734
gi|504991559~Oscillatoria_nigro~viridis_PCC_7112
gi|1021286622~Pseudovibrio_sp__Ad26
gi|939009629~Pseudovibrio_denitrificans
gi|1021261380~Pseudovibrio_sp__Ad14
gi|916606692~Maritalea_myrionectae
gi|916711917~Chitinimonas_koreensis
gi|916247399~Rubrivivax_gelatinosus
gi|1033938166~Polyangium_spumosum
gi|917363094~Kitasatospora_azatica
gi|644689533~Kutzneria_albida_DSM_43870
gi|380732161~Corallococcus_coralloides_DSM_2259
gi|491222945~Mycobacterium_abscessus_M93
gi|918784173~Mycobacterium_abscessus
gi|119433785~Streptoalloteichus_hindustanus
gi|506282671~Actinosynnema_mirum_DSM_43827
gi|529087614~uncultured_bacterium_esnapd14
gi|748260026~Xanthomonas_arboricola
gi|502686184~Kribbella_flavida_DSM_17836
gi|488684230~Stigmatella_aurantiaca_DW4/3~1
gi|947295052~Frateuria_sp__Soil773
gi|486239727~Escherichia_coli_KTE72
gi|486216637~Escherichia_coli_KTE57
gi|486152660~Escherichia_coli
gi|501081222~Klebsiella_pneumoniae
gi|823311933~Escherichia_coli
gi|895963118~Enterobacteriaceae
gi|490279361~Enterobacter_aerogenes
gi|486170173~Klebsiella_pneumoniae
gi|1046191759~Gilliamella_apicola
gi|746031460~Frischella_perrara
gi|1039000585~Rheinheimera_sp__SA_1
gi|237685977~Teredinibacter_turnerae_T7901
gi|797069305~Teredinibacter_sp__991H_S_0a_06
gi|365270593|emb|CCD88937_1|~Bradyrhizobium_sp__ORS_285
gi|917168299~Paenibacillus_tyrfis
gi|504412992~Paenibacillus_polymyxa_M1
gi|654064369~Zooshikella_ganghwensis
gi|966421753~Legionella_cincinnatiensis
gi|797010177~Teredinibacter_sp__1162T_S_0a_05
gi|655073408~Ruminococcus_flavefaciens
gi|488629024~Clostridium_bolteae_90B7
gi|974635766~Fusobacterium_nucleatum_subsp__nucleatum
gi|658415492~Clostridium_botulinum_A2B7_92
gi|556600401~Bacillus_amyloliquefaciens_CC178
gi|644178132~Bacillus_velezensis_TrigoCor1448
gi|850381645~Brevibacillus_brevis
gi|970571948~Paenibacillus_polymyxa
gi|915009959~Pseudobacteroides_cellulosolvens_ATCC_35603_=_DSM_2933
gi|929862463~Thermoactinomyces_vulgaris
gi|763400162~Rhodopirellula_sp__SWK7
gi|460843288~Rhodopirellula_sp__SWK7
gi|740790053~Novosphingobium_pentaromativorans_US6~1
gi|497935795~Acetivibrio_cellulolyticus
gi|495230116~Pelosinus_fermentans_JBW45
gi|428251649~Microcoleus_sp__PCC_7113
gi|766763358~Thalassomonas_viridans
gi|1039000803~Rheinheimera_sp__SA_1
gi|542099866~Serratia_sp__ATCC_39006
Bacteria
SAG
NA
0.1

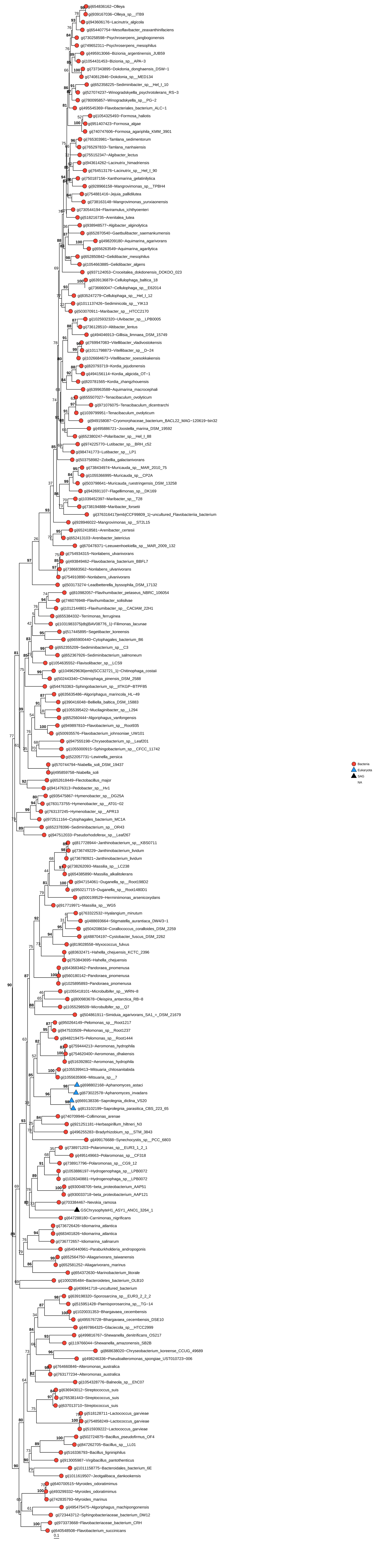

gi|654836162~Olleya
gi|939167036~Olleya_sp__ITB9
gi|943606176~Lacinutrix_algicola
gi|654407754~Mesoflavibacter_zeaxanthinifaciens
gi|730258598~Psychroserpens_jangbogonensis
gi|749652311~Psychroserpens_mesophilus
gi|495913066~Bizionia_argentinensis_JUB59
gi|1054431453~Bizionia_sp__APA~3
gi|737343895~Dokdonia_donghaensis_DSW~1
gi|740812846~Dokdonia_sp__MED134
gi|652358225~Sediminibacter_sp__Hel_I_10
gi|527074237~Winogradskyella_psychrotolerans_RS~3
gi|780095857~Winogradskyella_sp__PG~2
gi|495545369~Flavobacteriales_bacterium_ALC~1
gi|1054325493~Formosa_haliotis
gi|951407423~Formosa_algae
gi|740747606~Formosa_agariphila_KMM_3901
gi|765303981~Tamlana_sedimentorum
gi|765297833~Tamlana_nanhaiensis
gi|755152347~Algibacter_lectus
gi|943614262~Lacinutrix_himadriensis
gi|764513176~Lacinutrix_sp__Hel_I_90
gi|750187156~Xanthomarina_gelatinilytica
gi|928966158~Mangrovimonas_sp__TPBH4
gi|754881416~Jejuia_pallidilutea
gi|738163148~Mangrovimonas_yunxiaonensis
gi|730544194~Flaviramulus_ichthyoenteri
gi|518216735~Arenitalea_lutea
gi|938948577~Algibacter_alginolytica
gi|652870540~Gaetbulibacter_saemankumensis
gi|498209180~Aquimarina_agarivorans
gi|656263549~Aquimarina_agarilytica
gi|652850842~Gelidibacter_mesophilus
gi|1054663885~Gelidibacter_algens
gi|937124053~Croceitalea_dokdonensis_DOKDO_023
gi|639136879~Cellulophaga_baltica_18
gi|736660047~Cellulophaga_sp__E62014
gi|835247279~Cellulophaga_sp__Hel_I_12
gi|1011137426~Sediminicola_sp__YIK13
gi|503070911~Maribacter_sp__HTCC2170
gi|1025932320~Ulvibacter_sp__LPB0005
gi|736128510~Altibacter_lentus
gi|494046913~Gillisia_limnaea_DSM_15749
gi|769947083~Vitellibacter_vladivostokensis
gi|1011798873~Vitellibacter_sp__D~24
gi|1026684673~Vitellibacter_soesokkakensis
gi|820793719~Kordia_jejudonensis
gi|494156114~Kordia_algicida_OT~1
gi|820781565~Kordia_zhangzhouensis
gi|639963588~Aquimarina_macrocephali
gi|655507027~Tenacibaculum_ovolyticum
gi|971076075~Tenacibaculum_dicentrarchi
gi|1039799951~Tenacibaculum_ovolyticum
gi|949158087~Cryomorphaceae_bacterium_BACL22_MAG~120619~bin32
gi|495886721~Joostella_marina_DSM_19592
gi|652380247~Polaribacter_sp__Hel_I_88
gi|974225770~Lutibacter_sp__BRH_c52
gi|984741773~Lutibacter_sp__LP1
gi|503758982~Zobellia_galactanivorans
gi|738434974~Muricauda_sp__MAR_2010_75
gi|1055366995~Muricauda_sp__CP2A
gi|503798641~Muricauda_ruestringensis_DSM_13258
gi|942691107~Flagellimonas_sp__DK169
gi|1039452397~Maribacter_sp__T28
gi|738194888~Maribacter_forsetii
gi|376316417|emb|CCF99809_1|~uncultured_Flavobacteriia_bacterium
gi|928946022~Mangrovimonas_sp__ST2L15
gi|652418581~Arenibacter_certesii
gi|652413103~Arenibacter_latericius
gi|670478371~Leeuwenhoekiella_sp__MAR_2009_132
gi|754934315~Nonlabens_ulvanivorans
gi|493849462~Flavobacteria_bacterium_BBFL7
gi|738683562~Nonlabens_ulvanivorans
gi|754910890~Nonlabens_ulvanivorans
gi|503173274~Leadbetterella_byssophila_DSM_17132
gi|810982057~Flavihumibacter_petaseus_NBRC_106054
gi|746076948~Flavihumibacter_solisilvae
gi|1012144801~Flavihumibacter_sp__CACIAM_22H1
gi|655384332~Terrimonas_ferruginea
gi|1031983375|dbj|BAV08776_1|~Filimonas_lacunae
gi|517445895~Segetibacter_koreensis
gi|665900440~Cytophagales_bacterium_B6
gi|652355209~Sediminibacterium_sp__C3
gi|652367926~Sediminibacterium_salmoneum
gi|1054635552~Flavisolibacter_sp__LCS9
gi|1049629636|emb|SCC32721_1|~Chitinophaga_costaii
gi|502443340~Chitinophaga_pinensis_DSM_2588
gi|544763363~Sphingobacterium_sp__IITKGP~BTPF85
gi|635635486~Algoriphagus_marincola_HL~49
gi|390416048~Belliella_baltica_DSM_15883
gi|1055395422~Mucilaginibacter_sp__L294
gi|652560444~Algoriphagus_vanfongensis
gi|949897810~Flavobacterium_sp__Root935
gi|500935576~Flavobacterium_johnsoniae_UW101
gi|947555198~Chryseobacterium_sp__Leaf201
gi|1055000915~Sphingobacterium_sp__CFCC_11742
gi|522057731~Lewinella_persica
gi|570744794~Niabella_soli_DSM_19437
gi|495859758~Niabella_soli
gi|652618449~Flectobacillus_major
gi|941476313~Pedobacter_sp__Hv1
gi|935475867~Hymenobacter_sp__DG25A
gi|783173755~Hymenobacter_sp__AT01~02
gi|763137245~Hymenobacter_sp__APR13
gi|972511164~Cytophagales_bacterium_MC1A
gi|652378396~Sediminibacterium_sp__OR43
gi|947512033~Pseudorhodoferax_sp__Leaf267
gi|817728944~Janthinobacterium_sp__KBS0711
gi|736749229~Janthinobacterium_lividum
gi|736780921~Janthinobacterium_lividum
gi|738262093~Massilia_sp__LC238
gi|654385890~Massilia_alkalitolerans
gi|947154061~Duganella_sp__Root198D2
gi|950217715~Duganella_sp__Root1480D1
gi|500199529~Herminiimonas_arsenicoxydans
gi|917719971~Massilia_sp__WG5
gi|763322532~Hyalangium_minutum
gi|488693664~Stigmatella_aurantiaca_DW4/3~1
gi|504208634~Corallococcus_coralloides_DSM_2259
gi|488704197~Cystobacter_fuscus_DSM_2262
gi|819028558~Myxococcus_fulvus
gi|836324712~Hahella_chejuensis_KCTC_2396
gi|753843695~Hahella_chejuensis
gi|643683462~Pandoraea_pnomenusa
gi|560180142~Pandoraea_pnomenusa
gi|1025895893~Pandoraea_pnomenusa
gi|1055418101~Microbulbifer_sp__WRN~8
gi|800983678~Oleispira_antarctica_RB~8
gi|1055298509~Microbulbifer_sp__Q7
gi|504861911~Simiduia_agarivorans_SA1_=_DSM_21679
gi|950264149~Pelomonas_sp__Root1217
gi|947533509~Pelomonas_sp__Root1237
gi|948219475~Pelomonas_sp__Root1444
gi|759444213~Aeromonas_hydrophila
gi|754620400~Aeromonas_dhakensis
gi|516392802~Aeromonas_hydrophila
gi|1055399413~Mitsuaria_chitosanitabida
gi|1055635906~Mitsuaria_sp__7
gi|698802168~Aphanomyces_astaci
gi|673002578~Aphanomyces_invadans
gi|669138336~Saprolegnia_diclina_VS20
gi|813102199~Saprolegnia_parasitica_CBS_223_65
gi|740709946~Collimonas_arenae
gi|921251181~Herbaspirillum_hiltneri_N3
gi|496255283~Bradyrhizobium_sp__STM_3843
gi|499176688~Synechocystis_sp__PCC_6803
gi|738971203~Polaromonas_sp__EUR3_1_2_1
gi|495149663~Polaromonas_sp__CF318
gi|738917796~Polaromonas_sp__CG9_12
gi|1053886197~Hydrogenophaga_sp__LPB0072
gi|1026340881~Hydrogenophaga_sp__LPB0072
gi|930048705~beta_proteobacterium_AAP51
gi|930033718~beta_proteobacterium_AAP121
gi|703384467~Nevskia_ramosa
GSChrysophyteH1_ASY1_ANO1_3264_1
gi|647288180~Carnimonas_nigrificans
gi|736726426~Idiomarina_atlantica
gi|683401826~Idiomarina_atlantica
gi|736772657~Idiomarina_salinarum
gi|640440961~Paraburkholderia_andropogonis
gi|652564750~Aliagarivorans_taiwanensis
gi|652581252~Aliagarivorans_marinus
gi|654372630~Marinobacterium_litorale
gi|1000285484~Bacteroidetes_bacterium_OLB10
gi|406941718~uncultured_bacterium
gi|639198320~Sporosarcina_sp__EUR3_2_2_2
gi|515951428~Paenisporosarcina_sp__TG~14
gi|1020031353~Bhargavaea_cecembensis
gi|495576728~Bhargavaea_cecembensis_DSE10
gi|497864325~Glaciecola_sp__HTCC2999
gi|499816767~Shewanella_denitrificans_OS217
gi|119766044~Shewanella_amazonensis_SB2B
gi|868638020~Chryseobacterium_koreense_CCUG_49689
gi|498246336~Pseudoalteromonas_spongiae_UST010723~006
gi|764660846~Alteromonas_australica
gi|763177234~Alteromonas_australica
gi|1054328776~Balneola_sp__EhC07
gi|636943012~Streptococcus_suis
gi|765381443~Streptococcus_suis
gi|637013710~Streptococcus_suis
gi|518128711~Lactococcus_garvieae
gi|754858249~Lactococcus_garvieae
gi|515932422~Lactococcus_garvieae
gi|502724875~Bacillus_pseudofirmus_OF4
gi|847262705~Bacillus_sp__LL01
gi|516336793~Bacillus_ligniniphilus
gi|913005987~Virgibacillus_pantothenticus
gi|1011158775~Bacteroidales_bacterium_6E
gi|1011619507~Jeotgalibaca_dankookensis
gi|640700515~Myroides_odoratimimus
gi|493299332~Myroides_odoratimimus
gi|742835793~Myroides_marinus
gi|495475475~Algoriphagus_machipongonensis
gi|723443712~Sphingobacteriaceae_bacterium_DW12
gi|973373668~Flavobacteriaceae_bacterium_CRH
gi|640548508~Flavobacterium_succinicans
0.1
Bacteria
Eukaryota
SAG
NA

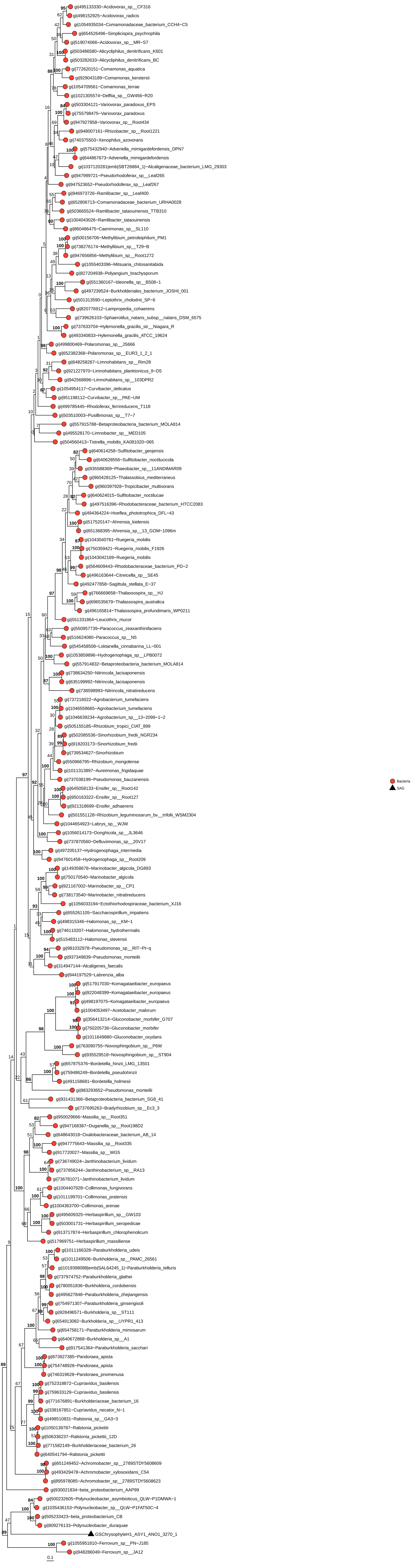

gi|495133330~Acidovorax_sp__CF316
gi|498152925~Acidovorax_radicis
gi|1054935034~Comamonadaceae_bacterium_CCH4~C5
gi|654526496~Simplicispira_psychrophila
gi|519074066~Acidovorax_sp__MR~S7
gi|503486580~Alicycliphilus_denitrificans_K601
gi|503282633~Alicycliphilus_denitrificans_BC
gi|772620151~Comamonas_aquatica
gi|929043189~Comamonas_kerstersii
gi|1054709561~Comamonas_terrae
gi|1021305574~Delftia_sp__GW456~R20
gi|503304121~Variovorax_paradoxus_EPS
gi|755798475~Variovorax_paradoxus
gi|947927858~Variovorax_sp__Root434
gi|948007161~Rhizobacter_sp__Root1221
gi|740375503~Xenophilus_azovorans
gi|575432940~Advenella_mimigardefordensis_DPN7
gi|644867673~Advenella_mimigardefordensis
gi|1037120281|emb|SBT26884_1|~Alcaligenaceae_bacterium_LMG_29303
gi|947999721~Pseudorhodoferax_sp__Leaf265
gi|947523652~Pseudorhodoferax_sp__Leaf267
gi|946973726~Ramlibacter_sp__Leaf400
gi|652806713~Comamonadaceae_bacterium_URHA0028
gi|503665524~Ramlibacter_tataouinensis_TTB310
gi|1004043026~Ramlibacter_tataouinensis
gi|860486475~Caenimonas_sp__SL110
gi|500156706~Methylibium_petroleiphilum_PM1
gi|738276174~Methylibium_sp__T29~B
gi|947656856~Methylibium_sp__Root1272
gi|1055403396~Mitsuaria_chitosanitabida
gi|827204938~Polyangium_brachysporum
gi|551360167~Ideonella_sp__B508~1
gi|497239524~Burkholderiales_bacterium_JOSHI_001
gi|501313590~Leptothrix_cholodnii_SP~6
gi|820776912~Lampropedia_cohaerens
gi|739626103~Sphaerotilus_natans_subsp__natans_DSM_6575
gi|737633704~Hylemonella_gracilis_str__Niagara_R
gi|493340833~Hylemonella_gracilis_ATCC_19624
gi|499800469~Polaromonas_sp__JS666
gi|652382368~Polaromonas_sp__EUR3_1_2_1
gi|648258267~Limnohabitans_sp__Rim28
gi|921227970~Limnohabitans_planktonicus_II~D5
gi|942568896~Limnohabitans_sp__103DPR2
gi|1054954117~Curvibacter_delicatus
gi|951198112~Curvibacter_sp__PAE~UM
gi|499785445~Rhodoferax_ferrireducens_T118
gi|503510003~Pusillimonas_sp__T7~7
gi|557915788~Betaproteobacteria_bacterium_MOLA814
gi|495528170~Limnobacter_sp__MED105
gi|504560413~Tistrella_mobilis_KA081020~065
gi|640614258~Sulfitobacter_geojensis
gi|640628556~Sulfitobacter_noctilucicola
gi|935588369~Phaeobacter_sp__11ANDIMAR09
gi|960428125~Thalassobius_mediterraneus
gi|960397928~Tropicibacter_multivorans
gi|640624015~Sulfitobacter_noctilucae
gi|497516396~Rhodobacteraceae_bacterium_HTCC2083
gi|494364224~Hoeflea_phototrophica_DFL~43
gi|517520147~Ahrensia_kielensis
gi|651368395~Ahrensia_sp__13_GOM~1096m
gi|1043040761~Ruegeria_mobilis
gi|750359421~Ruegeria_mobilis_F1926
gi|1043042169~Ruegeria_mobilis
gi|564609443~Rhodobacteraceae_bacterium_PD~2
gi|496163644~Citreicella_sp__SE45
gi|492477858~Sagittula_stellata_E~37
gi|766669658~Thalassospira_sp__HJ
gi|696535679~Thalassospira_australica
gi|496165814~Thalassospira_profundimaris_WP0211
gi|551331864~Leucothrix_mucor
gi|550957739~Paracoccus_zeaxanthinifaciens
gi|516624080~Paracoccus_sp__N5
gi|545458508~Loktanella_cinnabarina_LL~001
gi|1053859896~Hydrogenophaga_sp__LPB0072
gi|557914832~Betaproteobacteria_bacterium_MOLA814
gi|738634250~Nitrincola_lacisaponensis
gi|635199992~Nitrincola_lacisaponensis
gi|738598993~Nitrincola_nitratireducens
gi|737216022~Agrobacterium_tumefaciens
gi|1046559665~Agrobacterium_tumefaciens
gi|1046639234~Agrobacterium_sp__13~2099~1~2
gi|505155185~Rhizobium_tropici_CIAT_899
gi|502085536~Sinorhizobium_fredii_NGR234
gi|918203173~Sinorhizobium_fredii
gi|739534627~Sinorhizobium
gi|550966795~Rhizobium_mongolense
gi|1011313897~Aureimonas_frigidaquae
gi|737038199~Pseudomonas_bauzanensis
gi|645058133~Ensifer_sp__Root142
gi|950163322~Ensifer_sp__Root127
gi|921318699~Ensifer_adhaerens
gi|501551128~Rhizobium_leguminosarum_bv__trifolii_WSM2304
gi|1044654923~Labrys_sp__WJW
gi|1056014173~Donghicola_sp__JL3646
gi|737870560~Defluviimonas_sp__20V17
gi|497205137~Hydrogenophaga_intermedia
gi|947601458~Hydrogenophaga_sp__Root209
gi|149358678~Marinobacter_algicola_DG893
gi|750170540~Marinobacter_algicola
gi|921167002~Marinobacter_sp__CP1
gi|738173540~Marinobacter_nitratireducens
gi|1056033194~Ectothiorhodospiraceae_bacterium_XJ16
gi|655261105~Saccharospirillum_impatiens
gi|498315346~Halomonas_sp__KM~1
gi|746110207~Halomonas_hydrothermalis
gi|515483112~Halomonas_stevensii
gi|981032978~Pseudomonas_sp__RIT~PI~q
gi|937349839~Pseudomonas_monteilii
gi|314947144~Alcaligenes_faecalis
gi|944197529~Labrenzia_alba
gi|517917030~Komagataeibacter_europaeus
gi|922048399~Komagataeibacter_europaeus
gi|498197075~Komagataeibacter_europaeus
gi|1004053497~Acetobacter_malorum
gi|356413214~Gluconobacter_morbifer_G707
gi|750205736~Gluconobacter_morbifer
gi|1011649880~Gluconobacter_oxydans
gi|763090755~Novosphingobium_sp__P6W
gi|935529518~Novosphingobium_sp__ST904
gi|657875376~Bordetella_hinzii_LMG_13501
gi|759486249~Bordetella_pseudohinzii
gi|491158681~Bordetella_holmesii
gi|983293652~Pseudomonas_monteilii
gi|931431366~Betaproteobacteria_bacterium_SG8_41
gi|737695263~Bradyrhizobium_sp__Ec3_3
gi|950029666~Massilia_sp__Root351
gi|947168387~Duganella_sp__Root198D2
gi|648643018~Oxalobacteraceae_bacterium_AB_14
gi|947775643~Massilia_sp__Root335
gi|917720027~Massilia_sp__WG5
gi|736749024~Janthinobacterium_lividum
gi|737856244~Janthinobacterium_sp__RA13
gi|736781071~Janthinobacterium_lividum
gi|1004407928~Collimonas_fungivorans
gi|1011199701~Collimonas_pratensis
gi|1004363700~Collimonas_arenae
gi|495609325~Herbaspirillum_sp__GW103
gi|503001731~Herbaspirillum_seropedicae
gi|913717874~Herbaspirillum_chlorophenolicum
gi|517969751~Herbaspirillum_massiliense
gi|1011166328~Paraburkholderia_udeis
gi|1011249506~Burkholderia_sp__YI23
gi|1019398088|emb|SAL64245_1|~Paraburkholderia_telluris
gi|737974752~Paraburkholderia_glathei
gi|780051836~Burkholderia_cordobensis
gi|495627848~Paraburkholderia_zhejiangensis
gi|754971307~Paraburkholderia_ginsengisoli
gi|928496571~Burkholderia_sp__ST111
gi|654913082~Burkholderia_sp__UYPR1_413
gi|654758171~Paraburkholderia_mimosarum
gi|640672868~Burkholderia_sp__A1
gi|917541364~Paraburkholderia_sacchari
gi|873927385~Pandoraea_apista
gi|754748928~Pandoraea_apista
gi|746319628~Pandoraea_pnomenusa
gi|752318872~Cupriavidus_basilensis
gi|759633129~Cupriavidus_basilensis
gi|771676891~Burkholderiaceae_bacterium_16
gi|338167851~Cupriavidus_necator_N~1
gi|498510831~Ralstonia_sp__GA3~3
gi|1050139787~Ralstonia_pickettii
gi|506336237~Ralstonia_pickettii_12D
gi|771582149~Burkholderiaceae_bacterium_26
gi|640541794~Ralstonia_pickettii
gi|651249452~Achromobacter_sp__2789STDY5608609
gi|493429478~Achromobacter_xylosoxidans_C54
gi|895978085~Achromobacter_sp__2789STDY5608623
gi|930021834~beta_proteobacterium_AAP99
gi|500232605~Polynucleobacter_asymbioticus_QLW~P1DMWA~1
gi|1035436153~Polynucleobacter_sp__QLW~P1FAT50C~4
gi|505233423~beta_proteobacterium_CB
gi|809276133~Polynucleobacter_duraquae
GSChrysophyteH1_ASY1_ANO1_3270_1
gi|1055951810~Ferrovum_sp__PN~J185
gi|948286049~Ferrovum_sp__JA12
0.1
Bacteria
SAG

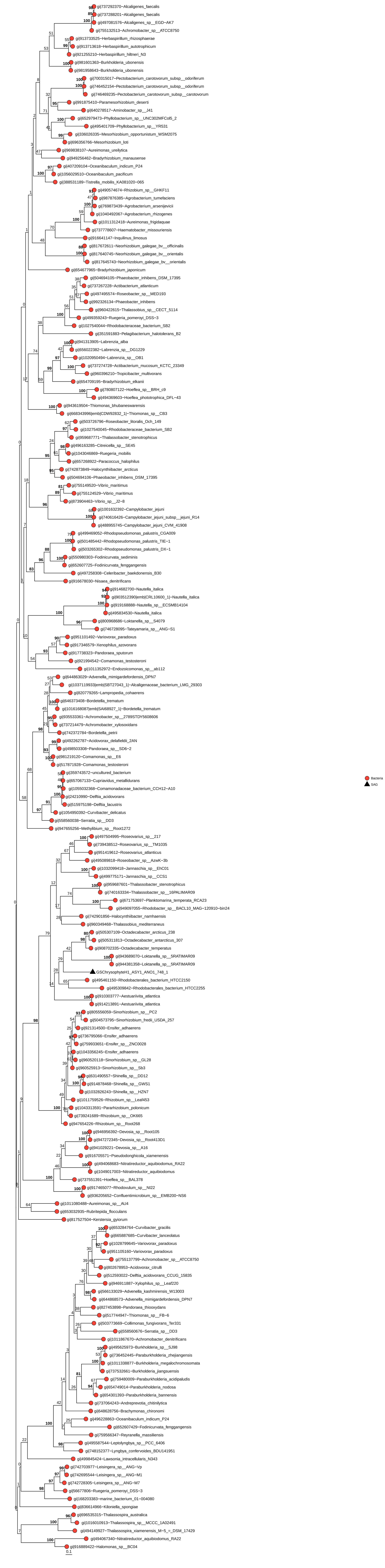

gi|737292370~Alcaligenes_faecalis
gi|737288201~Alcaligenes_faecalis
gi|497081576~Alcaligenes_sp__EGD-AK7
gi|755132513~Achromobacter_sp__ATCC8750
gi|913733525~Herbaspirillum_rhizosphaerae
gi|913713618~Herbaspirillum_autotrophicum
gi|921255210~Herbaspirillum_hiltneri_N3
gi|981601363~Burkholderia_ubonensis
gi|981958643~Burkholderia_ubonensis
gi|700315017~Pectobacterium_carotovorum_subsp__odoriferum
gi|746452154~Pectobacterium_carotovorum_subsp__odoriferum
gi|746469235~Pectobacterium_carotovorum_subsp__carotovorum
gi|991875410~Paramesorhizobium_deserti
gi|640278517~Aminobacter_sp__J41
gi|652979473~Phyllobacterium_sp__UNC302MFCol5_2
gi|495401709~Phyllobacterium_sp__YR531
gi|336026335~Mesorhizobium_opportunistum_WSM2075
gi|696356766~Mesorhizobium_loti
gi|969838107~Aureimonas_ureilytica
gi|949256462~Bradyrhizobium_manausense
gi|407209104~Oceanibaculum_indicum_P24
gi|1056029510~Oceanibaculum_pacificum
gi|388531189~Tistrella_mobilis_KA081020-065
gi|490574674~Rhizobium_sp__GHKF11
gi|998876385~Agrobacterium_tumefaciens
gi|769873439~Agrobacterium_arsenijevicii
gi|1040492067~Agrobacterium_rhizogenes
gi|1011312418~Aureimonas_frigidaquae
gi|737778607~Haematobacter_missouriensis
gi|916641147~Inquilinus_limosus
gi|817672611~Neorhizobium_galegae_bv__officinalis
gi|817640745~Neorhizobium_galegae_bv__orientalis
gi|817645743~Neorhizobium_galegae_bv__orientalis
gi|654677965~Bradyrhizobium_japonicum
gi|504694105~Phaeobacter_inhibens_DSM_17395
gi|737267228~Actibacterium_atlanticum
gi|497495574~Roseobacter_sp__MED193
gi|992326134~Phaeobacter_inhibens
gi|960422615~Thalassobius_sp__CECT_5114
gi|499359243~Ruegeria_pomeroyi_DSS-3
gi|1027540044~Rhodobacteraceae_bacterium_SB2
gi|351591883~Pelagibacterium_halotolerans_B2
gi|941313905~Labrenzia_alba
gi|656022382~Labrenzia_sp__DG1229
gi|1020950494~Labrenzia_sp__OB1
gi|737274728~Actibacterium_mucosum_KCTC_23349
gi|960396210~Tropicibacter_multivorans
gi|654709195~Bradyrhizobium_elkanii
gi|780807122~Hoeflea_sp__BRH_c9
gi|494369603~Hoeflea_phototrophica_DFL-43
gi|943619504~Thiomonas_bhubaneswarensis
gi|668343996|emb|CDW92832_1|~Thiomonas_sp__CB3
gi|503726796~Roseobacter_litoralis_Och_149
gi|1027540045~Rhodobacteraceae_bacterium_SB2
gi|959687771~Thalassobacter_stenotrophicus
gi|496163285~Citreicella_sp__SE45
gi|1043046869~Ruegeria_mobilis
gi|657268922~Paracoccus_halophilus
gi|742873849~Halocynthiibacter_arcticus
gi|504694106~Phaeobacter_inhibens_DSM_17395
gi|755149520~Vibrio_maritimus
gi|755124529~Vibrio_maritimus
gi|873904463~Vibrio_sp__J2-8
gi|1001632392~Campylobacter_jejuni
gi|740616426~Campylobacter_jejuni_subsp__jejuni_R14
gi|488955745~Campylobacter_jejuni_CVM_41908
gi|499469052~Rhodopseudomonas_palustris_CGA009
gi|501485442~Rhodopseudomonas_palustris_TIE-1
gi|503265302~Rhodopseudomonas_palustris_DX-1
gi|550980303~Fodinicurvata_sediminis
gi|652607725~Fodinicurvata_fenggangensis
gi|497258308~Celeribacter_baekdonensis_B30
gi|916678030~Nisaea_denitrificans
gi|914682700~Nautella_italica
gi|903512390|emb|CRL10600_1|~Nautella_italica
gi|919168888~Nautella_sp__ECSMB14104
gi|495834530~Nautella_italica
gi|800968686~Loktanella_sp__S4079
gi|746728095~Tateyamaria_sp__ANG-S1
gi|951101492~Variovorax_paradoxus
gi|917346579~Xenophilus_azovorans
gi|917738323~Pandoraea_sputorum
gi|921994542~Comamonas_testosteroni
gi|1011352972~Endozoicomonas_sp__ab112
gi|644863029~Advenella_mimigardefordensis_DPN7
gi|1037119933|emb|SBT27043_1|~Alcaligenaceae_bacterium_LMG_29303
gi|820779265~Lampropedia_cohaerens
gi|646373408~Bordetella_trematum
gi|1016168087|emb|SAI68927_1|~Bordetella_trematum
gi|935533361~Achromobacter_sp__2789STDY5608606
gi|737214479~Achromobacter_xylosoxidans
gi|742372784~Bordetella_petrii
gi|492262787~Acidovorax_delafieldii_2AN
gi|498503308~Pandoraea_sp__SD6-2
gi|981219120~Comamonas_sp__E6
gi|517871928~Comamonas_testosteroni
gi|359743572~uncultured_bacterium
gi|657067133~Cupriavidus_metallidurans
gi|1050032368~Comamonadaceae_bacterium_CCH12-A10
gi|24210990~Delftia_acidovorans
gi|515975198~Delftia_lacustris
gi|1054950392~Curvibacter_delicatus
gi|558560038~Serratia_sp__DD3
gi|947655256~Methylibium_sp__Root1272
gi|497504995~Roseovarius_sp__217
gi|739438512~Roseovarius_sp__TM1035
gi|951419612~Roseovarius_atlanticus
gi|495089818~Roseobacter_sp__AzwK-3b
gi|1032099418~Jannaschia_sp__EhC01
gi|499775171~Jannaschia_sp__CCS1
gi|959687601~Thalassobacter_stenotrophicus
gi|740163344~Thalassobacter_sp__16PALIMAR09
gi|671753697~Planktomarina_temperata_RCA23
gi|949097055~Rhodobacter_sp__BACL10_MAG-120910-bin24
gi|742901856~Halocynthiibacter_namhaensis
gi|960349468~Thalassobius_mediterraneus
gi|505307109~Octadecabacter_arcticus_238
gi|505311813~Octadecabacter_antarcticus_307
gi|908702335~Octadecabacter_temperatus
gi|943689070~Loktanella_sp__3RATIMAR09
gi|944381358~Loktanella_sp__5RATIMAR09
GSChrysophyteH1_ASY1_ANO1_748_1
gi|495461150~Rhodobacterales_bacterium_HTCC2150
gi|495309842~Rhodobacterales_bacterium_HTCC2255
gi|910303777~Aestuariivita_atlantica
gi|911423891~Aestuariivita_atlantica
gi|805556059~Sinorhizobium_sp__PC2
gi|504573795~Sinorhizobium_fredii_USDA_257
gi|921314500~Ensifer_adhaerens
gi|736795066~Ensifer_adhaerens
gi|759933651~Ensifer_sp__ZNC0028
gi|1043356245~Ensifer_adhaerens
gi|960520118~Sinorhizobium_sp__GL28
gi|960525913~Sinorhizobium_sp__Sb3
gi|631490557~Shinella_sp__DD12
gi|914878468~Shinella_sp__GWS1
gi|1032826243~Shinella_sp__HZN7
gi|1011759526~Rhizobium_sp__Leaf453
gi|1043313591~Pararhizobium_polonicum
gi|739241689~Rhizobium_sp__OK665
gi|947654226~Rhizobium_sp__Root268
gi|946956392~Devosia_sp__Root105
gi|947272345~Devosia_sp__Root413D1
gi|941029221~Devosia_sp__A16
gi|916705571~Pseudodonghicola_xiamenensis
gi|494068683~Nitratireductor_aquibiodomus_RA22
gi|1049017003~Nitratireductor_aquibiodomus
gi|737551391~Hoeflea_sp__BAL378
gi|917465077~Rhodovulum_sp__NI22
gi|936205652~Confluentimicrobium_sp__EMB200-NS6
gi|1011080488~Aureimonas_sp__AU4
gi|653032935~Rubritepida_flocculans
gi|817527504~Kerstersia_gyiorum
gi|653284764~Curvibacter_gracilis
gi|655887685~Curvibacter_lanceolatus
gi|1028799645~Variovorax_paradoxus
gi|951105160~Variovorax_paradoxus
gi|755137799~Achromobacter_sp__ATCC8750
gi|802678953~Acidovorax_citrulli
gi|512593022~Delftia_acidovorans_CCUG_15835
gi|946911887~Xylophilus_sp__Leaf220
gi|566133029~Advenella_kashmirensis_W13003
gi|644868573~Advenella_mimigardefordensis_DPN7
gi|827453898~Pandoraea_thiooxydans
gi|517744947~Thiomonas_sp__FB-6
gi|503773669~Collimonas_fungivorans_Ter331
gi|558560676~Serratia_sp__DD3
gi|1011867670~Achromobacter_denitrificans
gi|495625973~Burkholderia_sp__SJ98
gi|736452445~Paraburkholderia_zhejiangensis
gi|1011338877~Burkholderia_megalochromosomata
gi|737532661~Burkholderia_jiangsuensis
gi|759480009~Paraburkholderia_acidipaludis
gi|654749014~Paraburkholderia_nodosa
gi|654301393~Paraburkholderia_bannensis
gi|737064243~Andreprevotia_chitinilytica
gi|648628756~Brachymonas_chironomi
gi|496228863~Oceanibaculum_indicum_P24
gi|652607429~Fodinicurvata_fenggangensis
gi|759566347~Reyranella_massiliensis
gi|495587544~Leptolyngbya_sp__PCC_6406
gi|748152377~Lyngbya_confervoides_BDU141951
gi|499845424~Lawsonia_intracellularis_N343
gi|742703977~Leisingera_sp__ANG-Vp
gi|742695544~Leisingera_sp__ANG-M1
gi|742728305~Leisingera_sp__ANG-M7
gi|56677806~Ruegeria_pomeroyi_DSS-3
gi|168203383~marine_bacterium_01-004080
gi|836614966~Kiloniella_spongiae
gi|696535315~Thalassospira_australica
gi|1016010913~Thalassospira_sp__MCCC_1A02491
gi|494149927~Thalassospira_xiamenensis_M-5_=_DSM_17429
gi|494067340~Nitratireductor_aquibiodomus_RA22
gi|916889422~Halomonas_sp__BC04
0.1
Bacteria
SAG

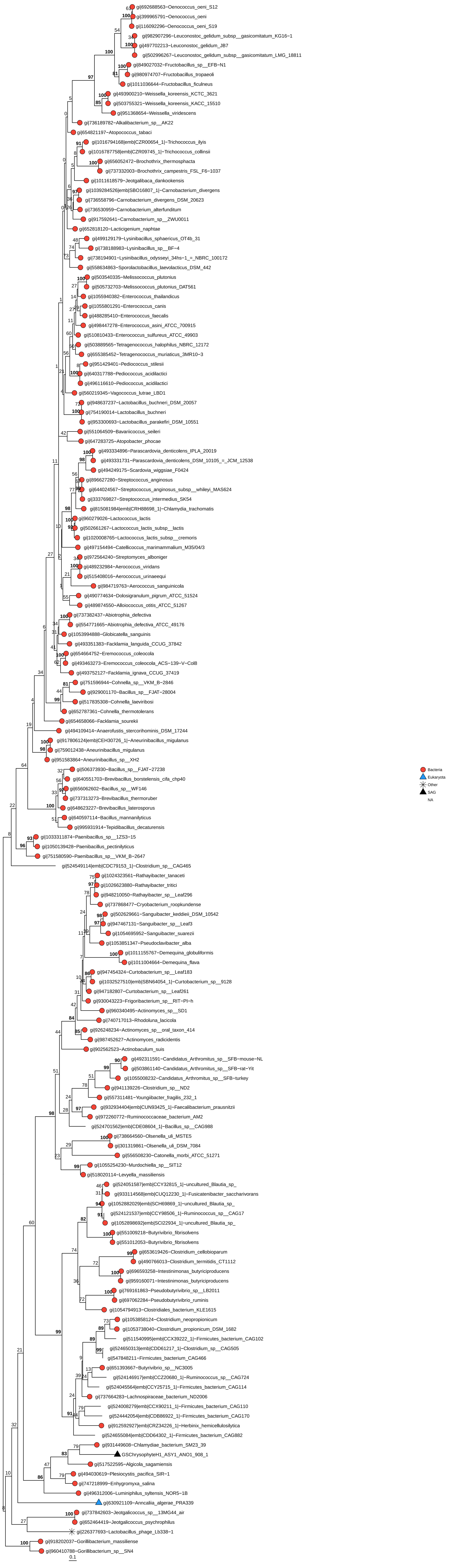
gi|692688563~Oenococcus_oeni_S12
gi|399965791~Oenococcus_oeni
gi|116092296~Oenococcus_oeni_S19
gi|982907296~Leuconostoc_gelidum_subsp__gasicomitatum_KG16~1
gi|497702213~Leuconostoc_gelidum_JB7
gi|502996267~Leuconostoc_gelidum_subsp__gasicomitatum_LMG_18811
gi|849027032~Fructobacillus_sp__EFB~N1
gi|980974707~Fructobacillus_tropaeoli
gi|1011036644~Fructobacillus_ficulneus
gi|493900210~Weissella_koreensis_KCTC_3621
gi|503755321~Weissella_koreensis_KACC_15510
gi|951368654~Weissella_viridescens
gi|736189782~Alkalibacterium_sp__AK22
gi|654821197~Atopococcus_tabaci
gi|1016794168|emb|CZR00654_1|~Trichococcus_ilyis
gi|1016787758|emb|CZR09745_1|~Trichococcus_collinsii
gi|656052472~Brochothrix_thermosphacta
gi|737332003~Brochothrix_campestris_FSL_F6~1037
gi|1011618579~Jeotgalibaca_dankookensis
gi|1039284526|emb|SBO16807_1|~Carnobacterium_divergens
gi|736558796~Carnobacterium_divergens_DSM_20623
gi|736530959~Carnobacterium_alterfunditum
gi|917592641~Carnobacterium_sp__ZWU0011
gi|652818120~Lacticigenium_naphtae
gi|499129179~Lysinibacillus_sphaericus_OT4b_31
gi|738188983~Lysinibacillus_sp__BF~4
gi|738194901~Lysinibacillus_odysseyi_34hs~1_=_NBRC_100172
gi|558634863~Sporolactobacillus_laevolacticus_DSM_442
gi|503540335~Melissococcus_plutonius
gi|505732703~Melissococcus_plutonius_DAT561
gi|1055940382~Enterococcus_thailandicus
gi|1055801291~Enterococcus_canis
gi|488285410~Enterococcus_faecalis
gi|498447278~Enterococcus_asini_ATCC_700915
gi|510810433~Enterococcus_sulfureus_ATCC_49903
gi|503889565~Tetragenococcus_halophilus_NBRC_12172
gi|655385452~Tetragenococcus_muriaticus_3MR10~3
gi|951429401~Pediococcus_stilesii
gi|640317788~Pediococcus_acidilactici
gi|496116610~Pediococcus_acidilactici
gi|560219345~Vagococcus_lutrae_LBD1
gi|948637237~Lactobacillus_buchneri_DSM_20057
gi|754190014~Lactobacillus_buchneri
gi|953300693~Lactobacillus_parakefiri_DSM_10551
gi|551064509~Bavariicoccus_seileri
gi|647283725~Atopobacter_phocae
gi|493334896~Parascardovia_denticolens_IPLA_20019
gi|493331731~Parascardovia_denticolens_DSM_10105_=_JCM_12538
gi|494249175~Scardovia_wiggsiae_F0424
gi|896627280~Streptococcus_anginosus
gi|644024567~Streptococcus_anginosus_subsp__whileyi_MAS624
gi|333769827~Streptococcus_intermedius_SK54
gi|815081984|emb|CRH88698_1|~Chlamydia_trachomatis
gi|960279026~Lactococcus_lactis
gi|502661267~Lactococcus_lactis_subsp__lactis
gi|1020008765~Lactococcus_lactis_subsp__cremoris
gi|497154494~Catellicoccus_marimammalium_M35/04/3
gi|972564240~Streptomyces_alboniger
gi|489232984~Aerococcus_viridans
gi|515408016~Aerococcus_urinaeequi
gi|984719763~Aerococcus_sanguinicola
gi|490774634~Dolosigranulum_pigrum_ATCC_51524
gi|489874550~Alloiococcus_otitis_ATCC_51267
gi|737382437~Abiotrophia_defectiva
gi|554771665~Abiotrophia_defectiva_ATCC_49176
gi|1053994888~Globicatella_sanguinis
gi|493351383~Facklamia_languida_CCUG_37842
gi|654664752~Eremococcus_coleocola
gi|493463273~Eremococcus_coleocola_ACS~139~V~Col8
gi|493752127~Facklamia_ignava_CCUG_37419
gi|751596944~Cohnella_sp__VKM_B~2846
gi|929001170~Bacillus_sp__FJAT~28004
gi|517835308~Cohnella_laeviribosi
gi|652787361~Cohnella_thermotolerans
gi|654658066~Facklamia_sourekii
gi|494109414~Anaerofustis_stercorihominis_DSM_17244
gi|917806124|emb|CEH30726_1|~Aneurinibacillus_migulanus
gi|759012438~Aneurinibacillus_migulanus
gi|951583864~Aneurinibacillus_sp__XH2
gi|506373930~Bacillus_sp__FJAT~27238
gi|640551703~Brevibacillus_borstelensis_cifa_chp40
gi|656062602~Bacillus_sp__WF146
gi|737313273~Brevibacillus_thermoruber
gi|648623227~Brevibacillus_laterosporus
gi|640597114~Bacillus_mannanilyticus
gi|995931914~Tepidibacillus_decaturensis
gi|1033311874~Paenibacillus_sp__1ZS3~15
gi|1050139428~Paenibacillus_pectinilyticus
gi|751580590~Paenibacillus_sp__VKM_B~2647
gi|524549114|emb|CDC79153_1|~Clostridium_sp__CAG465
gi|1024323561~Rathayibacter_tanaceti
gi|1026623880~Rathayibacter_tritici
gi|948210050~Rathayibacter_sp__Leaf296
gi|737868477~Cryobacterium_roopkundense
gi|502629661~Sanguibacter_keddieii_DSM_10542
gi|947467131~Sanguibacter_sp__Leaf3
gi|1054695952~Sanguibacter_suarezii
gi|1053851347~Pseudoclavibacter_alba
gi|1011155767~Demequina_globuliformis
gi|1011004664~Demequina_flava
gi|947454324~Curtobacterium_sp__Leaf183
gi|1032527510|emb|SBN64054_1|~Curtobacterium_sp__9128
gi|947182807~Curtobacterium_sp__Leaf261
gi|930043223~Frigoribacterium_sp__RIT~PI~h
gi|960340495~Actinomyces_sp__SD1
gi|740717013~Rhodoluna_lacicola
gi|926248234~Actinomyces_sp__oral_taxon_414
gi|987452627~Actinomyces_radicidentis
gi|902562523~Actinobaculum_suis
gi|492311591~Candidatus_Arthromitus_sp__SFB~mouse~NL
gi|503861140~Candidatus_Arthromitus_sp__SFB~rat~Yit
gi|1055008232~Candidatus_Arthromitus_sp__SFB~turkey
gi|941139226~Clostridium_sp__ND2
gi|557311481~Youngiibacter_fragilis_232_1
gi|932934404|emb|CUN93425_1|~Faecalibacterium_prausnitzii
gi|972260772~Ruminococcaceae_bacterium_AM2
gi|524701562|emb|CDE08604_1|~Bacillus_sp__CAG988
gi|738664560~Olsenella_uli_MSTE5
gi|301319861~Olsenella_uli_DSM_7084
gi|556508230~Catonella_morbi_ATCC_51271
gi|1055254230~Murdochiella_sp__SIT12
gi|518020114~Levyella_massiliensis
gi|524051587|emb|CCY32815_1|~uncultured_Blautia_sp_
gi|933114568|emb|CUQ12230_1|~Fusicatenibacter_saccharivorans
gi|1052882029|emb|SCH69869_1|~uncultured_Blautia_sp_
gi|524121537|emb|CCY98506_1|~Ruminococcus_sp__CAG17
gi|1052898692|emb|SCI22934_1|~uncultured_Blautia_sp_
gi|551009218~Butyrivibrio_fibrisolvens
gi|551012053~Butyrivibrio_fibrisolvens
gi|653619426~Clostridium_cellobioparum
gi|490766013~Clostridium_termitidis_CT1112
gi|696593258~Intestinimonas_butyriciproducens
gi|959160071~Intestinimonas_butyriciproducens
gi|769161863~Pseudobutyrivibrio_sp__LB2011
gi|697062284~Pseudobutyrivibrio_ruminis
gi|1054794913~Clostridiales_bacterium_KLE1615
gi|1053858124~Clostridium_neopropionicum
gi|1053738040~Clostridium_propionicum_DSM_1682
gi|511540995|emb|CCX39222_1|~Firmicutes_bacterium_CAG102
gi|524650313|emb|CDD61217_1|~Clostridium_sp__CAG505
gi|547848211~Firmicutes_bacterium_CAG466
gi|651393667~Butyrivibrio_sp__NC3005
gi|524146917|emb|CCZ20680_1|~Ruminococcus_sp__CAG724
gi|524045564|emb|CCY25715_1|~Firmicutes_bacterium_CAG114
gi|737664283~Lachnospiraceae_bacterium_ND2006
gi|524008279|emb|CCX90211_1|~Firmicutes_bacterium_CAG110
gi|524442054|emb|CDB86922_1|~Firmicutes_bacterium_CAG170
gi|912592927|emb|CRZ34226_1|~Herbinix_hemicellulosilytica
gi|524655084|emb|CDD64302_1|~Firmicutes_bacterium_CAG882
gi|931449608~Chlamydiae_bacterium_SM23_39
GSChrysophyteH1_ASY1_ANO1_908_1
gi|517522595~Algicola_sagamiensis
gi|494030619~Plesiocystis_pacifica_SIR~1
gi|747218999~Enhygromyxa_salina
gi|496312006~Luminiphilus_syltensis_NOR5~1B
gi|630921109~Anncaliia_algerae_PRA339
gi|737842603~Jeotgalicoccus_sp__13MG44_air
gi|652464419~Jeotgalicoccus_psychrophilus
gi|226377693~Lactobacillus_phage_Lb338~1
gi|918202037~Gorillibacterium_massiliense
gi|960410788~Gorillibacterium_sp__SN4
0.1
Bacteria
Eukaryota
Other
SAG
NA

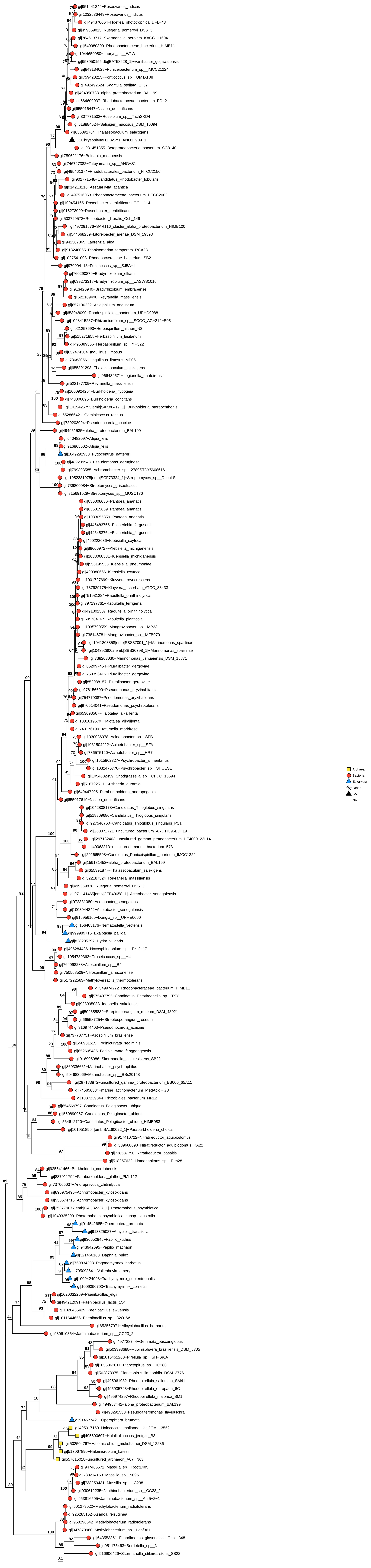

Archaea
Bacteria
Eukaryota
Other
SAG
NA
0.1

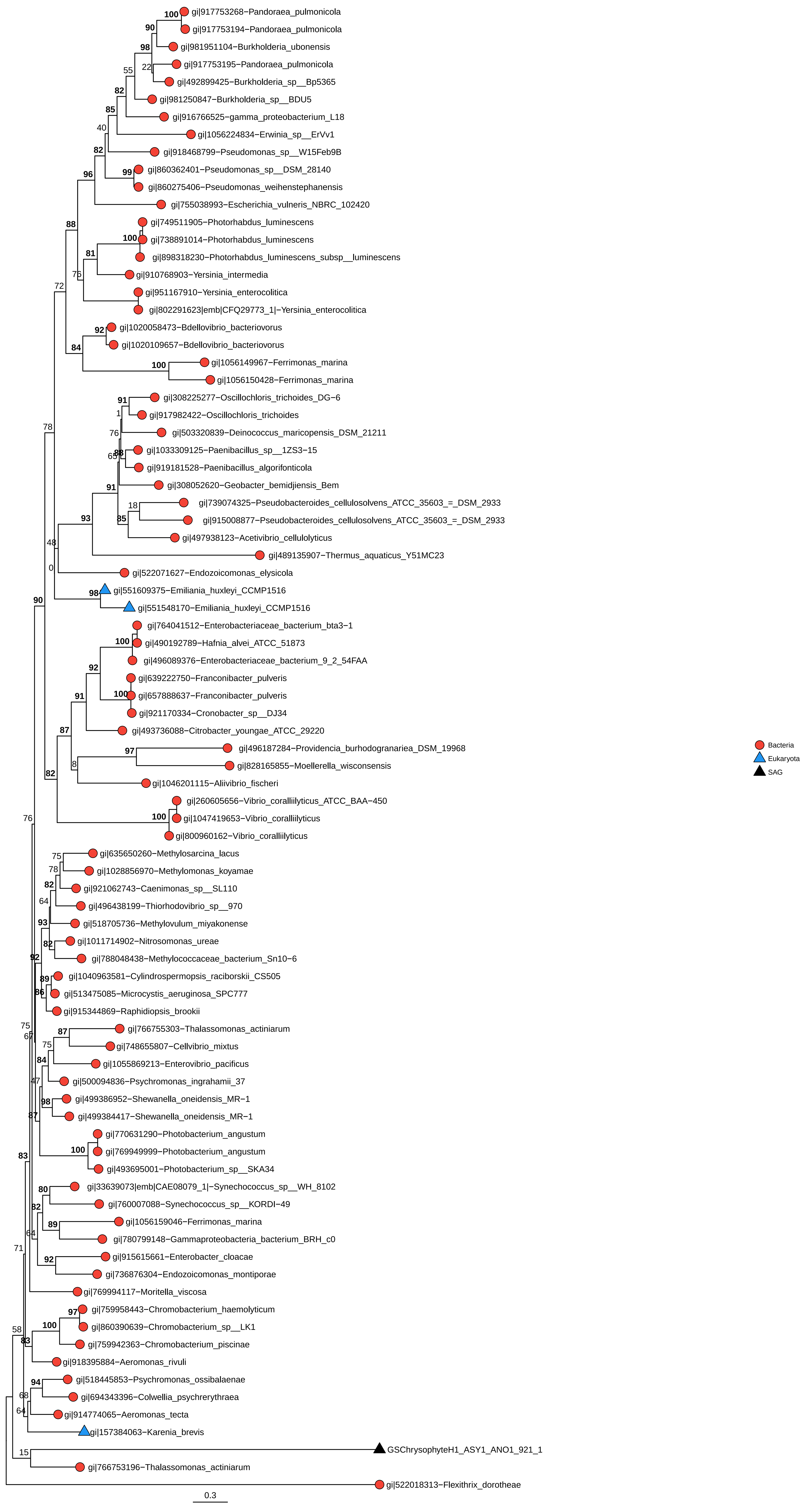
gi|917753268−Pandoraea_pulmonicola
gi|917753194−Pandoraea_pulmonicola
gi|981951104−Burkholderia_ubonensis
gi|917753195−Pandoraea_pulmonicola
gi|492899425−Burkholderia_sp__Bp5365
gi|981250847−Burkholderia_sp__BDU5
gi|916766525−gamma_proteobacterium_L18
gi|1056224834−Erwinia_sp__ErVv1
gi|918468799−Pseudomonas_sp__W15Feb9B
gi|860362401−Pseudomonas_sp__DSM_28140
gi|860275406−Pseudomonas_weihenstephanensis
gi|755038993−Escherichia_vulneris_NBRC_102420
gi|749511905−Photorhabdus_luminescens
gi|738891014−Photorhabdus_luminescens
gi|898318230−Photorhabdus_luminescens_subsp__luminescens
gi|910768903−Yersinia_intermedia
gi|951167910−Yersinia_enterocolitica
gi|802291623|emb|CFQ29773_1|−Yersinia_enterocolitica
gi|1020058473−Bdellovibrio_bacteriovorus
gi|1020109657−Bdellovibrio_bacteriovorus
gi|1056149967−Ferrimonas_marina
gi|1056150428−Ferrimonas_marina
gi|308225277−Oscillochloris_trichoides_DG−6
gi|917982422−Oscillochloris_trichoides
gi|503320839−Deinococcus_maricopensis_DSM_21211
gi|1033309125−Paenibacillus_sp__1ZS3−15
gi|919181528−Paenibacillus_algorifonticola
gi|308052620−Geobacter_bemidjiensis_Bem
gi|739074325−Pseudobacteroides_cellulosolvens_ATCC_35603_=_DSM_2933
gi|915008877−Pseudobacteroides_cellulosolvens_ATCC_35603_=_DSM_2933
gi|497938123−Acetivibrio_cellulolyticus
gi|489135907−Thermus_aquaticus_Y51MC23
gi|522071627−Endozoicomonas_elysicola
gi|551609375−Emiliania_huxleyi_CCMP1516
gi|551548170−Emiliania_huxleyi_CCMP1516
gi|764041512−Enterobacteriaceae_bacterium_bta3−1
gi|490192789−Hafnia_alvei_ATCC_51873
gi|496089376−Enterobacteriaceae_bacterium_9_2_54FAA
gi|639222750−Franconibacter_pulveris
gi|657888637−Franconibacter_pulveris
gi|921170334−Cronobacter_sp__DJ34
gi|493736088−Citrobacter_youngae_ATCC_29220
gi|496187284−Providencia_burhodogranariea_DSM_19968
gi|828165855−Moellerella_wisconsensis
gi|1046201115−Aliivibrio_fischeri
gi|260605656−Vibrio_coralliilyticus_ATCC_BAA−450
gi|1047419653−Vibrio_coralliilyticus
gi|800960162−Vibrio_coralliilyticus
gi|635650260−Methylosarcina_lacus
gi|1028856970−Methylomonas_koyamae
gi|921062743−Caenimonas_sp__SL110
gi|496438199−Thiorhodovibrio_sp__970
gi|518705736−Methylovulum_miyakonense
gi|1011714902−Nitrosomonas_ureae
gi|788048438−Methylococcaceae_bacterium_Sn10−6
gi|1040963581−Cylindrospermopsis_raciborskii_CS505
gi|513475085−Microcystis_aeruginosa_SPC777
gi|915344869−Raphidiopsis_brookii
gi|766755303−Thalassomonas_actiniarum
gi|748655807−Cellvibrio_mixtus
gi|1055869213−Enterovibrio_pacificus
gi|500094836−Psychromonas_ingrahamii_37
gi|499386952−Shewanella_oneidensis_MR−1
gi|499384417−Shewanella_oneidensis_MR−1
gi|770631290−Photobacterium_angustum
gi|769949999−Photobacterium_angustum
gi|493695001−Photobacterium_sp__SKA34
gi|33639073|emb|CAE08079_1|−Synechococcus_sp__WH_8102
gi|760007088−Synechococcus_sp__KORDI−49
gi|1056159046−Ferrimonas_marina
gi|780799148−Gammaproteobacteria_bacterium_BRH_c0
gi|915615661−Enterobacter_cloacae
gi|736876304−Endozoicomonas_montiporae
gi|769994117−Moritella_viscosa
gi|759958443−Chromobacterium_haemolyticum
gi|860390639−Chromobacterium_sp__LK1
gi|759942363−Chromobacterium_piscinae
gi|918395884−Aeromonas_rivuli
gi|518445853−Psychromonas_ossibalaenae
gi|694343396−Colwellia_psychrerythraea
gi|914774065−Aeromonas_tecta
gi|157384063−Karenia_brevis
GSChrysophyteH1_ASY1_ANO1_921_1
gi|766753196−Thalassomonas_actiniarum
gi|522018313−Flexithrix_dorotheae
0.3
Bacteria
Eukaryota
SAG

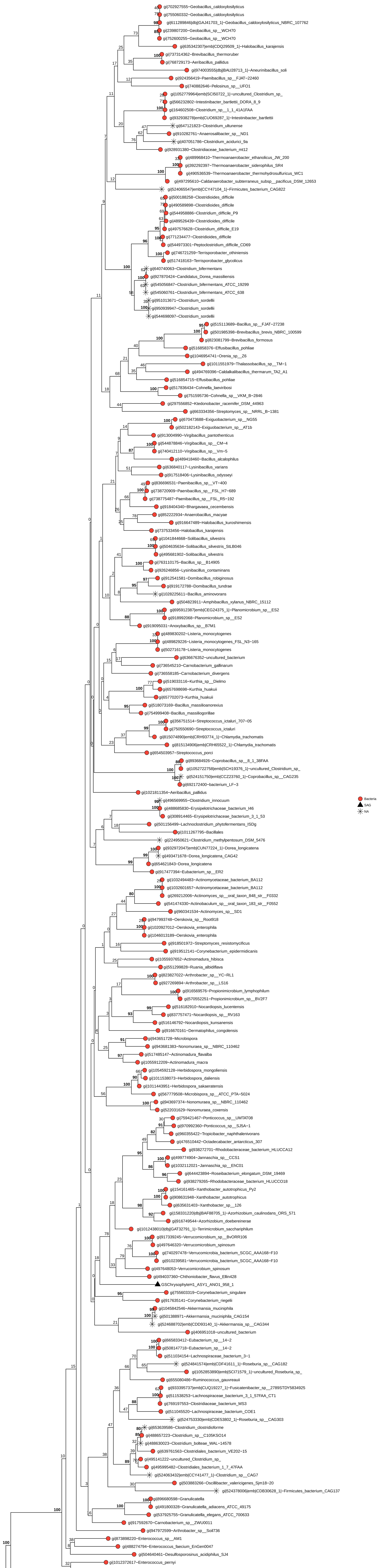

Bacteria
SAG
NA
0.3

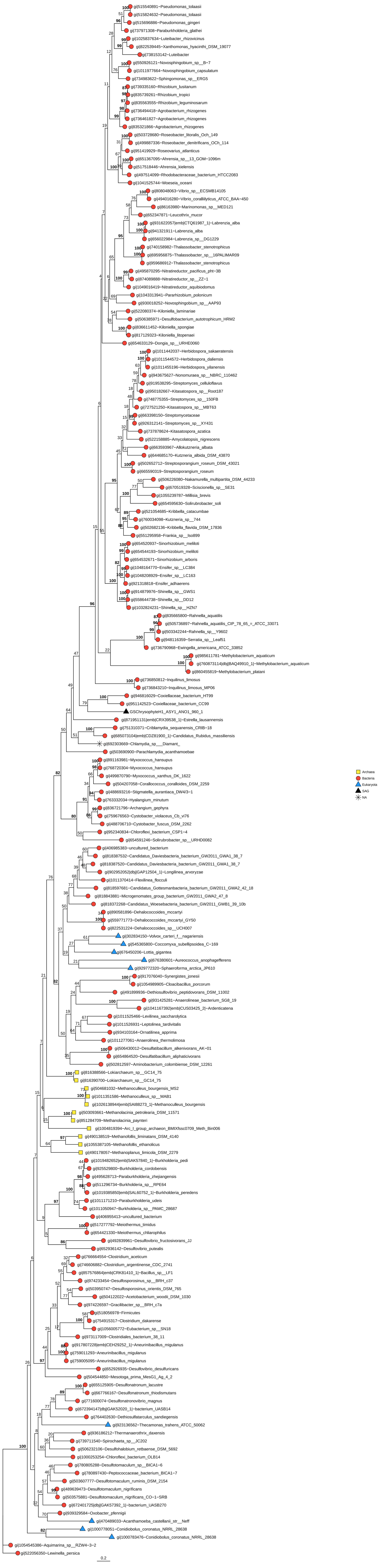
gi|515540891~Pseudomonas_tolaasii
gi|515824632~Pseudomonas_tolaasii
gi|515696886~Pseudomonas_gingeri
gi|737971308~Paraburkholderia_glathei
gi|1025837634~Luteibacter_rhizovicinus
gi|822539445~Xanthomonas_hyacinthi_DSM_19077
gi|738153142~Luteibacter
gi|550926121~Novosphingobium_sp__B-7
gi|1011977664~Novosphingobium_capsulatum
gi|734983622~Sphingomonas_sp__ERG5
gi|739335160~Rhizobium_lusitanum
gi|835739261~Rhizobium_tropici
gi|835563555~Rhizobium_leguminosarum
gi|736494418~Agrobacterium_rhizogenes
gi|736461827~Agrobacterium_rhizogenes
gi|835321866~Agrobacterium_rhizogenes
gi|503728680~Roseobacter_litoralis_Och_149
gi|499887336~Roseobacter_denitrificans_OCh_114
gi|951419929~Roseovarius_atlanticus
gi|651367095~Ahrensia_sp__13_GOM-1096m
gi|517518446~Ahrensia_kielensis
gi|497514099~Rhodobacteraceae_bacterium_HTCC2083
gi|1041525744~Woeseia_oceani
gi|808048063~Vibrio_sp__ECSMB14105
gi|494016280~Vibrio_coralliilyticus_ATCC_BAA-450
gi|86163980~Marinomonas_sp__MED121
gi|652347871~Leucothrix_mucor
gi|931622057|emb|CTQ61987_1|~Labrenzia_alba
gi|941321911~Labrenzia_alba
gi|656022984~Labrenzia_sp_DG1229
gi|740158982~Thalassobacter_stenotrophicus
gi|695956875~Thalassobacter_sp__16PALIMAR09
gi|959686912~Thalassobacter_stenotrophicus
gi|495870295~Nitratireductor_pacificus_pht-3B
gi|874089888~Nitratireductor_sp__ZZ-1
gi|1049016419~Nitratireductor_aquibiodomus
gi|1043313941~Pararhizobium_polonicum
gi|930018252~Novosphingobium_sp__AAP93
gi|522080374~Kiloniella_laminariae
gi|506385971~Desulfobacterium_autotrophicum_HRM2
gi|836611452~Kiloniella_spongiae
gi|817129323~Kiloniella_litopenaei
gi|654633129~Dongia_sp__URHE0060
gi|1011442037~Herbidospora_sakaeratensis
gi|1011544572~Herbidospora_daliensis
gi|1011455196~Herbidospora_yilanensis
gi|943675627~Nonomuraea_sp__NBRC_110462
gi|919538295~Streptomyces_cellulolflavus
gi|950182667~Kitasatospora_sp__Root187
gi|748775355~Streptomyces_sp__150FB
gi|727521250~Kitasatospora_sp__MBT63
gi|663398150~Streptomycetaceae
gi|926312141~Streptomyces_sp__XY431
gi|737878624~Kitasatospora_azatica
gi|522158885~Amycolatopsis_nigrescens
gi|663593967~Allokutzneria_albata
gi|644685170~Kutzneria_albida_DSM_43870
gi|502652712~Streptosporangium_roseum_DSM_43021
gi|665590319~Streptosporangium_roseum
gi|506226080~Nakamurella_multipartita_DSM_44233
gi|670519328~Sciscionella_sp__SE31
gi|1055239787~Millisia_brevis
gi|654595630~Solirubrobacter_soli
gi|521054685~Kribbella_catacumbae
gi|760034098~Kutzneria_sp__744
gi|502682136~Kribbella_flavida_DSM_17836
gi|551295958~Frankia_sp__Iso899
gi|654520937~Sinorhizobium_meliloti
gi|654544193~Sinorhizobium_meliloti
gi|654532671~Sinorhizobium_arboris
gi|1048164770~Ensifer_sp__LC384
gi|1048208929~Ensifer_sp__LC163
gi|921318818~Ensifer_adhaerens
gi|914879976~Shinella_sp__GWS1
gi|558644738~Shinella_sp__DD12
gi|1032824231~Shinella_sp__HZN7
gi|835665800~Rahnella_aquatilis
gi|505736897~Rahnella_aquatilis_CIP_78_65_=_ATCC_33071
gi|503342244~Rahnella_sp__Y9602
gi|948116359~Serratia_sp__Leaf51
gi|736790968~Ewingella_americana_ATCC_33852
gi|985611781~Methylobacterium_aquaticum
gi|760873114|dbj|BAQ49910_1|~Methylobacterium_aquaticum
gi|860455819~Methylobacterium_platani
gi|736850812~Inquilinus_limosus
gi|736843210~Inquilinus_limosus_MP06
gi|946816029~Coxiellaceae_bacterium_HT99
gi|951142523~Coxiellaceae_bacterium_CC99
GSChrysophyteH1_ASY1_ANO1_960_1
gi|871951131|emb|CRX39538_1|~Estrella_lausannensis
gi|751310371~Criblamydia_sequanensis_CRIB-18
gi|685073104|emb|CDZ81900_1|~Candidatus_Rubidus_massiliensis
gi|692303669~Chlamydia_sp__Diamant_
gi|503690900~Parachlamydia_acanthamoebae
gi|891163981~Myxococcus_hansupus
gi|768720304~Myxococcus_hansupus
gi|499870790~Myxococcus_xanthus_DK_1622
gi|504207058~Corallococcus_coralloides_DSM_2259
gi|488693216~Stigmatella_aurantiaca_DW4/3-1
gi|763332034~Hyalangium_minutum
gi|836721796~Archangium_gephyra
gi|759676563~Cystobacter_violaceus_Cb_vi76
gi|488706710~Cystobacter_fuscus_DSM_2262
gi|952340834~Chloroflexi_bacterium_CSP1-4
gi|654591246~Solirubrobacter_sp__URHD0082
gi|406985383~uncultured_bacterium
gi|818387532~Candidatus_Daviesbacteria_bacterium_GW2011_GWA1_38_7
gi|818387520~Candidatus_Daviesbacteria_bacterium_GW2011_GWA1_38_7
gi|902952052|dbj|GAP12504_1|~Longilinea_arvoryzae
gi|1011370414~Flexilinea_flocculi
gi|818597681~Candidatus_Gottesmanbacteria_bacterium_GW2011_GWA2_42_18
gi|818843881~Microgenomates_group_bacterium_GW2011_GWA2_47_8
gi|818372268~Candidatus_Woesebacteria_bacterium_GW2011_GWB1_39_10b
gi|890581896~Dehalococcoides_mccartyi
gi|559771773~Dehalococcoides_mccartyi_GY50
gi|822531224~Dehalococcoides_sp__UCH007
gi|302834150~Volvox_carteri_f__nagariensis
gi|545365800~Coccomyxa_subellipsoidea_C-169
gi|676450206~Lotia_gigantea
gi|676380601~Aureococcus_anophagefferens
gi|929772320~Sphaeroforma_arctica_JP610
gi|917076040~Synergistes_jonesii
gi|1054989905~Cloacibacillus_porcorum
gi|491899936~Dethiosulfovibrio_peptidovorans_DSM_11002
gi|931425281~Anaerolineae_bacterium_SG8_19
gi|1041167392|emb|CUS03425_2|~Ardenticatena
gi|1011525466~Levilinea_saccharolytica
gi|1011526931~Leptolinea_tardivitalis
gi|934103164~Ornatilinea_apprima
gi|1011277061~Anaerolinea_thermolimosa
gi|506430012~Desulfatibacillum_alkenivorans_AK-01
gi|654864520~Desulfatibacillum_aliphaticivorans
gi|502812597~Aminobacterium_colombiense_DSM_12261
gi|816388566~Lokiarchaeum_sp__GC14_75
gi|816390700~Lokiarchaeum_sp__GC14_75
gi|504681032~Methanoculleus_bourgensis_MS2
gi|1011351586~Methanoculleus_sp__MAB1
gi|1026138944|emb|SAI88273_1|~Methanoculleus_bourgensis
gi|503093661~Methanolacinia_petrolearia_DSM_11571
gi|851284709~Methanolacinia_paynteri
gi|1004819394~Arc_I_group_archaeon_BMIXfssc0709_Meth_Bin006
gi|490138519~Methanofollis_liminatans_DSM_4140
gi|1055387105~Methanofollis_ethanolicus
gi|490178057~Methanoplanus_limicola_DSM_2279
gi|1019482652|emb|SAK57840_1|~Burkholderia_pedi
gi|925529800~Burkholderia_cordobensis
gi|495628713~Paraburkholderia_zhejiangensis
gi|511296734~Burkholderia_sp__RPE64
gi|1019385850|emb|SAL60752_1|~Burkholderia_peredens
gi|1011171210~Paraburkholderia_udeis
gi|1011050947~Burkholderia_sp__PAMC_28687
gi|406955413~uncultured_bacterium
gi|517277792~Meiothermus_timidus
gi|654421330~Meiothermus_chliarophilus
gi|492839961~Desulfovibrio_fructosivorans_JJ
gi|652936142~Desulfovibrio_putealis
gi|766664554~Clostridium_aceticum
gi|746606882~Clostridium_argentinense_CDC_2741
gi|857576864|emb|CRK81410_1|~Bacillus_sp__LF1
gi|974233454~Desulfosporosinus_sp__BRH_c37
gi|503950747~Desulfosporosinus_orientis_DSM_765
gi|504122022~Acetobacterium_woodii_DSM_1030
gi|974226597~Gracilibacter_sp__BRH_c7a
gi|518056978~Firmicutes
gi|754915317~Clostridium_dakarense
gi|1056005772~Eubacterium_sp__SN18
gi|973117009~Clostridiales_bacterium_38_11
gi|917807228|emb|CEH29252_1|~Aneurinibacillus_migulanus
gi|759011293~Aneurinibacillus_migulanus
gi|759005095~Aneurinibacillus_migulanus
gi|652926935~Desulfovibrio_desulfuricans
gi|504544850~Mesotoga_prima_MesG1_Ag_4_2
gi|655125905~Desulfonatronum_lacustre
gi|667766167~Desulfonatronum_thiodismutans
gi|771600074~Desulfonatronovibrio_magnus
gi|672394147|dbj|GAK52020_1|~bacterium_UASB14
gi|764402630~Dethiosulfatarculus_sandiegensis
gi|923136562~Thecamonas_trahens_ATCC_50062
gi|936186212~Thermanaerothrix_daxensis
gi|739711540~Spirochaeta_sp__JC202
gi|506232106~Desulfohalobium_retbaense_DSM_5692
gi|1000253254~Chloroflexi_bacterium_OLB14
gi|780805288~Desulfotomaculum_sp__BICA1-6
gi|780897430~Peptococcaceae_bacterium_BICA1-7
gi|503607777~Desulfotomaculum_ruminis_DSM_2154
gi|489639473~Desulfotomaculum_nigrificans
gi|503575881~Desulfotomaculum_nigrificans_CO-1-SRB
gi|672401725|dbj|GAK57392_1|~bacterium_UASB270
gi|939329584~Oxobacter_pfennigii
gi|470489033~Acanthamoeba_castellanii_str__Neff
gi|1000778051~Conidiobolus_coronatus_NRRL_28638
gi|1000783476~Conidiobolus_coronatus_NRRL_28638
gi|1054545386~Aquimarina_sp__RZW4-3-2
gi|522056350~Lewinella_persica
0.2
Archaea
Bacteria
Eukaryota
SAG
NA

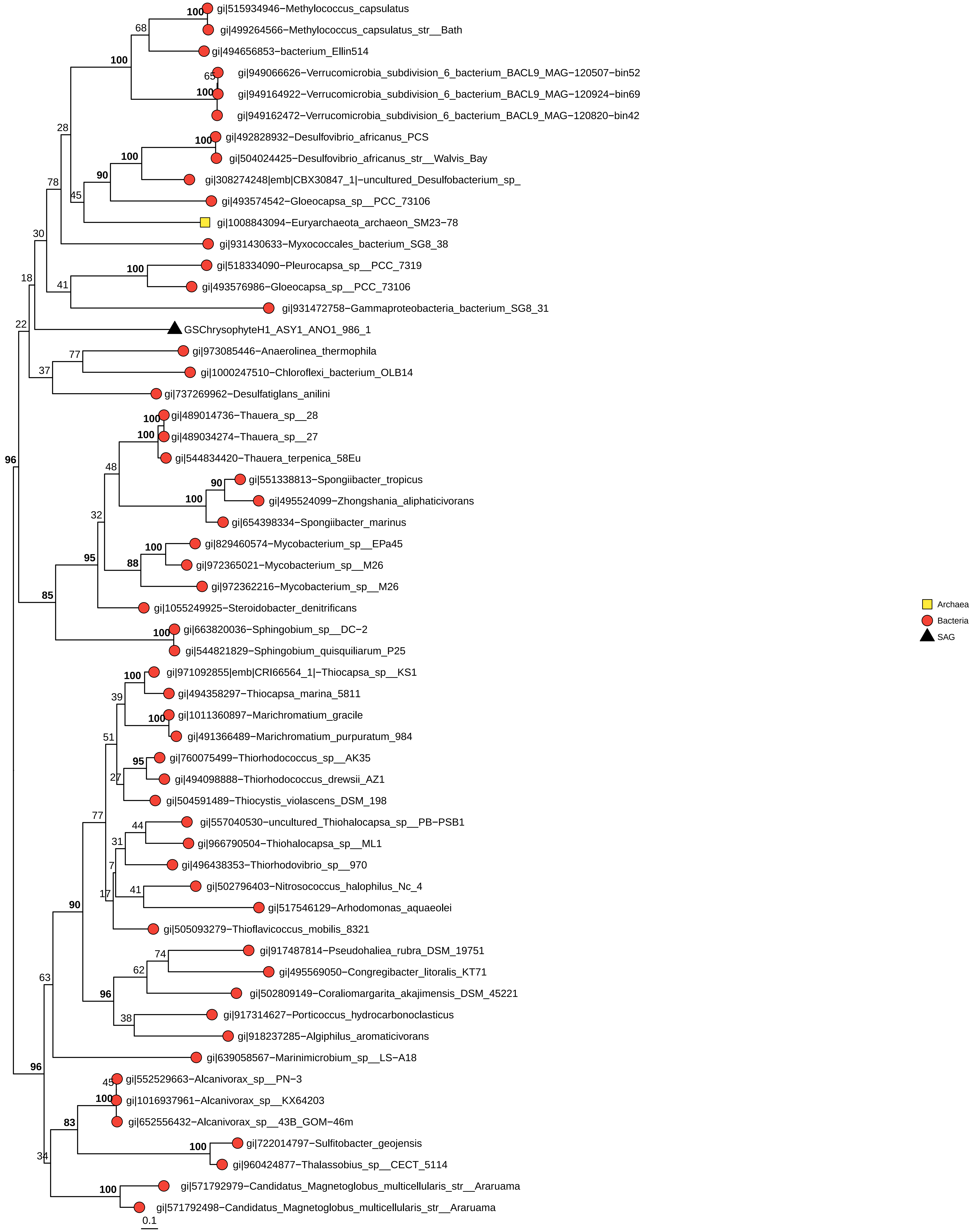
gi|515934946−Methylococcus_capsulatus
gi|499264566−Methylococcus_capsulatus_str__Bath
gi|494656853−bacterium_Ellin514
gi|949066626−Verrucomicrobia_subdivision_6_bacterium_BACL9_MAG−120507−bin52
gi|949164922−Verrucomicrobia_subdivision_6_bacterium_BACL9_MAG−120924−bin69
gi|949162472−Verrucomicrobia_subdivision_6_bacterium_BACL9_MAG−120820−bin42
gi|492828932−Desulfovibrio_africanus_PCS
gi|504024425−Desulfovibrio_africanus_str__Walvis_Bay
gi|308274248|emb|CBX30847_1|−uncultured_Desulfobacterium_sp_
gi|493574542−Gloeocapsa_sp__PCC_73106
gi|1008843094−Euryarchaeota_archaeon_SM23−78
gi|931430633−Myxococcales_bacterium_SG8_38
gi|518334090−Pleurocapsa_sp__PCC_7319
gi|493576986−Gloeocapsa_sp__PCC_73106
gi|931472758−Gammaproteobacteria_bacterium_SG8_31
GSChrysophyteH1_ASY1_ANO1_986_1
gi|973085446−Anaerolinea_thermophila
gi|1000247510−Chloroflexi_bacterium_OLB14
gi|737269962−Desulfatiglans_anilini
gi|489014736−Thauera_sp__28
gi|489034274−Thauera_sp__27
gi|544834420−Thauera_terpenica_58Eu
gi|551338813−Spongiibacter_tropicus
gi|495524099−Zhongshania_aliphaticivorans
gi|654398334−Spongiibacter_marinus
gi|829460574−Mycobacterium_sp__EPa45
gi|972365021−Mycobacterium_sp__M26
gi|972362216−Mycobacterium_sp__M26
gi|1055249925−Steroidobacter_denitrificans
gi|663820036−Sphingobium_sp__DC−2
gi|544821829−Sphingobium_quisquiliarum_P25
gi|971092855|emb|CRI66564_1|−Thiocapsa_sp__KS1
gi|494358297−Thiocapsa_marina_5811
gi|1011360897−Marichromatium_gracile
gi|491366489−Marichromatium_purpuratum_984
gi|760075499−Thiorhodococcus_sp__AK35
gi|494098888−Thiorhodococcus_drewsii_AZ1
gi|504591489−Thiocystis_violascens_DSM_198
gi|557040530−uncultured_Thiohalocapsa_sp__PB−PSB1
gi|966790504−Thiohalocapsa_sp__ML1
gi|496438353−Thiorhodovibrio_sp__970
gi|502796403−Nitrosococcus_halophilus_Nc_4
gi|517546129−Arhodomonas_aquaeolei
gi|505093279−Thioflavicoccus_mobilis_8321
gi|917487814−Pseudohaliea_rubra_DSM_19751
gi|495569050−Congregibacter_litoralis_KT71
gi|502809149−Coraliomargarita_akajimensis_DSM_45221
gi|917314627−Porticoccus_hydrocarbonoclasticus
gi|918237285−Algiphilus_aromaticivorans
gi|639058567−Marinimicrobium_sp__LS−A18
gi|552529663−Alcanivorax_sp__PN−3
gi|1016937961−Alcanivorax_sp__KX64203
gi|652556432−Alcanivorax_sp__43B_GOM−46m
gi|722014797−Sulfitobacter_geojensis
gi|960424877−Thalassobius_sp__CECT_5114
gi|571792979−Candidatus_Magnetoglobus_multicellularis_str__Araruama
gi|571792498−Candidatus_Magnetoglobus_multicellularis_str__Araruama
0.1
Archaea
Bacteria
SAG

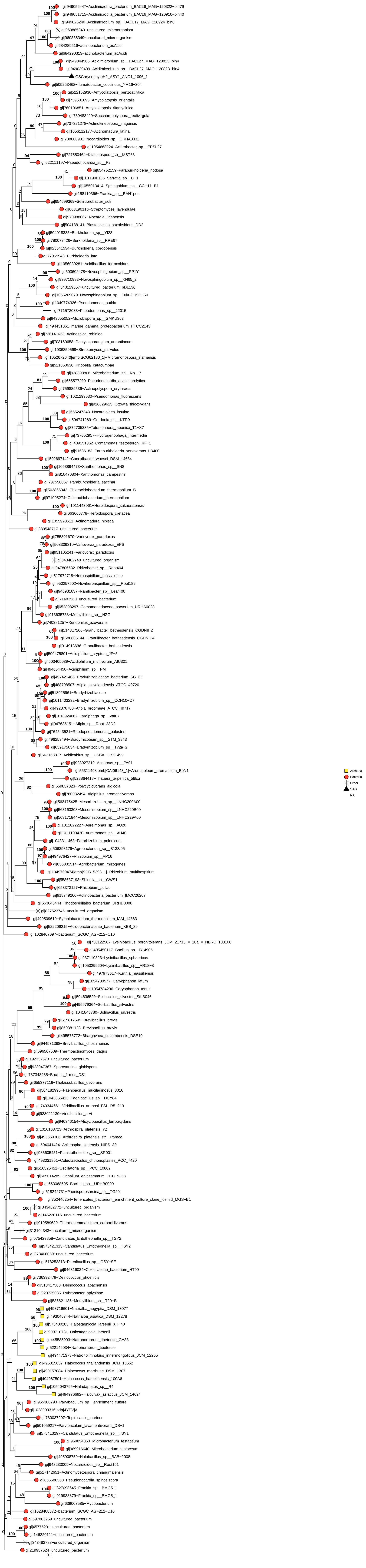
Archaea
Bacteria
Other
SAG
NA
0.1

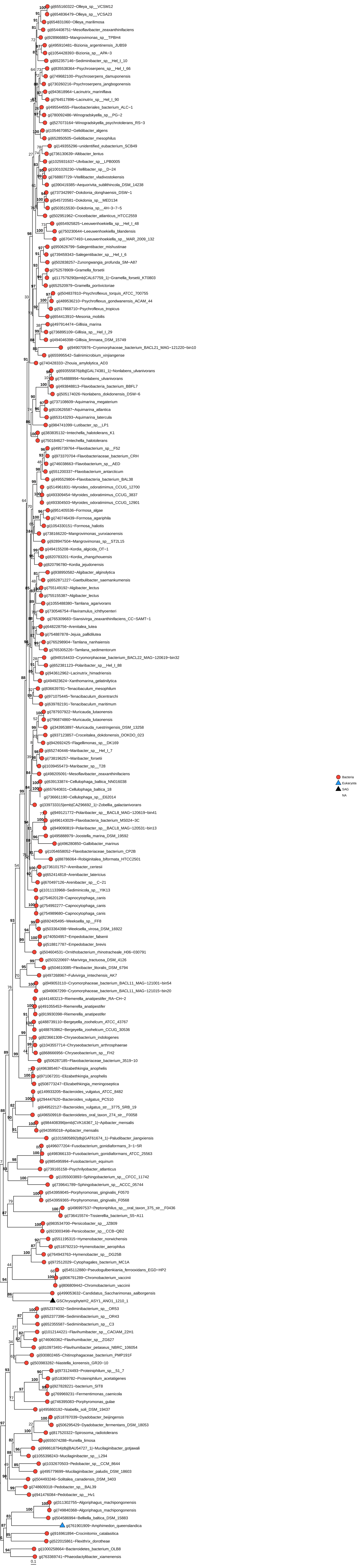
Bacteria
Eukaryota
SAG
NA
0.1

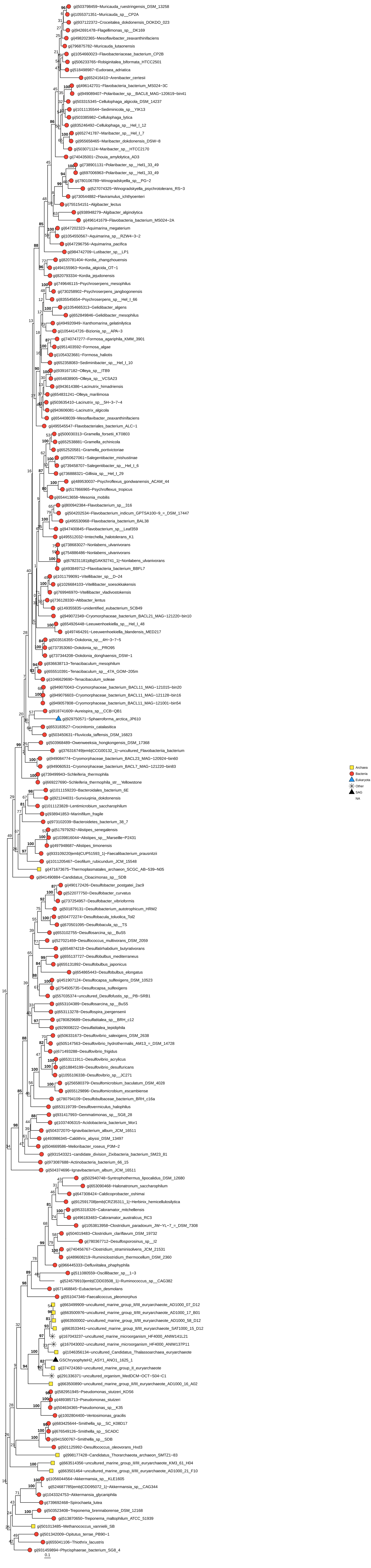

gi|503798459~Muricauda_ruestringensis_DSM_13258
gi|1055371351~Muricauda_sp__CIP2A
gi|937122372~Croceitalea_dokdonensis_DOKDO_023
gi|942691478~Flagellimonas_sp__DK169
gi|498202365~Mesoflavibacter_zeaxanthinifaciens
gi|796875782~Muricauda_lutaonensis
gi|1054660023~Flavobacteriaceae_bacterium_CP2B
gi|506233765~Robiginitalea_biformata_HTCC2501
gi|518498987~Eudoraea_adriatica
gi|652416410~Arenibacter_certesii
gi|496142701~Flavobacteria_bacterium_MS024~3C
gi|949089407~Polaribacter_sp__BACL8_MAG~120619~bin41
gi|503315345~Cellulophaga_algicola_DSM_14237
gi|1011155544~Sediminicola_sp__YIK13
gi|503385982~Cellulophaga_lytica
gi|835246492~Cellulophaga_sp__Hel_I_12
gi|652741787~Maribacter_sp__Hel_I_7
gi|955658465~Maribacter_dokdonensis_DSW~8
gi|503071124~Maribacter_sp__HTCC2170
gi|740435001~Zhouia_amylolytica_AD3
gi|738901131~Polaribacter_sp__Hel1_33_49
gi|697006963~Polaribacter_sp__Hel1_33_49
gi|780106789~Winogradskyella_sp__PG~2
gi|527074325~Winogradskyella_psychrotolerans_RS~3
gi|730544882~Flavimonas_ichthyoenteri
gi|755154151~Algibacter_lectus
gi|938948279~Algibacter_alginolytica
gi|496141679~Flavobacteria_bacterium_MS024~2A
gi|647202323~Aquimarina_megaterium
gi|1054550567~Aquimarina_sp__RZW4~3~2
gi|647296756~Aquimarina_pacifica
gi|984742709~Lutibacter_sp__LP1
gi|820781404~Kordia_zhangzhouensis
gi|494155963~Kordia_algicida_OT~1
gi|820793334~Kordia_jejudonensis
gi|749646115~Psychroserpens_mesophilus
gi|730258902~Psychroserpens_jangbogonensis
gi|835545654~Psychroserpens_sp__Hel_I_66
gi|1054665313~Gelidibacter_algens
gi|652849846~Gelidibacter_mesophilus
gi|494920949~Xanthomarina_gelatinilytica
gi|1054414726~Bizionia_sp__APA~3
gi|740747277~Formosa_agariphila_KMM_3901
gi|951403592~Formosa_algae
gi|1054323681~Formosa_haliotis
gi|652358083~Sediminibacter_sp__Hel_I_10
gi|939167182~Olleya_sp__ITB9
gi|654838905~Olleya_sp__VCSA23
gi|943614386~Lacinutrix_himadriensis
gi|654831241~Olleya_marilimosa
gi|503635410~Lacinutrix_sp__5H~3~7~4
gi|643606081~Lacinutrix_algicola
gi|655408039~Mesoflavibacter_zeaxanthinifaciens
gi|495545547~Flavobacteriales_bacterium_ALC~1
gi|500030313~Gramella_forsetii_KT0803
gi|652538881~Gramella_echinicola
gi|652520581~Gramella_portivictoriae
gi|950627061~Salegentibacter_mishustinae
gi|739458707~Salegentibacter_sp__Hel_I_6
gi|736888321~Gillisia_sp__Hel_I_29
gi|489530037~Psychroflexus_gondwanensis_ACAM_44
gi|517866965~Psychroflexus_tropicus
gi|654413658~Mesonia_mobilis
gi|800942384~Flavobacterium_sp__316
gi|504202534~Flavobacterium_indicum_GPTSA100~9_=_DSM_17447
gi|495530968~Flavobacteria_bacterium_BAL38
gi|947400845~Flavobacterium_sp__Leaf359
gi|495512032~Imtechella_halotolerans_K1
gi|738683027~Nonlabens_ulvanivorans
gi|754886486~Nonlabens_ulvanivorans
gi|678231181|dbj|GAK92741_1|~Nonlabens_ulvanivorans
gi|493849712~Flavobacteria_bacterium_BBFL7
gi|1011799091~Vitellibacter_sp__D~24
gi|1026684103~Vitellibacter_soesokkakensis
gi|769946970~Vitellibacter_vladivostokensis
gi|736128330~Altibacter_lentus
gi|149355835~unidentified_eubacterium_SCB49
gi|949072349~Cryomorphaceae_bacterium_BACL21_MAG~121220~bin10
gi|655492648~Leeuwenhoekiella_sp__Hel_I_48
gi|497464291~Leeuwenhoekiella_blandensis_MED217
gi|503516355~Dokdonia_sp__4H~3~7~5
gi|737353060~Dokdonia_sp__PRO95
gi|737344208~Dokdonia_donghaensis_DSW~1
gi|836638713~Tenacibaculum_mesophilum
gi|655510391~Tenacibaculum_sp__47A_GOM~205m
gi|1046629690~Tenacibaculum_soleae
gi|949070043~Cryomorphaceae_bacterium_BACL11_MAG~121015~bin20
gi|949076603~Cryomorphaceae_bacterium_BACL11_MAG~121128~bin16
gi|949057808~Cryomorphaceae_bacterium_BACL11_MAG~121001~bin54
gi|918741609~Aureispira_sp__CCB~QB1
gi|929750571~Sphaeroforma_arctica_JP610
gi|653183527~Crocinitomix_catalasitica
gi|503450631~Fluviicola_taffensis_DSM_16823
gi|503968489~Owenweeksia_hongkongensis_DSM_17368
gi|376316749|emb|CCG00132_1|~uncultured_Flavobacteria_bacterium
gi|949084774~Cryomorphaceae_bacterium_BACL23_MAG~120924~bin60
gi|949060531~Cryomorphaceae_bacterium_BACL7_MAG~121220~bin83
gi|739499943~Schleiferia_thermophila
gi|669227690~Schleiferia_thermophila_str__Yellowstone
gi|1011159220~Bacteroidales_bacterium_6E
gi|921244031~Sunxiuqinia_dokdonensis
gi|1011123828~Lentimicrobium_saccharophilum
gi|938941853~Marinifilum_fragile
gi|973102039~Bacteroidetes_bacterium_38_7
gi|517979292~Alistipes_senegalensis
gi|1039816044~Alistipes_sp__Marseille~P2431
gi|497948687~Alistipes_timonensis
gi|933109220|emb|CUP51593_1|~Faecalibacterium_prausnitzii
gi|1011205467~Geofilum_rubicundum_JCM_15548
gi|471673675~Thermoplasmatales_archaeon_SCGC_AB~539~N05
gi|941490884~Candidatus_Cloacimonas_sp__SDB
gi|490172426~Desulfobacter_postgatei_2ac9
gi|522077750~Desulfobacter_curvatus
gi|737254957~Desulfobacter_vibrioformis
gi|501879131~Desulfobacterium_autotrophicum_HRM2
gi|504772274~Desulfobacula_toluolica_Tol2
gi|670501095~Desulfobacula_sp__TS
gi|653102755~Desulfosarcina_sp__BuS5
gi|527021459~Desulfococcus_multivorans_DSM_2059
gi|654874218~Desulfatirhabdium_butyrativorans
gi|655137727~Desulfobulbus_mediterraneus
gi|655131892~Desulfobulbus_japonicus
gi|654865443~Desulfobulbus_elongatus
gi|451907124~Desulfocapsa_sulfexigens_DSM_10523
gi|754505735~Desulfocapsa_sulfexigens
gi|557035374~uncultured_Desulfofustis_sp__PB~SRB1
gi|653104389~Desulfosarcina_sp__BuS5
gi|653113278~Desulfospira_joergensenii
gi|780829689~Desulfatitalea_sp__BRH_c12
gi|929008222~Desulfatitalea_tepidiphila
gi|506331673~Desulfovibrio_salexigens_DSM_2638
gi|505147563~Desulfovibrio_hydrothermalis_AM13_=_DSM_14728
gi|671493288~Desulfovibrio_frigidus
gi|653111911~Desulfovibrio_acrylicus
gi|518845199~Desulfovibrio_desulfuricans
gi|1055106338~Desulfovibrio_sp__JC271
gi|256580379~Desulfomicrobium_baculatum_DSM_4028
gi|655129896~Desulfomicrobium_escambiense
gi|780794109~Desulfobulbaceae_bacterium_BRH_c16a
gi|653119739~Desulfovermiculus_halophilus
gi|931417993~Gemmatimonas_sp__SG8_28
gi|1037406315~Acidobacteria_bacterium_Mor1
gi|504372070~Ignavibacterium_album_JCM_16511
gi|493986345~Caldithrix_abyssi_DSM_13497
gi|504669586~Melioribacter_roseus_P3M~2
gi|931543321~candidate_division_Zixibacteria_bacterium_SM23_81
gi|973087688~Actinobacteria_bacterium_66_15
gi|504374696~Ignavibacterium_album_JCM_16511
gi|502940748~Syntrophothermus_lipocalidus_DSM_12680
gi|653090468~Halonatronum_saccharophilum
gi|647308424~Caldicoprobacter_oshimai
gi|912591708|emb|CRZ35311_1|~Herbinix_hemicellulosilytica
gi|953318326~Caloramator_mitchellensis
gi|496183483~Caloramator_australicus_RC3
gi|1053813958~Clostridium_paradoxum_JW~YL~7_=_DSM_7308
gi|504019483~Clostridium_clariflavum_DSM_19732
gi|780367712~Desulfosporosinus_sp__I2
gi|740456767~Clostridium_straminisolvens_JCM_21531
gi|489608219~Ruminiclostridium_thermocellum_DSM_2360
gi|966445333~Defluviitalea_phaphyphila
gi|511080559~Oscillibacter_sp__1~3
gi|524579910|emb|CDD03508_1|~Ruminococcus_sp__CAG382
gi|671468845~Eubacterium_desmolans
gi|551047346~Faecalicoccus_pleomorphus
gi|663499909~uncultured_marine_group_II/III_euryarchaeote_AD1000_07_D12
gi|663500976~uncultured_marine_group_II/III_euryarchaeote_AD1000_17_B01
gi|663500002~uncultured_marine_group_II/III_euryarchaeote_AD1000_58_D12
gi|663533441~uncultured_marine_group_II/III_euryarchaeote_SAT1000_15_D12
gi|167043237~uncultured_marine_microorganism_HF4000_ANIW141L21
gi|167043002~uncultured_marine_microorganism_HF4000_ANIW137P11
gi|1046356134~uncultured_Candidatus_Thalassoarchaea_euryarchaeote
GSChrysophyteH2_ASY1_ANO1_1625_1
gi|374724360~uncultured_marine_group_II_euryarchaeote
gi|291336371~uncultured_organism_MedDCM~OCT~S04~C1
gi|663500890~uncultured_marine_group_II/III_euryarchaeote_AD1000_16_A02
gi|582951945~Pseudomonas_stutzeri_KOS6
gi|489385713~Pseudomonas_stutzeri
gi|504634365~Pseudomonas_sp__K35
gi|1002804400~Ventosimonas_gracilis
gi|683425644~Smithella_sp__SC_K08D17
gi|676549126~Smithella_sp__SCADC
gi|941500767~Smithella_sp__SDB
gi|501125992~Desulfococcus_oleovorans_Hxd3
gi|998177428~Candidatus_Thorarchaeota_archaeon_SMTZ1~83
gi|663514356~uncultured_marine_group_II/III_euryarchaeote_KM3_61_H04
gi|663501464~uncultured_marine_group_II/III_euryarchaeote_AD1000_21_F10
gi|1056044564~Akkermansia_sp__KLE1605
gi|524687785|emb|CDD95072_1|~Akkermansia_sp__CAG344
gi|1043324753~Akkermansia_glycaniphila
gi|739692468~Spirochaeta_lutea
gi|503523408~Treponema_brennaborense_DSM_12168
gi|513870650~Treponema_maltophilum_ATCC_51939
gi|501013485~Methanococcus_vannielii_SB
gi|501342009~Opitutus_terrae_PB90~1
gi|655041106~Thiothrix_lacustris
gi|931459894~Phycisphaerae_bacterium_SG8_4
0.1
Archaea
Bacteria
Eukaryota
Other
SAG
NA

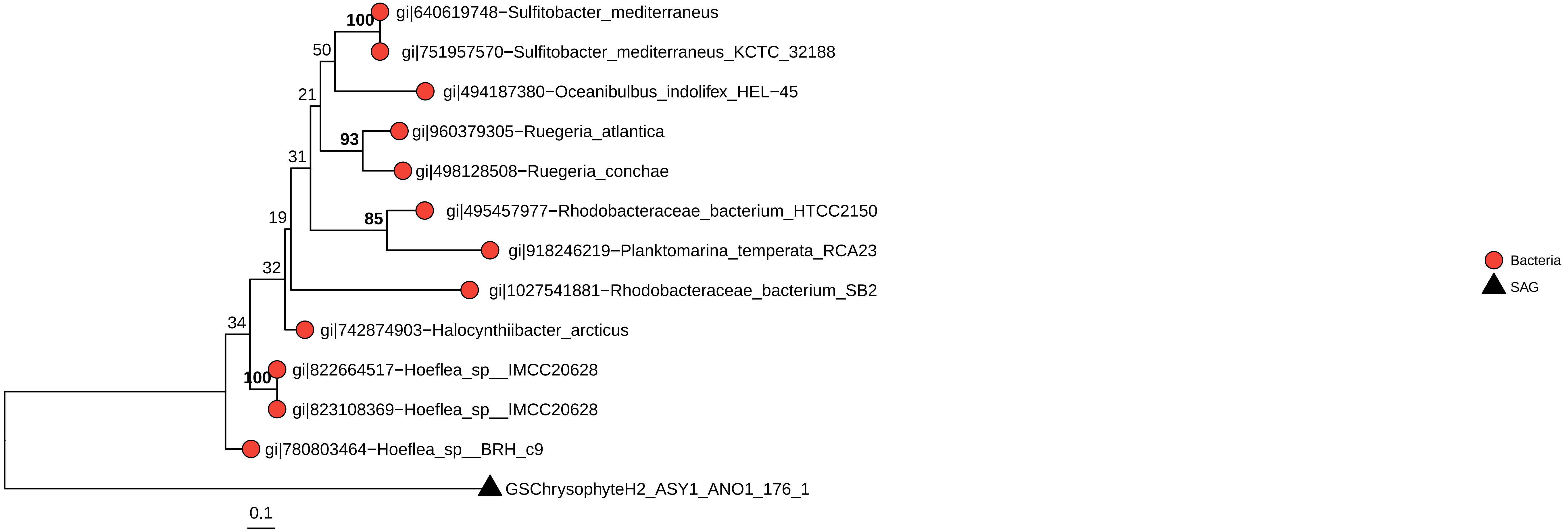
gi|640619748−Sulfitobacter_mediterraneus
gi|751957570−Sulfitobacter_mediterraneus_KCTC_32188
gi|494187380−Oceanibulbus_indolifex_HEL−45
gi|960379305−Ruegeria_atlantica
gi|498128508−Ruegeria_conchae
gi|495457977−Rhodobacteraceae_bacterium_HTCC2150
gi|918246219−Planktomarina_temperata_RCA23
gi|1027541881−Rhodobacteraceae_bacterium_SB2
gi|742874903−Halocynthiibacter_arcticus
gi|822664517−Hoeflea_sp__IMCC20628
gi|823108369−Hoeflea_sp__IMCC20628
gi|780803464−Hoeflea_sp__BRH_c9
GSChrysophyteH2_ASY1_ANO1_176_1
100
50
21
93
31
85
19
32
34
100
0.1
Bacteria
SAG

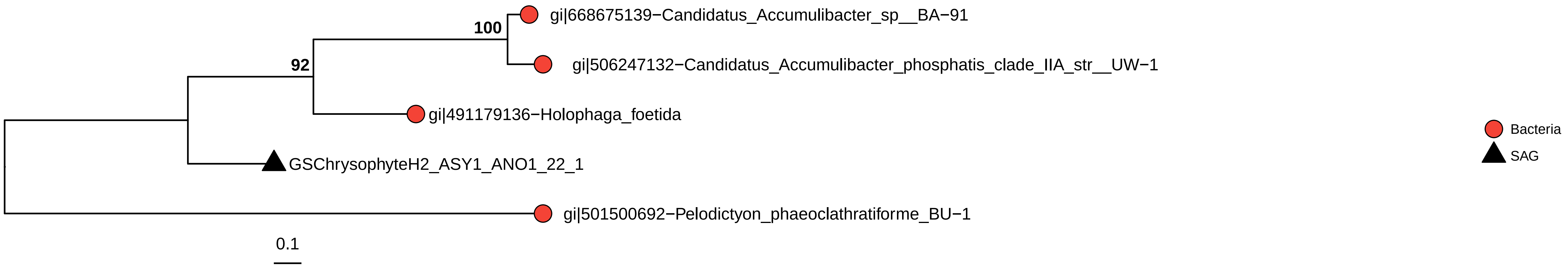

100
gi|668675139−Candidatus_Accumulibacter_sp__BA−91
92
gi|506247132−Candidatus_Accumulibacter_phosphatis_clade_IIA_str__UW−1
gi|491179136−Holophaga_foetida
GSChrysophyteH2_ASY1_ANO1_22_1
gi|501500692−Pelodictyon_phaeoclathratiforme_BU−1
0.1
Bacteria
SAG

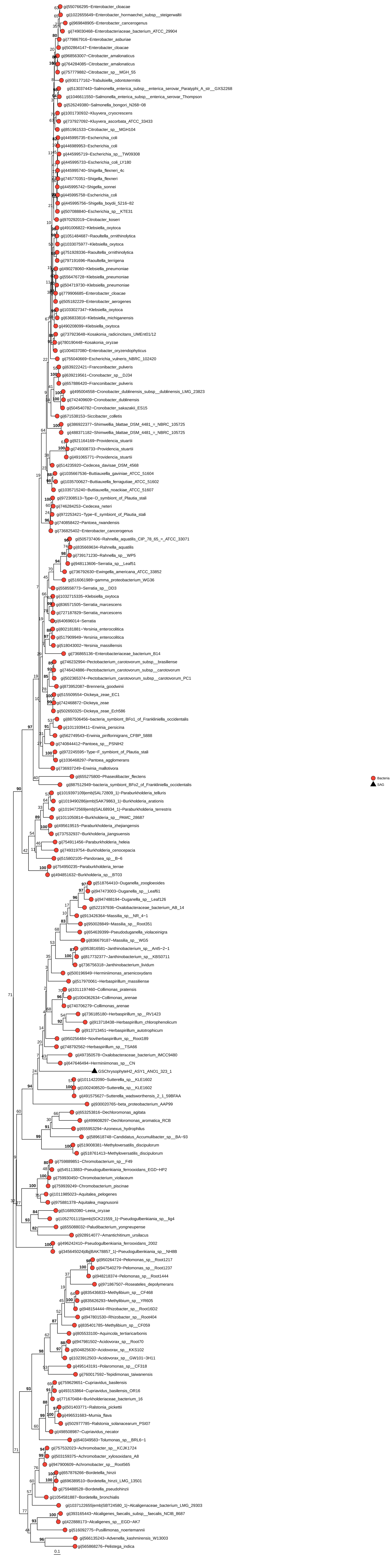

gi|550766295~Enterobacter_cloacae
gi|1022655649~Enterobacter_hormaechei_subsp__steigerwaltii
gi|969848905~Enterobacter_cancerogenus
gi|749030468~Enterobacteriaceae_bacterium_ATCC_29904
gi|779867916~Enterobacter_asburiae
gi|502864147~Enterobacter_cloacae
gi|968563007~Citrobacter_amalonaticus
gi|764284085~Citrobacter_amalonaticus
gi|757779882~Citrobacter_sp__MGH_55
gi|930177162~Trabulsiella_odontotermitis
gi|513037443~Salmonella_enterica_subsp__enterica_serovar_Paratyphi_A_str__GXS2268
gi|1046611550~Salmonella_enterica_subsp__enterica_serovar_Thompson
gi|526249380~Salmonella_bongori_N268-08
gi|1001730932~Kluyvera_cryocrescens
gi|737927092~Kluyvera_ascorbata_ATCC_33433
gi|851961533~Citrobacter_sp__MGH104
gi|445995735~Escherichia_coli
gi|446989953~Escherichia_coli
gi|445995719~Escherichia_sp__TW09308
gi|445995733~Escherichia_coli_LY180
gi|445995740~Shigella_flexneri_4c
gi|745770351~Shigella_flexneri
gi|445995742~Shigella_sonnei
gi|445995758~Escherichia_coli
gi|445995736~Shigella_boydii_5216-82
gi|507088840~Escherichia_sp__KTE31
gi|970292019~Citrobacter_koseri
gi|491006822~Klebsiella_oxytoca
gi|1051484687~Raoultella_ornithinolytica
gi|1033075977~Klebsiella_oxytoca
gi|751928336~Raoultella_ornithinolytica
gi|797191696~Raoultella_terrigena
gi|490278060~Klebsiella_pneumoniae
gi|556476728~Klebsiella_pneumoniae
gi|504719730~Klebsiella_pneumoniae
gi|779906685~Enterobacter_cloacae
gi|505182229~Enterobacter_aerogenes
gi|1033027347~Klebsiella_oxytoca
gi|636833816~Klebsiella_michiganensis
gi|490208099~Klebsiella_oxytoca
gi|737923648~Kosakonia_radicincitans_UMEnt01/12
gi|780190448~Kosakonia_oryzae
gi|1004037080~Enterobacter_oryzendophyticus
gi|755040669~Escherichia_vulneris_NBRC_102420
gi|639222421~Franconibacter_pulveris
gi|639219561~Cronobacter_sp__DJ34
gi|657886420~Franconibacter_pulveris
gi|495004558~Cronobacter_dublinensis_subsp__dublinensis_LMG_23823
gi|742409609~Cronobacter_dublinensis
gi|504540782~Cronobacter_sakazakii_ES15
gi|671538153~Siccibacter_colletis
gi|386322377~Shimwellia_blattae_DSM_4481_=_NBRC_105725
gi|488371182~Shimwellia_blattae_DSM_4481_=_NBRC_105725
gi|921164169~Providencia_stuartii
gi|749308733~Providencia_stuartii
gi|491065771~Providencia_stuartii
gi|514235920~Cedecea_davisae_DSM_4568
gi|1035667536~Buttiauxella_gaviniae_ATCC_51604
gi|1035700627~Buttiauxella_ferragutiae_ATCC_51602
gi|1035715240~Buttiauxella_noackiae_ATCC_51607
gi|972308513~Type-D_symbiont_of_Plautia_stali
gi|746284253~Cedecea_neteri
gi|972253421~Type-E_symbiont_of_Plautia_stali
gi|740858422~Pantoea_rwandensis
gi|736825402~Enterobacter_cancerogenus
gi|505737406~Rahnella_aquatilis_CIP_78_65_=_ATCC_33071
gi|835669634~Rahnella_aquatilis
gi|739171230~Rahnella_sp__WP5
gi|948113606~Serratia_sp__Leaf51
gi|736792630~Ewingella_americana_ATCC_33852
gi|516061989~gamma_proteobacterium_WG36
gi|558558773~Serratia_sp__DD3
gi|1032715335~Klebsiella_oxytoca
gi|836571505~Serratia_marcescens
gi|727187829~Serratia_marcescens
gi|640696014~Serratia
gi|802181881~Yersinia_enterocolitica
gi|517909949~Yersinia_enterocolitica
gi|518043002~Yersinia_massiliensis
gi|736865136~Enterobacteriaceae_bacterium_B14
gi|746232994~Pectobacterium_carotovorum_subsp__brasiliense
gi|746424886~Pectobacterium_carotovorum_subsp__carotovorum
gi|502365374~Pectobacterium_carotovorum_subsp__carotovorum_PC1
gi|873952087~Brenneria_goodwinii
gi|515509554~Dickeya_zeae_EC1
gi|742468872~Dickeya_zeae
gi|502650325~Dickeya_zeae_Ech586
gi|887506456~bacteria_symbiont_BFo1_of_Frankliniella_occidentalis
gi|1011939411~Erwinia_persicina
gi|562749543~Erwinia_piriflorinigrans_CFBP_5888
gi|740844412~Pantoea_sp__PSNIH2
gi|972245595~Type-F_symbiont_of_Plautia_stali
gi|1036468297~Pantoea_agglomerans
gi|736937249~Erwinia_mallotivora
gi|655275800~Phaseolibacter_flectens
gi|887512949~bacteria_symbiont_BFo2_of_Frankliniella_occidentalis
gi|1019397109|emb|SAL72809_1|~Paraburkholderia_telluris
gi|1019490286|emb|SAK79863_1|~Burkholderia_arationis
gi|1019472569|emb|SAL68934_1|~Paraburkholderia_terrestris
gi|1011050814~Burkholderia_sp__PAMC_28687
gi|495619515~Paraburkholderia_zhejiangensis
gi|737532937~Burkholderia_jiangsuensis
gi|754911456~Paraburkholderia_heleia
gi|749319754~Burkholderia_cenocepacia
gi|515802105~Pandoraea_sp__B-6
gi|754950235~Paraburkholderia_terrae
gi|494851632~Burkholderia_sp__BT03
gi|518764410~Duganella_zoogloeoides
gi|947473003~Duganella_sp__Leaf61
gi|947488194~Duganella_sp__Leaf126
gi|522197936~Oxalobacteraceae_bacterium_AB_14
gi|913426364~Massilia_sp__NR_4-1
gi|950028849~Massilia_sp__Root351
gi|654639399~Pseudoduganella_violaceinigra
gi|836679187~Massilia_sp__WG5
gi|953816581~Janthinobacterium_sp__Ant5-2-1
gi|817732377~Janthinobacterium_sp__KBS0711
gi|736756318~Janthinobacterium_lividum
gi|500196949~Herminiimonas_arsenicoxydans
gi|517970061~Herbaspirillum_massiliense
gi|1011197460~Collimonas_pratensis
gi|1004362634~Collimonas_arenae
gi|740706279~Collimonas_arenae
gi|736185180~Herbaspirillum_sp__RV1423
gi|913718438~Herbaspirillum_chlorophenolicum
gi|913713451~Herbaspirillum_autotrophicum
gi|950256484~Noviherbaspirillum_sp__Root189
gi|748792562~Herbaspirillum_sp__TSA66
gi|497350578~Oxalobacteraceae_bacterium_IMCC9480
gi|647646494~Herminiimonas_sp__CN
GSChrysophyteH2_ASY1_ANO1_323_1
gi|1011422090~Sutterella_sp__KLE1602
gi|1002408520~Sutterella_sp__KLE1602
gi|491575627~Sutterella_wadsworthensis_2_1_59BFAA
gi|930020765~beta_proteobacterium_AAP99
gi|653253816~Dechloromonas_agitata
gi|499608297~Dechloromonas_aromatica_RCB
gi|655953294~Azonexus_hydrophilus
gi|589618748~Candidatus_Accumulibacter_sp__BA-93
gi|519008381~Methyloversatilis_discipulorum
gi|518761413~Methyloversatilis_discipulorum
gi|759889851~Chromobacterium_sp__F49
gi|545113883~Pseudogulbenkiania_ferrooxidans_EGD-HP2
gi|759930450~Chromobacterium_violaceum
gi|759939249~Chromobacterium_piscinae
gi|1011985023~Aquitalea_pelogenes
gi|759881378~Aquitalea_magnusonii
gi|516892080~Leeia_oryzae
gi|1052701115|emb|SCK21559_1|~Pseudogulbenkiania_sp__Ilg4
gi|655088032~Paludibacterium_yongneupense
gi|928914077~Amantichitinum_ursilacus
gi|496242410~Pseudogulbenkiania_ferrooxidans_2002
gi|345645024|dbj|BAK78857_1|~Pseudogulbenkiania_sp__NH8B
gi|950264724~Pelomonas_sp__Root1217
gi|947540279~Pelomonas_sp__Root1237
gi|948218374~Pelomonas_sp__Root1444
gi|971867507~Roseateles_depolymerans
gi|835436833~Methylibium_sp__CF468
gi|835626293~Methylibium_sp__YR605
gi|948154444~Rhizobacter_sp__Root16D2
gi|947801530~Rhizobacter_sp__Root404
gi|835401785~Methylibium_sp__CF059
gi|805533100~Aquincola_tertiaricarbonis
gi|947981502~Acidovorax_sp__Root70
gi|504825630~Acidovorax_sp__KKS102
gi|1023912503~Acidovorax_sp__GW101-3H11
gi|495143191~Polaromonas_sp__CF318
gi|760017592~Tepidimonas_taiwanensis
gi|759629651~Cupriavidus_basilensis
gi|493153864~Cupriavidus_basilensis_OR16
gi|771670484~Burkholderiaceae_bacterium_16
gi|501403771~Ralstonia_pickettii
gi|496531683~Mumia_flava
gi|502977785~Ralstonia_solanacearum_PSI07
gi|498508987~Cupriavidus_necator
gi|640349583~Tolumonas_sp__BRL6-1
gi|757532023~Achromobacter_sp__KCJK1724
gi|503159375~Achromobacter_xylosoxidans_A8
gi|947900609~Achromobacter_sp__Root565
gi|657876266~Bordetella_hinzii
gi|696389510~Bordetella_hinzii_LMG_13501
gi|759488528~Bordetella_pseudohinzii
gi|1054581887~Bordetella_bronchialis
gi|1037122655|emb|SBT24580_1|~Alcaligenaceae_bacterium_LMG_29303
gi|393165443~Alcaligenes_faecalis_subsp__faecalis_NCIB_8687
gi|422888173~Alcaligenes_sp__EGD-AK7
gi|516092775~Pusillimonas_noertemannii
gi|566135243~Advenella_kashmirensis_W13003
gi|565868276~Pelistega_indica
0.1
Bacteria
SAG

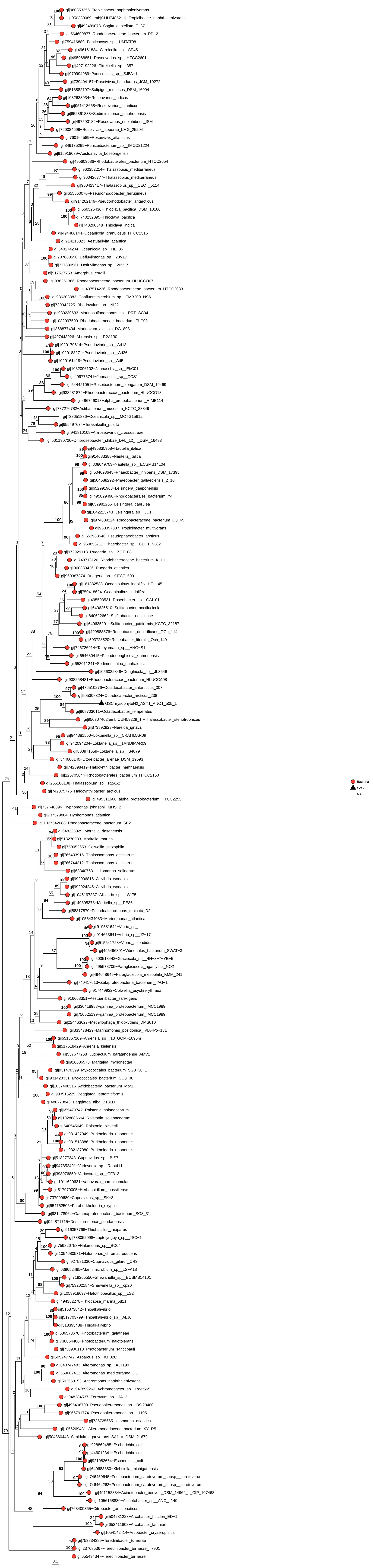
gi|960353355~Tropicibacter_naphthalenivorans
gi|950330089|emb|CUH74852_1|~Tropicibacter_naphthalenivorans
gi|492489073~Sagittula_stellata_E~37
gi|564609877~Rhodobacteraceae_bacterium_PD~2
gi|759416889~Ponticoccus_sp__UMTAT08
gi|496161834~Citreicella_sp__SE45
gi|495068851~Roseovarius_sp__HTCC2601
gi|497192226~Citreicella_sp__357
gi|970994989~Ponticoccus_sp__SJ5A~1
gi|739404157~Roseivivax_halodurans_JCM_10272
gi|518882707~Salipiger_mucosus_DSM_16094
gi|1032638934~Roseovarius_indicus
gi|951418658~Roseovarius_atlanticus
gi|652361833~Sediminimonas_qiaohouensis
gi|497500184~Roseovarius_nubinhibens_ISM
gi|760084696~Roseivivax_isoporae_LMG_25204
gi|760164589~Roseivivax_atlanticus
gi|849135299~Puniceibacterium_sp__IMCC21224
gi|915918039~Aestuariivita_boseongensis
gi|495603586~Rhodobacterales_bacterium_HTCC2654
gi|960352214~Thalassobius_mediterraneus
gi|960426777~Thalassobius_mediterraneus
gi|960423417~Thalassobius_sp__CECT_5114
gi|655560070~Pseudorhodobacter_ferrugineus
gi|914202146~Pseudorhodobacter_antarcticus
gi|660526436~Thioclava_pacifica_DSM_10166
gi|740232095~Thioclava_pacifica
gi|740290548~Thioclava_indica
gi|494466144~Oceanicola_granulosus_HTCC2516
gi|914213823~Aestuariivita_atlantica
gi|640174234~Oceanicola_sp__HL~35
gi|737880596~Defluviimonas_sp__20V17
gi|737880561~Defluviimonas_sp__20V17
gi|517527753~Amorphus_coralli
gi|938251366~Rhodobacteraceae_bacterium_HLUCCO07
gi|497514236~Rhodobacterales_bacterium_HTCC2083
gi|936203883~Confluentimicrobium_sp__EMB200~NS6
gi|739342725~Rhodovulum_sp__NI22
gi|939230633~Marinosulfonomonas_sp__PRT~SC04
gi|1032097500~Rhodobacteraceae_bacterium_EhC02
gi|868877434~Marinovum_algicola_DG_898
gi|497443926~Ahrensia_sp__R2A130
gi|1020170614~Pseudovibrio_sp__Ad13
gi|1020183271~Pseudovibrio_sp__Ad26
gi|1020161419~Pseudovibrio_sp__Ad5
gi|1032096102~Jannaschia_sp__EhC01
gi|499775741~Jannaschia_sp__CCS1
gi|644421051~Roseibacterium_elongatum_DSM_19469
gi|938281874~Rhodobacteraceae_bacterium_HLUCCO18
gi|496746018~alpha_proteobacterium_HIMB114
gi|737276782~Actibacterium_mucosum_KCTC_23349
gi|738651886~Oceanicola_sp__S124
gi|655497674~Tetrasakiella_pusilla
gi|941810109~Aliiroseovarius_crassostreae
gi|501130720~Dinoroseobacter_shibae_DFL_12_=_DSM_16493
gi|495835358~Nautella_italica
gi|914683388~Nautella_italica
gi|808049703~Nautella_sp__ECSMB14104
gi|504693645~Phaeobacter_inhibens_DSM_17395
gi|504688292~Phaeobacter_gallaeciensis_2_10
gi|652991963~Leisingera_daeponensis
gi|495829490~Rhodobacterales_bacterium_Y4I
gi|652982265~Leisingera_caerulea
gi|1042213743~Leisingera_sp__JC1
gi|974809224~Rhodobacteraceae_bacterium_O3_65
gi|960397807~Tropicibacter_multivorans
gi|652988546~Pseudophaeobacter_arcticus
gi|960856712~Phaeobacter_sp__CECT_5382
gi|972929118~Ruegeria_sp__ZGT108
gi|748713120~Rhodobacteraceae_bacterium_KLH11
gi|960383426~Ruegeria_atlantica
gi|960387874~Ruegeria_sp__CECT_5091
gi|161382538~Oceanibulbus_indolifex_HEL~45
gi|750418624~Oceanibulbus_indolifex
gi|495503531~Roseobacter_sp__GAI101
gi|640626510~Sulfitobacter_noctilucicola
gi|640622662~Sulfitobacter_noctilucae
gi|640635291~Sulfitobacter_guttiformis_KCTC_32187
gi|499888876~Roseobacter_denitrificans_OCh_114
gi|503728520~Roseobacter_litoralis_Och_149
gi|746726914~Tateyamaria_sp__ANG~S1
gi|654630415~Pseudodonghicola_xiamenensis
gi|653011241~Sedimentitalea_nanhaiensis
gi|1056022849~Donghicola_sp__JL3646
gi|938258481~Rhodobacteraceae_bacterium_HLUCCA08
gi|476510276~Octadecabacter_antarcticus_307
gi|505308324~Octadecabacter_arcticus_238
GSChrysophyteH2_ASY1_ANO1_505_1
gi|908703011~Octadecabacter_temperatus
gi|950307402|emb|CUH59229_1|~Thalassobacter_stenotrophicus
gi|873892923~Nereida_ignava
gi|944381550~Loktanella_sp__SRATIMAR09
gi|942094204~Loktanella_sp__1ANDIMAR09
gi|800971659~Loktanella_sp__S4079
gi|544666140~Litoreibacter_arenae_DSM_19593
gi|742898419~Halocynthiibacter_namhaensis
gi|126705044~Rhodobacterales_bacterium_HTCC2150
gi|255106108~Thalassobium_sp__R2A62
gi|742875776~Halocynthiibacter_arcticus
gi|495311606~alpha_proteobacterium_HTCC2255
gi|737648896~Hyphomonas_johnsonii_MHS~2
gi|737579804~Hyphomonas_atlantica
gi|1027542088~Rhodobacteraceae_bacterium_SB2
gi|648225029~Moritella_dasanensis
gi|518270933~Moritella_marina
gi|750052653~Colwellia_piezophila
gi|765433915~Thalassomonas_actiniarum
gi|766744312~Thalassomonas_actiniarum
gi|683407631~Idiomarina_salinarum
gi|992006816~Aliivibrio_wodanis
gi|992024246~Aliivibrio_wodanis
gi|1046197337~Aliivibrio_sp__1S175
gi|149805378~Moritella_sp__PE36
gi|88817870~Pseudoalteromonas_tunicata_D2
gi|1055434083~Marinomonas_atlantica
gi|919581642~Vibrio_sp_
gi|914663641~Vibrio_sp__J2~17
gi|515641728~Vibrio_splendidus
gi|495496801~Vibrionales_bacterium_SWAT~3
gi|503518442~Glaciecola_sp__4H~3~7+YE~5
gi|495578705~Paraglaciecola_agarilytica_NO2
gi|494048649~Paraglaciecola_mesophila_KMM_241
gi|740417613~Zetaproteobacteria_bacterium_TAG~1
gi|917449932~Colwellia_psychrerythraea
gi|916668351~Aestuariibacter_salexigens
gi|330418958~gamma_proteobacterium_IMCC1989
gi|750525199~gamma_proteobacterium_IMCC1989
gi|224463627~Methylophaga_thiooxydans_DMS010
gi|333478429~Marinomonas_posidonica_IVIA~Po~181
gi|651367109~Ahrensia_sp__13_GOM~1096m
gi|571518429~Ahrensia_kielensis
gi|557677258~Lutibaculum_baratangense_AMV1
gi|916606573~Mantalea_myrionectae
gi|931470399~Myxococcales_bacterium_SG8_38_1
gi|931428331~Myxococcales_bacterium_SG8_38
gi|1037408516~Acidobacteria_bacterium_Mor1
gi|933515225~Beggiatoa_leptomitiformis
gi|488778843~Beggiatoa_alba_B18LD
gi|655479742~Ralstonia_solanacearum
gi|1028885694~Ralstonia_solanacearum
gi|640545649~Ralstonia_pickettii
gi|981427949~Burkholderia_ubonensis
gi|981518886~Burkholderia_ubonensis
gi|982137080~Burkholderia_ubonensis
gi|518277348~Cupriavidus_sp__BIS7
gi|947852491~Variovorax_sp__Root411
gi|398076850~Variovorax_sp__CF313
gi|1011620631~Variovorax_boronicumulans
gi|517970005~Herbaspirillum_massiliense
gi|737909680~Cupriavidus_sp__SK~3
gi|654762506~Paraburkholderia_oxyphila
gi|931478964~Gammaproteobacteria_bacterium_SG8_31
gi|924871715~Desulfuromonas_soudanensis
gi|916357766~Thiobacillus_thioparus
gi|738052098~Leptolyngbya_sp__JSC~1
gi|759820758~Halomonas_sp__BC04
gi|1054680571~Halomonas_chromatireducens
gi|927581330~Cupriavidus_gilardii_CR3
gi|639052495~Marinimicrobium_sp__LS~A18
gi|719265550~Shewanella_sp__ECSMB14101
gi|753202184~Shewanella_sp__cp20
gi|1053918697~Halothiobacillus_sp__LS2
gi|494352278~Thiocapsa_marina_5811
gi|518373842~Thioalkalivibrio
gi|517703799~Thioalkalivibrio_sp__ALJ6
gi|518393488~Thioalkalivibrio
gi|636573678~Photobacterium_galatheae
gi|738864400~Photobacterium_halotolerans
gi|738930113~Photobacterium_sanctipauli
gi|505247742~Azoarcus_sp__KH32C
gi|643747483~Alteromonas_sp__ALT199
gi|559062412~Alteromonas_mediterranea_DE
gi|503550153~Alteromonas_naphthalenivorans
gi|947899262~Achromobacter_sp__Root565
gi|948284537~Ferrovum_sp__JA12
gi|495406799~Pseudoalteromonas_sp__BSi20480
gi|966791774~Pseudoalteromonas_sp__H105
gi|736725665~Idiomarina_atlantica
gi|1056269431~Alteromonadaceae_bacterium_XY~R5
gi|504860443~Simiduia_agarivorans_SA1_=_DSM_21679
gi|928869485~Escherichia_coli
gi|446012341~Escherichia_coli
gi|921982664~Escherichia_coli
gi|640683880~Klebsiella_michiganensis
gi|746459645~Pectobacterium_carotovorum_subsp__carotovorum
gi|746464263~Pectobacterium_carotovorum_subsp__carotovorum
gi|491152834~Acinetobacter_bouvetii_DSM_14964_=_CIP_107468
gi|1056168830~Acinetobacter_sp__ANC_4149
gi|763409355~Citrobacter_amalonaticus
gi|504281223~Arcobacter_butzleri_ED~1
gi|652411808~Arcobacter_lanthieri
gi|1054142414~Arcobacter_cryaerophilus
gi|753834389~Teredinibacter_turnerae
gi|237685367~Teredinibacter_turnerae_T7901
gi|655494347~Teredinibacter_turnerae
0.1
Bacteria
SAG
NA

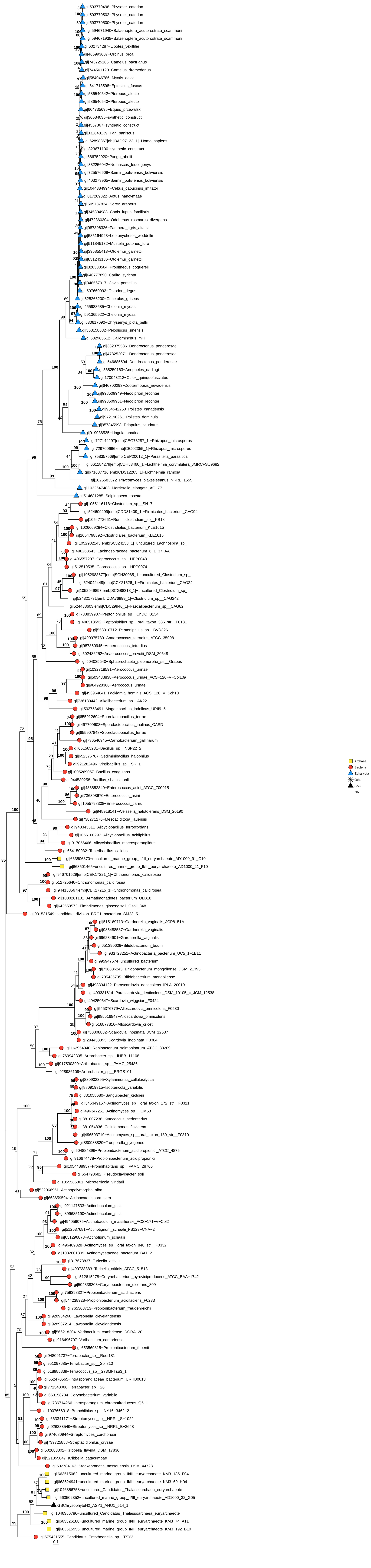

gi|593770498~Physeter_catodon
gi|593770502~Physeter_catodon
gi|593770500~Physeter_catodon
gi|594671940~Balaenoptera_acutorostrata_scammoni
gi|594671938~Balaenoptera_acutorostrata_scammoni
gi|602734287~Lipotes_vexillifer
gi|465993607~Orcinus_orca
gi|743725166~Camelus_bactrianus
gi|744561120~Camelus_dromedarius
gi|584046786~Myotis_davidii
gi|641713598~Eptesicus_fuscus
gi|586540542~Pteropus_alecto
gi|586540540~Pteropus_alecto
gi|664735695~Equus_przewalskii
gi|30584035~synthetic_construct
gi|4557367~synthetic_construct
gi|332848139~Pan_paniscus
gi|62898367|dbj|BAD97123_1|~Homo_sapiens
gi|823671100~synthetic_construct
gi|686752920~Pongo_abelii
gi|332256042~Nomascus_leucogenys
gi|725576609~Saimiri_boliviensis_boliviensis
gi|403279965~Saimiri_boliviensis_boliviensis
gi|1044384994~Cebus_capucinus_imitator
gi|817269322~Aotus_nancymaae
gi|505787824~Sorex_araneus
gi|345804988~Canis_lupus_familiaris
gi|472360304~Odobenus_rosmarus_divergens
gi|987396326~Panthera_tigris_altaica
gi|585164923~Leptonychotes_weddellii
gi|511845132~Mustela_putorius_furo
gi|395855413~Otolemur_garnettii
gi|831243186~Otolemur_garnettii
gi|826330504~Propithecus_coquereli
gi|640777890~Carlito_syrichta
gi|348567917~Cavia_porcellus
gi|507660992~Octodon_degus
gi|625266200~Cricetulus_griseus
gi|465988685~Chelonia_mydas
gi|591365922~Chelonia_mydas
gi|530617090~Chrysemys_picta_bellii
gi|558158632~Pelodiscus_sinensis
gi|632965612~Callorhinchus_milii
gi|332375536~Dendroctonus_ponderosae
gi|478252071~Dendroctonus_ponderosae
gi|546685594~Dendroctonus_ponderosae
gi|558250163~Anopheles_darlingi
gi|170043212~Culex_quinquefasciatus
gi|646700293~Zootermopsis_nevadensis
gi|998509949~Neodiprion_lecontei
gi|998509951~Neodiprion_lecontei
gi|954542253~Polistes_canadensis
gi|972190261~Polistes_dominula
gi|957845998~Priapulus_caudatus
gi|919086535~Lingula_anatina
gi|727144297|emb|CEG73287_1|~Rhizopus_microsporus
gi|729700666|emb|CEJ02355_1|~Rhizopus_microsporus
gi|758357569|emb|CEP20012_1|~Parasitella_parasitica
gi|661184279|emb|CDH53460_1|~Lichtheimia_corymbifera_JMRCFSU9682
gi|671687716|emb|CDS12265_1|~Lichtheimia_ramosa
gi|1026583572~Phycomyces_blakesleeanus_NRRL_1555~
gi|1032647483~Mortierella_elongata_AG~77
gi|514681285~Salpingoeca_rosetta
gi|1055116118~Clostridium_sp__SN17
gi|524609299|emb|CDD31409_1|~Firmicutes_bacterium_CAG94
gi|1054772661~Ruminiclostridium_sp__KB18
gi|1026669284~Clostridiales_bacterium_KLE1615
gi|1054798892~Clostridiales_bacterium_KLE1615
gi|1052932145|emb|SCJ24133_1|~uncultured_Lachnospira_sp_
gi|496263543~Lachnospiraceae_bacterium_6_1_37FAA
gi|496557207~Coprococcus_sp__HPP0048
gi|512510535~Coprococcus_sp__HPP0074
gi|1052983677|emb|SCH30085_1|~uncultured_Clostridium_sp_
gi|524042449|emb|CCY21526_1|~Firmicutes_bacterium_CAG24
gi|1052949893|emb|SCG88318_1|~uncultured_Clostridium_sp_
gi|524321731|emb|CDA76999_1|~Clostridium_sp__CAG242
gi|524488603|emb|CDC29946_1|~Faecalibacterium_sp__CAG82
gi|738839907~Peptoniphilus_sp__ChDC_B134
gi|496513592~Peptoniphilus_sp__oral_taxon_386_str__F0131
gi|553310712~Peptoniphilus_sp__BV3C26
gi|490975789~Anaerococcus_tetradius_ATCC_35098
gi|987860945~Anaerococcus_tetradius
gi|502486252~Anaerococcus_prevotii_DSM_20548
gi|504035540~Sphaerochaeta_pleomorpha_str__Grapes
gi|1032718591~Aerococcus_urinae
gi|503433838~Aerococcus_urinae_ACS~120~V~Col10a
gi|984928366~Aerococcus_urinae
gi|493964641~Facklamia_hominis_ACS~120~V~Sch10
gi|736189442~Alkalibacterium_sp__AK22
gi|502758491~Mageeibacillus_indolicus_UPII9~5
gi|655912694~Sporolactobacillus_terrae
gi|497709608~Sporolactobacillus_inulinus_CASD
gi|655907848~Sporolactobacillus_terrae
gi|736546945~Carnobacterium_gallinarum
gi|651565231~Bacillus_sp__NSP22_2
gi|652375767~Sediminibacillus_halophilus
gi|921282496~Virgibacillus_sp__SK~1
gi|1005269057~Bacillus_coagulans
gi|944530258~Bacillus_shackletonii
gi|486852849~Enterococcus_asini_ATCC_700915
gi|736808670~Enterococcus_asini
gi|1055798308~Enterococcus_canis
gi|948918141~Weissella_halotolerans_DSM_20190
gi|738271276~Mesoaciditoga_lauensis
gi|940343311~Alicyclobacillus_ferrooxydans
gi|1056100297~Alicyclobacillus_acidiphilus
gi|917056466~Alicyclobacillus_macrosporangiidus
gi|654150032~Tuberibacillus_calidus
gi|663506370~uncultured_marine_group_II/III_euryarchaeote_AD1000_91_C10
gi|663501465~uncultured_marine_group_II/III_euryarchaeote_AD1000_21_F10
gi|946701529|emb|CEK17221_1|~Chthonomonas_calidirosea
gi|512725640~Chthonomonas_calidirosea
gi|944158567|emb|CEK17215_1|~Chthonomonas_calidirosea
gi|1000261101~Armatimonadetes_bacterium_OLB18
gi|643550573~Fimbriimonas_ginsengisoli_Gsoil_348
gi|931531549~candidate_division_BRC1_bacterium_SM23_51
gi|515169713~Gardnerella_vaginalis_JCP8151A
gi|985488537~Gardnerella_vaginalis
gi|669234901~Gardnerella_vaginalis
gi|651390609~Bifidobacterium_boum
gi|933723251~Actinobacteria_bacterium_UC5_1~1B11
gi|995947574~uncultured_bacterium
gi|736886243~Bifidobacterium_mongoliense_DSM_21395
gi|705435795~Bifidobacterium_mongoliense
gi|493334122~Parascardovia_denticolens_IPLA_20019
gi|493331614~Parascardovia_denticolens_DSM_10105_=_JCM_12538
gi|494250547~Scardovia_wiggsiae_F0424
gi|545376779~Alloscardovia_omnicolens_F0580
gi|985516843~Alloscardovia_omnicolens
gi|651877816~Alloscardovia_criceti
gi|750308882~Scardovia_inopinata_JCM_12537
gi|294458353~Scardovia_inopinata_F0304
gi|162954940~Renibacterium_salmoninarum_ATCC_33209
gi|769942305~Arthrobacter_sp__IHBB_11108
gi|917530399~Arthrobacter_sp__PAMC_25486
gi|928986109~Arthrobacter_sp__ERGS1:01
gi|880902395~Xylanimonas_cellulosilytica
gi|880919315~Isoptericola_variabilis
gi|881058680~Sanguibacter_keddieii
gi|545349157~Actinomyces_sp__oral_taxon_172_str__F0311
gi|496347251~Actinomyces_sp__ICM58
gi|881007238~Kytococcus_sedentarius
gi|881054836~Cellulomonas_flavigena
gi|496503719~Actinomyces_sp__oral_taxon_180_str__F0310
gi|880988829~Trueperella_pyogenes
gi|504884896~Propionibacterium_acidipropionici_ATCC_4875
gi|916674478~Propionibacterium_acidipropionici
gi|1054488957~Frondihabitans_sp__PAMC_28766
gi|654790682~Pseudoclavibacter_soli
gi|1055585861~Microterricola_viridarii
gi|522066951~Actinopolymorpha_alba
gi|663659594~Actinocatenispora_sera
gi|921147533~Actinobaculum_suis
gi|899685190~Actinobaculum_suis
gi|494059075~Actinobaculum_massiliense_ACS~171~V~Col2
gi|512537681~Actinotignum_schaalii_FB123~CNA~2
gi|926876878~Actinotignum_schaalii
gi|496489328~Actinomyces_sp__oral_taxon_848_str__F0332
gi|1032601309~Actinomycetaceae_bacterium_BA112
gi|817678837~Turicella_otitidis
gi|490738883~Turicella_otitidis_ATCC_51513
gi|512615278~Corynebacterium_pyruviciproducens_ATCC_BAA~1742
gi|504338203~Corynebacterium_ulcerans_809
gi|759398327~Propionibacterium_acidifaciens
gi|554238928~Propionibacterium_acidifaciens_F0233
gi|765308713~Propionibacterium_freudenreichii
gi|928954260~Lawsonella_clevelandensis
gi|928937214~Lawsonella_clevelandensis
gi|656218204~Varibaculum_cambriense_DORA_20
gi|916496707~Varibaculum_cambriense
gi|653569815~Propionibacterium_thoenii
gi|948091737~Terrabacter_sp__Root181
gi|951097685~Terrabacter_sp__Soil810
gi|518985839~Terracoccus_sp__273MFTsu3_1
gi|652470565~Intrasporangiaceae_bacterium_URHB0013
gi|771548086~Terrabacter_sp__28
gi|663158734~Corynebacterium_variabile
gi|736714266~Intrasporangium_chromatireducens_Q5~1
gi|1007666318~Branchiibius_sp__NY16~3462~2
gi|663341171~Streptomyces_sp__NRRL_S~1022
gi|926383549~Streptomyces_sp__NRRL_B~3648
gi|974680944~Streptomyces_corchorusii
gi|739725858~Streptacidiphilus_oryzae
gi|502683302~Kribbella_flavida_DSM_17836
gi|521055047~Kribbella_catacumbae
gi|502784162~Stackebrandtia_nassauensis_DSM_44728
gi|663515082~uncultured_marine_group_II/III_euryarchaeote_KM3_185_F04
gi|663524941~uncultured_marine_group_II/III_euryarchaeote_KM3_69_H04
gi|1046356758~uncultured_Candidatus_Thalassoarchaea_euryarchaeote
gi|663502352~uncultured_marine_group_II/III_euryarchaeote_AD1000_32_G05
SCChrysophyteH2_ASY1_ANO1_514_1
gi|1046356786~uncultured_Candidatus_Thalassoarchaea_euryarchaeote
gi|663526188~uncultured_marine_group_II/III_euryarchaeote_KM3_74_A11
gi|663515955~uncultured_marine_group_II/III_euryarchaeote_KM3_192_B10
gi|575421555~Candidatus_Entotheonella_sp__TSY2
0.1
Archaea
Bacteria
Eukaryota
Other
SAG
NA

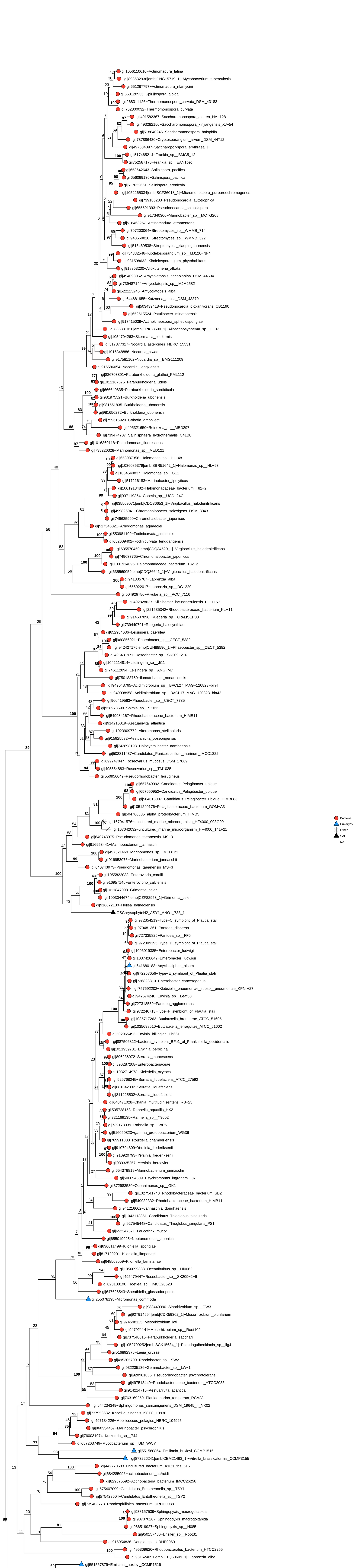
Bacteria
Eukaryota
Other
SAG
NA
0.2

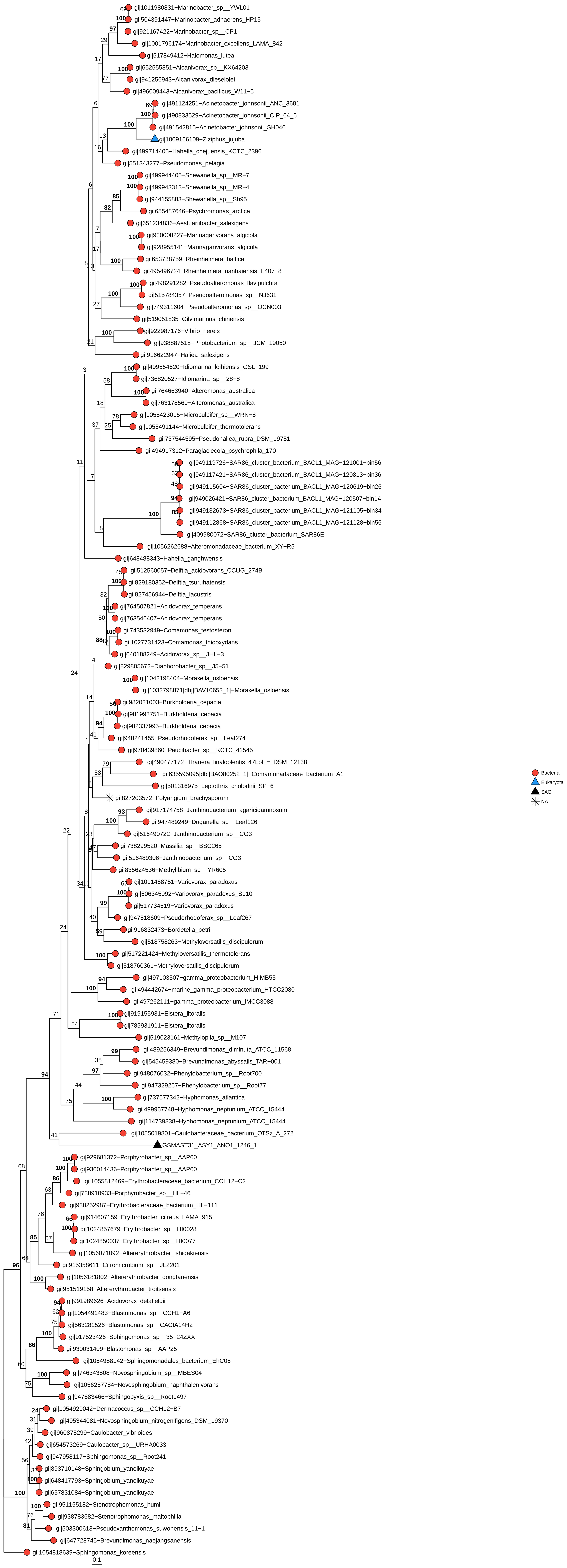
gi|1011980831−Marinobacter_sp__YWL01
gi|504391447−Marinobacter_adhaerens_HP15
gi|921167422−Marinobacter_sp__CP1
gi|1001796174−Marinobacter_excellens_LAMA_842
gi|517849412−Halomonas_lutea
gi|652555851−Alcanivorax_sp__KX64203
gi|941256943−Alcanivorax_dieselolei
gi|496009443−Alcanivorax_pacificus_W11−5
gi|491124251−Acinetobacter_johnsonii_ANC_3681
gi|490833529−Acinetobacter_johnsonii_CIP_64_6
gi|491542815−Acinetobacter_johnsonii_SH046
gi|1009166109−Ziziphus_jujuba
gi|499714405−Hahella_chejuensis_KCTC_2396
gi|551343277−Pseudomonas_pelagia
gi|499944405−Shewanella_sp__MR−7
gi|499943313−Shewanella_sp__MR−4
gi|944155883−Shewanella_sp__Sh95
gi|655487646−Psychromonas_arctica
gi|651234836−Aestuariibacter_salexigens
gi|930008227−Marinagarivorans_algicola
gi|928955141−Marinagarivorans_algicola
gi|653738759−Rheinheimera_baltica
gi|495496724−Rheinheimera_nanhaiensis_E407−8
gi|498291282−Pseudoalteromonas_flavipulchra
gi|515784357−Pseudoalteromonas_sp__NJ631
gi|749311604−Pseudoalteromonas_sp__OCN003
gi|519051835−Gilvimarinus_chinensis
gi|922987176−Vibrio_nereis
gi|938887518−Photobacterium_sp__JCM_19050
gi|916622947−Haliea_salexigens
gi|499554620−Idiomarina_loihiensis_GSL_199
gi|736820527−Idiomarina_sp__28−8
gi|764663940−Alteromonas_australica
gi|763178569−Alteromonas_australica
gi|1055423015−Microbulbifer_sp__WRN−8
gi|1055491144−Microbulbifer_thermotolerans
gi|737544595−Pseudohaliea_rubra_DSM_19751
gi|494917312−Paraglaciecola_psychrophila_170
gi|949119726−SAR86_cluster_bacterium_BACL1_MAG−121001−bin56
gi|949117421−SAR86_cluster_bacterium_BACL1_MAG−120813−bin36
gi|949115604−SAR86_cluster_bacterium_BACL1_MAG−120619−bin26
gi|949026421−SAR86_cluster_bacterium_BACL1_MAG−120507−bin14
gi|949132673−SAR86_cluster_bacterium_BACL1_MAG−121105−bin34
gi|949112868−SAR86_cluster_bacterium_BACL1_MAG−121128−bin56
gi|409980072−SAR86_cluster_bacterium_SAR86E
gi|1056262688−Alteromonadaceae_bacterium_XY−R5
gi|648488343−Hahella_ganghwensis
gi|512560057−Delftia_acidovorans_CCUG_274B
gi|829180352−Delftia_tsuruhatensis
gi|827456944−Delftia_lacustris
gi|764507821−Acidovorax_temperans
gi|763546407−Acidovorax_temperans
gi|743532949−Comamonas_testosteroni
gi|1027731423−Comamonas_thiooxydans
gi|640188249−Acidovorax_sp__JHL−3
gi|829805672−Diaphorobacter_sp__J5−51
gi|1042198404−Moraxella_osloensis
gi|1032798871|dbj|BAV10653_1|−Moraxella_osloensis
gi|982021003−Burkholderia_cepacia
gi|981993751−Burkholderia_cepacia
gi|982337995−Burkholderia_cepacia
gi|948241455−Pseudorhodoferax_sp__Leaf274
gi|970439860−Paucibacter_sp__KCTC_42545
gi|490477172−Thauera_linaloolentis_47Lol_=_DSM_12138
gi|635595095|dbj|BAO80252_1|−Comamonadaceae_bacterium_A1
gi|501316975−Leptothrix_cholodnii_SP−6
gi|827203572−Polyangium_brachysporum
gi|917174758−Janthinobacterium_agaricidamnosum
gi|947489249−Duganella_sp__Leaf126
gi|516490722−Janthinobacterium_sp__CG3
gi|738299520−Massilia_sp__BSC265
gi|516489306−Janthinobacterium_sp__CG3
gi|835624536−Methylibium_sp__YR605
gi|1011468751−Variovorax_paradoxus
gi|506345992−Variovorax_paradoxus_S110
gi|517734519−Variovorax_paradoxus
gi|947518609−Pseudorhodoferax_sp__Leaf267
gi|916832473−Bordetella_petrii
gi|518758263−Methyloversatilis_discipulorum
gi|517221424−Methyloversatilis_thermotolerans
gi|518760361−Methyloversatilis_discipulorum
gi|497103507−gamma_proteobacterium_HIMB55
gi|494442674−marine_gamma_proteobacterium_HTCC2080
gi|497262111−gamma_proteobacterium_IMCC3088
gi|919155931−Elstera_litoralis
gi|785931911−Elstera_litoralis
gi|519023161−Methylopila_sp__M107
gi|489256349−Brevundimonas_diminuta_ATCC_11568
gi|545459380−Brevundimonas_abyssalis_TAR−001
gi|948076032−Phenylobacterium_sp__Root700
gi|947329267−Phenylobacterium_sp__Root77
gi|737577342−Hyphomonas_atlantica
gi|499967748−Hyphomonas_neptunium_ATCC_15444
gi|114739838−Hyphomonas_neptunium_ATCC_15444
gi|1055019801−Caulobacteraceae_bacterium_OTSz_A_272
GSMAST31_ASY1_ANO1_1246_1
gi|929681372−Porphyrobacter_sp__AAP60
gi|930014436−Porphyrobacter_sp__AAP60
gi|1055812469−Erythrobacteraceae_bacterium_CCH12−C2
gi|738910933−Porphyrobacter_sp__HL−46
gi|938252987−Erythrobacteraceae_bacterium_HL−111
gi|914607159−Erythrobacter_citreus_LAMA_915
gi|1024857679−Erythrobacter_sp__HI0028
gi|1024850037−Erythrobacter_sp__HI0077
gi|1056071092−Altererythrobacter_ishigakiensis
gi|915358611−Citromicrobium_sp__JL2201
gi|1056181802−Altererythrobacter_dongtanensis
gi|951519158−Altererythrobacter_troitsensis
gi|991989626−Acidovorax_delafieldii
gi|1054491483−Blastomonas_sp__CCH1−A6
gi|563281526−Blastomonas_sp__CACIA14H2
gi|917523426−Sphingomonas_sp__35−24ZXX
gi|930031409−Blastomonas_sp__AAP25
gi|1054988142−Sphingomonadales_bacterium_EhC05
gi|746343808−Novosphingobium_sp__MBES04
gi|1056257784−Novosphingobium_naphthalenivorans
gi|947683466−Sphingopyxis_sp__Root1497
gi|1054929042−Dermacoccus_sp__CCH12−B7
gi|495344081−Novosphingobium_nitrogenifigens_DSM_19370
gi|960875299−Caulobacter_vibrioides
gi|654573269−Caulobacter_sp__URHA0033
gi|947958117−Sphingomonas_sp__Root241
gi|893710148−Sphingobium_yanoikuyae
gi|648417793−Sphingobium_yanoikuyae
gi|657831084−Sphingobium_yanoikuyae
gi|951155182−Stenotrophomonas_humi
gi|938783682−Stenotrophomonas_maltophilia
gi|503300613−Pseudoxanthomonas_suwonensis_11−1
gi|647728745−Brevundimonas_naejangsanensis
gi|1054818639−Sphingomonas_koreensis
0.1
Bacteria
Eukaryota
SAG
NA

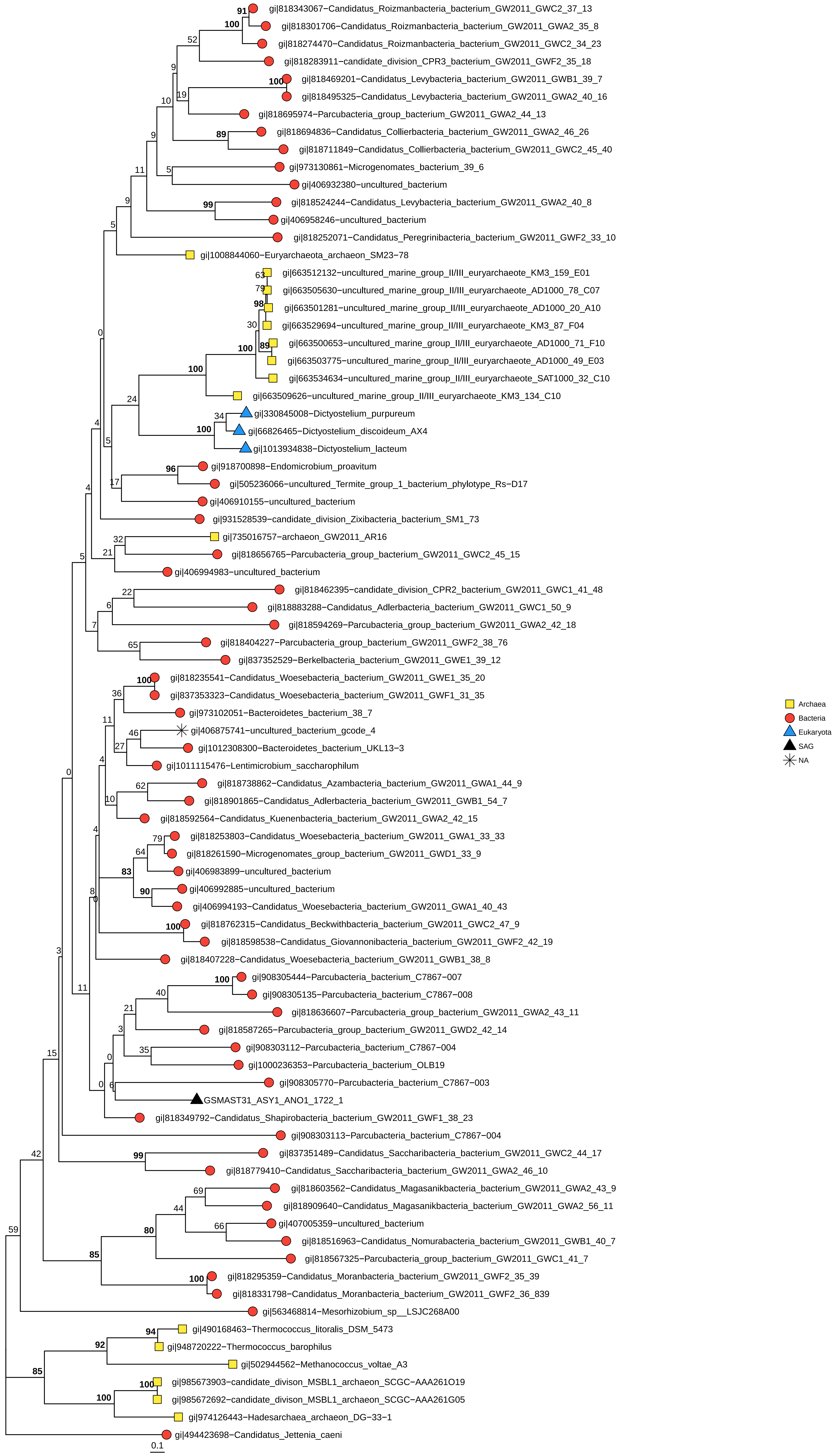
gi|818343067−Candidatus_Roizmanbacteria_bacterium_GW2011_GWC2_37_13
gi|818301706−Candidatus_Roizmanbacteria_bacterium_GW2011_GWA2_35_8
gi|818274470−Candidatus_Roizmanbacteria_bacterium_GW2011_GWC2_34_23
gi|818283911−candidate_division_CPR3_bacterium_GW2011_GWF2_35_18
gi|818469201−Candidatus_Levybacteria_bacterium_GW2011_GWB1_39_7
gi|818495325−Candidatus_Levybacteria_bacterium_GW2011_GWA2_40_16
gi|818695974−Parcubacteria_group_bacterium_GW2011_GWA2_44_13
gi|818694836−Candidatus_Collierbacteria_bacterium_GW2011_GWA2_46_26
gi|818711849−Candidatus_Collierbacteria_bacterium_GW2011_GWC2_45_40
gi|973130861−Microgenomates_bacterium_39_6
gi|406932380−uncultured_bacterium
gi|818524244−Candidatus_Levybacteria_bacterium_GW2011_GWA2_40_8
gi|406958246−uncultured_bacterium
gi|818252071−Candidatus_Peregrinibacteria_bacterium_GW2011_GWF2_33_10
gi|1008844060−Euryarchaeota_archaeon_SM23−78
gi|663512132−uncultured_marine_group_II/III_euryarchaeote_KM3_159_E01
gi|663505630−uncultured_marine_group_II/III_euryarchaeote_AD1000_78_C07
gi|663501281−uncultured_marine_group_II/III_euryarchaeote_AD1000_20_A10
gi|663529694−uncultured_marine_group_II/III_euryarchaeote_KM3_87_F04
gi|663500653−uncultured_marine_group_II/III_euryarchaeote_AD1000_71_F10
gi|663503775−uncultured_marine_group_II/III_euryarchaeote_AD1000_49_E03
gi|663534634−uncultured_marine_group_II/III_euryarchaeote_SAT1000_32_C10
gi|663509626−uncultured_marine_group_II/III_euryarchaeote_KM3_134_C10
gi|330845008−Dictyostelium_purpureum
gi|66826465−Dictyostelium_discoideum_AX4
gi|1013934838−Dictyostelium_lacteum
gi|918700898−Endomicrobium_proavitum
gi|505236066−uncultured_Termite_group_1_bacterium_phylotype_Rs−D17
gi|406910155−uncultured_bacterium
gi|931528539−candidate_division_Zixibacteria_bacterium_SM1_73
gi|735016757−archaeon_GW2011_AR16
gi|818656765−Parcubacteria_group_bacterium_GW2011_GWC2_45_15
gi|406994983−uncultured_bacterium
gi|818462395−candidate_division_CPR2_bacterium_GW2011_GWC1_41_48
gi|818883288−Candidatus_Adlerbacteria_bacterium_GW2011_GWC1_50_9
gi|818594269−Parcubacteria_group_bacterium_GW2011_GWA2_42_18
gi|818404227−Parcubacteria_group_bacterium_GW2011_GWF2_38_76
gi|837352529−Berkelbacteria_bacterium_GW2011_GWE1_39_12
gi|818235541−Candidatus_Woesebacteria_bacterium_GW2011_GWE1_35_20
gi|837353323−Candidatus_Woesebacteria_bacterium_GW2011_GWF1_31_35
gi|973102051−Bacteroidetes_bacterium_38_7
gi|406875741−uncultured_bacterium_gcode_4
gi|1012308300−Bacteroidetes_bacterium_UKL13−3
gi|1011115476−Lentimicrobium_saccharophilum
gi|818738862−Candidatus_Azambacteria_bacterium_GW2011_GWA1_44_9
gi|818901865−Candidatus_Adlerbacteria_bacterium_GW2011_GWB1_54_7
gi|818592564−Candidatus_Kuenenbacteria_bacterium_GW2011_GWA2_42_15
gi|818253803−Candidatus_Woesebacteria_bacterium_GW2011_GWA1_33_33
gi|818261590−Microgenomates_group_bacterium_GW2011_GWD1_33_9
gi|406983899−uncultured_bacterium
gi|406992885−uncultured_bacterium
gi|406994193−Candidatus_Woesebacteria_bacterium_GW2011_GWA1_40_43
gi|818762315−Candidatus_Beckwithbacteria_bacterium_GW2011_GWC2_47_9
gi|818598538−Candidatus_Giovannonibacteria_bacterium_GW2011_GWF2_42_19
gi|818407228−Candidatus_Woesebacteria_bacterium_GW2011_GWB1_38_8
gi|908305444−Parcubacteria_bacterium_C7867−007
gi|908305135−Parcubacteria_bacterium_C7867−008
gi|818636607−Parcubacteria_group_bacterium_GW2011_GWA2_43_11
gi|818587265−Parcubacteria_group_bacterium_GW2011_GWD2_42_14
gi|908303112−Parcubacteria_bacterium_C7867−004
gi|1000236353−Parcubacteria_bacterium_OLB19
gi|908305770−Parcubacteria_bacterium_C7867−003
GSMAST31_ASY1_ANO1_1722_1
gi|818349792−Candidatus_Shapirobacteria_bacterium_GW2011_GWF1_38_23
gi|908303113−Parcubacteria_bacterium_C7867−004
gi|837351489−Candidatus_Saccharibacteria_bacterium_GW2011_GWC2_44_17
gi|818779410−Candidatus_Saccharibacteria_bacterium_GW2011_GWA2_46_10
gi|818603562−Candidatus_Magasanikbacteria_bacterium_GW2011_GWA2_43_9
gi|818909640−Candidatus_Magasanikbacteria_bacterium_GW2011_GWA2_56_11
gi|407005359−uncultured_bacterium
gi|818516963−Candidatus_Nomurabacteria_bacterium_GW2011_GWB1_40_7
gi|818567325−Parcubacteria_group_bacterium_GW2011_GWC1_41_7
gi|818295359−Candidatus_Moranbacteria_bacterium_GW2011_GWF2_35_39
gi|818331798−Candidatus_Moranbacteria_bacterium_GW2011_GWF2_36_839
gi|563468814−Mesorhizobium_sp__LSJC268A00
gi|490168463−Thermococcus_litoralis_DSM_5473
gi|948720222−Thermococcus_barophilus
gi|502944562−Methanococcus_voltae_A3
gi|985673903−candidate_divison_MSBL1_archaeon_SCGC−AAA261O19
gi|985672692−candidate_divison_MSBL1_archaeon_SCGC−AAA261G05
gi|974126443−Hadesarchaea_archaeon_DG−33−1
gi|494423698−Candidatus_Jettenia_caeni
0.1
Archaea
Bacteria
Eukaryota
SAG
NA

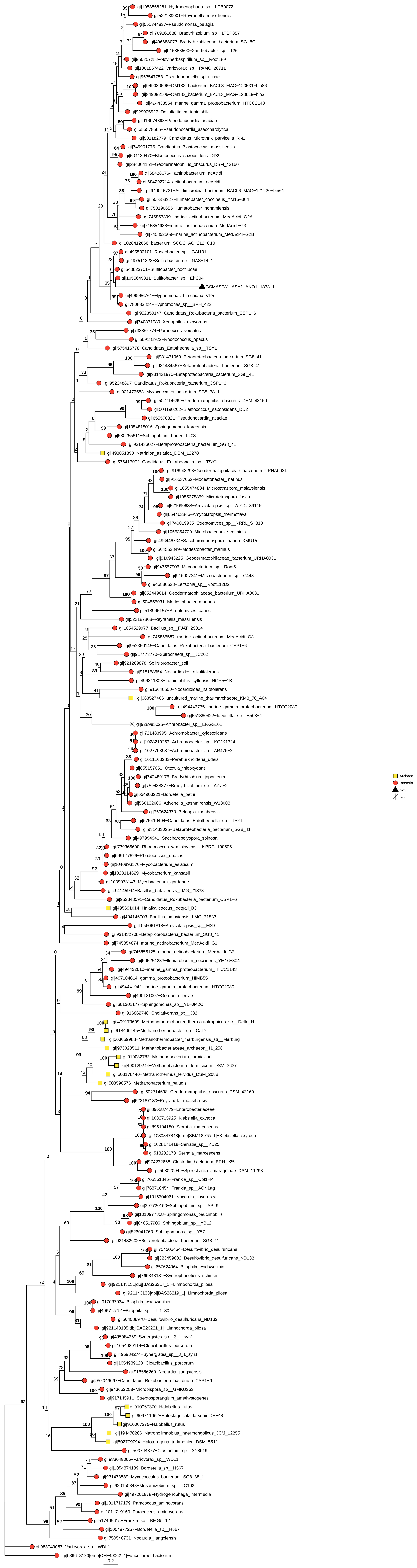

gi|1053868261~Hydrogenophaga_sp__LPB0072
gi|522189001~Reyranella_massiliensis
gi|551344837~Pseudomonas_pelagia
gi|769261688~Bradyrhizobium_sp__LTSP857
gi|496888073~Bradyrhizobiaceae_bacterium_SG-6C
gi|916853500~Xanthobacter_sp__126
gi|950257252~Noviherbaspirillum_sp__Root189
gi|1001857422~Variovorax_sp__PAMC_28711
gi|953547753~Pseudohongiella_spirulinae
gi|949080696~OM182_bacterium_BACL3_MAG-120531-bin86
gi|949092106~OM182_bacterium_BACL3_MAG-120619-bin3
gi|494433554~marine_gamma_proteobacterium_HTCC2143
gi|929005527~Desulfatitalea_tepidiphila
gi|916974893~Pseudonocardia_acaciae
gi|655578565~Pseudonocardia_asaccharolytica
gi|501182779~Candidatus_Microthrix_parvicella_RN1
gi|749991776~Candidatus_Blastococcus_massiliensis
gi|504189470~Blastococcus_saxobsidens_DD2
gi|284064151~Geodermatophilus_obscurus_DSM_43160
gi|684286764~actinobacterium_acAcidi
gi|684292714~actinobacterium_acAcidi
gi|949046721~Acidimicrobiia_bacterium_BACL6_MAG-121220-bin61
gi|505253927~Ilumatobacter_coccineus_YM16-304
gi|750190655~Ilumatobacter_nonamiensis
gi|745853899~marine_actinobacterium_MedAcidi-G2A
gi|745854938~marine_actinobacterium_MedAcidi-G3
gi|745852569~marine_actinobacterium_MedAcidi-G2B
gi|1028412666~bacterium_SCGC_AG-212-C10
gi|495503101~Roseobacter_sp__GAI101
gi|497511823~Sulfitobacter_sp__NAS-14_1
gi|640623701~Sulfitobacter_noctilucae
gi|1055649311~Sulfitobacter_sp__EhC04
GSMAST31_ASY1_ANO1_1878_1
gi|499966761~Hyphomonas_hirschiana_VP5
gi|780833824~Hyphomonas_sp__BRH_c22
gi|952350147~Candidatus_Rokubacteria_bacterium_CSP1-6
gi|740371989~Xenophilus_azovorans
gi|738864774~Paracoccus_versutus
gi|669182922~Rhodococcus_opacus
gi|575416778~Candidatus_Entotheonella_sp__TSY1
gi|931431969~Betaproteobacteria_bacterium_SG8_41
gi|931434567~Betaproteobacteria_bacterium_SG8_41
gi|931431970~Betaproteobacteria_bacterium_SG8_41
gi|952348897~Candidatus_Rokubacteria_bacterium_CSP1-6
gi|931473583~Myxococcales_bacterium_SG8_38_1
gi|502714699~Geodermatophilus_obscurus_DSM_43160
gi|504190202~Blastococcus_saxobsidens_DD2
gi|655570321~Pseudonocardia_acaciae
gi|1054818016~Sphingomonas_koreensis
gi|530255611~Sphingobium_baderi_LL03
gi|931433027~Betaproteobacteria_bacterium_SG8_41
gi|493051893~Natrialba_asiatica_DSM_12278
gi|575417072~Candidatus_Entotheonella_sp__TSY1
gi|916943293~Geodermatophilaceae_bacterium_URHA0031
gi|916537062~Modestobacter_marinus
gi|1055474834~Microtetraspora_malaysiensis
gi|1055278859~Microtetraspora_fusca
gi|521090638~Amycolatopsis_sp__ATCC_39116
gi|654463846~Amycolatopsis_thermoflava
gi|740019935~Streptomyces_sp__NRRL_S-813
gi|1055364729~Microbacterium_sediminis
gi|496446734~Saccharomonospora_marina_XMU15
gi|504553849~Modestobacter_marinus
gi|916943225~Geodermatophilaceae_bacterium_URHA0031
gi|947557906~Microbacterium_sp__Root61
gi|916907341~Microbacterium_sp__C448
gi|946886628~Leifsonia_sp__Root112D2
gi|652449614~Geodermatophilaceae_bacterium_URHA0031
gi|504555031~Modestobacter_marinus
gi|518966157~Streptomyces_canus
gi|522187808~Reyranella_massiliensis
gi|1054529977~Bacillus_sp__FJAT-29814
gi|745855587~marine_actinobacterium_MedAcidi-G3
gi|952350145~Candidatus_Rokubacteria_bacterium_CSP1-6
gi|917473770~Spirochaeta_sp__JC202
gi|921289878~Solirubrobacter_soli
gi|918158654~Nocardioides_alkalitolerans
gi|496311808~Luminiphilus_syltensis_NOR5-1B
gi|916640500~Nocardioides_halotolerans
gi|663527406~uncultured_marine_thaumarchaeote_KM3_78_A04
gi|494442775~marine_gamma_proteobacterium_HTCC2080
gi|551360422~Ideonella_sp__B508-1
gi|928985025~Arthrobacter_sp__ERGS101
gi|721483995~Achromobacter_xylosoxidans
gi|1028219263~Achromobacter_sp__KCJK1724
gi|1027703987~Achromobacter_sp__AR476-2
gi|1011163282~Paraburkholderia_udeis
gi|655157651~Ottowia_thiooxydans
gi|742489176~Bradyrhizobium_japonicum
gi|759438377~Bradyrhizobium_sp__Al1a-2
gi|654903221~Bordetella_petrii
gi|566132606~Advenella_kashmirensis_W13003
gi|759624373~Belnapia_moabensis
gi|575410404~Candidatus_Entotheonella_sp__TSY1
gi|931433025~Betaproteobacteria_bacterium_SG8_41
gi|497994941~Saccharopolyspora_spinosa
gi|739366690~Rhodococcus_wratislaviensis_NBRC_100605
gi|669177629~Rhodococcus_opacus
gi|1040893576~Mycobacterium_asiaticum
gi|1023114629~Mycobacterium_kansasii
gi|1039978143~Mycobacterium_gordonae
gi|494145994~Bacillus_bataviensis_LMG_21833
gi|952343591~Candidatus_Rokubacteria_bacterium_CSP1-6
gi|495691014~Halalkalicoccus_jeotgali_B3
gi|494146003~Bacillus_bataviensis_LMG_21833
gi|1056061818~Amycolatopsis_sp__M39
gi|931432708~Betaproteobacteria_bacterium_SG8_41
gi|745854874~marine_actinobacterium_MedAcidi-G1
gi|745856125~marine_actinobacterium_MedAcidi-G3
gi|505254283~Ilumatobacter_coccineus_YM16-304
gi|494432610~marine_gamma_proteobacterium_HTCC2143
gi|497104614~gamma_proteobacterium_HIMB55
gi|494441942~marine_gamma_proteobacterium_HTCC2080
gi|490121007~Gordonia_terrae
gi|661302177~Sphingomonas_sp__YL-JM2C
gi|916862748~Chelativorans_sp__J32
gi|499179609~Methanothermobacter_thermautotrophicus_str__Delta_H
gi|918406145~Methanothermobacter_sp__CaT2
gi|503059988~Methanothermobacter_marburgensis_str__Marburg
gi|973020511~Methanobacteriaceae_archaeon_41_258
gi|919082783~Methanobacterium_formicicum
gi|490129244~Methanobacterium_formicicum_DSM_3637
gi|503178440~Methanothermus_fervidus_DSM_2088
gi|503590576~Methanobacterium_paludis
gi|502714698~Geodermatophilus_obscurus_DSM_43160
gi|522187130~Reyranella_massiliensis
gi|896287479~Enterobacteriaceae
gi|1032715925~Klebsiella_oxytoca
gi|896194180~Serratia_marcescens
gi|1030347848|emb|SBM18975_1|~Klebsiella_oxytoca
gi|1028171418~Serratia_sp__YD25
gi|518282173~Serratia_marcescens
gi|974232658~Clostridia_bacterium_BRH_c25
gi|503020949~Spirochaeta_smaragdinae_DSM_11293
gi|765351846~Frankia_sp__CpI1-P
gi|768716454~Frankia_sp__ACN1ag
gi|1016304061~Nocardia_flavorosea
gi|397720150~Sphingobium_sp__AP49
gi|1010977808~Sphingomonas_paucimobilis
gi|646517906~Sphingobium_sp__YBL2
gi|826041763~Sphingomonas_sp__Y57
gi|931432602~Betaproteobacteria_bacterium_SG8_41
gi|754505454~Desulfovibrio_desulfuricans
gi|323459682~Desulfovibrio_desulfuricans_ND132
gi|657624064~Bilophila_wadsworthia
gi|765348137~Syntrophaceticus_schinkii
gi|921143131|dbj|BAS26217_1|~Limnochorda_pilosa
gi|921143133|dbj|BAS26219_1|~Limnochorda_pilosa
gi|917037034~Bilophila_wadsworthia
gi|496775791~Bilophila_sp__4_1_30
gi|504088978~Desulfovibrio_desulfuricans_ND132
gi|921143135|dbj|BAS26221_1|~Limnochorda_pilosa
gi|495984269~Synergistes_sp__3_1_syn1
gi|1054989114~Cloacibacillus_porcorum
gi|495984274~Synergistes_sp__3_1_syn1
gi|1054989128~Cloacibacillus_porcorum
gi|916586260~Nocardia_jiangxiensis
gi|952346067~Candidatus_Rokubacteria_bacterium_CSP1-6
gi|943652253~Microbispora_sp__GMKU363
gi|917145911~Streptosporangium_amethystogenes
gi|910067370~Halobellus_rufus
gi|909711662~Halostagnicola_larsenii_XH-48
gi|910067375~Halobellus_rufus
gi|494470286~Natronolimnobius_innermongolicus_JCM_12255
gi|502709794~Haloterrigena_turkmenica_DSM_5511
gi|503744377~Clostridium_sp__SY8519
gi|983049066~Variovorax_sp__WDL1
gi|1054874189~Bordetella_sp__H567
gi|931473589~Myxococcales_bacterium_SG8_38_1
gi|920150848~Mesorhizobium_sp__LC103
gi|497201878~Hydrogenophaga_intermedia
gi|1011719179~Paracoccus_aminovorans
gi|1011719169~Paracoccus_aminovorans
gi|517465615~Frankia_sp__BMG5_12
gi|1054877257~Bordetella_sp__H567
gi|750548731~Nocardia_jiangxiensis
gi|983049057~Variovorax_sp__WDL1
gi|689678120|emb|CEF49062_1|~uncultured_bacterium
0.2
Archaea
Bacteria
SAG
NA

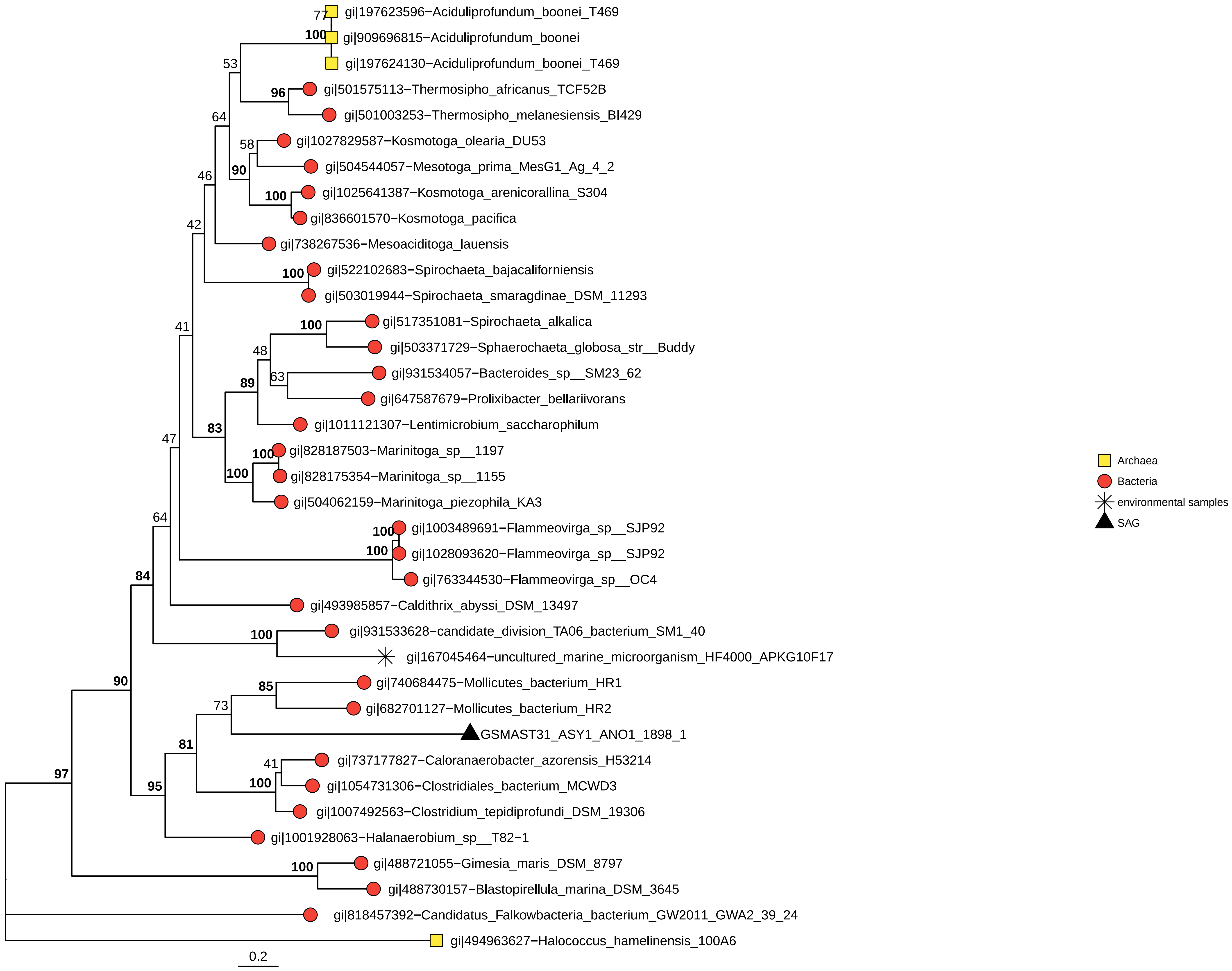

gi|197623596−Aciduliprofundum_boonei_T469
gi|909696815−Aciduliprofundum_boonei
gi|197624130−Aciduliprofundum_boonei_T469
gi|501575113−Thermosipho_africanus_TCF52B
gi|501003253−Thermosipho_melanesiensis_BI429
gi|1027829587−Kosmotoga_olearia_DU53
gi|504544057−Mesotoga_prima_MesG1_Ag_4_2
gi|1025641387−Kosmotoga_arenicorallina_S304
gi|836601570−Kosmotoga_pacifica
gi|738267536−Mesoaciditoga_lauensis
gi|522102683−Spirochaeta_bajacaliforniensis
gi|503019944−Spirochaeta_smaragdinae_DSM_11293
gi|517351081−Spirochaeta_alkalica
gi|503371729−Sphaerochaeta_globosa_str__Buddy
gi|931534057−Bacteroides_sp__SM23_62
gi|647587679−Prolixibacter_bellariivorans
gi|1011121307−Lentimicrobium_saccharophilum
gi|828187503−Marinitoga_sp__1197
gi|828175354−Marinitoga_sp__1155
gi|504062159−Marinitoga_piezophila_KA3
gi|1003489691−Flammeovirga_sp__SJP92
gi|1028093620−Flammeovirga_sp__SJP92
gi|763344530−Flammeovirga_sp__OC4
gi|493985857−Caldithrix_abyssi_DSM_13497
gi|931533628−candidate_division_TA06_bacterium_SM1_40
gi|167045464−uncultured_marine_microorganism_HF4000_APKG10F17
gi|740684475−Mollicutes_bacterium_HR1
gi|682701127−Mollicutes_bacterium_HR2
GSMAST31_ASY1_ANO1_1898_1
gi|737177827−Caloranaerobacter_azorensis_H53214
gi|1054731306−Clostridiales_bacterium_MCWD3
gi|1007492563−Clostridium_tepidiprofundi_DSM_19306
gi|1001928063−Halanaerobium_sp__T82−1
gi|488721055−Gimesia_maris_DSM_8797
gi|488730157−Blastopirellula_marina_DSM_3645
gi|818457392−Candidatus_Falkowbacteria_bacterium_GW2011_GWA2_39_24
gi|494963627−Halococcus_hamelinensis_100A6
77
100
53
96
64
58
90
100
46
42
100
41
100
48
63
89
83
100
100
47
100
100
64
84
100
85
73
81
41
100
90
95
97
100
0.2
Archaea
Bacteria
environmental samples
SAG

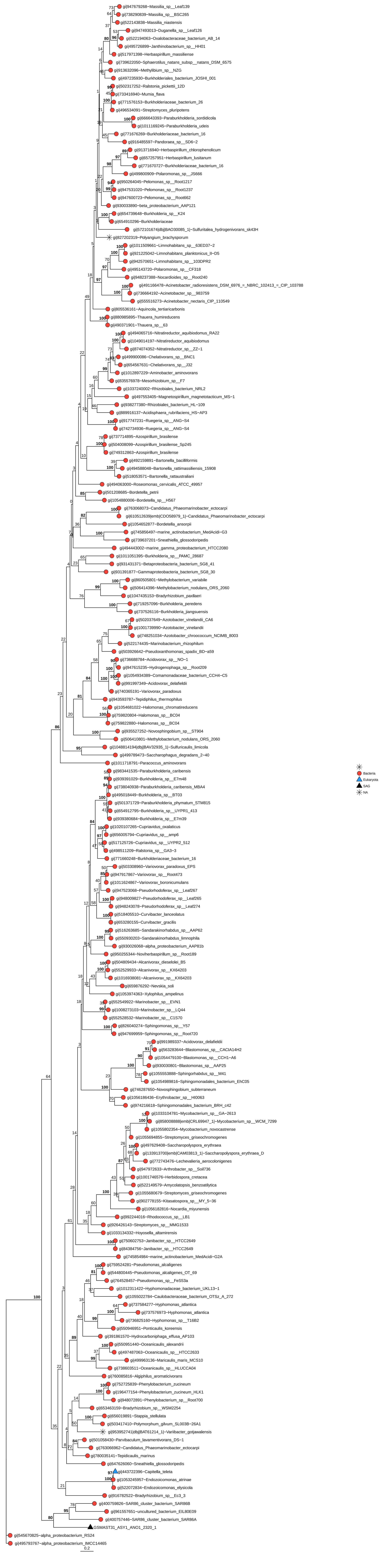

gi|947679268~Massilia_sp__Leaf139
gi|738290839~Massilia_sp__BSC265
gi|522143838~Massilia_niastensis
gi|947493013~Duganella_sp__Leaf126
gi|522194063~Oxalobacteraceae_bacterium_AB_14
gi|495726899~Janthinobacterium_sp__HH01
gi|517971398~Herbaspirillum_massiliense
gi|739622050~Sphaerotilus_natans_subsp__natans_DSM_6575
gi|913632096~Methylibium_sp__NZG
gi|497235930~Burkholderiales_bacterium_JOSHI_001
gi|502317252~Ralstonia_pickettii_12D
gi|733416940~Mumia_flava
gi|771576153~Burkholderiaceae_bacterium_26
gi|496534091~Streptomyces_pluripotens
gi|666643393~Paraburkholderia_sordidicola
gi|1011169245~Paraburkholderia_udeis
gi|771676269~Burkholderiaceae_bacterium_16
gi|916485597~Pandoraea_sp__SD6-2
gi|913716940~Herbaspirillum_chlorophenolicum
gi|657257951~Herbaspirillum_lusitanum
gi|771670727~Burkholderiaceae_bacterium_16
gi|499800909~Polaromonas_sp__JS666
gi|950264045~Pelomonas_sp__Root1217
gi|947531020~Pelomonas_sp__Root1237
gi|947600723~Pelomonas_sp__Root662
gi|930033890~beta_proteobacterium_AAP121
gi|654733648~Burkholderia_sp__K24
gi|654910296~Burkholderiaceae
gi|572101674|dbj|BAO30085_1|~Sulfuritalea_hydrogenivorans_sk43H
gi|827202319~Polyangium_brachysporum
gi|1011509661~Limnohabitans_sp__63ED37-2
gi|921225042~Limnohabitans_planktonicus_II-D5
gi|942570651~Limnohabitans_sp__103DPR2
gi|495143720~Polaromonas_sp__CF318
gi|948237388~Nocardioides_sp__Root240
gi|491166478~Acinetobacter_radioresistens_DSM_6976_=_NBRC_102413_=_CIP_103788
gi|736664192~Acinetobacter_sp__983759
gi|555516273~Acinetobacter_nectaris_CIP_110549
gi|805536161~Aquincola_tertiaricarbonis
gi|880985895~Thauera_humireducens
gi|490371901~Thauera_sp__63
gi|494065716~Nitratireductor_aquibiodomus_RA22
gi|1049014197~Nitratireductor_aquibiodomus
gi|874074352~Nitratireductor_sp__ZZ-1
gi|499900086~Chelativorans_sp__BNC1
gi|654567631~Chelativorans_sp__J32
gi|1012897229~Aminobacter_aminovorans
gi|835576978~Mesorhizobium_sp__F7
gi|1037240002~Rhizobiales_bacterium_NRL2
gi|497553405~Magnetospirillum_magnetotacticum_MS-1
gi|938277380~Rhizobiales_bacterium_HL-109
gi|889916137~Acidisphaera_rubrifaciens_HS-AP3
gi|917747231~Ruegeria_sp__ANG-S4
gi|742734936~Ruegeria_sp__ANG-S4
gi|737714895~Azospirillum_brasilense
gi|504008099~Azospirillum_brasilense_Sp245
gi|749312863~Azospirillum_brasilense
gi|492159891~Bartonella_bacilliformis
gi|494588048~Bartonella_rattimassiliensis_15908
gi|518053571~Bartonella_rattaustraliani
gi|494063000~Roseomonas_cervicalis_ATCC_49957
gi|501208685~Bordetella_petrii
gi|1054880006~Bordetella_sp__H567
gi|763068073~Candidatus_Phaeomarinobacter_ectocarpi
gi|610512639|emb|CDO58979_1|~Candidatus_Phaeomarinobacter_ectocarpi
gi|1054652877~Bordetella_ansorpii
gi|745856497~marine_actinobacterium_MedAcidi-G3
gi|739637201~Sneathiella_glossodoripedis
gi|494443002~marine_gamma_proteobacterium_HTCC2080
gi|1011051395~Burkholderia_sp__PAMC_28687
gi|931431371~Betaproteobacteria_bacterium_SG8_41
gi|931391877~Gammaproteobacteria_bacterium_SG8_30
gi|860505801~Methylobacterium_variabile
gi|506414396~Methylobacterium_nodulans_ORS_2060
gi|1047435153~Bradyrhizobium_paxllaeri
gi|719257096~Burkholderia_peredens
gi|737526116~Burkholderia_jiangsuensis
gi|502037649~Azotobacter_vinelandii_CA6
gi|1001739990~Azotobacter_vinelandii
gi|748251034~Azotobacter_chroococcum_NCIMB_8003
gi|522174435~Marinobacterium_rhizophilum
gi|503926642~Pseudoxanthomonas_spadix_BD-a59
gi|736688784~Acidovorax_sp__NO-1
gi|947615235~Hydrogenophaga_sp__Root209
gi|1054934389~Comamonadaceae_bacterium_CCH4-C5
gi|991997349~Acidovorax_delafieldii
gi|740365191~Variovorax_paradoxus
gi|943593787~Tepidiphilus_thermophilus
gi|1054681022~Halomonas_chromatireducens
gi|759820804~Halomonas_sp__BC04
gi|759822880~Halomonas_sp__BC04
gi|935527252~Novosphingobium_sp__ST904
gi|506410801~Methylobacterium_nodulans_ORS_2060
gi|1048814194|dbj|BAV32935_1|~Sulfuricaulis_limicola
gi|499789473~Saccharophagus_degradans_2-40
gi|1011718791~Paracoccus_aminovorans
gi|983441535~Paraburkholderia_caribensis
gi|939391029~Burkholderia_sp__E7m48
gi|738040938~Paraburkholderia_caribensis_MBA4
gi|495018449~Burkholderia_sp__BT03
gi|501371729~Paraburkholderia_phymatum_STM815
gi|654912795~Burkholderia_sp__UYPR1_413
gi|939380684~Burkholderia_sp__E7m39
gi|1020107265~Cupriavidus_oxalaticus
gi|656005794~Cupriavidus_sp__amp6
gi|517125726~Cupriavidus_sp__UYPR2_512
gi|498511209~Ralstonia_sp__GA3-3
gi|771660248~Burkholderiaceae_bacterium_16
gi|503308960~Variovorax_paradoxus_EPS
gi|947917867~Variovorax_sp__Root473
gi|1011624867~Variovorax_boronicumulans
gi|947523068~Pseudorhodoferax_sp__Leaf267
gi|948009827~Pseudorhodoferax_sp__Leaf265
gi|948243078~Pseudorhodoferax_sp__Leaf274
gi|518405510~Curvibacter_lanceolatus
gi|653280155~Curvibacter_gracilis
gi|516263685~Sandarakinorhabdus_sp__AAP62
gi|550930203~Sandarakinorhabdus_limnophila
gi|930026068~alpha_proteobacterium_AAP81b
gi|950255344~Noviherbaspirillum_sp__Root189
gi|504809434~Alcanivorax_dieselolei_B5
gi|552529933~Alcanivorax_sp__KX64203
gi|1016938081~Alcanivorax_sp__KX64203
gi|659876292~Nevskia_soli
gi|1053974363~Xylophilus_ampelinus
gi|552549922~Marinobacter_sp__EVN1
gi|1008273103~Marinobacter_sp__LQ44
gi|552528532~Marinobacter_sp__C1S70
gi|826040274~Sphingomonas_sp__Y57
gi|947699959~Sphingomonas_sp__Root720
gi|991989337~Acidovorax_delafieldii
gi|563283644~Blastomonas_sp__CACIA14H2
gi|1054479100~Blastomonas_sp__CCH1-A6
gi|930030801~Blastomonas_sp__AAP25
gi|1055553888~Sphingorhabdus_sp__M41
gi|1054989816~Sphingomonadales_bacterium_EhC05
gi|746287650~Novosphingobium_subterraneum
gi|1056186436~Erythrobacter_sp__HI0063
gi|974216618~Sphingomonadales_bacterium_BRH_c42
gi|1033104781~Mycobacterium_sp__GA-2613
gi|858008888|emb|CRL69947_1|~Mycobacterium_sp__WCM_7299
gi|1055802354~Mycobacterium_novocastrense
gi|1055694855~Streptomyces_griseochromogenes
gi|497629408~Saccharopolyspora_erythraea
gi|133913700|emb|CAM03813_1|~Saccharopolyspora_erythraea_D
gi|772743476~Lechevalieria_aerocolonigenes
gi|947972633~Arthrobacter_sp__Soil736
gi|1001746576~Herbidospora_cretacea
gi|522149579~Amycolatopsis_benzoatilytica
gi|1055680679~Streptomyces_griseochromogenes
gi|902778155~Kitasatospora_sp__MY_5-36
gi|1056182816~Nocardia_miyunensis
gi|992244016~Rhodococcus_sp__LB1
gi|926426143~Streptomyces_sp__MMG1533
gi|1033134332~Hoyosella_altamirensis
gi|750602753~Janibacter_sp__HTCC2649
gi|84384756~Janibacter_sp__HTCC2649
gi|745854984~marine_actinobacterium_MedAcidi-G2A
gi|759524281~Pseudomonas_alcaligenes
gi|544800445~Pseudomonas_alcaligenes_OT_69
gi|764528457~Pseudomonas_sp__FeS53a
gi|1012311422~Hyphomonadaceae_bacterium_UKL13-1
gi|1055022784~Caulobacteraceae_bacterium_OTSz_A_272
gi|737584277~Hyphomonas_atlantica
gi|737576973~Hyphomonas_atlantica
gi|736825160~Hyphomonas_sp__T16B2
gi|550946951~Ponticaulis_koreensis
gi|391861570~Hydrocarboniphaga_effusa_AP103
gi|550951440~Oceanicaulis_alexandrii
gi|497487063~Oceanicaulis_sp__HTCC2633
gi|499963136~Maricaulis_maris_MCS10
gi|738603511~Oceanicaulis_sp__HLUCCA04
gi|760085816~Algiphilus_aromaticivorans
gi|752725839~Phenylobacterium_zucineum
gi|196477154~Phenylobacterium_zucineum_HLK1
gi|948072891~Phenylobacterium_sp__Root700
gi|653463159~Bradyrhizobium_sp__WSM2254
gi|656019891~Stappia_stellulata
gi|503417410~Polymorphum_gilvum_SL003B-26A1
gi|953952741|dbj|BAT61214_1|~Variibacter_gotjawalensis
gi|501058430~Parvibaculum_lavamentivorans_DS-1
gi|763066962~Candidatus_Phaeomarinobacter_ectocarpi
gi|780035141~Tepidicaulis_marinus
gi|647626060~Sneathiella_glossodoripedis
gi|443722396~Capitella_teleta
gi|1053245957~Endozoicomonas_atrinae
gi|522072834~Endozoicomonas_elysicola
gi|916782522~Bradyrhizobium_sp__Ec3_3
gi|400759826~SAR86_cluster_bacterium_SAR86B
gi|961557651~uncultured_bacterium_EIL80E09
gi|400757446~SAR86_cluster_bacterium_SAR86A
GSMAST31_ASY1_ANO1_2320_1
gi|546570825~alpha_proteobacterium_RS24
gi|495793767~alpha_proteobacterium_IMCC14465
0.2
Bacteria
Eukaryota
SAG
NA

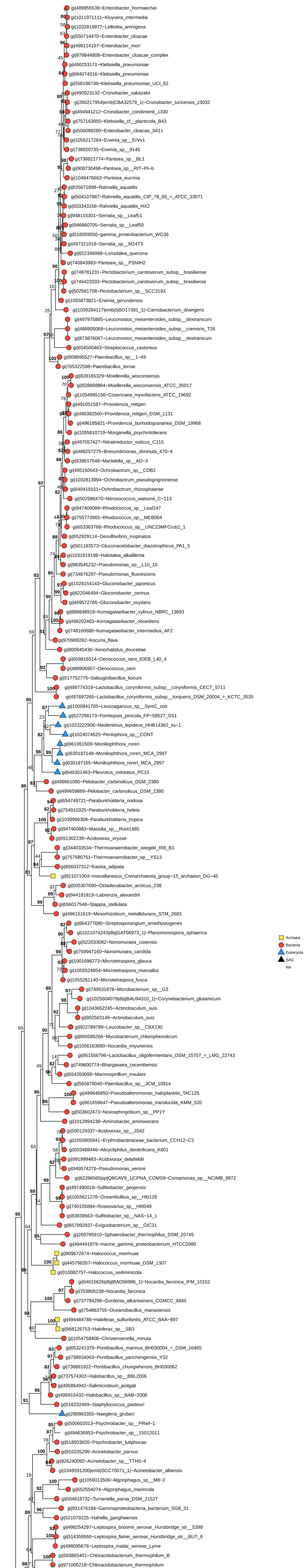

Archaea
Bacteria
Eukaryota
SAG
NA
0.1

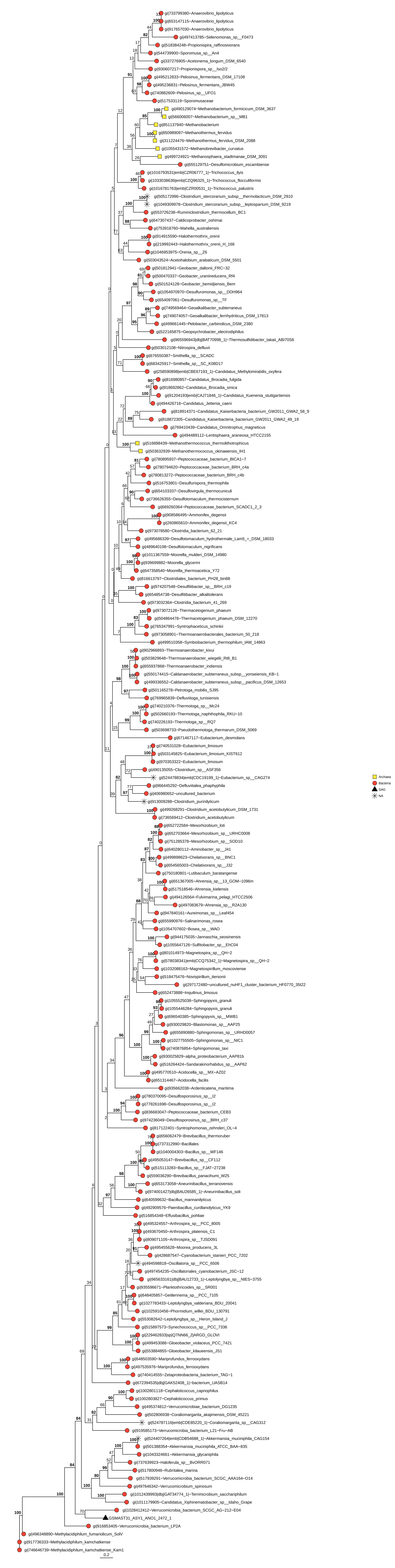

gi|733799380~Anaerovibrio_lipolyticus
gi|653147115~Anaerovibrio_lipolyticus
gi|917657030~Anaerovibrio_lipolyticus
gi|497413785~Selenomonas_sp__F0473
gi|518384248~Propionispira_raffinosivorans
gi|544739900~Sporomusa_sp__An4
gi|337276905~Acetonema_longum_DSM_6540
gi|930607217~Propionispora_sp__Iso2/2
gi|495212833~Pelosinus_fermentans_DSM_17108
gi|495236831~Pelosinus_fermentans_JBW45
gi|740882609~Pelosinus_sp__UFO1
gi|517533119~Sporomusaceae
gi|490129074~Methanobacterium_formicicum_DSM_3637
gi|566006007~Methanobacterium_sp__MB1
gi|851137940~Methanobacterium
gi|850989097~Methanothermus_fervidus
gi|311224476~Methanothermus_fervidus_DSM_2088
gi|1055431572~Methanobrevibacter_curvatus
gi|499724921~Methanosphaera_stadtmanae_DSM_3091
gi|655129751~Desulfomicrobium_escambiense
gi|1016793531|emb|CZR06777_1|~Trichococcus_ilyis
gi|1033083636|emb|CZQ96325_1|~Trichococcus_flocculiformis
gi|1016781763|emb|CZR00531_1|~Trichococcus_palustris
gi|505172996~Clostridium_stercorarium_subsp__thermolacticum_DSM_2910
gi|1049309978~Clostridium_stercorarium_subsp__leptospartum_DSM_9219
gi|553726238~Ruminiclostridium_thermocellum_BC1
gi|647307437~Caldicoprobacter_oshimai
gi|753918760~Mahella_australiensis
gi|914915590~Halothermothrix_orenii
gi|219992443~Halothermothrix_orenii_H_168
gi|1046953975~Orenia_sp__Z6
gi|503043524~Acetohalobium_arabaticum_DSM_5501
gi|501812941~Geobacter_daltonii_FRC-32
gi|500470337~Geobacter_uraniireducens_Rf4
gi|501524128~Geobacter_bemidjiensis_Bem
gi|1054970970~Desulfuromonas_sp__DDH964
gi|654097061~Desulfuromonas_sp__TF
gi|749569464~Geoalkalibacter_subterraneus
gi|749074057~Geoalkalibacter_ferrihydriticus_DSM_17813
gi|499661445~Pelobacter_carbinolicus_DSM_2380
gi|522165875~Geopsychrobacter_electrodiphilus
gi|965596943|dbj|BAT70998_1|~Thermosulfidibacter_takaii_ABI70S6
gi|503012108~Nitrospira_defluvii
gi|676550387~Smithella_sp__SCADC
gi|683425917~Smithella_sp__SC_K08D17
gi|258590898|emb|CBE67193_1|~Candidatus_Methylomirabilis_oxyfera
gi|816980857~Candidatus_Brocadia_fulgida
gi|918692862~Candidatus_Brocadia_sinica
gi|91204193|emb|CAJ71846_1|~Candidatus_Kuenenia_stuttgartiensis
gi|494426716~Candidatus_Jettenia_caeni
gi|818914371~Candidatus_Kaiserbacteria_bacterium_GW2011_GWA2_58_9
gi|818872305~Candidatus_Kaiserbacteria_bacterium_GW2011_GWA2_49_19
gi|769410439~Candidatus_Omnitrophus_magneticus
gi|494489112~Lentisphaera_araneosa_HTCC2155
gi|516898439~Methanothermococcus_thermolithotrophicus
gi|503632939~Methanothermococcus_okinawensis_IH1
gi|780895937~Peptococcaceae_bacterium_BICA1-7
gi|780794620~Peptococcaceae_bacterium_BRH_c4a
gi|780813272~Peptococcaceae_bacterium_BRH_c4b
gi|516753801~Desulfurispora_thermophila
gi|654103337~Desulfovirgula_thermocuniculi
gi|736626355~Desulfotomaculum_thermocisternum
gi|669260364~Peptococcaceae_bacterium_SCADC1_2_3
gi|908586495~Ammonifex_degensii
gi|260865810~Ammonifex_degensii_KC4
gi|973078580~Clostridia_bacterium_62_21
gi|495686339~Desulfotomaculum_hydrothermale_Lam5_=_DSM_18033
gi|489640198~Desulfotomaculum_nigrificans
gi|1011367559~Moorella_mulderi_DSM_14980
gi|939699882~Moorella_glycerini
gi|647358540~Moorella_thermoacetica_Y72
gi|816613797~Clostridiales_bacterium_PH28_bin88
gi|974207548~Desulfitibacter_sp__BRH_c19
gi|654854738~Desulfitibacter_alkalitolerans
gi|973032364~Clostridia_bacterium_41_269
gi|973072126~Thermacetogenium_phaeum
gi|504864478~Thermacetogenium_phaeum_DSM_12270
gi|765347991~Syntrophaceticus_schinkii
gi|973058901~Thermoanaerobacterales_bacterium_50_218
gi|499510358~Symbiobacterium_thermophilum_IAM_14863
gi|902966893~Thermoanaerobacter_kivui
gi|503829648~Thermoanaerobacter_wiegelii_Rt8_B1
gi|655937868~Thermoanaerobacter_indiensis
gi|550174415~Caldanaerobacter_subterraneus_subsp__yonseiensis_KB-1
gi|499336552~Caldanaerobacter_subterraneus_subsp__pacificus_DSM_12653
gi|501165278~Petrotoga_mobilis_SJ95
gi|769965839~Defluviitoga_tunisiensis
gi|740210376~Thermotoga_sp__Mc24
gi|502660193~Thermotoga_naphthophila_RKU-10
gi|740226193~Thermotoga_sp__RQ7
gi|503698733~Pseudothermotoga_thermarum_DSM_5069
gi|671467117~Eubacterium_desmolans
gi|740531028~Eubacterium_limosum
gi|503145825~Eubacterium_limosum_KIST612
gi|970353322~Eubacterium_limosum
gi|490135055~Clostridium_sp__ASF356
gi|524478834|emb|CDC19199_1|~Eubacterium_sp__CAG274
gi|966445292~Defluviitalea_phaphyphila
gi|406980652~uncultured_bacterium
gi|913009288~Clostridium_purinilyticum
gi|499268291~Clostridium_acetobutylicum_DSM_1731
gi|736569412~Clostridium_acetobutylicum
gi|652722584~Mesorhizobium_loti
gi|652703664~Mesorhizobium_sp__URHC0008
gi|751285378~Mesorhizobium_sp__SOD10
gi|640280112~Aminobacter_sp__J41
gi|499898623~Chelativorans_sp__BNC1
gi|654565003~Chelativorans_sp__J32
gi|750180801~Lutibaculum_baratangense
gi|651367005~Ahrensia_sp__13_GOM-1096m
gi|517518546~Ahrensia_kielensis
gi|494126564~Fulvimarina_pelagi_HTCC2506
gi|497083679~Ahrensia_sp__R2A130
gi|947840161~Aureimonas_sp__Leaf454
gi|655990976~Salinarimonas_rosea
gi|1054707602~Bosea_sp__WAO
gi|944175035~Jannaschia_seosinensis
gi|1055647126~Sulfitobacter_sp__EhC04
gi|810014973~Magnetospira_sp__QH-2
gi|578038341|emb|CCQ75342_1|~Magnetospira_sp__QH-2
gi|1032088163~Magnetospirillum_moscoviense
gi|518475476~Novispirillum_itersonii
gi|297172480~uncultured_nuHF1_cluster_bacterium_HF0770_35I22
gi|652473888~Inquilinus_limosus
gi|1055525038~Sphingopyxis_granuli
gi|1055446284~Sphingopyxis_granuli
gi|696540385~Sphingopyxis_sp__MWB1
gi|930029820~Blastomonas_sp__AAP25
gi|655890880~Sphingomonas_sp__URHD0057
gi|1027755505~Sphingomonas_sp__NIC1
gi|740876854~Sphingomonas_taxi
gi|930025829~alpha_proteobacterium_AAP81b
gi|516264424~Sandarakinorhabdus_sp__AAP62
gi|495770510~Acidocella_sp__MX-AZ02
gi|651314467~Acidocella_facilis
gi|935662038~Ardenticatena_maritima
gi|780370095~Desulfosporosinus_sp__I2
gi|778261698~Desulfosporosinus_sp__I2
gi|836683047~Peptococcaceae_bacterium_CEB3
gi|974236049~Desulfosporosinus_sp__BRH_c37
gi|817122401~Syntrophomonas_zehnderi_OL-4
gi|656062479~Brevibacillus_thermoruber
gi|737312990~Bacillales
gi|1040004303~Bacillus_sp__WF146
gi|495053147~Brevibacillus_sp__CF112
gi|515113283~Bacillus_sp__FJAT-27238
gi|559036290~Brevibacillus_panacihumi_W25
gi|653173058~Aneurinibacillus_terranovensis
gi|974001427|dbj|BAU26585_1|~Aneurinibacillus_soli
gi|640599632~Bacillus_mannanilyticus
gi|492909576~Paenibacillus_curdlanolyticus_YK9
gi|516854348~Effusibacillus_pohliae
gi|495324557~Arthrospira_sp__PCC_8005
gi|493670450~Arthrospira_platensis_C1
gi|809071105~Arthrospira_sp__TJSD091
gi|495455628~Moorea_producens_3L
gi|428687547~Cyanobacterium_stanieri_PCC_7202
gi|494598818~Oscillatoria_sp__PCC_6506
gi|497454235~Oscillatoriales_cyanobacterium_JSC-12
gi|965633161|dbj|BAU12733_1|~Leptolyngbya_sp__NIES-3755
gi|935596671~Planktothricoides_sp__SR001
gi|648405857~Geitlerinema_sp__PCC_7105
gi|1027783433~Leptolyngbya_valderiana_BDU_20041
gi|1025910456~Phormidium_willei_BDU_130791
gi|553082642~Leptolyngbya_sp__Heron_Island_J
gi|515897573~Synechococcus_sp__PCC_7336
gi|229462833|sp|Q7NN66_2|ARGD_GLOVI
gi|499453086~Gloeobacter_violaceus_PCC_7421
gi|553884855~Gloeobacter_kilaueensis_JS1
gi|648503590~Mariprofundus_ferrooxydans
gi|497535976~Mariprofundus_ferrooxydans
gi|740414555~Zetaproteobacteria_bacterium_TAG-1
gi|672394535|dbj|GAK52408_1|~bacterium_UASB14
gi|1002801118~Cephaloticoccus_capnophilus
gi|1002803827~Cephaloticoccus_primus
gi|495374812~Verrucomicrobiae_bacterium_DG1235
gi|502806938~Coraliomargarita_akajimensis_DSM_45221
gi|524787116|emb|CDE85220_1|~Coraliomargarita_sp__CAG312
gi|919585173~Verrucomicrobia_bacterium_L21-Fru-AB
gi|524407264|emb|CDB54688_1|~Akkermansia_muciniphila_CAG154
gi|501388354~Akkermansia_muciniphila_ATCC_BAA-835
gi|1043324661~Akkermansia_glycaniphila
gi|737639923~Haloferula_sp__BvORR071
gi|517800946~Rubritalea_marina
gi|517939291~Verrucomicrobia_bacterium_SCGC_AAA164-O14
gi|497646342~Verrucomicrobium_spinosum
gi|1012439993|dbj|GAT34774_1|~Terrimicrobium_sacchariphilum
gi|1011179905~Candidatus_Xiphinematobacter_sp__Idaho_Grape
gi|1028412412~Verrucomicrobia_bacterium_SCGC_AG-212-E04
GSMAST31_ASY1_ANO1_2472_1
gi|916853405~Verrucomicrobia_bacterium_LP2A
gi|496348890~Methylacidiphilum_fumariolicum_SolV
gi|917736333~Methylacidiphilum_kamchatkense
gi|746646739~Methylacidiphilum_kamchatkense_Kam1
0.2
Archaea
Bacteria
SAG
NA

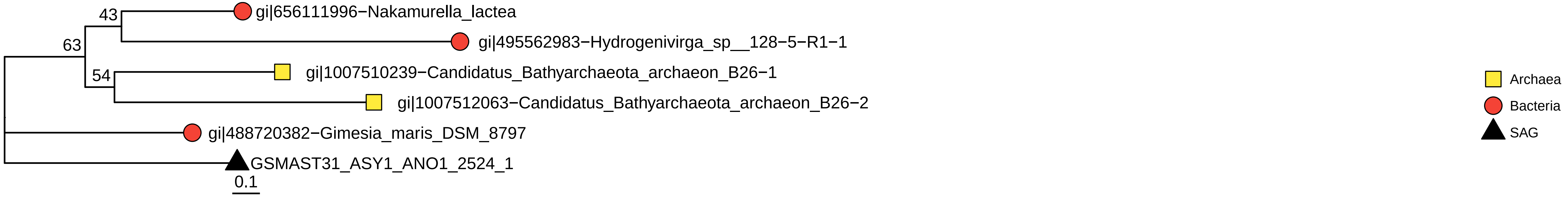
43
63
54
gi|656111996−Nakamurella_lactea
gi|495562983−Hydrogenivirga_sp__128−5−R1−1
gi|1007510239−Candidatus_Bathyarchaeota_archaeon_B26−1
gi|1007512063−Candidatus_Bathyarchaeota_archaeon_B26−2
gi|488720382−Gimesia_maris_DSM_8797
GSMAST31_ASY1_ANO1_2524_1
0.1
Archaea
Bacteria
SAG

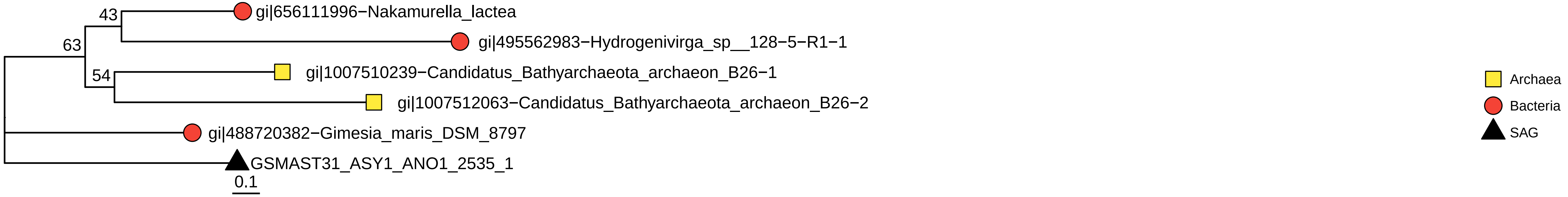
43
63
54
gi|656111996−Nakamurella_lactea
gi|495562983−Hydrogenivirga_sp__128−5−R1−1
gi|1007510239−Candidatus_Bathyarchaeota_archaeon_B26−1
gi|1007512063−Candidatus_Bathyarchaeota_archaeon_B26−2
gi|488720382−Gimesia_maris_DSM_8797
GSMAST31_ASY1_ANO1_2535_1
0.1
Archaea
Bacteria
SAG

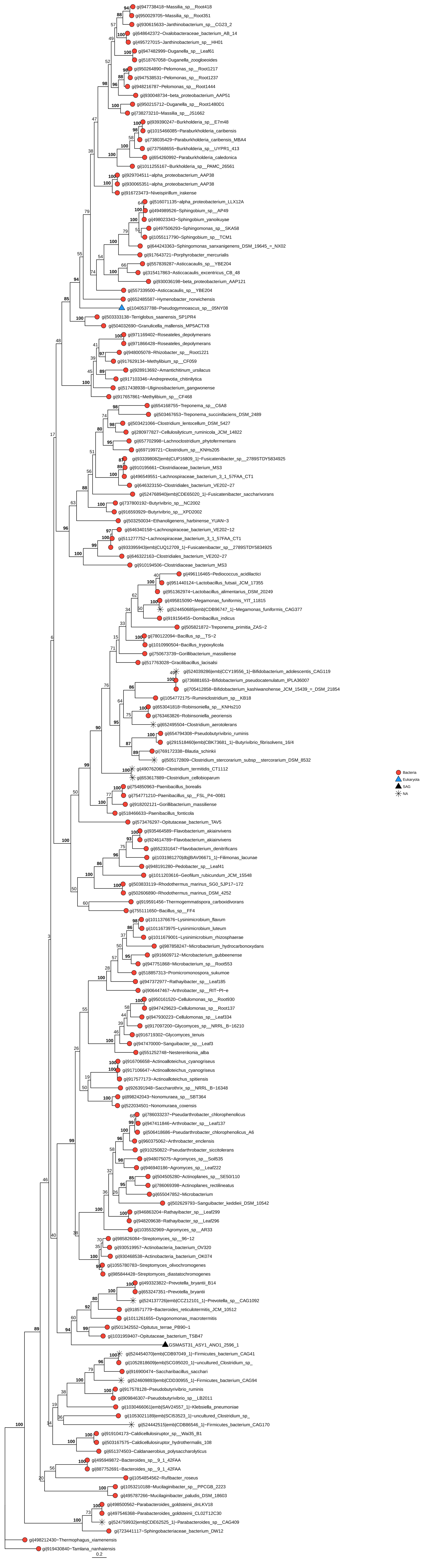
gi|947738418~Massilia_sp__Root418
gi|950029705~Massilia_sp__Root351
gi|930615633~Janthinobacterium_sp__CG23_2
gi|648642372~Oxalobacteraceae_bacterium_AB_14
gi|495727015~Janthinobacterium_sp__HH01
gi|947482999~Duganella_sp__Leaf61
gi|518767058~Duganella_zoogloeoides
gi|950264890~Pelomonas_sp__Root1217
gi|947538531~Pelomonas_sp__Root1237
gi|948216787~Pelomonas_sp__Root1444
gi|930048734~beta_proteobacterium_AAP51
gi|950215712~Duganella_sp__Root1480D1
gi|738273210~Massilia_sp__JS1662
gi|939390247~Burkholderia_sp__E7m48
gi|1015466085~Paraburkholderia_caribensis
gi|738035429~Paraburkholderia_caribensis_MBA4
gi|737568655~Burkholderia_sp__UYPR1_413
gi|654260992~Paraburkholderia_caledonica
gi|1011255167~Burkholderia_sp__PAMC_26561
gi|929704511~alpha_proteobacterium_AAP38
gi|930065351~alpha_proteobacterium_AAP38
gi|916723473~Niveispirillum_irakense
gi|516071135~alpha_proteobacterium_LLX12A
gi|494989526~Sphingobium_sp__AP49
gi|498023343~Sphingobium_yanoikuyae
gi|497506293~Sphingomonas_sp__SKA58
gi|1055117790~Sphingobium_sp__TCM1
gi|644243363~Sphingomonas_sanxanigenens_DSM_19645_=_NX02
gi|917643721~Porphyrobacter_mercurialis
gi|557839287~Asticcacaulis_sp__YBE204
gi|315417863~Asticcacaulis_excentricus_CB_48
gi|930036198~beta_proteobacterium_AAP121
gi|557339500~Asticcacaulis_sp__YBE204
gi|652485587~Hymenobacter_norwichensis
gi|1040537788~Pseudogymnoascus_sp__05NY08
gi|503333138~Terriglobus_saanensis_SP1PR4
gi|504032690~Granulicella_mallensis_MP5ACTX8
gi|971169402~Roseateles_depolymerans
gi|971866428~Roseateles_depolymerans
gi|948005078~Rhizobacter_sp__Root1221
gi|917629134~Methylibium_sp__CF059
gi|928913692~Amantichitinum_ursilacus
gi|917103346~Andreprevotia_chitinilytica
gi|517438938~Uliginosibacterium_gangwonense
gi|917657861~Methylibium_sp__CF468
gi|654168755~Treponema_sp__C6A8
gi|503467653~Treponema_succinifaciens_DSM_2489
gi|503421066~Clostridium_lentocellum_DSM_5427
gi|280977827~Cellulosilyticum_ruminicola_JCM_14822
gi|657702998~Lachnoclostridium_phytofermentans
gi|697199721~Clostridium_sp__KNHs205
gi|933398082|emb|CUP16809_1|~Fusicatenibacter_sp__2789STDY5834925
gi|910195661~Clostridiaceae_bacterium_MS3
gi|496549551~Lachnospiraceae_bacterium_3_1_57FAA_CT1
gi|646323150~Clostridiales_bacterium_VE202-27
gi|524768940|emb|CDE65020_1|~Fusicatenibacter_saccharivorans
gi|737800192~Butyrivibrio_sp__NC2002
gi|916593929~Butyrivibrio_sp__XPD2002
gi|503250034~Ethanoligenens_harbinense_YUAN-3
gi|646340158~Lachnospiraceae_bacterium_VE202-12
gi|511277752~Lachnospiraceae_bacterium_3_1_57FAA_CT1
gi|933395943|emb|CUQ12709_1|~Fusicatenibacter_sp__2789STDY5834925
gi|646322163~Clostridiales_bacterium_VE202-27
gi|910194506~Clostridiaceae_bacterium_MS3
gi|496116465~Pediococcus_acidilactici
gi|951440124~Lactobacillus_futsaii_JCM_17355
gi|951362974~Lactobacillus_alimentarius_DSM_20249
gi|495815090~Megamonas_funiformis_YIT_11815
gi|524450685|emb|CDB96747_1|~Megamonas_funiformis_CAG377
gi|919156455~Domibacillus_indicus
gi|505821872~Treponema_primitia_ZAS-2
gi|780122094~Bacillus_sp__TS-2
gi|1010990504~Bacillus_trypoxylicola
gi|750673739~Gorillibacterium_massiliense
gi|517763028~Gracilibacillus_lacisalsi
gi|524039286|emb|CCY19556_1|~Bifidobacterium_adolescentis_CAG119
gi|736881653~Bifidobacterium_pseudocatenulatum_IPLA36007
gi|705412858~Bifidobacterium_kashiwanohense_JCM_15439_=_DSM_21854
gi|1054772175~Ruminiclostridium_sp__KB18
gi|653041818~Robinsoniella_sp__KNHs210
gi|763463826~Robinsoniella_peoriensis
gi|652495504~Clostridium_aerotolerans
gi|654794308~Pseudobutyrivibrio_ruminis
gi|291518460|emb|CBK73681_1|~Butyrivibrio_fibrisolvens_16/4
gi|769172338~Blautia_schinkii
gi|505172809~Clostridium_stercorarium_subsp__stercorarium_DSM_8532
gi|490762068~Clostridium_termitidis_CT1112
gi|653617889~Clostridium_cellobioparum
gi|754850963~Paenibacillus_borealis
gi|754771210~Paenibacillus_sp__FSL_P4-0081
gi|918202121~Gorillibacterium_massiliense
gi|518466633~Paenibacillus_fonticola
gi|573476297~Opitutaceae_bacterium_TAV5
gi|935464589~Flavobacterium_akiainvivens
gi|924614789~Flavobacterium_akiainvivens
gi|652331647~Flavobacterium_denitrificans
gi|1031981270|dbj|BAV06671_1|~Filimonas_lacunae
gi|948191280~Pedobacter_sp__Leaf41
gi|1011203616~Geofilum_rubicundum_JCM_15548
gi|503833119~Rhodothermus_marinus_SG0_5JP17-172
gi|502606890~Rhodothermus_marinus_DSM_4252
gi|919591456~Thermogemmatispora_carboxidivorans
gi|755111650~Bacillus_sp__FF4
gi|1011376676~Lysinimicrobium_flavum
gi|1011673975~Lysinimicrobium_luteum
gi|1011679001~Lysinimicrobium_rhizosphaerae
gi|987858247~Microbacterium_hydrocarbonoxydans
gi|916609712~Microbacterium_gubbeenense
gi|947751868~Microbacterium_sp__Root553
gi|518857313~Promicromonospora_sukumoe
gi|947372977~Rathayibacter_sp__Leaf185
gi|906447467~Arthrobacter_sp__RIT-PI-e
gi|950161520~Cellulomonas_sp__Root930
gi|947429623~Cellulomonas_sp__Root137
gi|947930223~Cellulomonas_sp__Leaf334
gi|917097200~Glycomyces_sp__NRRL_B-16210
gi|916719302~Glycomyces_tenuis
gi|947470000~Sanguibacter_sp__Leaf3
gi|551252748~Nesterenkonia_alba
gi|916706658~Actinoalloteichus_cyanogriseus
gi|917106647~Actinoalloteichus_cyanogriseus
gi|917577173~Actinoalloteichus_spitiensis
gi|926391948~Saccharothrix_sp__NRRL_B-16348
gi|898242043~Nonomuraea_sp__SBT364
gi|522034501~Nonomuraea_coxensis
gi|786033237~Pseudarthrobacter_chlorophenolicus
gi|947411846~Arthrobacter_sp__Leaf137
gi|506418686~Pseudarthrobacter_chlorophenolicus_A6
gi|960375062~Arthrobacter_enclensis
gi|910250822~Pseudarthrobacter_siccitolerans
gi|948075075~Agromyces_sp__Soil535
gi|946940186~Agromyces_sp__Leaf222
gi|504505280~Actinoplanes_sp__SE50/110
gi|786069398~Actinoplanes_rectilineatus
gi|655047852~Microbacterium
gi|502629793~Sanguibacter_keddieii_DSM_10542
gi|946863204~Rathayibacter_sp__Leaf299
gi|948209638~Rathayibacter_sp__Leaf296
gi|1035532969~Agromyces_sp__AR33
gi|985826084~Streptomyces_sp__96-12
gi|930519957~Actinobacteria_bacterium_OV320
gi|930468538~Actinobacteria_bacterium_OK074
gi|1055780783~Streptomyces_olivochromogenes
gi|985844428~Streptomyces_diastatochromogenes
gi|493323822~Prevotella_bryantii_B14
gi|653247351~Prevotella_bryantii
gi|524137726|emb|CCZ12101_1|~Prevotella_sp__CAG1092
gi|918571779~Bacteroides_reticulotermitis_JCM_10512
gi|1011261655~Dysgonomonas_macrotermitis
gi|501342552~Opitutus_terrae_PB90-1
gi|1031959407~Opitutaceae_bacterium_TSB47
GSMAST31_AYS1_ANO1_2596_1
gi|524454070|emb|CDB97049_1|~Firmicutes_bacterium_CAG41
gi|1052818609|emb|SCG95020_1|~uncultured_Clostridium_sp_
gi|916900474~Saccharibacillus_sacchari
gi|524609893|emb|CDD30955_1|~Firmicutes_bacterium_CAG94
gi|917578128~Pseudobutyrivibrio_ruminis
gi|909846307~Pseudobutyrivibrio_sp__LB2011
gi|1030466061|emb|SAV24557_1|~Klebsiella_pneumoniae
gi|1053021189|emb|SCI53523_1|~uncultured_Clostridium_sp_
gi|524442515|emb|CDB86546_1|~Firmicutes_bacterium_CAG170
gi|919104173~Caldicellulosiruptor_sp__Wai35_B1
gi|503167575~Caldicellulosiruptor_hydrothermalis_108
gi|651374503~Caldanaerobius_polysaccharolyticus
gi|495949872~Bacteroides_sp__9_1_42FAA
gi|887752691~Bacteroides_sp__9_1_42FAA
gi|1054854562~Rufibacter_roseus
gi|1053210188~Mucilaginibacter_sp__PPCGB_2223
gi|495787266~Mucilaginibacter_paludis_DSM_18603
gi|498500562~Parabacteroides_goldsteinii_dnLKV18
gi|497546368~Parabacteroides_goldsteinii_CL02T12C30
gi|524759932|emb|CDE62525_1|~Parabacteroides_sp__CAG409
gi|723441117~Sphingobacteriaceae_bacterium_DW12
gi|498212430~Thermophagus_xiamenensis
gi|919430840~Tamlana_nanhaiensis
0.2
Bacteria
Eukaryota
SAG
NA

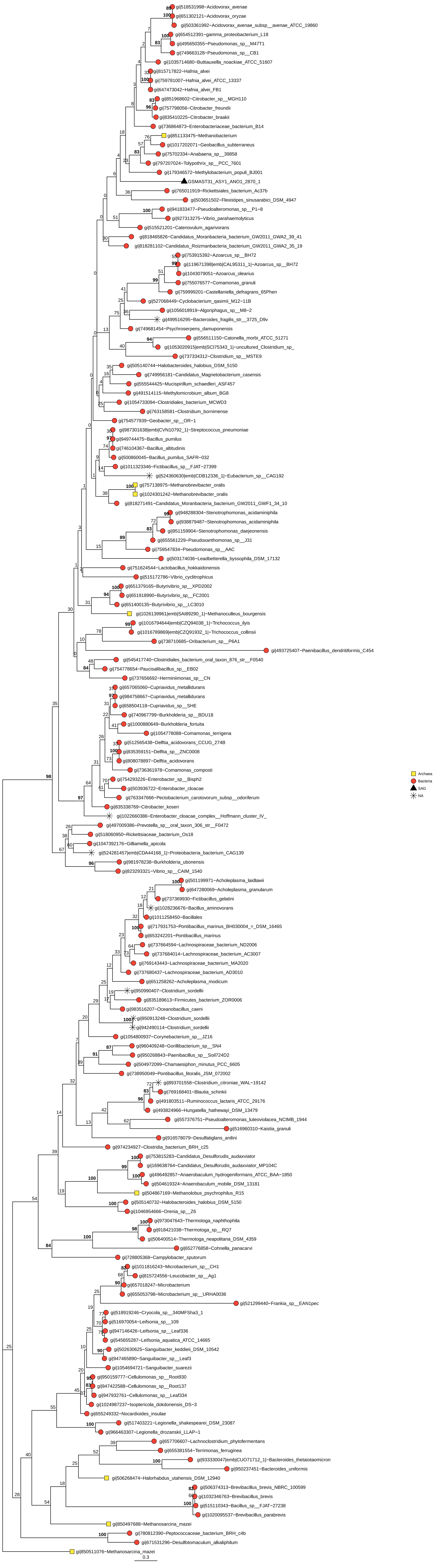
gi|518531998~Acidovorax_avenae
gi|551302121~Acidovorax_oryzae
gi|503361992~Acidovorax_avenae_subsp__avenae_ATCC_19860
gi|654512391~gamma_proteobacterium_L18
gi|495650355~Pseudomonas_sp__M47T1
gi|749663128~Pseudomonas_sp__CB1
gi|1035714680~Buttiauxella_noackiae_ATCC_51607
gi|815717822~Hafnia_alvei
gi|759781007~Hafnia_alvei_ATCC_13337
gi|647473042~Hafnia_alvei_FB1
gi|851968602~Citrobacter_sp__MGH110
gi|757798056~Citrobacter_freundii
gi|835410225~Citrobacter_braakii
gi|736864873~Enterobacteriaceae_bacterium_B14
gi|851133475~Methanobacterium
gi|1017202071~Geobacillus_subterraneus
gi|75702334~Anabaena_sp__39858
gi|797207024~Tolypothrix_sp__PCC_7601
gi|179346572~Methylobacterium_populi_BJ001
GSMAST31_ASY1_ANO1_2870_1
gi|765011919~Rickettsiales_bacterium_Ac37b
gi|503651502~Flexistipes_sinusarabici_DSM_4947
gi|941833477~Pseudoalteromonas_sp__P1-8
gi|927313275~Vibrio_parahaemolyticus
gi|515521201~Catenovulum_agarivorans
gi|818465826~Candidatus_Moranbacteria_bacterium_GW2011_GWA2_39_41
gi|818281102~Candidatus_Roizmanbacteria_bacterium_GW2011_GWA2_35_19
gi|753915392~Azoarcus_sp__BH72
gi|119671398|emb|CAL95311_1|~Azoarcus_sp__BH72
gi|1043079051~Azoarcus_olearius
gi|755076577~Comamonas_granuli
gi|759999201~Castellaniella_defragrans_65Phen
gi|527068449~Cyclobacterium_qasimii_M12-11B
gi|1056018919~Algoriphagus_sp__M8-2
gi|499516295~Bacteroides_fragilis_str__3725_D9v
gi|749681454~Psychroserpens_damuponensis
gi|556511150~Catonella_morbi_ATCC_51271
gi|1053020915|emb|SCI75343_1|~uncultured_Clostridium_sp_
gi|737334312~Clostridium_sp__MSTE9
gi|505140744~Halobacteroides_halobius_DSM_5150
gi|749956181~Candidatus_Magnetobacterium_casensis
gi|555544425~Mucispirillum_schaedleri_ASF457
gi|491514115~Methylomicrobium_album_BG8
gi|1054733094~Clostridiales_bacterium_MCWD3
gi|763158581~Clostridium_bornimense
gi|754577939~Geobacter_sp__OR-1
gi|987301638|emb|CVN10792_1|~Streptococcus_pneumoniae
gi|949744475~Bacillus_pumilus
gi|746104367~Bacillus_altitudinis
gi|500860045~Bacillus_pumilus_SAFR-032
gi|1011323346~Fictibacillus_sp__FJAT-27399
gi|524360630|emb|CDB12336_1|~Eubacterium_sp__CAG192
gi|757138975~Methanobrevibacter_oralis
gi|1024301242~Methanobrevibacter_oralis
gi|818271491~Candidatus_Moranbacteria_bacterium_GW2011_GWF1_34_10
gi|948288304~Stenotrophomonas_acidaminiphila
gi|938879487~Stenotrophomonas_acidaminiphila
gi|951159904~Stenotrophomonas_daejeonensis
gi|655561229~Pseudoxanthomonas_sp__J31
gi|759547834~Pseudomonas_sp__AAC
gi|503174036~Leadbetterella_byssophila_DSM_17132
gi|751624544~Lactobacillus_hokkaidonensis
gi|515172786~Vibrio_cyclitrophicus
gi|651379165~Butyrivibrio_sp__XPD2002
gi|651918990~Butyrivibrio_sp__FC2001
gi|651400135~Butyrivibrio_sp__LC3010
gi|1026139961|emb|SAI89290_1|~Methanoculleus_bourgensis
gi|1016794644|emb|CZQ94038_1|~Trichococcus_ilyis
gi|1016789869|emb|CZQ91932_1|~Trichococcus_collinsii
gi|738710685~Oribacterium_sp__P6A1
gi|493725407~Paenibacillus_dendritiformis_C454
gi|545417740~Clostridiales_bacterium_oral_taxon_876_str__F0540
gi|754778654~Paucisalibacillus_sp__EB02
gi|737656692~Herminiimonas_sp__CN
gi|657065060~Cupriavidus_metallidurans
gi|984758667~Cupriavidus_metallidurans
gi|658504118~Cupriavidus_sp__SHE
gi|740967799~Burkholderia_sp__BDU18
gi|1000880649~Burkholderia_fortuita
gi|1054778088~Comamonas_terrigena
gi|512565438~Delftia_acidovorans_CCUG_274B
gi|835359151~Delftia_sp__ZNC0008
gi|808078897~Delftia_acidovorans
gi|736361978~Comamonas_composti
gi|754293226~Enterobacter_sp__Bisph2
gi|503936722~Enterobacter_cloacae
gi|763347666~Pectobacterium_carotovorum_subsp__odoriferum
gi|835338769~Citrobacter_koseri
gi|1022660386~Enterobacter_cloacae_complex__Hoffmann_cluster_IV_
gi|497009386~Prevotella_sp__oral_taxon_306_str__F0472
gi|518060950~Rickettsiaceae_bacterium_Os18
gi|1047392176~Gilliamella_apicola
gi|524281457|emb|CDA44168_1|~Proteobacteria_bacterium_CAG139
gi|981978238~Burkholderia_ubonensis
gi|823293321~Vibrio_sp__CAIM_1540
gi|501199971~Acholeplasma_laidlawii
gi|647280069~Acholeplasma_granularum
gi|737369930~Ficitbacillus_gelatini
gi|1028236676~Bacillus_aminovorans
gi|1011258450~Bacillales
gi|717931753~Pontibacillus_marinus_BH030004_=_DSM_16465
gi|653242201~Pontibacillus_marinus
gi|737664594~Lachnospiraceae_bacterium_ND2006
gi|737684014~Lachnospiraceae_bacterium_AC3007
gi|769143443~Lachnospiraceae_bacterium_MA2020
gi|737680437~Lachnospiraceae_bacterium_AD3010
gi|651258262~Acholeplasma_modicum
gi|950990407~Clostridium_sordellii
gi|835189613~Firmicutes_bacterium_ZOR0006
gi|983516207~Oceanobacillus_caeni
gi|950913248~Clostridium_sordellii
gi|942490114~Clostridium_sordellii
gi|1054800937~Corynebacterium_sp__JZ16
gi|960409248~Gorillibacterium_sp__SN4
gi|950268843~Paenibacillus_sp__Soil724D2
gi|504972099~Chamaesiphon_minutus_PCC_6605
gi|738950049~Pontibacillus_litoralis_JSM_072002
gi|893701558~Clostridium_citroniae_WAL-19142
gi|769168401~Blautia_schinkii
gi|491803511~Ruminococcus_lactaris_ATCC_29176
gi|493824966~Hungatella_hathewayi_DSM_13479
gi|557376751~Pseudoalteromonas_luteoviolacea_NCIMB_1944
gi|516960310~Kaistia_granuli
gi|916578079~Desulfatiglans_anilini
gi|974234927~Clostridia_bacterium_BRH_c25
gi|753815283~Candidatus_Desulforudis_audaxviator
gi|169638764~Candidatus_Desulforudis_audaxviator_MP104C
gi|496492857~Anaerobaculum_hydrogeniformans_ATCC_BAA-1850
gi|504619324~Anaerobaculum_mobile_DSM_13181
gi|504867169~Methanolobus_psychrophilus_R15
gi|505140732~Halobacteroides_halobius_DSM_5150
gi|1046954666~Orenia_sp__Z6
gi|973047643~Thermotoga_naphthophila
gi|918421038~Thermotoga_sp__RQ7
gi|506400514~Thermotoga_neapolitana_DSM_4359
gi|652776858~Cohnella_panacarvi
gi|728805368~Campylobacter_sputorum
gi|1011816243~Microbacterium_sp__CH1
gi|815724556~Leucobacter_sp__Ag1
gi|657018247~Microbacterium
gi|655053798~Microbacterium_sp__URHA0036
gi|521299440~Frankia_sp__EAN1pec
gi|518919246~Cryocola_sp__340MFSha3_1
gi|516970054~Leifsonia_sp__109
gi|947146426~Leifsonia_sp__Leaf336
gi|545655287~Leifsonia_aquatica_ATCC_14665
gi|502630625~Sanguibacter_keddieii_DSM_10542
gi|947465890~Sanguibacter_sp__Leaf3
gi|1054694721~Sanguibacter_suarezii
gi|950159777~Cellulomonas_sp__Root930
gi|947422588~Cellulomonas_sp__Root137
gi|947932761~Cellulomonas_sp__Leaf334
gi|1024987237~Isoptericola_dokdonensis_DS-3
gi|655249332~Nocardioides_insulae
gi|517403221~Legionella_shakespearei_DSM_23087
gi|966463307~Legionella_drozanskii_LLAP-1
gi|657706607~Lachnoclostridium_phytofermentans
gi|655381554~Terrimonas_ferruginea
gi|933330047|emb|CUO71712_1|~Bacteroides_thetaiotaomicron
gi|950237451~Bacteroides_uniformis
gi|506268474~Halorhabdus_utahensis_DSM_12940
gi|506374313~Brevibacillus_brevis_NBRC_100599
gi|1032346763~Brevibacillus_brevis
gi|515110343~Bacillus_sp__FJAT-27238
gi|1020095537~Brevibacillus_parabrevis
gi|850497688~Methanosarcina_mazei
gi|780812390~Peptococcaceae_bacterium_BRH_c4b
gi|671531296~Desulfotomaculum_alkaliphilum
gi|850511076~Methanosarcina_mazei
0.3
Archaea
Bacteria
SAG
NA

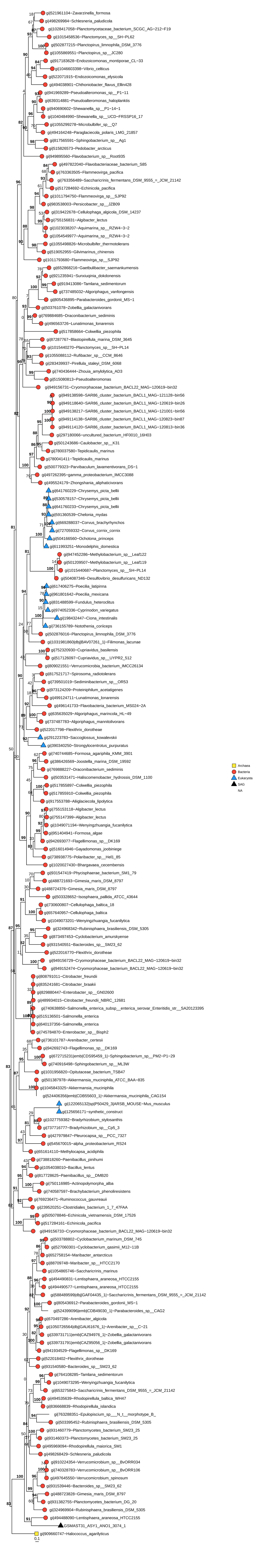
Archaea
Bacteria
Eukaryota
SAG
NA
0.1

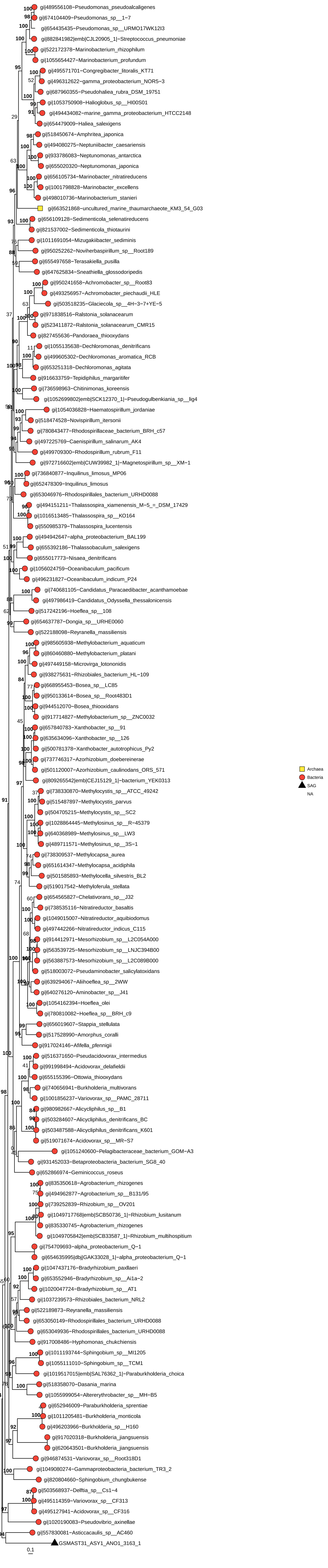
gi|489556108~Pseudomonas_pseudoalcaligenes
gi|674104409~Pseudomonas_sp__1~7
gi|654435435~Pseudomonas_sp__URMO17WK12I3
gi|882841982|emb|CJL20905_1|~Streptococcus_pneumoniae
gi|522172378~Marinobacterium_rhizophilum
gi|1055654427~Marinobacterium_profundum
gi|495571701~Congregibacter_litoralis_KT71
gi|496312622~gamma_proteobacterium_NOR5~3
gi|687960355~Pseudohaliea_rubra_DSM_19751
gi|1053750908~Halioglobus_sp__HI00S01
gi|494434082~marine_gamma_proteobacterium_HTCC2148
gi|654479009~Haliea_salexigens
gi|518450674~Amphritea_japonica
gi|494080275~Neptuniibacter_caesariensis
gi|933786083~Neptunomonas_antarctica
gi|655020320~Neptunomonas_japonica
gi|656105734~Marinobacter_nitratireducens
gi|1001798828~Marinobacter_excellens
gi|498010736~Marinobacterium_stanieri
gi|663521868~uncultured_marine_thaumarchaeote_KM3_54_G03
gi|656109128~Sedimenticola_selenatireducens
gi|821537002~Sedimenticola_thiotaurini
gi|1011691054~Mizugakiibacter_sediminis
gi|950252262~Noviherbaspirillum_sp__Root189
gi|655497658~Terasakiella_pusilla
gi|647625834~Sneathiella_glossodoripedis
gi|950241658~Achromobacter_sp__Root83
gi|493256957~Achromobacter_piechaudii_HLE
gi|503518235~Glaciecola_sp__4H~3~7+YE~5
gi|971838516~Ralstonia_solanacearum
gi|523411872~Ralstonia_solanacearum_CMR15
gi|827455636~Pandoraea_thiooxydans
gi|1055135638~Dechloromonas_denitrificans
gi|499605302~Dechloromonas_aromatica_RCB
gi|653251318~Dechloromonas_agitata
gi|916633759~Tepidiphilus_margaritifer
gi|736598963~Chitinimonas_koreensis
gi|1052699802|emb|SCK12370_1|~Pseudogulbenkiania_sp__lig4
gi|1054036828~Haematospirillum_jordaniae
gi|518474528~Novispirillum_itersonii
gi|780843477~Rhodospirillaceae_bacterium_BRH_c57
gi|497225769~Caenispirillum_salinarum_AK4
gi|499709300~Rhodospirillum_rubrum_F11
gi|972716602|emb|CUW39982_1|~Magnetospirillum_sp__XM~1
gi|736840877~Inquilinus_limosus_MP06
gi|652478309~Inquilinus_limosus
gi|653046976~Rhodospirillales_bacterium_URHD0088
gi|494151211~Thalassospira_xiamenensis_M~5_=_DSM_17429
gi|1016513485~Thalassospira_sp__KO164
gi|550985379~Thalassospira_lucentensis
gi|494942647~alpha_proteobacterium_BAL199
gi|655392186~Thalassobaculum_salexigens
gi|655017323~Nisaea_denitrificans
gi|1056024759~Oceanibaculum_pacificum
gi|496231827~Oceanibaculum_indicum_P24
gi|740681105~Candidatus_Paracaedibacter_acanthamoebae
gi|497986419~Candidatus_Odyssella_thessalonicensis
gi|517242196~Hoeflea_sp__108
gi|654637787~Dongia_sp__URHE0060
gi|522188098~Reyranella_massiliensis
gi|985605938~Methylobacterium_aquaticum
gi|860460880~Methylobacterium_platani
gi|497449158~Microvirga_lotononidis
gi|938275631~Rhizobiales_bacterium_HL~109
gi|668955453~Bosea_sp__LC85
gi|950133614~Bosea_sp__Root483D1
gi|944512070~Bosea_thiooxidans
gi|917714827~Methylobacterium_sp__ZNC0032
gi|657840783~Xanthobacter_sp__91
gi|635634096~Xanthobacter_sp__126
gi|500781378~Xanthobacter_autotrophicus_Py2
gi|737746317~Azorhizobium_doebereinerae
gi|501120007~Azorhizobium_caulinodans_ORS_571
gi|809265542|emb|CEJ15129_1|~bacterium_YEK0313
gi|738330870~Methylocystis_sp__ATCC_49242
gi|515487897~Methylocystis_parvus
gi|504705215~Methylocystis_sp__SC2
gi|1028864445~Methylosinus_sp__R~45379
gi|640368989~Methylosinus_sp__LW3
gi|489711571~Methylosinus_sp__3S~1
gi|738309537~Methylocapsa_aurea
gi|651614347~Methylocapsa_acidiphila
gi|501585893~Methylocella_silvestris_BL2
gi|519017542~Methyloferula_stellata
gi|654565827~Chelativorans_sp__J32
gi|738535116~Nitratireductor_basaltis
gi|1049015007~Nitratireductor_aquibiodomus
gi|497442266~Nitratireductor_indicus_C115
gi|914412971~Mesorhizobium_sp__L2C054A000
gi|563539725~Mesorhizobium_sp__LNJC394B00
gi|563887573~Mesorhizobium_sp__L2C089B000
gi|518003072~Pseudaminobacter_salicylatoxidans
gi|639294067~Aliihoeflea_sp__2WW
gi|640276120~Aminobacter_sp__J41
gi|1054162394~Hoeflea_olei
gi|780810682~Hoeflea_sp__BRH_c9
gi|656019607~Stappia_stellulata
gi|517528990~Amorphus_coralli
gi|917024146~Afifella_pfennigii
gi|516371650~Pseudacidovorax_intermedius
gi|991998494~Acidovorax_delafieldii
gi|655155396~Ottowia_thiooxydans
gi|740656941~Burkholderia_multivorans
gi|1001856237~Variovorax_sp__PAMC_28711
gi|980982667~Alicycliphilus_sp__B1
gi|503284607~Alicycliphilus_denitrificans_BC
gi|503487588~Alicycliphilus_denitrificans_K601
gi|519071674~Acidovorax_sp__MR~S7
gi|1051240600~Pelagibacteraceae_bacterium_GOM~A3
gi|931452033~Betaproteobacteria_bacterium_SG8_40
gi|652866974~Geminicoccus_roseus
gi|835350618~Agrobacterium_rhizogenes
gi|494962877~Agrobacterium_sp__B131/95
gi|739252839~Rhizobium_sp__OV201
gi|1049717768|emb|SCB50736_1|~Rhizobium_lusitanum
gi|835330745~Agrobacterium_rhizogenes
gi|1049705842|emb|SCB33587_1|~Rhizobium_multihospitium
gi|754709693~alpha_proteobacterium_Q~1
gi|654635995|dbj|GAK33028_1|~alpha_proteobacterium_Q~1
gi|1047437176~Bradyrhizobium_paxllaeri
gi|653552946~Bradyrhizobium_sp__Ai1a~2
gi|1020047724~Bradyrhizobium_sp__AT1
gi|1037239573~Rhizobiales_bacterium_NRL2
gi|522189873~Reyranella_massiliensis
gi|653050149~Rhodospirillales_bacterium_URHD0088
gi|653049936~Rhodospirillales_bacterium_URHD0088
gi|917008486~Hyphomonas_chukchiensis
gi|1011193744~Sphingobium_sp__MI1205
gi|1055111010~Sphingobium_sp__TCM1
gi|1019517015|emb|SAL76362_1|~Paraburkholderia_choica
gi|518358070~Dasania_marina
gi|1055999054~Altererythrobacter_sp__MH~B5
gi|652946009~Paraburkholderia_sprentiae
gi|1011205481~Burkholderia_monticola
gi|496203966~Burkholderia_sp__H160
gi|917020318~Burkholderia_jiangsuensis
gi|620643501~Burkholderia_jiangsuensis
gi|946874531~Variovorax_sp__Root318D1
gi|1049080274~Gammaproteobacteria_bacterium_TR3_2
gi|820804660~Sphingobium_chungbukense
gi|503568937~Delftia_sp__Cs1~4
gi|495114359~Variovorax_sp__CF313
gi|495127941~Acidovorax_sp__CF316
gi|1020190083~Pseudovibrio_axinellae
gi|557830081~Asticcacaulis_sp__AC460
GSMAST31_ASY1_ANO1_3163_1
0.1
Archaea
Bacteria
SAG
NA

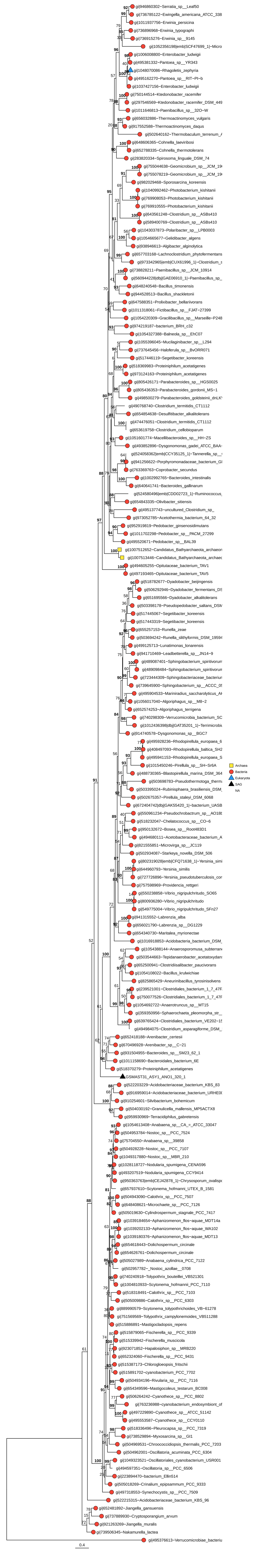

Archaea
Bacteria
Eukaryota
SAG
NA
0.4

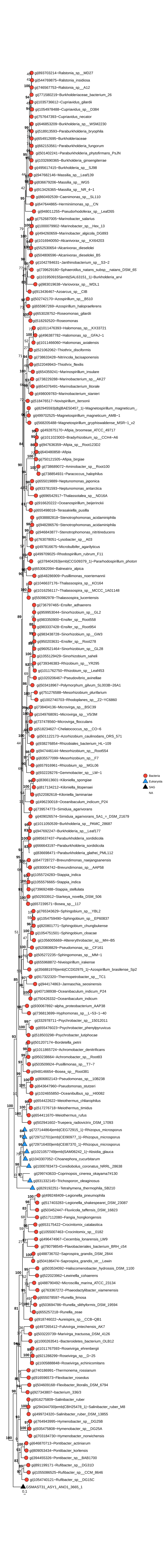
Bacteria
Eukaryota
SAG
NA
0.1

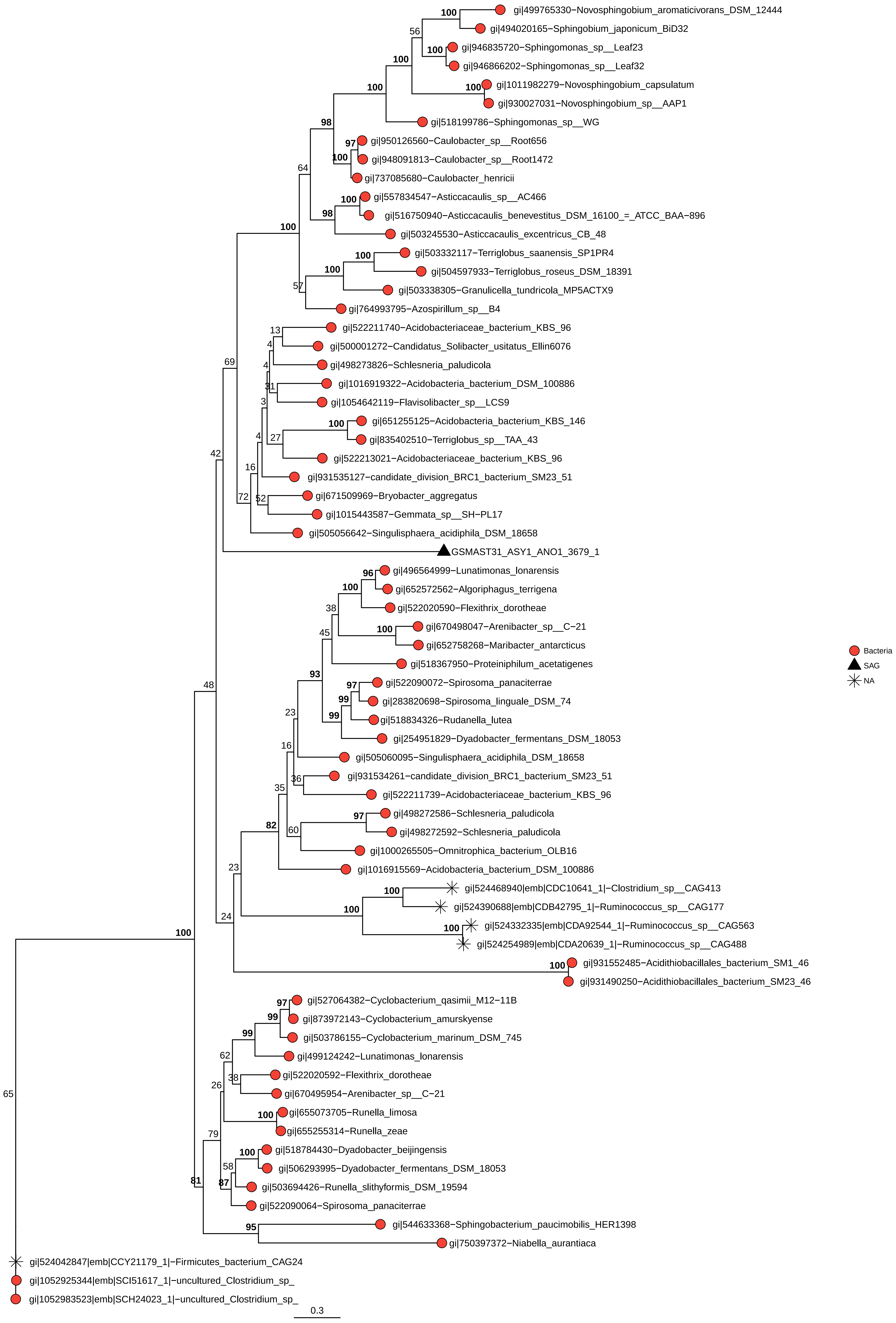
gi|499765330−Novosphingobium_aromaticivorans_DSM_12444
gi|494020165−Sphingobium_japonicum_BiD32
gi|946835720−Sphingomonas_sp__Leaf23
gi|946866202−Sphingomonas_sp__Leaf32
gi|1011982279−Novosphingobium_capsulatum
gi|930027031−Novosphingobium_sp__AAP1
gi|518199786−Sphingomonas_sp__WG
gi|950126560−Caulobacter_sp__Root656
gi|948091813−Caulobacter_sp__Root1472
gi|737085680−Caulobacter_henricii
gi|557834547−Asticcacaulis_sp__AC466
gi|516750940−Asticcacaulis_benevestitus_DSM_16100_=_ATCC_BAA−896
gi|503245530−Asticcacaulis_excentricus_CB_48
gi|503332117−Terriglobus_saanensis_SP1PR4
gi|504597933−Terriglobus_roseus_DSM_18391
gi|503338305−Granulicella_tundricola_MP5ACTX9
gi|764993795−Azospirillum_sp__B4
gi|522211740−Acidobacteriaceae_bacterium_KBS_96
gi|500001272−Candidatus_Solibacter_usitatus_Ellin6076
gi|498273826−Schlesneria_paludicola
gi|1016919322−Acidobacteria_bacterium_DSM_100886
gi|1054642119−Flavisolibacter_sp__LCS9
gi|651255125−Acidobacteria_bacterium_KBS_146
gi|835402510−Terriglobus_sp__TAA_43
gi|522213021−Acidobacteriaceae_bacterium_KBS_96
gi|931535127−candidate_division_BRC1_bacterium_SM23_51
gi|671509969−Bryobacter_aggregatus
gi|1015443587−Gemmata_sp__SH−PL17
gi|505056642−Singulisphaera_acidiphila_DSM_18658
GSMAST31_ASY1_ANO1_3679_1
gi|496564999−Lunatimonas_lonarensis
gi|652572562−Algoriphagus_terrigena
gi|522020590−Flexithrix_dorotheae
gi|670498047−Arenibacter_sp__C−21
gi|652758268−Maribacter_antarcticus
gi|518367950−Proteiniphilum_acetatigenes
gi|522090072−Spirosoma_panaciterrae
gi|283820698−Spirosoma_linguale_DSM_74
gi|518834326−Rudanella_lutea
gi|254951829−Dyadobacter_fermentans_DSM_18053
gi|505060095−Singulisphaera_acidiphila_DSM_18658
gi|931534261−candidate_division_BRC1_bacterium_SM23_51
gi|522211739−Acidobacteriaceae_bacterium_KBS_96
gi|498272586−Schlesneria_paludicola
gi|498272592−Schlesneria_paludicola
gi|1000265505−Omnitrophica_bacterium_OLB16
gi|1016915569−Acidobacteria_bacterium_DSM_100886
gi|524468940|emb|CDC10641_1|−Clostridium_sp__CAG413
gi|524390688|emb|CDB42795_1|−Ruminococcus_sp__CAG177
gi|524332335|emb|CDA92544_1|−Ruminococcus_sp__CAG563
gi|524254989|emb|CDA20639_1|−Ruminococcus_sp__CAG488
gi|931552485−Acidithiobacillales_bacterium_SM1_46
gi|931490250−Acidithiobacillales_bacterium_SM23_46
gi|527064382−Cyclobacterium_qasimii_M12−11B
gi|873972143−Cyclobacterium_amurskyense
gi|503786155−Cyclobacterium_marinum_DSM_745
gi|499124242−Lunatimonas_lonarensis
gi|522020592−Flexithrix_dorotheae
gi|670495954−Arenibacter_sp__C−21
gi|655073705−Runella_limosa
gi|655255314−Runella_zeae
gi|518784430−Dyadobacter_beijingensis
gi|506293995−Dyadobacter_fermentans_DSM_18053
gi|503694426−Runella_slithyformis_DSM_19594
gi|522090064−Spirosoma_panaciterrae
gi|544633368−Sphingobacterium_paucimobilis_HER1398
gi|750397372−Niabella_aurantiaca
gi|524042847|emb|CCY21179_1|−Firmicutes_bacterium_CAG24
gi|1052925344|emb|SCI51617_1|−uncultured_Clostridium_sp_
gi|1052983523|emb|SCH24023_1|−uncultured_Clostridium_sp_
0.3
Bacteria
SAG
NA

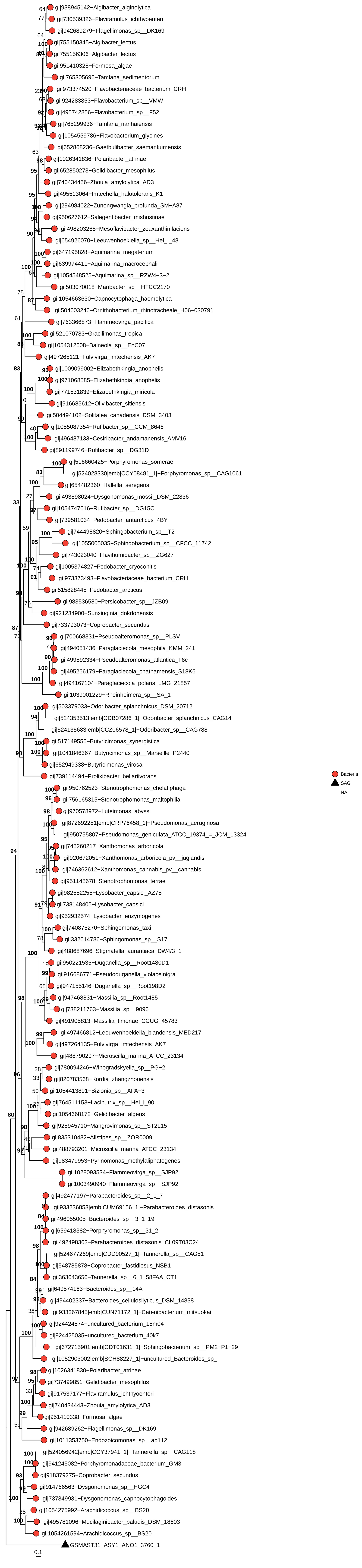
gi|938945142~Algibacter_alginolytica
gi|730539326~Flaviramulus_ichthyoenteri
gi|942689279~Flagellimonas_sp__DK169
gi|755150345~Algibacter_lectus
gi|755156306~Algibacter_lectus
gi|951410328~Formosa_algae
gi|765305696~Tamlana_sedimentorum
gi|973374520~Flavobacteriaceae_bacterium_CRH
gi|924283853~Flavobacterium_sp__VMW
gi|495742856~Flavobacterium_sp__F52
gi|765299936~Tamlana_nanhaiensis
gi|1054559786~Flavobacterium_glycines
gi|652868236~Gaetbulibacter_saemankumensis
gi|1026341836~Polaribacter_atrinae
gi|652850273~Gelidibacter_mesophilus
gi|740434456~Zhouia_amylolytica_AD3
gi|495513064~Imtechella_halotolerans_K1
gi|294984022~Zunongwangia_profunda_SM~A87
gi|950627612~Salegentibacter_mishustinae
gi|498203265~Mesoflavibacter_zeaxanthinifaciens
gi|654926070~Leeuwenhoekiella_sp__Hel_I_48
gi|647195828~Aquimarina_megaterium
gi|639974411~Aquimarina_macrocephali
gi|1054548525~Aquimarina_sp__RZW4~3~2
gi|503070018~Maribacter_sp__HTCC2170
gi|1054663630~Capnocytophaga_haemolytica
gi|504603246~Ornithobacterium_rhinotracheale_H06~030791
gi|763366873~Flammeovirga_pacifica
gi|521070783~Gracilimonas_tropica
gi|1054312608~Balneola_sp__EhC07
gi|497265121~Fulvivirga_imtechensis_AK7
gi|1009099002~Elizabethkingia_anophelis
gi|971068585~Elizabethkingia_anophelis
gi|771531839~Elizabethkingia_miricola
gi|916685612~Olivibacter_sitiensis
gi|504494102~Solitalea_canadensis_DSM_3403
gi|1055087354~Rufibacter_sp__CCM_8646
gi|496487133~Cesiribacter_andamanensis_AMV16
gi|891199746~Rufibacter_sp__DG31D
gi|516660425~Porphyromonas_somerae
gi|524028330|emb|CCY08481_1|~Porphyromonas_sp__CAG1061
gi|654482360~Hallella_seregens
gi|493898024~Dysgonomonas_mossii_DSM_22836
gi|1054747616~Rufibacter_sp__DG15C
gi|739581034~Pedobacter_antarcticus_4BY
gi|744498820~Sphingobacterium_sp__T2
gi|1055005035~Sphingobacterium_sp__CFCC_11742
gi|743023040~Flavihumibacter_sp__ZG627
gi|1005374827~Pedobacter_cryoconitis
gi|973373493~Flavobacteriaceae_bacterium_CRH
gi|515828445~Pedobacter_arcticus
gi|983536580~Persicobacter_sp__JZB09
gi|921234900~Sunxiuqinia_dokdonensis
gi|733793073~Coprobacter_secundus
gi|700668331~Pseudoalteromonas_sp__PLSV
gi|494051436~Paraglaciecola_mesophila_KMM_241
gi|499892334~Pseudoalteromonas_atlantica_T6c
gi|495266179~Paraglaciecola_chathamensis_S18K6
gi|494167104~Paraglaciecola_polaris_LMG_21857
gi|1039001229~Rheinheimera_sp__SA_1
gi|503379033~Odoribacter_splanchnicus_DSM_20712
gi|524353513|emb|CDB07286_1|~Odoribacter_splanchnicus_CAG14
gi|524135683|emb|CCZ06578_1|~Odoribacter_sp__CAG788
gi|517149556~Butyricimonas_synergistica
gi|1041846367~Butyricimonas_sp__Marseille~P2440
gi|652949338~Butyricimonas_virosa
gi|739114494~Prolixibacter_bellariivorans
gi|950762523~Stenotrophomonas_chelatiphaga
gi|756165315~Stenotrophomonas_maltophilia
gi|970578972~Luteimonas_abyssi
gi|872692281|emb|CRP76458_1|~Pseudomonas_aeruginosa
gi|950755807~Pseudomonas_geniculata_ATCC_19374_=_JCM_13324
gi|748260217~Xanthomonas_arboricola
gi|920672051~Xanthomonas_arboricola_pv__juglandis
gi|746362612~Xanthomonas_cannabis_pv__cannabis
gi|951148678~Stenotrophomonas_terrae
gi|982582255~Lysobacter_capsici_AZ78
gi|738148405~Lysobacter_capsici
gi|952932574~Lysobacter_enzymogenes
gi|740875270~Sphingomonas_taxi
gi|332014786~Sphingomonas_sp__S17
gi|488687696~Stigmatella_aurantiaca_DW4/3~1
gi|950221535~Duganella_sp__Root1480D1
gi|916686771~Pseudoduganella_violaceinigra
gi|947155146~Duganella_sp__Root198D2
gi|947468831~Massilia_sp__Root1485
gi|738211763~Massilia_sp__9096
gi|491905813~Massilia_timonae_CCUG_45783
gi|497466812~Leeuwenhoekiella_blandensis_MED217
gi|497264135~Fulvivirga_imtechensis_AK7
gi|488790297~Microscilla_marina_ATCC_23134
gi|780094246~Winogradskyella_sp__PG~2
gi|820783568~Kordia_zhangzhouensis
gi|1054413891~Bizionia_sp__APA~3
gi|764511153~Lacinutrix_sp__Hel_I_90
gi|1054668172~Gelidibacter_algens
gi|928945710~Mangrovimonas_sp__ST2L15
gi|835310482~Alistipes_sp__ZOR0009
gi|488793201~Microscilla_marina_ATCC_23134
gi|983479953~Pyrinomonas_methylaliphatogenes
gi|1028093534~Flammeovirga_sp__SJP92
gi|1003490940~Flammeovirga_sp__SJP92
gi|492477197~Parabacteroides_sp__2_1_7
gi|933236853|emb|CUM69156_1|~Parabacteroides_distasonis
gi|496055005~Bacteroides_sp__3_1_19
gi|659418382~Porphyromonas_sp__31_2
gi|492498363~Parabacteroides_distasonis_CL09T03C24
gi|524677269|emb|CDD90527_1|~Tannerella_sp__CAG51
gi|548785878~Coprobacter_fastidiosus_NSB1
gi|363643656~Tannerella_sp__6_1_58FAA_CT1
gi|649574163~Bacteroides_sp__14A
gi|494402337~Bacteroides_cellulosilyticus_DSM_14838
gi|933367845|emb|CUN71172_1|~Catenibacterium_mitsuokai
gi|924424574~uncultured_bacterium_15m04
gi|924425035~uncultured_bacterium_40k7
gi|672715901|emb|CDT01631_1|~Sphingobacterium_sp__PM2~P1~29
gi|1052903002|emb|SCH88227_1|~uncultured_Bacteroides_sp_
gi|1026341830~Polaribacter_atrinae
gi|737499851~Gelidibacter_mesophilus
gi|917537177~Flaviramulus_ichthyoenteri
gi|740434443~Zhouia_amylolytica_AD3
gi|951410338~Formosa_algae
gi|942689262~Flagellimonas_sp__DK169
gi|1011353750~Endozoicomonas_sp__ab112
gi|524056942|emb|CCY37941_1|~Tannerella_sp__CAG118
gi|941245082~Porphyromonadaceae_bacterium_GM3
gi|918379275~Coprobacter_secundus
gi|914766563~Dysgonomonas_sp__HGC4
gi|737349931~Dysgonomonas_capnocytophagoides
gi|1054275992~Arachidicoccus_sp__BS20
gi|495781096~Mucilaginibacter_paludis_DSM_18603
gi|1054261594~Arachidicoccus_sp__BS20
GSMAST31_ASY1_ANO1_3760_1
0.1
Bacteria
SAG
NA

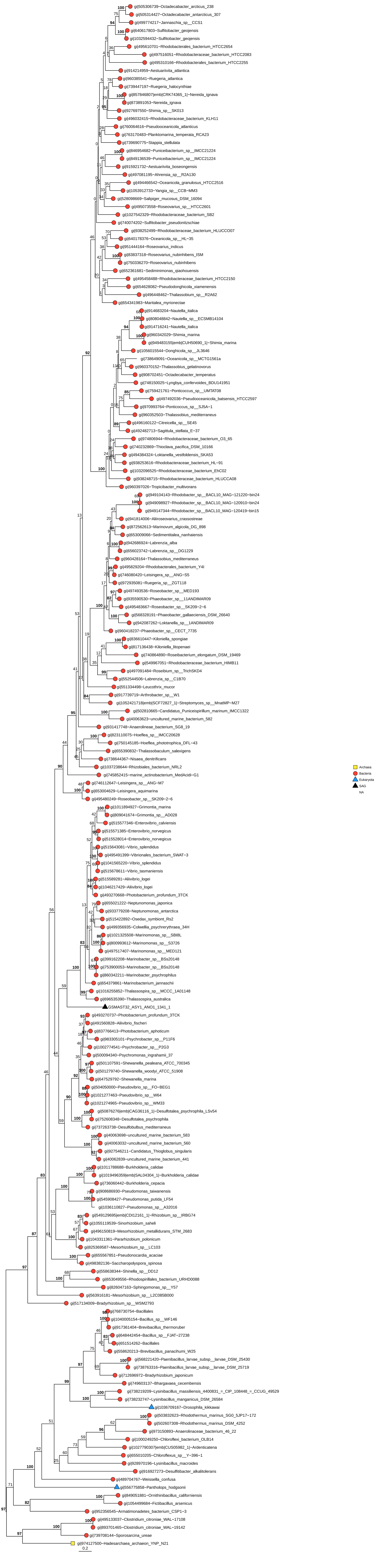
Archaea
Bacteria
Eukaryota
SAG
NA
0.2

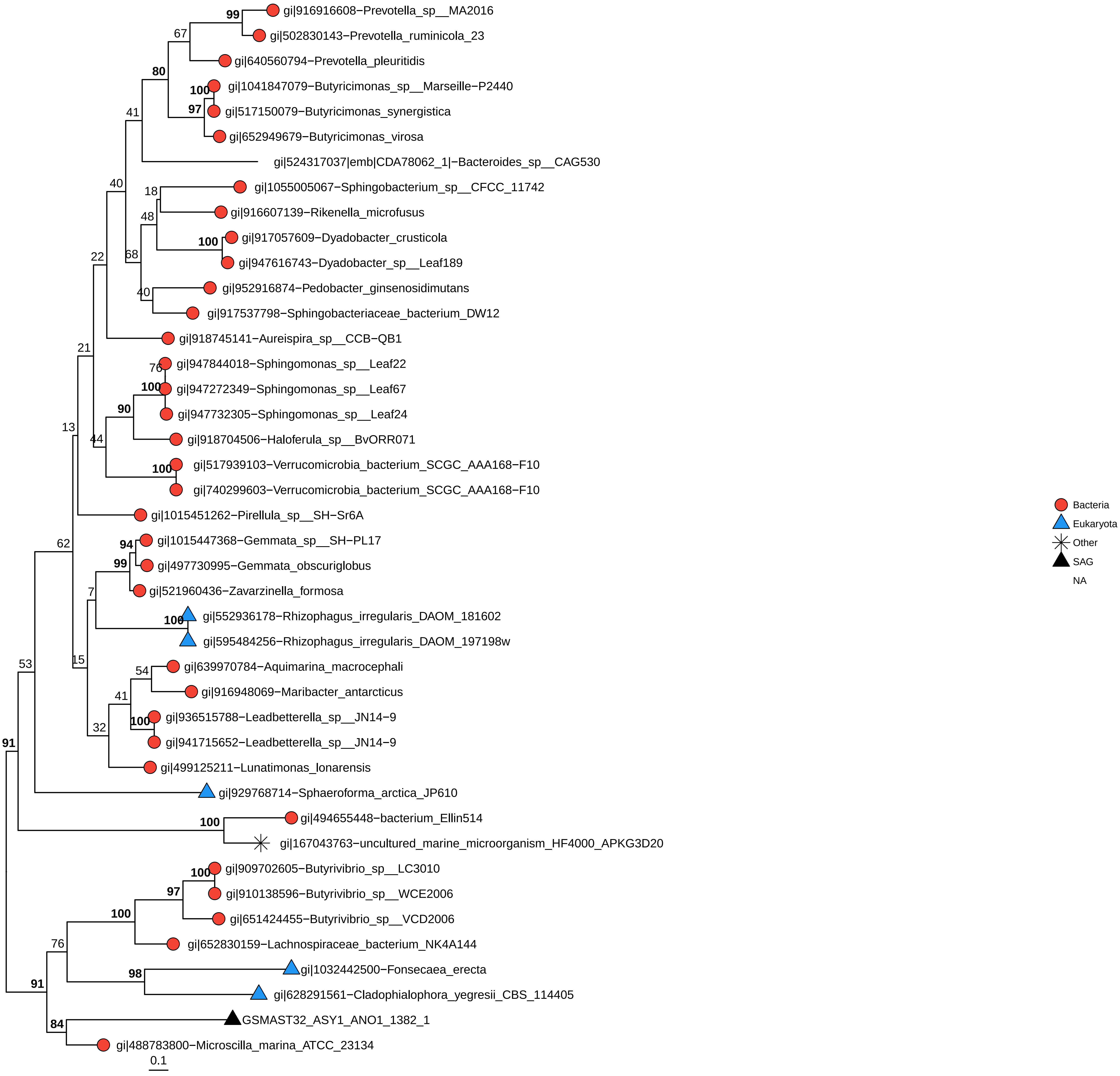
gi|916916608−Prevotella_sp__MA2016
gi|502830143−Prevotella_ruminicola_23
gi|640560794−Prevotella_pleuritidis
gi|1041847079−Butyricimonas_sp__Marseille−P2440
gi|517150079−Butyricimonas_synergistica
gi|652949679−Butyricimonas_virosa
gi|524317037|emb|CDA78062_1|−Bacteroides_sp__CAG530
gi|1055005067−Sphingobacterium_sp__CFCC_11742
gi|916607139−Rikenella_microfusus
gi|917057609−Dyadobacter_crusticola
gi|947616743−Dyadobacter_sp__Leaf189
gi|952916874−Pedobacter_ginsenosidimutans
gi|917537798−Sphingobacteriaceae_bacterium_DW12
gi|918745141−Aureispira_sp__CCB−QB1
gi|947844018−Sphingomonas_sp__Leaf22
gi|947272349−Sphingomonas_sp__Leaf67
gi|947732305−Sphingomonas_sp__Leaf24
gi|918704506−Haloferula_sp__BvORR071
gi|517939103−Verrucomicrobia_bacterium_SCGC_AAA168−F10
gi|740299603−Verrucomicrobia_bacterium_SCGC_AAA168−F10
gi|1015451262−Pirellula_sp__SH−Sr6A
gi|1015447368−Gemmata_sp__SH−PL17
gi|497730995−Gemmata_obscuriglobus
gi|521960436−Zavarzinella_formosa
gi|552936178−Rhizophagus_irregularis_DAOM_181602
gi|595484256−Rhizophagus_irregularis_DAOM_197198w
gi|639970784−Aquimarina_macrocephali
gi|916948069−Maribacter_antarcticus
gi|936515788−Leadbetterella_sp__JN14−9
gi|941715652−Leadbetterella_sp__JN14−9
gi|499125211−Lunatimonas_lonarensis
gi|929768714−Sphaeroforma_arctica_JP610
gi|494655448−bacterium_Ellin514
gi|167043763−uncultured_marine_microorganism_HF4000_APKG3D20
gi|909702605−Butyrivibrio_sp__LC3010
gi|910138596−Butyrivibrio_sp__WCE2006
gi|651424455−Butyrivibrio_sp__VCD2006
gi|652830159−Lachnospiraceae_bacterium_NK4A144
gi|1032442500−Fonsecaea_erecta
gi|628291561−Cladophialophora_yegresii_CBS_114405
GSMAST32_ASY1_ANO1_1382_1
gi|488783800−Microscilla_marina_ATCC_23134
0.1
Bacteria
Eukaryota
Other
SAG
NA

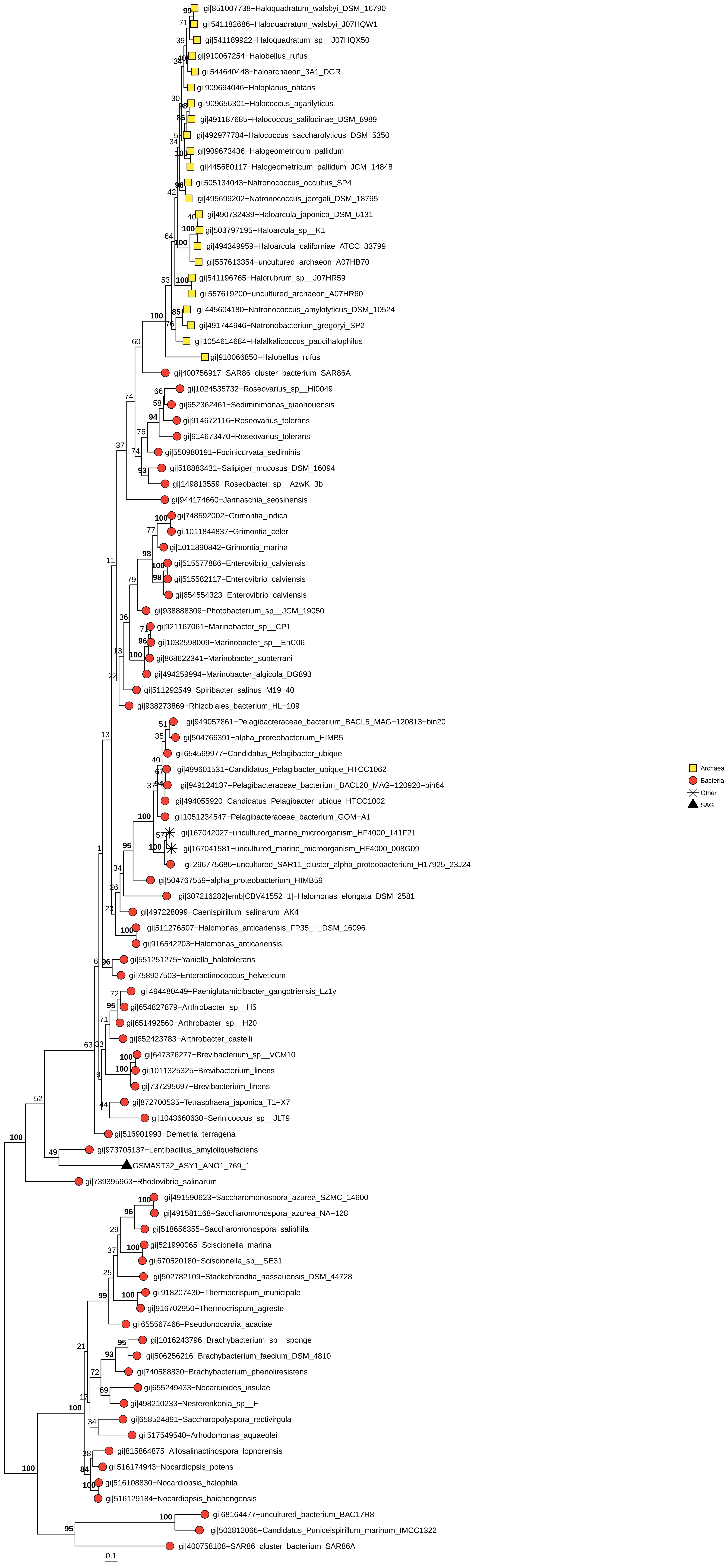
gi|851007738−Haloquadratum_walsbyi_DSM_16790
gi|541182686−Haloquadratum_walsbyi_J07HQW1
gi|541189922−Haloquadratum_sp__J07HQX50
gi|910067254−Halobellus_rufus
gi|544640448−haloarchaeon_3A1_DGR
gi|909694046−Haloplanus_natans
gi|909656301−Halococcus_agarilyticus
gi|491187685−Halococcus_salifodinae_DSM_8989
gi|492977784−Halococcus_saccharolyticus_DSM_5350
gi|909673436−Halogeometricum_pallidum
gi|445680117−Halogeometricum_pallidum_JCM_14848
gi|505134043−Natronococcus_occultus_SP4
gi|495699202−Natronococcus_jeotgali_DSM_18795
gi|490732439−Haloarcula_japonica_DSM_6131
gi|503797195−Haloarcula_sp__K1
gi|494349959−Haloarcula_californiae_ATCC_33799
gi|557613354−uncultured_archaeon_A07HB70
gi|541196765−Halorubrum_sp__J07HR59
gi|557619200−uncultured_archaeon_A07HR60
gi|445604180−Natronococcus_amylolyticus_DSM_10524
gi|491744946−Natronobacterium_gregoryi_SP2
gi|1054614684−Halalkalicoccus_paucihalophilus
gi|910066850−Halobellus_rufus
gi|400756917−SAR86_cluster_bacterium_SAR86A
gi|1024535732−Roseovarius_sp__HI0049
gi|652362461−Sediminimonas_qiaohouensis
gi|914672116−Roseovarius_tolerans
gi|914673470−Roseovarius_tolerans
gi|550980191−Fodinicurvata_sediminis
gi|518883431−Salipiger_mucosus_DSM_16094
gi|149813559−Roseobacter_sp__AzwK−3b
gi|944174660−Jannaschia_seosinensis
gi|748592002−Grimontia_indica
gi|1011844837−Grimontia_celer
gi|1011890842−Grimontia_marina
gi|515577886−Enterovibrio_calviensis
gi|515582117−Enterovibrio_calviensis
gi|654554323−Enterovibrio_calviensis
gi|938888309−Photobacterium_sp__JCM_19050
gi|921167061−Marinobacter_sp__CP1
gi|1032598009−Marinobacter_sp__EhC06
gi|868622341−Marinobacter_subterrani
gi|494259994−Marinobacter_algicola_DG893
gi|511292549−Spiribacter_salinus_M19−40
gi|938273869−Rhizobiales_bacterium_HL−109
gi|949057861−Pelagibacteraceae_bacterium_BACL5_MAG−120813−bin20
gi|504766391−alpha_proteobacterium_HIMB5
gi|654569977−Candidatus_Pelagibacter_ubique
gi|499601531−Candidatus_Pelagibacter_ubique_HTCC1062
gi|949124137−Pelagibacteraceae_bacterium_BACL20_MAG−120920−bin64
gi|494055920−Candidatus_Pelagibacter_ubique_HTCC1002
gi|1051234547−Pelagibacteraceae_bacterium_GOM−A1
gi|167042027−uncultured_marine_microorganism_HF4000_141F21
gi|167041581−uncultured_marine_microorganism_HF4000_008G09
gi|296775686−uncultured_SAR11_cluster_alpha_proteobacterium_H17925_23J24
gi|504767559−alpha_proteobacterium_HIMB59
gi|307216282|emb|CBV41552_1|−Halomonas_elongata_DSM_2581
gi|497228099−Caenispirillum_salinarum_AK4
gi|511276507−Halomonas_anticariensis_FP35_=_DSM_16096
gi|916542203−Halomonas_anticariensis
gi|551251275−Yaniella_halotolerans
gi|758927503−Enteractinococcus_helveticum
gi|494480449−Paeniglutamicibacter_gangotriensis_Lz1y
gi|654827879−Arthrobacter_sp__H5
gi|651492560−Arthrobacter_sp__H20
gi|652423783−Arthrobacter_castelli
gi|647376277−Brevibacterium_sp__VCM10
gi|1011325325−Brevibacterium_linens
gi|737295697−Brevibacterium_linens
gi|872700535−Tetrasphaera_japonica_T1−X7
gi|1043660630−Serinicoccus_sp__JLT9
gi|516901993−Demetria_terragena
gi|973705137−Lentibacillus_amyloliquefaciens
GSMAST32_ASY1_ANO1_769_1
gi|739395963−Rhodovibrio_salinarum
gi|491590623−Saccharomonospora_azurea_SZMC_14600
gi|491581168−Saccharomonospora_azurea_NA−128
gi|518656355−Saccharomonospora_saliphila
gi|521990065−Sciscionella_marina
gi|670520180−Sciscionella_sp__SE31
gi|502782109−Stackebrandtia_nassauensis_DSM_44728
gi|918207430−Thermocrispum_municipale
gi|916702950−Thermocrispum_agreste
gi|655567466−Pseudonocardia_acaciae
gi|1016243796−Brachybacterium_sp__sponge
gi|506256216−Brachybacterium_faecium_DSM_4810
gi|740588830−Brachybacterium_phenoliresistens
gi|655249433−Nocardioides_insulae
gi|498210233−Nesterenkonia_sp__F
gi|658524891−Saccharopolyspora_rectivirgula
gi|517549540−Arhodomonas_aquaeolei
gi|815864875−Allosalinactinospora_lopnorensis
gi|516174943−Nocardiopsis_potens
gi|516108830−Nocardiopsis_halophila
gi|516129184−Nocardiopsis_baichengensis
gi|68164477−uncultured_bacterium_BAC17H8
gi|502812066−Candidatus_Puniceispirillum_marinum_IMCC1322
gi|400758108−SAR86_cluster_bacterium_SAR86A
0.1
Archaea
Bacteria
Other
SAG

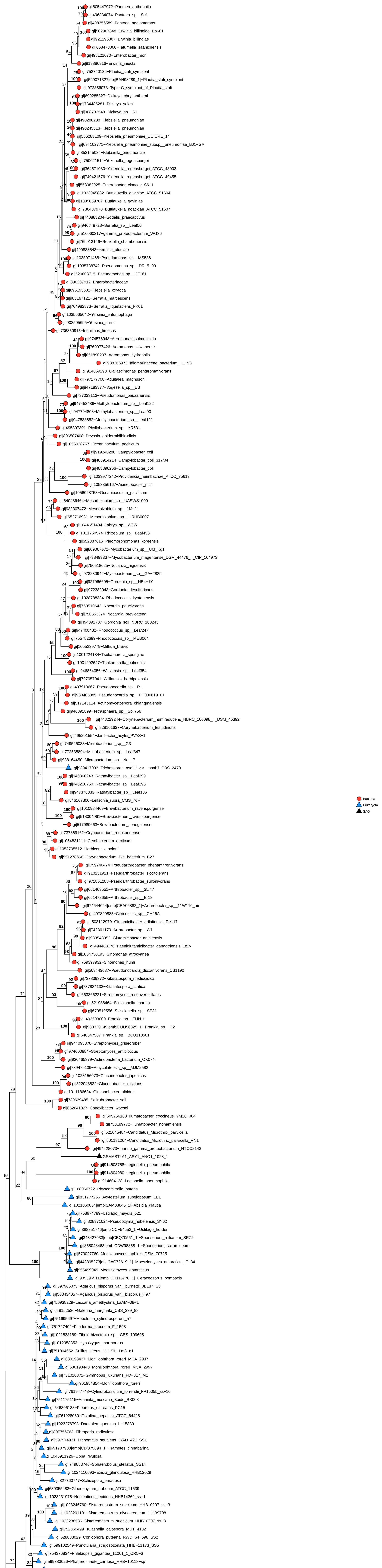
Bacteria
Eukaryota
SAG
0.1

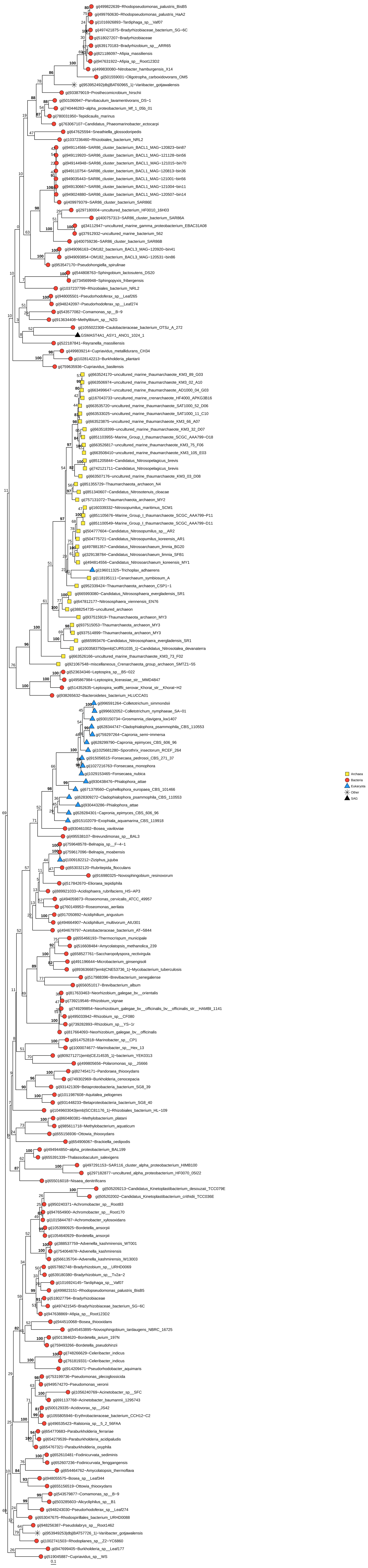
Archaea
Bacteria
Eukaryota
Other
SAG
0.1

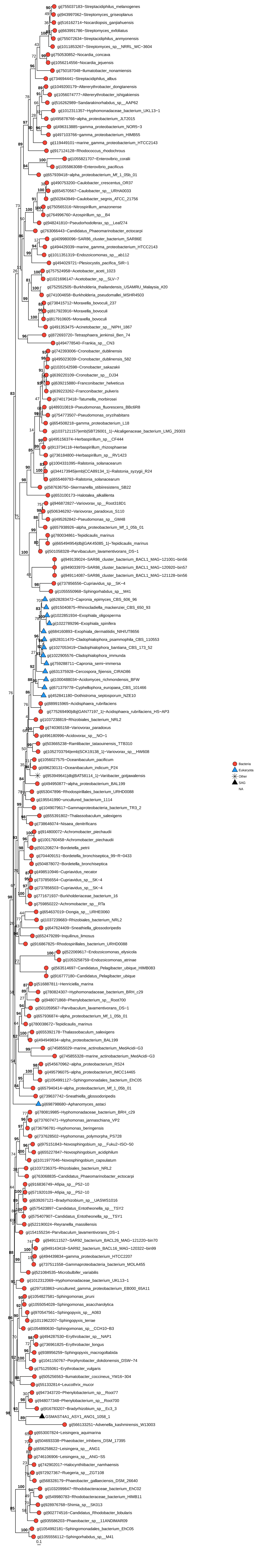

Bacteria
Eukaryota
Other
SAG
NA
0.1

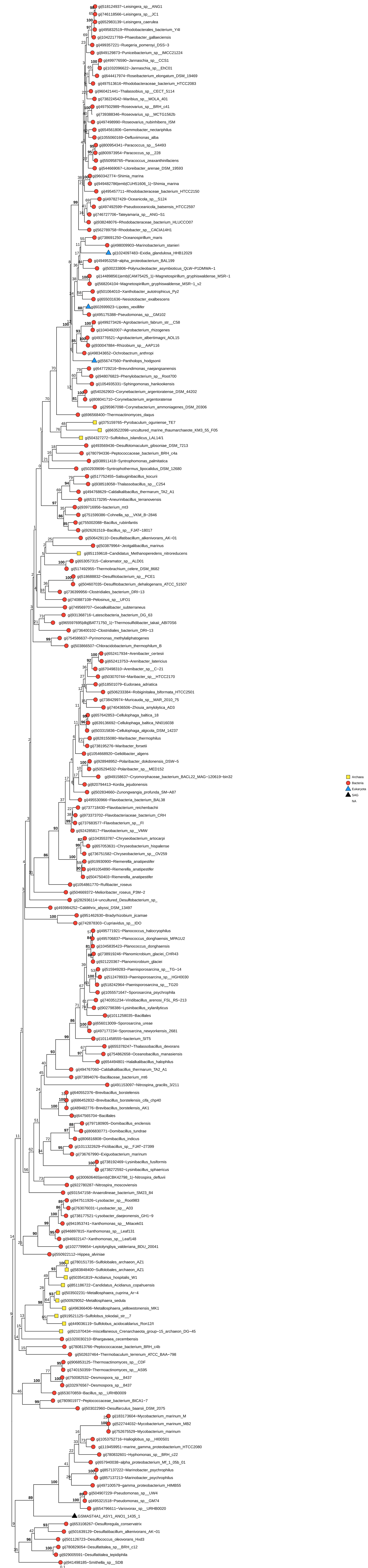

Archaea
Bacteria
Eukaryota
SAG
NA
0.1

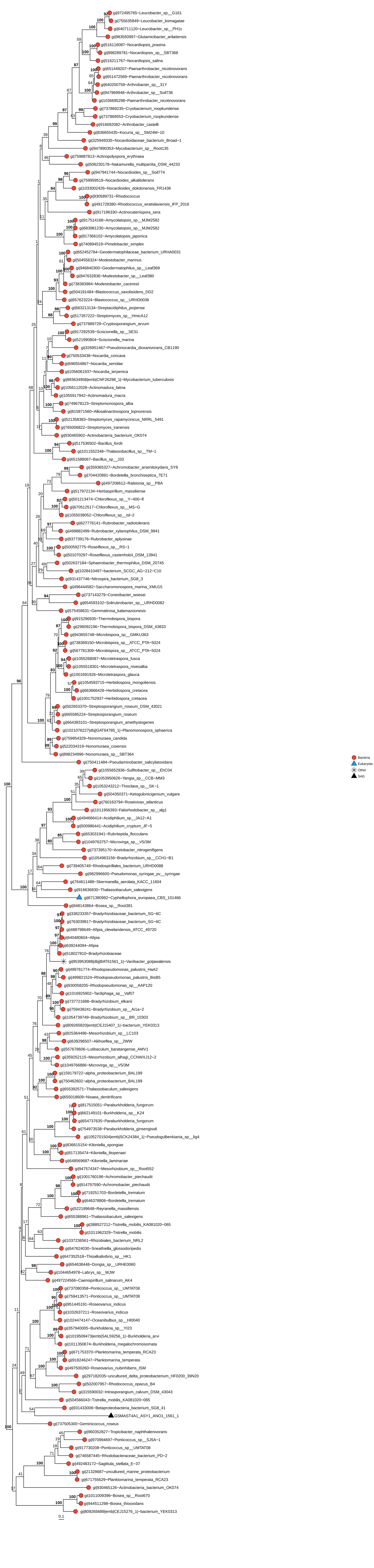
Bacteria
Eukaryota
Other
SAG
0.1

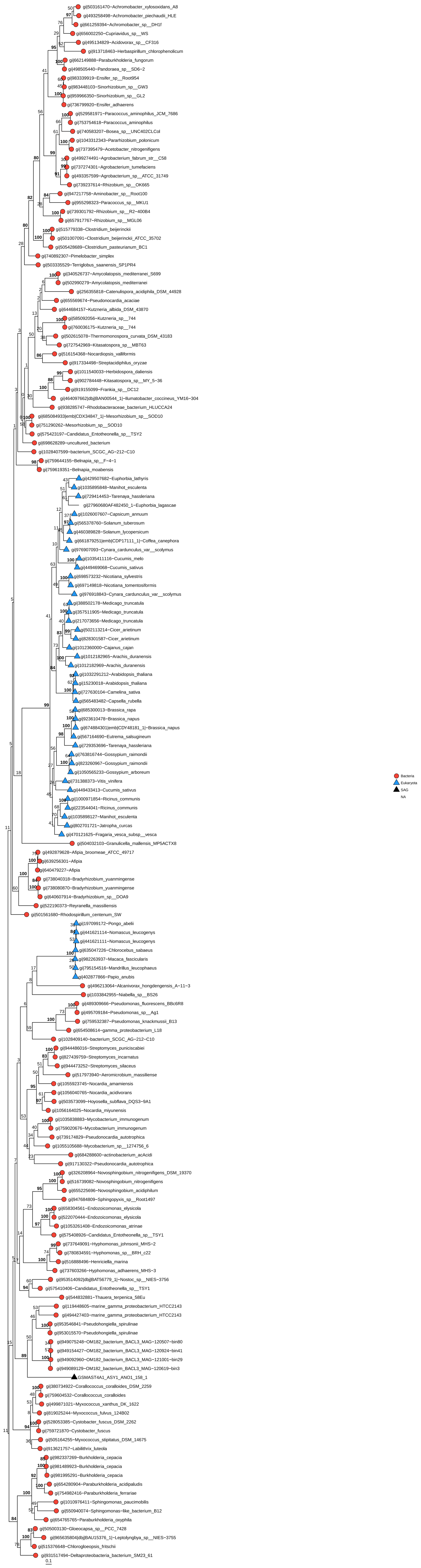
gi|503161470~Achromobacter_xylosoxidans_A8
gi|493258498~Achromobacter_piechaudii_HLE
gi|661259394~Achromobacter_sp__DH1f
gi|656002250~Cupriavidus_sp__WS
gi|495134829~Acidovorax_sp__CF316
gi|913718463~Herbaspirillum_chlorophenolicum
gi|662149888~Paraburkholderia_fungorum
gi|498505440~Pandoraea_sp__SD6-2
gi|983339919~Ensifer_sp__Root954
gi|983448103~Sinorhizobium_sp__GW3
gi|959966350~Sinorhizobium_sp__GL2
gi|736799920~Ensifer_adhaerens
gi|529581971~Paracoccus_aminophilus_JCM_7686
gi|753754618~Paracoccus_aminophilus
gi|740583207~Bosea_sp__UNC402CLCol
gi|1043312343~Pararhizobium_polonicum
gi|737395479~Acetobacter_nitrogenifigens
gi|499274491~Agrobacterium_fabrum_str__C58
gi|737274301~Agrobacterium_tumefaciens
gi|493357599~Agrobacterium_sp__ATCC_31749
gi|739237614~Rhizobium_sp__OK665
gi|947217758~Aminobacter_sp__Root100
gi|955298323~Paracoccus_sp__MKU1
gi|739301792~Rhizobium_sp__R2-400B4
gi|657917767~Rhizobium_sp__MGL06
gi|515779338~Clostridium_beijerinckii
gi|501007091~Clostridium_beijerinckii_ATCC_35702
gi|505428689~Clostridium_pasteurianum_BC1
gi|740892307~Pimelobacter_simplex
gi|503335529~Terriglobus_saanensis_SP1PR4
gi|340526737~Amycolatopsis_mediterranei_S699
gi|502990279~Amycolatopsis_mediterranei
gi|256355818~Catenulispora_acidiphila_DSM_44928
gi|655569674~Pseudonocardia_acaciae
gi|644684157~Kutzneria_albida_DSM_43870
gi|585092056~Kutzneria_sp__744
gi|760036175~Kutzneria_sp__744
gi|502615078~Thermomonospora_curvata_DSM_43183
gi|727542969~Kitasatospora_sp__MBT63
gi|516154368~Nocardiopsis_valliformis
gi|917334498~Streptacidiphilus_oryzae
gi|1011540033~Herbidospora_daliensis
gi|902784448~Kitasatospora_sp__MY_5-36
gi|919155099~Frankia_sp__DC12
gi|464097662|dbj|BAN00544_1|~Ilumatobacter_coccineus_YM16-304
gi|938285747~Rhodobacteraceae_bacterium_HLUCCA24
gi|685084933|emb|CDX34847_1|~Mesorhizobium_sp__SOD10
gi|751290262~Mesorhizobium_sp__SOD10
gi|575423197~Candidatus_Entotheonella_sp__TSY2
gi|698628289~uncultured_bacterium
gi|1028407599~bacterium_SCGC_AG-212-C10
gi|759644155~Belnapia_sp__F-4-1
gi|759619351~Belnapia_moabensis
gi|429507682~Euphorbia_lathyris
gi|1035895848~Manihot_esculenta
gi|729414453~Tarenaya_hassleriana
gi|27960680AF482450_1~Euphorbia_lagascae
gi|1026007607~Capsicum_annuum
gi|565378760~Solanum_tuberosum
gi|460389828~Solanum_lycopersicum
gi|661879251|emb|CDP17111_1|~Coffea_canephora
gi|976907093~Cynara_cardunculus_var__scolymus
gi|1035411116~Cucumis_melo
gi|449469068~Cucumis_sativus
gi|698573232~Nicotiana_sylvestris
gi|697149818~Nicotiana_tomentosiformis
gi|976918843~Cynara_cardunculus_var__scolymus
gi|388502178~Medicago_truncatula
gi|357511905~Medicago_truncatula
gi|217073656~Medicago_truncatula
gi|502113214~Cicer_arietinum
gi|828301587~Cicer_arietinum
gi|1012360000~Cajanus_cajan
gi|1012182965~Arachis_duranensis
gi|1012182969~Arachis_duranensis
gi|1032291212~Arabidopsis_thaliana
gi|15230018~Arabidopsis_thaliana
gi|727630104~Camelina_sativa
gi|565483482~Capsella_rubella
gi|685300013~Brassica_rapa
gi|923610478~Brassica_napus
gi|674884301|emb|CDY48181_1|~Brassica_napus
gi|567164690~Eutrema_salsugineum
gi|729353696~Tarenaya_hassleriana
gi|763816744~Gossypium_raimondii
gi|823260967~Gossypium_raimondii
gi|1050565233~Gossypium_arboreum
gi|731388373~Vitis_vinifera
gi|449433413~Cucumis_sativus
gi|1000971854~Ricinus_communis
gi|223544041~Ricinus_communis
gi|1035898127~Manihot_esculenta
gi|802701721~Jatropha_curcas
gi|470121625~Fragaria_vesca_subsp__vesca
gi|504032103~Granulicella_mallensis_MP5ACTX8
gi|492879628~Afipia_broomeae_ATCC_49717
gi|639256301~Afipia
gi|640479227~Afipia
gi|738040318~Bradyrhizobium_yuanmingense
gi|738080870~Bradyrhizobium_yuanmingense
gi|640607914~Bradyrhizobium_sp__DOA9
gi|522190373~Reyranella_massiliensis
gi|501561680~Rhodospirillum_centenum_SW
gi|197099172~Pongo_abelii
gi|441621114~Nomascus_leucogenys
gi|441621111~Nomascus_leucogenys
gi|635047226~Chlorocebus_sabaeus
gi|982263937~Macaca_fascicularis
gi|795154516~Mandrillus_leucophaeus
gi|402877866~Papio_anubis
gi|496213064~Alcanivorax_hongdengensis_A-11-3
gi|1033842955~Niabella_sp__BS26
gi|489309666~Pseudomonas_fluorescens_BBc6R8
gi|495709184~Pseudomonas_sp__Ag1
gi|759532387~Pseudomonas_knackmussii_B13
gi|654508614~gamma_proteobacterium_L18
gi|1028409140~bacterium_SCGC_AG-212-C10
gi|944486016~Streptomyces_puniciscabiei
gi|827439759~Streptomyces_incarnatus
gi|944473252~Streptomyces_silaceus
gi|517973940~Aeromicrobium_massiliense
gi|1055923745~Nocardia_amamiensis
gi|1056040765~Nocardia_acidivorans
gi|503573099~Hoyosella_subflava_DQS3-9A1
gi|1056164025~Nocardia_miyunensis
gi|1035838883~Mycobacterium_immunogenum
gi|759020676~Mycobacterium_immunogenum
gi|739174829~Pseudonocardia_autotrophica
gi|1055105688~Mycobacterium_sp__1274756_6
gi|684288600~actinobacterium_acAcidi
gi|917130322~Pseudonocardia_autotrophica
gi|326208964~Novosphingobium_nitrogenifigens_DSM_19370
gi|516739082~Novosphingobium_nitrogenifigens
gi|655225696~Novosphingobium_acidiphilum
gi|947684809~Sphingopyxis_sp__Root1497
gi|658304561~Endozoicomonas_elysicola
gi|522070444~Endozoicomonas_elysicola
gi|1053261408~Endozoicomonas_atrinae
gi|575408926~Candidatus_Entotheonella_sp__TSY1
gi|737649091~Hyphomonas_johnsonii_MHS-2
gi|780834591~Hyphomonas_sp__BRH_c22
gi|516888496~Henriciella_marina
gi|737603266~Hyphomonas_adhaerens_MHS-3
gi|953514092|dbj|BAT56779_1|~Nostoc_sp__NIES-3756
gi|575410406~Candidatus_Entotheonella_sp__TSY1
gi|544832881~Thauera_terpenica_58Eu
gi|119448605~marine_gamma_proteobacterium_HTCC2143
gi|494427403~marine_gamma_proteobacterium_HTCC2143
gi|953546841~Pseudohongiella_spirulinae
gi|953015570~Pseudohongiella_spirulinae
gi|949075248~OM182_bacterium_BACL3_MAG-120507-bin80
gi|949154427~OM182_bacterium_BACL3_MAG-120924-bin41
gi|949092960~OM182_bacterium_BACL3_MAG-121001-bin29
gi|949089129~OM182_bacterium_BACL3_MAG-120619-bin3
GSMAST4A1_ASY1_ANO1_158_1
gi|380734922~Corallococcus_coralloides_DSM_2259
gi|759604532~Corallococcus_coralloides
gi|499871021~Myxococcus_xanthus_DK_1622
gi|819025244~Myxococcus_fulvus_124B02
gi|528053385~Cystobacter_fuscus_DSM_2262
gi|759721870~Cystobacter_fuscus
gi|505164255~Myxococcus_stipitatus_DSM_14675
gi|913621757~Labilithrix_luteola
gi|982337269~Burkholderia_cepacia
gi|981489923~Burkholderia_cepacia
gi|981995291~Burkholderia_cepacia
gi|654280904~Paraburkholderia_acidipaludis
gi|754982416~Paraburkholderia_ferrariae
gi|1010976411~Sphingomonas_paucimobilis
gi|550940074~Sphingomonas-like_bacterium_B12
gi|654765765~Paraburkholderia_oxyphila
gi|505003130~Gloeocapsa_sp__PCC_7428
gi|965635804|dbj|BAU15376_1|~Leptolyngbya_sp__NIES-3755
gi|515376648~Chlorogloeopsis_fritschii
gi|931517494~Deltaproteobacteria_bacterium_SM23_61
0.1
Bacteria
Eukaryota
SAG
NA

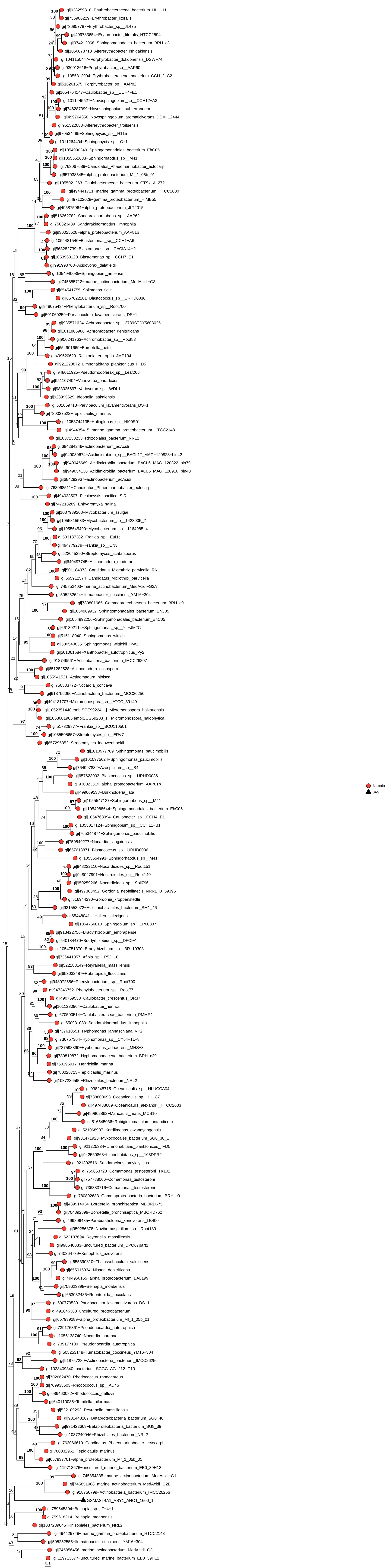

Bacteria
SAG
0.1

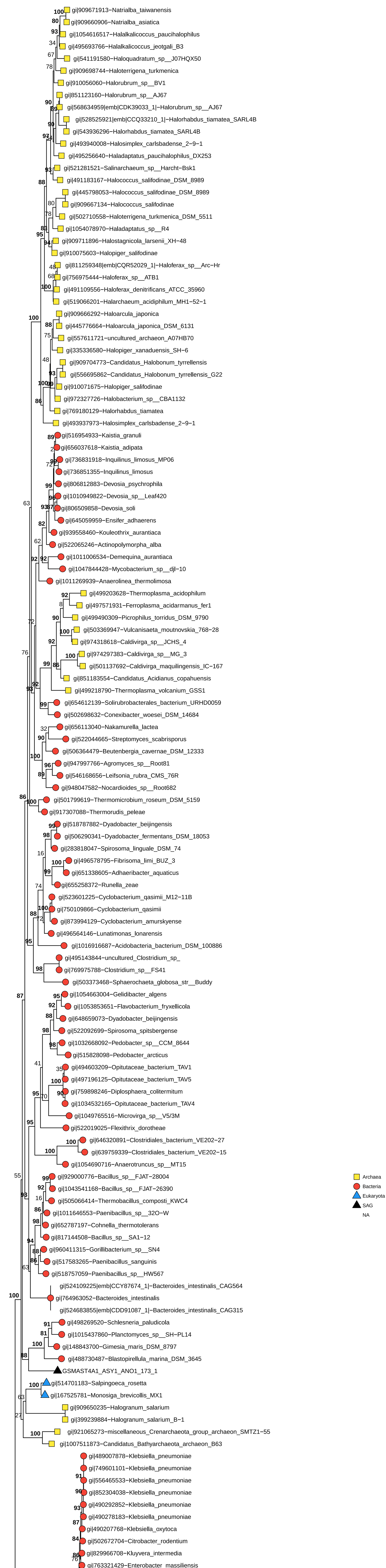
Archaea
Bacteria
Eukaryota
SAG
NA
0.1

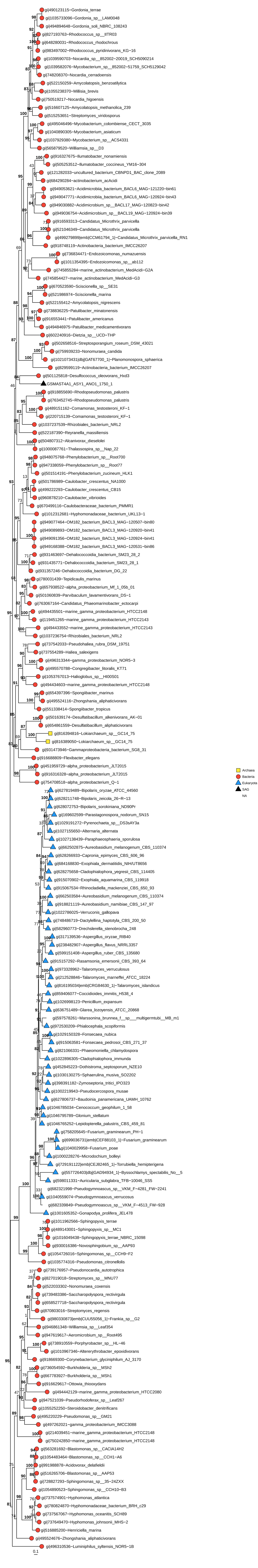
Archaea
Bacteria
Eukaryota
SAG
NA
0.1

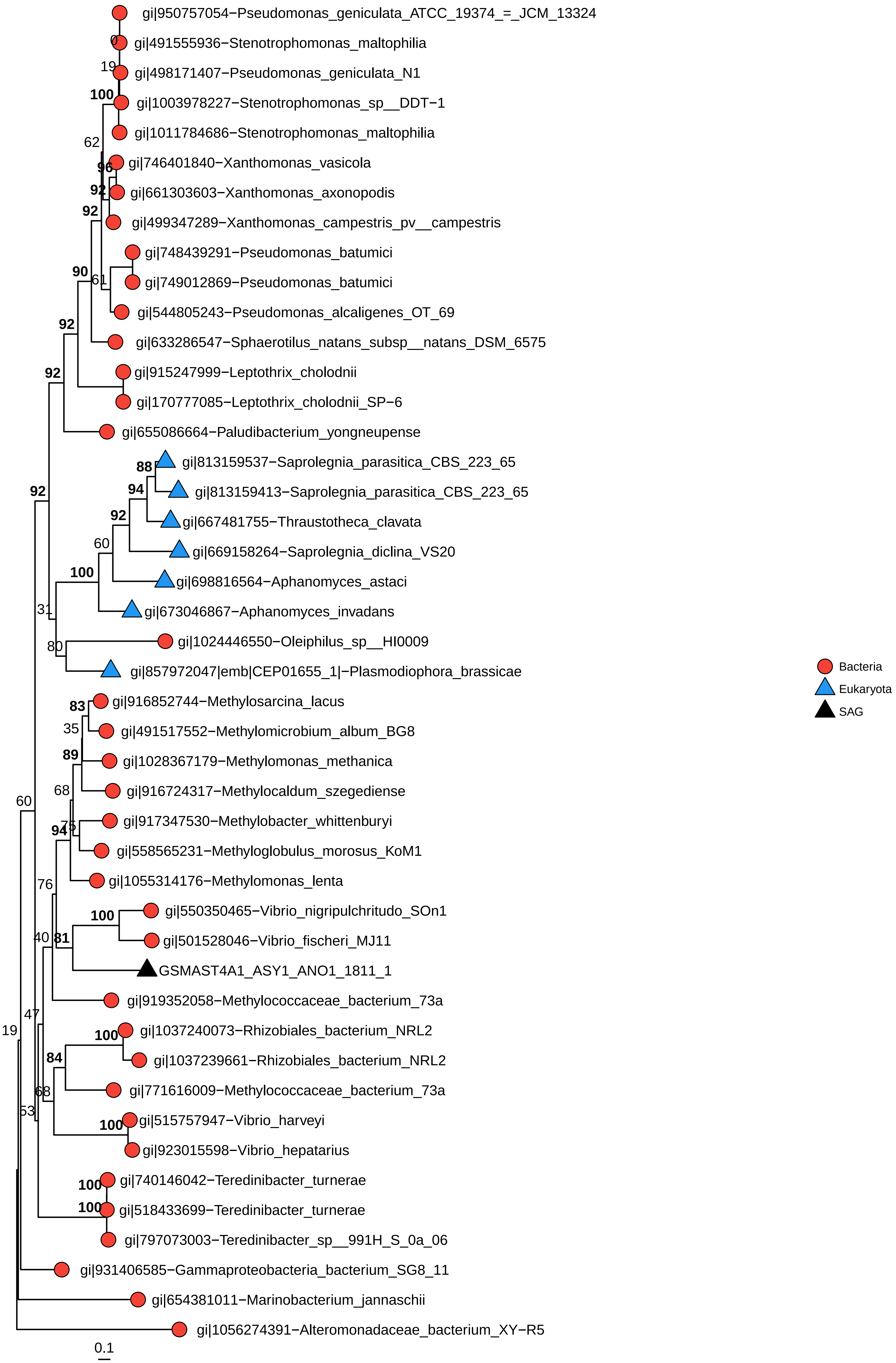

gi|950757054−Pseudomonas_geniculata_ATCC_19374_=_JCM_13324
gi|491555936−Stenotrophomonas_maltophilia
gi|498171407−Pseudomonas_geniculata_N1
gi|1003978227−Stenotrophomonas_sp__DDT−1
gi|1011784686−Stenotrophomonas_maltophilia
gi|746401840−Xanthomonas_vasicola
gi|661303603−Xanthomonas_axonopodis
gi|499347289−Xanthomonas_campestris_pv__campestris
gi|748439291−Pseudomonas_batumici
gi|749012869−Pseudomonas_batumici
gi|544805243−Pseudomonas_alcaligenes_OT_69
gi|633286547−Sphaerotilus_natans_subsp__natans_DSM_6575
gi|915247999−Leptothrix_cholodnii
gi|170777085−Leptothrix_cholodnii_SP−6
gi|655086664−Paludibacterium_yongneupense
gi|813159537−Saprolegnia_parasitica_CBS_223_65
gi|813159413−Saprolegnia_parasitica_CBS_223_65
gi|667481755−Thraustotheca_clavata
gi|669158264−Saprolegnia_diclina_VS20
gi|698816564−Aphanomyces_astaci
gi|673046867−Aphanomyces_invadans
gi|1024446550−Oleiphilus_sp__HI0009
gi|857972047|emb|CEP01655_1|−Plasmodiophora_brassicae
gi|916852744−Methylosarcina_lacus
gi|491517552−Methylomicrobium_album_BG8
gi|1028367179−Methylomonas_methanica
gi|916724317−Methylocaldum_szegediense
gi|917347530−Methylobacter_whittenburyi
gi|558565231−Methyloglobulus_morosus_KoM1
gi|1055314176−Methylomonas_lenta
gi|550350465−Vibrio_nigripulchritudo_SOn1
gi|501528046−Vibrio_fischeri_MJ11
GSMAST4A1_ASY1_ANO1_1811_1
gi|919352058−Methylococcaceae_bacterium_73a
gi|1037240073−Rhizobiales_bacterium_NRL2
gi|1037239661−Rhizobiales_bacterium_NRL2
gi|771616009−Methylococcaceae_bacterium_73a
gi|515757947−Vibrio_harveyi
gi|923015598−Vibrio_hepatarius
gi|740146042−Teredinibacter_turnerae
gi|518433699−Teredinibacter_turnerae
gi|797073003−Teredinibacter_sp__991H_S_0a_06
gi|931406585−Gammaproteobacteria_bacterium_SG8_11
gi|654381011−Marinobacterium_jannaschii
gi|1056274391−Alteromonadaceae_bacterium_XY−R5
0.1
Bacteria
Eukaryota
SAG

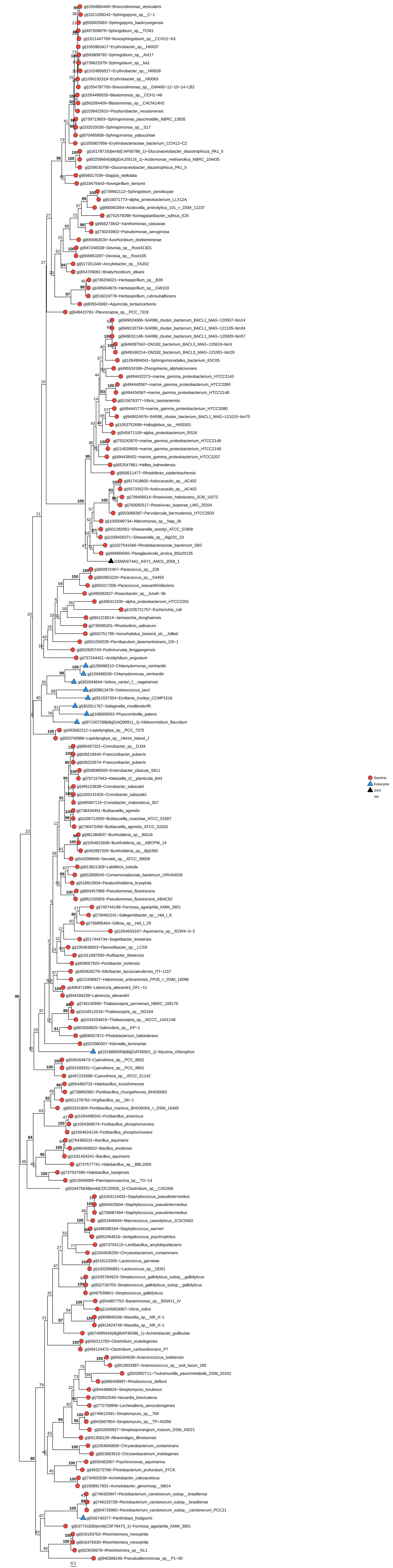
Bacteria
Eukaryota
SAG
NA
0.1

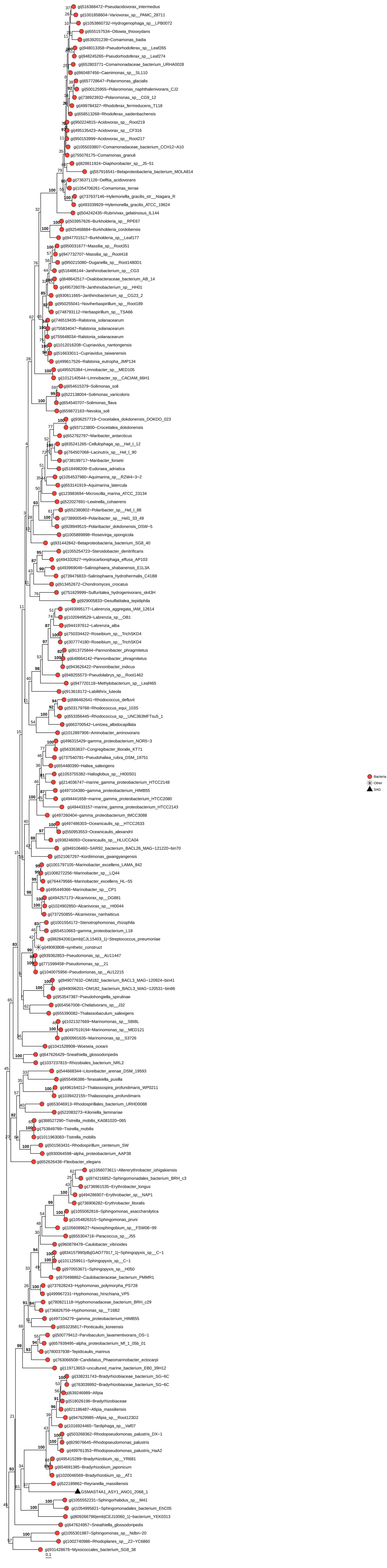

gi|516368472~Pseudacidovorax_intermedius
gi|1001858604~Variovorax_sp__PAMC_28711
gi|1053860732~Hydrogenophaga_sp__LPB0072
gi|655157534~Ottowia_thiooxydans
gi|639201239~Comamonas_badia
gi|948013358~Pseudorhodoferax_sp__Leaf265
gi|948245265~Pseudorhodoferax_sp__Leaf274
gi|652803771~Comamonadaceae_bacterium_URHA0028
gi|860487456~Caenimonas_sp__SL110
gi|657728647~Polaromonas_glacialis
gi|500125955~Polaromonas_naphthalenivorans_CJ2
gi|738923932~Polaromonas_sp__CG9_12
gi|499784327~Rhodoferax_ferrireducens_T118
gi|658513268~Rhodoferax_saidenbachensis
gi|950224815~Acidovorax_sp__Root219
gi|495135423~Acidovorax_sp__CF316
gi|950153999~Acidovorax_sp__Root217
gi|1055033807~Comamonadaceae_bacterium_CCH12~A10
gi|755076175~Comamonas_granuli
gi|829811924~Diaphorobacter_sp__J5~51
gi|557916541~Betaproteobacteria_bacterium_MOLA814
gi|736371126~Delftia_acidovorans
gi|754706261~Comamonas_terrae
gi|737637146~Hylemonella_gracilis_str__Niagara_R
gi|493339929~Hylemonella_gracilis_ATCC_19624
gi|504242435~Rubrivivax_gelatinosus_IL144
gi|503957626~Burkholderia_sp__RPE67
gi|925468884~Burkholderia_cordobensis
gi|947701517~Burkholderia_sp__Leaf177
gi|950031677~Massilia_sp__Root351
gi|947732707~Massilia_sp__Root418
gi|950215080~Duganella_sp__Root1480D1
gi|516486144~Janthinobacterium_sp__CG3
gi|648642517~Oxalobacteraceae_bacterium_AB_14
gi|495726078~Janthinobacterium_sp__HH01
gi|930611665~Janthinobacterium_sp__CG23_2
gi|950255041~Noviherbaspirillum_sp__Root189
gi|748793112~Herbaspirillum_sp__TSA66
gi|746519435~Ralstonia_solanacearum
gi|755834047~Ralstonia_solanacearum
gi|755648034~Ralstonia_solanacearum
gi|1012016208~Cupriavidus_nantongensis
gi|516633011~Cupriavidus_taiwanensis
gi|499617526~Ralstonia_eutropha_JMP134
gi|495525384~Limnobacter_sp__MED105
gi|1012140544~Limnobacter_sp__CACIAM_66H1
gi|654615379~Solimonas_soli
gi|522138004~Solimonas_variicoloris
gi|654540707~Solimonas_flava
gi|659872163~Nevskia_soli
gi|936257719~Croceitalea_dokdonensis_DOKDO_023
gi|937123800~Croceitalea_dokdonensis
gi|652762797~Maribacter_antarcticus
gi|835241265~Cellulophaga_sp__Hel_I_12
gi|764507068~Lacinutrix_sp__Hel_I_90
gi|738199717~Maribacter_forsetii
gi|518498209~Eudoraea_adriatica
gi|1054537980~Aquimarina_sp__RZW4~3~2
gi|653141919~Aquimarina_latercula
gi|123983694~Microscilla_marina_ATCC_23134
gi|522027691~Lewinella_cohaerens
gi|652380802~Polaribacter_sp__Hel_I_88
gi|738900549~Polaribacter_sp__Hel1_33_49
gi|928949515~Polaribacter_dokdonensis_DSW~5
gi|1005889898~Roseivirga_spongicola
gi|931442842~Betaproteobacteria_bacterium_SG8_40
gi|1055254723~Steroidobacter_denitrificans
gi|494332627~Hydrocarboniphaga_effusa_AP103
gi|493969046~Salinisphaera_shabanensis_E1L3A
gi|739476833~Salinisphaera_hydrothermalis_C41B8
gi|913452672~Chondromyces_crocatus
gi|751629999~Sulfuritalea_hydrogenivorans_sk43H
gi|929005833~Desulfatitalea_tepidiphila
gi|493995177~Labrenzia_aggregata_IAM_12614
gi|1020949529~Labrenzia_sp__OB1
gi|944197612~Labrenzia_alba
gi|750334422~Roseibium_sp__TrichSKD4
gi|307774180~Roseibium_sp__TrichSKD4
gi|913725844~Pannonibacter_phragmitetus
gi|648664142~Pannonibacter_phragmitetus
gi|943626422~Pannonibacter_indicus
gi|948255573~Pseudolabrys_sp__Root1462
gi|947720118~Methylobacterium_sp__Leaf465
gi|913618172~Labilithrix_luteola
gi|686462641~Rhodococcus_defluvii
gi|503179768~Rhodococcus_equi_103S
gi|653356445~Rhodococcus_sp__UNC363MFTsu5_1
gi|663700542~Lentzea_albidocapillata
gi|1012897906~Aminobacter_aminovorans
gi|496315429~gamma_proteobacterium_NOR5~3
gi|563353637~Congregibacter_litoralis_KT71
gi|737540781~Pseudohaliea_rubra_DSM_19751
gi|654480390~Haliea_salexigens
gi|1053755382~Halioglobus_sp__HI00S01
gi|214036747~marine_gamma_proteobacterium_HTCC2148
gi|497104380~gamma_proteobacterium_HIMB55
gi|494441658~marine_gamma_proteobacterium_HTCC2080
gi|494433157~marine_gamma_proteobacterium_HTCC2143
gi|497260404~gamma_proteobacterium_IMCC3088
gi|497486303~Oceanicaulis_sp__HTCC2633
gi|550953553~Oceanicaulis_alexandrii
gi|938246093~Oceanicaulis_sp__HLUCCA04
gi|949106460~SAR92_bacterium_BACL26_MAG~121220~bin70
gi|521067297~Kordiimonas_gwangyangensis
gi|1001797105~Marinobacter_excellens_LAMA_842
gi|1008272256~Marinobacter_sp__LQ44
gi|764479566~Marinobacter_excellens_HL~55
gi|495449366~Marinobacter_sp__CP1
gi|494257173~Alcanivorax_sp__DG881
gi|1024902850~Alcanivorax_sp__HI0044
gi|737250855~Alcanivorax_nanhaiticus
gi|1001554172~Stenotrophomonas_rhizophila
gi|654510663~gamma_proteobacterium_L18
gi|882842061|emb|CJL15403_1|~Streptococcus_pneumoniae
gi|49083808~synthetic_construct
gi|939362853~Pseudomonas_sp__AU11447
gi|771599458~Pseudomonas_sp__21
gi|1040075956~Pseudomonas_sp__AU12215
gi|949077632~OM182_bacterium_BACL3_MAG~120924~bin41
gi|949096201~OM182_bacterium_BACL3_MAG~120531~bin86
gi|953547387~Pseudohongiella_spirulinae
gi|654567008~Chelativorans_sp__J32
gi|655390082~Thalassobaculum_salexigens
gi|1021327669~Marinomonas_sp__SBI8L
gi|497519194~Marinomonas_sp__MED121
gi|800991635~Marinomonas_sp__S3726
gi|1041528908~Woeseia_oceani
gi|647626429~Sneathiella_glossodoripedis
gi|1037237815~Rhizobiales_bacterium_NRL2
gi|544668344~Litoreibacter_arenae_DSM_19593
gi|655496386~Terasakiella_pusilla
gi|496164012~Thalassospira_profundimaris_WP0211
gi|1039422155~Thalassospira_profundimaris
gi|653046913~Rhodospirillales_bacterium_URHD0088
gi|522083273~Kiloniella_laminariae
gi|388527290~Tistrella_mobilis_KA081020~065
gi|753849789~Tistrella_mobilis
gi|1011963083~Tistrella_mobilis
gi|501563431~Rhodospirillum_centenum_SW
gi|930064598~alpha_proteobacterium_AAP38
gi|652626438~Flexibacter_elegans
gi|1056073611~Altererythrobacter_ishigakiensis
gi|974216852~Sphingomonadales_bacterium_BRH_c3
gi|736961535~Erythrobacter_longus
gi|494286907~Erythrobacter_sp__NAP1
gi|736906282~Erythrobacter_litoralis
gi|1055062816~Sphingomonas_asaccharolytica
gi|1054826315~Sphingomonas_pruni
gi|1056089627~Novosphingobium_sp__FSW06~99
gi|655304716~Paracoccus_sp__J55
gi|960878478~Caulobacter_vibrioides
gi|834157990|dbj|GAO77917_1|~Sphingopyxis_sp__C~1
gi|1011259911~Sphingopyxis_sp__C~1
gi|970553671~Sphingopyxis_sp__H050
gi|670498862~Caulobacteraceae_bacterium_PMMR1
gi|737628243~Hyphomonas_polymorpha_PS728
gi|499967231~Hyphomonas_hirschiana_VP5
gi|780821118~Hyphomonadaceae_bacterium_BRH_c29
gi|736826759~Hyphomonas_sp__T16B2
gi|497104279~gamma_proteobacterium_HIMB55
gi|653235817~Ponticaulis_koreensis
gi|500779412~Parvibaculum_lavamentivorans_DS~1
gi|657939495~alpha_proteobacterium_Mf_1_05b_01
gi|780037938~Tepidicaulis_marinus
gi|763066508~Candidatus_Phaeomarinobacter_ectocarpi
gi|119713653~uncultured_marine_bacterium_EB0_39H12
gi|338231743~Bradyrhizobiaceae_bacterium_SG~6C
gi|763039992~Bradyrhizobiaceae_bacterium_SG~6C
gi|639246989~Afipia
gi|518026196~Bradyrhizobiaceae
gi|821186487~Afipia_massiliensis
gi|947629985~Afipia_sp__Root123D2
gi|1016924465~Tardiphaga_sp__Vaf07
gi|503268362~Rhodopseudomonas_palustris_DX~1
gi|829076645~Rhodopseudomonas_palustris
gi|499761353~Rhodopseudomonas_palustris_HaA2
gi|495415289~Bradyrhizobium_sp__YR681
gi|654691385~Bradyrhizobium_japonicum
gi|1020046569~Bradyrhizobium_sp__AT1
gi|622189862~Reyranella_massiliensis
GSMAST4A1_ASY1_ANO1_2068_1
gi|1055552231~Sphingorhabdus_sp__M41
gi|1054995821~Sphingomonadales_bacterium_EhC05
gi|809266796|emb|CEJ10060_1|~bacterium_YEK0313
gi|647624957~Sneathiella_glossodoripedis
gi|1055301987~Sphingomonas_sp__Ndbn~20
gi|1002740986~Rhodoplanes_sp__Z2~YC6860
gi|931428676~Myxococcales_bacterium_SG8_38
0.1
Bacteria
Other
SAG

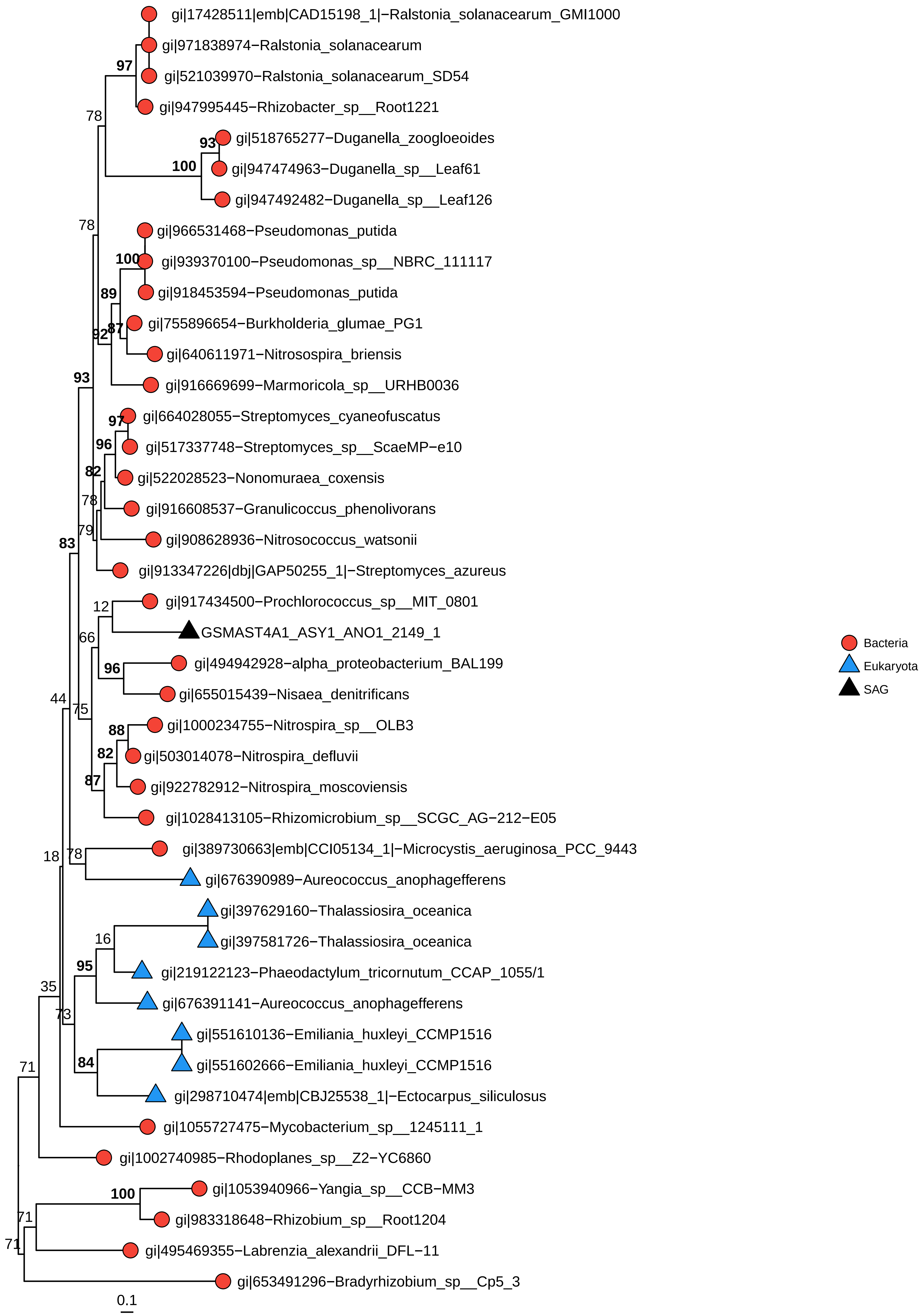

gi|17428511|emb|CAD15198_1|−Ralstonia_solanacearum_GMI1000
gi|971838974−Ralstonia_solanacearum
gi|521039970−Ralstonia_solanacearum_SD54
gi|947995445−Rhizobacter_sp__Root1221
gi|518765277−Duganella_zoogloeoides
gi|947474963−Duganella_sp__Leaf61
gi|947492482−Duganella_sp__Leaf126
gi|966531468−Pseudomonas_putida
gi|939370100−Pseudomonas_sp__NBRC_111117
gi|918453594−Pseudomonas_putida
gi|755896654−Burkholderia_glumae_PG1
gi|640611971−Nitrosospira_briensis
gi|916669699−Marmoricola_sp__URHB0036
gi|664028055−Streptomyces_cyaneofuscatus
gi|517337748−Streptomyces_sp__ScaeMP−e10
gi|522028523−Nonomuraea_coxensis
gi|916608537−Granulicoccus_phenolivorans
gi|908628936−Nitrosococcus_watsonii
gi|913347226|dbj|GAP50255_1|−Streptomyces_azureus
gi|917434500−Prochlorococcus_sp__MIT_0801
GSMAST4A1_ASY1_ANO1_2149_1
gi|494942928−alpha_proteobacterium_BAL199
gi|655015439−Nisaea_denitrificans
gi|1000234755−Nitrospira_sp__OLB3
gi|503014078−Nitrospira_defluvii
gi|922782912−Nitrospira_moscoviensis
gi|1028413105−Rhizomicrobium_sp__SCGC_AG−212−E05
gi|389730663|emb|CCI05134_1|−Microcystis_aeruginosa_PCC_9443
gi|676390989−Aureococcus_anophagefferens
gi|397629160−Thalassiosira_oceanica
gi|397581726−Thalassiosira_oceanica
gi|219122123−Phaeodactylum_tricornutum_CCAP_1055/1
gi|676391141−Aureococcus_anophagefferens
gi|551610136−Emiliania_huxleyi_CCMP1516
gi|551602666−Emiliania_huxleyi_CCMP1516
gi|298710474|emb|CBJ25538_1|−Ectocarpus_siliculosus
gi|1055727475−Mycobacterium_sp__1245111_1
gi|1002740985−Rhodoplanes_sp__Z2−YC6860
gi|1053940966−Yangia_sp__CCB−MM3
gi|983318648−Rhizobium_sp__Root1204
gi|495469355−Labrenzia_alexandrii_DFL−11
gi|653491296−Bradyrhizobium_sp__Cp5_3
0.1
Bacteria
Eukaryota
SAG

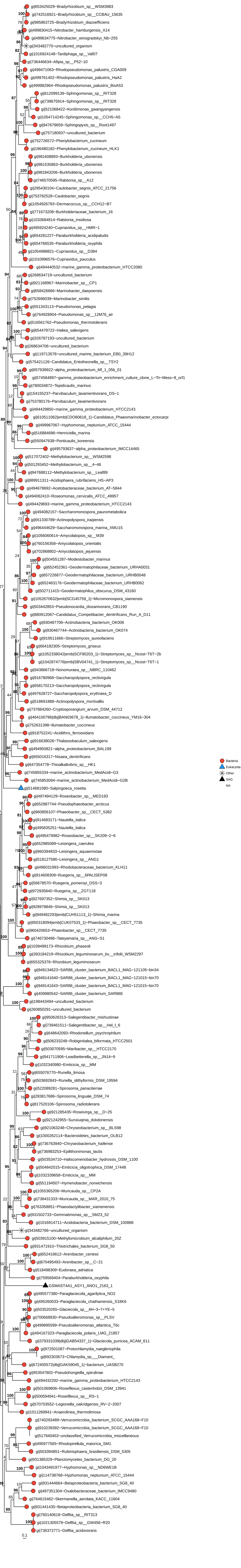

gi|653425029~Bradyrhizobium_sp__WSM3983
gi|742516921~Bradyrhizobium_sp__CCBAU_15635
gi|985863725~Bradyrhizobium_diazoefficiens
gi|499830415~Nitrobacter_hamburgensis_X14
gi|499634775~Nitrobacter_winogradskyi_Nb~255
gi|343482770~uncultured_organism
gi|1016924148~Tardiphaga_sp__Vaf07
gi|736446634~Afipia_sp__P52~10
gi|499471063~Rhodopseudomonas_palustris_CGA009
gi|499761402~Rhodopseudomonas_palustris_HaA2
gi|499982964~Rhodopseudomonas_palustris_BisA53
gi|612099139~Sphingomonas_sp__RIT328
gi|739675914~Sphingomonas_sp__RIT328
gi|521068422~Kordiimonas_gwangyangensis
gi|505714245~Sphingomonas_sp__CCH5~A5
gi|947679659~Sphingopyxis_sp__Root1497
gi|757180937~uncultured_bacterium
gi|752726572~Phenylobacterium_zucineum
gi|196480182~Phenylobacterium_zucineum_HLK1
gi|981608893~Burkholderia_ubonensis
gi|981535863~Burkholderia_ubonensis
gi|981843206~Burkholderia_ubonensis
gi|746570595~Ralstonia_sp__A12
gi|295430104~Caulobacter_segnis_ATCC_21756
gi|753782528~Caulobacter_segnis
gi|1054926793~Dermacoccus_sp__CCH12~B7
gi|771673208~Burkholderiaceae_bacterium_16
gi|1033684814~Ralstonia_insidiosa
gi|495924240~Cupriavidus_sp__HMR~1
gi|654281227~Paraburkholderia_acidipaludis
gi|654766535~Paraburkholderia_oxyphila
gi|1054988821~Cupriavidus_sp__D384
gi|1010996576~Cupriavidus_pauculus
gi|494440532~marine_gamma_proteobacterium_HTCC2080
gi|268634719~uncultured_bacterium
gi|921168967~Marinobacter_sp__CP1
gi|658426666~Marinobacter_daepoensis
gi|752696039~Marinobacter_similis
gi|551343113~Pseudomonas_pelagia
gi|764928904~Pseudomonas_sp__12M76_air
gi|516561762~Pseudomonas_thermotolerans
gi|654478722~Hahella_salexigens
gi|326787193~uncultured_bacterium
gi|268634706~uncultured_bacterium
gi|119713578~uncultured_marine_bacterium_EB0_39H12
gi|575421126~Candidatus_Entotheonella_sp__TSY2
gi|657938922~alpha_proteobacterium_Mf_1_05b_01
gi|574584897~gamma_proteobacterium_enrichment_culture_clone_L~Tri~Mess~8_orf1
gi|780034872~Tepidicaulis_marinus
gi|154155237~Parvibaculum_lavamentivorans_DS~1
gi|753780176~Parvibaculum_lavamentivorans
gi|494429850~marine_gamma_proteobacterium_HTCC2143
gi|810511062|emb|CDO60618_1|~Candidatus_Phaeomarinobacter_ectocarpi
gi|499967067~Hyphomonas_neptunium_ATCC_15444
gi|516884686~Henriciella_marina
gi|550947638~Ponticaulis_koreensis
gi|495793637~alpha_proteobacterium_IMCC14465
gi|517072402~Methylobacterium_sp__WSM2598
gi|501293452~Methylobacterium_sp__4~46
gi|947688112~Methylobacterium_sp__Leaf89
gi|889911311~Acidisphaera_rubrifaciens_HS~AP3
gi|496678692~Acetobacteraceae_bacterium_AT~5844
gi|494062410~Roseomonas_cervicalis_ATCC_49957
gi|494429693~marine_gamma_proteobacterium_HTCC2143
gi|494082157~Saccharomonospora_paurometabolica
gi|651330789~Actinopolyspora_iraqiensis
gi|496444629~Saccharomonospora_marina_XMU15
gi|1056060614~Amycolatopsis_sp__M39
gi|760156358~Amycolatopsis_orientalis
gi|702868802~Amycolatopsis_jejuensis
gi|504551287~Modestobacter_marinus
gi|652452361~Geodermatophilaceae_bacterium_URHA0031
gi|657226877~Geodermatophilaceae_bacterium_URHB0048
gi|652463176~Geodermatophilaceae_bacterium_URHB0062
gi|502711415~Geodermatophilus_obscurus_DSM_43160
gi|1052676632|emb|SCG45759_1|~Micromonospora_siamensis
gi|503442853~Pseudonocardia_dioxanivorans_CB1190
gi|880912067~Candidatus_Competibacter_denitrificans_Run_A_D11
gi|930487706~Actinobacteria_bacterium_OK006
gi|930467744~Actinobacteria_bacterium_OK074
gi|919511666~Streptomyces_aureofaciens
gi|664182305~Streptomyces_griseus
gi|1052338423|emb|SCF90203_1|~Streptomyces_sp__Ncost~T6T~2b
gi|1042874776|emb|SBV04741_1|~Streptomyces_sp__Ncost~T6T~1
gi|943866718~Nonomuraea_sp__NBRC_110462
gi|916780968~Saccharopolyspora_rectivirgula
gi|658170213~Saccharopolyspora_rectivirgula
gi|497628727~Saccharopolyspora_erythraea_D
gi|518691888~Actinopolyspora_mortivallis
gi|737884260~Cryptosporangium_arvum_DSM_44712
gi|464100796|dbj|BAN03678_1|~Ilumatobacter_coccineus_YM16~304
gi|752631398~Ilumatobacter_coccineus
gi|918752241~Acidithrix_ferrooxidans
gi|916638026~Thalassobaculum_salexigens
gi|494950821~alpha_proteobacterium_BAL199
gi|655016317~Nisaea_denitrificans
gi|647354778~Thioalkalivibrio_sp__HK1
gi|745855334~marine_actinobacterium_MedAcidi~G3
gi|745853094~marine_actinobacterium_MedAcidi~G2B
gi|514681580~Salpingoeca_rosetta
gi|497494129~Roseobacter_sp__MED193
gi|652987744~Pseudophaeobacter_arcticus
gi|960856107~Phaeobacter_sp__CECT_5382
gi|914683171~Nautella_italica
gi|495835251~Nautella_italica
gi|495479982~Roseobacter_sp__SK209~2~6
gi|652985089~Leisingera_caerulea
gi|960394833~Leisingera_aquaemixtae
gi|518127586~Leisingera_sp__ANG1
gi|496031993~Rhodobacteraceae_bacterium_KLH11
gi|914608309~Ruegeria_sp__6PALISEP08
gi|56678570~Ruegeria_pomeroyi_DSS~3
gi|972935840~Ruegeria_sp__2GT118
gi|927697352~Shimia_sp__SK013
gi|928978846~Shimia_sp__SK013
gi|494982293|emb|CUH51113_1|~Shimia_marina
gi|950318094|emb|CUK07533_1|~Phaeobacter_sp__CECT_7735
gi|960402653~Phaeobacter_sp__CECT_7735
gi|746730496~Tateyamaria_sp__ANG~S1
gi|1039499173~Rhizobium_phaseoli
gi|393184219~Rhizobium_leguminosarum_bv__trifolii_WSM2297
gi|655325376~Rhizobium_leguminosarum
gi|949134623~SAR86_cluster_bacterium_BACL1_MAG~121105~bin34
gi|949141640~SAR86_cluster_bacterium_BACL1_MAG~121105~bin70
gi|949141643~SAR86_cluster_bacterium_BACL1_MAG~121105~bin70
gi|409980542~SAR86_cluster_bacterium_SAR86E
gi|198443494~uncultured_bacterium
gi|260850291~uncultured_bacterium
gi|950626313~Salegentibacter_mishustinae
gi|739461511~Salegentibacter_sp__Hel_I_6
gi|648642093~Rhodonellum_psychrophilum
gi|506233248~Robiginitalea_biformata_HTCC2501
gi|503070595~Maribacter_sp__HTCC2170
gi|941711906~Leadbetterella_sp__JN14~9
gi|1032340980~Emticicia_sp__MM
gi|655076770~Runella_limosa
gi|503692843~Runella_slithyformis_DSM_19594
gi|522089281~Spirosoma_panaciterrae
gi|283817686~Spirosoma_linguale_DSM_74
gi|817520106~Spirosoma_radiotolerans
gi|921285435~Roseivirga_sp__D~25
gi|921242955~Sunxiuqinia_dokdonensis
gi|921063248~Chryseobacterium_sp__BLS98
gi|1000262114~Bacteroidetes_bacterium_OLB12
gi|736763940~Chryseobacterium_haifense
gi|736983253~Epilithonimonas_lactis
gi|503534710~Haliscomenobacter_hydrossis_DSM_1100
gi|504842015~Emticicia_oligotrophica_DSM_17448
gi|1032339658~Emticicia_sp__MM
gi|551194507~Hymenobacter_norwichensis
gi|1055365206~Muricauda_sp__CP2A
gi|738431333~Muricauda_sp__MAR_2010_75
gi|763358851~Phaeodactylibacter_xiamenensis
gi|931502733~Gemmatimonas_sp__SM23_52
gi|1016941711~Acidobacteria_bacterium_DSM_100886
gi|343482766~uncultured_organism
gi|503915100~Methylomicrobium_alcaliphilum_20Z
gi|931471910~Thiotrichales_bacterium_SG8_50
gi|652416612~Arenibacter_certesii
gi|670495493~Arenibacter_sp__C~21
gi|518498309~Eudoraea_adriatica
gi|759566904~Paraburkholderia_oxyphila
GSMAST4A1_ASY1_ANO1_2163_1
gi|495577380~Paraglaciecola_agarilytica_NO2
gi|495260033~Paraglaciecola_chathamensis_S18K6
gi|503520265~Glaciecola_sp__4H~3~7+YE~5
gi|700668930~Pseudoalteromonas_sp__PLSV
gi|499895599~Pseudoalteromonas_atlantica_T6c
gi|494167323~Paraglaciecola_polaris_LMG_21857
gi|379331039|dbj|GAB54337_1|~Glaciecola_punicea_ACAM_611
gi|972501087~Protochlamydia_naegleriophila
gi|692303673~Chlamydia_sp__Diamant_
gi|672400572|dbj|GAK59045_1|~bacterium_UASB270
gi|953547802~Pseudohongiella_spirulinae
gi|494432292~marine_gamma_proteobacterium_HTCC2143
gi|501069806~Roseiflexus_castenholzii_DSM_13941
gi|500594941~Roseiflexus_sp__RS~1
gi|570703552~Legionella_oakridgensis_RV~2~2007
gi|1011269941~Anaerolinea_thermolimosa
gi|740293489~Verrucomicrobia_bacterium_SCGC_AAA168~F10
gi|910239392~Verrucomicrobia_bacterium_SCGC_AAA168~F10
gi|517940453~unclassified_Verrucomicrobia_miscellaneous
gi|495977565~Rhodopirellula_maiorica_SM1
gi|503394851~Rubinisphaera_brasiliensis_DSM_5305
gi|931385929~Planctomycetes_bacterium_DG_20
gi|1043491977~Hyphomonas_sp__ND6WE1B
gi|114738768~Hyphomonas_neptunium_ATCC_15444
gi|931444664~Betaproteobacteria_bacterium_SG8_40
gi|497351304~Oxalobacteraceae_bacterium_IMCC9480
gi|764615462~Skermanella_aerolata_KACC_11604
gi|931441435~Betaproteobacteria_bacterium_SG8_40
gi|760140618~Delftia_sp__RIT313
gi|1021305579~Delftia_sp__GW456~R20
gi|736372771~Delftia_acidovorans
0.1
Bacteria
Eukaryota
Other
SAG
NA

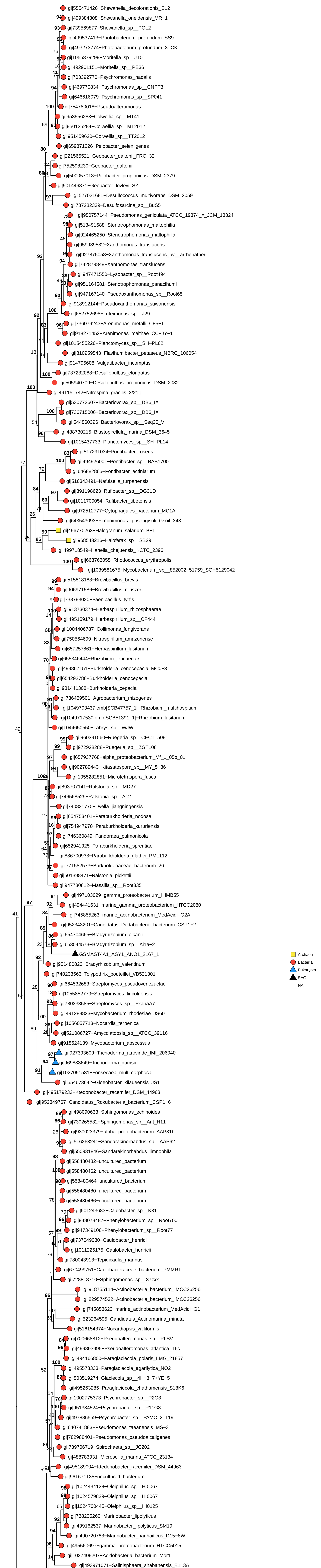

Archaea
Bacteria
Eukaryota
SAG
NA
0.1

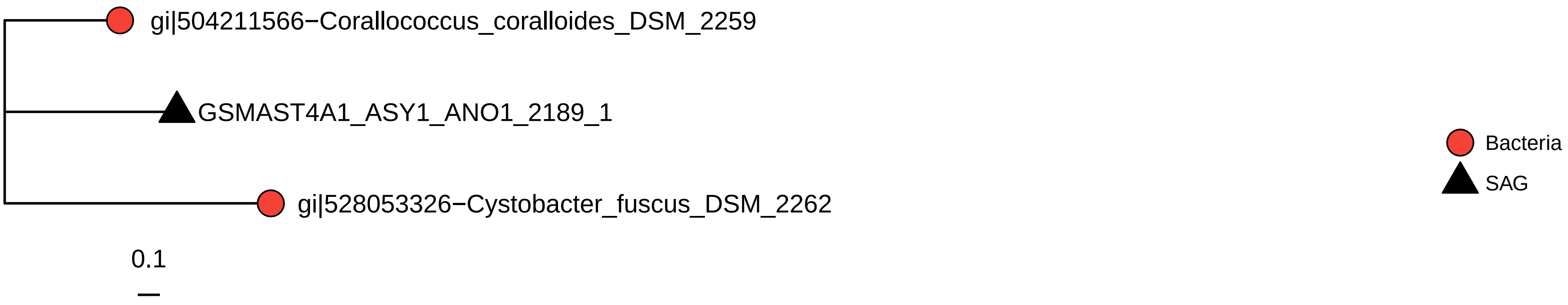
gi|504211566−Corallococcus_coralloides_DSM_2259
GSMAST4A1_ASY1_ANO1_2189_1
gi|528053326−Cystobacter_fuscus_DSM_2262
0.1
Bacteria
SAG

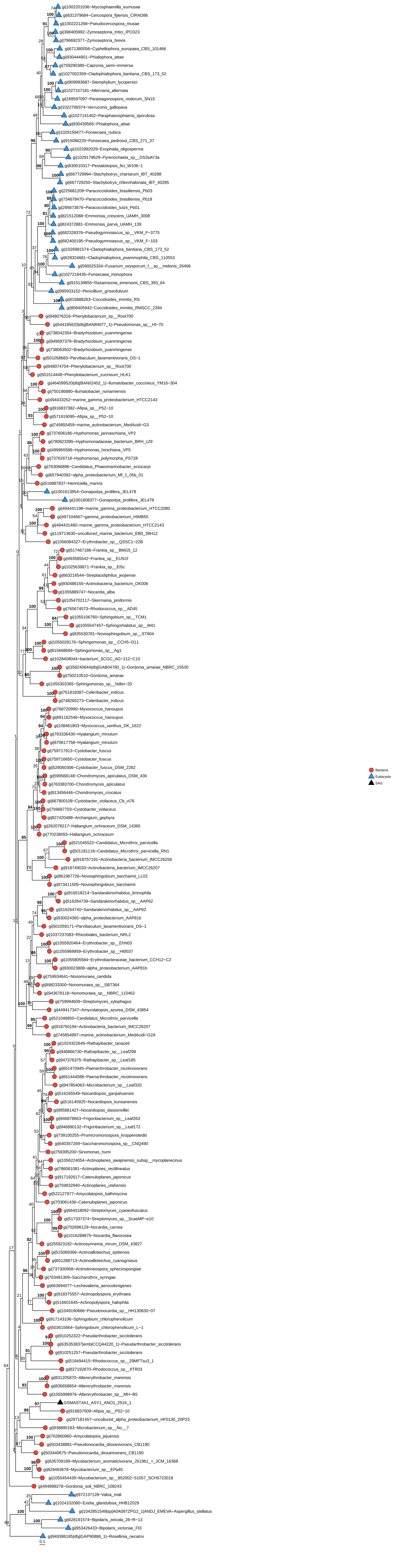

gi|1002201036~Mycosphaerella_eumusae
gi|631379684~Cercospora_fijiensis_CIRAD86
gi|1002221268~Pseudocercospora_musae
gi|398405992~Zymoseptoria_tritici_IPO323
gi|796692377~Zymoseptoria_brevis
gi|671380056~Cyphellophora_europaea_CBS_101466
gi|930444901~Phialophora_attae
gi|759290389~Capronia_semi~immersa
gi|1027002359~Cladophialophora_bantiana_CBS_173_52
gi|909993687~Stemphylium_lycopersici
gi|1027157181~Alternaria_alternata
gi|169597097~Parastagonospora_nodorum_SN15
gi|1022799374~Verruconis_gallopava
gi|1027141402~Paraphaeosphaeria_sporulosa
gi|930439565~Phialophora_attae
gi|1029159477~Fonsecaea_nubica
gi|915086220~Fonsecaea_pedrosoi_CBS_271_37
gi|1022892029~Exophiala_oligosperma
gi|1029179629~Pyrenochaeta_sp__DS3sAY3a
gi|630010317~Pestalotiopsis_fici_W106~1
gi|667729994~Stachybotrys_chartarum_IBT_40288
gi|667729250~Stachybotrys_chlorohalonata_IBT_40285
gi|225681209~Paracoccidioides_brasiliensis_Pb03
gi|734678470~Paracoccidioides_brasiliensis_Pb18
gi|295673676~Paracoccidioides_lutzii_Pb01
gi|821512088~Emmonsia_crescens_UAMH_3008
gi|824372881~Emmonsia_parva_UAMH_139
gi|682328376~Pseudogymnoascus_sp__VKM_F~3775
gi|682400195~Pseudogymnoascus_sp__VKM_F~103
gi|1026981574~Cladophialophora_bantiana_CBS_173_52
gi|628324681~Cladophialophora_psammophila_CBS_110553
gi|590025334~Fusarium_oxysporum_f__sp__melonis_26406
gi|1027218435~Fonsecaea_monophora
gi|915139855~Rasamsonia_emersonii_CBS_393_64
gi|995933152~Penicillium_griseofulvum
gi|815888263~Coccidioides_immitis_RS
gi|859405942~Coccidioides_immitis_RMSCC_2394
gi|948076316~Phenylobacterium_sp__Root700
gi|544185633|dbj|BAN84077_1|~Pseudomonas_sp__HI~70
gi|738042364~Bradyrhizobium_yuanmingense
gi|949597379~Bradyrhizobium_yuanmingense
gi|738063502~Bradyrhizobium_yuanmingense
gi|501058683~Parvibaculum_lavamentivorans_DS~1
gi|948074704~Phenylobacterium_sp__Root700
gi|501514448~Phenylobacterium_zucineum_HLK1
gi|464099520|dbj|BAN02402_1|~Illumatobacter_coccineus_YM16~304
gi|750186880~Illumatobacter_nonamiensis
gi|494433252~marine_gamma_proteobacterium_HTCC2143
gi|916837382~Alfipia_sp__P52~10
gi|571919095~Afipia_sp__P52~10
gi|745855459~marine_actinobacterium_MedAcidi~G3
gi|737606186~Hyphomonas_jannaschiana_VP2
gi|738823395~Hyphomonadaceae_bacterium_BRH_c29
gi|499965586~Hyphomonas_hirschiana_VP5
gi|737626718~Hyphomonas_polymorpha_PS728
gi|763066896~Candidatus_Phaeomarinobacter_ectocarpi
gi|657940392~alpha_proteobacterium_Mf_1_05b_01
gi|516887837~Henriciella_marina
gi|1001613854~Gonapodya_prolifera_JEL478
gi|1001608377~Gonapodya_prolifera_JEL478
gi|494441198~marine_gamma_proteobacterium_HTCC2080
gi|497104667~gamma_proteobacterium_HIMB55
gi|494431480~marine_gamma_proteobacterium_HTCC2143
gi|119713630~uncultured_marine_bacterium_EB0_39H12
gi|1056084327~Erythrobacter_sp__QSSC1~22B
gi|517467186~Frankia_sp__BMG5_12
gi|493585542~Frankia_sp__EUN1f
gi|1025639871~Frankia_sp__Ei5c
gi|663216544~Streptacidiphilus_jeojiense
gi|930486155~Actinobacteria_bacterium_OK006
gi|1055889747~Nocardia_alba
gi|1054702117~Skermania_piniformis
gi|765674573~Rhodococcus_sp__AD45
gi|1055106760~Sphingobium_sp__TCM1
gi|1055547457~Sphingorhabdus_sp__M41
gi|935530781~Novosphingobium_sp__ST904
gi|1055028176~Sphingomonas_sp__CCH5~D11
gi|815668694~Sphingomonas_sp__Ag1
gi|1028408044~bacterium_SCGC_AG~212~C10
gi|358240644|dbj|GAB04780_1|~Gordonia_amarae_NBRC_15530
gi|750210510~Gordonia_amarae
gi|1055303365~Sphingomonas_sp__Ndbn~20
gi|761818387~Celeribacter_indicus
gi|748265273~Celeribacter_indicus
gi|768720990~Myxococcus_hansupus
gi|891162546~Myxococcus_hansupus
gi|108461803~Myxococcus_xanthus_DK_1622
gi|763336430~Hyalangium_minutum
gi|670617758~Hyalangium_minutum
gi|759717913~Cystobacter_fuscus
gi|759716650~Cystobacter_fuscus
gi|528060306~Cystobacter_fuscus_DSM_2262
gi|599568148~Chondromyces_apiculatus_DSM_436
gi|763383700~Chondromyces_apiculatus
gi|913456446~Chondromyces_crocatus
gi|667800108~Cystobacter_violaceus_Cb_vi76
gi|759687703~Cystobacter_violaceus
gi|827420488~Archangium_gephyra
gi|262078217~Haliangium_ochraceum_DSM_14365
gi|770238055~Haliangium_ochraceum
gi|521045522~Candidatus_Microthrix_parvicella
gi|501181118~Candidatus_Microthrix_parvicella_RN1
gi|918757191~Actinobacteria_bacterium_IMCC26256
gi|918749033~Actinobacteria_bacterium_IMCC26207
gi|861967726~Novosphingobium_barchaimii_LL02
gi|973411505~Novosphingobium_barchaimii
gi|916518214~Sandarakinorhabdus_limnophila
gi|516264739~Sandarakinorhabdus_sp__AAP62
gi|516264740~Sandarakinorhabdus_sp__AAP62
gi|930024365~alpha_proteobacterium_AAP81b
gi|501059171~Parvibaculum_lavamentivorans_DS~1
gi|1037237083~Rhizobiales_bacterium_NRL2
gi|1055920464~Erythrobacter_sp__EhN03
gi|1055969959~Erythrobacter_sp__HI0037
gi|1055805584~Erythrobacteraceae_bacterium_CCH12~C2
gi|930023808~alpha_proteobacterium_AAP81b
gi|759934641~Nonomuraea_candida
gi|898233300~Nonomuraea_sp__SBT364
gi|943678118~Nonomuraea_sp__NBRC_110462
gi|759994609~Streptomyces_xylophagus
gi|449417347~Amycolatopsis_azurea_DSM_43854
gi|521046850~Candidatus_Microthrix_parvicella
gi|918750194~Actinobacteria_bacterium_IMCC26207
gi|745854997~marine_actinobacterium_MedAcidi~G2A
gi|1024322649~Rathayibacter_tanaceti
gi|946866730~Rathayibacter_sp__Leaf299
gi|947376375~Rathayibacter_sp__Leaf185
gi|651470945~Paenarthrobacter_nicotinovorans
gi|651444588~Paenarthrobacter_nicotinovorans
gi|947854063~Microbacterium_sp__Leaf320
gi|516165549~Nocardiopsis_ganjiahuensis
gi|516145925~Nocardiopsis_kunsanensis
gi|995881427~Nocardiopsis_dassonvillei
gi|946878663~Frigoribacterium_sp__Leaf263
gi|946890132~Frigoribacterium_sp__Leaf172
gi|739100255~Promicromonospora_kroppenstedtii
gi|640357269~Saccharomonospora_sp__CNQ490
gi|759395200~Sinomonas_humi
gi|1056224054~Actinoplanes_awajinensis_subsp__mycoplanecinus
gi|786061081~Actinoplanes_rectilineatus
gi|917192617~Catenuloplanes_japonicus
gi|759832940~Actinoplanes_utahensis
gi|522127977~Amycolatopsis_balhimycina
gi|703061436~Catenuloplanes_japonicus
gi|664018092~Streptomyces_cyaneofuscatus
gi|517337374~Streptomyces_sp__ScaeMP~e10
gi|702696129~Nocardia_carnea
gi|1016289675~Nocardia_flavorosea
gi|255923182~Actinosynnema_mirum_DSM_43827
gi|515069366~Actinoalloteichus_spitiensis
gi|651288713~Actinoalloteichus_cyanogriseus
gi|737300958~Actinokineospora_spheciospongiae
gi|703481309~Saccharothrix_syringae
gi|663694077~Lechevalieria_aerocolonigenes
gi|918375557~Actinopolyspora_erythraea
gi|516601645~Actinopolyspora_halophila
gi|1049190666~Pseudonocardia_sp__HH130630~07
gi|917143106~Sphingobium_chlorophenolicum
gi|503615664~Sphingobium_chlorophenolicum_L~1
gi|910252322~Pseudarthrobacter_siccitolerans
gi|635353837|emb|CCQ44220_1|~Pseudarthrobacter_siccitolerans
gi|910251257~Pseudarthrobacter_siccitolerans
gi|518494415~Rhodococcus_sp__29MFTsu3_1
gi|827192870~Rhodococcus_sp__IITR03
gi|831205870~Altererythrobacter_marensis
gi|836658654~Altererythrobacter_marensis
gi|1055998976~Altererythrobacter_sp__MH~B5
GSMAST4A1_ASY1_ANO1_2516_1
gi|916837009~Afipia_sp__P52~10
gi|297181457~uncultured_alpha_proteobacterium_HF0130_20P23
gi|938895183~Microbacterium_sp__No__7
gi|702860960~Amycolatopsis_jejuensis
gi|503438881~Pseudonocardia_dioxanivorans_CB1190
gi|503440675~Pseudonocardia_dioxanivorans_CB1190
gi|635709199~Mycobacterium_aromaticivorans_JS19b1_=_JCM_16368
gi|829463678~Mycobacterium_sp__EPa45
gi|1055454439~Mycobacterium_sp__852002~51057_SCH5723018
gi|494898278~Gordonia_soli_NBRC_108243
gi|972137128~Valsa_mali
gi|1024102080~Exidia_glandulosa_HHB12029
gi|1042851549|sp|A0A097ZPG2_1|ANDJ_EMEVA~Aspergillus_stellatus
gi|628191574~Bipolaris_zeicola_26~R~13
gi|953426433~Bipolaris_victoriae_FI3
gi|949386185|dbj|GAP90886_1|~Rosellinia_necatrix
0.1
Bacteria
Eukaryota
SAG

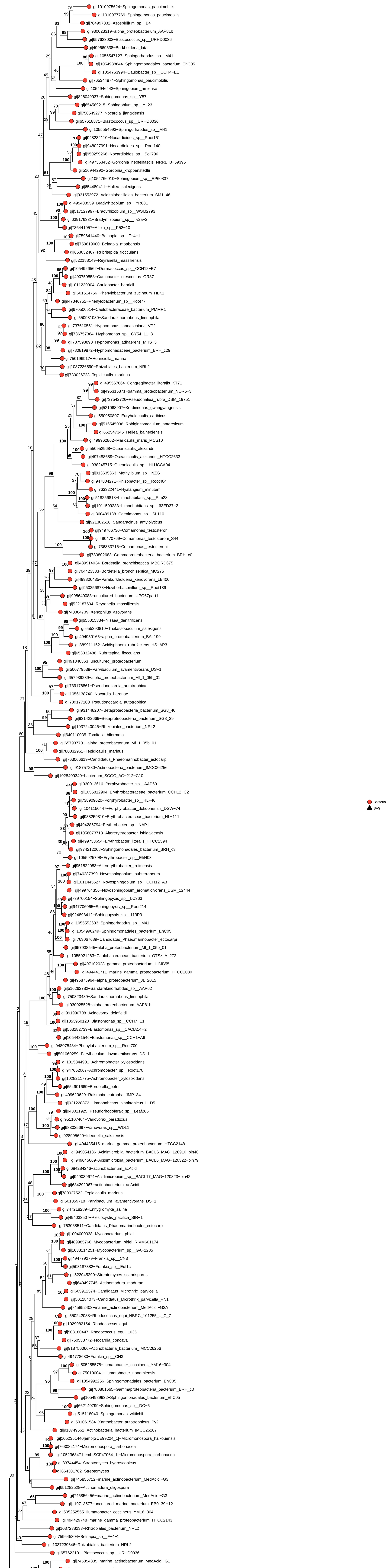
Bacteria
SAG
0.1

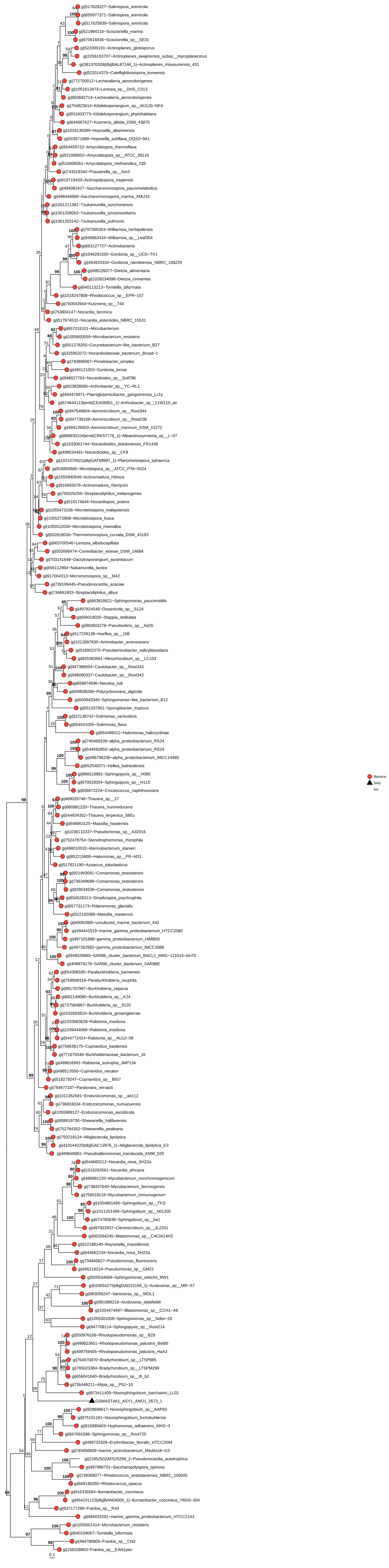
gi|517628227~Salinispora_arenicola
gi|655977371~Salinispora_arenicola
gi|517625839~Salinispora_arenicola
gi|521984216~Sciscionella_marina
gi|670516836~Sciscionella_sp__SE31
gi|522000191~Actinoplanes_globisporus
gi|1056193707~Actinoplanes_awajinensis_subsp__mycoplanecinus
gi|381370328|dbj|BAL87146_1|~Actinoplanes_missouriensis_431
gi|522014375~Catelliglobosispora_koreensis
gi|772750012~Lechevalieria_aerocolonigenes
gi|1051613473~Lentzea_sp__DHS_C013
gi|663692714~Lechevalieria_aerocolonigenes
gi|754823614~Kibdelosporangium_sp__MJ126~NF4
gi|931603773~Kibdelosporangium_phytohabitans
gi|644887627~Kutzneria_albida_DSM_43870
gi|1033136399~Hoyosella_altamirensis
gi|503571668~Hoyosella_subflava_DQS3~9A1
gi|654459722~Amycolatopsis_thermoflava
gi|521089002~Amycolatopsis_sp__ATCC_39116
gi|516608361~Amycolatopsis_methanolica_239
gi|743318340~Prauserella_sp__Am3
gi|918719433~Actinopolyspora_iraqiensis
gi|494082427~Saccharomonospora_paurometabolica
gi|496444868~Saccharomonospora_marina_XMU15
gi|1001211381~Tsukamurella_sunchonensis
gi|1001208052~Tsukamurella_tyrosinosolvens
gi|1001203142~Tsukamurella_pulmonis
gi|797065353~Williamsia_herbipolensis
gi|946863434~Williamsia_sp__Leaf354
gi|663127927~Actinobacteria
gi|1046281550~Gordonia_sp__UCD~TK1
gi|493920334~Gordonia_namibiensis_NBRC_108229
gi|498226077~Dietzia_alimentaria
gi|1028234098~Dietzia_cinnamea
gi|640113213~Tomitella_biformata
gi|1018247808~Rhodococcus_sp__EPR~157
gi|760043944~Kutzneria_sp__744
gi|753804147~Nocardia_farcinica
gi|517874531~Nocardia_asteroides_NBRC_15531
gi|657018101~Microbacterium
gi|1055600559~Microbacterium_resistens
gi|551278255~Corynebacterium~like_bacterium_B27
gi|325953272~Nocardioidaceae_bacterium_Broad~1
gi|740896967~Pimelobacter_simplex
gi|490121003~Gordonia_terrae
gi|948027763~Nocardioides_sp__Soil796
gi|823838080~Arthrobacter_sp__YC~RL1
gi|494479971~Paeniglutamicibacter_gangotriensis_Lz1y
gi|674644113|emb|CEA06951_1|~Arthrobacter_sp__11W110_air
gi|947546604~Aeromicrobium_sp__Root344
gi|947739108~Aeromicrobium_sp__Root236
gi|494139003~Aeromicrobium_marinum_DSM_15272
gi|886830104|emb|CRK57776_1|~Alloactinosynnema_sp__L~07
gi|1033001744~Nocardioides_dokdonensis_FR1436
gi|498534491~Nocardioides_sp__CF8
gi|1021070921|dbj|GAT69897_1|~Planomonospora_sphaerica
gi|916850685~Microbispora_sp__ATCC_PTA~5024
gi|1055940546~Actinomadura_hibisca
gi|916693076~Actinomadura_rifamycini
gi|755026259~Streptacidiphilus_melanogenes
gi|516174644~Nocardiopsis_potens
gi|1055473166~Microtetraspora_malaysiensis
gi|1055272808~Microtetraspora_fusca
gi|1055222030~Microtetraspora_niveoalba
gi|502618036~Thermomonospora_curvata_DSM_43183
gi|663700546~Lentzea_albidocapillata
gi|502698474~Conexibacter_woesei_DSM_14684
gi|703141648~Dactylosporangium_aurantiacum
gi|656112964~Nakamurella_lactea
gi|917004313~Micromonospora_sp__M42
gi|739199445~Pseudonocardia_acaciae
gi|734691903~Streptacidiphilus_albus
gi|663818621~Sphingomonas_paucimobilis
gi|497824546~Oceanicola_sp__S124
gi|656018035~Stappia_stellulata
gi|950603278~Pseudovibrio_sp__Ad26
gi|517239138~Hoeflea_sp__108
gi|1012897930~Aminobacter_aminovorans
gi|518002375~Pseudaminobacter_salicylatoxidans
gi|825383561~Mesorhizobium_sp__LC103
gi|947388504~Caulobacter_sp__Root343
gi|948090337~Caulobacter_sp__Root342
gi|659874596~Nevskia_soli
gi|659838266~Polycyclovorans_algicola
gi|550942040~Sphingomonas~like_bacterium_B12
gi|551337901~Spongiibacter_tropicus
gi|522138742~Solimonas_variicoloris
gi|654541005~Solimonas_flava
gi|654498012~Halomonas_halocynthiae
gi|740469109~alpha_proteobacterium_RS24
gi|544592853~alpha_proteobacterium_RS24
gi|495796236~alpha_proteobacterium_IMCC14465
gi|652548371~Hellea_balneolensis
gi|966519881~Sphingopyxis_sp__H085
gi|970533004~Sphingopyxis_sp__H115
gi|836672224~Croceicoccus_naphthovorans
gi|489028748~Thauera_sp__27
gi|880981220~Thauera_humireducens
gi|544834352~Thauera_terpenica_58Eu
gi|648663125~Massilia_niastensis
gi|1036110337~Pseudomonas_sp__A32016
gi|752478754~Stenotrophomonas_rhizophila
gi|498010532~Marinobacterium_stanieri
gi|852215895~Halomonas_sp__PR~M31
gi|517821190~Azoarcus_toluclasticus
gi|921993091~Comamonas_testosteroni
gi|736349699~Comamonas_testosteroni
gi|929534536~Comamonas_testosteroni
gi|654528313~Simplicispira_psychrophila
gi|657731173~Polaromonas_glacialis
gi|522142086~Massilia_niastensis
gi|40062865~uncultured_marine_bacterium_442
gi|494441519~marine_gamma_proteobacterium_HTCC2080
gi|497101888~gamma_proteobacterium_HIMB55
gi|497262582~gamma_proteobacterium_IMCC3088
gi|949026865~SAR86_cluster_bacterium_BACL1_MAG~121015~bin70
gi|409979178~SAR86_cluster_bacterium_SAR86E
gi|654306595~Paraburkholderia_bannensis
gi|759569318~Paraburkholderia_oxyphila
gi|981707967~Burkholderia_cepacia
gi|662149085~Burkholderia_sp__K24
gi|737584867~Burkholderia_sp__9120
gi|1032693810~Burkholderia_ginsengiterrae
gi|1033683628~Ralstonia_insidiosa
gi|1039444066~Ralstonia_insidiosa
gi|544772424~Ralstonia_sp__AU12~08
gi|759638175~Cupriavidus_basilensis
gi|771675546~Burkholderiaceae_bacterium_16
gi|499616991~Ralstonia_eutropha_JMP134
gi|498513550~Cupriavidus_necator
gi|518279247~Cupriavidus_sp__BIS7
gi|764677337~Pandoraea_vervacti
gi|1011352681~Endozoicomonas_sp__ab112
gi|736833034~Endozoicomonas_numazuensis
gi|1055989127~Endozoicomonas_ascidiicola
gi|908619730~Shewanella_halifaxensis
gi|752764352~Shewanella_pealeana
gi|750218124~Aliiglaciecola_lipolytica
gi|410144220|dbj|GAC13976_1|~Aliiglaciecola_lipolytica_E3
gi|499646861~Pseudoalteromonas_translucida_KMM_520
gi|644660212~Nocardia_nova_SH22a
gi|1016283561~Nocardia_africana
gi|489981220~Mycobacterium_nonchromogenicum
gi|738437649~Mycobacterium_farcinogenes
gi|759018216~Mycobacterium_immunogenum
gi|1054801495~Sphingobium_sp__TKS
gi|1011201489~Sphingobium_sp__MI1205
gi|674785638~Sphingobium_sp__ba1
gi|497922937~Citromicrobium_sp__JL2201
gi|563284245~Blastomonas_sp__CACIA14H2
gi|522188140~Reyranella_massiliensis
gi|644662234~Nocardia_nova_SH22a
gi|734945827~Pseudomonas_fluorescens
gi|495218224~Pseudomonas_sp__GM21
gi|500534669~Sphingomonas_wittichii_RW1
gi|524554275|dbj|GAD22156_1|~Acidovorax_sp__MR~S7
gi|983056247~Variovorax_sp__WDL1
gi|991989218~Acidovorax_delafieldii
gi|1054474597~Blastomonas_sp__CCH1~A6
gi|1055301059~Sphingomonas_sp__Ndbn~20
gi|947708114~Sphingopyxis_sp__Root214
gi|550976108~Rhodopseudomonas_sp__B29
gi|499823651~Rhodopseudomonas_palustris_BisB5
gi|499759405~Rhodopseudomonas_palustris_HaA2
gi|764970870~Bradyrhizobium_sp__LTSP885
gi|765023384~Bradyrhizobium_sp__LTSPM299
gi|656041640~Bradyrhizobium_sp__th_b2
gi|736448211~Afipia_sp__P52~10
gi|973411409~Novosphingobium_barchaimii_LL02
GSMAST4A1_ASY1_ANO1_2673_1
gi|929698617~Novosphingobium_sp__AAP93
gi|975151181~Novosphingobium_fuchskuhlense
gi|916989403~Hyphomonas_adhaerens_MHS~3
gi|947691586~Sphingomonas_sp__Root720
gi|499732928~Erythrobacter_litoralis_HTCC2594
gi|745856609~marine_actinobacterium_MedAcid~G3
gi|21952502AF525299_2~Pseudonocardia_autotrophica
gi|497996701~Saccharopolyspora_spinosa
gi|739368077~Rhodococcus_wratislaviensis_NBRC_100605
gi|669180350~Rhodococcus_opacus
gi|916336564~Ilumatobacter_coccineus
gi|464101123|dbj|BAN04005_1|~Ilumatobacter_coccineus_YM16~304
gi|937177299~Frankia_sp__R43
gi|494433291~marine_gamma_proteobacterium_HTCC2143
gi|1055601414~Microbacterium_resistens
gi|640109067~Tomitella_biformata
gi|494780805~Frankia_sp__CN3
gi|158108863~Frankia_sp__EAN1pec
0.1
Bacteria
SAG
NA

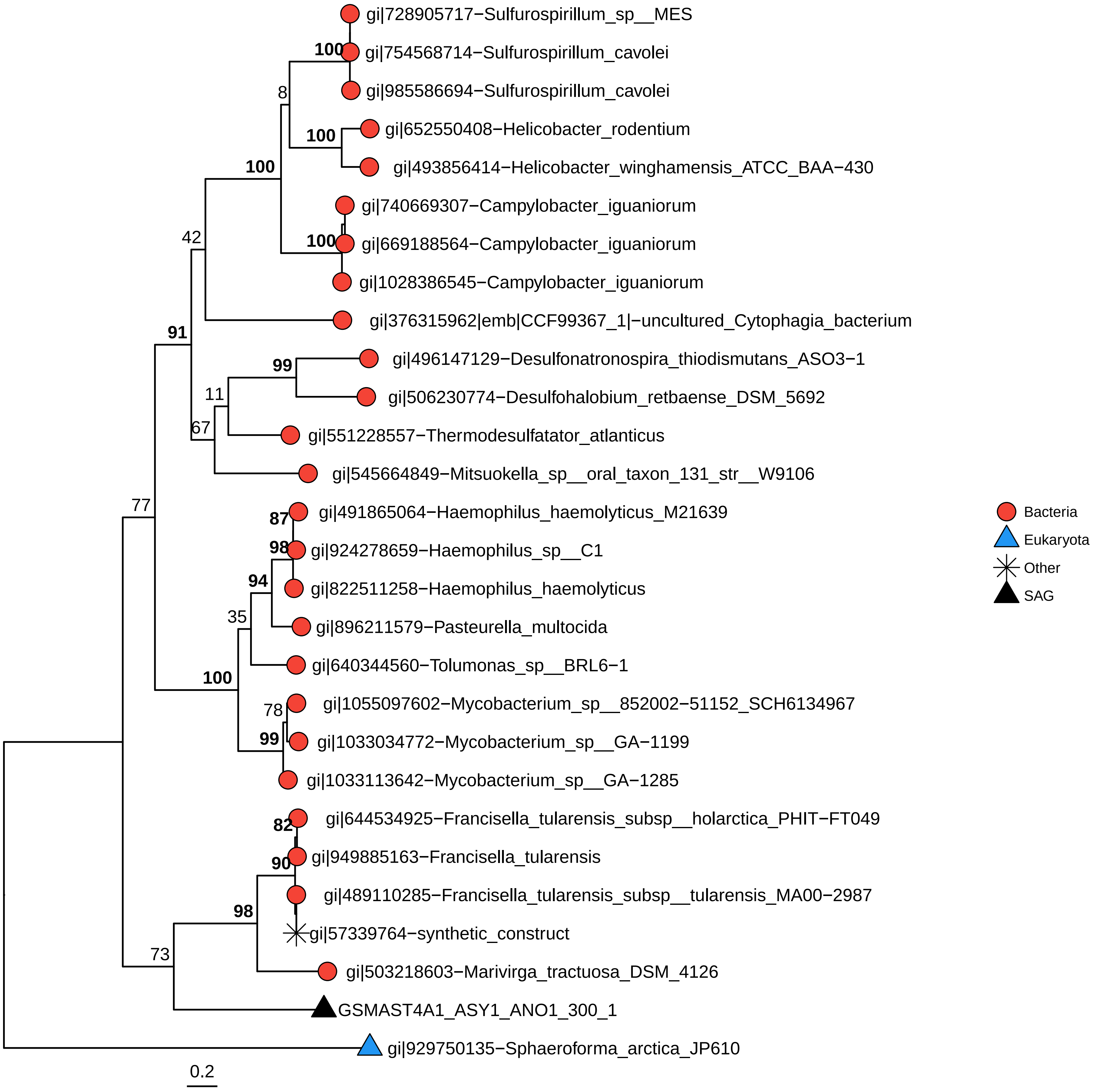
gi|728905717−Sulfurospirillum_sp__MES
gi|754568714−Sulfurospirillum_cavolei
gi|985586694−Sulfurospirillum_cavolei
gi|652550408−Helicobacter_rodentium
gi|493856414−Helicobacter_winghamensis_ATCC_BAA−430
gi|740669307−Campylobacter_iguaniorum
gi|669188564−Campylobacter_iguaniorum
gi|1028386545−Campylobacter_iguaniorum
gi|376315962|emb|CCF99367_1|−uncultured_Cytophagia_bacterium
gi|496147129−Desulfonatronospira_thiodismutans_ASO3−1
gi|506230774−Desulfohalobium_retbaense_DSM_5692
gi|551228557−Thermodesulfatator_atlanticus
gi|545664849−Mitsuokella_sp__oral_taxon_131_str__W9106
gi|491865064−Haemophilus_haemolyticus_M21639
gi|924278659−Haemophilus_sp__C1
gi|822511258−Haemophilus_haemolyticus
gi|896211579−Pasteurella_multocida
gi|640344560−Tolumonas_sp__BRL6−1
gi|1055097602−Mycobacterium_sp__852002−51152_SCH6134967
gi|1033034772−Mycobacterium_sp__GA−1199
gi|1033113642−Mycobacterium_sp__GA−1285
gi|644534925−Francisella_tularensis_subsp__holarctica_PHIT−FT049
gi|949885163−Francisella_tularensis
gi|489110285−Francisella_tularensis_subsp__tularensis_MA00−2987
gi|57339764−synthetic_construct
gi|503218603−Marivirga_tractuosa_DSM_4126
GSMAST4A1_ASY1_ANO1_300_1
gi|929750135−Sphaeroforma_arctica_JP610
100
8
100
100
42
100
91
99
11
67
77
87
98
94
35
100
78
99
82
90
98
73
0.2
Bacteria
Eukaryota
Other
SAG

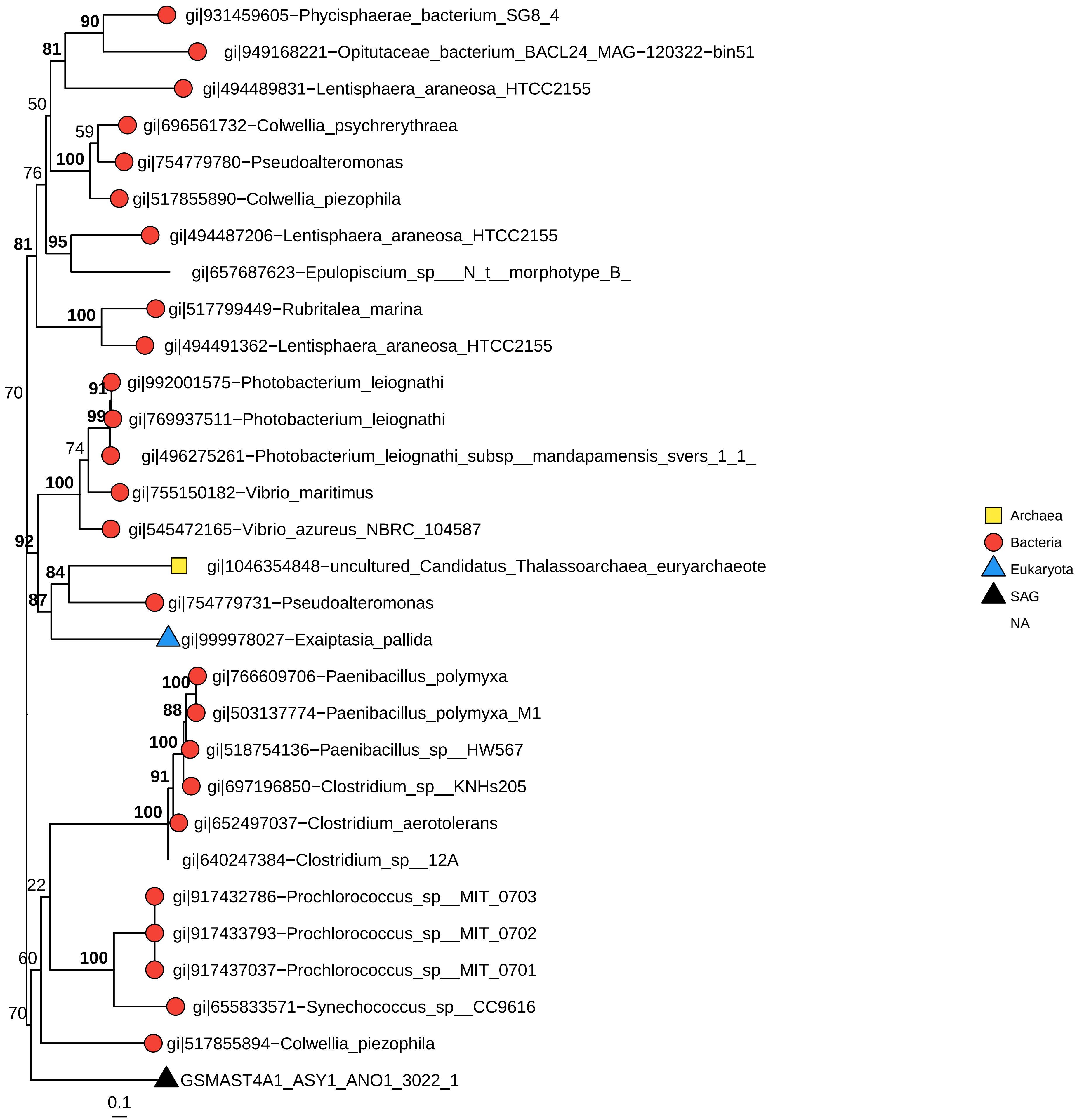
gi|931459605−Phycisphaerae_bacterium_SG8_4
gi|949168221−Opitutaceae_bacterium_BACL24_MAG−120322−bin51
gi|494489831−Lentisphaera_araneosa_HTCC2155
gi|696561732−Colwellia_psychrerythraea
gi|754779780−Pseudoalteromonas
gi|517855890−Colwellia_piezophila
gi|494487206−Lentisphaera_araneosa_HTCC2155
gi|657687623−Epulopiscium_sp___N_t__morphotype_B_
gi|517799449−Rubritalea_marina
gi|494491362−Lentisphaera_araneosa_HTCC2155
gi|992001575−Photobacterium_leiognathi
gi|769937511−Photobacterium_leiognathi
gi|496275261−Photobacterium_leiognathi_subsp__mandapamensis_svers_1_1_
gi|755150182−Vibrio_maritimus
gi|545472165−Vibrio_azureus_NBRC_104587
gi|1046354848−uncultured_Candidatus_Thalassoarchaea_euryarchaeote
gi|754779731−Pseudoalteromonas
gi|999978027−Exaiptasia_pallida
gi|766609706−Paenibacillus_polymyxa
gi|503137774−Paenibacillus_polymyxa_M1
gi|518754136−Paenibacillus_sp__HW567
gi|697196850−Clostridium_sp__KNHs205
gi|652497037−Clostridium_aerotolerans
gi|640247384−Clostridium_sp__12A
gi|917432786−Prochlorococcus_sp__MIT_0703
gi|917433793−Prochlorococcus_sp__MIT_0702
gi|917437037−Prochlorococcus_sp__MIT_0701
gi|655833571−Synechococcus_sp__CC9616
gi|517855894−Colwellia_piezophila
GSMAST4A1_ASY1_ANO1_3022_1
0.1
Archaea
Bacteria
Eukaryota
SAG
NA

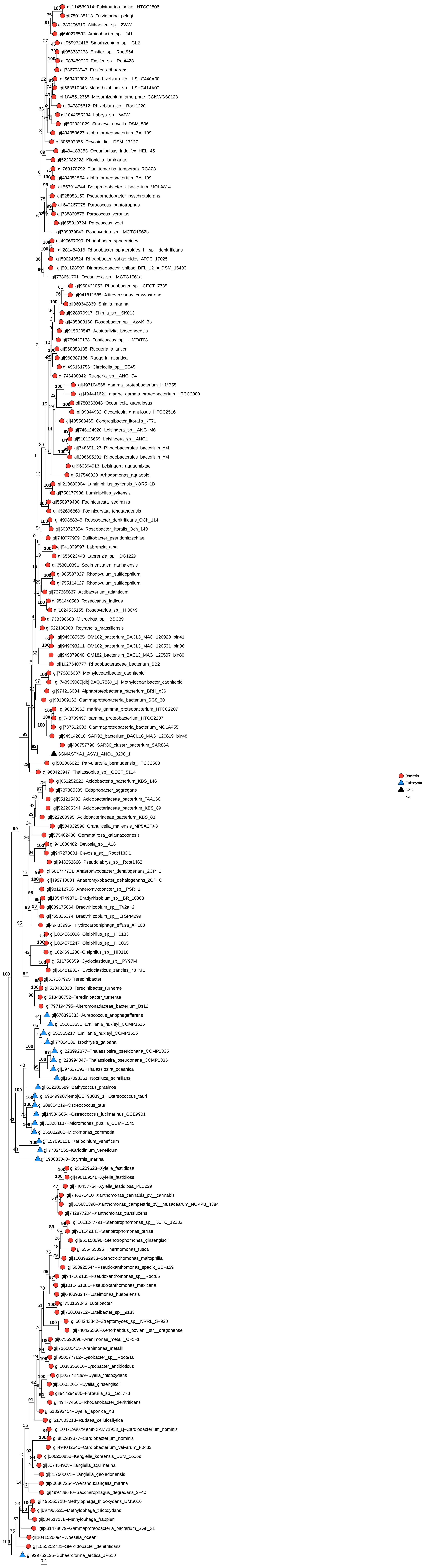

gi|114539014~Fulvimarina_pelagi_HTCC2506
gi|750185113~Fulvimarina_pelagi
gi|639296519~Aliihoeflea_sp__2WW
gi|640276593~Aminobacter_sp__J41
gi|959972415~Sinorhizobium_sp__GL2
gi|983337273~Ensifer_sp__Root954
gi|983489720~Ensifer_sp__Root423
gi|736793947~Ensifer_adhaerens
gi|563482302~Mesorhizobium_sp__LSHC440A00
gi|563510343~Mesorhizobium_sp__LSHC414A00
gi|1045512365~Mesorhizobium_amorphae_CCNWGS0123
gi|947875612~Rhizobium_sp__Root1220
gi|1044655284~Labrys_sp__WJW
gi|502931829~Starkeya_novella_DSM_506
gi|494950627~alpha_proteobacterium_BAL199
gi|806503355~Devosia_limi_DSM_17137
gi|494183353~Oceanibulbus_indolifex_HEL~45
gi|522082228~Kiloniella_laminariae
gi|763170792~Planktomarina_temperata_RCA23
gi|494951564~alpha_proteobacterium_BAL199
gi|557914544~Betaproteobacteria_bacterium_MOLA814
gi|928983150~Pseudorhodobacter_psychrotolerans
gi|640267078~Paracoccus_pantotrophus
gi|738860878~Paracoccus_versutus
gi|655310724~Paracoccus_yeei
gi|739379843~Roseovarius_sp__MCTG1562b
gi|499657990~Rhodobacter_sphaeroides
gi|281484916~Rhodobacter_sphaeroides_f__sp__denitrificans
gi|500249524~Rhodobacter_sphaeroides_ATCC_17025
gi|501128596~Dinoroseobacter_shibae_DFL_12_=_DSM_16493
gi|738651701~Oceanicola_sp__MCTG1561a
gi|960421053~Phaeobacter_sp__CECT_7735
gi|941811585~Aliiroseovarius_crassostreae
gi|960342869~Shimia_marina
gi|928979917~Shimia_sp__SK013
gi|495088160~Roseobacter_sp__AzwK~3b
gi|915920547~Aestuariivita_boseongensis
gi|759420178~Ponticoccus_sp__UMTAT08
gi|960383135~Ruegeria_atlantica
gi|960387186~Ruegeria_atlantica
gi|496161756~Citreicella_sp__SE45
gi|746488042~Ruegeria_sp__ANG~S4
gi|497104868~gamma_proteobacterium_HIMB55
gi|494441621~marine_gamma_proteobacterium_HTCC2080
gi|750333048~Oceanicola_granulosus
gi|89044982~Oceanicola_granulosus_HTCC2516
gi|495568465~Congregibacter_litoralis_KT71
gi|746124920~Leisingera_sp__ANG~M6
gi|518126669~Leisingera_sp__ANG1
gi|748691127~Rhodobacterales_bacterium_Y4I
gi|206685201~Rhodobacterales_bacterium_Y4I
gi|960394913~Leisingera_aquaemixtae
gi|517546323~Arhodomonas_aquaeolei
gi|219680004~Luminiphilus_syltensis_NOR5~1B
gi|750177986~Luminiphilus_syltensis
gi|550979400~Fodinicurvata_sediminis
gi|652606860~Fodinicurvata_fenggangensis
gi|499888345~Roseobacter_denitrificans_OCh_114
gi|503727354~Roseobacter_litoralis_Och_149
gi|740079959~Sulfitobacter_pseudonitzschiae
gi|941309597~Labrenzia_alba
gi|656023443~Labrenzia_sp__DG1229
gi|653010391~Sedimentitalea_nanhaiensis
gi|985597027~Rhodovulum_sulfidophilum
gi|755114127~Rhodovulum_sulfidophilum
gi|737268627~Actibacterium_atlanticum
gi|951440568~Roseovarius_indicus
gi|1024535353~Roseovarius_sp__HI0049
gi|738398683~Microvirga_sp__BSC39
gi|522190908~Reyranella_massiliensis
gi|949085585~OM182_bacterium_BACL3_MAG~120920~bin41
gi|949093211~OM182_bacterium_BACL3_MAG~120531~bin86
gi|949079840~OM182_bacterium_BACL3_MAG~120507~bin80
gi|1027540777~Rhodobacteraceae_bacterium_SB2
gi|779896037~Methyloceanibacter_caenitepidi
gi|743969085|dbj|BAQ17869_1|~Methyloceanibacter_caenitepidi
gi|974216004~Alphaproteobacteria_bacterium_BRH_c36
gi|931389162~Gammaproteobacteria_bacterium_SG8_30
gi|90330962~marine_gamma_proteobacterium_HTCC2207
gi|748709497~gamma_proteobacterium_HTCC2207
gi|737512603~Gammaproteobacteria_bacterium_MOLA455
gi|949142610~SAR92_bacterium_BACL16_MAG~120619~bin48
gi|400757790~SAR86_cluster_bacterium_SAR86A
GSMAST4A1_ASY1_ANO1_3200_1
gi|503066622~Parvularcula_bermudensis_HTCC2503
gi|960423947~Thalassobius_sp__CECT_5114
gi|651252822~Acidobacteria_bacterium_KBS_146
gi|737365335~Edaphobacter_aggregans
gi|551215482~Acidobacteriaceae_bacterium_TAA166
gi|522205344~Acidobacteriaceae_bacterium_KBS_89
gi|522200995~Acidobacteriaceae_bacterium_KBS_83
gi|504032590~Granulicella_mallensis_MP5ACTX8
gi|575462436~Gemmatirosa_kalamazoonesis
gi|941030482~Devosia_sp__A16
gi|947273601~Devosia_sp__Root413D1
gi|948253666~Pseudolabrys_sp__Root1462
gi|501747731~Anaeromyxobacter_dehalogenans_2CP~1
gi|499740634~Anaeromyxobacter_dehalogenans_2CP~C
gi|981212766~Anaeromyxobacter_sp__PSR~1
gi|1054749871~Bradyrhizobium_sp__BR_10303
gi|639175064~Bradyrhizobium_sp__Tv2a~2
gi|765026374~Bradyrhizobium_sp__LTSPM299
gi|494339954~Hydrocarboniphaga_effusa_AP103
gi|1024566006~Oleiphilus_sp__HI0133
gi|1024575247~Oleiphilus_sp__HI0065
gi|1024691288~Oleiphilus_sp__HI0118
gi|511756659~Cycloclasticus_sp__PY97M
gi|504819317~Cycloclasticus_zancles_78~ME
gi|517087995~Teredinibacter
gi|518433833~Teredinibacter_turnerae
gi|518430752~Teredinibacter_turnerae
gi|797194795~Alteromonadaceae_bacterium_Bs12
gi|676396333~Aureococcus_anophagefferens
gi|551613651~Emiliania_huxleyi_CCMP1516
gi|551555217~Emiliania_huxleyi_CCMP1516
gi|77024089~Isochrysis_galbana
gi|223992877~Thalassiosira_pseudonana_CCMP1335
gi|223994047~Thalassiosira_pseudonana_CCMP1335
gi|397627193~Thalassiosira_oceanica
gi|157093361~Noctiluca_scintillans
gi|612386589~Bathycoccus_prasinos
gi|693499987|emb|CEF98039_1|~Ostreococcus_tauri
gi|308804219~Ostreococcus_tauri
gi|145346654~Ostreococcus_lucimarinus_CCE9901
gi|303284187~Micromonas_pusilla_CCMP1545
gi|255082900~Micromonas_commoda
gi|157093121~Karlodinium_veneficum
gi|77024155~Karlodinium_veneficum
gi|190683040~Oxyrrhis_marina
gi|951209623~Xylella_fastidiosa
gi|490189548~Xylella_fastidiosa
gi|740437754~Xylella_fastidiosa_PLS229
gi|746371410~Xanthomonas_cannabis_pv__cannabis
gi|515680390~Xanthomonas_campestris_pv__musacearum_NCPPB_4384
gi|742877204~Xanthomonas_translucens
gi|1011247791~Stenotrophomonas_sp__KCTC_12332
gi|951149143~Stenotrophomonas_terrae
gi|951158896~Stenotrophomonas_ginsengisoli
gi|655455896~Thermomonas_fusca
gi|1003982933~Stenotrophomonas_maltophilia
gi|503925544~Pseudoxanthomonas_spadix_BD~a59
gi|947169135~Pseudoxanthomonas_sp__Root65
gi|1011461081~Pseudoxanthomonas_mexicana
gi|640393247~Luteimonas_huabeiensis
gi|738159045~Luteibacter
gi|760008712~Luteibacter_sp__9133
gi|664243342~Streptomyces_sp__NRRL_S~920
gi|740425566~Xenorhabdus_bovienii_str__oregonense
gi|755900098~Arenimonas_metalli_CF5~1
gi|736081425~Arenimonas_metalli
gi|950077762~Lysobacter_sp__Root916
gi|1038356616~Lysobacter_antibioticus
gi|1027737399~Dyella_thiooxydans
gi|516032614~Dyella_ginsengisoli
gi|947294936~Frateuria_sp__Soil773
gi|494774561~Rhodanobacter_denitrificans
gi|518293414~Dyella_japonica_A8
gi|517803213~Rudaea_cellulosilytica
gi|1047198079|emb|SAM71913_1|~Cardiobacterium_hominis
gi|880989877~Cardiobacterium_hominis
gi|494042346~Cardiobacterium_valvarum_F0432
gi|506260858~Kangiella_koreensis_DSM_16069
gi|517454908~Kangiella_aquimarina
gi|817505075~Kangiella_geojedonensis
gi|906867254~Wenzhouxiangella_marina
gi|499788640~Saccharophagus_degradans_2~40
gi|495565718~Methylophaga_thiooxydans_DMS010
gi|697965221~Methylophaga_thiooxydans
gi|504517178~Methylophaga_frappieri
gi|931478679~Gammaproteobacteria_bacterium_SG8_31
gi|1041526094~Woeseia_oceani
gi|1055252731~Steroidobacter_denitrificans
gi|929752125~Sphaeroforma_arctica_JP610
0.1
Bacteria
Eukaryota
SAG
NA

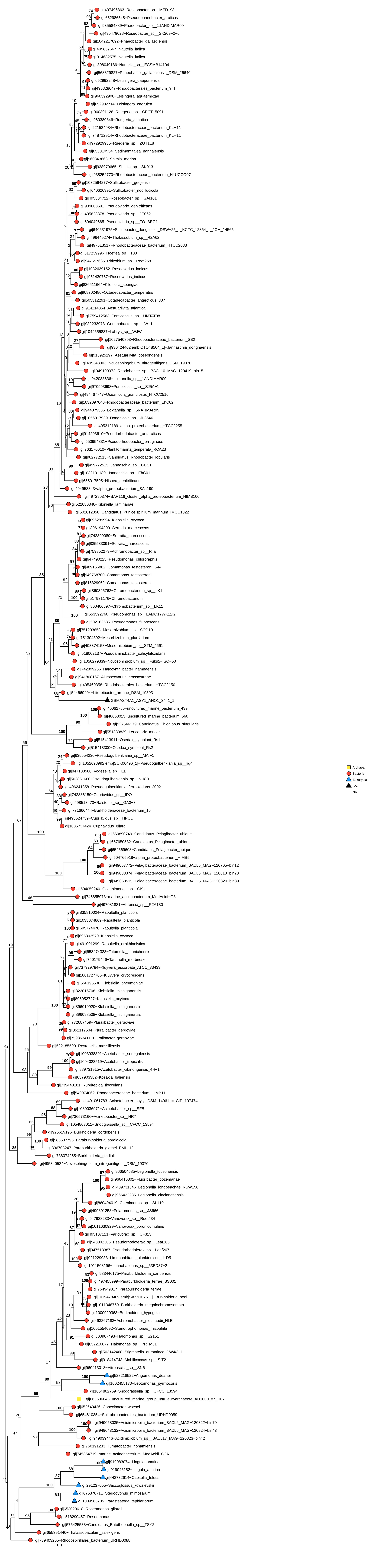
Archaea
Bacteria
Eukaryota
SAG
NA
0.1

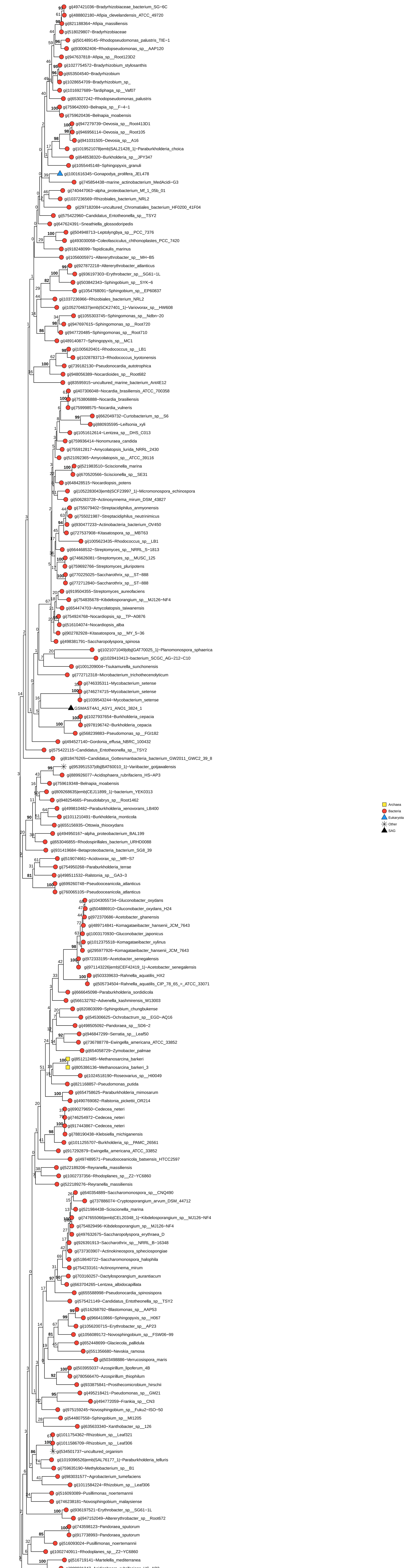
Archaea
Bacteria
Eukaryota
Other
SAG
0.1

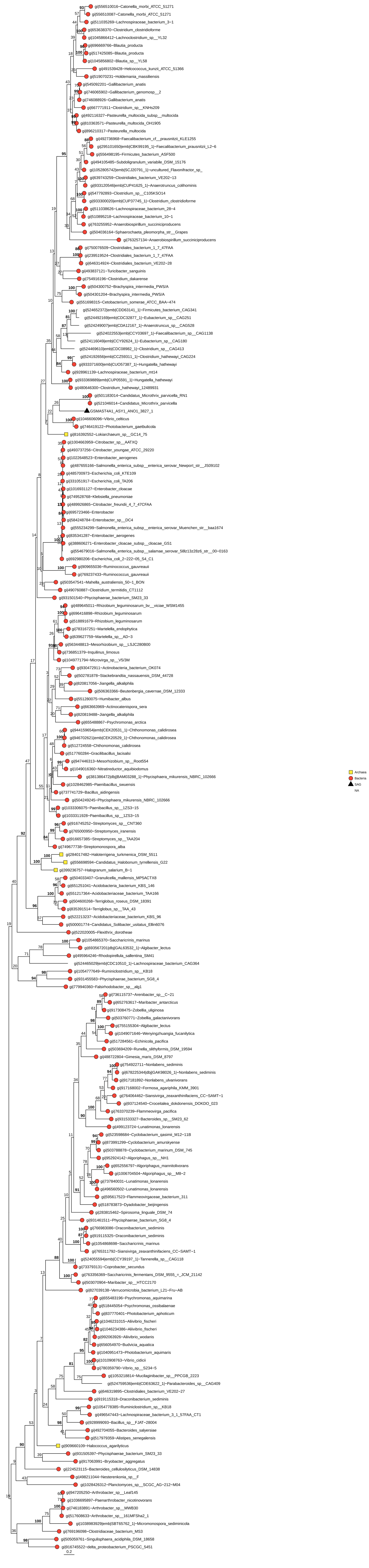

Archaea
Bacteria
SAG
NA
0.2

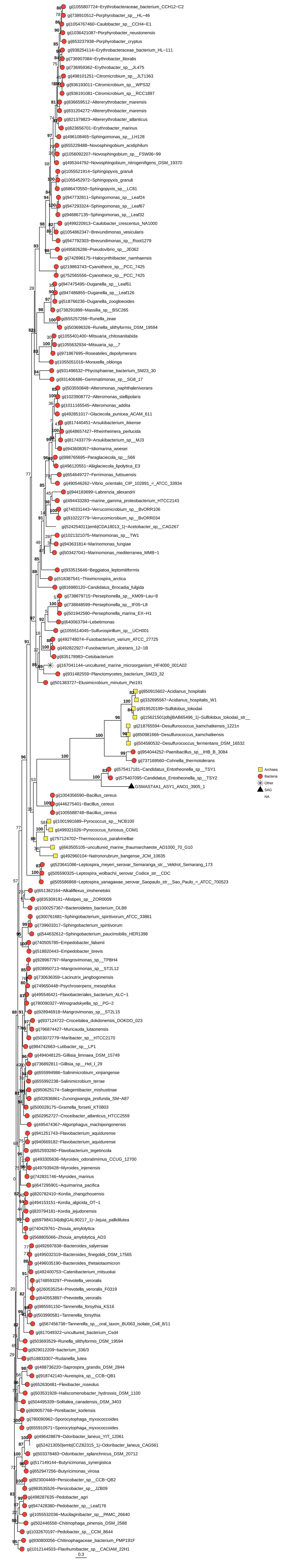

Archaea
Bacteria
Other
SAG
NA
0.3

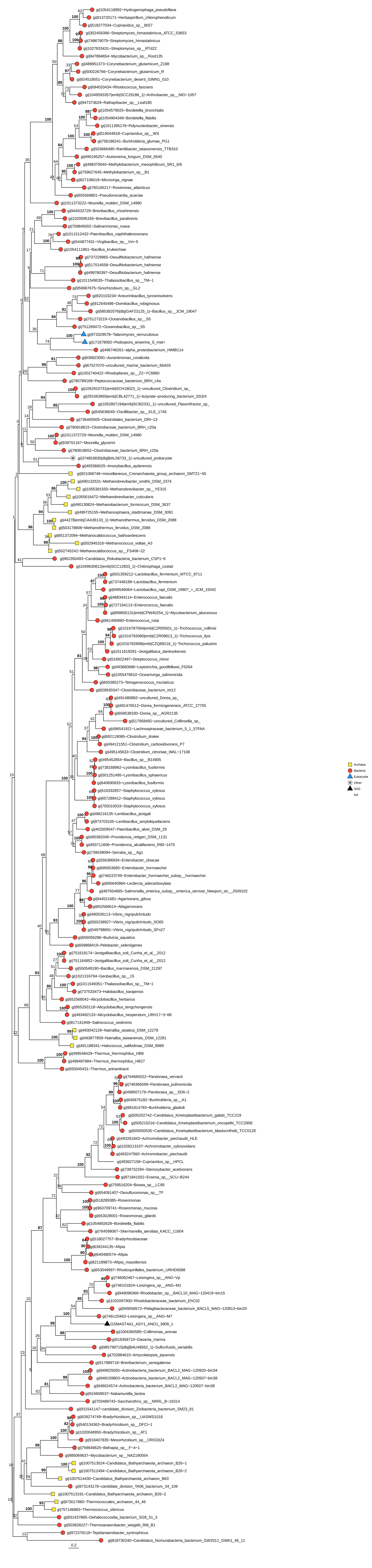

Archaea
Bacteria
Eukaryota
Other
SAG
NA
0.2

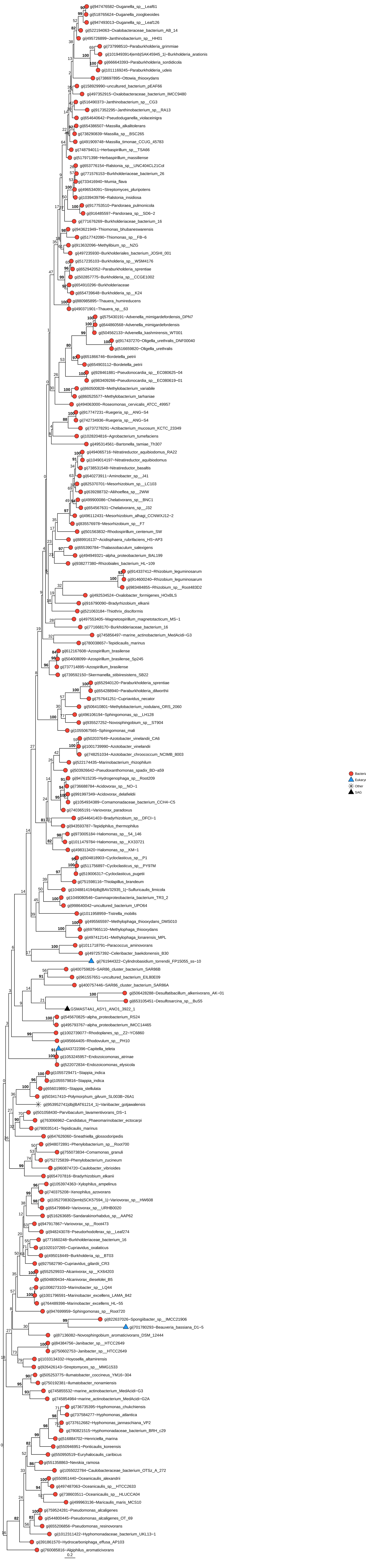
Bacteria
Eukaryota
Other
SAG
0.2

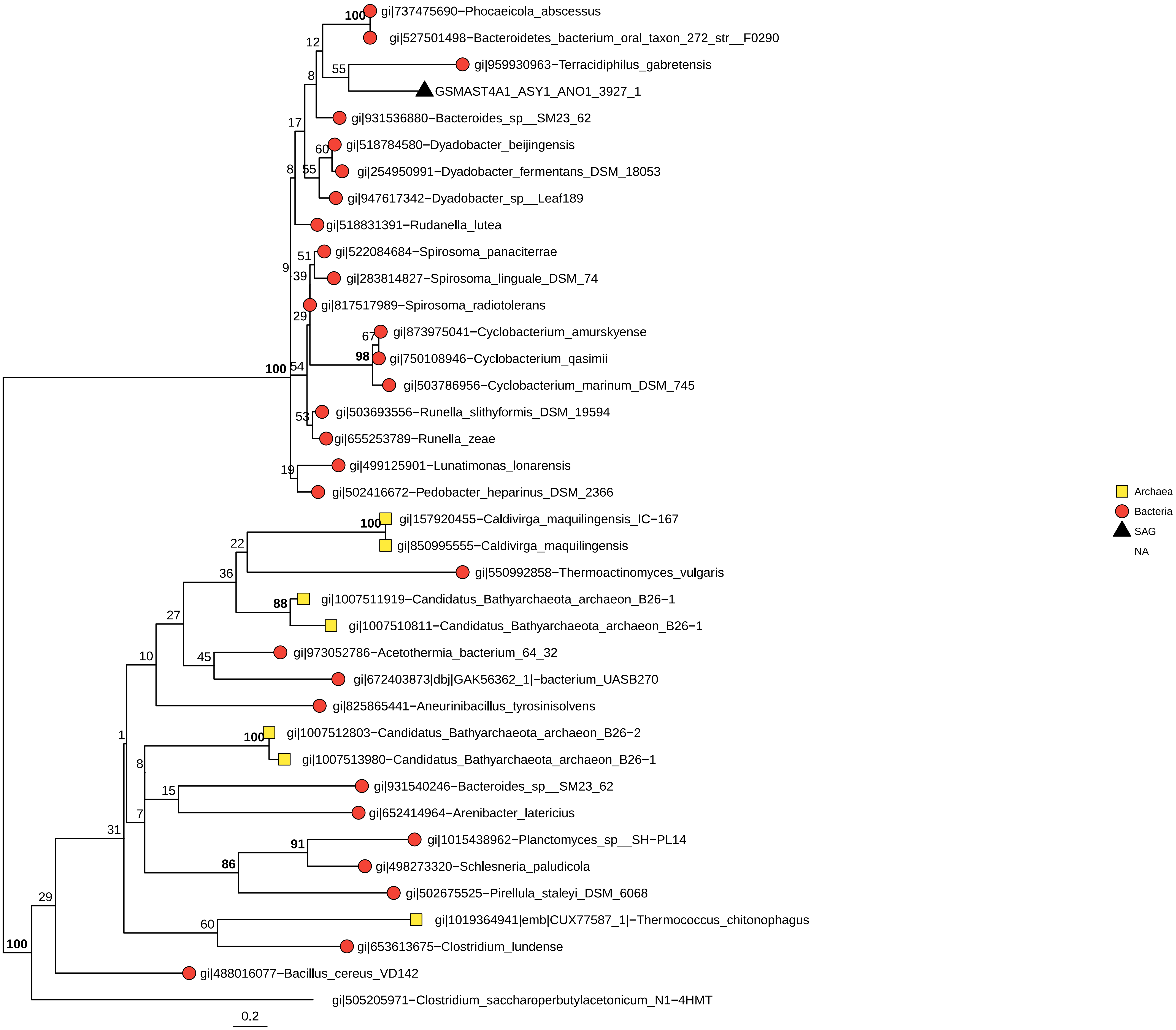
gi|737475690−Phocaeicola_abscessus
gi|527501498−Bacteroidetes_bacterium_oral_taxon_272_str__F0290
gi|959930963−Terracidiphilus_gabretensis
GSMAST4A1_ASY1_ANO1_3927_1
gi|931536880−Bacteroides_sp__SM23_62
gi|518784580−Dyadobacter_beijingensis
gi|254950991−Dyadobacter_fermentans_DSM_18053
gi|947617342−Dyadobacter_sp__Leaf189
gi|518831391−Rudanella_lutea
gi|522084684−Spirosoma_panaciterrae
gi|283814827−Spirosoma_linguale_DSM_74
gi|817517989−Spirosoma_radiotolerans
gi|873975041−Cyclobacterium_amurskyense
gi|750108946−Cyclobacterium_qasimii
gi|503786956−Cyclobacterium_marinum_DSM_745
gi|503693556−Runella_slithyformis_DSM_19594
gi|655253789−Runella_zeae
gi|499125901−Lunatimonas_lonarensis
gi|502416672−Pedobacter_heparinus_DSM_2366
gi|157920455−Caldivirga_maquilingensis_IC−167
gi|850995555−Caldivirga_maquilingensis
gi|550992858−Thermoactinomyces_vulgaris
gi|1007511919−Candidatus_Bathyarchaeota_archaeon_B26−1
gi|1007510811−Candidatus_Bathyarchaeota_archaeon_B26−1
gi|973052786−Acetothermia_bacterium_64_32
gi|672403873|dbj|GAK56362_1|−bacterium_UASB270
gi|825865441−Aneurinibacillus_tyrosinisolvens
gi|1007512803−Candidatus_Bathyarchaeota_archaeon_B26−2
gi|1007513980−Candidatus_Bathyarchaeota_archaeon_B26−1
gi|931540246−Bacteroides_sp__SM23_62
gi|652414964−Arenibacter_latericius
gi|1015438962−Planctomyces_sp__SH−PL14
gi|498273320−Schlesneria_paludicola
gi|502675525−Pirellula_staleyi_DSM_6068
gi|1019364941|emb|CUX77587_1|−Thermococcus_chitonophagus
gi|653613675−Clostridium_lundense
gi|488016077−Bacillus_cereus_VD142
gi|505205971−Clostridium_saccharoperbutylacetonicum_N1−4HMT
0.2
Archaea
Bacteria
SAG
NA

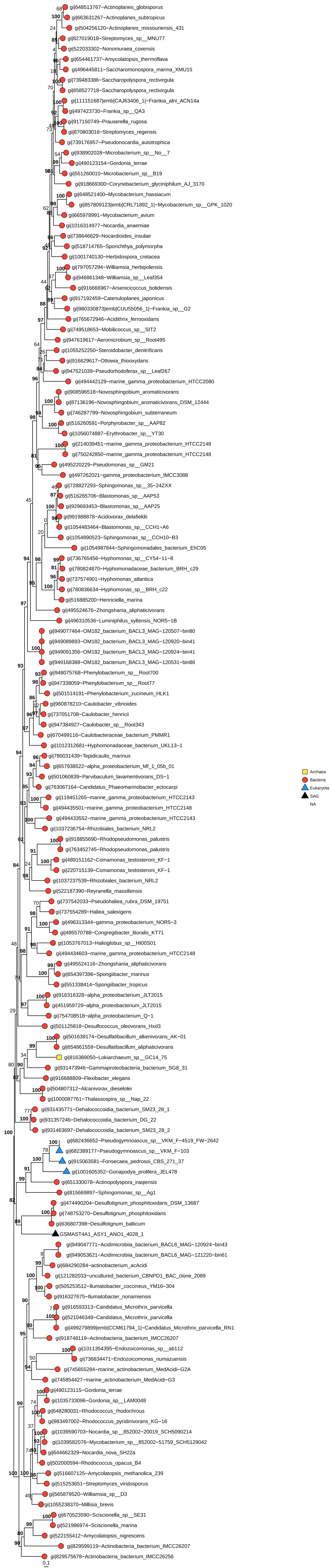
gi|648513767−Actinoplanes_globisporus
gi|663631267−Actinoplanes_subtropicus
gi|504256120−Actinoplanes_missouriensis_431
gi|827019018−Streptomyces_sp__MNU77
gi|522033302−Nonomuraea_coxensis
gi|654461737−Amycolatopsis_thermoflava
gi|496445811−Saccharomonospora_marina_XMU15
gi|739483386−Saccharopolyspora_rectivirgula
gi|658527718−Saccharopolyspora_rectivirgula
gi|111151687|emb|CAJ63406_1|−Frankia_alni_ACN14a
gi|497423730−Frankia_sp__QA3
gi|917150749−Prauserella_rugosa
gi|870803016−Streptomyces_regensis
gi|739176957−Pseudonocardia_autotrophica
gi|938902028−Microbacterium_sp__No__7
gi|490123154−Gordonia_terrae
gi|551260010−Microbacterium_sp__B19
gi|918669300−Corynebacterium_glyciniphilum_AJ_3170
gi|648521400−Mycobacterium_hassiacum
gi|857809123|emb|CRL71892_1|−Mycobacterium_sp__GPK_1020
gi|665978991−Mycobacterium_avium
gi|1016314977−Nocardia_anaemiae
gi|738646629−Nocardioides_insulae
gi|518714765−Sporichthya_polymorpha
gi|1001740130−Herbidospora_cretacea
gi|797057294−Williamsia_herbipolensis
gi|946861348−Williamsia_sp__Leaf354
gi|916666967−Arsenicicoccus_bolidensis
gi|917192459−Catenuloplanes_japonicus
gi|980330873|emb|CUU55056_1|−Frankia_sp__G2
gi|765672946−Acidithrix_ferrooxidans
gi|749518653−Mobilicoccus_sp__SIT2
gi|947619617−Aeromicrobium_sp__Root495
gi|1055252250−Steroidobacter_denitrificans
gi|916629617−Ottowia_thiooxydans
gi|947521039−Pseudorhodoferax_sp__Leaf267
gi|494442129−marine_gamma_proteobacterium_HTCC2080
gi|908596518−Novosphingobium_aromaticivorans
gi|87136196−Novosphingobium_aromaticivorans_DSM_12444
gi|746287799−Novosphingobium_subterraneum
gi|516260591−Porphyrobacter_sp__AAP82
gi|1056074887−Erythrobacter_sp__YT30
gi|214039451−marine_gamma_proteobacterium_HTCC2148
gi|750242850−marine_gamma_proteobacterium_HTCC2148
gi|495220229−Pseudomonas_sp__GM21
gi|497262021−gamma_proteobacterium_IMCC3088
gi|728827293−Sphingomonas_sp__35−24ZXX
gi|516265706−Blastomonas_sp__AAP53
gi|929693453−Blastomonas_sp__AAP25
gi|991988878−Acidovorax_delafieldii
gi|1054483464−Blastomonas_sp__CCH1−A6
gi|1054890523−Sphingomonas_sp__CCH10−B3
gi|1054987844−Sphingomonadales_bacterium_EhC05
gi|736765456−Hyphomonas_sp__CY54−11−8
gi|780824870−Hyphomonadaceae_bacterium_BRH_c29
gi|737574901−Hyphomonas_atlantica
gi|780836634−Hyphomonas_sp__BRH_c22
gi|516885200−Henriciella_marina
gi|495524676−Zhongshania_aliphaticivorans
gi|496310536−Luminiphilus_syltensis_NOR5−1B
gi|949077464−OM182_bacterium_BACL3_MAG−120507−bin80
gi|949089893−OM182_bacterium_BACL3_MAG−120920−bin41
gi|949091356−OM182_bacterium_BACL3_MAG−120924−bin41
gi|949168388−OM182_bacterium_BACL3_MAG−120531−bin86
gi|948075768−Phenylobacterium_sp__Root700
gi|947338059−Phenylobacterium_sp__Root77
gi|501514191−Phenylobacterium_zucineum_HLK1
gi|960878210−Caulobacter_vibrioides
gi|737051708−Caulobacter_henricii
gi|947384927−Caulobacter_sp__Root343
gi|670499116−Caulobacteraceae_bacterium_PMMR1
gi|1012312681−Hyphomonadaceae_bacterium_UKL13−1
gi|780031439−Tepidicaulis_marinus
gi|657938522−alpha_proteobacterium_Mf_1_05b_01
gi|501060839−Parvibaculum_lavamentivorans_DS−1
gi|763067164−Candidatus_Phaeomarinobacter_ectocarpi
gi|119451265−marine_gamma_proteobacterium_HTCC2143
gi|494435501−marine_gamma_proteobacterium_HTCC2148
gi|494433552−marine_gamma_proteobacterium_HTCC2143
gi|1037236754−Rhizobiales_bacterium_NRL2
gi|918855690−Rhodopseudomonas_palustris
gi|763452745−Rhodopseudomonas_palustris
gi|489151162−Comamonas_testosteroni_KF−1
gi|220715139−Comamonas_testosteroni_KF−1
gi|1037237539−Rhizobiales_bacterium_NRL2
gi|522187390−Reyranella_massiliensis
gi|737542033−Pseudohaliea_rubra_DSM_19751
gi|737554289−Haliea_salexigens
gi|496313344−gamma_proteobacterium_NOR5−3
gi|495570788−Congregibacter_litoralis_KT71
gi|1053767013−Halioglobus_sp__HI00S01
gi|494434603−marine_gamma_proteobacterium_HTCC2148
gi|495524116−Zhongshania_aliphaticivorans
gi|654397396−Spongiibacter_marinus
gi|551338414−Spongiibacter_tropicus
gi|916316328−alpha_proteobacterium_JLT2015
gi|451959729−alpha_proteobacterium_JLT2015
gi|754708518−alpha_proteobacterium_Q−1
gi|501125818−Desulfococcus_oleovorans_Hxd3
gi|501639174−Desulfatibacillum_alkenivorans_AK−01
gi|654861559−Desulfatibacillum_aliphaticivorans
gi|816389050−Lokiarchaeum_sp__GC14_75
gi|931473946−Gammaproteobacteria_bacterium_SG8_31
gi|916688809−Flexibacter_elegans
gi|504807312−Alcanivorax_dieselolei
gi|1000087761−Thalassospira_sp__Nap_22
gi|931435771−Dehalococcoidia_bacterium_SM23_28_1
gi|931357246−Dehalococcoidia_bacterium_DG_22
gi|931463697−Dehalococcoidia_bacterium_SM23_28_2
gi|682436652−Pseudogymnoascus_sp__VKM_F−4519_FW−2642
gi|682389177−Pseudogymnoascus_sp__VKM_F−103
gi|915063581−Fonsecaea_pedrosoi_CBS_271_37
gi|1001605352−Gonapodya_prolifera_JEL478
gi|651330078−Actinopolyspora_iraqiensis
gi|815669897−Sphingomonas_sp__Ag1
gi|474490204−Desulfotignum_phosphitoxidans_DSM_13687
gi|748753270−Desulfotignum_phosphitoxidans
gi|636807398−Desulfotignum_balticum
GSMAST4A1_ASY1_ANO1_4028_1
gi|949047771−Acidimicrobiia_bacterium_BACL6_MAG−120924−bin43
gi|949053621−Acidimicrobiia_bacterium_BACL6_MAG−121220−bin61
gi|684290284−actinobacterium_acAcidi
gi|121282033−uncultured_bacterium_CBNPD1_BAC_clone_2089
gi|505253512−Ilumatobacter_coccineus_YM16−304
gi|916327675−Ilumatobacter_nonamiensis
gi|916593313−Candidatus_Microthrix_parvicella
gi|521046349−Candidatus_Microthrix_parvicella
gi|499279899|emb|CCM61794_1|−Candidatus_Microthrix_parvicella_RN1
gi|918748119−Actinobacteria_bacterium_IMCC26207
gi|1011354395−Endozoicomonas_sp__ab112
gi|736834471−Endozoicomonas_numazuensis
gi|745855284−marine_actinobacterium_MedAcidi−G2A
gi|745854427−marine_actinobacterium_MedAcidi−G3
gi|490123115−Gordonia_terrae
gi|1035733096−Gordonia_sp__LAM0048
gi|648280031−Rhodococcus_rhodochrous
gi|983497002−Rhodococcus_pyridinivorans_KG−16
gi|1039590703−Nocardia_sp__852002−20019_SCH5090214
gi|1039582076−Mycobacterium_sp__852002−51759_SCH5129042
gi|644662329−Nocardia_nova_SH22a
gi|502000594−Rhodococcus_opacus_B4
gi|516607125−Amycolatopsis_methanolica_239
gi|515253651−Streptomyces_viridosporus
gi|565879520−Williamsia_sp__D3
gi|1055238370−Millisia_brevis
gi|670523590−Sciscionella_sp__SE31
gi|521986974−Sciscionella_marina
gi|522155412−Amycolatopsis_nigrescens
gi|829599119−Actinobacteria_bacterium_IMCC26207
gi|829575678−Actinobacteria_bacterium_IMCC26256
0.1
Archaea
Bacteria
Eukaryota
SAG
NA

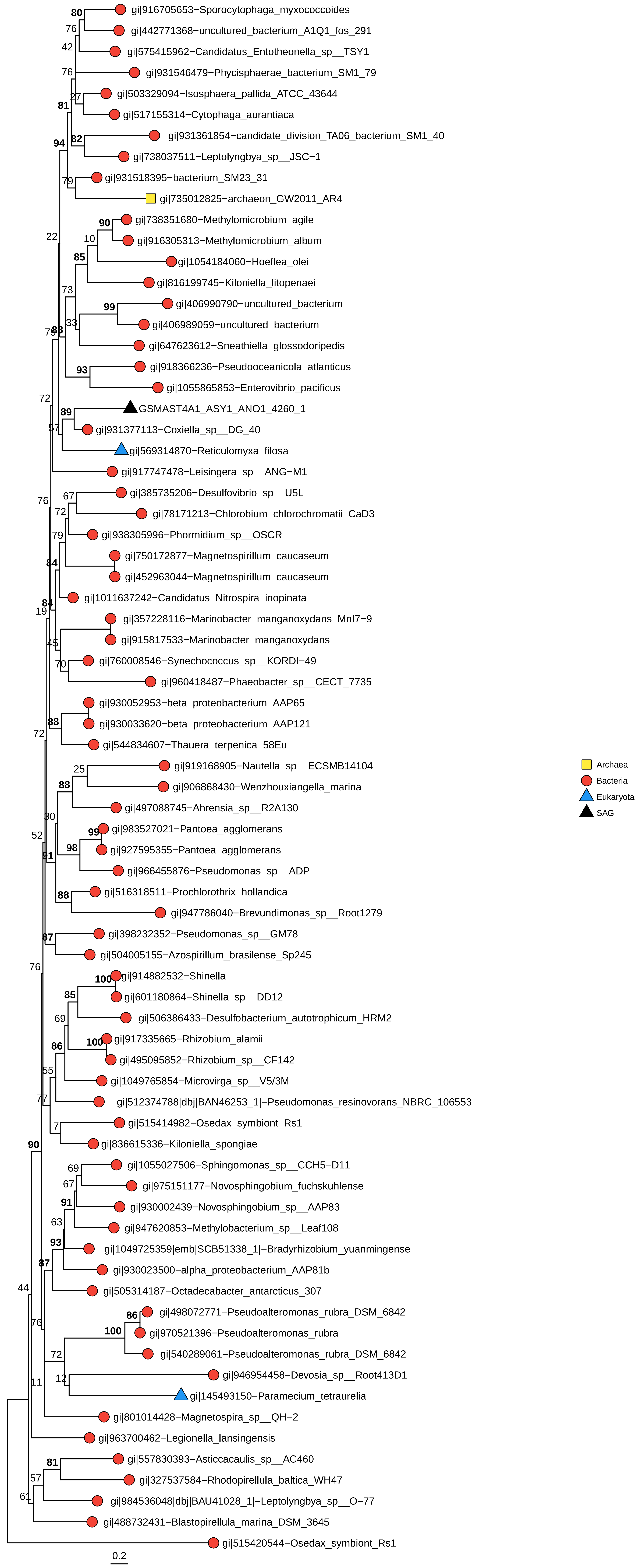
gi|916705653−Sporocytophaga_myxococcoides
gi|442771368−uncultured_bacterium_A1Q1_fos_291
gi|575415962−Candidatus_Entotheonella_sp__TSY1
gi|931546479−Phycisphaerae_bacterium_SM1_79
gi|503329094−Isosphaera_pallida_ATCC_43644
gi|517155314−Cytophaga_aurantiaca
gi|931361854−candidate_division_TA06_bacterium_SM1_40
gi|738037511−Leptolyngbya_sp__JSC−1
gi|931518395−bacterium_SM23_31
gi|735012825−archaeon_GW2011_AR4
gi|738351680−Methylomicrobium_agile
gi|916305313−Methylomicrobium_album
gi|1054184060−Hoeflea_olei
gi|816199745−Kiloniella_litopenaei
gi|406990790−uncultured_bacterium
gi|406989059−uncultured_bacterium
gi|647623612−Sneathiella_glossodoripedis
gi|918366236−Pseudooceanicola_atlanticus
gi|1055865853−Enterovibrio_pacificus
GSMAST4A1_ASY1_ANO1_4260_1
gi|931377113−Coxiella_sp__DG_40
gi|569314870−Reticulomyxa_filosa
gi|917747478−Leisingera_sp__ANG−M1
gi|385735206−Desulfovibrio_sp__U5L
gi|78171213−Chlorobium_chlorochromatii_CaD3
gi|938305996−Phormidium_sp__OSCR
gi|750172877−Magnetospirillum_caucaseum
gi|452963044−Magnetospirillum_caucaseum
gi|1011637242−Candidatus_Nitrospira_inopinata
gi|357228116−Marinobacter_manganoxydans_MnI7−9
gi|915817533−Marinobacter_manganoxydans
gi|760008546−Synechococcus_sp__KORDI−49
gi|960418487−Phaeobacter_sp__CECT_7735
gi|930052953−beta_proteobacterium_AAP65
gi|930033620−beta_proteobacterium_AAP121
gi|544834607−Thauera_terpenica_58Eu
gi|919168905−Nautella_sp__ECSMB14104
gi|906868430−Wenzhouxiangella_marina
gi|497088745−Ahrensia_sp__R2A130
gi|983527021−Pantoea_agglomerans
gi|927595355−Pantoea_agglomerans
gi|966455876−Pseudomonas_sp__ADP
gi|516318511−Prochlorothrix_hollandica
gi|947786040−Brevundimonas_sp__Root1279
gi|398232352−Pseudomonas_sp__GM78
gi|504005155−Azospirillum_brasilense_Sp245
gi|914882532−Shinella
gi|601180864−Shinella_sp__DD12
gi|506386433−Desulfobacterium_autotrophicum_HRM2
gi|917335665−Rhizobium_alamii
gi|495095852−Rhizobium_sp__CF142
gi|1049765854−Microvirga_sp__V5/3M
gi|512374788|dbj|BAN46253_1|−Pseudomonas_resinovorans_NBRC_106553
gi|515414982−Osedax_symbiont_Rs1
gi|836615336−Kiloniella_spongiae
gi|1055027506−Sphingomonas_sp__CCH5−D11
gi|975151177−Novosphingobium_fuchskuhlense
gi|930002439−Novosphingobium_sp__AAP83
gi|947620853−Methylobacterium_sp__Leaf108
gi|1049725359|emb|SCB51338_1|−Bradyrhizobium_yuanmingense
gi|930023500−alpha_proteobacterium_AAP81b
gi|505314187−Octadecabacter_antarcticus_307
gi|498072771−Pseudoalteromonas_rubra_DSM_6842
gi|970521396−Pseudoalteromonas_rubra
gi|540289061−Pseudoalteromonas_rubra_DSM_6842
gi|946954458−Devosia_sp__Root413D1
gi|145493150−Paramecium_tetraurelia
gi|801014428−Magnetospira_sp__QH−2
gi|963700462−Legionella_lansingensis
gi|557830393−Asticcacaulis_sp__AC460
gi|327537584−Rhodopirellula_baltica_WH47
gi|984536048|dbj|BAU41028_1|−Leptolyngbya_sp__O−77
gi|488732431−Blastopirellula_marina_DSM_3645
gi|515420544−Osedax_symbiont_Rs1
0.2
Archaea
Bacteria
Eukaryota
SAG

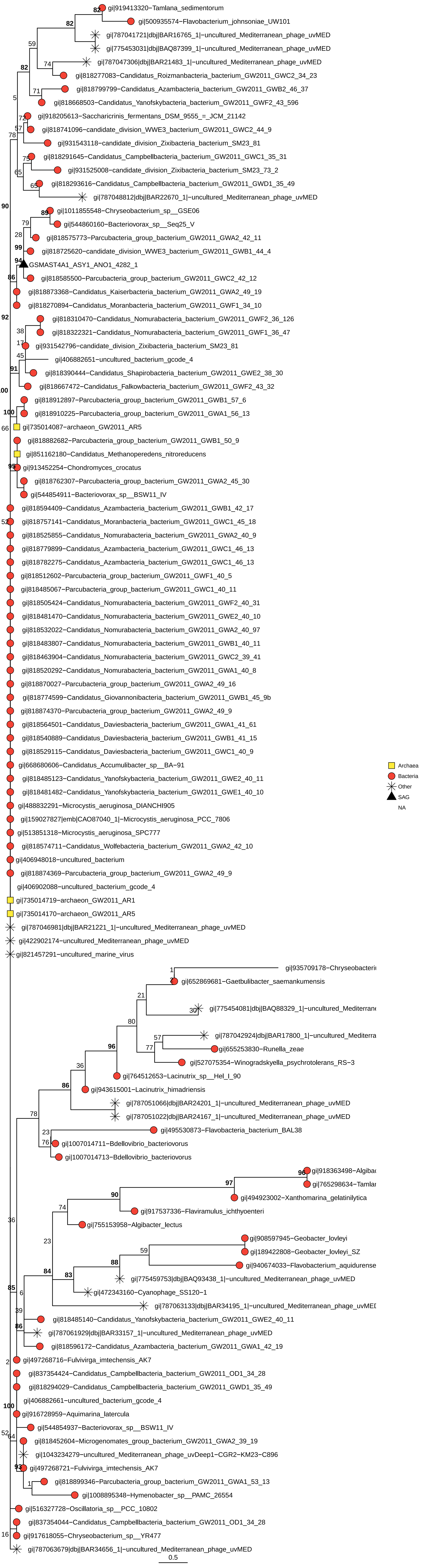

gi|919413320−Tamlana_sedimentorum
gi|500935574−Flavobacterium_johnsoniae_UW101
gi|787041721|dbj|BAR16765_1|−uncultured_Mediterranean_phage_uvMED
gi|775453031|dbj|BAQ87399_1|−uncultured_Mediterranean_phage_uvMED
gi|787047306|dbj|BAR21483_1|−uncultured_Mediterranean_phage_uvMED
gi|818277083−Candidatus_Roizmanbacteria_bacterium_GW2011_GWC2_34_23
gi|818799799−Candidatus_Azambacteria_bacterium_GW2011_GWB2_46_37
gi|818668503−Candidatus_Yanofskybacteria_bacterium_GW2011_GWF2_43_596
gi|918205613−Saccharicrinis_fermentans_DSM_9555_=_JCM_21142
gi|818741096−candidate_division_WWE3_bacterium_GW2011_GWC2_44_9
gi|931543118−candidate_division_Zixibacteria_bacterium_SM23_81
gi|818291645−Candidatus_Campbellbacteria_bacterium_GW2011_GWC1_35_31
gi|931525008−candidate_division_Zixibacteria_bacterium_SM23_73_2
gi|818293616−Candidatus_Campbellbacteria_bacterium_GW2011_GWD1_35_49
gi|787048812|dbj|BAR22670_1|−uncultured_Mediterranean_phage_uvMED
gi|1011855548−Chryseobacterium_sp__GSE06
gi|544860160−Bacteriovorax_sp__Seq25_V
gi|818575773−Parcubacteria_group_bacterium_GW2011_GWA2_42_11
gi|818725620−candidate_division_WWE3_bacterium_GW2011_GWB1_44_4
GSMAST4A1_ASY1_ANO1_4282_1
gi|818585500−Parcubacteria_group_bacterium_GW2011_GWC2_42_12
gi|818873368−Candidatus_Kaiserbacteria_bacterium_GW2011_GWA2_49_19
gi|818270894−Candidatus_Moranbacteria_bacterium_GW2011_GWF1_34_10
gi|818310470−Candidatus_Nomurabacteria_bacterium_GW2011_GWF2_36_126
gi|818322321−Candidatus_Nomurabacteria_bacterium_GW2011_GWF1_36_47
gi|931542796−candidate_division_Zixibacteria_bacterium_SM23_81
gi|406882651−uncultured_bacterium_gcode_4
gi|818390444−Candidatus_Shapirobacteria_bacterium_GW2011_GWE2_38_30
gi|818667472−Candidatus_Falkowbacteria_bacterium_GW2011_GWF2_43_32
gi|818912897−Parcubacteria_group_bacterium_GW2011_GWB1_57_6
gi|818910225−Parcubacteria_group_bacterium_GW2011_GWA1_56_13
gi|735014087−archaeon_GW2011_AR5
gi|818882682−Parcubacteria_group_bacterium_GW2011_GWB1_50_9
gi|851162180−Candidatus_Methanoperedens_nitroreducens
gi|913452254−Chondromyces_crocatus
gi|818762307−Parcubacteria_group_bacterium_GW2011_GWA2_45_30
gi|544854911−Bacteriovorax_sp__BSW11_IV
gi|818594409−Candidatus_Azambacteria_bacterium_GW2011_GWB1_42_17
gi|818757141−Candidatus_Moranbacteria_bacterium_GW2011_GWC1_45_18
gi|818525855−Candidatus_Nomurabacteria_bacterium_GW2011_GWA2_40_9
gi|818779899−Candidatus_Azambacteria_bacterium_GW2011_GWC1_46_13
gi|818782275−Candidatus_Azambacteria_bacterium_GW2011_GWC1_46_13
gi|818512602−Parcubacteria_group_bacterium_GW2011_GWF1_40_5
gi|818485067−Parcubacteria_group_bacterium_GW2011_GWC1_40_11
gi|818505424−Candidatus_Nomurabacteria_bacterium_GW2011_GWF2_40_31
gi|818481470−Candidatus_Nomurabacteria_bacterium_GW2011_GWE2_40_10
gi|818532022−Candidatus_Nomurabacteria_bacterium_GW2011_GWA2_40_97
gi|818483807−Candidatus_Nomurabacteria_bacterium_GW2011_GWB1_40_11
gi|818463904−Candidatus_Nomurabacteria_bacterium_GW2011_GWC2_39_41
gi|818520292−Candidatus_Nomurabacteria_bacterium_GW2011_GWA1_40_8
gi|818870027−Parcubacteria_group_bacterium_GW2011_GWA2_49_16
gi|818774599−Candidatus_Giovannonibacteria_bacterium_GW2011_GWB1_45_9b
gi|818874370−Parcubacteria_group_bacterium_GW2011_GWA2_49_9
gi|818564501−Candidatus_Daviesbacteria_bacterium_GW2011_GWA1_41_61
gi|818540889−Candidatus_Daviesbacteria_bacterium_GW2011_GWB1_41_15
gi|818529115−Candidatus_Daviesbacteria_bacterium_GW2011_GWC1_40_9
gi|668680606−Candidatus_Accumulibacter_sp__BA−91
gi|818485123−Candidatus_Yanofskybacteria_bacterium_GW2011_GWE2_40_11
gi|818481482−Candidatus_Yanofskybacteria_bacterium_GW2011_GWE1_40_10
gi|488832291−Microcystis_aeruginosa_DIANCHI905
gi|159027827|emb|CAO87040_1|−Microcystis_aeruginosa_PCC_7806
gi|513851318−Microcystis_aeruginosa_SPC777
gi|818574711−Candidatus_Wolfebacteria_bacterium_GW2011_GWA2_42_10
gi|406948018−uncultured_bacterium
gi|818874369−Parcubacteria_group_bacterium_GW2011_GWA2_49_9
gi|406902088−uncultured_bacterium_gcode_4
gi|735014719−archaeon_GW2011_AR1
gi|735014170−archaeon_GW2011_AR5
gi|787046981|dbj|BAR21221_1|−uncultured_Mediterranean_phage_uvMED
gi|422902174−uncultured_Mediterranean_phage_uvMED
gi|821457291−uncultured_marine_virus
gi|935709178−Chryseobacteri
gi|652869681−Gaetbulibacter_saemankumensis
gi|775454081|dbj|BAQ88329_1|−uncultured_Mediterrane
gi|787042924|dbj|BAR17800_1|−uncultured_Mediterra
gi|655253830−Runella_zeae
gi|527075354−Winogradskyella_psychrotolerans_RS−3
gi|764512653−Lacinutrix_sp__Hel_I_90
gi|943615001−Lacinutrix_himadriensis
gi|787051066|dbj|BAR24201_1|−uncultured_Mediterranean_phage_uvMED
gi|787051022|dbj|BAR24167_1|−uncultured_Mediterranean_phage_uvMED
gi|495530873−Flavobacteria_bacterium_BAL38
gi|1007014711−Bdellovibrio_bacteriovorus
gi|1007014713−Bdellovibrio_bacteriovorus
gi|918363498−Algibac
gi|765298634−Tamlar
gi|494923002−Xanthomarina_gelatinilytica
gi|917537336−Flaviramulus_ichthyoenteri
gi|755153958−Algibacter_lectus
gi|908597945−Geobacter_lovleyi
gi|189422808−Geobacter_lovleyi_SZ
gi|940674033−Flavobacterium_aquidurense
gi|775459753|dbj|BAQ93438_1|−uncultured_Mediterranean_phage_uvMED
gi|472343160−Cyanophage_SS120−1
gi|787063133|dbj|BAR34195_1|−uncultured_Mediterranean_phage_uvMEI
gi|818485140−Candidatus_Yanofskybacteria_bacterium_GW2011_GWE2_40_11
gi|787061929|dbj|BAR33157_1|−uncultured_Mediterranean_phage_uvMED
gi|818596172−Candidatus_Azambacteria_bacterium_GW2011_GWA1_42_19
gi|497268716−Fulvivirga_imtechensis_AK7
gi|837354424−Candidatus_Campbellbacteria_bacterium_GW2011_OD1_34_28
gi|818294029−Candidatus_Campbellbacteria_bacterium_GW2011_GWD1_35_49
gi|406882661−uncultured_bacterium_gcode_4
gi|916728959−Aquimarina_latercula
gi|544854937−Bacteriovorax_sp__BSW11_IV
gi|818452604−Microgenomates_group_bacterium_GW2011_GWA2_39_19
gi|1043234279−uncultured_Mediterranean_phage_uvDeep1−CGR2−KM23−C896
gi|497268721−Fulvivirga_imtechensis_AK7
gi|818899346−Parcubacteria_group_bacterium_GW2011_GWA1_53_13
gi|1008895348−Hymenobacter_sp__PAMC_26554
gi|516327728−Oscillatoria_sp__PCC_10802
gi|837354044−Candidatus_Campbellbacteria_bacterium_GW2011_OD1_34_28
gi|917618055−Chryseobacterium_sp__YR477
gi|787063679|dbj|BAR34656_1|−uncultured_Mediterranean_phage_uvMED
0.5
Archaea
Bacteria
Other
SAG
NA

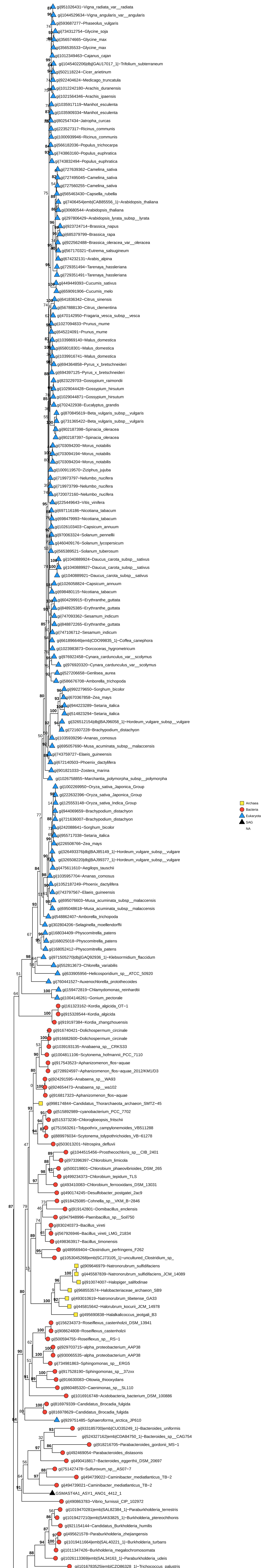
Archaea
Bacteria
Eukaryota
SAG
NA
0.2

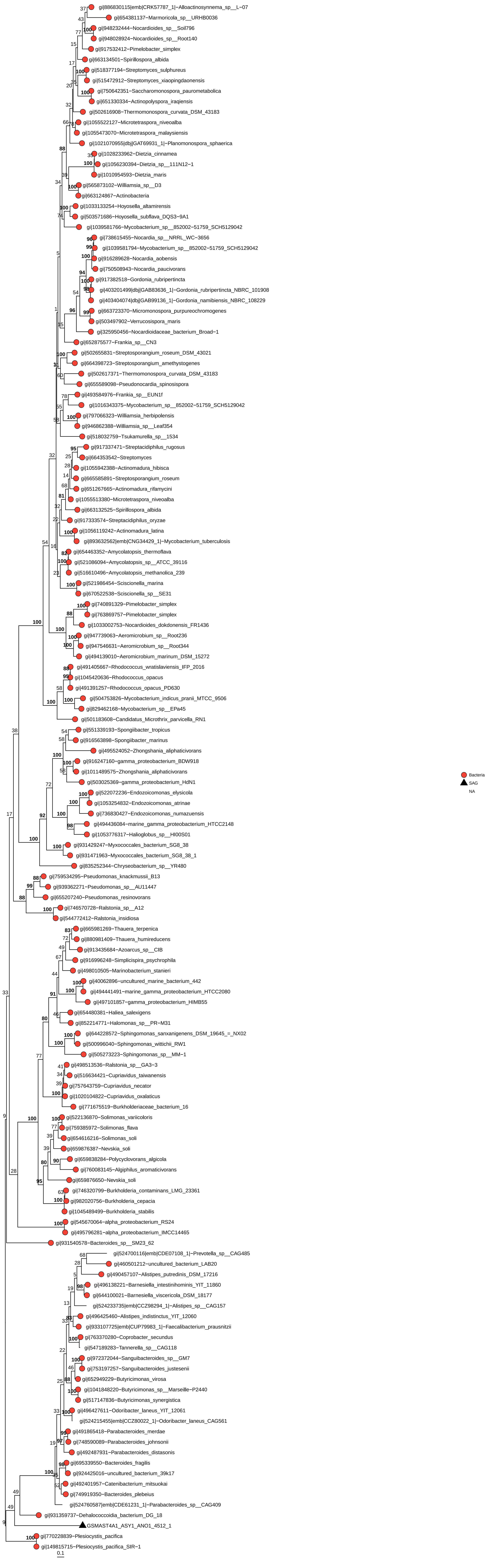

gi|886830115|emb|CRK57787_1|~Alloactinosynnema_sp__L~07
gi|654381137~Marmoricola_sp__URHB0036
gi|948232444~Nocardioides_sp__Soil796
gi|948028924~Nocardioides_sp__Root140
gi|917532412~Pimelobacter_simplex
gi|663134501~Spirillospora_albida
gi|518377194~Streptomyces_sulphureus
gi|515472912~Streptomyces_xiaopingdaonensis
gi|750642351~Saccharomonospora_paurometabolica
gi|651330334~Actinopolyspora_iraqiensis
gi|502616908~Thermomonospora_curvata_DSM_43183
gi|1055522127~Microtetraspora_niveoalba
gi|1055473070~Microtetraspora_malaysiensis
gi|1021070955|dbj|GAT69931_1|~Planomonospora_sphaerica
gi|1028233962~Dietzia_cinnamea
gi|1056230394~Dietzia_sp__111N12~1
gi|1010954593~Dietzia_maris
gi|565873102~Williamsia_sp__D3
gi|663124867~Actinobacteria
gi|1033133254~Hoyosella_altamirensis
gi|503571686~Hoyosella_subflava_DQS3~9A1
gi|1039581766~Mycobacterium_sp__852002~51759_SCH5129042
gi|738615455~Nocardia_sp__NRRL_WC~3656
gi|1039581794~Mycobacterium_sp__852002~51759_SCH5129042
gi|916289628~Nocardia_aobensis
gi|750508943~Nocardia_paucivorans
gi|917382518~Gordonia_rubripertincta
gi|403201499|dbj|GAB83636_1|~Gordonia_rubripertincta_NBRC_101908
gi|403404074|dbj|GAB99136_1|~Gordonia_namibiensis_NBRC_108229
gi|663723370~Micromonospora_purpureochromogenes
gi|503497902~Verrucosispora_maris
gi|325950456~Nocardioidaceae_bacterium_Broad~1
gi|652875577~Frankia_sp__CN3
gi|502655831~Streptosporangium_roseum_DSM_43021
gi|664398723~Streptosporangium_amethystogenes
gi|502617371~Thermomonospora_curvata_DSM_43183
gi|655589098~Pseudonocardia_spinosispora
gi|493584976~Frankia_sp__EUN1f
gi|1016343375~Mycobacterium_sp__852002~51759_SCH5129042
gi|797066323~Williamsia_herbipolensis
gi|946862388~Williamsia_sp__Leaf354
gi|518032759~Tsukamurella_sp__1534
gi|917337471~Streptacidiphilus_rugosus
gi|664353542~Streptomyces
gi|1055942388~Actinomadura_hibisca
gi|665585891~Streptosporangium_roseum
gi|651267665~Actinomadura_rifamycini
gi|1055513380~Microtetraspora_niveoalba
gi|663132525~Spirillospora_albida
gi|917333574~Streptacidiphilus_oryzae
gi|1056119242~Actinomadura_latina
gi|893632562|emb|CNG34429_1|~Mycobacterium_tuberculosis
gi|654463352~Amycolatopsis_thermoflava
gi|521086094~Amycolatopsis_sp__ATCC_39116
gi|516610496~Amycolatopsis_methanolica_239
gi|521986454~Sciscionella_marina
gi|670522538~Sciscionella_sp__SE31
gi|740891329~Pimelobacter_simplex
gi|763869757~Pimelobacter_simplex
gi|1033002753~Nocardioides_dokdonensis_FR1436
gi|947739063~Aeromicrobium_sp__Root236
gi|947546631~Aeromicrobium_sp__Root344
gi|494139010~Aeromicrobium_marinum_DSM_15272
gi|491405667~Rhodococcus_wratislaviensis_IFP_2016
gi|1045420636~Rhodococcus_opacus
gi|491391257~Rhodococcus_opacus_PD630
gi|504753826~Mycobacterium_indicus_pranii_MTCC_9506
gi|829462168~Mycobacterium_sp__EPa45
gi|501183608~Candidatus_Microthrix_parvicella_RN1
gi|551339193~Spongiibacter_tropicus
gi|916563898~Spongiibacter_marinus
gi|495524052~Zhongshania_aliphaticivorans
gi|916247160~gamma_proteobacterium_BDW918
gi|1011489575~Zhongshania_aliphaticivorans
gi|503025369~gamma_proteobacterium_HdN1
gi|522072236~Endozoicomonas_elysicola
gi|1053254832~Endozoicomonas_atrinae
gi|736830427~Endozoicomonas_numazuensis
gi|494436084~marine_gamma_proteobacterium_HTCC2148
gi|1053776317~Halioglobus_sp__HI00S01
gi|931429247~Myxococcales_bacterium_SG8_38
gi|931471963~Myxococcales_bacterium_SG8_38_1
gi|835252344~Chryseobacterium_sp__YR480
gi|759534295~Pseudomonas_knackmussii_B13
gi|939362271~Pseudomonas_sp__AU11447
gi|655207240~Pseudomonas_resinovorans
gi|746570728~Ralstonia_sp__A12
gi|544772412~Ralstonia_insidiosa
gi|665981269~Thauera_terpenica
gi|880981409~Thauera_humireducens
gi|913435684~Azoarcus_sp__CIB
gi|916996248~Simplicispira_psychrophila
gi|498010505~Marinobacterium_stanieri
gi|40062896~uncultured_marine_bacterium_442
gi|494441491~marine_gamma_proteobacterium_HTCC2080
gi|497101857~gamma_proteobacterium_HIMB55
gi|654480381~Haliea_salexigens
gi|852214771~Halomonas_sp__PR~M31
gi|644228572~Sphingomonas_sanxanigenens_DSM_19645_=_NX02
gi|500996040~Sphingomonas_wittichii_RW1
gi|505273223~Sphingomonas_sp__MM~1
gi|498513536~Ralstonia_sp__GA3~3
gi|516634421~Cupriavidus_taiwanensis
gi|757643759~Cupriavidus_necator
gi|1020104822~Cupriavidus_oxalaticus
gi|771675519~Burkholderiaceae_bacterium_16
gi|522136870~Solimonas_variicoloris
gi|759385972~Solimonas_flava
gi|654616216~Solimonas_soli
gi|659876387~Nevskia_soli
gi|659838284~Polycyclovorans_algicola
gi|760083145~Algiphilus_aromaticivorans
gi|659876650~Nevskia_soli
gi|746320799~Burkholderia_contaminans_LMG_23361
gi|982020756~Burkholderia_cepacia
gi|1045489499~Burkholderia_stabilis
gi|545670064~alpha_proteobacterium_RS24
gi|495796281~alpha_proteobacterium_IMCC14465
gi|931540578~Bacteroides_sp__SM23_62
gi|524700116|emb|CDE07108_1|~Prevotella_sp__CAG485
gi|460501212~uncultured_bacterium_LAB20
gi|490457107~Alistipes_putredinis_DSM_17216
gi|496138221~Barnesiella_intestinihominis_YIT_11860
gi|644100021~Barnesiella_viscericola_DSM_18177
gi|524233735|emb|CCZ98294_1|~Alistipes_sp__CAG157
gi|496425460~Alistipes_indistinctus_YIT_12060
gi|933107725|emb|CUP79983_1|~Faecalibacterium_prausnitzii
gi|763370280~Coprobacter_secundus
gi|547189283~Tannerella_sp__CAG118
gi|972372044~Sanguibacteroides_sp__GM7
gi|753197257~Sanguibacteroides_justesenii
gi|652949229~Butyricimonas_virosa
gi|1041848220~Butyricimonas_sp__Marseille~P2440
gi|517147836~Butyricimonas_synergistica
gi|496427611~Odoribacter_laneus_YIT_12061
gi|524215455|emb|CCZ80022_1|~Odoribacter_laneus_CAG561
gi|491865418~Parabacteroides_merdae
gi|748590089~Parabacteroides_johnsonii
gi|492487931~Parabacteroides_distasonis
gi|695339550~Bacteroides_fragilis
gi|924425016~uncultured_bacterium_39k17
gi|492401957~Catenibacterium_mitsuokai
gi|749919350~Bacteroides_plebeius
gi|524760587|emb|CDE61231_1|~Parabacteroides_sp__CAG409
gi|931359737~Dehalococcoidia_bacterium_DG_18
GSMAST4A1_ASY1_ANO1_4512_1
gi|770228839~Plesiocystis_pacifica
gi|149815715~Plesiocystis_pacifica_SIR~1
0.1
Bacteria
SAG
NA

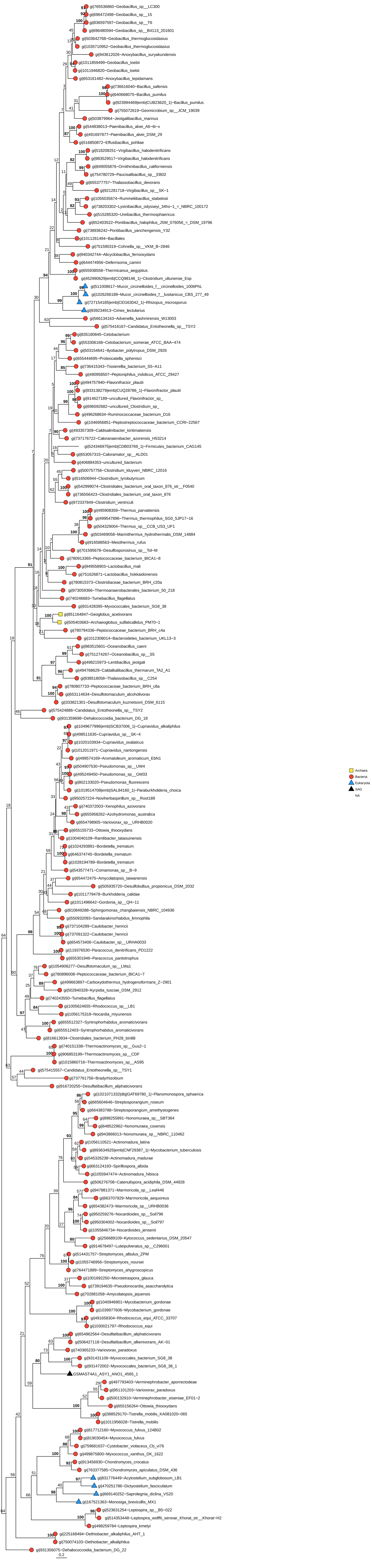

Archaea
Bacteria
Eukaryota
SAG
NA
0.2

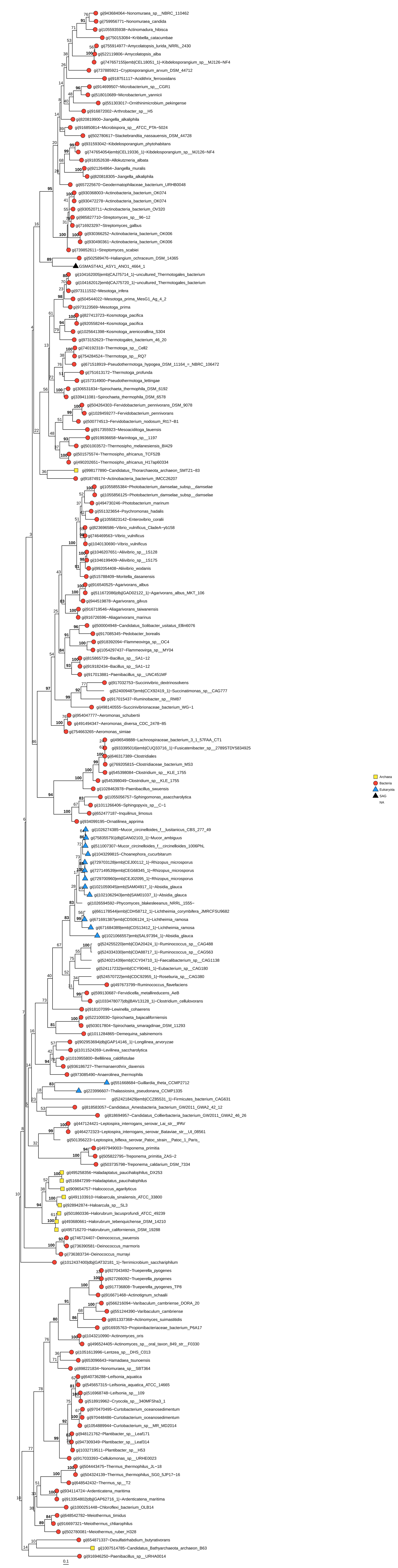
Archaea
Bacteria
Eukaryota
SAG
NA
0.1

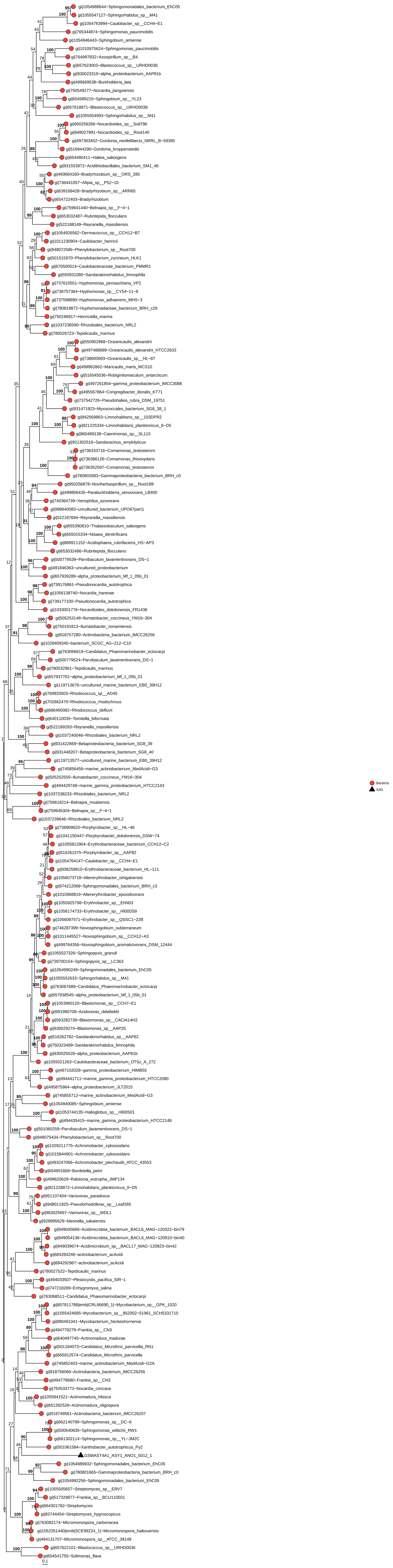

GSMAST4A1_ASY1_ANO1_5012_1
0.1
Bacteria
SAG

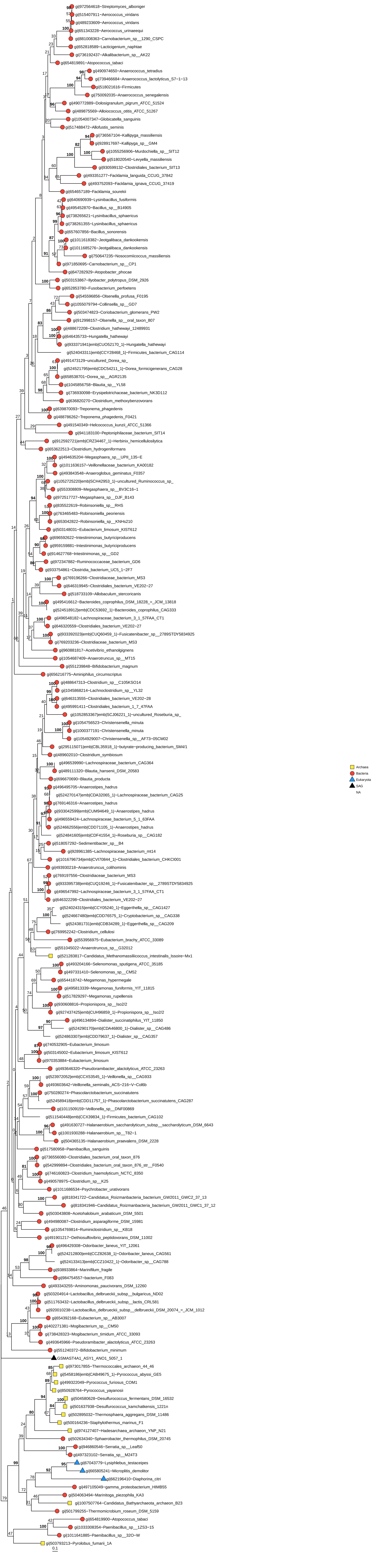

Archaea
Bacteria
Eukaryota
SAG
NA
gi|972564618~Streptomyces_albonigер
gi|515407911~Aerococcus_viridans
gi|489233609~Aerococcus_viridans
gi|651343228~Aerococcus_urinaeequi
gi|881008363~Carnobacterium_sp__1290_CSPC
gi|652818589~Lacticigenium_naphtae
gi|736192437~Alkalibacterium_sp__AK22
gi|654819891~Atopococcus_tabaci
gi|490974650~Anaerococcus_tetradius
gi|739466684~Anaerococcus_lactolyticus_S7-1-13
gi|518021616~Firmicutes
gi|750092035~Anaerococcus_senegalensis
gi|490772889~Dolosigranulum_pigrum_ATCC_51524
gi|489875569~Alloiococcus_otitis_ATCC_51267
gi|1054007347~Globicatella_sanguinis
gi|517488472~Allofustis_seminis
gi|736567104~Kallipyga_massiliensis
gi|928917697~Kallipyga_sp__GM4
gi|1055256906~Murdochiella_sp__SIT12
gi|518020540~Levyella_massiliensis
gi|930599132~Clostridiales_bacterium_SIT13
gi|493351277~Facklamia_languida_CCUG_37842
gi|493752093~Facklamia_ignava_CCUG_37419
gi|654657189~Facklamia_sourekii
gi|640690939~Lysinibacillus_fusiformis
gi|495452870~Bacillus_sp__B14905
gi|738265621~Lysinibacillus_sphaericus
gi|738261355~Lysinibacillus_sphaericus
gi|657607856~Bacillus_sonorensis
gi|1011618382~Jeotgalibaca_dankookensis
gi|1011685276~Jeotgalibaca_dankookensis
gi|750647235~Nosocomiicoccus_massiliensis
gi|971850695~Carnobacterium_sp__CP1
gi|647282929~Atopobacter_phocae
gi|503153867~Ilyobacter_polytropus_DSM_2926
gi|652853780~Fusobacterium_perfoetens
gi|545596856~Olsenella_profusa_F0195
gi|1055079794~Collinsella_sp__GD7
gi|503474823~Coriobacterium_glomerans_PW2
gi|912998157~Olsenella_sp__oral_taxon_807
gi|488672208~Clostridium_hathewayi_12489931
gi|646435733~Hungatella_hathewayi
gi|933371941|emb|CUO52170_1|~Hungatella_hathewayi
gi|524043311|emb|CCY28468_1|~Firmicutes_bacterium_CAG114
gi|491473129~uncultured_Dorea_sp_
gi|524521795|emb|CDC54211_1|~Dorea_formicigenerans_CAG28
gi|658538701~Dorea_sp__AGR2135
gi|1045856758~Blautia_sp__YL58
gi|736930098~Erysipelotrichaceae_bacterium_NK3D112
gi|636820270~Clostridium_methoxybenzovorans
gi|639870093~Treponema_phagedenis
gi|488786262~Treponema_phagedenis_F0421
gi|491540349~Helcococcus_kunzii_ATCC_51366
gi|941183100~Peptoniphilaceae_bacterium_SIT14
gi|912592721|emb|CRZ34467_1|~Herbinix_hemicellulosilytica
gi|653622513~Clostridium_hydrogeniformans
gi|494635204~Megasphaera_sp__UPII_135-E
gi|1011636157~Veillonellaceae_bacterium_KA00182
gi|493843548~Anaeroglobus_geminatus_F0357
gi|1052725220|emb|SCH42953_1|~uncultured_Ruminococcus_sp_
gi|553308809~Megasphaera_sp__BV3C16-1
gi|972517727~Megasphaera_sp__DJF_B143
gi|835522619~Robinsoniella_sp__RHS
gi|763465483~Robinsoniella_peoriensis
gi|653042822~Robinsoniella_sp__KNHs210
gi|503148031~Eubacterium_limosum_KIST612
gi|696592622~Intestinimonas_butyriciproducens
gi|959159881~Intestinimonas_butyriciproducens
gi|914627768~Intestinimonas_sp__GD2
gi|972347882~Ruminococcaceae_bacterium_GD6
gi|933754861~Clostridia_bacterium_UC5_1-2F7
gi|769196266~Clostridiaceae_bacterium_MS3
gi|646319945~Clostridiales_bacterium_VE202-27
gi|518733109~Allobaculum_stercoricanis
gi|495416612~Bacteroides_coprophilus_DSM_18228_=_JCM_13818
gi|524518912|emb|CDC53692_1|~Bacteroides_coprophilus_CAG333
gi|496548182~Lachnospiraceae_bacterium_3_1_57FAA_CT1
gi|646320559~Clostridiales_bacterium_VE202-27
gi|933392023|emb|CUQ60459_1|~Fusicatenibacter_sp__2789STDY5834925
gi|769203236~Clostridiaceae_bacterium_MS3
gi|960881817~Acetivibrio_ethanolgignens
gi|1054687409~Anaerotruncus_sp__MT15
gi|551239848~Bifidobacterium_magnum
gi|656216775~Aminiphilus_circumscriptus
gi|488647313~Clostridium_sp__C105KSO14
gi|1045868214~Lachnoclostridium_sp__YL32
gi|646313555~Clostridiales_bacterium_VE202-28
gi|495991411~Clostridiales_bacterium_1_7_47FAA
gi|1052853367|emb|SCJ06221_1|~uncultured_Roseburia_sp_
gi|1054756523~Christensenella_minuta
gi|1000377191~Christensenella_minuta
gi|1054929007~Christensenella_sp__AF73-05CM02
gi|295115071|emb|CBL35918_1|~butyrate-producing_bacterium_SM4/1
gi|489602010~Clostridium_symbiosum
gi|496539990~Lachnospiraceae_bacterium_CAG364
gi|489111320~Blautia_hansenii_DSM_20583
gi|696670690~Blautia_producta
gi|496495705~Anaerostipes_hadrus
gi|524270147|emb|CDA32065_1|~Lachnospiraceae_bacterium_CAG25
gi|769146316~Anaerostipes_hadrus
gi|933042599|emb|CUM94649_1|~Anaerostipes_hadrus
gi|496559424~Lachnospiraceae_bacterium_5_1_63FAA
gi|524662556|emb|CDD71105_1|~Anaerostipes_hadrus
gi|524841605|emb|CDF41554_1|~Roseburia_sp__CAG182
gi|518057292~Sedimentibacter_sp__B4
gi|928961385~Lachnospiraceae_bacterium_mt14
gi|1016796734|emb|CVI70844_1|~Clostridiales_bacterium_CHKCI001
gi|493930218~Anaerotruncus_colihominis
gi|769197556~Clostridiaceae_bacterium_MS3
gi|933395738|emb|CUQ19246_1|~Fusicatenibacter_sp__2789STDY5834925
gi|496547992~Lachnospiraceae_bacterium_3_1_57FAA_CT1
gi|646322298~Clostridiales_bacterium_VE202-27
gi|524024315|emb|CCY05240_1|~Eggerthella_sp__CAG1427
gi|524667480|emb|CDD76575_1|~Cryptobacterium_sp__CAG338
gi|524381731|emb|CDB34289_1|~Eggerthella_sp__CAG209
gi|769952242~Clostridium_cellulosi
gi|553956975~Eubacterium_brachy_ATCC_33089
gi|551045022~Anaerotruncus_sp__G32012
gi|521283817~Candidatus_Methanomassiliicoccus_intestinalis_Issoire-Mx1
gi|493204166~Selenomonas_sputigena_ATCC_35185
gi|497331410~Selenomonas_sp__CM52
gi|654418742~Megamonas_hypermegale
gi|495813339~Megamonas_funiformis_YIT_11815
gi|517829297~Megamonas_rupellensis
gi|930608816~Propionispora_sp__2/2
gi|927437425|emb|CUH96859_1|~Propionispora_sp__Iso2/2
gi|496134894~Dialister_succinatiphilus_YIT_11850
gi|524290170|emb|CDA46800_1|~Dialister_sp__CAG486
gi|524863307|emb|CDD79637_1|~Dialister_sp__CAG357
gi|740532905~Eubacterium_limosum
gi|503145002~Eubacterium_limosum_KIST612
gi|970353884~Eubacterium_limosum
gi|493646320~Pseudoramibacter_alactolyticus_ATCC_23263
gi|523972052|emb|CCX53545_1|~Veillonella_sp__CAG933
gi|493603642~Veillonella_seminalis_ACS-216-V-Col6b
gi|750280274~Phascolarctobacterium_succinatutens
gi|524589418|emb|CDD11757_1|~Phascolarctobacterium_succinatutens_CAG287
gi|1011509159~Veillonella_sp__DNF00869
gi|511540448|emb|CCX39834_1|~Firmicutes_bacterium_CAG102
gi|491630727~Halanaerobium_saccharolyticum_subsp__saccharolyticum_DSM_6643
gi|1001930288~Halanaerobium_sp__T82-1
gi|504365135~Halanaerobium_praevalens_DSM_2228
gi|517580958~Paenibacillus_sanguinis
gi|736556080~Clostridiales_bacterium_oral_taxon_876
gi|542999894~Clostridiales_bacterium_oral_taxon_876_str__F0540
gi|746160823~Clostridium_haemolyticum_NCTC_8350
gi|490578975~Clostridium_sp__K25
gi|1011686534~Psychrobacter_urativorans
gi|818341722~Candidatus_Roizmanbacteria_bacterium_GW2011_GWC2_37_13
gi|818341946~Candidatus_Roizmanbacteria_bacterium_GW2011_GWC1_37_12
gi|503043808~Acetohalobium_arabaticum_DSM_5501
gi|494980087~Clostridium_asparagiforme_DSM_15981
gi|1054769814~Ruminiclostridium_sp__KB18
gi|491901217~Dethiosulfovibrio_peptidovorans_DSM_11002
gi|496429308~Odoribacter_laneus_YIT_12061
gi|524212800|emb|CCZ82638_1|~Odoribacter_laneus_CAG561
gi|524133413|emb|CCZ10422_1|~Odoribacter_sp__CAG788
gi|938933864~Marinifilum_fragile
gi|984754557~bacterium_F083
gi|493343255~Aminomonas_paucivorans_DSM_12260
gi|503204914~Lactobacillus_delbrueckii_subsp__bulgaricus_ND02
gi|511763432~Lactobacillus_delbrueckii_subsp__lactis_CRL581
gi|920010238~Lactobacillus_delbrueckii_subsp__delbrueckii_DSM_20074_=_JCM_1012
gi|654392168~Eubacterium_sp__AB3007
gi|402271381~Mogibacterium_sp__CM50
gi|738428323~Mogibacterium_timidum_ATCC_33093
gi|493645966~Pseudoramibacter_alactolyticus_ATCC_23263
gi|551240372~Bifidobacterium_minimum
GSMAST4A1_ASY1_ANO1_5057_1
gi|973017855~Thermococcales_archaeon_44_46
gi|5458186|emb|CAB49675_1|~Pyrococcus_abyssi_GE5
gi|499322049~Pyrococcus_furiosus_COM1
gi|850928764~Pyrococcus_yayanosii
gi|504580628~Desulfurococcus_fermentans_DSM_16532
gi|501637938~Desulfurococcus_kamchatkensis_1221n
gi|502895032~Thermosphaera_aggregans_DSM_11486
gi|500164236~Staphylothermus_marinus_F1
gi|974127407~Hadesarchaea_archaeon_YNP_N21
gi|502634340~Sphaerobacter_thermophilus_DSM_20745
gi|946860546~Serratia_sp__Leaf50
gi|497323102~Serratia_sp__M24T3
gi|67043779~Lysiphlebus_testaceipes
gi|665805241~Microplitis_demolitor
gi|662196410~Diaphorina_citri
gi|497105049~gamma_proteobacterium_HIMB55
gi|504063494~Marinitoga_piezophila_KA3
gi|1007507764~Candidatus_Bathyarchaeota_archaeon_B23
gi|501799255~Thermomicrobium_roseum_DSM_5159
gi|654819900~Atopococcus_tabaci
gi|1033308354~Paenibacillus_sp__1ZS3-15
gi|1011641885~Paenibacillus_sp__32O-W
gi|503793213~Pyrolobus_fumarii_1A
0.1

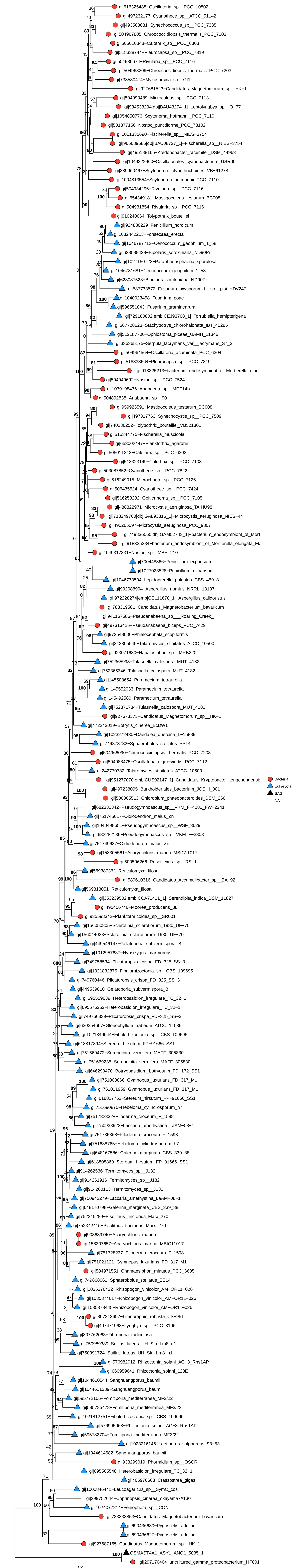
gi|516325488~Oscillatoria_sp__PCC_10802
gi|497232177~Cyanothece_sp__ATCC_51142
gi|493503631~Synechococcus_sp__PCC_7335
gi|504967805~Chroococcidiopsis_thermalis_PCC_7203
gi|505010848~Calothrix_sp__PCC_6303
gi|518338744~Pleurocapsa_sp__PCC_7319
gi|504930674~Rivularia_sp__PCC_7116
gi|504968209~Chroococcidiopsis_thermalis_PCC_7203
gi|738530474~Myxosarcina_sp__GI1
gi|927681523~Candidatus_Magnetomorum_sp__HK-1
gi|504993489~Microcoleus_sp__PCC_7113
gi|984538294|dbj|BAU43274_1|~Leptolyngbya_sp__O-77
gi|1054850776~Scytonema_hofmannii_PCC_7110
gi|501377156~Nostoc_punctiforme_PCC_73102
gi|1011335690~Fischerella_sp__NIES-3754
gi|965689585|dbj|BAU08727_1|~Fischerella_sp__NIES-3754
gi|495188165~Ktedonobacter_racemifer_DSM_44963
gi|1004322960~Oscillatoriales_cyanobacterium_USR001
gi|889960467~Scytonema_tolypothrichoides_VB-61278
gi|1004813554~Scytonema_hofmannii_PCC_7110
gi|504934296~Rivularia_sp__PCC_7116
gi|654349181~Mastigocoleus_testarum_BC008
gi|504931854~Rivularia_sp__PCC_7116
gi|910240064~Tolypothrix_bouteillei
gi|924880229~Penicillium_nordicum
gi|1032442213~Fonsecaea_erecta
gi|1046787712~Cenococcum_geophilum_1_58
gi|628088428~Bipolaris_sorokiniana_ND90Pr
gi|1027150722~Paraphaeosphaeria_sporulosa
gi|1046781681~Cenococcum_geophilum_1_58
gi|628087528~Bipolaris_sorokiniana_ND90Pr
gi|587733572~Fusarium_oxysporum_f__sp__pisi_HDV247
gi|1040023458~Fusarium_poae
gi|596551043~Fusarium_graminearum
gi|729180802|emb|CEJ93768_1|~Torrubiella_hemipterigena
gi|667728623~Stachybotrys_chlorohalonata_IBT_40285
gi|512187700~Ophiostoma_piceae_UAMH_11346
gi|336365175~Serpula_lacrymans_var__lacrymans_S7_3
gi|504964564~Oscillatoria_acuminata_PCC_6304
gi|518333664~Pleurocapsa_sp__PCC_7319
gi|918325213~bacterium_endosymbiont_of_Mortierella_elong
gi|504949692~Nostoc_sp__PCC_7524
gi|1039198476~Anabaena_sp__MDT14b
gi|504892838~Anabaena_sp__90
gi|959923591~Mastigocoleus_testarum_BC008
gi|497317763~Synechocystis_sp__PCC_7509
gi|740236252~Tolypothrix_bouteillei_VB521301
gi|515344775~Fischerella_muscicola
gi|653002447~Planktothrix_agardhii
gi|505011242~Calothrix_sp__PCC_6303
gi|518323149~Calothrix_sp__PCC_7103
gi|503087852~Cyanothece_sp__PCC_7822
gi|516249015~Microchaete_sp__PCC_7126
gi|506435524~Cyanothece_sp__PCC_7424
gi|516258282~Geitlerinema_sp__PCC_7105
gi|488822971~Microcystis_aeruginosa_TAIHU98
gi|718249760|dbj|GAL93316_1|~Microcystis_aeruginosa_NIES-44
gi|490265097~Microcystis_aeruginosa_PCC_9807
gi|749836565|dbj|GAM52743_1|~bacterium_endosymbiont_of_Mort
gi|918325284~bacterium_endosymbiont_of_Mortierella_elongata_FM
gi|1049317831~Nostoc_sp__MBR_210
gi|700448866~Penicillium_expansum
gi|1027023528~Penicillium_expansum
gi|1046773504~Lepidopterella_palustris_CBS_459_81
gi|992088994~Aspergillus_nomius_NRRL_13137
gi|972228274|emb|CEL11678_1|~Aspergillus_calidoustus
gi|783319581~Candidatus_Magnetobacterium_bavaricum
gi|941167586~Pseudanabaena_sp__Roaring_Creek_
gi|497313425~Pseudanabaena_biceps_PCC_7429
gi|972548006~Phialocephala_scopiformis
gi|242805545~Talaromyces_stipitatus_ATCC_10500
gi|923071630~Hapalosiphon_sp__MRB220
gi|752365998~Tulasnella_calospora_MUT_4182
gi|752365346~Tulasnella_calospora_MUT_4182
gi|145508654~Paramecium_tetraurelia
gi|145552033~Paramecium_tetraurelia
gi|145492580~Paramecium_tetraurelia
gi|752371734~Tulasnella_calospora_MUT_4182
gi|927673373~Candidatus_Magnetomorum_sp__HK-1
gi|472243019~Botrytis_cinerea_BcDW1
gi|1023272430~Daedalea_quercina_L-15889
gi|749873782~Sphaerobolus_stellatus_SS14
gi|504966090~Chroococcidiopsis_thermalis_PCC_7203
gi|504988475~Oscillatoria_nigro-viridis_PCC_7112
gi|242770782~Talaromyces_stipitatus_ATCC_10500
gi|951277070|emb|CUS92147_1|~Candidatus_Kryptobacter_tengchongensis
gi|497238095~Burkholderiales_bacterium_JOSHI_001
gi|500065513~Chlorobium_phaeobacteroides_DSM_266
gi|682332342~Pseudogymnoascus_sp__VKM_F-4281_FW-2241
gi|751745017~Oidiodendron_maius_Zn
gi|1040498651~Pseudogymnoascus_sp__WSF_3629
gi|682282186~Pseudogymnoascus_sp__VKM_F-3808
gi|751749637~Oidiodendron_maius_Zn
gi|158305561~Acaryochloris_marina_MBIC11017
gi|500596266~Roseiflexus_sp__RS-1
gi|569387362~Reticulomyxa_filosa
gi|589610316~Candidatus_Accumulibacter_sp__BA-92
gi|569313051~Reticulomyxa_filosa
gi|353239502|emb|CCA71411_1|~Serendipita_indica_DSM_11827
gi|495456746~Moorea_producens_3L
gi|935598342~Planktothricoides_sp__SR001
gi|156050805~Sclerotinia_sclerotiorum_1980_UF-70
gi|156044028~Sclerotinia_sclerotiorum_1980_UF-70
gi|449546147~Gelatoporia_subvermispora_B
gi|1012957637~Hypsizygus_marmoreus
gi|749758534~Plicaturopsis_crispa_FD-325_SS-3
gi|1021832875~Fibulorhizoctonia_sp__CBS_109695
gi|749760446~Plicaturopsis_crispa_FD-325_SS-3
gi|449539810~Gelatoporia_subvermispora_B
gi|695569639~Heterobasidion_irregulare_TC_32-1
gi|695576252~Heterobasidion_irregulare_TC_32-1
gi|749766339~Plicaturopsis_crispa_FD-325_SS-3
gi|630354667~Gloeophyllum_trabeum_ATCC_11539
gi|1021846644~Fibulorhizoctonia_sp__CBS_109695
gi|618817894~Stereum_hirsutum_FP-91666_SS1
gi|751669472~Serendipita_vermifera_MAFF_305830
gi|751665235~Serendipita_vermifera_MAFF_305830
gi|646290470~Botryobasidium_botryosum_FD-172_SS1
gi|751008866~Gymnopus_luxurians_FD-317_M1
gi|751011959~Gymnopus_luxurians_FD-317_M1
gi|618817762~Stereum_hirsutum_FP-91666_SS1
gi|751690870~Hebeloma_cylindrosporum_h7
gi|751732332~Piloderma_croceum_F_1598
gi|750938922~Laccaria_amethystina_LaAM-08-1
gi|751735368~Piloderma_croceum_F_1598
gi|751688765~Hebeloma_cylindrosporum_h7
gi|648167286~Galerina_marginata_CBS_339_88
gi|618808869~Stereum_hirsutum_FP-91666_SS1
gi|914262536~Termitomyces_sp__J132
gi|914261916~Termitomyces_sp__J132
gi|914260113~Termitomyces_sp__J132
gi|750942279~Laccaria_amethystina_LaAM-08-1
gi|648170798~Galerina_marginata_CBS_339_88
gi|752345289~Pisolithus_tinctorius_Marx_270
gi|752342415~Pisolithus_tinctorius_Marx_270
gi|908639740~Acaryochloris_marina
gi|158307657~Acaryochloris_marina_MBIC11017
gi|751728237~Piloderma_croceum_F_1598
gi|751021121~Gymnopus_luxurians_FD-317_M1
gi|504971551~Chamaesiphon_minutus_PCC_6605
gi|749868061~Sphaerobolus_stellatus_SS14
gi|1035376422~Rhizopogon_vinicolor_AM-OR11-026
gi|1035374617~Rhizopogon_vinicolor_AM-OR11-026
gi|1035373445~Rhizopogon_vinicolor_AM-OR11-026
gi|807213697~Limnoraphis_robusta_CS-951
gi|497471963~Lyngbya_sp__PCC_8106
gi|807762063~Fibroporia_radiculosa
gi|750989389~Suillus_luteus_UH-Slu-Lm8-n1
gi|750991724~Suillus_luteus_UH-Slu-Lm8-n1
gi|576982012~Rhizoctonia_solani_AG-3_Rhs1AP
gi|660959641~Rhizoctonia_solani_123E
gi|1044610544~Sanghuangporus_baumii
gi|1044611289~Sanghuangporus_baumii
gi|595772106~Fomitiporia_mediterranea_MF3/22
gi|595785478~Fomitiporia_mediterranea_MF3/22
gi|1021812751~Fibulorhizoctonia_sp__CBS_109695
gi|576995068~Rhizoctonia_solani_AG-3_Rhs1AP
gi|595782704~Fomitiporia_mediterranea_MF3/22
gi|1023216146~Laetiporus_sulphureus_93-53
gi|1044614682~Sanghuangporus_baumii
gi|938299019~Phormidium_sp__OSCR
gi|695565548~Heterobasidion_irregulare_TC_32-1
gi|405976663~Crassostrea_gigas
gi|1000846441~Leucoagaricus_sp__SymC_cos
gi|299752644~Coprinopsis_cinerea_okayama7#130
gi|1024077214~Peniophora_sp__CONT
gi|783333853~Candidatus_Magnetobacterium_bavaricum
gi|690436630~Pygoscelis_adeliae
gi|690436627~Pygoscelis_adeliae
gi|927687165~Candidatus_Magnetomorum_sp__HK-1
GSMAST4A1_ASY1_ANO1_5085_1
gi|297170404~uncultured_gamma_proteobacterium_HF0010
0.3
Bacteria
Eukaryota
SAG
NA

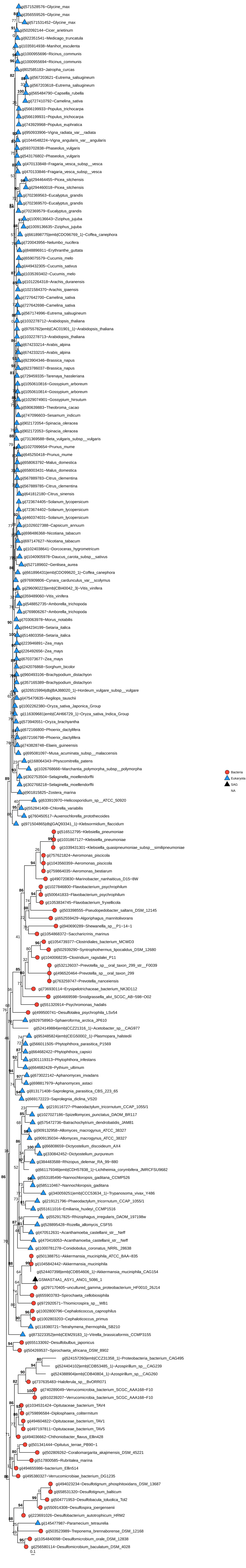
Bacteria
Eukaryota
SAG
NA
0.1

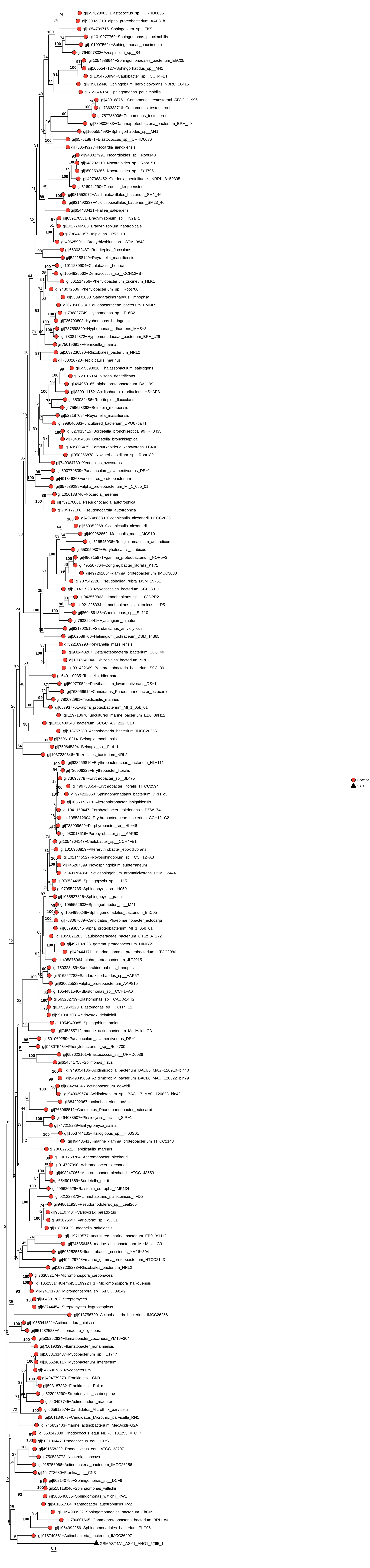

gi|657623003~Blastococcus_sp__URHD0036
gi|930023319~alpha_proteobacterium_AAP81b
gi|1054799716~Sphingobium_sp__TKS
gi|1010977769~Sphingomonas_paucimobilis
gi|1010975624~Sphingomonas_paucimobilis
gi|764997832~Azospirillum_sp__B4
gi|1054988644~Sphingomonadales_bacterium_EhC05
gi|1055547127~Sphingorhabdus_sp__M41
gi|1054763994~Caulobacter_sp__CCH4~E1
gi|739612448~Sphingobium_herbicidovorans_NBRC_16415
gi|765344874~Sphingomonas_paucimobilis
gi|489168761~Comamonas_testosteroni_ATCC_11996
gi|736333716~Comamonas_testosteroni
gi|757788006~Comamonas_testosteroni
gi|780802683~Gammaproteobacteria_bacterium_BRH_c0
gi|1055554993~Sphingorhabdus_sp__M41
gi|657618871~Blastococcus_sp__URHD0036
gi|750549277~Nocardia_jiangxiensis
gi|948027991~Nocardioides_sp__Root140
gi|948232110~Nocardioides_sp__Root151
gi|950259266~Nocardioides_sp__Soil796
gi|497363452~Gordonia_neofelifaecis_NRRL_B~59395
gi|516944290~Gordonia_kroppenstedtii
gi|931553972~Acidithiobacillales_bacterium_SM1_46
gi|931490337~Acidithiobacillales_bacterium_SM23_46
gi|654480411~Hallea_salexigens
gi|639176331~Bradyrhizobium_sp__Tv2a~2
gi|1027746580~Bradyrhizobium_neotropicale
gi|736441057~Afipia_sp__P52~10
gi|496259011~Bradyrhizobium_sp__STM_3843
gi|653032487~Rubritepida_flocculans
gi|522188149~Reyranella_massiliensis
gi|1011230904~Caulobacter_henricii
gi|1054926562~Dermacoccus_sp__CCH12~B7
gi|501514756~Phenylobacterium_zucineum_HLK1
gi|948072586~Phenylobacterium_sp__Root700
gi|550931080~Sandarakinorhabdus_limnophila
gi|670500514~Caulobacteraceae_bacterium_PMMR1
gi|736827749~Hyphomonas_sp__T16B2
gi|736790803~Hyphomonas_beringensis
gi|737598890~Hyphomonas_adhaerens_MHS~3
gi|780819872~Hyphomonadaceae_bacterium_BRH_c29
gi|750196917~Henriciella_marina
gi|1037236590~Rhizobiales_bacterium_NRL2
gi|780026723~Tepidicaulis_marinus
gi|655390810~Thalassobaculum_salexigens
gi|655015334~Nisaea_denitrificans
gi|494950165~alpha_proteobacterium_BAL199
gi|889911152~Acidisphaera_rubrifaciens_HS~AP3
gi|653032486~Rubritepida_flocculans
gi|759623398~Belnapia_moabensis
gi|522187694~Reyranella_massiliensis
gi|998640083~uncultured_bacterium_UPO67part1
gi|627913415~Bordetella_bronchiseptica_99~R~0433
gi|704394584~Bordetella_bronchiseptica
gi|499806435~Paraburkholderia_xenovorans_LB400
gi|950256878~Noviherbaspirillum_sp__Root189
gi|740364739~Xenophilus_azovorans
gi|500779539~Parvibaculum_lavamentivorans_DS~1
gi|491846363~uncultured_proteobacterium
gi|657939289~alpha_proteobacterium_Mf_1_05b_01
gi|1056138740~Nocardia_harenae
gi|739176861~Pseudonocardia_autotrophica
gi|739177100~Pseudonocardia_autotrophica
gi|497488689~Oceanicaulis_alexandrii_HTCC2633
gi|550952968~Oceanicaulis_alexandrii
gi|499962862~Maricaulis_maris_MCS10
gi|516545036~Robiginitomaculum_antarcticum
gi|550950807~Euryhalocaulis_caribicus
gi|496315871~gamma_proteobacterium_NOR5~3
gi|495567864~Congregibacter_litoralis_KT71
gi|497261854~gamma_proteobacterium_IMCC3088
gi|737542726~Pseudohaliea_rubra_DSM_19751
gi|931471923~Myxococcales_bacterium_SG8_38_1
gi|942569863~Limnohabitans_sp__103DPR2
gi|921225334~Limnohabitans_planktonicus_II~D5
gi|860489138~Caenimonas_sp__SL110
gi|763322441~Hyalangium_minutum
gi|921302516~Sandaracinus_amylolyticus
gi|502589700~Haliangium_ochraceum_DSM_14365
gi|522189293~Reyranella_massiliensis
gi|931448207~Betaproteobacteria_bacterium_SG8_40
gi|1037240046~Rhizobiales_bacterium_NRL2
gi|931422669~Betaproteobacteria_bacterium_SG8_39
gi|640110035~Tomitella_biformata
gi|500779524~Parvibaculum_lavamentivorans_DS~1
gi|763066619~Candidatus_Phaeomarinobacter_ectocarpi
gi|780032961~Tepidicaulis_marinus
gi|657937701~alpha_proteobacterium_Mf_1_05b_01
gi|119713676~uncultured_marine_bacterium_EB0_39H12
gi|1028409340~bacterium_SCGC_AG~212~C10
gi|918757280~Actinobacteria_bacterium_IMCC26256
gi|759618214~Belnapia_moabensis
gi|759645304~Belnapia_sp__F~4~1
gi|1037239646~Rhizobiales_bacterium_NRL2
gi|938259810~Erythrobacteraceae_bacterium_HL~111
gi|736906229~Erythrobacter_litoralis
gi|736957787~Erythrobacter_sp__JL475
gi|499733654~Erythrobacter_litoralis_HTCC2594
gi|974212068~Sphingomonadales_bacterium_BRH_c3
gi|1056073718~Altererythrobacter_ishigakiensis
gi|1041150447~Porphyrobacter_dokdonensis_DSW~74
gi|1055812904~Erythrobacteraceae_bacterium_CCH12~C2
gi|738909620~Porphyrobacter_sp__HL~46
gi|930013616~Porphyrobacter_sp__AAP60
gi|1054764147~Caulobacter_sp__CCH4~E1
gi|1010968819~Altererythrobacter_epoxidivorans
gi|1011445527~Novosphingobium_sp__CCH12~A3
gi|746287399~Novosphingobium_subterraneum
gi|499764356~Novosphingobium_aromaticivorans_DSM_12444
gi|970534495~Sphingopyxis_sp__H115
gi|970552785~Sphingopyxis_sp__H050
gi|1055527326~Sphingopyxis_granuli
gi|1055552633~Sphingorhabdus_sp__M41
gi|1054990249~Sphingomonadales_bacterium_EhC05
gi|763067689~Candidatus_Phaeomarinobacter_ectocarpi
gi|657938545~alpha_proteobacterium_Mf_1_05b_01
gi|1055021263~Caulobacteraceae_bacterium_OTSz_A_272
gi|497102028~gamma_proteobacterium_HIMB55
gi|494441711~marine_gamma_proteobacterium_HTCC2080
gi|495875964~alpha_proteobacterium_JLT2015
gi|750323489~Sandarakinorhabdus_limnophila
gi|516262782~Sandarakinorhabdus_sp__AAP62
gi|930025528~alpha_proteobacterium_AAP81b
gi|1054481546~Blastomonas_sp__CCH1~A6
gi|563282739~Blastomonas_sp__CACIA14H2
gi|1053960120~Blastomonas_sp__CCH7~E1
gi|991990708~Acidovorax_delafieldii
gi|1054940085~Sphingobium_amiense
gi|745855712~marine_actinobacterium_MedAcidi~G3
gi|501060259~Parvibaculum_lavamentivorans_DS~1
gi|948075434~Phenylobacterium_sp__Root700
gi|657622101~Blastococcus_sp__URHD0036
gi|654541755~Solimonas_flava
gi|949054136~Acidimicrobia_bacterium_BACL6_MAG~120910~bin40
gi|949045669~Acidimicrobia_bacterium_BACL6_MAG~120322~bin79
gi|684284246~actinobacterium_acAcidi
gi|949039674~Acidimicrobium_sp__BACL17_MAG~120823~bin42
gi|684292967~actinobacterium_acAcidi
gi|763068511~Candidatus_Phaeomarinobacter_ectocarpi
gi|494035507~Plesiocystis_pacifica_SIR~1
gi|747218289~Enhygromyxa_salina
gi|1053744135~Halioglobus_sp__HI00S01
gi|494435415~marine_gamma_proteobacterium_HTCC2148
gi|780027522~Tepidicaulis_marinus
gi|1001758764~Achromobacter_piechaudii
gi|914797990~Achromobacter_piechaudii
gi|493247066~Achromobacter_piechaudii_ATCC_43553
gi|654901669~Bordetella_petrii
gi|499620929~Ralstonia_eutropha_JMP134
gi|921228872~Limnohabitans_planktonicus_II~D5
gi|948011925~Pseudorhodoferax_sp__Leaf265
gi|951107404~Variovorax_paradoxus
gi|983025697~Variovorax_sp__WDL1
gi|928995629~Ideonella_sakaiensis
gi|119713577~uncultured_marine_bacterium_EB0_39H12
gi|745856446~marine_actinobacterium_MedAcidi~G3
gi|505252555~Illumatobacter_coccineus_YM16~304
gi|494429748~marine_gamma_proteobacterium_HTCC2143
gi|1037238233~Rhizobiales_bacterium_NRL2
gi|763082174~Micromonospora_carbonacea
gi|1052351440|emb|SCE99224_1|~Micromonospora_haikouensis
gi|494131707~Micromonospora_sp__ATCC_39149
gi|664301782~Streptomyces
gi|83744454~Streptomyces_hygroscopicus
gi|918756799~Actinobacteria_bacterium_IMCC26256
gi|1055941521~Actinomadura_hibisca
gi|651282528~Actinomadura_oligospora
gi|505252624~Illumatobacter_coccineus_YM16~304
gi|750190398~Illumatobacter_nonamiensis
gi|1038131487~Mycobacterium_sp__E1747
gi|1055248116~Mycobacterium_interjectum
gi|942696786~Mycobacterium
gi|494779729~Frankia_sp__CN3
gi|503187382~Frankia_sp__EuI1c
gi|522045290~Streptomyces_scabrisporus
gi|640497745~Actinomadura_madurae
gi|665912574~Candidatus_Microthrix_parvicella
gi|501184073~Candidatus_Microthrix_parvicella_RN1
gi|745852403~marine_actinobacterium_MedAcidi~G2A
gi|550242038~Rhodococcus_equi_NBRC_101255_=_C_7
gi|503180447~Rhodococcus_equi_103S
gi|491658229~Rhodococcus_equi_ATCC_33707
gi|750533772~Nocardia_concava
gi|918756066~Actinobacteria_bacterium_IMCC26256
gi|494778680~Frankia_sp__CN3
gi|662140799~Sphingomonas_sp__DC~6
gi|515118040~Sphingomonas_wittichii
gi|500540835~Sphingomonas_wittichii_RW1
gi|501061584~Xanthobacter_autotrophicus_Py2
gi|1054989932~Sphingomonadales_bacterium_EhC05
gi|780801665~Gammaproteobacteria_bacterium_BRH_c0
gi|1054992256~Sphingomonadales_bacterium_EhC05
gi|918749561~Actinobacteria_bacterium_IMCC26207
GSMAST4A1_ASY1_ANO1_5265_1
0.1
Bacteria
SAG

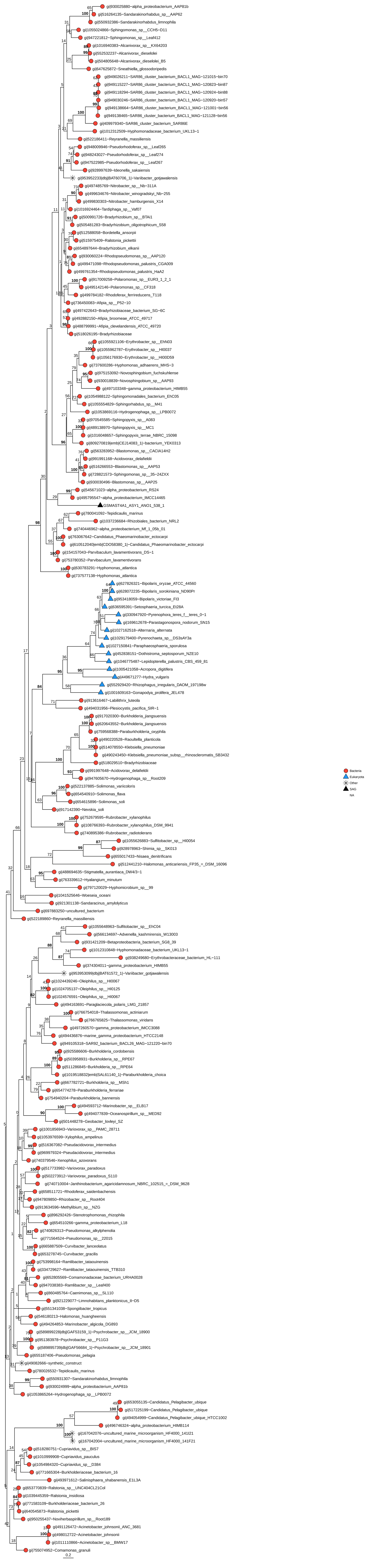

Bacteria
Eukaryota
Other
SAG
NA
0.2

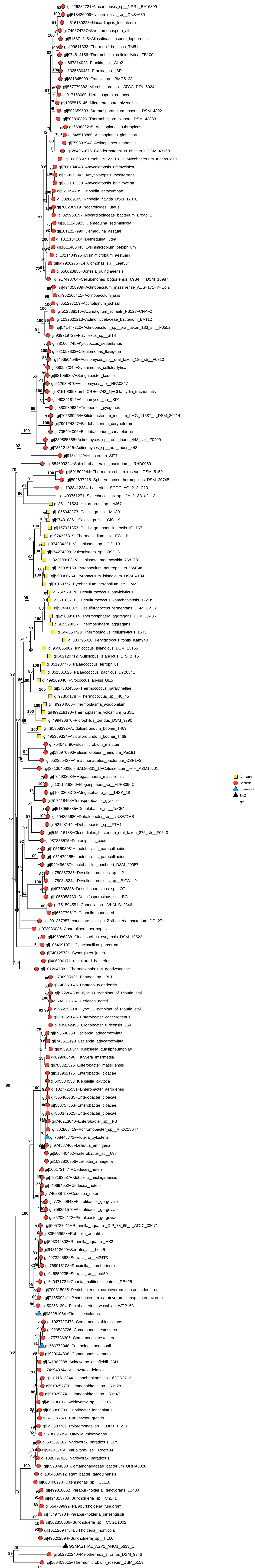
Archaea
Bacteria
Eukaryota
SAG
NA
0.1

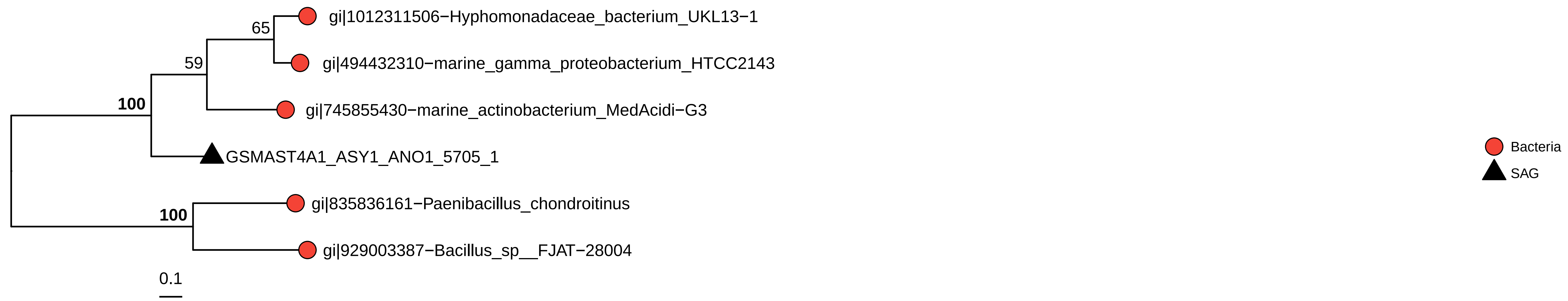
gi|1012311506−Hyphomonadaceae_bacterium_UKL13−1
gi|494432310−marine_gamma_proteobacterium_HTCC2143
gi|745855430−marine_actinobacterium_MedAcidi−G3
GSMAST4A1_ASY1_ANO1_5705_1
gi|835836161−Paenibacillus_chondroitinus
gi|929003387−Bacillus_sp__FJAT−28004
65
59
100
100
0.1
Bacteria
SAG

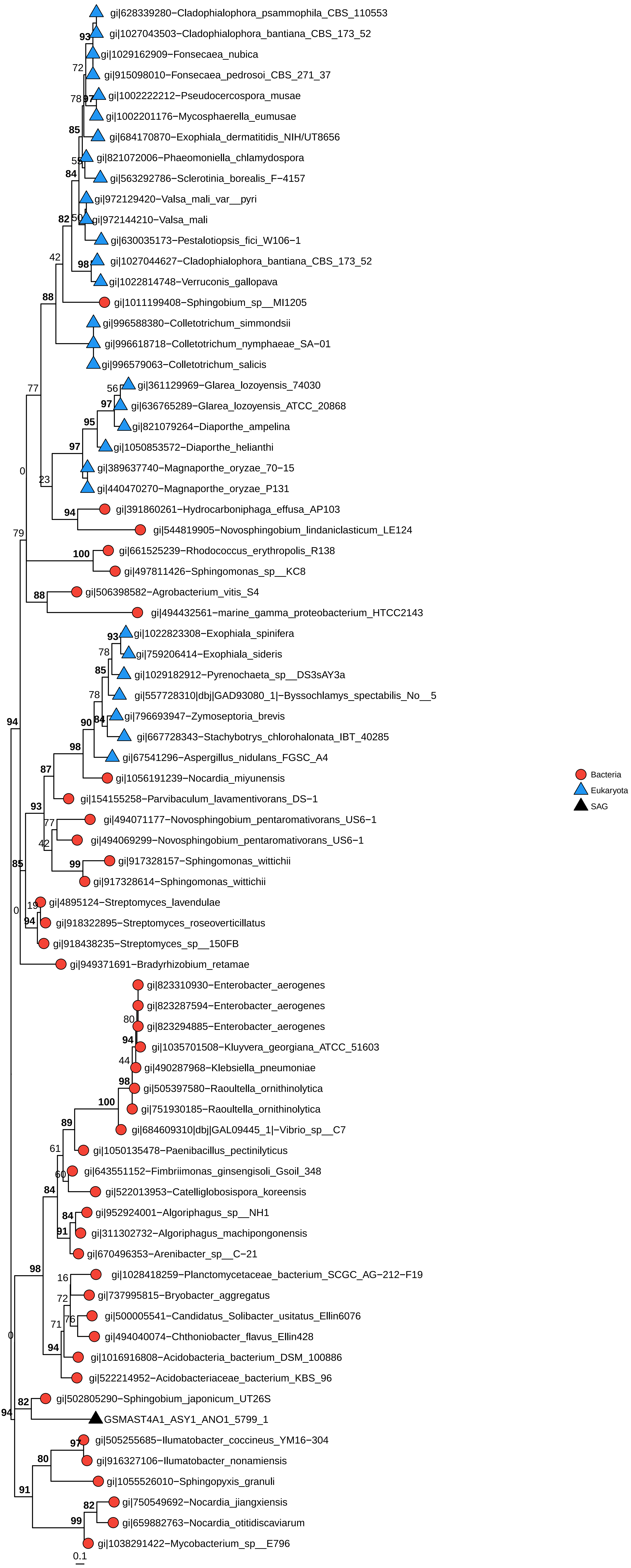
gi|628339280−Cladophialophora_psammophila_CBS_110553
gi|1027043503−Cladophialophora_bantiana_CBS_173_52
gi|1029162909−Fonsecaea_nubica
gi|915098010−Fonsecaea_pedrosoi_CBS_271_37
gi|1002222212−Pseudocercospora_musae
gi|1002201176−Mycosphaerella_eumusae
gi|684170870−Exophiala_dermatitidis_NIH/UT8656
gi|821072006−Phaeomoniella_chlamydospora
gi|563292786−Sclerotinia_borealis_F−4157
gi|972129420−Valsa_mali_var__pyri
gi|972144210−Valsa_mali
gi|630035173−Pestalotiopsis_fici_W106−1
gi|1027044627−Cladophialophora_bantiana_CBS_173_52
gi|1022814748−Verruconis_gallopava
gi|1011199408−Sphingobium_sp__MI1205
gi|996588380−Colletotrichum_simmondsii
gi|996618718−Colletotrichum_nymphaeae_SA−01
gi|996579063−Colletotrichum_salicis
gi|361129969−Glarea_lozoyensis_74030
gi|636765289−Glarea_lozoyensis_ATCC_20868
gi|821079264−Diaporthe_ampelina
gi|1050853572−Diaporthe_helianthi
gi|389637740−Magnaporthe_oryzae_70−15
gi|440470270−Magnaporthe_oryzae_P131
gi|391860261−Hydrocarboniphaga_effusa_AP103
gi|544819905−Novosphingobium_lindaniclasticum_LE124
gi|661525239−Rhodococcus_erythropolis_R138
gi|497811426−Sphingomonas_sp__KC8
gi|506398582−Agrobacterium_vitis_S4
gi|494432561−marine_gamma_proteobacterium_HTCC2143
gi|1022823308−Exophiala_spinifera
gi|759206414−Exophiala_sideris
gi|1029182912−Pyrenochaeta_sp__DS3sAY3a
gi|557728310|dbj|GAD93080_1|−Byssochlamys_spectabilis_No__5
gi|796693947−Zymoseptoria_brevis
gi|667728343−Stachybotrys_chlorohalonata_IBT_40285
gi|67541296−Aspergillus_nidulans_FGSC_A4
gi|1056191239−Nocardia_miyunensis
gi|154155258−Parvibaculum_lavamentivorans_DS−1
gi|494071177−Novosphingobium_pentaromativorans_US6−1
gi|494069299−Novosphingobium_pentaromativorans_US6−1
gi|917328157−Sphingomonas_wittichii
gi|917328614−Sphingomonas_wittichii
gi|4895124−Streptomyces_lavendulae
gi|918322895−Streptomyces_roseoverticillatus
gi|918438235−Streptomyces_sp__150FB
gi|949371691−Bradyrhizobium_retamae
gi|823310930−Enterobacter_aerogenes
gi|823287594−Enterobacter_aerogenes
gi|823294885−Enterobacter_aerogenes
gi|1035701508−Kluyvera_georgiana_ATCC_51603
gi|490287968−Klebsiella_pneumoniae
gi|505397580−Raoultella_ornithinolytica
gi|751930185−Raoultella_ornithinolytica
gi|684609310|dbj|GAL09445_1|−Vibrio_sp__C7
gi|1050135478−Paenibacillus_pectinilyticus
gi|643551152−Fimbriimonas_ginsengisoli_Gsoil_348
gi|522013953−Catelliglobosispora_koreensis
gi|952924001−Algoriphagus_sp__NH1
gi|311302732−Algoriphagus_machipongonensis
gi|670496353−Arenibacter_sp__C−21
gi|1028418259−Planctomycetaceae_bacterium_SCGC_AG−212−F19
gi|737995815−Bryobacter_aggregatus
gi|500005541−Candidatus_Solibacter_usitatus_Ellin6076
gi|494040074−Chthoniobacter_flavus_Ellin428
gi|1016916808−Acidobacteria_bacterium_DSM_100886
gi|522214952−Acidobacteriaceae_bacterium_KBS_96
gi|502805290−Sphingobium_japonicum_UT26S
GSMAST4A1_ASY1_ANO1_5799_1
gi|505255685−Ilumatobacter_coccineus_YM16−304
gi|916327106−Ilumatobacter_nonamiensis
gi|1055526010−Sphingopyxis_granuli
gi|750549692−Nocardia_jiangxiensis
gi|659882763−Nocardia_otitidiscaviarum
gi|1038291422−Mycobacterium_sp__E796
0.1
Bacteria
Eukaryota
SAG

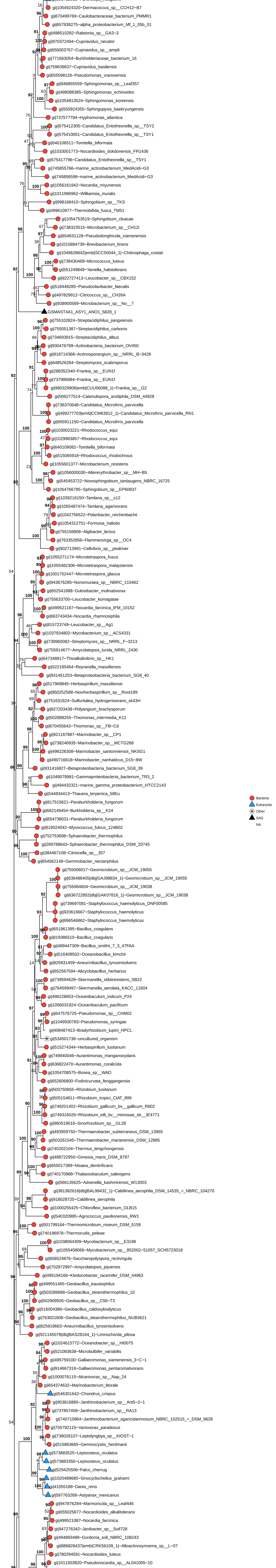
Bacteria
Eukaryota
Other
SAG
NA
0.1

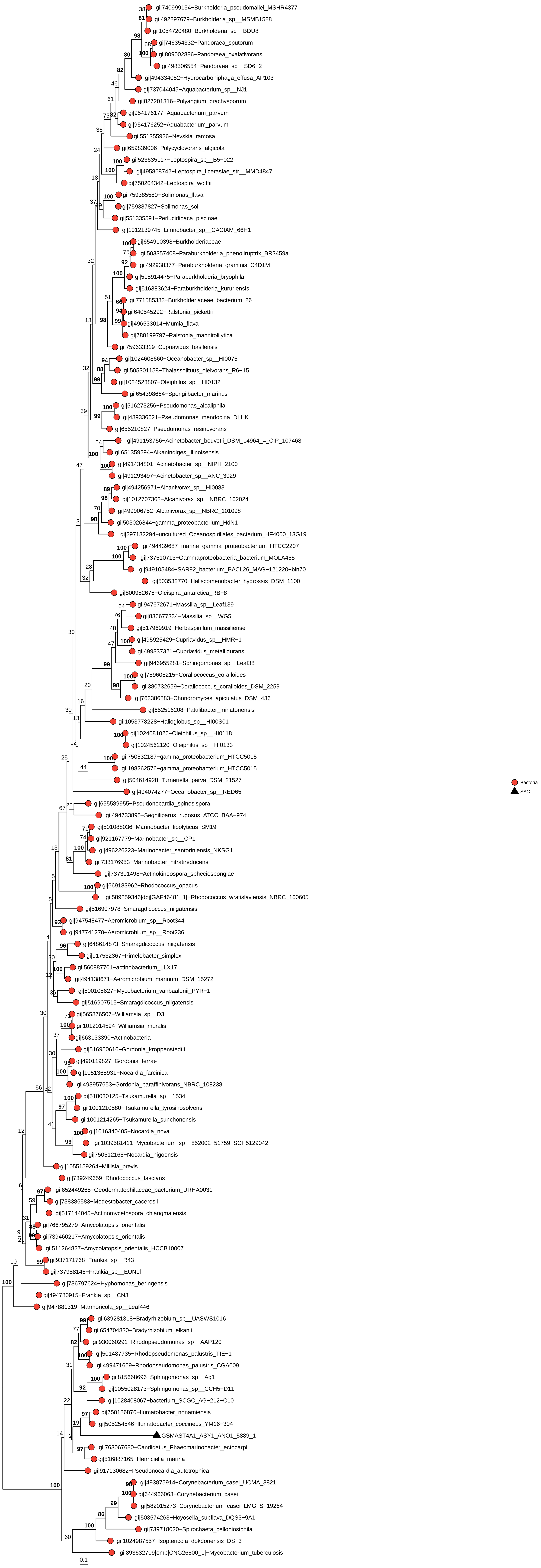

gi|740999154~Burkholderia_pseudomallei_MSHR4377
gi|492897679~Burkholderia_sp__MSMB1588
gi|1054720480~Burkholderia_sp__BDU8
gi|746354332~Pandoraea_sputorum
gi|809002886~Pandoraea_oxalativorans
gi|498506554~Pandoraea_sp__SD6~2
gi|494334052~Hydrocarboniphaga_effusa_AP103
gi|737044045~Aquabacterium_sp__NJ1
gi|827201316~Polyangium_brachysporum
gi|954176177~Aquabacterium_parvum
gi|954176252~Aquabacterium_parvum
gi|551355926~Nevskia_ramosa
gi|659839006~Polycyclovorans_algicola
gi|523635117~Leptospira_sp__B5~022
gi|495868742~Leptospira_licerasiae_str__MMD4847
gi|750204342~Leptospira_wolffii
gi|759385580~Solimonas_flava
gi|759387827~Solimonas_soli
gi|551335591~Perlucidibaca_piscinae
gi|1012139745~Limnobacter_sp__CACIAM_66H1
gi|654910398~Burkholderiaceae
gi|503357408~Paraburkholderia_phenoliruptrix_BR3459a
gi|492938377~Paraburkholderia_graminis_C4D1M
gi|518914475~Paraburkholderia_bryophila
gi|516383624~Paraburkholderia_kururiensis
gi|771585383~Burkholderiaceae_bacterium_26
gi|640545292~Ralstonia_pickettii
gi|496533014~Mumia_flava
gi|788199797~Ralstonia_mannitolilytica
gi|759633319~Cupriavidus_basilensis
gi|1024608660~Oceanobacter_sp__HI0075
gi|505301158~Thalassolituus_oleivorans_R6~15
gi|1024523807~Oleiphilus_sp__HI0132
gi|654398664~Spongiibacter_marinus
gi|516273256~Pseudomonas_alcaliphila
gi|489336621~Pseudomonas_mendocina_DLHK
gi|655210827~Pseudomonas_resinovorans
gi|491153756~Acinetobacter_bouvetii_DSM_14964_=_CIP_107468
gi|651359294~Alkanindiges_illinoisensis
gi|491434801~Acinetobacter_sp__NIPH_2100
gi|491293497~Acinetobacter_sp__ANC_3929
gi|494256971~Alcanivorax_sp__HI0083
gi|1012707362~Alcanivorax_sp__NBRC_102024
gi|499906752~Alcanivorax_sp__NBRC_101098
gi|503026844~gamma_proteobacterium_HdN1
gi|297182294~uncultured_Oceanospirillales_bacterium_HF4000_13G19
gi|494439687~marine_gamma_proteobacterium_HTCC2207
gi|737510713~Gammaproteobacteria_bacterium_MOLA455
gi|949105484~SAR92_bacterium_BACL26_MAG~121220~bin70
gi|503532770~Haliscomenobacter_hydrossis_DSM_1100
gi|800982676~Oleispira_antarctica_RB~8
gi|947672671~Massilia_sp__Leaf139
gi|836677334~Massilia_sp__WG5
gi|517969919~Herbaspirillum_massiliense
gi|495925429~Cupriavidus_sp__HMR~1
gi|499837321~Cupriavidus_metallidurans
gi|946955281~Sphingomonas_sp__Leaf38
gi|759605215~Corallococcus_coralloides
gi|380732659~Corallococcus_coralloides_DSM_2259
gi|763386883~Chondromyces_apiculatus_DSM_436
gi|652516208~Patulibacter_minatonensis
gi|1053778228~Halioglobus_sp__HI00S01
gi|1024681026~Oleiphilus_sp__HI0118
gi|1024562120~Oleiphilus_sp__HI0133
gi|750532187~gamma_proteobacterium_HTCC5015
gi|198262576~gamma_proteobacterium_HTCC5015
gi|504614928~Turneriella_parva_DSM_21527
gi|494074277~Oceanobacter_sp__RED65
gi|655589955~Pseudonocardia_spinosispora
gi|494733895~Segniliparus_rugosus_ATCC_BAA~974
gi|501088036~Marinobacter_lipolyticus_SM19
gi|921167779~Marinobacter_sp__CP1
gi|496226223~Marinobacter_santoriniensis_NKSG1
gi|738176953~Marinobacter_nitratireducens
gi|737301498~Actinokineospora_spheciospongiae
gi|669183962~Rhodococcus_opacus
gi|589259346|dbj|GAF46481_1|~Rhodococcus_wratislaviensis_NBRC_100605
gi|516907978~Smaragdicoccus_niigatensis
gi|947548477~Aeromicrobium_sp__Root344
gi|947741270~Aeromicrobium_sp__Root236
gi|648614873~Smaragdicoccus_niigatensis
gi|917532367~Pimelobacter_simplex
gi|560887701~actinobacterium_LLX17
gi|494138671~Aeromicrobium_marinum_DSM_15272
gi|500105627~Mycobacterium_vanbaalenii_PYR~1
gi|516907515~Smaragdicoccus_niigatensis
gi|565876507~Williamsia_sp__D3
gi|1012014594~Williamsia_muralis
gi|663133390~Actinobacteria
gi|516950616~Gordonia_kroppenstedtii
gi|490119827~Gordonia_terrae
gi|1051365931~Nocardia_farcinica
gi|493957653~Gordonia_paraffinivorans_NBRC_108238
gi|518030125~Tsukamurella_sp__1534
gi|1001210580~Tsukamurella_tyrosinosolvens
gi|1001214265~Tsukamurella_sunchonensis
gi|1016340405~Nocardia_nova
gi|1039581411~Mycobacterium_sp__852002~51759_SCH5129042
gi|750512165~Nocardia_higoensis
gi|1055159264~Millisia_brevis
gi|739249659~Rhodococcus_fascians
gi|652449265~Geodermatophilaceae_bacterium_URHA0031
gi|738386583~Modestobacter_caceresii
gi|517144045~Actinomycetospora_chiangmaiensis
gi|766795279~Amycolatopsis_orientalis
gi|739460217~Amycolatopsis_orientalis
gi|511264827~Amycolatopsis_orientalis_HCCB10007
gi|937171768~Frankia_sp__R43
gi|737988146~Frankia_sp__EUN1f
gi|736797624~Hyphomonas_beringensis
gi|494780915~Frankia_sp__CN3
gi|947881319~Marmoricola_sp__Leaf446
gi|639281318~Bradyrhizobium_sp__UASWS1016
gi|654704830~Bradyrhizobium_elkanii
gi|930060291~Rhodopseudomonas_sp__AAP120
gi|501487735~Rhodopseudomonas_palustris_TIE~1
gi|499471659~Rhodopseudomonas_palustris_CGA009
gi|815668696~Sphingomonas_sp__Ag1
gi|1055028173~Sphingomonas_sp__CCH5~D11
gi|1028408067~bacterium_SCGC_AG~212~C10
gi|750186876~Ilumatobacter_nonamiensis
gi|505254546~Ilumatobacter_coccineus_YM16~304
GSMAST4A1_ASY1_ANO1_5889_1
gi|763067680~Candidatus_Phaeomarinobacter_ectocarpi
gi|516887165~Henriciella_marina
gi|917130682~Pseudonocardia_autotrophica
gi|493875914~Corynebacterium_casei_UCMA_3821
gi|644966063~Corynebacterium_casei
gi|582015273~Corynebacterium_casei_LMG_S~19264
gi|503574263~Hoyosella_subflava_DQS3~9A1
gi|739718020~Spirochaeta_cellobiosiphila
gi|1024987557~Isoptericola_dokdonensis_DS~3
gi|893632709|emb|CNG26500_1|~Mycobacterium_tuberculosis
0.1
Bacteria
SAG

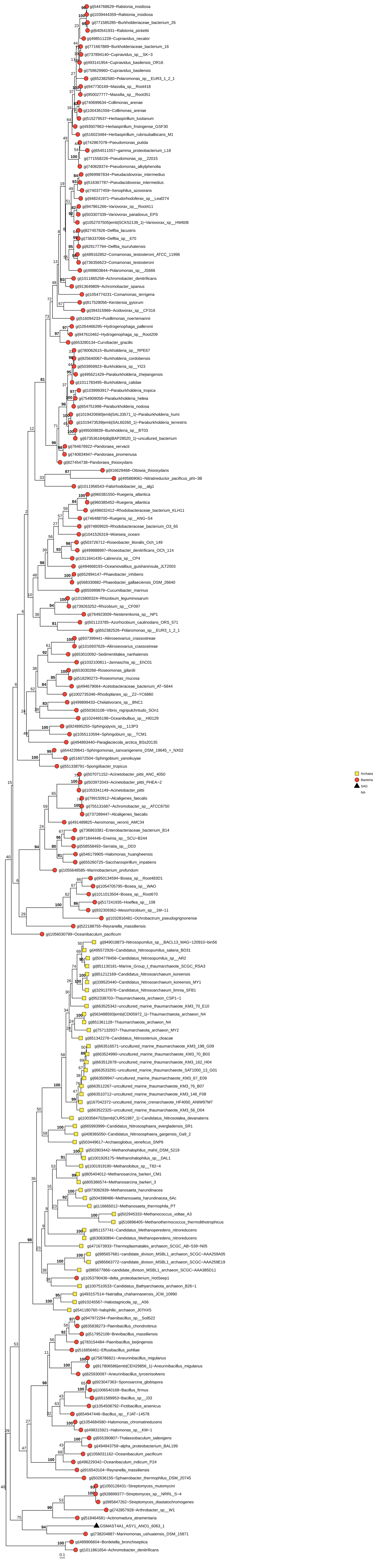
gi|544768629~Ralstonia_insidiosa
gi|1039444359~Ralstonia_insidiosa
gi|771585285~Burkholderiaceae_bacterium_26
gi|640541931~Ralstonia_pickettii
gi|498511228~Cupriavidus_necator
gi|771667889~Burkholderiaceae_bacterium_16
gi|737894140~Cupriavidus_sp__SK-3
gi|493141954~Cupriavidus_basilensis_OR16
gi|759629960~Cupriavidus_basilensis
gi|652382580~Polaromonas_sp__EUR3_1_2_1
gi|947730169~Massilia_sp__Root418
gi|950027777~Massilia_sp__Root351
gi|740699634~Collimonas_arenae
gi|1004361556~Collimonas_arenae
gi|515279537~Herbaspirillum_lusitanum
gi|493507963~Herbaspirillum_frisingense_GSF30
gi|516023484~Herbaspirillum_rubrisubalbicans_M1
gi|742867078~Pseudomonas_putida
gi|654511557~gamma_proteobacterium_L18
gi|771558226~Pseudomonas_sp__22015
gi|740828374~Pseudomonas_alkylphenolia
gi|969987834~Pseudacidovorax_intermedius
gi|516367787~Pseudacidovorax_intermedius
gi|740377459~Xenophilus_azovorans
gi|948241971~Pseudorhodoferax_sp__Leaf274
gi|947861266~Variovorax_sp__Root411
gi|503307339~Variovorax_paradoxus_EPS
gi|1052707505|emb|SCK52139_1|~Variovorax_sp__HW608
gi|827457826~Delftia_lacustris
gi|736337066~Delftia_sp__670
gi|829177794~Delftia_tsuruhatensis
gi|489162852~Comamonas_testosteroni_ATCC_11996
gi|736356623~Comamonas_testosteroni
gi|499803844~Polaromonas_sp__JS666
gi|1011865258~Achromobacter_denitrificans
gi|913649809~Achromobacter_spanius
gi|1054774231~Comamonas_terrigena
gi|817528056~Kerstersia_gyiorum
gi|394315966~Acidovorax_sp__CF316
gi|516094233~Pusillimonas_noertemannii
gi|1054466295~Hydrogenophaga_palleronii
gi|947610462~Hydrogenophaga_sp__Root209
gi|653280134~Curvibacter_gracilis
gi|780062615~Burkholderia_sp__RPE67
gi|925640067~Burkholderia_cordobensis
gi|503959923~Burkholderia_sp__YI23
gi|495621429~Paraburkholderia_zhejiangensis
gi|1011783495~Burkholderia_calidae
gi|1039993917~Paraburkholderia_tropica
gi|754909058~Paraburkholderia_heleia
gi|654751998~Paraburkholderia_nodosa
gi|1019420690|emb|SAL33571_1|~Paraburkholderia_humi
gi|1019473539|emb|SAL60260_1|~Paraburkholderia_terrestris
gi|495009839~Burkholderia_sp__BT03
gi|73536184|dbj|BAP28520_1|~uncultured_bacterium
gi|764676922~Pandoraea_vervacti
gi|740834947~Pandoraea_pnomenusa
gi|827454738~Pandoraea_thiooxydans
gi|916629468~Ottowia_thiooxydans
gi|495869061~Nitratireductor_pacificus_pht-3B
gi|1011956543~Falsirhodobacter_sp__alg1
gi|960381550~Ruegeria_atlantica
gi|960385452~Ruegeria_atlantica
gi|496032412~Rhodobacteraceae_bacterium_KLH11
gi|746488700~Ruegeria_sp__ANG-S4
gi|974809920~Rhodobacteraceae_bacterium_O3_65
gi|1041526319~Woeseia_oceani
gi|503726712~Roseobacter_litoralis_Och_149
gi|499888997~Roseobacter_denitrificans_OCh_114
gi|1011641435~Labrenzia_sp__CP4
gi|494668193~Oceanivolvalibus_guishainsula_JLT2003
gi|652994147~Phaeobacter_inhibens
gi|568330882~Phaeobacter_gallaeciensis_DSM_26640
gi|655999879~Cucumibacter_marinus
gi|1015800324~Rhizobium_leguminosarum
gi|739263252~Rhizobium_sp__CF097
gi|764923009~Nesterenkonia_sp__NP1
gi|501123785~Azorhizobium_caulinodans_ORS_571
gi|652382526~Polaromonas_sp__EUR3_1_2_1
gi|937399441~Aliiroseovarius_crassostreae
gi|1016937626~Aliiroseovarius_crassostreae
gi|653010092~Sedimentitalea_nanhaiensis
gi|1032100811~Jannaschia_sp__EhC01
gi|653030268~Roseomonas_gilardii
gi|518290273~Roseomonas_mucosa
gi|494679064~Acetobacteraceae_bacterium_AT-5844
gi|1002735346~Rhodoplanes_sp__Z2-YC6860
gi|499899433~Chelativorans_sp__BNC1
gi|550363108~Vibrio_nigripulchritudo_SOn1
gi|1024465198~Oceanibulbus_sp__HI0129
gi|924895255~Sphingopyxis_sp__113P3
gi|1055110594~Sphingobium_sp__TCM1
gi|494893440~Paraglaciecola_arctica_BSs20135
gi|644239641~Sphingomonas_sanxanigenens_DSM_19645_=_NX02
gi|516072504~Sphingobium_yanoikuyae
gi|551338791~Spongiibacter_tropicus
gi|507071152~Acinetobacter_pittii_ANC_4050
gi|503972043~Acinetobacter_pittii_PHEA-2
gi|1053341149~Acinetobacter_pittii
gi|799150912~Alcaligenes_faecalis
gi|755131687~Achromobacter_sp__ATCC8750
gi|737289447~Alcaligenes_faecalis
gi|491489825~Aeromonas_veronii_AMC34
gi|736863381~Enterobacteriaceae_bacterium_B14
gi|971844446~Erwinia_sp__SCU-B244
gi|558558493~Serratia_sp__DD3
gi|546179905~Halomonas_huangheensis
gi|655260725~Saccharospirillum_impatiens
gi|1055648585~Marinobacterium_profundum
gi|950134594~Bosea_sp__Root483D1
gi|947405795~Bosea_sp__WAO
gi|1011013504~Bosea_sp__Root670
gi|517241935~Hoeflea_sp__108
gi|932309362~Mesorhizobium_sp__1M-11
gi|1032816481~Ochrobactrum_pseudogrignonense
gi|522188755~Reyranella_massiliensis
gi|1056030799~Oceanibaculum_pacificum
gi|949018873~Nitrosopumilus_sp__BACL13_MAG-120910-bin56
gi|495572926~Candidatus_Nitrosopumilus_salaria_BD31
gi|504778456~Candidatus_Nitrosopumilus_sp__AR2
gi|851130181~Marine_Group_I_thaumarchaeote_SCGC_RSA3
gi|851212169~Candidatus_Nitrosoarchaeum_koreensis
gi|339520440~Candidatus_Nitrosoarchaeum_koreensis_MY1
gi|329137876~Candidatus_Nitrosoarchaeum_limnia_SFB1
gi|952338703~Thaumarchaeota_archaeon_CSP1-1
gi|663352342~uncultured_marine_thaumarchaeote_KM3_70_E10
gi|563488593|emb|CDI05972_1|~Thaumarchaeota_archaeon_N4
gi|851361128~Thaumarchaeota_archaeon_N4
gi|757132937~Thaumarchaeota_archaeon_MY2
gi|851342276~Candidatus_Nitrosotenuis_cloacae
gi|663516571~uncultured_marine_thaumarchaeote_KM3_198_G09
gi|663524990~uncultured_marine_thaumarchaeote_KM3_70_B03
gi|663512678~uncultured_marine_thaumarchaeote_KM3_162_H04
gi|663533291~uncultured_marine_thaumarchaeote_SAT1000_13_G01
gi|663509947~uncultured_marine_thaumarchaeote_KM3_67_E09
gi|663512267~uncultured_marine_thaumarchaeote_KM3_76_B07
gi|663510712~uncultured_marine_thaumarchaeote_KM3_146_F08
gi|167042372~uncultured_marine_crenarchaeote_HF4000_ANIW97M7
gi|663522325~uncultured_marine_thaumarchaeote_KM3_56_D04
gi|1003584702|emb|CUR51987_1|~Candidatus_Nitrosotalea_devanaterra
gi|665993999~Candidatus_Nitrosphaera_evergladensis_SR1
gi|408365050~Candidatus_Nitrososphaera_gargensis_Ga9_2
gi|503449617~Archaeoglobus_veneficus_SNP6
gi|502803442~Methanohalophilus_mahii_DSM_5219
gi|1001926175~Methanohalophilus_sp__DAL1
gi|1001919190~Methanolobus_sp__T82-4
gi|805404012~Methanosarcina_barkeri_CM1
gi|805386574~Methanosarcina_barkeri_3
gi|973082939~Methanosaeta_harundinacea
gi|504398486~Methanosaeta_harundinacea_6Ac
gi|116665012~Methanosaeta_thermophila_PT
gi|502945333~Methanococcus_voltae_A3
gi|516896405~Methanothermococcus_thermolithotrophicus
gi|851157741~Candidatus_Methanoperedens_nitroreducens
gi|630830894~Candidatus_Methanoperedens_nitroreducens
gi|471673933~Thermoplasmatales_archaeon_SCGC_AB-539-N05
gi|985657681~candidate_divison_MSBL1_archaeon_SCGC-AAA259A05
gi|985663772~candidate_divison_MSBL1_archaeon_SCGC-AAA259E19
gi|985677866~candidate_divison_MSBL1_archaeon_SCGC-AAA385D11
gi|1053790436~delta_proteobacterium_HotSeep1
gi|1007510533~Candidatus_Bathyarchaeota_archaeon_B26-1
gi|493157514~Natrialba_chahannaoensis_JCM_10990
gi|910245567~Halostagnicola_sp__A56
gi|541180760~halophilic_archaeon_J07HX5
gi|947972294~Paenibacillus_sp__Soil522
gi|835838273~Paenibacillus_chondroitinus
gi|517952108~Brevibacillus_massiliensis
gi|783154484~Paenibacillus_beijingensis
gi|516856461~Effusibacillus_pohliae
gi|758786821~Aneurinibacillus_migulanus
gi|917806586|emb|CEH29856_1|~Aneurinibacillus_migulanus
gi|825930097~Aneurinibacillus_tyrosinisolvens
gi|923047363~Sporosarcina_globispora
gi|1006540168~Bacillus_firmus
gi|651589953~Bacillus_sp__J33
gi|1054508792~Fictibacillus_arsenicus
gi|654947446~Bacillus_sp__FJAT-14578
gi|1054684580~Halomonas_chromatireducens
gi|498315921~Halomonas_sp__KM-1
gi|655390807~Thalassobaculum_salexigens
gi|494943759~alpha_proteobacterium_BAL199
gi|1056031162~Oceanibaculum_pacificum
gi|496229342~Oceanibaculum_indicum_P24
gi|916543104~Reyranella_massiliensis
gi|502636155~Sphaerobacter_thermophilus_DSM_20745
gi|1050128431~Streptomyces_mutomycini
gi|928889377~Streptomyces_sp__NRRL_S-4
gi|985847262~Streptomyces_diastatochromogenes
gi|742857928~Arthrobacter_sp__W1
gi|518464581~Actinomadura_atramentaria
GSMAST4A1_ASY1_ANO1_6063_1
gi|738204887~Marinomonas_ushuaiensis_DSM_15871
gi|489906604~Bordetella_bronchiseptica
gi|1011861654~Achromobacter_denitrificans
0.1
Archaea
Bacteria
SAG
NA

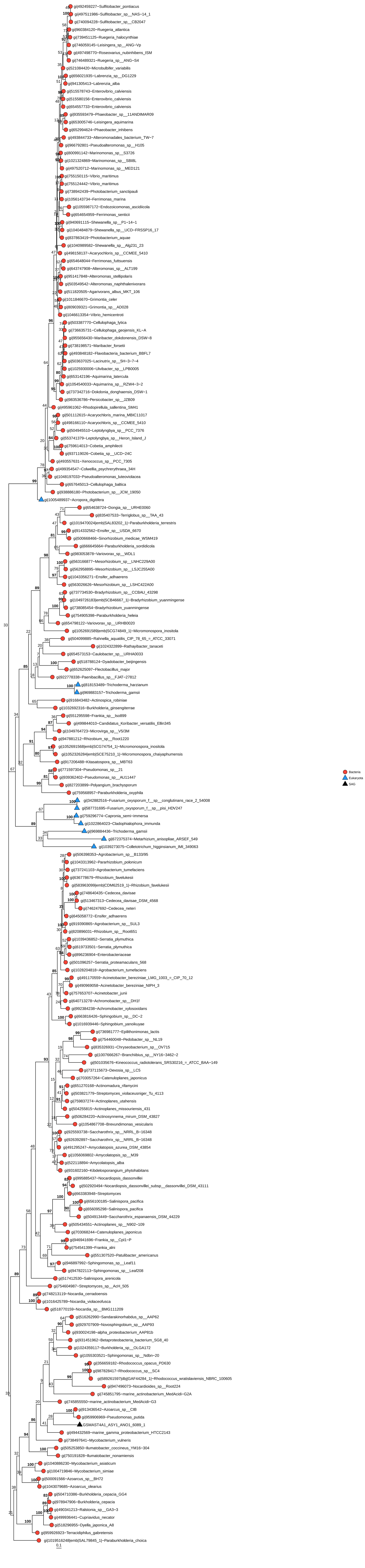

Bacteria
Eukaryota
SAG
0.1

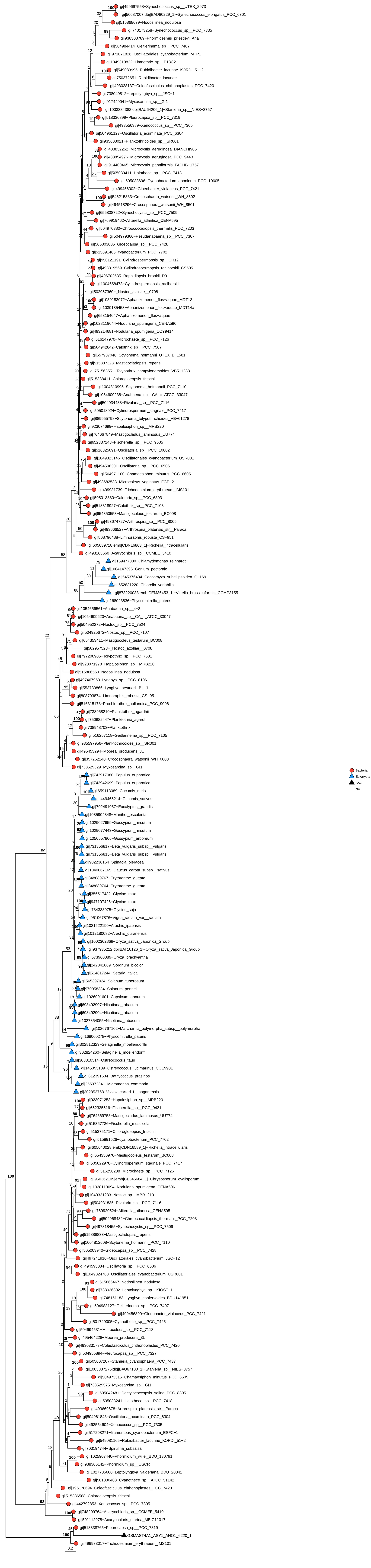
Bacteria
Eukaryota
SAG
NA
0.2

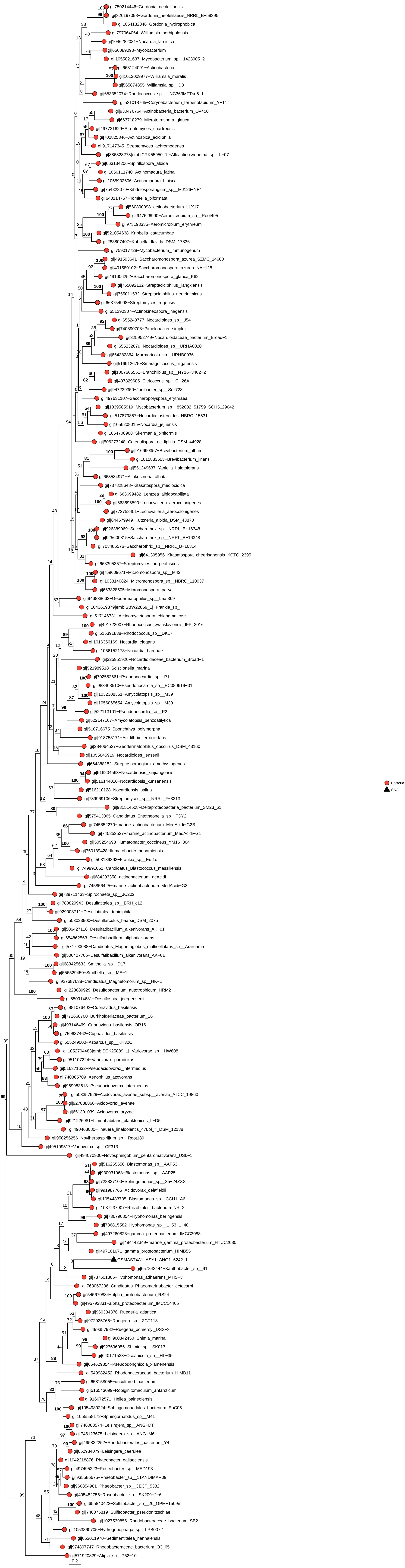

Bacteria
SAG

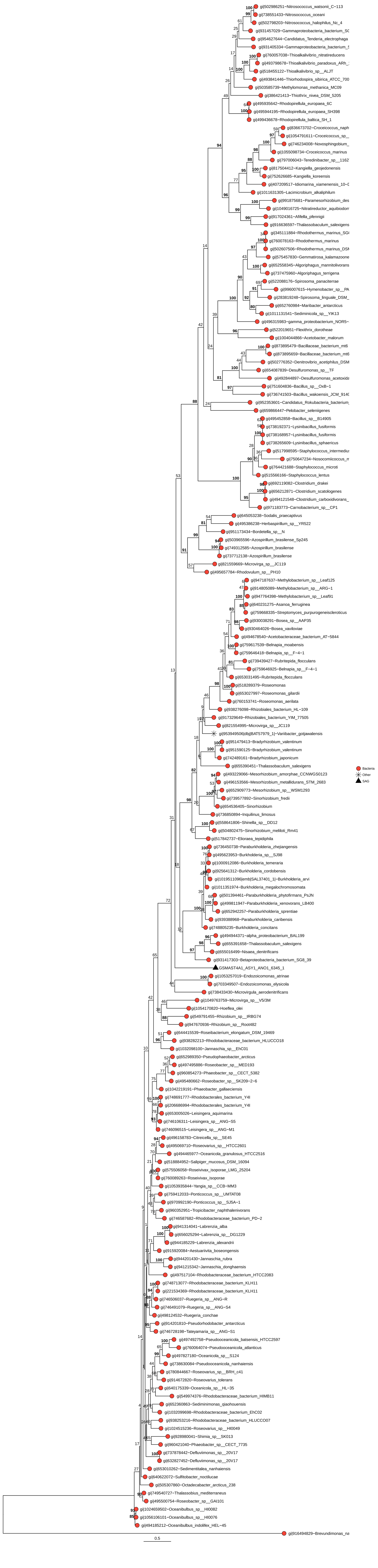

Bacteria
Other
SAG
0.5

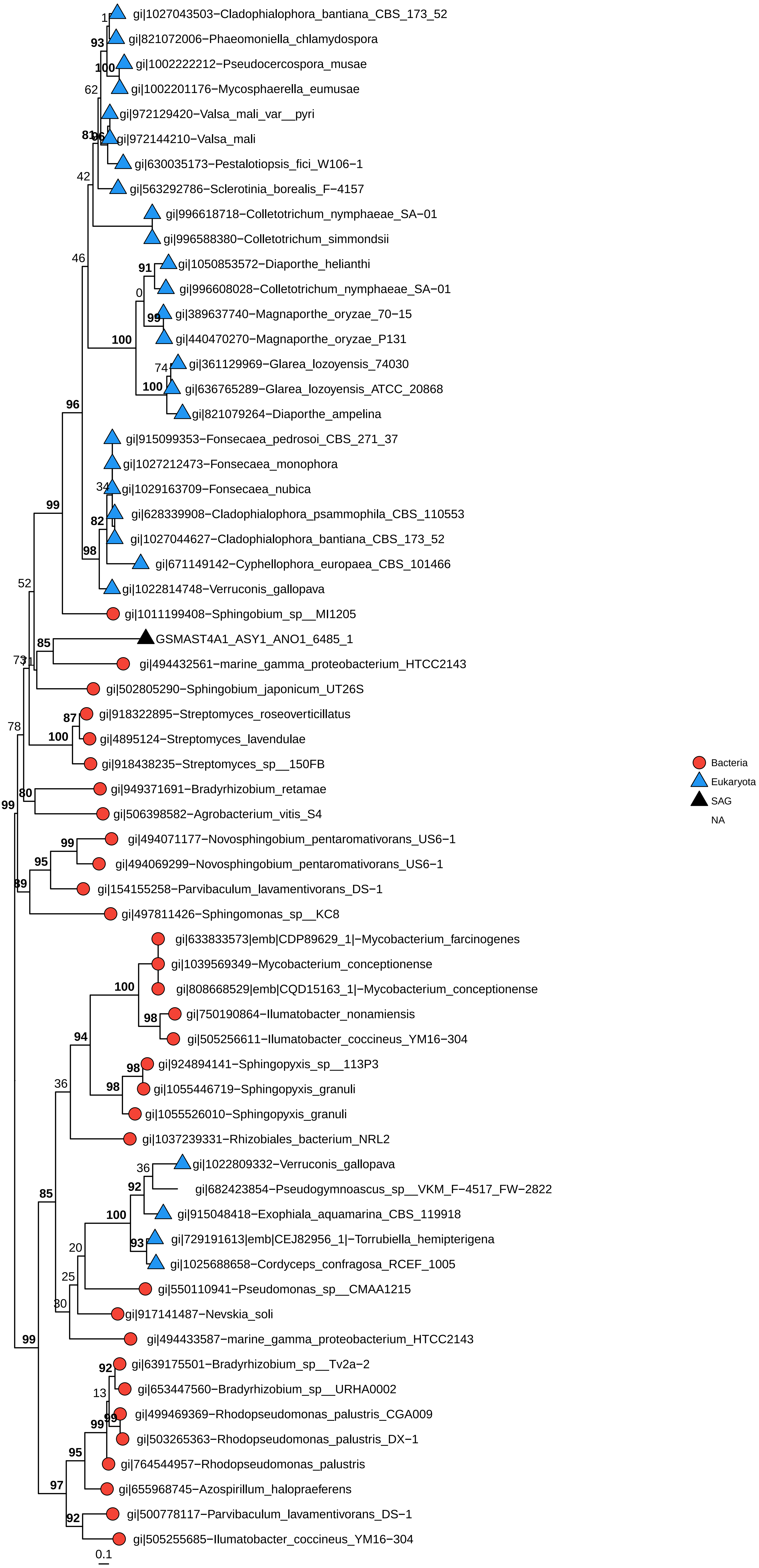
gi|1027043503−Cladophialophora_bantiana_CBS_173_52
gi|821072006−Phaeomoniella_chlamydospora
gi|1002222212−Pseudocercospora_musae
gi|1002201176−Mycosphaerella_eumusae
gi|972129420−Valsa_mali_var__pyri
gi|972144210−Valsa_mali
gi|630035173−Pestalotiopsis_fici_W106−1
gi|563292786−Sclerotinia_borealis_F−4157
gi|996618718−Colletotrichum_nymphaeae_SA−01
gi|996588380−Colletotrichum_simmondsii
gi|1050853572−Diaporthe_helianthi
gi|996608028−Colletotrichum_nymphaeae_SA−01
gi|389637740−Magnaporthe_oryzae_70−15
gi|440470270−Magnaporthe_oryzae_P131
gi|361129969−Glarea_lozoyensis_74030
gi|636765289−Glarea_lozoyensis_ATCC_20868
gi|821079264−Diaporthe_ampelina
gi|915099353−Fonsecaea_pedrosoi_CBS_271_37
gi|1027212473−Fonsecaea_monophora
gi|1029163709−Fonsecaea_nubica
gi|628339908−Cladophialophora_psammophila_CBS_110553
gi|1027044627−Cladophialophora_bantiana_CBS_173_52
gi|671149142−Cyphellophora_europaea_CBS_101466
gi|1022814748−Verruconis_gallopava
gi|1011199408−Sphingobium_sp__MI1205
GSMAST4A1_ASY1_ANO1_6485_1
gi|494432561−marine_gamma_proteobacterium_HTCC2143
gi|502805290−Sphingobium_japonicum_UT26S
gi|918322895−Streptomyces_roseoverticillatus
gi|4895124−Streptomyces_lavendulae
gi|918438235−Streptomyces_sp__150FB
gi|949371691−Bradyrhizobium_retamae
gi|506398582−Agrobacterium_vitis_S4
gi|494071177−Novosphingobium_pentaromativorans_US6−1
gi|494069299−Novosphingobium_pentaromativorans_US6−1
gi|154155258−Parvibaculum_lavamentivorans_DS−1
gi|497811426−Sphingomonas_sp__KC8
gi|633833573|emb|CDP89629_1|−Mycobacterium_farcinogenes
gi|1039569349−Mycobacterium_conceptionense
gi|808668529|emb|CQD15163_1|−Mycobacterium_conceptionense
gi|750190864−Ilumatobacter_nonamiensis
gi|505256611−Ilumatobacter_coccineus_YM16−304
gi|924894141−Sphingopyxis_sp__113P3
gi|1055446719−Sphingopyxis_granuli
gi|1055526010−Sphingopyxis_granuli
gi|1037239331−Rhizobiales_bacterium_NRL2
gi|1022809332−Verruconis_gallopava
gi|682423854−Pseudogymnoascus_sp__VKM_F−4517_FW−2822
gi|915048418−Exophiala_aquamarina_CBS_119918
gi|729191613|emb|CEJ82956_1|−Torrubiella_hemipterigena
gi|1025688658−Cordyceps_confragosa_RCEF_1005
gi|550110941−Pseudomonas_sp__CMAA1215
gi|917141487−Nevskia_soli
gi|494433587−marine_gamma_proteobacterium_HTCC2143
gi|639175501−Bradyrhizobium_sp__Tv2a−2
gi|653447560−Bradyrhizobium_sp__URHA0002
gi|499469369−Rhodopseudomonas_palustris_CGA009
gi|503265363−Rhodopseudomonas_palustris_DX−1
gi|764544957−Rhodopseudomonas_palustris
gi|655968745−Azospirillum_halopraeferens
gi|500778117−Parvibaculum_lavamentivorans_DS−1
gi|505255685−Ilumatobacter_coccineus_YM16−304
0.1
Bacteria
Eukaryota
SAG
NA

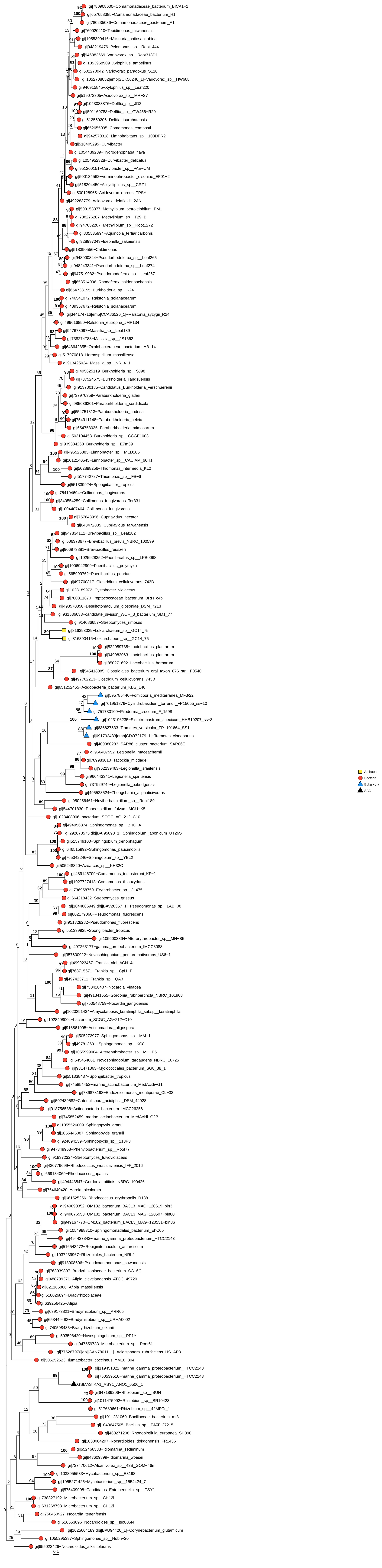
gi|780908600~Comamonadaceae_bacterium_BICA1-1
gi|657658385~Comamonadaceae_bacterium_H1
gi|780235036~Comamonadaceae_bacterium_A1
gi|760020410~Tepidimonas_taiwanensis
gi|1055399416~Mitsuaria_chitosanitabida
gi|948219476~Pelomonas_sp__Root1444
gi|946883669~Variovorax_sp__Root318D1
gi|1053968909~Xylophilus_ampelinus
gi|502270942~Variovorax_paradoxus_S110
gi|1052708052|emb|SCK56246_1|~Variovorax_sp__HW608
gi|946915845~Xylophilus_sp__Leaf220
gi|519072305~Acidovorax_sp__MR-S7
gi|1043083876~Delftia_sp__JD2
gi|501160788~Delftia_sp__GW456-R20
gi|512559206~Delftia_tsuruhatensis
gi|652655095~Comamonas_composti
gi|942570318~Limnohabitans_sp__103DPR2
gi|518405295~Curvibacter
gi|1054439289~Hydrogenophaga_flava
gi|1054952328~Curvibacter_delicatus
gi|951200151~Curvibacter_sp__PAE-UM
gi|500134562~Verminephrobacter_eiseniae_EF01-2
gi|518204450~Alicycliphilus_sp__CRZ1
gi|500128965~Acidovorax_ebreus_TPSY
gi|492283779~Acidovorax_delafieldii_2AN
gi|500153377~Methylibium_petroleiphilum_PM1
gi|738276207~Methylibium_sp__T29-B
gi|947652207~Methylibium_sp__Root1272
gi|805535994~Aquincola_tertiaricarbonis
gi|928997049~Ideonella_sakaiensis
gi|518390556~Caldimonas
gi|948000844~Pseudorhodoferax_sp__Leaf265
gi|948243341~Pseudorhodoferax_sp__Leaf274
gi|947519982~Pseudorhodoferax_sp__Leaf267
gi|658514096~Rhodoferax_saidenbachensis
gi|654738155~Burkholderia_sp__K24
gi|746541072~Ralstonia_solanacearum
gi|489357672~Ralstonia_solanacearum
gi|344174716|emb|CCA86526_1|~Ralstonia_syzygii_R24
gi|499616850~Ralstonia_eutropha_JMP134
gi|947673097~Massilia_sp__Leaf139
gi|738274788~Massilia_sp__JS1662
gi|648642855~Oxalobacteraceae_bacterium_AB_14
gi|517970818~Herbaspirillum_massiliense
gi|913425024~Massilia_sp__NR_4-1
gi|495625119~Burkholderia_sp__SJ98
gi|737524575~Burkholderia_jiangsuensis
gi|913700185~Candidatus_Burkholderia_verschuerenii
gi|737970359~Paraburkholderia_glathei
gi|985636301~Paraburkholderia_sordidicola
gi|654751813~Paraburkholderia_nodosa
gi|754911148~Paraburkholderia_heleia
gi|654758035~Paraburkholderia_mimosarum
gi|503104453~Burkholderia_sp__CCGE1003
gi|939384260~Burkholderia_sp__E7m39
gi|495525383~Limnobacter_sp__MED105
gi|1012140545~Limnobacter_sp__CACIAM_66H1
gi|502888256~Thiomonas_intermedia_K12
gi|517742787~Thiomonas_sp__FB-6
gi|551339924~Spongiibacter_tropicus
gi|754104694~Collimonas_fungivorans
gi|340554259~Collimonas_fungivorans_Ter331
gi|1004407464~Collimonas_fungivorans
gi|757643996~Cupriavidus_necator
gi|648472835~Cupriavidus_taiwanensis
gi|947834111~Brevibacillus_sp__Leaf182
gi|506373677~Brevibacillus_brevis_NBRC_100599
gi|906973881~Brevibacillus_reuszeri
gi|1025928352~Paenibacillus_sp__LPB0068
gi|1006942909~Paenibacillus_polymyxa
gi|565999762~Paenibacillus_peoriae
gi|497760817~Clostridium_cellulovorans_743B
gi|1028189972~Cystobacter_violaceus
gi|780811670~Peptococcaceae_bacterium_BRH_c4b
gi|493570850~Desulfotomaculum_gibsoniae_DSM_7213
gi|931536633~candidate_division_WOR_3_bacterium_SM1_77
gi|914086657~Streptomyces_rimosus
gi|816393029~Lokiarchaeum_sp__GC14_75
gi|816390416~Lokiarchaeum_sp__GC14_75
gi|822089738~Lactobacillus_plantarum
gi|949982063~Lactobacillus_plantarum
gi|850271692~Lactobacillus_herbarum
gi|545418085~Clostridiales_bacterium_oral_taxon_876_str__F0540
gi|497762213~Clostridium_cellulovorans_743B
gi|651252455~Acidobacteria_bacterium_KBS_146
gi|595785446~Fomitiporia_mediterranea_MF3/22
gi|761951876~Cylindrobasidium_torrendii_FP15055_ss-10
gi|751730109~Piloderma_croceum_F_1598
gi|1023196235~Sistotremastrum_suecicum_HHB10207_ss-3
gi|636627533~Trametes_versicolor_FP-101664_SS1
gi|691792433|emb|CDO72179_1|~Trametes_cinnabarina
gi|409980283~SAR86_cluster_bacterium_SAR86E
gi|966407552~Legionella_maceachernii
gi|769983010~Tatlockia_micdadei
gi|962239463~Legionella_israelensis
gi|966443341~Legionella_spiritensis
gi|737929749~Legionella_oakridgensis
gi|495523524~Zhongshania_aliphaticivorans
gi|950256461~Novilerbaspirillum_sp__Root189
gi|544701830~Phaeospirillum_fulvum_MGU-K5
gi|1028408006~bacterium_SCGC_AG-212-C10
gi|494956874~Sphingomonas_sp__BHC-A
gi|292673575|dbj|BAI95093_1|~Sphingobium_japonicum_UT26S
gi|515749100~Sphingobium_xenophagum
gi|646515992~Sphingomonas_paucimobilis
gi|765342246~Sphingobium_sp__YBL2
gi|505248820~Azoarcus_sp__KH32C
gi|489146709~Comamonas_testosteroni_KF-1
gi|1027727418~Comamonas_thiooxydans
gi|736958759~Erythrobacter_sp__JL475
gi|664218432~Streptomyces_griseus
gi|1044866949|dbj|BAV26357_1|~Pseudomonas_sp__LAB-08
gi|802179060~Pseudomonas_fluorescens
gi|951328282~Pseudomonas_fluorescens
gi|551339925~Spongiibacter_tropicus
gi|1056003864~Altererythrobacter_sp__MH-B5
gi|497263177~gamma_proteobacterium_IMCC3088
gi|357600922~Novosphingobium_pentaromativorans_US6-1
gi|499923467~Frankia_alni_ACN14a
gi|768715671~Frankia_sp__CpI1-P
gi|497423711~Frankia_sp__QA3
gi|750418407~Nocardia_vinacea
gi|491341555~Gordonia_rubripertincta_NBRC_101908
gi|750548759~Nocardia_jiangxiensis
gi|1020291434~Amycolatopsis_keratiniphila_subsp__keratiniphila
gi|1028408004~bacterium_SCGC_AG-212-C10
gi|916861095~Actinomadura_oligospora
gi|505272977~Sphingomonas_sp__MM-1
gi|497813691~Sphingomonas_sp__KC8
gi|1055099004~Altererythrobacter_sp__MH-B5
gi|545454061~Novosphingobium_tardaugens_NBRC_16725
gi|931471363~Myxococcales_bacterium_SG8_38_1
gi|551338437~Spongiibacter_tropicus
gi|745854452~marine_actinobacterium_MedAcidi-G1
gi|736873193~Endozoicomonas_montiporae_CL-33
gi|502439582~Catenulispora_acidiphila_DSM_44928
gi|918756588~Actinobacteria_bacterium_IMCC26256
gi|745852459~marine_actinobacterium_MedAcidi-G2B
gi|1055526009~Sphingopyxis_granuli
gi|1055445087~Sphingopyxis_granuli
gi|922894139~Sphingopyxis_sp__113P3
gi|947349968~Phenylobacterium_sp__Root77
gi|918372324~Streptomyces_fulvoviolaceus
gi|430779699~Rhodococcus_wratislaviensis_IFP_2016
gi|669184069~Rhodococcus_opacus
gi|494443847~Gordonia_otitidis_NBRC_100426
gi|764640420~Agreia_bicolorata
gi|661525255~Rhodococcus_erythropolis_R138
gi|949090352~OM182_bacterium_BACL3_MAG-120619-bin3
gi|949076553~OM182_bacterium_BACL3_MAG-120507-bin80
gi|949167770~OM182_bacterium_BACL3_MAG-120531-bin86
gi|1054988310~Sphingomonadales_bacterium_EhC05
gi|494427842~marine_gamma_proteobacterium_HTCC2143
gi|516543472~Robiginitomaculum_antarcticum
gi|1037239967~Rhizobiales_bacterium_NRL2
gi|918908696~Pseudoxanthomonas_suwonensis
gi|763039897~Bradyrhizobiaceae_bacterium_SG-6C
gi|488799371~Afipia_clevelandensis_ATCC_49720
gi|821185866~Afipia_massiliensis
gi|518026894~Bradyrhizobiaceae
gi|639256425~Afipia
gi|639173821~Bradyrhizobium_sp__ARR65
gi|653449482~Bradyrhizobium_sp__URHA0002
gi|740598485~Bradyrhizobium_elkanii
gi|503598420~Novosphingobium_sp__PP1Y
gi|947559733~Microbacterium_sp__Root61
gi|775267970|dbj|GAN78011_1|~Acidisphaera_rubrifaciens_HS-AP3
gi|505252523~Illumatobacter_coccineus_YM16-304
gi|119451322~marine_gamma_proteobacterium_HTCC2143
gi|750539510~marine_gamma_proteobacterium_HTCC2143
GSMAST4A1_ASY1_ANO1_6506_1
gi|647189206~Rhizobium_sp__IBUN
gi|1011475992~Rhizobium_sp__BR10423
gi|517689661~Rhizobium_sp__42MFCr_1
gi|1011281060~Bacillaceae_bacterium_mt8
gi|1043647505~Bacillus_sp__FJAT-27215
gi|460271228~Rhodopirellula_europaea_SH398
gi|1033004297~Nocardioides_dokdonensis_FR1436
gi|652466333~Idiomarina_sediminum
gi|943609899~Idiomarina_woesei
gi|737470612~Alcanivorax_sp__43B_GOM-46m
gi|1038055533~Mycobacterium_sp__E3198
gi|1055271425~Mycobacterium_sp__1554424_7
gi|575409008~Candidatus_Entotheonella_sp__TSY1
gi|738327192~Microbacterium_sp__CH12i
gi|631268798~Microbacterium_sp__CH12i
gi|750460927~Nocardia_tenerifensis
gi|516553096~Nocardioides_sp__Iso805N
gi|1025604189|dbj|BAU94420_1|~Corynebacterium_glutamicum
gi|1055295387~Sphingomonas_sp__Ndbn-20
gi|655023426~Nocardioides_alkalitolerans
0.1
Archaea
Bacteria
Eukaryota
SAG

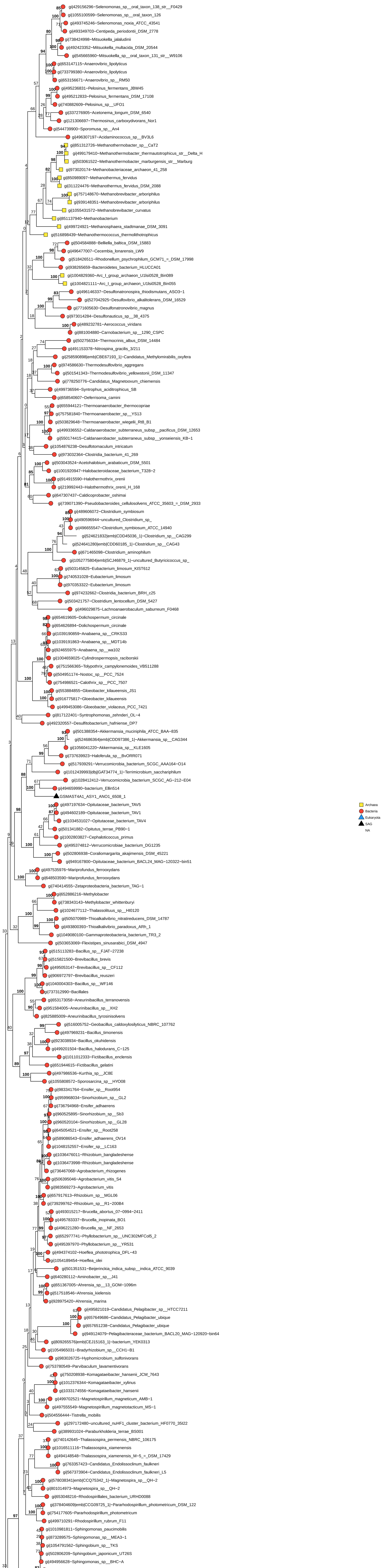
Archaea
Bacteria
Eukaryota
SAG
NA

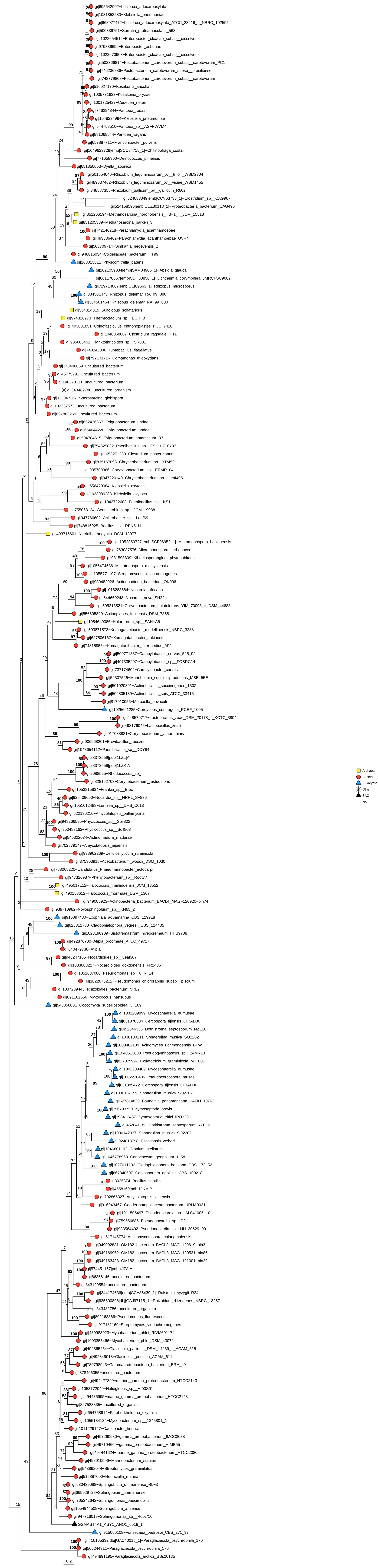
Archaea
Bacteria
Eukaryota
Other
SAG
NA
0.2

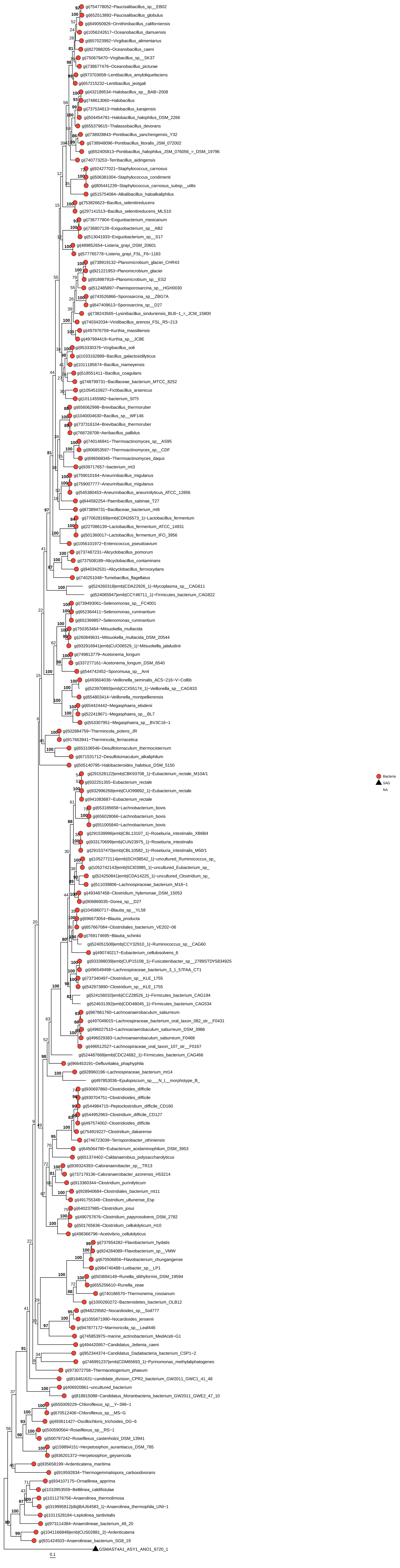

gi|754778052−Paucisalibacillus_sp__EB02
gi|652513892−Paucisalibacillus_globulus
gi|849050926−Ornithinibacillus_californiensis
gi|1056242617−Oceanobacillus_damuensis
gi|657023982−Virgibacillus_alimentarius
gi|827088205−Oceanobacillus_caeni
gi|750679470−Virgibacillus_sp__SK37
gi|738677476−Oceanobacillus_picturae
gi|973703658−Lentibacillus_amyloliquefaciens
gi|657215232−Lentibacillus_jeotgali
gi|432189534−Halobacillus_sp__BAB−2008
gi|748613060−Halobacillus
gi|737534613−Halobacillus_karajensis
gi|504454781−Halobacillus_halophilus_DSM_2266
gi|655379615−Thalassobacillus_devorans
gi|738928843−Pontibacillus_yanchengensis_Y32
gi|738948096−Pontibacillus_litoralis_JSM_072002
gi|652405813−Pontibacillus_halophilus_JSM_076056_=_DSM_19796
gi|740773253−Terribacillus_aidingensis
gi|924277021−Staphylococcus_carnosus
gi|506381004−Staphylococcus_condimenti
gi|805441239−Staphylococcus_carnosus_subsp__utilis
gi|515754084−Alkalibacillus_haloalkaliphilus
gi|753826623−Bacillus_selenitireducens
gi|297141513−Bacillus_selenitireducens_MLS10
gi|736777904−Exiguobacterium_mexicanum
gi|736807128−Exiguobacterium_sp__AB2
gi|513041933−Exiguobacterium_sp__S17
gi|489852654−Listeria_grayi_DSM_20601
gi|577765778−Listeria_grayi_FSL_F6−1183
gi|738919132−Planomicrobium_glaciei_CHR43
gi|921221953−Planomicrobium_glaciei
gi|918987918−Planomicrobium_sp__ES2
gi|512485897−Paenisporosarcina_sp__HGH0030
gi|743526866−Sporosarcina_sp__ZBG7A
gi|647409613−Sporosarcina_sp__D27
gi|738243565−Lysinibacillus_sinduriensis_BLB−1_=_JCM_15800
gi|740342034−Viridibacillus_arenosi_FSL_R5−213
gi|497976759−Kurthia_massiliensis
gi|497994419−Kurthia_sp__JC8E
gi|953330376−Virgibacillus_soli
gi|1033162889−Bacillus_galactosidilyticus
gi|1011185674−Bacillus_niameyensis
gi|518551411−Bacillus_coagulans
gi|748799731−Bacillaceae_bacterium_MTCC_8252
gi|1054510927−Fictibacillus_arsenicus
gi|1011455982−bacterium_SIT5
gi|656062998−Brevibacillus_thermoruber
gi|1040004630−Bacillus_sp__WF146
gi|737316104−Brevibacillus_thermoruber
gi|768728708−Aeribacillus_pallidus
gi|740146941−Thermoactinomyces_sp__AS95
gi|906853597−Thermoactinomyces_sp__CDF
gi|696568345−Thermoactinomyces_daqus
gi|939717657−bacterium_mt3
gi|759010164−Aneurinibacillus_migulanus
gi|759007777−Aneurinibacillus_migulanus
gi|545380453−Aneurinibacillus_aneurinilyticus_ATCC_12856
gi|644582254−Paenibacillus_sabinae_T27
gi|873894731−Bacillaceae_bacterium_mt6
gi|770628169|emb|CDN26573_1|−Lactobacillus_fermentum
gi|227086139−Lactobacillus_fermentum_ATCC_14931
gi|501360017−Lactobacillus_fermentum_IFO_3956
gi|1056101972−Enterococcus_pseudoavium
gi|737487231−Alicyclobacillus_pomorum
gi|737508189−Alicyclobacillus_contaminans
gi|940342531−Alicyclobacillus_ferrooxydans
gi|740251048−Tumebacillus_flagellatus
gi|524260318|emb|CDA22926_1|−Mycoplasma_sp__CAG611
gi|524065947|emb|CCY46711_1|−Firmicutes_bacterium_CAG822
gi|739493061−Selenomonas_sp__FC4001
gi|652364411−Selenomonas_ruminantium
gi|652369857−Selenomonas_ruminantium
gi|750353464−Mitsuokella_multacida
gi|260849631−Mitsuokella_multacida_DSM_20544
gi|932916941|emb|CUO06529_1|−Mitsuokella_jalaludinii
gi|749813779−Acetonema_longum
gi|337277161−Acetonema_longum_DSM_6540
gi|544742452−Sporomusa_sp__An4
gi|493604036−Veillonella_seminalis_ACS−216−V−Col6b
gi|523970893|emb|CCX55174_1|−Veillonella_sp__CAG933
gi|654803414−Veillonella_montpellierensis
gi|654424442−Megasphaera_elsdenii
gi|522418671−Megasphaera_sp__BL7
gi|553307951−Megasphaera_sp__BV3C16−1
gi|502884759−Thermincola_potens_JR
gi|917663941−Thermincola_ferriacetica
gi|653106546−Desulfotomaculum_thermocisternum
gi|671531712−Desulfotomaculum_alkaliphilum
gi|505140795−Halobacteroides_halobius_DSM_5150
gi|291528122|emb|CBK93708_1|−Eubacterium_rectale_M104/1
gi|502251355−Eubacterium_rectale
gi|932996269|emb|CUO99892_1|−Eubacterium_rectale
gi|941083687−Eubacterium_rectale
gi|653185658−Lachnobacterium_bovis
gi|656028066−Lachnobacterium_bovis
gi|551005840−Lachnobacterium_bovis
gi|291539996|emb|CBL13107_1|−Roseburia_intestinalis_XB6B4
gi|933170699|emb|CUN23975_1|−Roseburia_intestinalis
gi|291537470|emb|CBL10582_1|−Roseburia_intestinalis_M50/1
gi|1052772114|emb|SCH38542_1|−uncultured_Ruminococcus_sp_
gi|1052742143|emb|SCI03985_1|−uncultured_Eubacterium_sp_
gi|524250841|emb|CDA14225_1|−uncultured_Clostridium_sp_
gi|511039806−Lachnospiraceae_bacterium_M18−1
gi|493487458−Clostridium_hylemonae_DSM_15053
gi|906869035−Dorea_sp__D27
gi|1045860717−Blautia_sp__YL58
gi|696673054−Blautia_producta
gi|657667084−Clostridiales_bacterium_VE202−06
gi|769174695−Blautia_schinkii
gi|524051508|emb|CCY32910_1|−Ruminococcus_sp__CAG60
gi|490740217−Eubacterium_cellulosolvens_6
gi|933398039|emb|CUP15108_1|−Fusicatenibacter_sp__2789STDY5834925
gi|496549498−Lachnospiraceae_bacterium_3_1_57FAA_CT1
gi|737340497−Clostridium_sp__KLE_1755
gi|542973890−Clostridium_sp__KLE_1755
gi|524156032|emb|CCZ28526_1|−Firmicutes_bacterium_CAG194
gi|524631392|emb|CDD48045_1|−Firmicutes_bacterium_CAG534
gi|987861760−Lachnoanaerobaculum_saburreum
gi|497049015−Lachnospiraceae_bacterium_oral_taxon_082_str__F0431
gi|496027510−Lachnoanaerobaculum_saburreum_DSM_3986
gi|496029383−Lachnoanaerobaculum_saburreum_F0468
gi|496512527−Lachnospiraceae_oral_taxon_107_str__F0167
gi|524487668|emb|CDC24682_1|−Firmicutes_bacterium_CAG466
gi|966453191−Defluviitalea_phaphyphila
gi|928960196−Lachnospiraceae_bacterium_mt14
gi|497853036−Epulopiscium_sp___N_t__morphotype_B_
gi|930697860−Clostridioides_difficile
gi|930704751−Clostridioides_difficile
gi|544984715−Peptoclostridium_difficile_CD160
gi|544952963−Clostridium_difficile_CD127
gi|497574002−Clostridioides_difficile
gi|754919227−Clostridium_dakarense
gi|746723039−Terrisporobacter_othiniensis
gi|645064780−Eubacterium_acidaminophilum_DSM_3953
gi|651374402−Caldanaerobius_polysaccharolyticus
gi|939324393−Caloranaerobacter_sp__TR13
gi|737179136−Caloranaerobacter_azorensis_H53214
gi|913360344−Clostridium_purinilyticum
gi|928940684−Clostridiales_bacterium_mt11
gi|491755346−Clostridium_ultunense_Esp
gi|640237985−Clostridium_josui
gi|490757676−Clostridium_papyrosolvens_DSM_2782
gi|501765636−Clostridium_cellulolyticum_H10
gi|498366796−Acetivibrio_cellulolyticus
gi|737654282−Flavobacterium_hydatis
gi|924284089−Flavobacterium_sp__VMW
gi|670506856−Flavobacterium_chungangense
gi|984740488−Lutibacter_sp__LP1
gi|503694149−Runella_slithyformis_DSM_19594
gi|655256610−Runella_zeae
gi|740186570−Thermonema_rossianum
gi|1000260272−Bacteroidetes_bacterium_OLB12
gi|948229582−Nocardioides_sp__Soil777
gi|1055871990−Nocardioides_jensenii
gi|947877172−Marmoricola_sp__Leaf446
gi|745853975−marine_actinobacterium_MedAcidi−G1
gi|494420957−Candidatus_Jettenia_caeni
gi|952344374−Candidatus_Dadabacteria_bacterium_CSP1−2
gi|746991237|emb|CDM65693_1|−Pyrinomonas_methylaliphatogenes
gi|973072758−Thermacetogenium_phaeum
gi|818461631−candidate_division_CPR2_bacterium_GW2011_GWC1_41_48
gi|406920861−uncultured_bacterium
gi|818815088−Candidatus_Moranbacteria_bacterium_GW2011_GWE2_47_10
gi|655009229−Chloroflexus_sp__Y−396−1
gi|670512406−Chloroflexus_sp__MS−G
gi|493611427−Oscillochloris_trichoides_DG−6
gi|500590564−Roseiflexus_sp__RS−1
gi|500797242−Roseiflexus_castenholzii_DSM_13941
gi|159894151−Herpetosiphon_aurantiacus_DSM_785
gi|936201372−Herpetosiphon_geysericola
gi|935658199−Ardenticatena_maritima
gi|919592834−Thermogemmatispora_carboxidivorans
gi|934107175−Ornatilinea_apprima
gi|1010953559−Bellilinea_caldifistulae
gi|1011276756−Anaerolinea_thermolimosa
gi|319995812|dbj|BAJ64583_1|−Anaerolinea_thermophila_UNI−1
gi|1011528184−Leptolinea_tardivitalis
gi|973114384−Anaerolineae_bacterium_49_20
gi|1041166848|emb|CUS02881_2|−Ardenticatena
gi|931424503−Anaerolineae_bacterium_SG8_19
GSMAST4A1_ASY1_ANO1_6720_1
0.1
Bacteria
SAG
NA

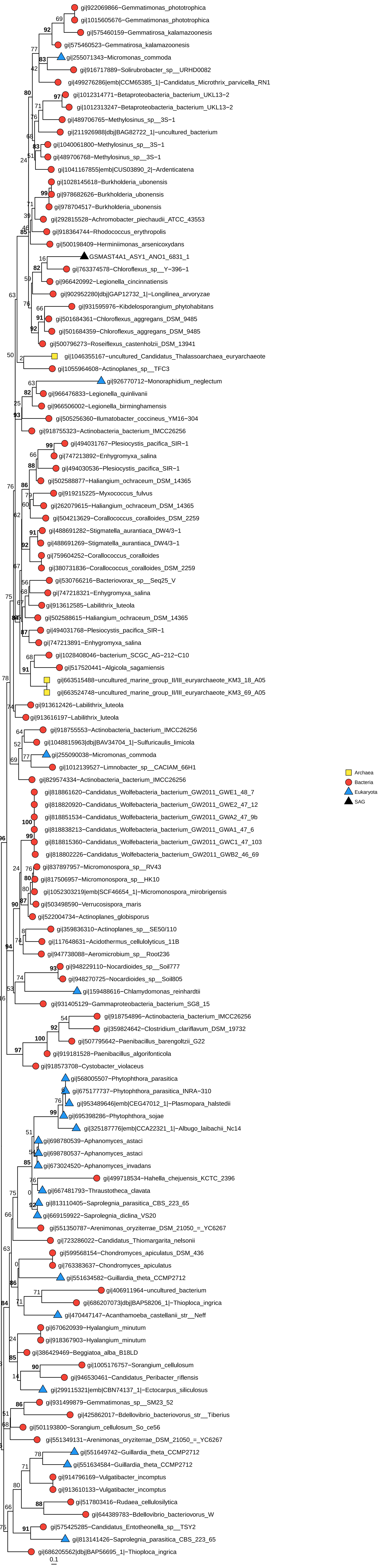

gi|922069866-Gemmatimonas_phototrophica
gi|1015605676-Gemmatimonas_phototrophica
gi|575460159-Gemmatirosa_kalamazoonesis
gi|575460523-Gemmatirosa_kalamazoonesis
gi|255071343-Micromonas_commoda
gi|916717889-Solirubrobacter_sp__URHD0082
gi|499276286|emb|CCM65385_1|-Candidatus_Microthrix_parvicella_RN1
gi|1012314771-Betaproteobacteria_bacterium_UKL13-2
gi|1012313247-Betaproteobacteria_bacterium_UKL13-2
gi|489706765-Methylosinus_sp__3S-1
gi|211926988|dbj|BAG82722_1|-uncultured_bacterium
gi|1040061800-Methylosinus_sp__3S-1
gi|489706768-Methylosinus_sp__3S-1
gi|1041167855|emb|CUS03890_2|-Ardenticatena
gi|1028145618-Burkholderia_ubonensis
gi|978682626-Burkholderia_ubonensis
gi|978704517-Burkholderia_ubonensis
gi|292815528-Achromobacter_piechaudii_ATCC_43553
gi|918364744-Rhodococcus_erythropolis
gi|500198409-Herminiimonas_arsenicoxydans
GSMAST4A1_ASY1_ANO1_6831_1
gi|763374578-Chloroflexus_sp__Y-396-1
gi|966420992-Legionella_cincinnatiensis
gi|902952280|dbj|GAP12732_1|-Longilinea_arvoryzae
gi|931595976-Kibdelosporangium_phytohabitans
gi|501684361-Chloroflexus_aggregans_DSM_9485
gi|501684359-Chloroflexus_aggregans_DSM_9485
gi|500796273-Roseiflexus_castenholzii_DSM_13941
gi|1046355167-uncultured_Candidatus_Thalassoarchaea_euryarchaeote
gi|1055964608-Actinoplanes_sp__TFC3
gi|926770712-Monoraphidium_neglectum
gi|966476833-Legionella_quinlivanii
gi|966506002-Legionella_birminghamensis
gi|505256360-Ilumatobacter_coccineus_YM16-304
gi|918755323-Actinobacteria_bacterium_IMCC26256
gi|494031767-Plesiocystis_pacifica_SIR-1
gi|747213892-Enhygromyxa_salina
gi|494030536-Plesiocystis_pacifica_SIR-1
gi|502588877-Haliangium_ochraceum_DSM_14365
gi|919215225-Myxococcus_fulvus
gi|262079615-Haliangium_ochraceum_DSM_14365
gi|504213629-Corallococcus_coralloides_DSM_2259
gi|488691282-Stigmatella_aurantiaca_DW4/3-1
gi|488691269-Stigmatella_aurantiaca_DW4/3-1
gi|759604252-Corallococcus_coralloides
gi|380731836-Corallococcus_coralloides_DSM_2259
gi|530766216-Bacteriovorax_sp__Seq25_V
gi|747218321-Enhygromyxa_salina
gi|913612585-Labilithrix_luteola
gi|502588615-Haliangium_ochraceum_DSM_14365
gi|494031768-Plesiocystis_pacifica_SIR-1
gi|747213891-Enhygromyxa_salina
gi|1028408046-bacterium_SCGC_AG-212-C10
gi|517520441-Algicola_sagamiensis
gi|663515488-uncultured_marine_group_II/III_euryarchaeote_KM3_18_A05
gi|663524748-uncultured_marine_group_II/III_euryarchaeote_KM3_69_A05
gi|913612426-Labilithrix_luteola
gi|913616197-Labilithrix_luteola
gi|918755553-Actinobacteria_bacterium_IMCC26256
gi|1048815963|dbj|BAV34704_1|-Sulfuricaulis_limicola
gi|255090038-Micromonas_commoda
gi|1012139527-Limnobacter_sp__CACIAM_66H1
gi|829574334-Actinobacteria_bacterium_IMCC26256
gi|818861620-Candidatus_Wolfebacteria_bacterium_GW2011_GWE1_48_7
gi|818820920-Candidatus_Wolfebacteria_bacterium_GW2011_GWE2_47_12
gi|818851534-Candidatus_Wolfebacteria_bacterium_GW2011_GWA2_47_9b
gi|818838213-Candidatus_Wolfebacteria_bacterium_GW2011_GWA1_47_6
gi|818815360-Candidatus_Wolfebacteria_bacterium_GW2011_GWC1_47_103
gi|818802226-Candidatus_Wolfebacteria_bacterium_GW2011_GWB2_46_69
gi|837897957-Micromonospora_sp__RV43
gi|817506957-Micromonospora_sp__HK10
gi|1052303219|emb|SCF46654_1|-Micromonospora_mirobrigensis
gi|503498590-Verrucosispora_maris
gi|522004734-Actinoplanes_globisporus
gi|359836310-Actinoplanes_sp__SE50/110
gi|117648631-Acidothermus_cellulolyticus_11B
gi|947738088-Aeromicrobium_sp__Root236
gi|948229110-Nocardioides_sp__Soil777
gi|948270725-Nocardioides_sp__Soil805
gi|159488616-Chlamydomonas_reinhardtii
gi|931405129-Gammaproteobacteria_bacterium_SG8_15
gi|918754896-Actinobacteria_bacterium_IMCC26256
gi|359824642-Clostridium_clariflavum_DSM_19732
gi|507795642-Paenibacillus_barengoltzii_G22
gi|919181528-Paenibacillus_algorifonticola
gi|918573708-Cystobacter_violaceus
gi|568005507-Phytophthora_parasitica
gi|675177737-Phytophthora_parasitica_INRA-310
gi|953489646|emb|CEG47012_1|-Plasmopara_halstedii
gi|695398286-Phytophthora_sojae
gi|325187776|emb|CCA22321_1|-Albugo_laibachii_Nc14
gi|698780539-Aphanomyces_astaci
gi|698780537-Aphanomyces_astaci
gi|673024520-Aphanomyces_invadans
gi|499718534-Hahella_chejuensis_KCTC_2396
gi|667481793-Thraustotheca_clavata
gi|813110405-Saprolegnia_parasitica_CBS_223_65
gi|669159922-Saprolegnia_diclina_VS20
gi|551350787-Arenimonas_oryziterrae_DSM_21050_=_YC6267
gi|723286022-Candidatus_Thiomargarita_nelsonii
gi|599568154-Chondromyces_apiculatus_DSM_436
gi|763383637-Chondromyces_apiculatus
gi|551634582-Guillardia_theta_CCMP2712
gi|406911964-uncultured_bacterium
gi|686207073|dbj|BAP58206_1|-Thioploca_ingrica
gi|470447147-Acanthamoeba_castellanii_str__Neff
gi|670620939-Hyalangium_minutum
gi|913367903-Hyalangium_minutum
gi|386429469-Beggiatoa_alba_B18LD
gi|1005176757-Sorangium_cellulosum
gi|946530461-Candidatus_Peribacter_riflensis
gi|299115321|emb|CBN74137_1|-Ectocarpus_siliculosus
gi|931499879-Gemmatimonas_sp__SM23_52
gi|425862017-Bdellovibrio_bacteriovorus_str__Tiberius
gi|501193800-Sorangium_cellulosum_So_ce56
gi|551349131-Arenimonas_oryziterrae_DSM_21050_=_YC6267
gi|551649742-Guillardia_theta_CCMP2712
gi|551634584-Guillardia_theta_CCMP2712
gi|914796169-Vulgatibacter_incomptus
gi|913610133-Vulgatibacter_incomptus
gi|517803416-Rudaea_cellulosilytica
gi|644389783-Bdellovibrio_bacteriovorus_W
gi|575425285-Candidatus_Entotheonella_sp__TSY2
gi|813141426-Saprolegnia_parasitica_CBS_223_65
gi|686205562|dbj|BAP56695_1|-Thioploca_ingrica
0.1
Archaea
Bacteria
Eukaryota
SAG

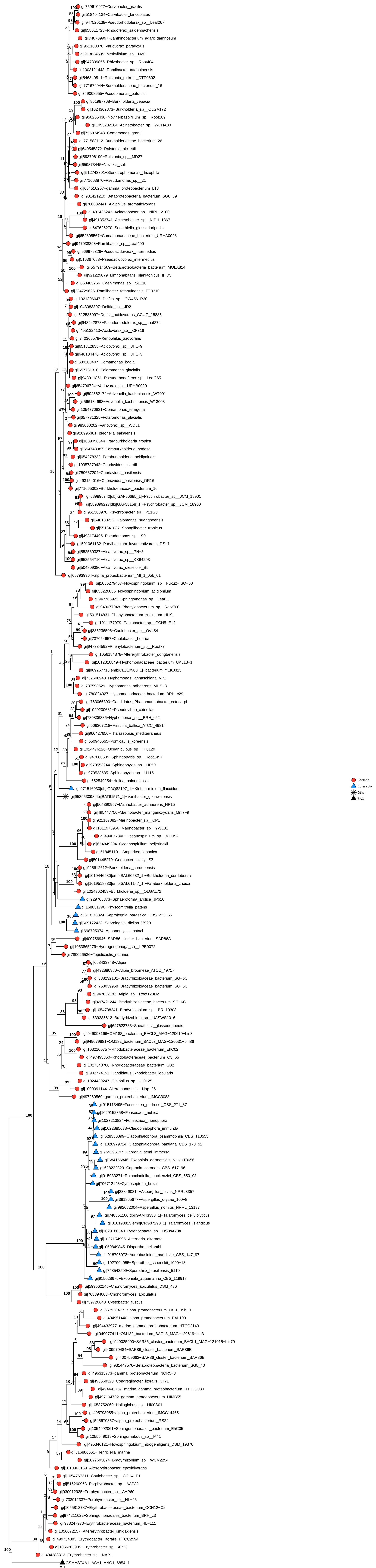
Bacteria
Eukaryota
Other
SAG
GSMAST4A1_ASY1_ANO1_6854_1
0.1

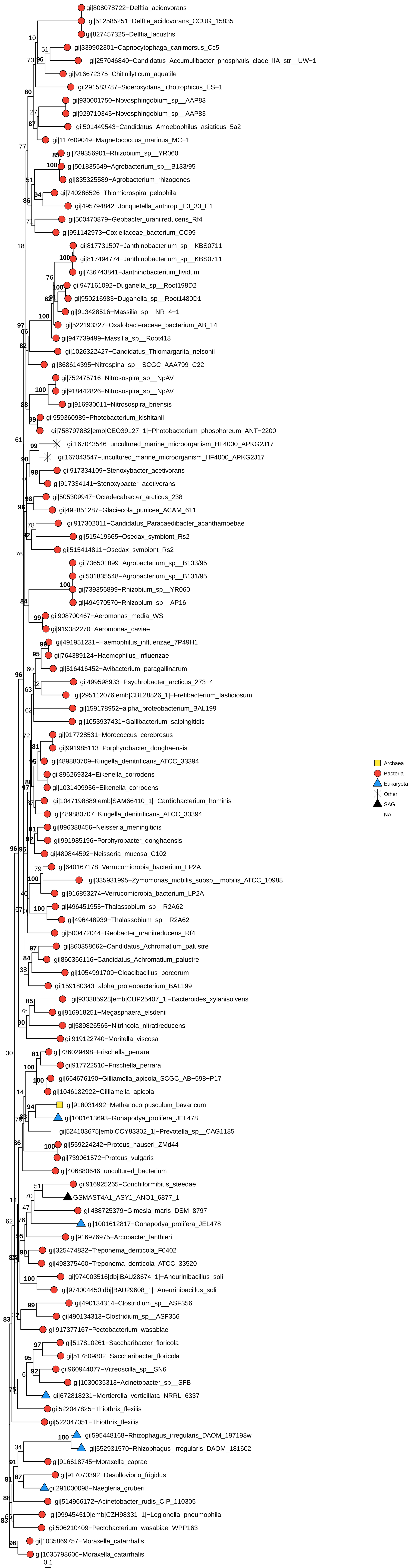
gi|808078722−Delftia_acidovorans
gi|512585251−Delftia_acidovorans_CCUG_15835
gi|827457325−Delftia_lacustris
gi|339902301−Capnocytophaga_canimorsus_Cc5
gi|257046840−Candidatus_Accumulibacter_phosphatis_clade_IIA_str__UW−1
gi|916672375−Chitinilyticum_aquatile
gi|291583787−Sideroxydans_lithotrophicus_ES−1
gi|930001750−Novosphingobium_sp__AAP83
gi|929710345−Novosphingobium_sp__AAP83
gi|501449543−Candidatus_Amoebophilus_asiaticus_5a2
gi|117609049−Magnetococcus_marinus_MC−1
gi|739356901−Rhizobium_sp__YR060
gi|501835549−Agrobacterium_sp__B133/95
gi|835325589−Agrobacterium_rhizogenes
gi|740286526−Thiomicrospira_pelophila
gi|495794842−Jonquetella_anthropi_E3_33_E1
gi|500470879−Geobacter_uraniireducens_Rf4
gi|951142973−Coxiellaceae_bacterium_CC99
gi|817731507−Janthinobacterium_sp__KBS0711
gi|817494774−Janthinobacterium_sp__KBS0711
gi|736743841−Janthinobacterium_lividum
gi|947161092−Duganella_sp__Root198D2
gi|950216983−Duganella_sp__Root1480D1
gi|913428516−Massilia_sp__NR_4−1
gi|522193327−Oxalobacteraceae_bacterium_AB_14
gi|947739499−Massilia_sp__Root418
gi|1026322427−Candidatus_Thiomargarita_nelsonii
gi|868614395−Nitrospina_sp__SCGC_AAA799_C22
gi|752475716−Nitrosospira_sp__NpAV
gi|918442826−Nitrosospira_sp__NpAV
gi|916930011−Nitrosospira_briensis
gi|959360989−Photobacterium_kishitanii
gi|758797882|emb|CEO39127_1|−Photobacterium_phosphoreum_ANT−2200
gi|167043546−uncultured_marine_microorganism_HF4000_APKG2J17
gi|167043547−uncultured_marine_microorganism_HF4000_APKG2J17
gi|917334109−Stenoxybacter_acetivorans
gi|917334141−Stenoxybacter_acetivorans
gi|505309947−Octadecabacter_arcticus_238
gi|492851287−Glaciecola_punicea_ACAM_611
gi|917302011−Candidatus_Paracaedibacter_acanthamoebae
gi|515419665−Osedax_symbiont_Rs2
gi|515414811−Osedax_symbiont_Rs2
gi|736501899−Agrobacterium_sp__B133/95
gi|501835548−Agrobacterium_sp__B131/95
gi|739356899−Rhizobium_sp__YR060
gi|494970570−Rhizobium_sp__AP16
gi|908700467−Aeromonas_media_WS
gi|919382270−Aeromonas_caviae
gi|491951231−Haemophilus_influenzae_7P49H1
gi|764389124−Haemophilus_influenzae
gi|516416452−Avibacterium_paragallinarum
gi|499598933−Psychrobacter_arcticus_273−4
gi|295112076|emb|CBL28826_1|−Fretibacterium_fastidiosum
gi|159178952−alpha_proteobacterium_BAL199
gi|1053937431−Gallibacterium_salpingitidis
gi|917728531−Morococcus_cerebrosus
gi|991985113−Porphyrobacter_donghaensis
gi|489880709−Kingella_denitrificans_ATCC_33394
gi|896269324−Eikenella_corrodens
gi|1031409956−Eikenella_corrodens
gi|1047198889|emb|SAM66410_1|−Cardiobacterium_hominis
gi|489880707−Kingella_denitrificans_ATCC_33394
gi|896388456−Neisseria_meningitidis
gi|991985196−Porphyrobacter_donghaensis
gi|489844592−Neisseria_mucosa_C102
gi|640167178−Verrucomicrobia_bacterium_LP2A
gi|335931995−Zymomonas_mobilis_subsp__mobilis_ATCC_10988
gi|916853274−Verrucomicrobia_bacterium_LP2A
gi|496451955−Thalassobium_sp__R2A62
gi|496448939−Thalassobium_sp__R2A62
gi|500472044−Geobacter_uraniireducens_Rf4
gi|860358662−Candidatus_Achromatium_palustre
gi|860366116−Candidatus_Achromatium_palustre
gi|1054991709−Cloacibacillus_porcorum
gi|159180343−alpha_proteobacterium_BAL199
gi|933385928|emb|CUP25407_1|−Bacteroides_xylanisolvens
gi|916918251−Megasphaera_elsdenii
gi|589826565−Nitrincola_nitratireducens
gi|919122740−Moritella_viscosa
gi|736029498−Frischella_perrara
gi|917722510−Frischella_perrara
gi|664676190−Gilliamella_apicola_SCGC_AB−598−P17
gi|1046182922−Gilliamella_apicola
gi|918031492−Methanocorpusculum_bavaricum
gi|1001613693−Gonapodya_prolifera_JEL478
gi|524103675|emb|CCY83302_1|−Prevotella_sp__CAG1185
gi|559224242−Proteus_hauseri_ZMd44
gi|739061572−Proteus_vulgaris
gi|406880646−uncultured_bacterium
gi|916925265−Conchiformibius_steedae
GSMAST4A1_ASY1_ANO1_6877_1
gi|488725379−Gimesia_maris_DSM_8797
gi|1001612817−Gonapodya_prolifera_JEL478
gi|916976975−Arcobacter_lanthieri
gi|325474832−Treponema_denticola_F0402
gi|498375460−Treponema_denticola_ATCC_33520
gi|974003516|dbj|BAU28674_1|−Aneurinibacillus_soli
gi|974004450|dbj|BAU29608_1|−Aneurinibacillus_soli
gi|490134314−Clostridium_sp__ASF356
gi|490134313−Clostridium_sp__ASF356
gi|917377167−Pectobacterium_wasabiae
gi|517810261−Saccharibacter_floricola
gi|517809802−Saccharibacter_floricola
gi|960944077−Vitreoscilla_sp__SN6
gi|1030035313−Acinetobacter_sp__SFB
gi|672818231−Mortierella_verticillata_NRRL_6337
gi|522047825−Thiothrix_flexilis
gi|522047051−Thiothrix_flexilis
gi|595448168−Rhizophagus_irregularis_DAOM_197198w
gi|552931570−Rhizophagus_irregularis_DAOM_181602
gi|916618745−Moraxella_caprae
gi|917070392−Desulfovibrio_frigidus
gi|291000098−Naegleria_gruberi
gi|514966172−Acinetobacter_rudis_CIP_110305
gi|999454510|emb|CZH98331_1|−Legionella_pneumophila
gi|506210409−Pectobacterium_wasabiae_WPP163
gi|1035869757−Moraxella_catarrhalis
gi|1035798606−Moraxella_catarrhalis
0.1
Archaea
Bacteria
Eukaryota
Other
SAG
NA

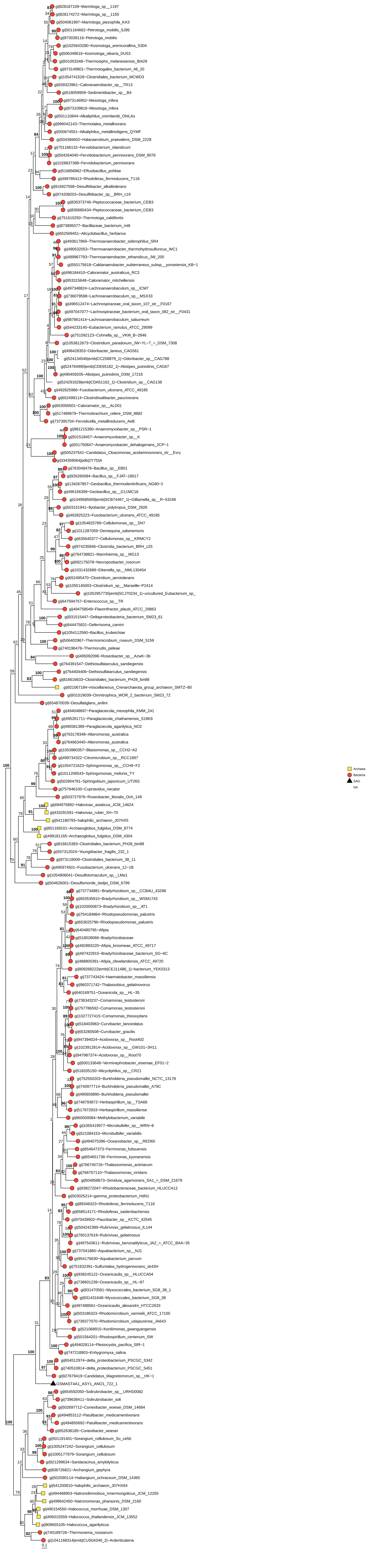
Archaea
Bacteria
SAG
NA

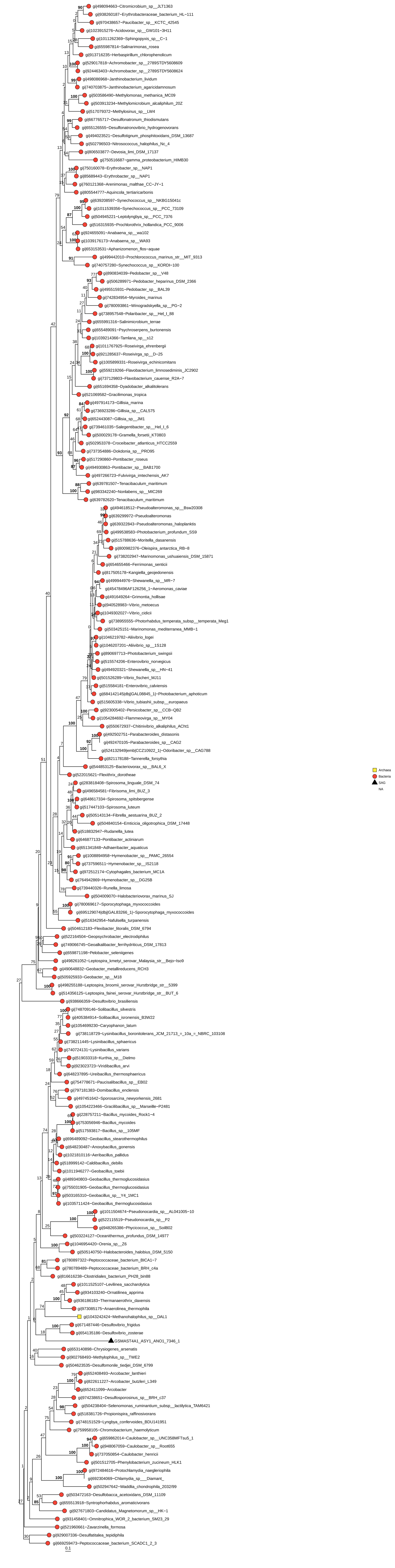
Archaea
Bacteria
SAG
NA
0.1

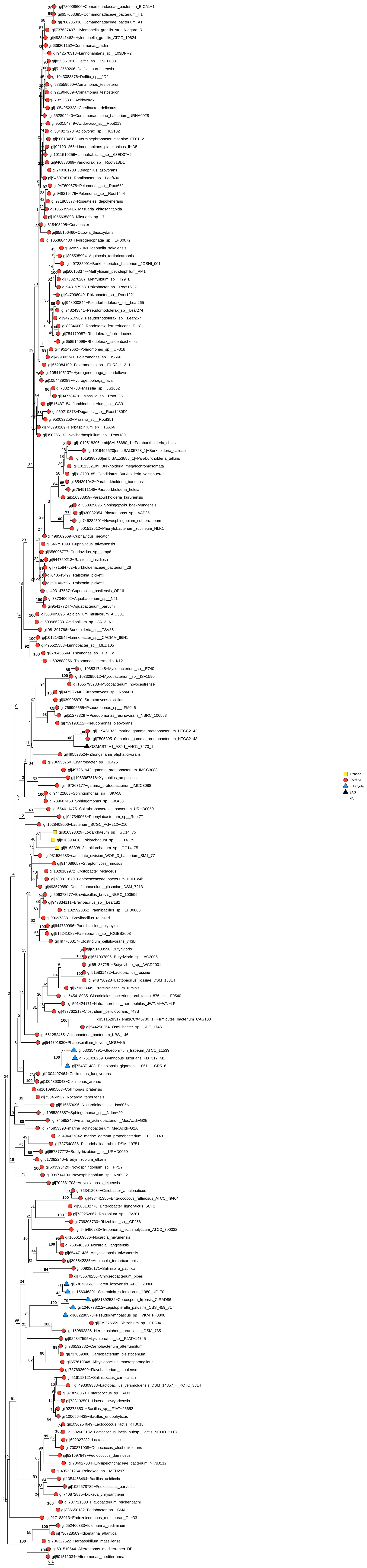

Archaea
Bacteria
Eukaryota
SAG
NA

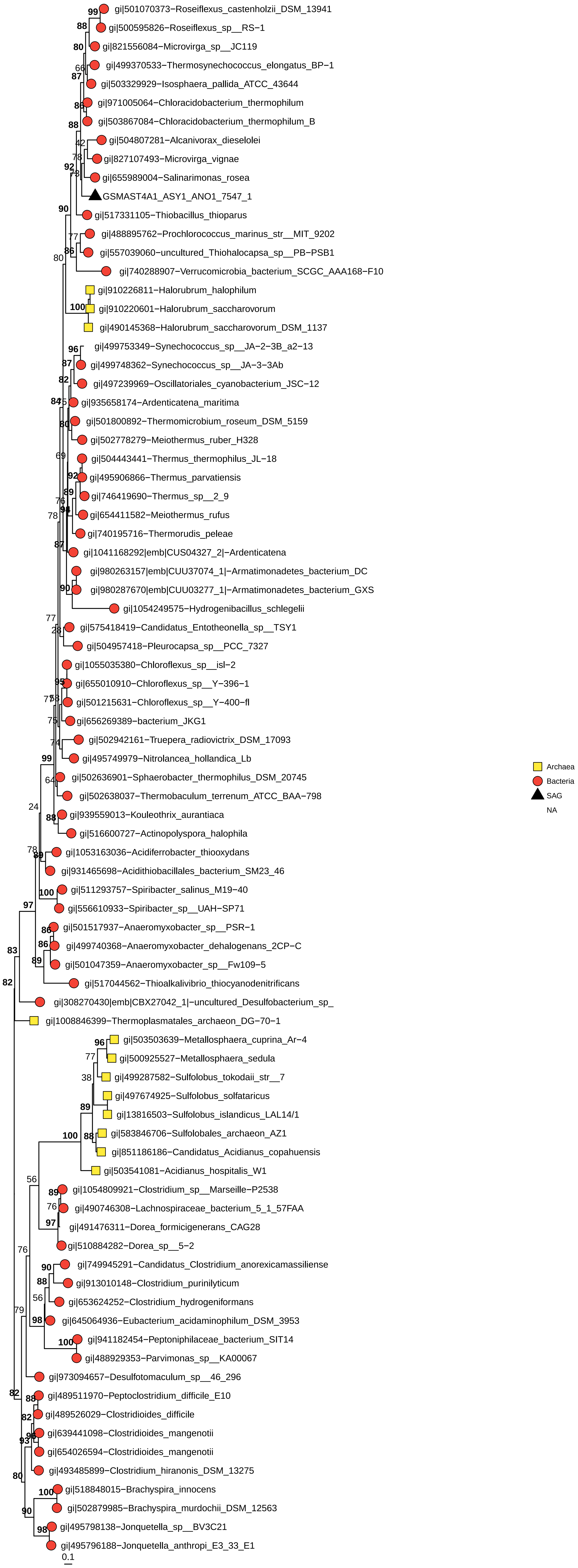
gi|501070373−Roseiflexus_castenholzii_DSM_13941
gi|500595826−Roseiflexus_sp__RS−1
gi|821556084−Microvirga_sp__JC119
gi|499370533−Thermosynechococcus_elongatus_BP−1
gi|503329929−Isosphaera_pallida_ATCC_43644
gi|971005064−Chloracidobacterium_thermophilum
gi|503867084−Chloracidobacterium_thermophilum_B
gi|504807281−Alcanivorax_dieselolei
gi|827107493−Microvirga_vignae
gi|655989004−Salinarimonas_rosea
GSMAST4A1_ASY1_ANO1_7547_1
gi|517331105−Thiobacillus_thioparus
gi|488895762−Prochlorococcus_marinus_str__MIT_9202
gi|557039060−uncultured_Thiohalocapsa_sp__PB−PSB1
gi|740288907−Verrucomicrobia_bacterium_SCGC_AAA168−F10
gi|910226811−Halorubrum_halophilum
gi|910220601−Halorubrum_saccharovorum
gi|490145368−Halorubrum_saccharovorum_DSM_1137
gi|499753349−Synechococcus_sp__JA−2−3B_a2−13
gi|499748362−Synechococcus_sp__JA−3−3Ab
gi|497239969−Oscillatoriales_cyanobacterium_JSC−12
gi|935658174−Ardenticatena_maritima
gi|501800892−Thermomicrobium_roseum_DSM_5159
gi|502778279−Meiothermus_ruber_H328
gi|504443441−Thermus_thermophilus_JL−18
gi|495906866−Thermus_parvatiensis
gi|746419690−Thermus_sp__2_9
gi|654411582−Meiothermus_rufus
gi|740195716−Thermorudis_peleae
gi|1041168292|emb|CUS04327_2|−Ardenticatena
gi|980263157|emb|CUU37074_1|−Armatimonadetes_bacterium_DC
gi|980287670|emb|CUU03277_1|−Armatimonadetes_bacterium_GXS
gi|1054249575−Hydrogenibacillus_schlegelii
gi|575418419−Candidatus_Entotheonella_sp__TSY1
gi|504957418−Pleurocapsa_sp__PCC_7327
gi|1055035380−Chloroflexus_sp__isl−2
gi|655010910−Chloroflexus_sp__Y−396−1
gi|501215631−Chloroflexus_sp__Y−400−fl
gi|656269389−bacterium_JKG1
gi|502942161−Truepera_radiovictrix_DSM_17093
gi|495749979−Nitrolancea_hollandica_Lb
gi|502636901−Sphaerobacter_thermophilus_DSM_20745
gi|502638037−Thermobaculum_terrenum_ATCC_BAA−798
gi|939559013−Kouleothrix_aurantiaca
gi|516600727−Actinopolyspora_halophila
gi|1053163036−Acidiferrobacter_thiooxydans
gi|931465698−Acidithiobacillales_bacterium_SM23_46
gi|511293757−Spiribacter_salinus_M19−40
gi|556610933−Spiribacter_sp__UAH−SP71
gi|501517937−Anaeromyxobacter_sp__PSR−1
gi|499740368−Anaeromyxobacter_dehalogenans_2CP−C
gi|501047359−Anaeromyxobacter_sp__Fw109−5
gi|517044562−Thioalkalivibrio_thiocyanodenitrificans
gi|308270430|emb|CBX27042_1|−uncultured_Desulfobacterium_sp_
gi|1008846399−Thermoplasmatales_archaeon_DG−70−1
gi|503503639−Metallosphaera_cuprina_Ar−4
gi|500925527−Metallosphaera_sedula
gi|499287582−Sulfolobus_tokodaii_str__7
gi|497674925−Sulfolobus_solfataricus
gi|13816503−Sulfolobus_islandicus_LAL14/1
gi|583846706−Sulfolobales_archaeon_AZ1
gi|851186186−Candidatus_Acidianus_copahuensis
gi|503541081−Acidianus_hospitalis_W1
gi|1054809921−Clostridium_sp__Marseille−P2538
gi|490746308−Lachnospiraceae_bacterium_5_1_57FAA
gi|491476311−Dorea_formicigenerans_CAG28
gi|510884282−Dorea_sp__5−2
gi|749945291−Candidatus_Clostridium_anorexicamassiliense
gi|913010148−Clostridium_purinilyticum
gi|653624252−Clostridium_hydrogeniformans
gi|645064936−Eubacterium_acidaminophilum_DSM_3953
gi|941182454−Peptoniphilaceae_bacterium_SIT14
gi|488929353−Parvimonas_sp__KA00067
gi|973094657−Desulfotomaculum_sp__46_296
gi|489511970−Peptoclostridium_difficile_E10
gi|489526029−Clostridioides_difficile
gi|639441098−Clostridioides_mangenotii
gi|654026594−Clostridioides_mangenotii
gi|493485899−Clostridium_hiranonis_DSM_13275
gi|518848015−Brachyspira_innocens
gi|502879985−Brachyspira_murdochii_DSM_12563
gi|495798138−Jonquetella_sp__BV3C21
gi|495796188−Jonquetella_anthropi_E3_33_E1
0.1
Archaea
Bacteria
SAG
NA

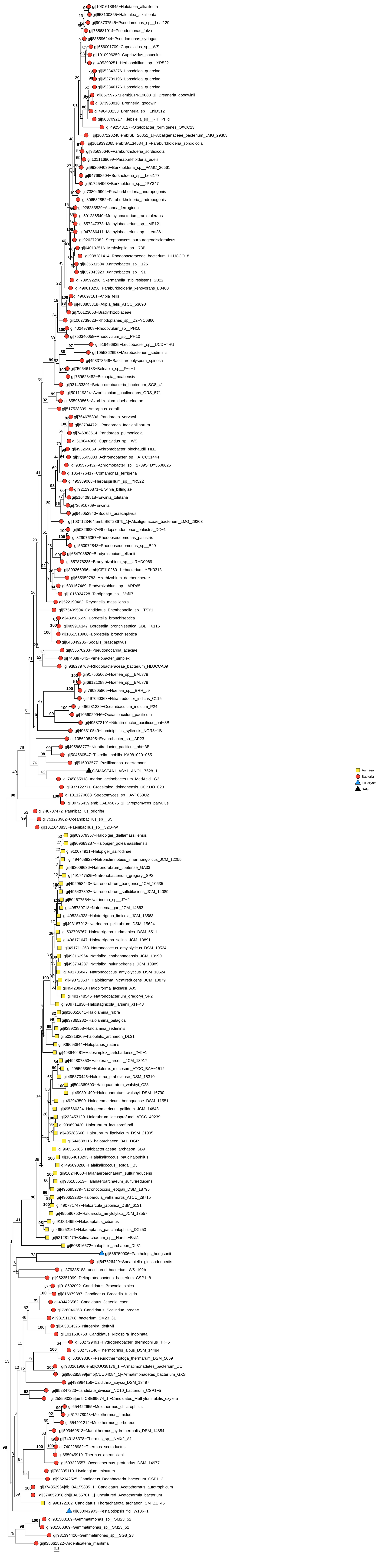
gi|1031618845~Halotalea_alkalilenta
gi|653100365~Halotalea_alkalilenta
gi|908737545~Pseudomonas_sp__Leaf129
gi|755681914~Pseudomonas_fulva
gi|835596244~Pseudomonas_syringae
gi|656001709~Cupriavidus_sp__WS
gi|1010996259~Cupriavidus_pauculus
gi|495390251~Herbaspirillum_sp__YR522
gi|652343376~Lonsdalea_quercina
gi|652739196~Lonsdalea_quercina
gi|652346176~Lonsdalea_quercina
gi|857597571|emb|CPR19083_1|~Brenneria_goodwinii
gi|873963818~Brenneria_goodwinii
gi|496403233~Brenneria_sp__EniD312
gi|908709217~Klebsiella_sp__RIT~PI~d
gi|492543117~Oxalobacter_formigenes_OXCC13
gi|1037120248|emb|SBT26851_1|~Alcaligenaceae_bacterium_LMG_29303
gi|1019392065|emb|SAL34584_1|~Paraburkholderia_sordidicola
gi|985635646~Paraburkholderia_sordidicola
gi|1011168099~Paraburkholderia_udeis
gi|992094089~Burkholderia_sp__PAMC_26561
gi|947698504~Burkholderia_sp__Leaf177
gi|517254968~Burkholderia_sp__JPY347
gi|738049904~Paraburkholderia_andropogonis
gi|806532852~Paraburkholderia_andropogonis
gi|926283829~Asanoa_ferruginea
gi|501286540~Methylobacterium_radiotolerans
gi|657247373~Methylobacterium_sp__ME121
gi|947866411~Methylobacterium_sp__Leaf361
gi|926272082~Streptomyces_purpurogeneiscleroticus
gi|640192516~Methylopila_sp__73B
gi|938281414~Rhodobacteraceae_bacterium_HLUCCO18
gi|653631504~Xanthobacter_sp__126
gi|657843923~Xanthobacter_sp__91
gi|739592290~Skermanella_stibiiresistens_SB22
gi|499810258~Paraburkholderia_xenovorans_LB400
gi|496697181~Afipia_felis
gi|488805318~Afipia_felis_ATCC_53690
gi|750123053~Bradyrhizobiaceae
gi|1002739623~Rhodoplanes_sp__Z2~YC6860
gi|402497908~Rhodovulum_sp__PH10
gi|750340058~Rhodovulum_sp__PH10
gi|516496835~Leucobacter_sp__UCD~THU
gi|1055362693~Microbacterium_sediminis
gi|498378549~Saccharopolyspora_spinosa
gi|759646183~Belnapia_sp__F~4~1
gi|759623482~Belnapia_moabensis
gi|931433391~Betaproteobacteria_bacterium_SG8_41
gi|501119324~Azorhizobium_caulinodans_ORS_571
gi|655963866~Azorhizobium_doebereinerae
gi|517528809~Amorphus_coralli
gi|764675806~Pandoraea_vervacti
gi|837944721~Pandoraea_faecigallinarum
gi|746363514~Pandoraea_pulmonicola
gi|519044986~Cupriavidus_sp__WS
gi|493269059~Achromobacter_piechaudii_HLE
gi|935505083~Achromobacter_sp__ATCC31444
gi|935575432~Achromobacter_sp__2789STDY5608625
gi|1054776417~Comamonas_terrigena
gi|495389068~Herbaspirillum_sp__YR522
gi|921196871~Erwinia_billingiae
gi|516409518~Erwinia_toletana
gi|736916769~Erwinia
gi|645052940~Sodalis_praecaptivus
gi|1037123464|emb|SBT23679_1|~Alcaligenaceae_bacterium_LMG_29303
gi|503268207~Rhodopseudomonas_palustris_DX~1
gi|829076357~Rhodopseudomonas_palustris
gi|550972843~Rhodopseudomonas_sp__B29
gi|654703620~Bradyrhizobium_elkanii
gi|657878235~Bradyrhizobium_sp__URHD0069
gi|809266996|emb|CEJ10260_1|~bacterium_YEK0313
gi|655959783~Azorhizobium_doebereinerae
gi|639167469~Bradyrhizobium_sp__ARR65
gi|1016924728~Tardiphaga_sp__Vaf07
gi|522190462~Reyranella_massiliensis
gi|575409504~Candidatus_Entotheonella_sp__TSY1
gi|489905599~Bordetella_bronchiseptica
gi|489916147~Bordetella_bronchiseptica_SBL~F6116
gi|1051510988~Bordetella_bronchiseptica
gi|645049205~Sodalis_praecaptivus
gi|655570203~Pseudonocardia_acaciae
gi|740897045~Pimelobacter_simplex
gi|938279768~Rhodobacteraceae_bacterium_HLUCCA09
gi|917565662~Hoeflea_sp__BAL378
gi|691212880~Hoeflea_sp__BAL378
gi|780805809~Hoeflea_sp__BRH_c9
gi|497060363~Nitratireductor_indicus_C115
gi|496231239~Oceanibaculum_indicum_P24
gi|1056029946~Oceanibaculum_pacificum
gi|495872101~Nitratireductor_pacificus_pht~3B
gi|496310549~Luminiphilus_syltensis_NOR5~1B
gi|1056208495~Erythrobacter_sp__AP23
gi|495868777~Nitratireductor_pacificus_pht~3B
gi|504560547~Tistrella_mobilis_KA081020~065
gi|516093577~Pusillimonas_noertemannii
GSMAST4A1_ASY1_ANO1_7628_1
gi|745855918~marine_actinobacterium_MedAcid~G3
gi|937122771~Croceitalea_dokdonensis_DOKDO_023
gi|1011270668~Streptomyces_sp__AVP053U2
gi|39725439|emb|CAE45675_1|~Streptomyces_parvulus
gi|740787472~Paenibacillus_odorifer
gi|751273962~Oceanobacillus_sp__S5
gi|1011643835~Paenibacillus_sp__32O~W
gi|909679357~Halopiger_djelfamassiliensis
gi|909683287~Halopiger_goleamassiliensis
gi|910074911~Halopiger_salifodinae
gi|494468922~Natronolimnobius_innermongolicus_JCM_12255
gi|493009636~Natronorubrum_tibetense_GA33
gi|491747525~Natronobacterium_gregoryi_SP2
gi|492958443~Natronorubrum_bangense_JCM_10635
gi|495437892~Natronorubrum_sulfidifaciens_JCM_14089
gi|504677554~Natrinema_sp__J7~2
gi|495730718~Natrinema_gari_JCM_14663
gi|495284328~Haloterrigena_limicola_JCM_13563
gi|493187912~Natrinema_pellirubrum_DSM_15624
gi|502706767~Haloterrigena_turkmenica_DSM_5511
gi|496171647~Haloterrigena_salina_JCM_13891
gi|491711268~Natronococcus_amylolyticus_DSM_10524
gi|493162964~Natrialba_chahannaoensis_JCM_10990
gi|493704237~Natrialba_hulunbeirensis_JCM_10989
gi|491705847~Natronococcus_amylolyticus_DSM_10524
gi|493723537~Halobiforma_nitratireducens_JCM_10879
gi|494238463~Halobiforma_lacisalsi_AJ5
gi|491748546~Natronobacterium_gregoryi_SP2
gi|909711830~Halostagnicola_larsenii_XH~48
gi|910051641~Halolamina_rubra
gi|937365282~Halolamina_pelagica
gi|928923858~Halolamina_sediminis
gi|503818209~halophilic_archaeon_DL31
gi|909693844~Haloplanus_natans
gi|493940481~Halosimplex_carlsbadense_2~9~1
gi|494807853~Haloferax_larsenii_JCM_13917
gi|495595869~Haloferax_mucosum_ATCC_BAA~1512
gi|495370445~Haloferax_prahovense_DSM_18310
gi|504369600~Haloquadratum_walsbyi_C23
gi|499891499~Haloquadratum_walsbyi_DSM_16790
gi|492943509~Halogeometricum_borinquense_DSM_11551
gi|495660324~Halogeometricum_pallidum_JCM_14848
gi|222453129~Halorubrum_lacusprofundi_ATCC_49239
gi|909690420~Halorubrum_lacusprofundi
gi|495283660~Halorubrum_lipolyticum_DSM_21995
gi|544638116~haloarchaeon_3A1_DGR
gi|968555386~Halobacteriaceae_archaeon_SB9
gi|1054613293~Halalkalicoccus_paucihalophilus
gi|495690280~Halalkalicoccus_jeotgali_B3
gi|910244068~Halanaeroarchaeum_sulfurireducens
gi|936185513~Halanaeroarchaeum_sulfurireducens
gi|495695279~Natronococcus_jeotgali_DSM_18795
gi|490653280~Haloarcula_vallismortis_ATCC_29715
gi|490731747~Haloarcula_japonica_DSM_6131
gi|495586750~Haloarcula_amylolytica_JCM_13557
gi|910014958~Haladaptatus_cibarius
gi|495252161~Haladaptatus_paucihalophilus_DX253
gi|521281479~Salinarchaeum_sp__Harcht~Bsk1
gi|503816672~halophilic_archaeon_DL31
gi|556750006~Pantholops_hodgsonii
gi|647626429~Sneathiella_glossodoripedis
gi|379335188~uncultured_bacterium_W5~102b
gi|952351099~Deltaproteobacteria_bacterium_CSP1~8
gi|918692092~Candidatus_Brocadia_sinica
gi|816979887~Candidatus_Brocadia_fulgida
gi|494426562~Candidatus_Jettenia_caeni
gi|726046368~Candidatus_Scalindua_brodae
gi|931511708~bacterium_SM23_31
gi|503014326~Nitrospira_defluvii
gi|1011636768~Candidatus_Nitrospira_inopinata
gi|502729491~Hydrogenobacter_thermophilus_TK~6
gi|502757146~Thermocrinis_albus_DSM_14484
gi|503698367~Pseudothermotoga_thermarum_DSM_5069
gi|980261966|emb|CUU38176_1|~Armatimonadetes_bacterium_DC
gi|980285899|emb|CUU04084_1|~Armatimonadetes_bacterium_GXS
gi|493984156~Caldithrix_abyssi_DSM_13497
gi|952347223~candidate_division_NC10_bacterium_CSP1~5
gi|258593335|emb|CBE69674_1|~Candidatus_Methylomirabilis_oxyfera
gi|654422655~Meiothermus_chliarophilus
gi|517278043~Meiothermus_timidus
gi|654401212~Meiothermus_cerbereus
gi|503469813~Marinithermus_hydrothermalis_DSM_14884
gi|740186378~Thermus_sp__NMX2_A1
gi|740288982~Thermus_scotoductus
gi|655045919~Thermus_antranikianii
gi|503223557~Oceanithermus_profundus_DSM_14977
gi|763335110~Hyalangium_minutum
gi|952342525~Candidatus_Dadabacteria_bacterium_CSP1~2
gi|374852964|dbj|BAL55885_1|~Candidatus_Acetothermus_autotrophicum
gi|374852858|dbj|BAL55781_1|~uncultured_Acetothermia_bacterium
gi|998172202~Candidatus_Thorarchaeota_archaeon_SMTZ1~45
gi|630042903~Pestalotiopsis_fici_W106~1
gi|931503189~Gemmatimonas_sp__SM23_52
gi|931500369~Gemmatimonas_sp__SM23_52
gi|931394426~Gemmatimonas_sp__SG8_23
gi|935661522~Ardenticatena_maritima
0.1
Archaea
Bacteria
Eukaryota
SAG

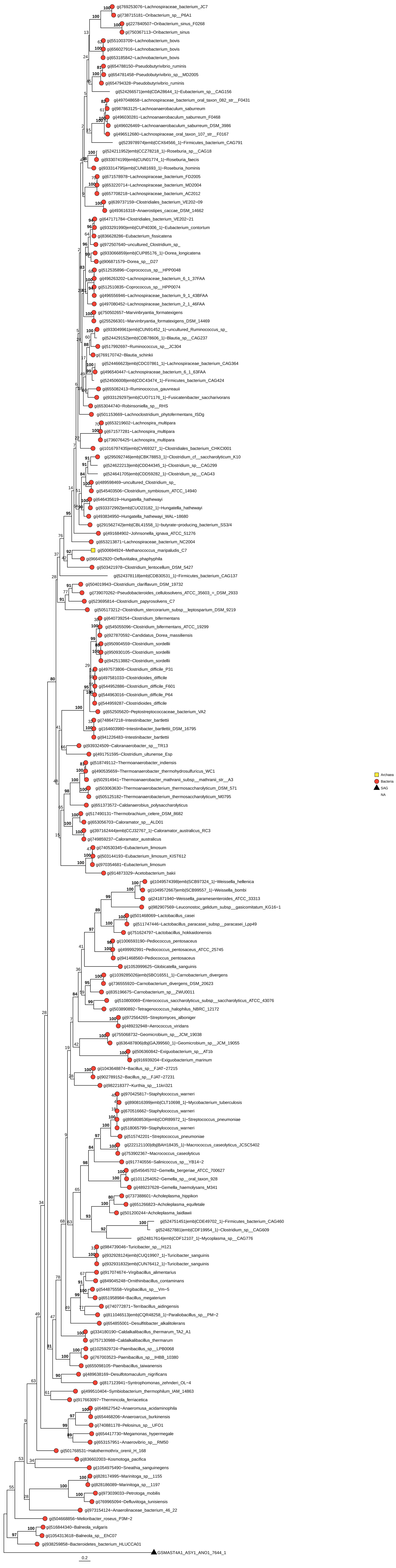

gi|769253076~Lachnospiraceae_bacterium_JC7
gi|738715181~Oribacterium_sp__P6A1
gi|227840507~Oribacterium_sinus_F0268
gi|750367113~Oribacterium_sinus
gi|551003709~Lachnobacterium_bovis
gi|656027916~Lachnobacterium_bovis
gi|653185842~Lachnobacterium_bovis
gi|654788150~Pseudobutyrivibrio_ruminis
gi|654781458~Pseudobutyrivibrio_sp__MD2005
gi|654794328~Pseudobutyrivibrio_ruminis
gi|524266571|emb|CDA28644_1|~Eubacterium_sp__CAG156
gi|497048658~Lachnospiraceae_bacterium_oral_taxon_082_str__F0431
gi|987863125~Lachnoanaerobaculum_saburreum
gi|496030281~Lachnoanaerobaculum_saburreum_F0468
gi|496026469~Lachnoanaerobaculum_saburreum_DSM_3986
gi|496512680~Lachnospiraceae_oral_taxon_107_str__F0167
gi|523978974|emb|CCX64566_1|~Firmicutes_bacterium_CAG791
gi|524211952|emb|CCZ78218_1|~Roseburia_sp__CAG18
gi|933074199|emb|CUN01774_1|~Roseburia_faecis
gi|933314795|emb|CUN81693_1|~Roseburia_hominis
gi|671578978~Lachnospiraceae_bacterium_FD2005
gi|653220714~Lachnospiraceae_bacterium_MD2004
gi|657708218~Lachnospiraceae_bacterium_AC2012
gi|639737159~Clostridiales_bacterium_VE202~09
gi|493616318~Anaerostipes_caccae_DSM_14662
gi|647171784~Clostridiales_bacterium_VE202~21
gi|933291990|emb|CUP40306_1|~Eubacterium_contortum
gi|936628286~Eubacterium_fissicatena
gi|972507640~uncultured_Clostridium_sp_
gi|933066859|emb|CUP85176_1|~Dorea_longicatena
gi|906871579~Dorea_sp__D27
gi|512535896~Coprococcus_sp__HPP0048
gi|496263202~Lachnospiraceae_bacterium_6_1_37FAA
gi|512510835~Coprococcus_sp__HPP0074
gi|496556946~Lachnospiraceae_bacterium_9_1_43BFAA
gi|497080452~Lachnospiraceae_bacterium_2_1_46FAA
gi|750502657~Marvinbryantia_formatexigens
gi|255266301~Marvinbryantia_formatexigens_DSM_14469
gi|933049961|emb|CUN91452_1|~uncultured_Ruminococcus_sp_
gi|524429152|emb|CDB78606_1|~Blautia_sp__CAG237
gi|517992697~Ruminococcus_sp__JC304
gi|769170742~Blautia_schinkii
gi|524466623|emb|CDC07861_1|~Lachnospiraceae_bacterium_CAG364
gi|496540447~Lachnospiraceae_bacterium_6_1_63FAA
gi|524506008|emb|CDC43474_1|~Firmicutes_bacterium_CAG424
gi|655082413~Ruminococcus_gauvreauii
gi|933129297|emb|CUO71176_1|~Fusicatenibacter_saccharivorans
gi|653044740~Robinsoniella_sp__RHS
gi|501153669~Lachnoclostridium_phytofermentans_ISDg
gi|653219602~Lachnospira_multipara
gi|671577281~Lachnospira_multipara
gi|736076425~Lachnospira_multipara
gi|1016797435|emb|CVI69327_1|~Clostridiales_bacterium_CHKCI001
gi|295092746|emb|CBK78853_1|~Clostridium_cf__saccharolyticum_K10
gi|524622213|emb|CDD44345_1|~Clostridium_sp__CAG299
gi|524641705|emb|CDD59282_1|~Clostridium_sp__CAG43
gi|489598469~uncultured_Clostridium_sp_
gi|545403506~Clostridium_symbiosum_ATCC_14940
gi|646435619~Hungatella_hathewayi
gi|933372992|emb|CUO23182_1|~Hungatella_hathewayi
gi|493834950~Hungatella_hathewayi_WAL~18680
gi|291562742|emb|CBL41558_1|~butyrate~producing_bacterium_SS3/4
gi|491684902~Johnsonella_ignava_ATCC_51276
gi|653213871~Lachnospiraceae_bacterium_NC2004
gi|500694924~Methanococcus_maripaludis_C7
gi|966452920~Defluviitalea_phaphyphila
gi|503421978~Clostridium_lentocellum_DSM_5427
gi|524378118|emb|CDB30531_1|~Firmicutes_bacterium_CAG137
gi|504019943~Clostridium_clariflavum_DSM_19732
gi|739070260~Pseudobacteroides_cellulosolvens_ATCC_35603_=_DSM_2933
gi|523695814~Clostridium_papyrosolvens_C7
gi|505173212~Clostridium_stercorarium_subsp__leptospartum_DSM_9219
gi|640739254~Clostridium_bifermentans
gi|545055096~Clostridium_bifermentans_ATCC_19299
gi|927870592~Candidatus_Dorea_massiliensis
gi|950904559~Clostridium_sordellii
gi|950930105~Clostridium_sordellii
gi|942513882~Clostridium_sordellii
gi|497573806~Clostridium_difficile_P31
gi|497581033~Clostridioides_difficile
gi|544952886~Clostridium_difficile_F601
gi|544963016~Clostridium_difficile_P64
gi|544959287~Clostridioides_difficile
gi|652505620~Peptostreptococcaceae_bacterium_VA2
gi|748647218~Intestinibacter_bartlettii
gi|164603980~Intestinibacter_bartlettii_DSM_16795
gi|941226483~Intestinibacter_bartlettii
gi|933324509~Caloranaerobacter_sp__TR13
gi|491751595~Clostridium_ultunense_Esp
gi|518749112~Thermoanaerobacter_indiensis
gi|490535659~Thermoanaerobacter_thermohydrosulfuricus_WC1
gi|502914941~Thermoanaerobacter_mathranii_subsp__mathranii_str__A3
gi|503063630~Thermoanaerobacterium_thermosaccharolyticum_DSM_571
gi|505125182~Thermoanaerobacterium_thermosaccharolyticum_M0795
gi|651373572~Caldanaerobius_polysaccharolyticus
gi|517490131~Thermobrachium_celere_DSM_8682
gi|653056703~Caloramator_sp__ALD01
gi|397162444|emb|CCJ32767_1|~Caloramator_australicus_RC3
gi|749859237~Caloramator_australicus
gi|740530345~Eubacterium_limosum
gi|503144193~Eubacterium_limosum_KIST612
gi|970354681~Eubacterium_limosum
gi|914873329~Acetobacterium_bakii
gi|1049574398|emb|SCB97324_1|~Weissella_hellenica
gi|1049572667|emb|SCB99557_1|~Weissella_bombi
gi|241871940~Weissella_paramesenteroides_ATCC_33313
gi|982907569~Leuconostoc_gelidum_subsp__gasicomitatum_KG16~1
gi|501468069~Lactobacillus_casei
gi|511747446~Lactobacillus_paracasei_subsp__paracasei_Lpp49
gi|751624797~Lactobacillus_hokkaidonensis
gi|1006593190~Pediococcus_pentosaceus
gi|499992991~Pediococcus_pentosaceus_ATCC_25745
gi|941468560~Pediococcus_pentosaceus
gi|1053999625~Globicatella_sanguinis
gi|1039285026|emb|SBO16551_1|~Carnobacterium_divergens
gi|736555920~Carnobacterium_divergens_DSM_20623
gi|835196675~Carnobacterium_sp__ZWU0011
gi|510800069~Enterococcus_saccharolyticus_subsp__saccharolyticus_ATCC_43076
gi|503890892~Tetragenococcus_halophilus_NBRC_12172
gi|972564265~Streptomyces_albongiger
gi|489232948~Aerococcus_viridans
gi|755068732~Geomicrobium_sp__JCM_19038
gi|636487806|dbj|GAJ99560_1|~Geomicrobium_sp__JCM_19055
gi|506360842~Exiguobacterium_sp__AT1b
gi|916939204~Exiguobacterium_marinum
gi|1043648874~Bacillus_sp__FJAT~27215
gi|902789152~Bacillus_sp__FJAT~27231
gi|982218377~Kurthia_sp__11kri321
gi|970425817~Staphylococcus_warneri
gi|890816399|emb|CLT10698_1|~Mycobacterium_tuberculosis
gi|670516662~Staphylococcus_warneri
gi|895808536|emb|COR89972_1|~Streptococcus_pneumoniae
gi|518065799~Staphylococcus_warneri
gi|515742201~Streptococcus_pneumoniae
gi|222121100|dbj|BAH18435_1|~Macrococcus_caseolyticus_JCSC5402
gi|753902367~Macrococcus_caseolyticus
gi|917740556~Salinicoccus_sp__YB14~2
gi|545645702~Gemella_bergeriae_ATCC_700627
gi|1011254052~Gemella_sp__oral_taxon_928
gi|489237628~Gemella_haemolysans_M341
gi|737388601~Acholeplasma_hippikon
gi|651266823~Acholeplasma_equifetale
gi|501200244~Acholeplasma_laidlawii
gi|524751451|emb|CDE49702_1|~Firmicutes_bacterium_CAG460
gi|524827881|emb|CDF19954_1|~Clostridium_sp__CAG609
gi|524817614|emb|CDF12107_1|~Mycoplasma_sp__CAG776
gi|984739046~Turicibacter_sp__H121
gi|932928124|emb|CUQ19907_1|~Turicibacter_sanguinis
gi|932931832|emb|CUN76412_1|~Turicibacter_sanguinis
gi|917074674~Virgibacillus_alimentarius
gi|849045248~Ornithinibacillus_contaminans
gi|544875558~Virgibacillus_sp__Vm~5
gi|651958984~Bacillus_megaterium
gi|740772871~Terribacillus_aidingensis
gi|811046513|emb|CQR48258_1|~Paraliobacillus_sp__PM~2
gi|654855001~Desulfitibacter_alkalitolerans
gi|334180190~Caldalkalibacillus_thermarum_TA2_A1
gi|757130988~Caldalkalibacillus_thermarum
gi|1025929724~Paenibacillus_sp__LPB0068
gi|767003523~Paenibacillus_sp__IHBB_10380
gi|655098105~Paenibacillus_taiwanensis
gi|489638169~Desulfotomaculum_nigrificans
gi|817123941~Syntrophomonas_zehnderi_OL~4
gi|499510404~Symbiobacterium_thermophilum_IAM_14863
gi|917663097~Thermincola_ferriacetica
gi|648627542~Anaeromusa_acidaminophila
gi|654468206~Anaeroarcus_burkinensis
gi|740881178~Pelosinus_sp__UFO1
gi|654417730~Megamonas_hypermegale
gi|653157951~Anaerovibrio_sp__RM50
gi|501768531~Halothermothrix_orenii_H_168
gi|836602003~Kosmotoga_pacifica
gi|1054975490~Sneathia_sanguinegens
gi|828174995~Marinitoga_sp__1155
gi|828186089~Marinitoga_sp__1197
gi|973039033~Petrotoga_mobilis
gi|969965094~Defluviitoga_tunisiensis
gi|973154124~Anaerolinaceae_bacterium_46_22
gi|504668856~Meiothermus_roseus_P3M~2
gi|516844340~Balneola_vulgaris
gi|1054313618~Balneola_sp__EhC07
gi|938259858~Bacteroidetes_bacterium_HLUCCA01
GSMAST4A1_ASY1_ANO1_7644_1
0.2
Archaea
Bacteria
SAG
NA

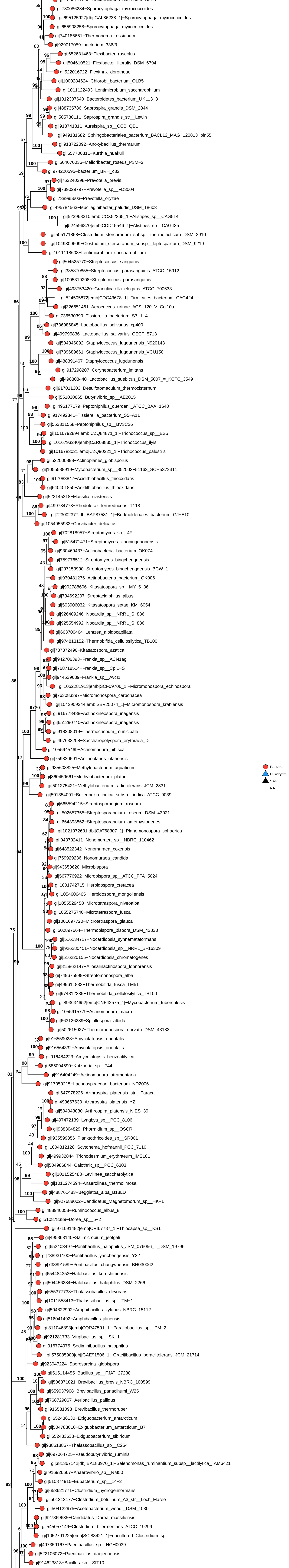

Bacteria
Eukaryota
SAG
NA
0.1

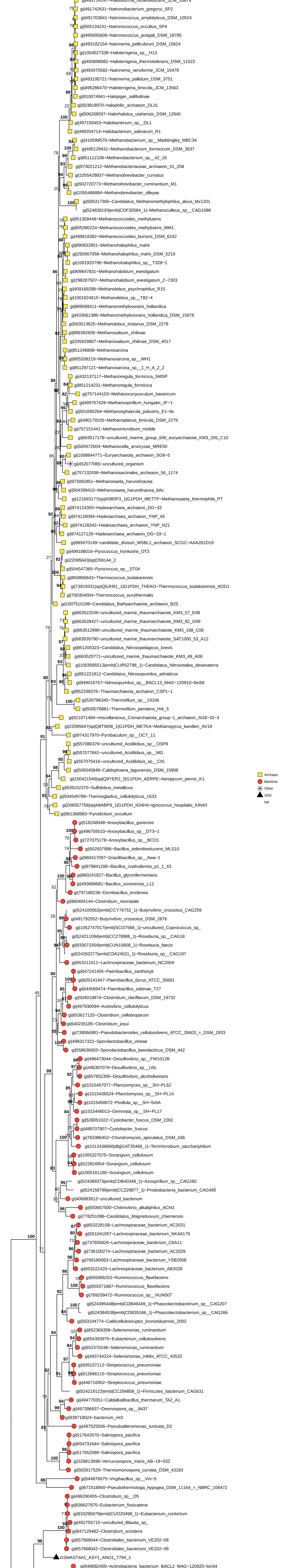

Archaea
Bacteria
Other
SAG
NA
0.1

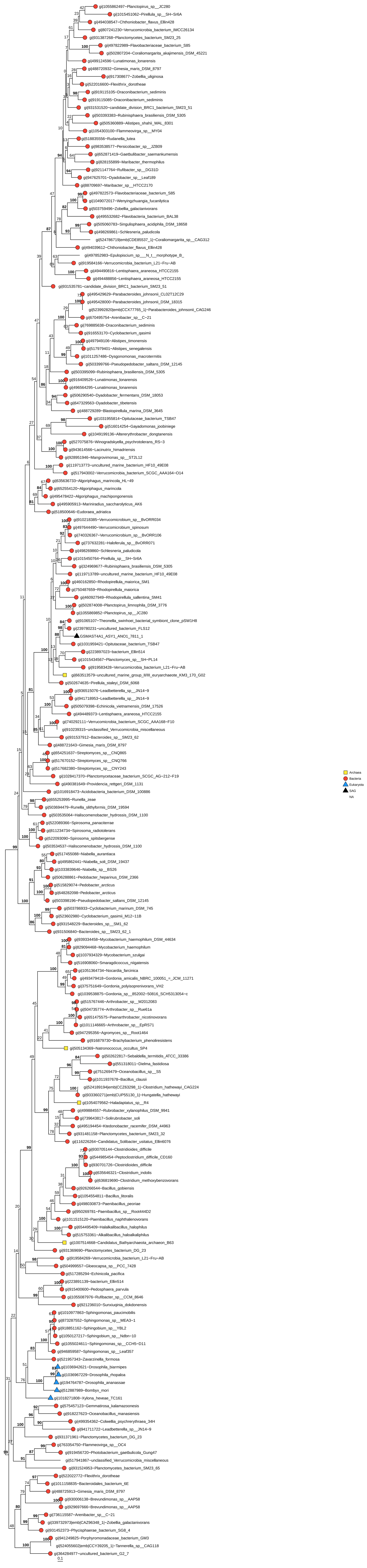
Archaea
Bacteria
Eukaryota
SAG
NA

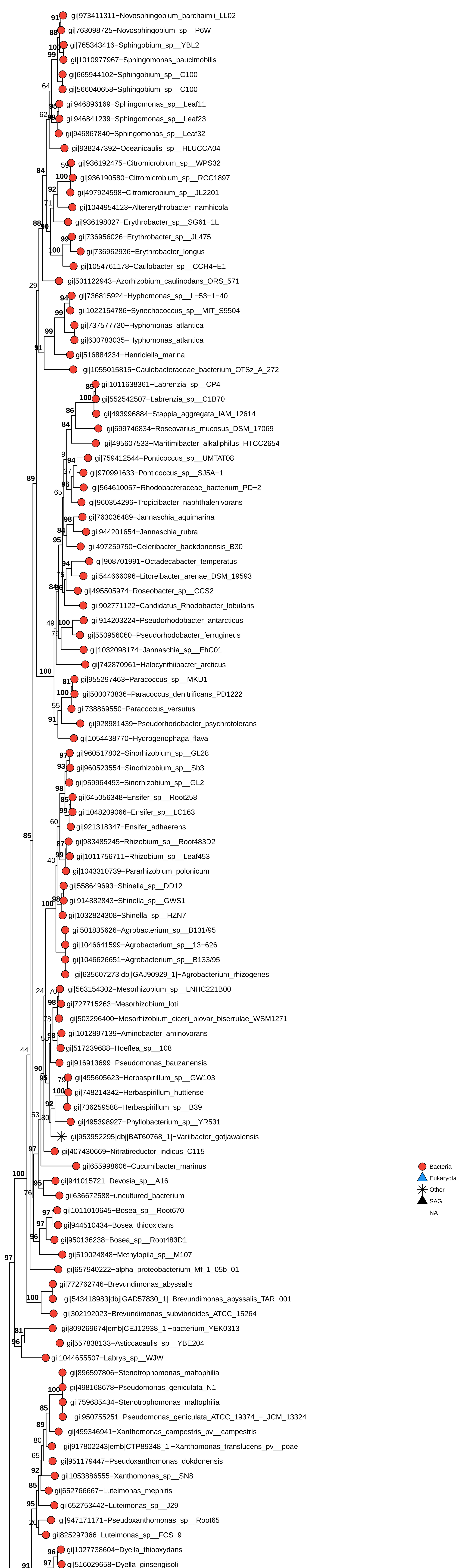

gi|973411311~Novosphingobium_barchaimii_LL02
gi|763098725~Novosphingobium_sp__P6W
gi|765343416~Sphingobium_sp__YBL2
gi|1010977967~Sphingomonas_paucimobilis
gi|665944102~Sphingobium_sp__C100
gi|566040658~Sphingobium_sp__C100
gi|946896169~Sphingomonas_sp__Leaf11
gi|946841239~Sphingomonas_sp__Leaf23
gi|946867840~Sphingomonas_sp__Leaf32
gi|938247392~Oceanicaulis_sp__HLUCCA04
gi|936192475~Citromicrobium_sp__WPS32
gi|936190580~Citromicrobium_sp__RCC1897
gi|497924598~Citromicrobium_sp__JL2201
gi|1044954123~Altererythrobacter_namhicola
gi|936198027~Erythrobacter_sp__SG61~1L
gi|736956026~Erythrobacter_sp__JL475
gi|736962936~Erythrobacter_longus
gi|1054761178~Caulobacter_sp__CCH4~E1
gi|501122943~Azorhizobium_caulinodans_ORS_571
gi|736815924~Hyphomonas_sp__L~53~1~40
gi|1022154786~Synechococcus_sp__MIT_S9504
gi|737577730~Hyphomonas_atlantica
gi|630783035~Hyphomonas_atlantica
gi|516884234~Henriciella_marina
gi|1055015815~Caulobacteraceae_bacterium_OTSz_A_272
gi|1011638361~Labrenzia_sp__CP4
gi|552542507~Labrenzia_sp__C1B70
gi|493996884~Stappia_aggregata_IAM_12614
gi|699746834~Roseovarius_mucosus_DSM_17069
gi|495607533~Maritimibacter_alkaliphilus_HTCC2654
gi|759412544~Ponticoccus_sp__UMTAT08
gi|970991633~Ponticoccus_sp__SJ5A~1
gi|564610057~Rhodobacteraceae_bacterium_PD~2
gi|960354296~Tropicibacter_naphthalenivorans
gi|763036489~Jannaschia_aquimarina
gi|944201654~Jannaschia_rubra
gi|497259750~Celeribacter_baekdonensis_B30
gi|908701991~Octadecabacter_temperatus
gi|544666096~Litoreibacter_arenae_DSM_19593
gi|495505974~Roseobacter_sp__CCS2
gi|902771122~Candidatus_Rhodobacter_lobularis
gi|914203224~Pseudorhodobacter_antarcticus
gi|550956060~Pseudorhodobacter_ferrugineus
gi|1032098174~Jannaschia_sp__EhC01
gi|742870961~Halocynthiibacter_arcticus
gi|955297463~Paracoccus_sp__MKU1
gi|500073836~Paracoccus_denitrificans_PD1222
gi|738869550~Paracoccus_versutus
gi|928981439~Pseudorhodobacter_psychrotolerans
gi|1054438770~Hydrogenophaga_flava
gi|960517802~Sinorhizobium_sp__GL28
gi|960523554~Sinorhizobium_sp__Sb3
gi|959964493~Sinorhizobium_sp__GL2
gi|645056348~Ensifer_sp__Root258
gi|1048209066~Ensifer_sp__LC163
gi|921318347~Ensifer_adhaerens
gi|983485245~Rhizobium_sp__Root483D2
gi|1011756711~Rhizobium_sp__Leaf453
gi|1043310739~Pararhizobium_polonicum
gi|558649693~Shinella_sp__DD12
gi|914882843~Shinella_sp__GWS1
gi|1032824308~Shinella_sp__HZN7
gi|501835626~Agrobacterium_sp__B131/95
gi|1046641599~Agrobacterium_sp__13~626
gi|1046626651~Agrobacterium_sp__B133/95
gi|635607273|dbj|GAJ90929_1|~Agrobacterium_rhizogenes
gi|563154302~Mesorhizobium_sp__LNHC221B00
gi|727715263~Mesorhizobium_loti
gi|503296400~Mesorhizobium_ciceri_biovar_biserrulae_WSM1271
gi|1012897139~Aminobacter_aminovorans
gi|517239688~Hoeflea_sp__108
gi|916913699~Pseudomonas_bauzanensis
gi|495605623~Herbaspirillum_sp__GW103
gi|748214342~Herbaspirillum_huttiense
gi|736259588~Herbaspirillum_sp__B39
gi|495398927~Phyllobacterium_sp__YR531
gi|953952295|dbj|BAT60768_1|~Variibacter_gotjawalensis
gi|407430669~Nitratireductor_indicus_C115
gi|655998606~Cucumibacter_marinus
gi|941015721~Devosia_sp__A16
gi|636672588~uncultured_bacterium
gi|1011010645~Bosea_sp__Root670
gi|944510434~Bosea_thiooxidans
gi|950136238~Bosea_sp__Root483D1
gi|519024848~Methylopila_sp__M107
gi|657940222~alpha_proteobacterium_Mf_1_05b_01
gi|772762746~Brevundimonas_abyssalis
gi|543418983|dbj|GAD57830_1|~Brevundimonas_abyssalis_TAR~001
gi|302192023~Brevundimonas_subvibrioides_ATCC_15264
gi|809269674|emb|CEJ12938_1|~bacterium_YEK0313
gi|557838133~Asticcacaulis_sp__YBE204
gi|1044655507~Labrys_sp__WJW
gi|896597806~Stenotrophomonas_maltophilia
gi|498168678~Pseudomonas_geniculata_N1
gi|759685434~Stenotrophomonas_maltophilia
gi|950755251~Pseudomonas_geniculata_ATCC_19374_=_JCM_13324
gi|499346941~Xanthomonas_campestris_pv__campestris
gi|917802243|emb|CTP89348_1|~Xanthomonas_translucens_pv__poae
gi|951179447~Pseudoxanthomonas_dokdonensis
gi|1053886555~Xanthomonas_sp__SN8
gi|652766667~Luteimonas_mephitis
gi|652753442~Luteimonas_sp__J29
gi|947171171~Pseudoxanthomonas_sp__Root65
gi|825297366~Luteimonas_sp__FCS~9
gi|1027738604~Dyella_thiooxydans
gi|516029658~Dyella_ginsengisoli
gi|759372424~Oleiagrimonas_soli
gi|738177738~Lysobacter_daejeonensis_GH1~9
gi|652420520~Arenimonas_composti_TR7~09_=_DSM_18010
gi|950053349~Lysobacter_sp__Root983
gi|947509435~Lysobacter_sp__Root604
gi|736086394~Arenimonas_donghaensis_DSM_18148_=_HO3~R19
gi|517801064~Rudaea_cellulosilytica
gi|916942214~Dyella_japonica
gi|797105389~Luteibacter_yeojuensis
gi|966435079~Legionella_quateirensis
gi|916583556~Legionella_moravica
gi|966514959~Legionella_rubrilucens
gi|290998535~Naegleria_gruberi
GSMAST4A1_ASY1_ANO1_7965_1
gi|1010977660~Collimonas_pratensis
gi|1004064386~Collimonas_fungivorans
gi|1004360565~Collimonas_arenae
gi|654434009~Pseudomonas_sp__URMO17WK12I3
gi|755678722~Pseudomonas_fulva
gi|498494378~Pseudomonas_sp__HPB0071
gi|948218930~Pelomonas_sp__Root1444
gi|947532702~Pelomonas_sp__Root1237
gi|921062698~Caenimonas_sp__SL110
gi|494567145~Rhodopirellula_baltica_SWK14
gi|831421337~Rhodopirellula_islandica
gi|460274124~Rhodopirellula_europaea_SH398
gi|1028050886~Gimesia_maris
gi|148843505~Gimesia_maris_DSM_8797
gi|196222814~Chthoniobacter_flavus_Ellin428
gi|948239168~Nocardioides_sp__Root240
gi|948090990~Nocardioides_sp__Root190
gi|739642387~Solirubrobacter_soli
gi|1004346682~Acinetobacter_venetianus
gi|497797593~Acinetobacter_sp__P8~3~8
gi|1041885925~Acinetobacter_gandensis
gi|651358661~Alkanindiges_illinoisensis
gi|494255998~Alcanivorax_sp__DG881
gi|1024903056~Alcanivorax_sp__HI0044
gi|1012707485~Alcanivorax_sp__NBRC_102024
gi|763064618~Ralstonia_solanacearum
gi|1028878989~Ralstonia_solanacearum
gi|504430787~Ralstonia_solanacearum
gi|759850213~Achromobacter_sp__RTa
gi|935571692~Achromobacter_sp__2789STDY5608625
gi|946914043~Xylophilus_sp__Leaf220
gi|947681525~Massilia_sp__Leaf139
gi|494940590~Caulobacter_sp__AP07
gi|917088667~Caulobacter_sp__UNC358MFTsu5_1
gi|917343619~Caulobacter_henricii
gi|948072594~Phenylobacterium_sp__Root700
gi|947346724~Phenylobacterium_sp__Root77
gi|497487401~Oceanicaulis_sp__HTCC2633
gi|550951758~Oceanicaulis_alexandrii
Bacteria
Eukaryota
Other
SAG
NA

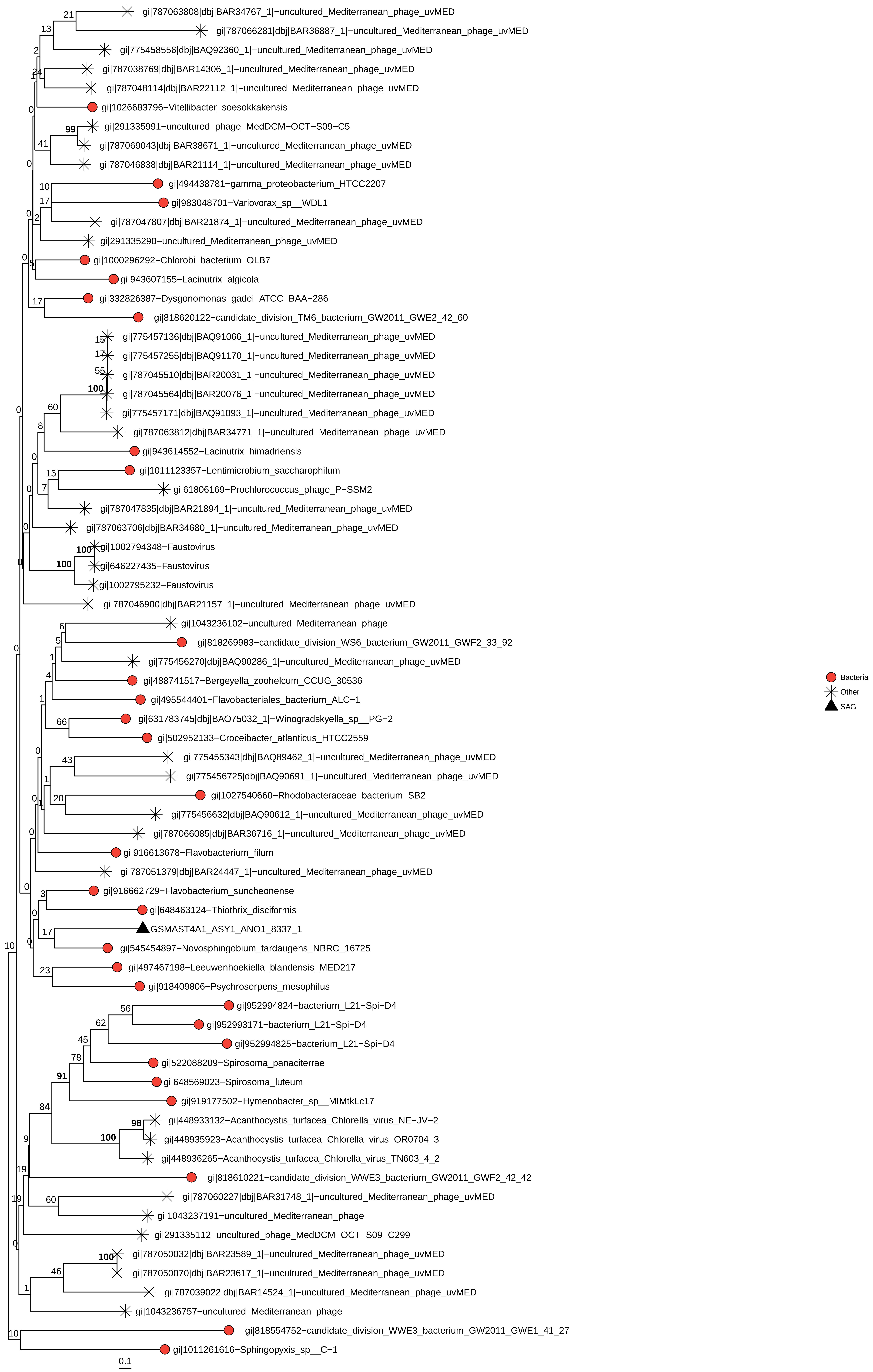
gi|787063808|dbj|BAR34767_1|−uncultured_Mediterranean_phage_uvMED
gi|787066281|dbj|BAR36887_1|−uncultured_Mediterranean_phage_uvMED
gi|775458556|dbj|BAQ92360_1|−uncultured_Mediterranean_phage_uvMED
gi|787038769|dbj|BAR14306_1|−uncultured_Mediterranean_phage_uvMED
gi|787048114|dbj|BAR22112_1|−uncultured_Mediterranean_phage_uvMED
gi|1026683796−Vitellibacter_soesokkakensis
gi|291335991−uncultured_phage_MedDCM−OCT−S09−C5
gi|787069043|dbj|BAR38671_1|−uncultured_Mediterranean_phage_uvMED
gi|787046838|dbj|BAR21114_1|−uncultured_Mediterranean_phage_uvMED
gi|494438781−gamma_proteobacterium_HTCC2207
gi|983048701−Variovorax_sp__WDL1
gi|787047807|dbj|BAR21874_1|−uncultured_Mediterranean_phage_uvMED
gi|291335290−uncultured_Mediterranean_phage_uvMED
gi|1000296292−Chlorobi_bacterium_OLB7
gi|943607155−Lacinutrix_algicola
gi|332826387−Dysgonomonas_gadei_ATCC_BAA−286
gi|818620122−candidate_division_TM6_bacterium_GW2011_GWE2_42_60
gi|775457136|dbj|BAQ91066_1|−uncultured_Mediterranean_phage_uvMED
gi|775457255|dbj|BAQ91170_1|−uncultured_Mediterranean_phage_uvMED
gi|787045510|dbj|BAR20031_1|−uncultured_Mediterranean_phage_uvMED
gi|787045564|dbj|BAR20076_1|−uncultured_Mediterranean_phage_uvMED
gi|775457171|dbj|BAQ91093_1|−uncultured_Mediterranean_phage_uvMED
gi|787063812|dbj|BAR34771_1|−uncultured_Mediterranean_phage_uvMED
gi|943614552−Lacinutrix_himadriensis
gi|1011123357−Lentimicrobium_saccharophilum
gi|61806169−Prochlorococcus_phage_P−SSM2
gi|787047835|dbj|BAR21894_1|−uncultured_Mediterranean_phage_uvMED
gi|787063706|dbj|BAR34680_1|−uncultured_Mediterranean_phage_uvMED
gi|1002794348−Faustovirus
gi|646227435−Faustovirus
gi|1002795232−Faustovirus
gi|787046900|dbj|BAR21157_1|−uncultured_Mediterranean_phage_uvMED
gi|1043236102−uncultured_Mediterranean_phage
gi|818269983−candidate_division_WS6_bacterium_GW2011_GWF2_33_92
gi|775456270|dbj|BAQ90286_1|−uncultured_Mediterranean_phage_uvMED
gi|488741517−Bergeyella_zoohelcum_CCUG_30536
gi|495544401−Flavobacteriales_bacterium_ALC−1
gi|631783745|dbj|BAO75032_1|−Winogradskyella_sp__PG−2
gi|502952133−Croceibacter_atlanticus_HTCC2559
gi|775455343|dbj|BAQ89462_1|−uncultured_Mediterranean_phage_uvMED
gi|775456725|dbj|BAQ90691_1|−uncultured_Mediterranean_phage_uvMED
gi|1027540660−Rhodobacteraceae_bacterium_SB2
gi|775456632|dbj|BAQ90612_1|−uncultured_Mediterranean_phage_uvMED
gi|787066085|dbj|BAR36716_1|−uncultured_Mediterranean_phage_uvMED
gi|916613678−Flavobacterium_filum
gi|787051379|dbj|BAR24447_1|−uncultured_Mediterranean_phage_uvMED
gi|916662729−Flavobacterium_suncheonense
gi|648463124−Thiothrix_disciformis
GSMAST4A1_ASY1_ANO1_8337_1
gi|545454897−Novosphingobium_tardaugens_NBRC_16725
gi|497467198−Leeuwenhoekiella_blandensis_MED217
gi|918409806−Psychroserpens_mesophilus
gi|952994824−bacterium_L21−Spi−D4
gi|952993171−bacterium_L21−Spi−D4
gi|952994825−bacterium_L21−Spi−D4
gi|522088209−Spirosoma_panaciterrae
gi|648569023−Spirosoma_luteum
gi|919177502−Hymenobacter_sp__MIMtkLc17
gi|448933132−Acanthocystis_turfacea_Chlorella_virus_NE−JV−2
gi|448935923−Acanthocystis_turfacea_Chlorella_virus_OR0704_3
gi|448936265−Acanthocystis_turfacea_Chlorella_virus_TN603_4_2
gi|818610221−candidate_division_WWE3_bacterium_GW2011_GWF2_42_42
gi|787060227|dbj|BAR31748_1|−uncultured_Mediterranean_phage_uvMED
gi|1043237191−uncultured_Mediterranean_phage
gi|291335112−uncultured_phage_MedDCM−OCT−S09−C299
gi|787050032|dbj|BAR23589_1|−uncultured_Mediterranean_phage_uvMED
gi|787050070|dbj|BAR23617_1|−uncultured_Mediterranean_phage_uvMED
gi|787039022|dbj|BAR14524_1|−uncultured_Mediterranean_phage_uvMED
gi|1043236757−uncultured_Mediterranean_phage
gi|818554752−candidate_division_WWE3_bacterium_GW2011_GWE1_41_27
gi|1011261616−Sphingopyxis_sp__C−1
0.1
Bacteria
Other
SAG

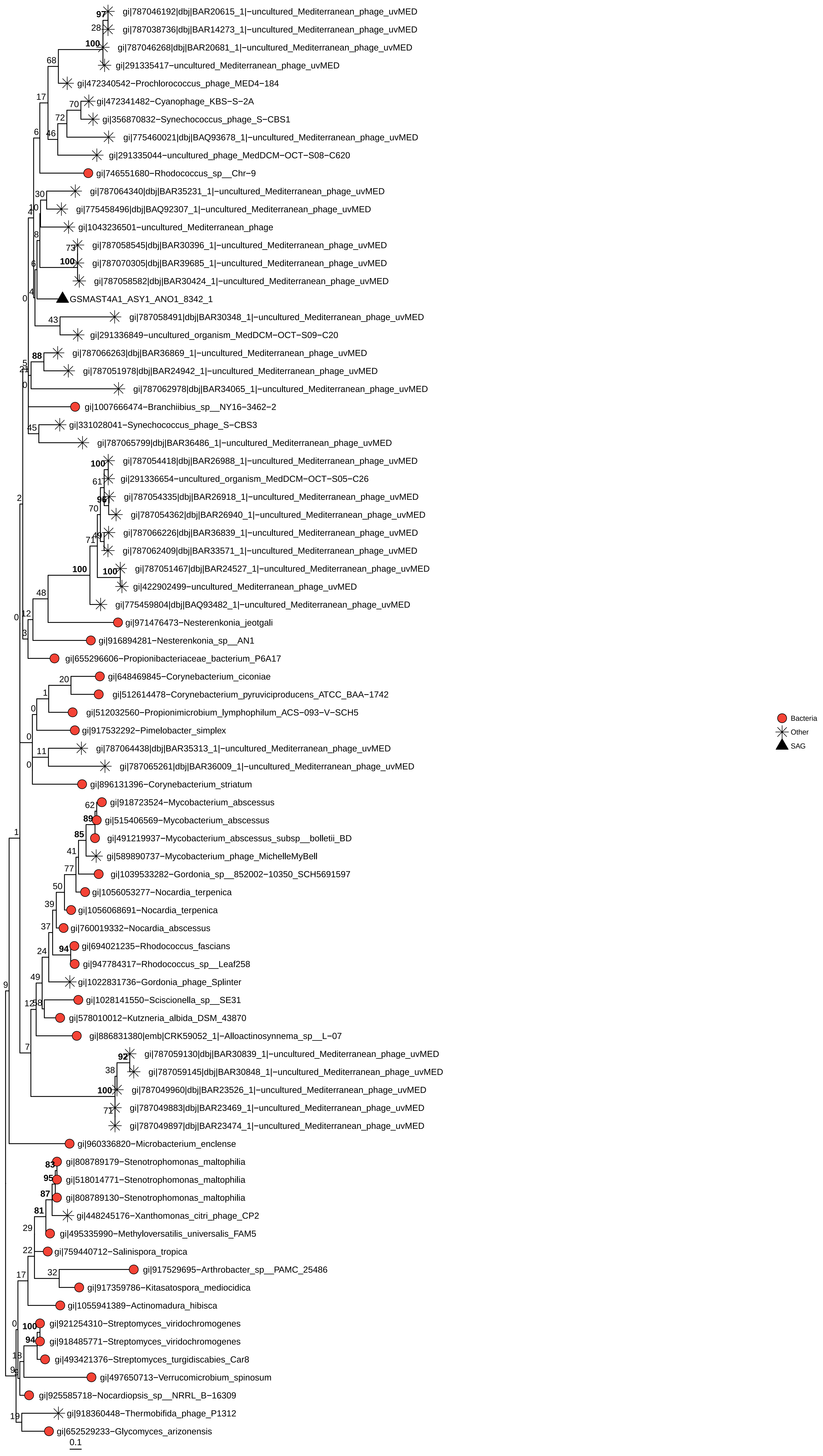

gi|787046192|dbj|BAR20615_1|−uncultured_Mediterranean_phage_uvMED
gi|787038736|dbj|BAR14273_1|−uncultured_Mediterranean_phage_uvMED
gi|787046268|dbj|BAR20681_1|−uncultured_Mediterranean_phage_uvMED
gi|291335417−uncultured_Mediterranean_phage_uvMED
gi|472340542−Prochlorococcus_phage_MED4−184
gi|472341482−Cyanophage_KBS−S−2A
gi|356870832−Synechococcus_phage_S−CBS1
gi|775460021|dbj|BAQ93678_1|−uncultured_Mediterranean_phage_uvMED
gi|291335044−uncultured_phage_MedDCM−OCT−S08−C620
gi|746551680−Rhodococcus_sp__Chr−9
gi|787064340|dbj|BAR35231_1|−uncultured_Mediterranean_phage_uvMED
gi|775458496|dbj|BAQ92307_1|−uncultured_Mediterranean_phage_uvMED
gi|1043236501−uncultured_Mediterranean_phage
gi|787058545|dbj|BAR30396_1|−uncultured_Mediterranean_phage_uvMED
gi|787070305|dbj|BAR39685_1|−uncultured_Mediterranean_phage_uvMED
gi|787058582|dbj|BAR30424_1|−uncultured_Mediterranean_phage_uvMED
GSMAST4A1_ASY1_ANO1_8342_1
gi|787058491|dbj|BAR30348_1|−uncultured_Mediterranean_phage_uvMED
gi|291336849−uncultured_organism_MedDCM−OCT−S09−C20
gi|787066263|dbj|BAR36869_1|−uncultured_Mediterranean_phage_uvMED
gi|787051978|dbj|BAR24942_1|−uncultured_Mediterranean_phage_uvMED
gi|787062978|dbj|BAR34065_1|−uncultured_Mediterranean_phage_uvMED
gi|1007666474−Branchiibius_sp__NY16−3462−2
gi|331028041−Synechococcus_phage_S−CBS3
gi|787065799|dbj|BAR36486_1|−uncultured_Mediterranean_phage_uvMED
gi|787054418|dbj|BAR26988_1|−uncultured_Mediterranean_phage_uvMED
gi|291336654−uncultured_organism_MedDCM−OCT−S05−C26
gi|787054335|dbj|BAR26918_1|−uncultured_Mediterranean_phage_uvMED
gi|787054362|dbj|BAR26940_1|−uncultured_Mediterranean_phage_uvMED
gi|787066226|dbj|BAR36839_1|−uncultured_Mediterranean_phage_uvMED
gi|787062409|dbj|BAR33571_1|−uncultured_Mediterranean_phage_uvMED
gi|787051467|dbj|BAR24527_1|−uncultured_Mediterranean_phage_uvMED
gi|422902499−uncultured_Mediterranean_phage_uvMED
gi|775459804|dbj|BAQ93482_1|−uncultured_Mediterranean_phage_uvMED
gi|971476473−Nesterenkonia_jeotgali
gi|916894281−Nesterenkonia_sp__AN1
gi|655296606−Propionibacteriaceae_bacterium_P6A17
gi|648469845−Corynebacterium_ciconiae
gi|512614478−Corynebacterium_pyruviciproducens_ATCC_BAA−1742
gi|512032560−Propionimicrobium_lymphophilum_ACS−093−V−SCH5
gi|917532292−Pimelobacter_simplex
gi|787064438|dbj|BAR35313_1|−uncultured_Mediterranean_phage_uvMED
gi|787065261|dbj|BAR36009_1|−uncultured_Mediterranean_phage_uvMED
gi|896131396−Corynebacterium_striatum
gi|918723524−Mycobacterium_abscessus
gi|515406569−Mycobacterium_abscessus
gi|491219937−Mycobacterium_abscessus_subsp__bolletii_BD
gi|589890737−Mycobacterium_phage_MichelleMyBell
gi|1039533282−Gordonia_sp__852002−10350_SCH5691597
gi|1056053277−Nocardia_terpenica
gi|1056068691−Nocardia_terpenica
gi|760019332−Nocardia_abscessus
gi|694021235−Rhodococcus_fascians
gi|947784317−Rhodococcus_sp__Leaf258
gi|1022831736−Gordonia_phage_Splinter
gi|1028141550−Sciscionella_sp__SE31
gi|578010012−Kutzneria_albida_DSM_43870
gi|886831380|emb|CRK59052_1|−Alloactinosynnema_sp__L−07
gi|787059130|dbj|BAR30839_1|−uncultured_Mediterranean_phage_uvMED
gi|787059145|dbj|BAR30848_1|−uncultured_Mediterranean_phage_uvMED
gi|787049960|dbj|BAR23526_1|−uncultured_Mediterranean_phage_uvMED
gi|787049883|dbj|BAR23469_1|−uncultured_Mediterranean_phage_uvMED
gi|787049897|dbj|BAR23474_1|−uncultured_Mediterranean_phage_uvMED
gi|960336820−Microbacterium_enclense
gi|808789179−Stenotrophomonas_maltophilia
gi|518014771−Stenotrophomonas_maltophilia
gi|808789130−Stenotrophomonas_maltophilia
gi|448245176−Xanthomonas_citri_phage_CP2
gi|495335990−Methyloversatilis_universalis_FAM5
gi|759440712−Salinispora_tropica
gi|917529695−Arthrobacter_sp__PAMC_25486
gi|917359786−Kitasatospora_mediocidica
gi|1055941389−Actinomadura_hibisca
gi|921254310−Streptomyces_viridochromogenes
gi|918485771−Streptomyces_viridochromogenes
gi|493421376−Streptomyces_turgidiscabies_Car8
gi|497650713−Verrucomicrobium_spinosum
gi|925585718−Nocardiopsis_sp__NRRL_B−16309
gi|918360448−Thermobifida_phage_P1312
gi|652529233−Glycomyces_arizonensis
0.1
Bacteria
Other
SAG

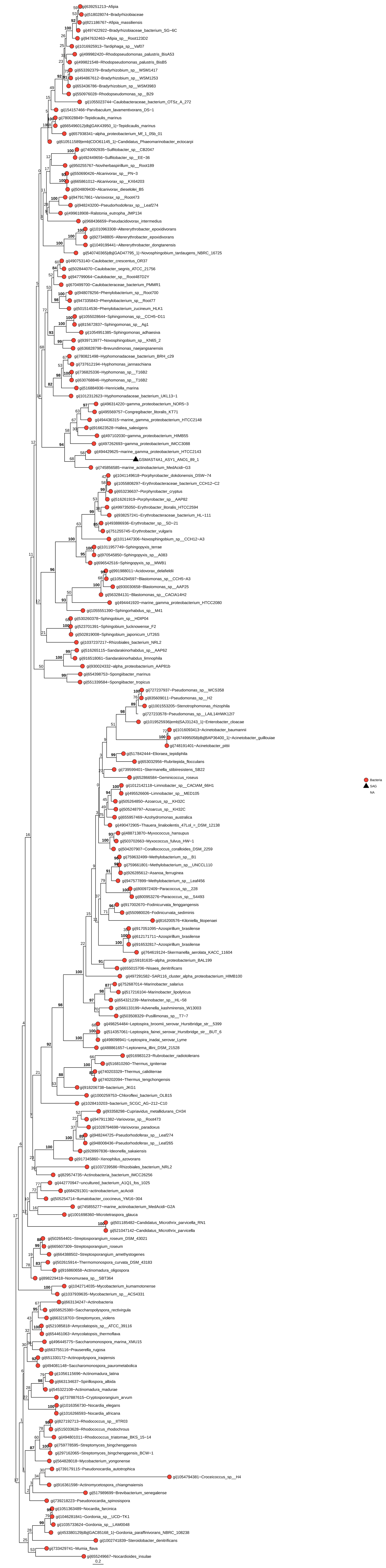

GSMAST4A1_ASY1_ANO1_89_1
Bacteria
SAG
NA
0.2

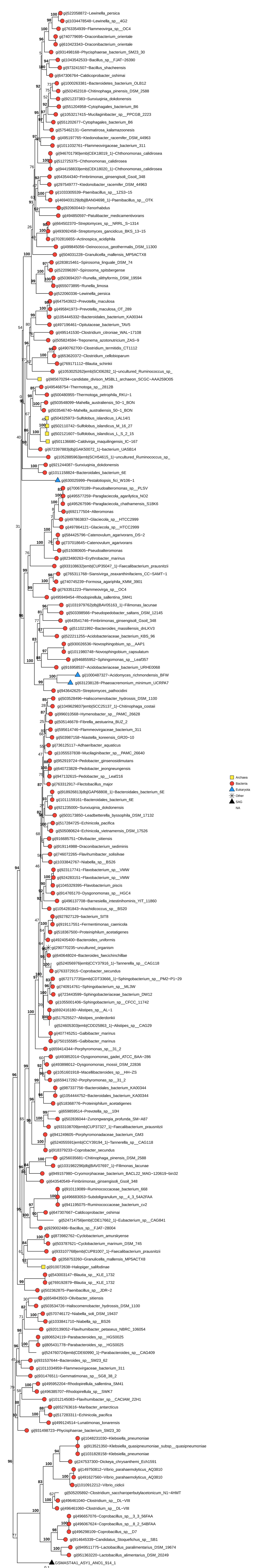
Archaea
Bacteria
Eukaryota
Other
SAG
NA
0.1

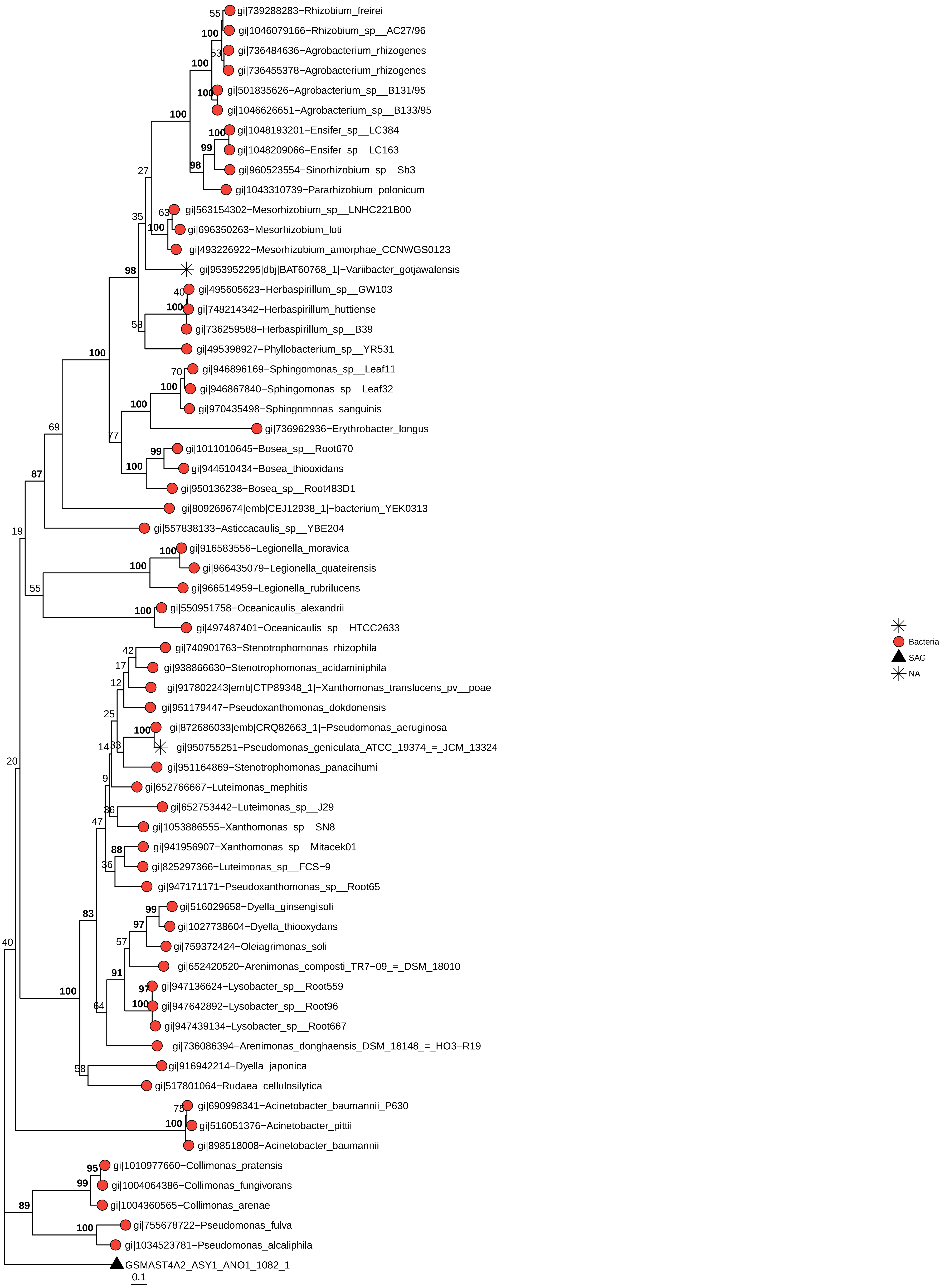
gi|739288283−Rhizobium_freirei
gi|1046079166−Rhizobium_sp__AC27/96
gi|736484636−Agrobacterium_rhizogenes
gi|736455378−Agrobacterium_rhizogenes
gi|501835626−Agrobacterium_sp__B131/95
gi|1046626651−Agrobacterium_sp__B133/95
gi|1048193201−Ensifer_sp__LC384
gi|1048209066−Ensifer_sp__LC163
gi|960523554−Sinorhizobium_sp__Sb3
gi|1043310739−Pararhizobium_polonicum
gi|563154302−Mesorhizobium_sp__LNHC221B00
gi|696350263−Mesorhizobium_loti
gi|493226922−Mesorhizobium_amorphae_CCNWGS0123
gi|953952295|dbj|BAT60768_1|−Variibacter_gotjawalensis
gi|495605623−Herbaspirillum_sp__GW103
gi|748214342−Herbaspirillum_huttiense
gi|736259588−Herbaspirillum_sp__B39
gi|495398927−Phyllobacterium_sp__YR531
gi|946896169−Sphingomonas_sp__Leaf11
gi|946867840−Sphingomonas_sp__Leaf32
gi|970435498−Sphingomonas_sanguinis
gi|736962936−Erythrobacter_longus
gi|1011010645−Bosea_sp__Root670
gi|944510434−Bosea_thiooxidans
gi|950136238−Bosea_sp__Root483D1
gi|809269674|emb|CEJ12938_1|−bacterium_YEK0313
gi|557838133−Asticcacaulis_sp__YBE204
gi|916583556−Legionella_moravica
gi|966435079−Legionella_quateirensis
gi|966514959−Legionella_rubrilucens
gi|550951758−Oceanicaulis_alexandrii
gi|497487401−Oceanicaulis_sp__HTCC2633
gi|740901763−Stenotrophomonas_rhizophila
gi|938866630−Stenotrophomonas_acidaminiphila
gi|917802243|emb|CTP89348_1|−Xanthomonas_translucens_pv__poae
gi|951179447−Pseudoxanthomonas_dokdonensis
gi|872686033|emb|CRQ82663_1|−Pseudomonas_aeruginosa
gi|950755251−Pseudomonas_geniculata_ATCC_19374_=_JCM_13324
gi|951164869−Stenotrophomonas_panacihumi
gi|652766667−Luteimonas_mephitis
gi|652753442−Luteimonas_sp__J29
gi|1053886555−Xanthomonas_sp__SN8
gi|941956907−Xanthomonas_sp__Mitacek01
gi|825297366−Luteimonas_sp__FCS−9
gi|947171171−Pseudoxanthomonas_sp__Root65
gi|516029658−Dyella_ginsengisoli
gi|1027738604−Dyella_thiooxydans
gi|759372424−Oleiagrimonas_soli
gi|652420520−Arenimonas_composti_TR7−09_=_DSM_18010
gi|947136624−Lysobacter_sp__Root559
gi|947642892−Lysobacter_sp__Root96
gi|947439134−Lysobacter_sp__Root667
gi|736086394−Arenimonas_donghaensis_DSM_18148_=_HO3−R19
gi|916942214−Dyella_japonica
gi|517801064−Rudaea_cellulosilytica
gi|690998341−Acinetobacter_baumannii_P630
gi|516051376−Acinetobacter_pittii
gi|898518008−Acinetobacter_baumannii
gi|1010977660−Collimonas_pratensis
gi|1004064386−Collimonas_fungivorans
gi|1004360565−Collimonas_arenae
gi|755678722−Pseudomonas_fulva
gi|1034523781−Pseudomonas_alcaliphila
GSMAST4A2_ASY1_ANO1_1082_1
0.1
Bacteria
SAG
NA

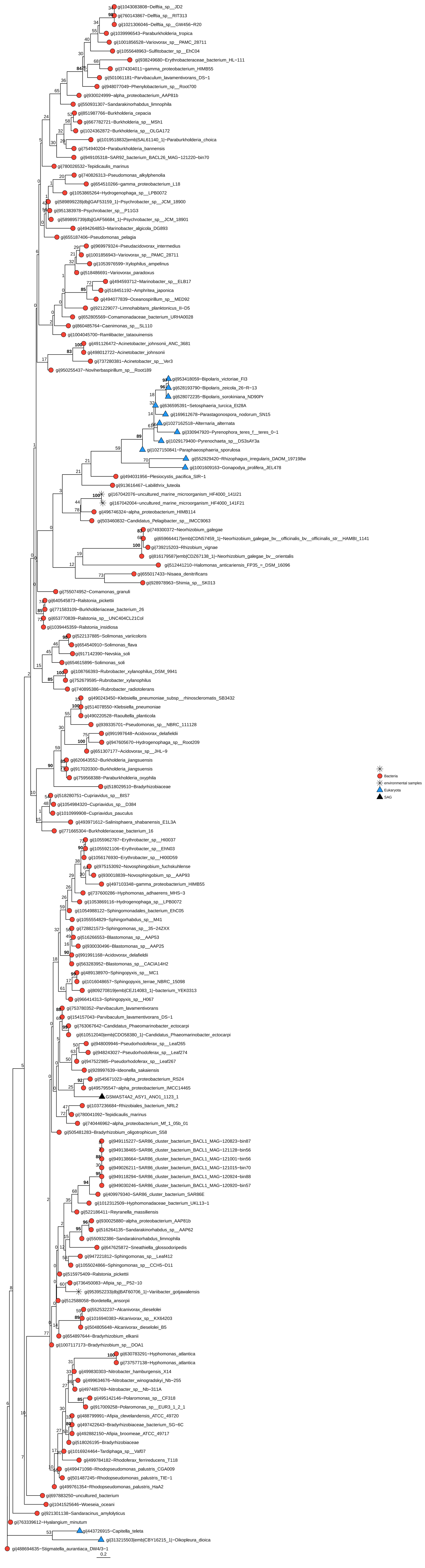

gi|1043083808~Delftia_sp__JD2
gi|760143867~Delftia_sp__RIT313
gi|1021306046~Delftia_sp__GW456~R20
gi|1039996543~Paraburkholderia_tropica
gi|1001856528~Variovorax_sp__PAMC_28711
gi|1055648963~Sulfitobacter_sp__EhC04
gi|938249680~Erythrobacteraceae_bacterium_HL~111
gi|374304011~gamma_proteobacterium_HIMB55
gi|501061181~Parvibaculum_lavamentivorans_DS~1
gi|948077049~Phenylobacterium_sp__Root700
gi|930024999~alpha_proteobacterium_AAP81b
gi|550931307~Sandarakinorhabdus_limnophila
gi|851987766~Burkholderia_cepacia
gi|667782721~Burkholderia_sp__MSh1
gi|1024362872~Burkholderia_sp__OLGA172
gi|1019518832|emb|SAL61140_1|~Paraburkholderia_choica
gi|754940204~Paraburkholderia_bannensis
gi|949105318~SAR92_bacterium_BACL26_MAG~121220~bin70
gi|780026532~Tepidicaulis_marinus
gi|740826313~Pseudomonas_alkylphenolia
gi|654510266~gamma_proteobacterium_L18
gi|1053865264~Hydrogenophaga_sp__LPB0072
gi|589899228|dbj|GAF53159_1|~Psychrobacter_sp__JCM_18900
gi|951383978~Psychrobacter_sp__P11G3
gi|589895739|dbj|GAF56684_1|~Psychrobacter_sp__JCM_18901
gi|494264853~Marinobacter_algicola_DG893
gi|655187406~Pseudomonas_pelagia
gi|969979324~Pseudacidovorax_intermedius
gi|1001856949~Variovorax_sp__PAMC_28711
gi|1053976599~Xylophilus_ampelinus
gi|518486691~Variovorax_paradoxus
gi|494593712~Marinobacter_sp__ELB17
gi|518451192~Amphritea_japonica
gi|494077839~Oceanospirillum_sp__MED92
gi|921229077~Limnohabitans_planktonicus_II~D5
gi|652805569~Comamonadaceae_bacterium_URHA0028
gi|860485764~Caenimonas_sp__SL110
gi|1004045700~Ramlibacter_tataouinensis
gi|491126472~Acinetobacter_johnsonii_ANC_3681
gi|498012722~Acinetobacter_johnsonii
gi|737280381~Acinetobacter_sp__Ver3
gi|950255437~Noviherbaspirillum_sp__Root189
gi|953418059~Bipolaris_victoriae_FI3
gi|628193790~Bipolaris_zeicola_26~R~13
gi|628072235~Bipolaris_sorokiniana_ND90Pr
gi|636595391~Setosphaeria_turcica_Et28A
gi|169612678~Parastagonospora_nodorum_SN15
gi|1027162518~Alternaria_alternata
gi|330947920~Pyrenophora_teres_f__teres_0~1
gi|1029179400~Pyrenochaeta_sp__DS3sAY3a
gi|1027150841~Paraphaeosphaeria_sporulosa
gi|552929420~Rhizophagus_irregularis_DAOM_197198w
gi|1001609163~Gonapodya_prolifera_JEL478
gi|494031956~Plesiocystis_pacifica_SIR~1
gi|913616467~Labilithrix_luteola
gi|167047076~uncultured_marine_microorganism_HF4000_141I21
gi|167042004~uncultured_marine_microorganism_HF4000_141F21
gi|496746324~alpha_proteobacterium_HIMB114
gi|503460832~Candidatus_Pelagibacter_sp__IMCC9063
gi|749300372~Neorhizobium_galegae
gi|659664417|emb|CDN57459_1|~Neorhizobium_galegae_bv__officinalis_bv__officinalis_str__HAMBI_1141
gi|739215203~Rhizobium_vignae
gi|816179587|emb|CDZ67138_1|~Neorhizobium_galegae_bv__orientalis
gi|512441210~Halomonas_anticariensis_FP35_=_DSM_16096
gi|655017433~Nisaea_denitrificans
gi|928978963~Shimia_sp__SK013
gi|755074952~Comamonas_granuli
gi|640545873~Ralstonia_pickettii
gi|771583109~Burkholderiaceae_bacterium_26
gi|653770839~Ralstonia_sp__UNC404CL21Col
gi|1039445359~Ralstonia_insidiosa
gi|522137885~Solimonas_variicoloris
gi|654540910~Solimonas_flava
gi|917142390~Nevskia_soli
gi|654615896~Solimonas_soli
gi|108766393~Rubrobacter_xylanophilus_DSM_9941
gi|752679595~Rubrobacter_xylanophilus
gi|740895386~Rubrobacter_radiotolerans
gi|490243450~Klebsiella_pneumoniae_subsp__rhinoscleromatis_SB3432
gi|514078550~Klebsiella_pneumoniae
gi|490220528~Raoultella_planticola
gi|939335701~Pseudomonas_sp__NBRC_111128
gi|991997648~Acidovorax_delafieldii
gi|947605670~Hydrogenophaga_sp__Root209
gi|651307177~Acidovorax_sp__JHL~9
gi|620643552~Burkholderia_jiangsuensis
gi|917020300~Burkholderia_jiangsuensis
gi|759568388~Paraburkholderia_oxyphila
gi|518029510~Bradyrhizobiaceae
gi|518280751~Cupriavidus_sp__BIS7
gi|1054984320~Cupriavidus_sp__D384
gi|1010999908~Cupriavidus_pauculus
gi|493971612~Salinisphaera_shabanensis_E1L3A
gi|771665304~Burkholderiaceae_bacterium_16
gi|1055962787~Erythrobacter_sp__HI0037
gi|1055921106~Erythrobacter_sp__EhN03
gi|1056176930~Erythrobacter_sp__HI00D59
gi|975153092~Novosphingobium_fuchskuhlense
gi|930018839~Novosphingobium_sp__AAP93
gi|497103348~gamma_proteobacterium_HIMB55
gi|737600286~Hyphomonas_adhaerens_MHS~3
gi|1053869116~Hydrogenophaga_sp__LPB0072
gi|1054988122~Sphingomonadales_bacterium_EhC05
gi|1055554829~Sphingorhabdus_sp__M41
gi|728821573~Sphingomonas_sp__35~24ZXX
gi|516266553~Blastomonas_sp__AAP53
gi|930030496~Blastomonas_sp__AAP25
gi|991991168~Acidovorax_delafieldii
gi|563283952~Blastomonas_sp__CACIA14H2
gi|489138970~Sphingopyxis_sp__MC1
gi|1016048657~Sphingopyxis_terrae_NBRC_15098
gi|809270819|emb|CEJ14083_1|~bacterium_YEK0313
gi|966414313~Sphingopyxis_sp__H067
gi|753780352~Parvibaculum_lavamentivorans
gi|154157043~Parvibaculum_lavamentivorans_DS~1
gi|763067642~Candidatus_Phaeomarinobacter_ectocarpi
gi|610512040|emb|CDO58380_1|~Candidatus_Phaeomarinobacter_ectocarpi
gi|948009946~Pseudorhodoferax_sp__Leaf265
gi|948243027~Pseudorhodoferax_sp__Leaf274
gi|947522985~Pseudorhodoferax_sp__Leaf267
gi|928897639~Ideonella_sakaiensis
gi|545671023~alpha_proteobacterium_RS24
gi|495795547~alpha_proteobacterium_IMCC14465
GSMAST4A2_ASY1_ANO1_1123_1
gi|1037236684~Rhizobiales_bacterium_NRL2
gi|780041092~Tepidicaulis_marinus
gi|740446962~alpha_proteobacterium_Mf_1_05b_01
gi|505481283~Bradyrhizobium_oligotrophicum_S58
gi|949115227~SAR86_cluster_bacterium_BACL1_MAG~120823~bin87
gi|949138465~SAR86_cluster_bacterium_BACL1_MAG~121128~bin56
gi|949138664~SAR86_cluster_bacterium_BACL1_MAG~121001~bin56
gi|949026211~SAR86_cluster_bacterium_BACL1_MAG~121015~bin70
gi|949118294~SAR86_cluster_bacterium_BACL1_MAG~120924~bin88
gi|949030246~SAR86_cluster_bacterium_BACL1_MAG~120920~bin57
gi|409979340~SAR86_cluster_bacterium_SAR86E
gi|1012312509~Hyphomonadaceae_bacterium_UKL13~1
gi|522186411~Reyranella_massiliensis
gi|930025880~alpha_proteobacterium_AAP81b
gi|516264135~Sandarakinorhabdus_sp__AAP62
gi|550932386~Sandarakinorhabdus_limnophila
gi|647625872~Sneathiella_glossodoripedis
gi|947221812~Sphingomonas_sp__Leaf412
gi|1055024866~Sphingomonas_sp__CCH5~D11
gi|515975409~Ralstonia_pickettii
gi|736450083~Afipia_sp__P52~10
gi|953952233|dbj|BAT60706_1|~Variibacter_gotjawalensis
gi|512588058~Bordetella_ansorpii
gi|552532237~Alcanivorax_dieselolei
gi|1016940383~Alcanivorax_sp__KX64203
gi|504805648~Alcanivorax_dieselolei_B5
gi|654897644~Bradyrhizobium_elkanii
gi|1007117173~Bradyrhizobium_sp__DOA1
gi|630783291~Hyphomonas_atlantica
gi|737577138~Hyphomonas_atlantica
gi|499830303~Nitrobacter_hamburgensis_X14
gi|499634676~Nitrobacter_winogradskyi_Nb~255
gi|497485769~Nitrobacter_sp__Nb~311A
gi|495142146~Polaromonas_sp__CF318
gi|917009258~Polaromonas_sp__EUR3_1_2_1
gi|488799991~Afipia_clevelandensis_ATCC_49720
gi|497422643~Bradyrhizobiaceae_bacterium_SG~6C
gi|492882150~Afipia_broomeae_ATCC_49717
gi|518026195~Bradyrhizobiaceae
gi|1016924464~Tardiphaga_sp__Vaf07
gi|499784182~Rhodoferax_ferrireducens_T118
gi|499471098~Rhodopseudomonas_palustris_CGA009
gi|501487245~Rhodopseudomonas_palustris_TIE~1
gi|499761354~Rhodopseudomonas_palustris_HaA2
gi|697883250~uncultured_bacterium
gi|1041525646~Woeseia_oceani
gi|921301138~Sandaracinus_amylolyticus
gi|763333612~Hyalangium_minutum
gi|443726915~Capitella_teleta
gi|313215503|emb|CBY16215_1|~Oikopleura_dioica
gi|488694635~Stigmatella_aurantiaca_DW4/3~1
0.2
Bacteria
environmental samples
Eukaryota
SAG

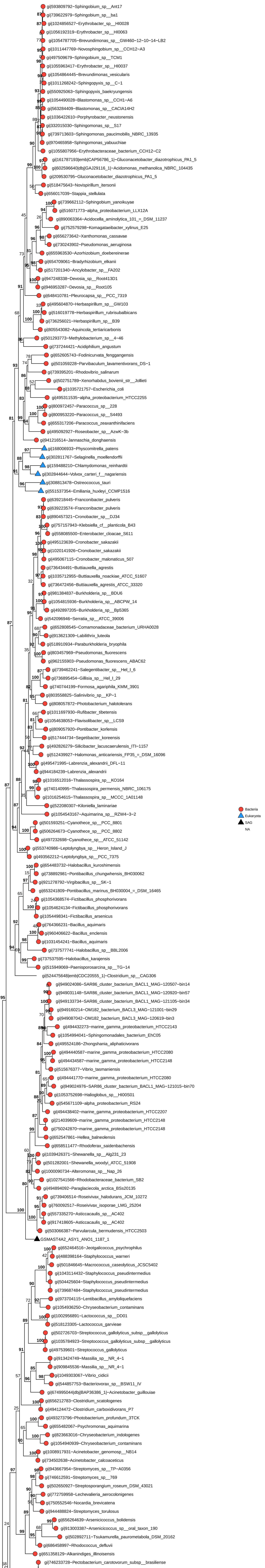

Bacteria
Eukaryota
SAG
NA
0.1

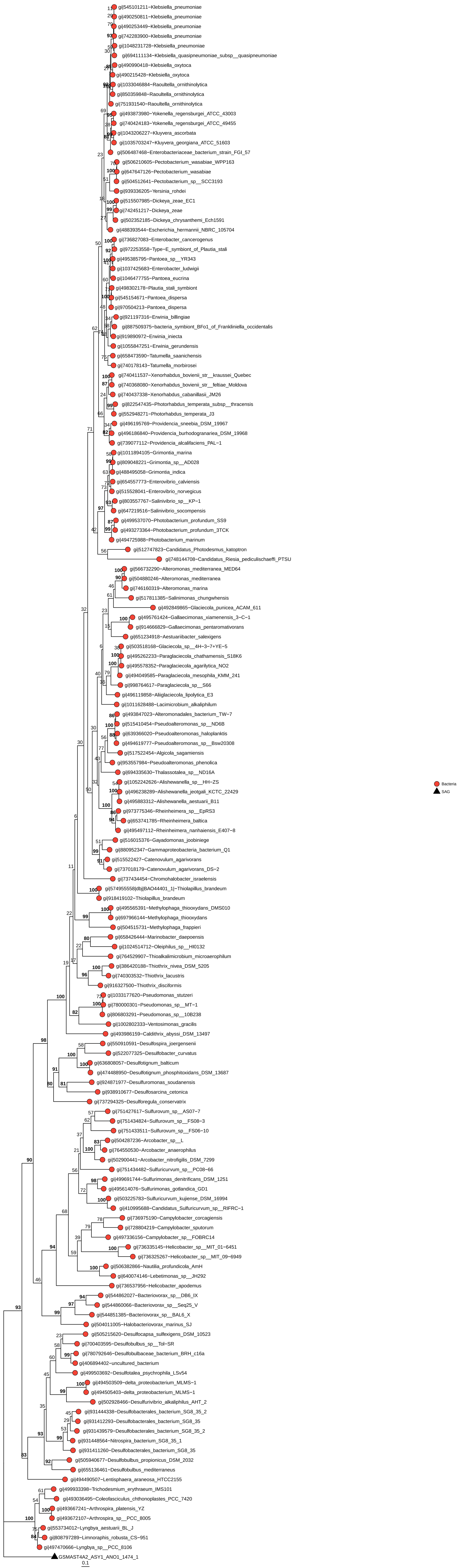
gi|545101211~Klebsiella_pneumoniae
gi|490250811~Klebsiella_pneumoniae
gi|490253449~Klebsiella_pneumoniae
gi|742283900~Klebsiella_pneumoniae
gi|1048231728~Klebsiella_pneumoniae
gi|694111134~Klebsiella_quasipneumoniae_subsp__quasipneumoniae
gi|490990418~Klebsiella_oxytoca
gi|490215428~Klebsiella_oxytoca
gi|1033046884~Raoultella_ornithinolytica
gi|850359848~Raoultella_ornithinolytica
gi|751931540~Raoultella_ornithinolytica
gi|493873980~Yokenella_regensburgei_ATCC_43003
gi|740424183~Yokenella_regensburgei_ATCC_49455
gi|1043206227~Kluyvera_ascorbata
gi|1035703247~Kluyvera_georgiana_ATCC_51603
gi|506487468~Enterobacteriaceae_bacterium_strain_FGI_57
gi|506210605~Pectobacterium_wasabiae_WPP163
gi|647647126~Pectobacterium_wasabiae
gi|504512641~Pectobacterium_sp__SCC3193
gi|939336205~Yersinia_rohdei
gi|515507985~Dickeya_zeae_EC1
gi|742451217~Dickeya_zeae
gi|502352185~Dickeya_chrysanthemi_Ech1591
gi|488393544~Escherichia_hermannii_NBRC_105704
gi|736827083~Enterobacter_cancerogenus
gi|972253558~Type−E_symbiont_of_Plautia_stali
gi|495385795~Pantoea_sp__YR343
gi|1037425683~Enterobacter_ludwigii
gi|1046477755~Pantoea_eucrina
gi|498302178~Plautia_stali_symbiont
gi|545154671~Pantoea_dispersa
gi|970504213~Pantoea_dispersa
gi|921197316~Erwinia_billingiae
gi|887509375~bacteria_symbiont_BFo1_of_Frankliniella_occidentalis
gi|919890972~Erwinia_iniecta
gi|1055847251~Erwinia_gerundensis
gi|658473590~Tatumella_saanichensis
gi|740178143~Tatumella_morbirosei
gi|740411537~Xenorhabdus_bovienii_str__kraussei_Quebec
gi|740368080~Xenorhabdus_bovienii_str__feltiae_Moldova
gi|740437338~Xenorhabdus_cabanillasii_JM26
gi|822547435~Photorhabdus_temperata_subsp__thracensis
gi|552948271~Photorhabdus_temperata_J3
gi|496195769~Providencia_sneebia_DSM_19967
gi|496186840~Providencia_burhodogranariea_DSM_19968
gi|739077112~Providencia_alcalifaciens_PAL−1
gi|1011894105~Grimontia_marina
gi|809048221~Grimontia_sp__AD028
gi|488495058~Grimontia_indica
gi|654557773~Enterovibrio_calviensis
gi|515528041~Enterovibrio_norvegicus
gi|803557767~Salinivibrio_sp__KP−1
gi|647219516~Salinivibrio_socompensis
gi|499537070~Photobacterium_profundum_SS9
gi|493273364~Photobacterium_profundum_3TCK
gi|494725988~Photobacterium_marinum
gi|512747823~Candidatus_Photodesmus_katoptron
gi|748144708~Candidatus_Riesia_pediculischaeffi_PTSU
gi|566732290~Alteromonas_mediterranea_MED64
gi|504880246~Alteromonas_mediterranea
gi|746160319~Alteromonas_marina
gi|517811385~Salinimonas_chungwhensis
gi|492849865~Glaciecola_punicea_ACAM_611
gi|495761424~Gallaecimonas_xiamenensis_3−C−1
gi|914666829~Gallaecimonas_pentaromativorans
gi|651234918~Aestuariibacter_salexigens
gi|503518168~Glaciecola_sp__4H−3−7+YE−5
gi|495262233~Paraglaciecola_chathamensis_S18K6
gi|495578352~Paraglaciecola_agarilytica_NO2
gi|494049585~Paraglaciecola_mesophila_KMM_241
gi|998764617~Paraglaciecola_sp__S66
gi|496119858~Aliiglaciecola_lipolytica_E3
gi|1011628488~Lacimicrobium_alkaliphilum
gi|493847023~Alteromonadales_bacterium_TW−7
gi|515410454~Pseudoalteromonas_sp__ND6B
gi|639366020~Pseudoalteromonas_haloplanktis
gi|494619777~Pseudoalteromonas_sp__Bsw20308
gi|517522454~Algicola_sagamiensis
gi|953557984~Pseudoalteromonas_phenolica
gi|694335630~Thalassotalea_sp__ND16A
gi|1052242626~Alishewanella_sp__HH−ZS
gi|496238289~Alishewanella_jeotgali_KCTC_22429
gi|495883312~Alishewanella_aestuarii_B11
gi|973775346~Rheinheimera_sp__EpRS3
gi|653741785~Rheinheimera_baltica
gi|495497112~Rheinheimera_nanhaiensis_E407−8
gi|516015376~Gayadomonas_joobiniege
gi|880952347~Gammaproteobacteria_bacterium_Q1
gi|515522427~Catenovulum_agarivorans
gi|737018179~Catenovulum_agarivorans_DS−2
gi|737434454~Chromohalobacter_israelensis
gi|574955558|dbj|BAO44401_1|~Thiolapillus_brandeum
gi|918419102~Thiolapillus_brandeum
gi|495565391~Methylophaga_thiooxydans_DMS010
gi|697966144~Methylophaga_thiooxydans
gi|504515731~Methylophaga_frappieri
gi|658426444~Marinobacter_daepoensis
gi|1024514712~Oleiphilus_sp__HI0132
gi|764529907~Thioalkalimicrobium_microaerophilum
gi|386420188~Thiothrix_nivea_DSM_5205
gi|740303532~Thiothrix_lacustris
gi|916327500~Thiothrix_disciformis
gi|1033177620~Pseudomonas_stutzeri
gi|780000301~Pseudomonas_sp__MT−1
gi|806803291~Pseudomonas_sp__10B238
gi|1002802333~Ventosimonas_gracilis
gi|493986159~Caldithrix_abyssi_DSM_13497
gi|550910591~Desulfospira_joergensenii
gi|522077325~Desulfobacter_curvatus
gi|636808057~Desulfotignum_balticum
gi|474488950~Desulfotignum_phosphitoxidans_DSM_13687
gi|924871977~Desulfuromonas_soudanensis
gi|938910677~Desulfuromonas_cetonica
gi|737294325~Desulforegula_conservatrix
gi|751427617~Sulfurovum_sp__AS07−7
gi|751434824~Sulfurovum_sp__FS08−3
gi|751433511~Sulfurovum_sp__FS06−10
gi|504287236~Arcobacter_sp__L
gi|764550530~Arcobacter_anaerophilus
gi|502900441~Arcobacter_nitrofigilis_DSM_7299
gi|751434482~Sulfuricurvum_sp__PC08−66
gi|499691744~Sulfurimonas_denitrificans_DSM_1251
gi|495614076~Sulfurimonas_gotlandica_GD1
gi|503225783~Sulfuricurvum_kujiense_DSM_16994
gi|410995688~Candidatus_Sulfuricurvum_sp__RIFRC−1
gi|736975190~Campylobacter_corcagiensis
gi|728804219~Campylobacter_sputorum
gi|497336156~Campylobacter_sp__FOBRC14
gi|736335145~Helicobacter_sp__MIT_01−6451
gi|736325267~Helicobacter_sp__MIT_09−6949
gi|506382866~Nautilia_profundicola_AmH
gi|640074146~Lebetimonas_sp__JH292
gi|736537956~Helicobacter_apodemus
gi|544862027~Bacteriovorax_sp__DB6_IX
gi|544860066~Bacteriovorax_sp__Seq25_V
gi|544851385~Bacteriovorax_sp__BAL6_X
gi|504011005~Halobacteriovorax_marinus_SJ
gi|505215620~Desulfocapsa_sulfexigens_DSM_10523
gi|700403595~Desulfobulbus_sp__Tol−SR
gi|780792646~Desulfobulbaceae_bacterium_BRH_c16a
gi|406894402~uncultured_bacterium
gi|499503692~Desulfotalea_psychrophila_LSv54
gi|494503509~delta_proteobacterium_MLMS−1
gi|494505403~delta_proteobacterium_MLMS−1
gi|502928466~Desulfurivibrio_alkaliphilus_AHT_2
gi|931444338~Desulfobacterales_bacterium_SG8_35_2
gi|931412293~Desulfobacterales_bacterium_SG8_35
gi|931439579~Desulfobacterales_bacterium_SG8_35_2
gi|931448564~Nitrospira_bacterium_SG8_35_1
gi|931411260~Desulfobacterales_bacterium_SG8_35
gi|505940677~Desulfobulbus_propionicus_DSM_2032
gi|655136461~Desulfobulbus_mediterraneus
gi|494490507~Lentisphaera_araneosa_HTCC2155
gi|499933398~Trichodesmium_erythraeum_IMS101
gi|493036495~Coleofasciculus_chthonoplastes_PCC_7420
gi|493667241~Arthrospira_platensis_YZ
gi|493672107~Arthrospira_sp__PCC_8005
gi|553734012~Lyngbya_aestuarii_BL_J
gi|808797289~Limnoraphis_robusta_CS−951
gi|497470666~Lyngbya_sp__PCC_8106
GSMAST4A2_ASY1_ANO1_1474_1
0.1
Bacteria
SAG

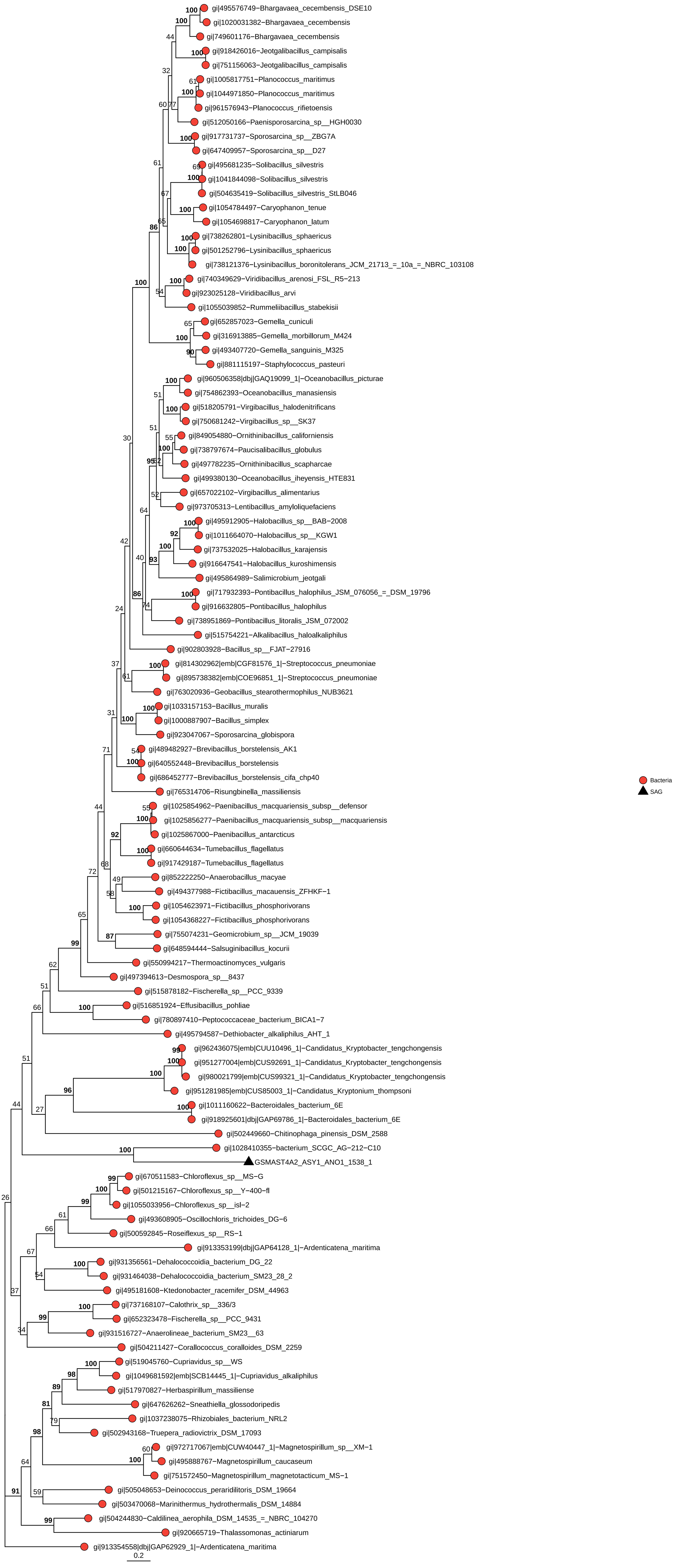
gi|495576749−Bhargavaea_cecembensis_DSE10
gi|1020031382−Bhargavaea_cecembensis
gi|749601176−Bhargavaea_cecembensis
gi|918426016−Jeotgalibacillus_campisalis
gi|751156063−Jeotgalibacillus_campisalis
gi|1005817751−Planococcus_maritimus
gi|1044971850−Planococcus_maritimus
gi|961576943−Planococcus_rifietoensis
gi|512050166−Paenisporosarcina_sp__HGH0030
gi|917731737−Sporosarcina_sp__ZBG7A
gi|647409957−Sporosarcina_sp__D27
gi|495681235−Solibacillus_silvestris
gi|1041844098−Solibacillus_silvestris
gi|504635419−Solibacillus_silvestris_StLB046
gi|1054784497−Caryophanon_tenue
gi|1054698817−Caryophanon_latum
gi|738262801−Lysinibacillus_sphaericus
gi|501252796−Lysinibacillus_sphaericus
gi|738121376−Lysinibacillus_boronitolerans_JCM_21713_=_10a_=_NBRC_103108
gi|740349629−Viridibacillus_arenosi_FSL_R5−213
gi|923025128−Viridibacillus_arvi
gi|1055039852−Rummeliibacillus_stabekisii
gi|652857023−Gemella_cuniculi
gi|316913885−Gemella_morbillorum_M424
gi|493407720−Gemella_sanguinis_M325
gi|881115197−Staphylococcus_pasteuri
gi|960506358|dbj|GAQ19099_1|−Oceanobacillus_picturae
gi|754862393−Oceanobacillus_manasiensis
gi|518205791−Virgibacillus_halodenitrificans
gi|750681242−Virgibacillus_sp__SK37
gi|849054880−Ornithinibacillus_californiensis
gi|738797674−Paucisalibacillus_globulus
gi|497782235−Ornithinibacillus_scapharcae
gi|499380130−Oceanobacillus_iheyensis_HTE831
gi|657022102−Virgibacillus_alimentarius
gi|973705313−Lentibacillus_amyloliquefaciens
gi|495912905−Halobacillus_sp__BAB−2008
gi|1011664070−Halobacillus_sp__KGW1
gi|737532025−Halobacillus_karajensis
gi|916647541−Halobacillus_kuroshimensis
gi|495864989−Salimicrobium_jeotgali
gi|717932393−Pontibacillus_halophilus_JSM_076056_=_DSM_19796
gi|916632805−Pontibacillus_halophilus
gi|738951869−Pontibacillus_litoralis_JSM_072002
gi|515754221−Alkalibacillus_haloalkaliphilus
gi|902803928−Bacillus_sp__FJAT−27916
gi|814302962|emb|CGF81576_1|−Streptococcus_pneumoniae
gi|895738382|emb|COE96851_1|−Streptococcus_pneumoniae
gi|763020936−Geobacillus_stearothermophilus_NUB3621
gi|1033157153−Bacillus_muralis
gi|1000887907−Bacillus_simplex
gi|923047607−Sporosarcina_globispora
gi|489482927−Brevibacillus_borstelensis_AK1
gi|640552448−Brevibacillus_borstelensis
gi|686452777−Brevibacillus_borstelensis_cifa_chp40
gi|765314706−Risungbinella_massiliensis
gi|1025854962−Paenibacillus_macquariensis_subsp__defensor
gi|1025856277−Paenibacillus_macquariensis_subsp__macquariensis
gi|1025867000−Paenibacillus_antarcticus
gi|660644634−Tumebacillus_flagellatus
gi|917429187−Tumebacillus_flagellatus
gi|852222250−Anaerobacillus_macyae
gi|494377988−Fictibacillus_macauensis_ZFHKF−1
gi|1054623971−Fictibacillus_phosphorivorans
gi|1054368227−Fictibacillus_phosphorivorans
gi|755074231−Geomicrobium_sp__JCM_19039
gi|648594444−Salsuginibacillus_kocurii
gi|550994217−Thermoactinomyces_vulgaris
gi|497394613−Desmospora_sp__8437
gi|515878182−Fischerella_sp__PCC_9339
gi|516851924−Effusibacillus_pohliae
gi|780897410−Peptococcaceae_bacterium_BICA1−7
gi|495794587−Dethiobacter_alkaliphilus_AHT_1
gi|962436075|emb|CUU10496_1|−Candidatus_Kryptobacter_tengchongensis
gi|951277004|emb|CUS92691_1|−Candidatus_Kryptobacter_tengchongensis
gi|980021799|emb|CUS99321_1|−Candidatus_Kryptobacter_tengchongensis
gi|951281985|emb|CUS85003_1|−Candidatus_Kryptonium_thompsoni
gi|1011160622−Bacteroidales_bacterium_6E
gi|918925601|dbj|GAP69786_1|−Bacteroidales_bacterium_6E
gi|502449660−Chitinophaga_pinensis_DSM_2588
gi|1028410355−bacterium_SCGC_AG−212−C10
GSMAST4A2_ASY1_ANO1_1538_1
gi|670511583−Chloroflexus_sp__MS−G
gi|501215167−Chloroflexus_sp__Y−400−fl
gi|1055033956−Chloroflexus_sp__isl−2
gi|493608905−Oscillochloris_trichoides_DG−6
gi|500592845−Roseiflexus_sp__RS−1
gi|913353199|dbj|GAP64128_1|−Ardenticatena_maritima
gi|931356561−Dehalococcoidia_bacterium_DG_22
gi|931464038−Dehalococcoidia_bacterium_SM23_28_2
gi|495181608−Ktedonobacter_racemifer_DSM_44963
gi|737168107−Calothrix_sp__336/3
gi|652323478−Fischerella_sp__PCC_9431
gi|931516727−Anaerolineae_bacterium_SM23__63
gi|504211427−Corallococcus_coralloides_DSM_2259
gi|519045760−Cupriavidus_sp__WS
gi|1049681592|emb|SCB14445_1|−Cupriavidus_alkaliphilus
gi|517970827−Herbaspirillum_massiliense
gi|647626262−Sneathiella_glossodoripedis
gi|1037238075−Rhizobiales_bacterium_NRL2
gi|502943168−Truepera_radiovictrix_DSM_17093
gi|972717067|emb|CUW40447_1|−Magnetospirillum_sp__XM−1
gi|495888767−Magnetospirillum_caucaseum
gi|751572450−Magnetospirillum_magnetotacticum_MS−1
gi|505048653−Deinococcus_peraridilitoris_DSM_19664
gi|503470068−Marinithermus_hydrothermalis_DSM_14884
gi|504244830−Caldilinea_aerophila_DSM_14535_=_NBRC_104270
gi|920665719−Thalassomonas_actiniarum
gi|913354558|dbj|GAP62929_1|−Ardenticatena_maritima
0.2
Bacteria
SAG

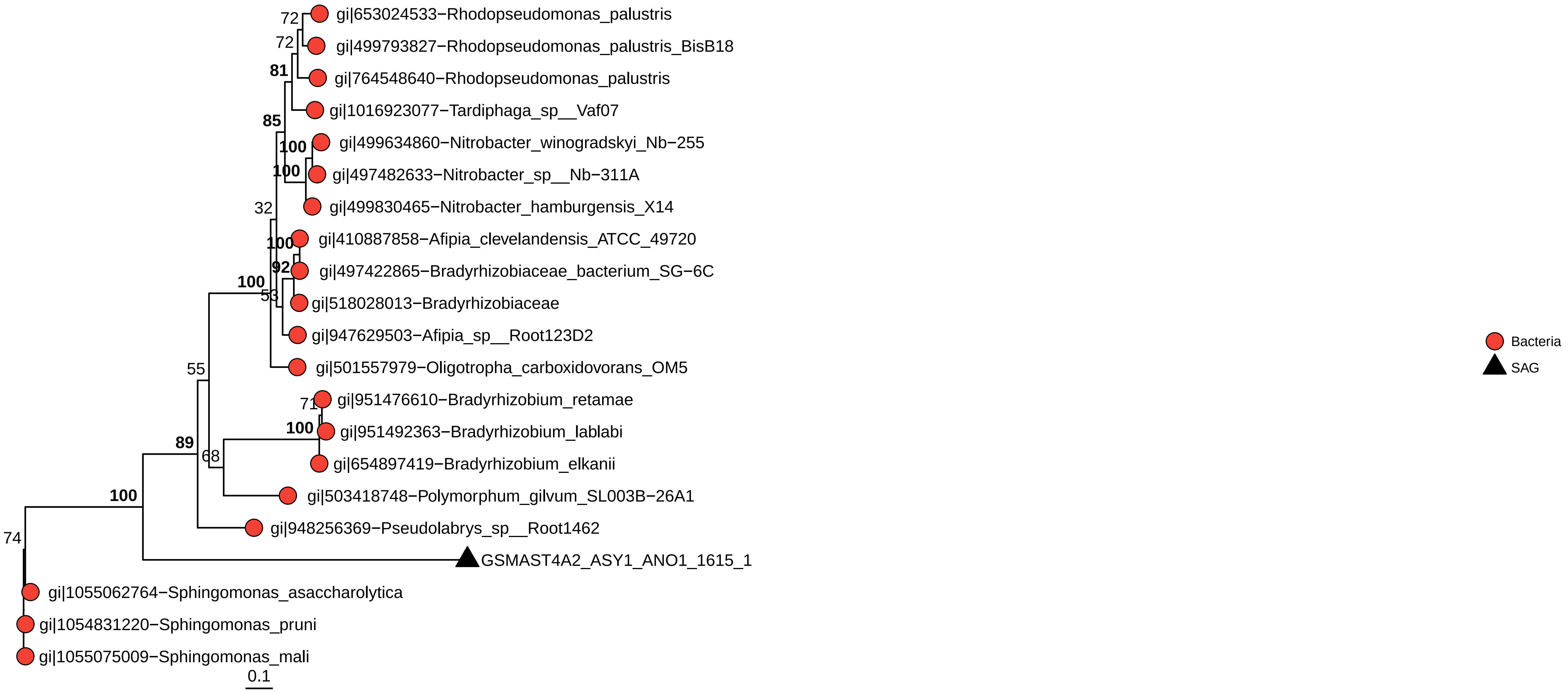

gi|653024533−Rhodopseudomonas_palustris
gi|499793827−Rhodopseudomonas_palustris_BisB18
gi|764548640−Rhodopseudomonas_palustris
gi|1016923077−Tardiphaga_sp__Vaf07
gi|499634860−Nitrobacter_winogradskyi_Nb−255
gi|497482633−Nitrobacter_sp__Nb−311A
gi|499830465−Nitrobacter_hamburgensis_X14
gi|410887858−Afipia_clevelandensis_ATCC_49720
gi|497422865−Bradyrhizobiaceae_bacterium_SG−6C
gi|518028013−Bradyrhizobiaceae
gi|947629503−Afipia_sp__Root123D2
gi|501557979−Oligotropha_carboxidovorans_OM5
gi|951476610−Bradyrhizobium_retamae
gi|951492363−Bradyrhizobium_lablabi
gi|654897419−Bradyrhizobium_elkanii
gi|503418748−Polymorphum_gilvum_SL003B−26A1
gi|948256369−Pseudolabrys_sp__Root1462
GSMAST4A2_ASY1_ANO1_1615_1
gi|1055062764−Sphingomonas_asaccharolytica
gi|1054831220−Sphingomonas_pruni
gi|1055075009−Sphingomonas_mali
0.1
Bacteria
SAG

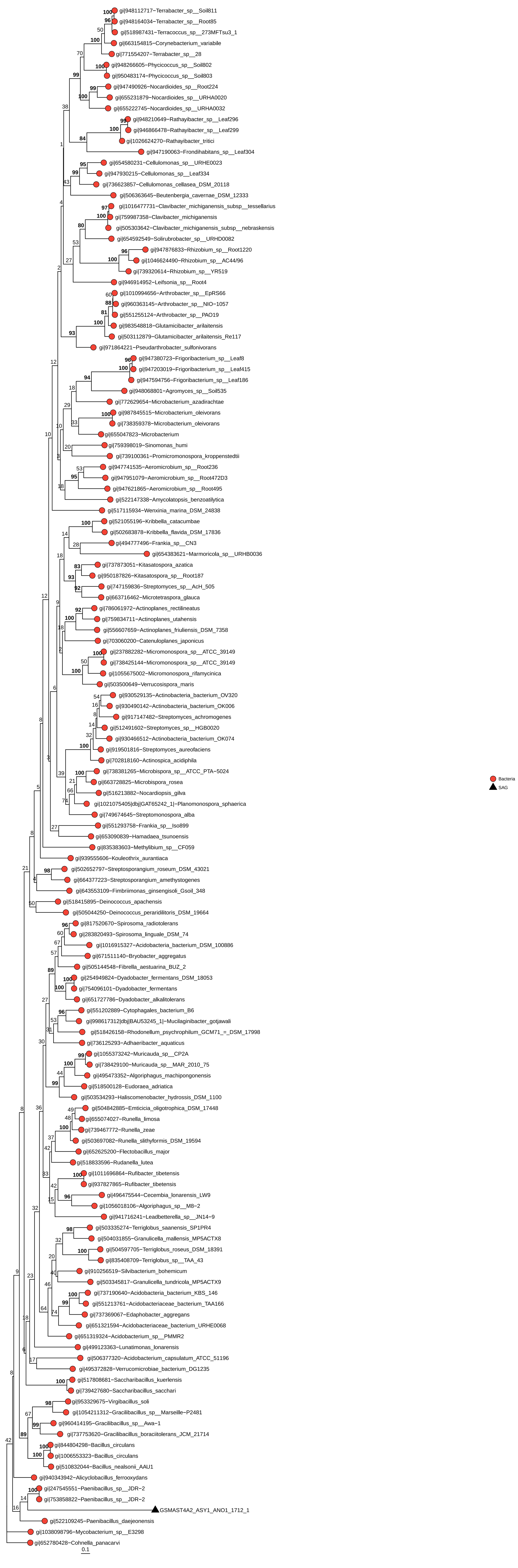
gi|948112717~Terrabacter_sp__Soil811
gi|948164034~Terrabacter_sp__Root85
gi|518987431~Terracoccus_sp__273MFTsu3_1
gi|663154815~Corynebacterium_variabile
gi|771554207~Terrabacter_sp__28
gi|948266605~Phycicoccus_sp__Soil802
gi|950483174~Phycicoccus_sp__Soil803
gi|947490926~Nocardioides_sp__Root224
gi|655231879~Nocardioides_sp__URHA0020
gi|655222745~Nocardioides_sp__URHA0032
gi|948210649~Rathayibacter_sp__Leaf296
gi|946866478~Rathayibacter_sp__Leaf299
gi|1026624270~Rathayibacter_tritici
gi|947190063~Frondihabitans_sp__Leaf304
gi|654580231~Cellulomonas_sp__URHE0023
gi|947930215~Cellulomonas_sp__Leaf334
gi|736623857~Cellulomonas_cellasea_DSM_20118
gi|506363645~Beutenbergia_cavernae_DSM_12333
gi|1016477731~Clavibacter_michiganensis_subsp__tessellarius
gi|759987358~Clavibacter_michiganensis
gi|505303642~Clavibacter_michiganensis_subsp__nebraskensis
gi|654592549~Solirubrobacter_sp__URHD0082
gi|947876833~Rhizobium_sp__Root1220
gi|1046624490~Rhizobium_sp__AC44/96
gi|739320614~Rhizobium_sp__YR519
gi|946914952~Leifsonia_sp__Root4
gi|1010994656~Arthrobacter_sp__EpRS66
gi|960363145~Arthrobacter_sp__NIO~1057
gi|551255124~Arthrobacter_sp__PAO19
gi|983548818~Glutamicibacter_arilaitensis
gi|503112879~Glutamicibacter_arilaitensis_Re117
gi|971864221~Pseudarthrobacter_sulfonivorans
gi|947380723~Frigoribacterium_sp__Leaf8
gi|947203019~Frigoribacterium_sp__Leaf415
gi|947594756~Frigoribacterium_sp__Leaf186
gi|948068801~Agromyces_sp__Soil535
gi|772629654~Microbacterium_azadirachtae
gi|987845515~Microbacterium_oleivorans
gi|738359378~Microbacterium_oleivorans
gi|655047823~Microbacterium
gi|759398019~Sinomonas_humi
gi|739100361~Promicromonospora_kroppenstedtii
gi|947741535~Aeromicrobium_sp__Root236
gi|947951079~Aeromicrobium_sp__Root472D3
gi|947621865~Aeromicrobium_sp__Root495
gi|522147338~Amycolatopsis_benzoatilytica
gi|517115934~Wenxinia_marina_DSM_24838
gi|521055196~Kribbella_catacumbae
gi|502683878~Kribbella_flavida_DSM_17836
gi|494777496~Frankia_sp__CN3
gi|654383621~Marmoricola_sp__URHB0036
gi|737873051~Kitasatospora_azatica
gi|950187826~Kitasatospora_sp__Root187
gi|747159836~Streptomyces_sp__AcH_505
gi|663716462~Microtetraspora_glauca
gi|786061972~Actinoplanes_rectilineatus
gi|759834711~Actinoplanes_utahensis
gi|556607659~Actinoplanes_friuliensis_DSM_7358
gi|703060200~Catenuloplanes_japonicus
gi|237882282~Micromonospora_sp__ATCC_39149
gi|738425144~Micromonospora_sp__ATCC_39149
gi|1055675002~Micromonospora_rifamycinica
gi|503500649~Verrucosispora_maris
gi|930529135~Actinobacteria_bacterium_OV320
gi|930490142~Actinobacteria_bacterium_OK006
gi|917147482~Streptomyces_achromogenes
gi|512491602~Streptomyces_sp__HGB0020
gi|930466512~Actinobacteria_bacterium_OK074
gi|919501816~Streptomyces_aureofaciens
gi|702818160~Actinospica_acidiphila
gi|738381265~Microbispora_sp__ATCC_PTA~5024
gi|663728825~Microbispora_rosea
gi|516213882~Nocardiopsis_gilva
gi|1021075405|dbj|GAT65242_1|~Planomonospora_sphaerica
gi|749674645~Streptomonospora_alba
gi|551293758~Frankia_sp__Iso899
gi|653090839~Hamadaea_tsunoensis
gi|835383603~Methylibium_sp__CF059
gi|939555606~Kouleothrix_aurantiaca
gi|502652797~Streptosporangium_roseum_DSM_43021
gi|664377223~Streptosporangium_amethystogenes
gi|643553109~Fimbriimonas_ginsengisoli_Gsoil_348
gi|518415895~Deinococcus_apachensis
gi|505044250~Deinococcus_peraridilitoris_DSM_19664
gi|817520670~Spirosoma_radiotolerans
gi|283820493~Spirosoma_linguale_DSM_74
gi|1016915327~Acidobacteria_bacterium_DSM_100886
gi|671511140~Bryobacter_aggregatus
gi|505144548~Fibrella_aestuarina_BUZ_2
gi|254949824~Dyadobacter_fermentans_DSM_18053
gi|754096101~Dyadobacter_fermentans
gi|651727786~Dyadobacter_alkalitolerans
gi|551202889~Cytophagales_bacterium_B6
gi|998617312|dbj|BAU53245_1|~Mucilaginibacter_gotjawali
gi|518426158~Rhodonellum_psychrophilum_GCM71_=_DSM_17998
gi|736125293~Adhaeribacter_aquaticus
gi|1055373242~Muricauda_sp__CP2A
gi|738429100~Muricauda_sp__MAR_2010_75
gi|495473352~Algoriphagus_machipongonensis
gi|518500128~Eudoraea_adriatica
gi|503534293~Haliscomenobacter_hydrossis_DSM_1100
gi|504842885~Emticicia_oligotrophica_DSM_17448
gi|655074027~Runella_limosa
gi|739467772~Runella_zeae
gi|503697082~Runella_slithyformis_DSM_19594
gi|652625200~Flectobacillus_major
gi|518833596~Rudanella_lutea
gi|1011696864~Rufibacter_tibetensis
gi|937827865~Rufibacter_tibetensis
gi|496475544~Cecembia_lonarensis_LW9
gi|1056018106~Algoriphagus_sp__M8~2
gi|941716241~Leadbetterella_sp__JN14~9
gi|503335274~Terriglobus_saanensis_SP1PR4
gi|504031855~Granulicella_mallensis_MP5ACTX8
gi|504597705~Terriglobus_roseus_DSM_18391
gi|835408709~Terriglobus_sp__TAA_43
gi|910256519~Silvibacterium_bohemicum
gi|503345817~Granulicella_tundricola_MP5ACTX9
gi|737190640~Acidobacteria_bacterium_KBS_146
gi|551213761~Acidobacteriaceae_bacterium_TAA166
gi|737369067~Edaphobacter_aggregans
gi|651321594~Acidobacteriaceae_bacterium_URHE0068
gi|651319324~Acidobacterium_sp__PMMR2
gi|499123363~Lunatimonas_lonarensis
gi|506377320~Acidobacterium_capsulatum_ATCC_51196
gi|495372828~Verrucomicrobiae_bacterium_DG1235
gi|517808681~Saccharibacillus_kuerlensis
gi|739427680~Saccharibacillus_sacchari
gi|953329675~Virgibacillus_soli
gi|1054211312~Gracilibacillus_sp__Marseille~P2481
gi|960414195~Gracilibacillus_sp__Awa~1
gi|737753620~Gracilibacillus_boraciitolerans_JCM_21714
gi|844804298~Bacillus_circulans
gi|1006553323~Bacillus_circulans
gi|510832044~Bacillus_nealsonii_AAU1
gi|940343942~Alicyclobacillus_ferrooxydans
gi|247545551~Paenibacillus_sp__JDR~2
gi|753858822~Paenibacillus_sp__JDR~2
GSMAST4A2_ASY1_ANO1_1712_1
gi|522109245~Paenibacillus_daejeonensis
gi|1038098796~Mycobacterium_sp__E3298
gi|652780428~Cohnella_panacarvi
0.1
Bacteria
SAG

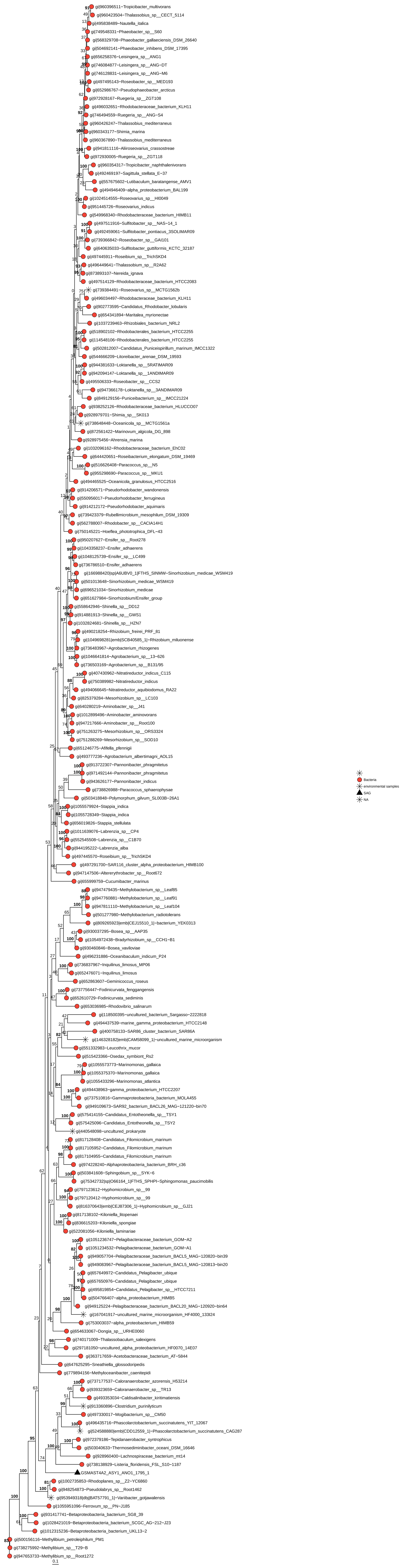
gi|960396511~Tropicibacter_multivorans
gi|960423504~Thalassobius_sp__CECT_5114
gi|495838489~Nautella_italica
gi|749548331~Phaeobacter_sp__S60
gi|568329708~Phaeobacter_gallaeciensis_DSM_26640
gi|504692141~Phaeobacter_inhibens_DSM_17395
gi|656258376~Leisingera_sp__ANG1
gi|746084877~Leisingera_sp__ANG-DT
gi|746128831~Leisingera_sp__ANG-M6
gi|497495143~Roseobacter_sp__MED193
gi|652986767~Pseudophaeobacter_arcticus
gi|972928167~Ruegeria_sp__ZGT108
gi|496032651~Rhodobacteraceae_bacterium_KLH11
gi|746494559~Ruegeria_sp__ANG-S4
gi|960426247~Thalassobius_mediterraneus
gi|960343177~Shimia_marina
gi|960367890~Thalassobius_mediterraneus
gi|941811116~Aliiroseovarius_crassostreae
gi|972930005~Ruegeria_sp__ZGT118
gi|960354317~Tropicibacter_naphthalenivorans
gi|492469197~Sagittula_stellata_E-37
gi|557675602~Lutibaculum_baratangense_AMV1
gi|494946409~alpha_proteobacterium_BAL199
gi|1024514555~Roseovarius_sp__HI0049
gi|951445726~Roseovarius_indicus
gi|549968340~Rhodobacteraceae_bacterium_HIMB11
gi|497511916~Sulfitobacter_sp__NAS-14_1
gi|492459061~Sulfitobacter_pontiacus_3SOLIMAR09
gi|739366842~Roseobacter_sp__GAI101
gi|640635033~Sulfitobacter_guttiformis_KCTC_32187
gi|497445911~Roseibium_sp__TrichSKD4
gi|496449641~Thalassobium_sp__R2A62
gi|873893107~Nereida_ignava
gi|497514129~Rhodobacteraceae_bacterium_HTCC2083
gi|739384491~Roseovarius_sp__MCTG1562b
gi|496034497~Rhodobacteraceae_bacterium_KLH11
gi|902773595~Candidatus_Rhodobacter_lobularis
gi|654341894~Maritalea_myrionectae
gi|1037239463~Rhizobiales_bacterium_NRL2
gi|518902102~Rhodobacterales_bacterium_HTCC2255
gi|114548106~Rhodobacterales_bacterium_HTCC2255
gi|502812007~Candidatus_Puniceispirillum_marinum_IMCC1322
gi|544666209~Litoreibacter_arenae_DSM_19593
gi|944381633~Loktanella_sp__5RATIMAR09
gi|942094147~Loktanella_sp__1ANDIMAR09
gi|495506333~Roseobacter_sp__CCS2
gi|947366178~Loktanella_sp__3ANDIMAR09
gi|849129156~Puniceibacterium_sp__IMCC21224
gi|938252126~Rhodobacteraceae_bacterium_HLUCCO07
gi|928979701~Shimia_sp__SK013
gi|738648448~Oceanicola_sp__MCTG1561a
gi|872561422~Marinovum_algicola_DG_898
gi|928975456~Ahrensia_marina
gi|1032096162~Rhodobacteraceae_bacterium_EhC02
gi|644420651~Roseibacterium_elongatum_DSM_19469
gi|516626408~Paracoccus_sp__N5
gi|955298690~Paracoccus_sp__MKU1
gi|494465525~Oceanicola_granulosus_HTCC2516
gi|914206571~Pseudorhodobacter_wandonensis
gi|550956017~Pseudorhodobacter_ferrugineus
gi|914212172~Pseudorhodobacter_aquimaris
gi|739423379~Rubellimicrobium_mesophilum_DSM_19309
gi|562788007~Rhodobacter_sp__CACIA14H1
gi|750145221~Hoeflea_phototrophica_DFL-43
gi|950207627~Ensifer_sp__Root278
gi|1043358237~Ensifer_adhaerens
gi|1048125739~Ensifer_sp__LC499
gi|736786510~Ensifer_adhaerens
gi|166988420|sp|A6UBV0_1|FTHS_SINMW~Sinorhizobium_medicae_WSM419
gi|501013648~Sinorhizobium_medicae_WSM419
gi|696521034~Sinorhizobium_medicae
gi|651627984~Sinorhizobium/Ensifer_group
gi|558642946~Shinella_sp__DD12
gi|914881913~Shinella_sp__GWS1
gi|1032824681~Shinella_sp__HZN7
gi|490218254~Rhizobium_freirei_PRF_81
gi|1049698281|emb|SCB40585_1|~Rhizobium_miluonense
gi|736483967~Agrobacterium_rhizogenes
gi|1046641814~Agrobacterium_sp__13-626
gi|736503169~Agrobacterium_sp__B131/95
gi|407430962~Nitratireductor_indicus_C115
gi|750389982~Nitratireductor_indicus
gi|494066645~Nitratireductor_aquibiodomus_RA22
gi|825379284~Mesorhizobium_sp__LC103
gi|640280219~Aminobacter_sp__J41
gi|1012899496~Aminobacter_aminovorans
gi|947217666~Aminobacter_sp__Root100
gi|751263275~Mesorhizobium_sp__ORS3324
gi|751288269~Mesorhizobium_sp__SOD10
gi|651246775~Afifella_pfennigii
gi|493777236~Agrobacterium_albertimagni_AOL15
gi|913722307~Pannonibacter_phragmitetus
gi|971492144~Pannonibacter_phragmitetus
gi|943626177~Pannonibacter_indicus
gi|738826988~Paracoccus_sphaerophysae
gi|503418848~Polymorphum_gilvum_SL003B-26A1
gi|1055579924~Stappia_indica
gi|1055728349~Stappia_indica
gi|656019826~Stappia_stellulata
gi|1011639076~Labrenzia_sp__CP4
gi|552545508~Labrenzia_sp__C1B70
gi|944195222~Labrenzia_alba
gi|497445570~Roseibium_sp__TrichSKD4
gi|497291700~SAR116_cluster_alpha_proteobacterium_HIMB100
gi|947147506~Altererythrobacter_sp__Root672
gi|655999759~Cucumibacter_marinus
gi|947479435~Methylobacterium_sp__Leaf85
gi|947760881~Methylobacterium_sp__Leaf91
gi|947811110~Methylobacterium_sp__Leaf104
gi|501277980~Methylobacterium_radiotolerans
gi|809265923|emb|CEJ15510_1|~bacterium_YEK0313
gi|930037295~Bosea_sp__AAP35
gi|1054972438~Bradyrhizobium_sp__CCH1-B1
gi|930460846~Bosea_vaviloviae
gi|496231886~Oceanibaculum_indicum_P24
gi|736837967~Inquilinus_limosus_MP06
gi|652476071~Inquilinus_limosus
gi|652863607~Geminicoccus_roseus
gi|737756447~Fodinicurvata_fenggangensis
gi|652610729~Fodinicurvata_sediminis
gi|653036985~Rhodovibrio_salinarum
gi|118500395~uncultured_bacterium_Sargasso~2222818
gi|494437539~marine_gamma_proteobacterium_HTCC2148
gi|400758133~SAR86_cluster_bacterium_SAR86A
gi|146328182|emb|CAM58099_1|~uncultured_marine_microorganism
gi|551332983~Leucothrix_mucor
gi|515423366~Osedax_symbiont_Rs2
gi|1055373773~Marinomonas_gallaica
gi|1055375370~Marinomonas_gallaica
gi|1055433296~Marinomonas_atlantica
gi|494438963~gamma_proteobacterium_HTCC2207
gi|737510816~Gammaproteobacteria_bacterium_MOLA455
gi|949109673~SAR92_bacterium_BACL26_MAG-121220-bin70
gi|575414155~Candidatus_Entotheonella_sp__TSY1
gi|575425096~Candidatus_Entotheonella_sp__TSY2
gi|440548098~uncultured_prokaryote
gi|817128408~Candidatus_Filomicrobium_marinum
gi|817105952~Candidatus_Filomicrobium_marinum
gi|817104955~Candidatus_Filomicrobium_marinum
gi|974228240~Alphaproteobacteria_bacterium_BRH_c36
gi|503841608~Sphingobium_sp__SYK-6
gi|75342732|sp|Q66164_1|FTHS_SPHPI~Sphingomonas_paucimobilis
gi|797123612~Hyphomicrobium_sp__99
gi|797120412~Hyphomicrobium_sp__99
gi|816370643|emb|CEJ87306_1|~Hyphomicrobium_sp__GJ21
gi|817138102~Kiloniella_litopenaei
gi|836615203~Kiloniella_spongiae
gi|522081056~Kiloniella_laminariae
gi|1051236747~Pelagibacteraceae_bacterium_GOM-A2
gi|1051234532~Pelagibacteraceae_bacterium_GOM-A1
gi|949057704~Pelagibacteraceae_bacterium_BACL5_MAG-120820-bin39
gi|949083967~Pelagibacteraceae_bacterium_BACL5_MAG-120813-bin20
gi|657649972~Candidatus_Pelagibacter_ubique
gi|657650976~Candidatus_Pelagibacter_ubique
gi|495819854~Candidatus_Pelagibacter_sp__HTCC7211
gi|504766407~alpha_proteobacterium_HIMB5
gi|949125224~Pelagibacteraceae_bacterium_BACL20_MAG-120920-bin64
gi|167041917~uncultured_marine_microorganism_HF4000_133I24
gi|753003037~alpha_proteobacterium_HIMB59
gi|654633067~Dongia_sp__URHE0060
gi|740171009~Thalassobaculum_salexigens
gi|297181050~uncultured_alpha_proteobacterium_HF0070_14E07
gi|363717659~Acetobacteraceae_bacterium_AT-5844
gi|647625295~Sneathiella_glossodoripedis
gi|779894156~Methyloceanibacter_caenitepidi
gi|737177537~Caloranaerobacter_azorensis_H53214
gi|939323659~Caloranaerobacter_sp__TR13
gi|493353034~Caldisalinibacter_kiritimatiensis
gi|913360896~Clostridium_purinilyticum
gi|497330017~Mogibacterium_sp__CM50
gi|496435716~Phascolarctobacterium_succinatutens_YIT_12067
gi|524588880|emb|CDD12559_1|~Phascolarctobacterium_succinatutens_CAG:287
gi|923792196~Tepidanaerobacter_syntrophicus
gi|503040633~Thermosediminibacter_oceani_DSM_16646
gi|928960400~Lachnospiraceae_bacterium_mt14
gi|738138929~Listeria_floridensis_FSL_S10-1187
GSMAST4A2_ASY1_ANO1_1795_1
gi|1002735853~Rhodoplanes_sp__Z2-YC6860
gi|948254873~Pseudolabrys_sp__Root1462
gi|953949318|dbj|BAT57791_1|~Variibacter_gotjawalensis
gi|1055951096~Ferrovum_sp__PN-J185
gi|931417741~Betaproteobacteria_bacterium_SG8_39
gi|1028421019~Betaproteobacteria_bacterium_SCGC_AG-212-J23
gi|1012315236~Betaproteobacteria_bacterium_UKL13-2
gi|500156116~Methylibium_petroleiphilum_PM1
gi|738275992~Methylibium_sp__T29-B
gi|947653733~Methylibium_sp__Root1272
0.1
Bacteria
environmental samples
SAG
NA

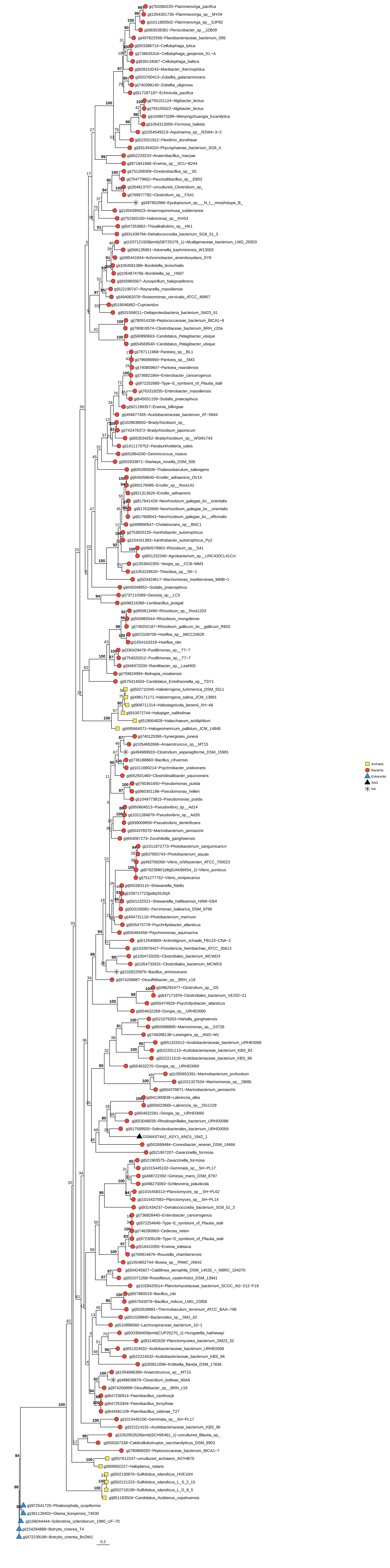
Archaea
Bacteria
Eukaryota
SAG
NA
0.2

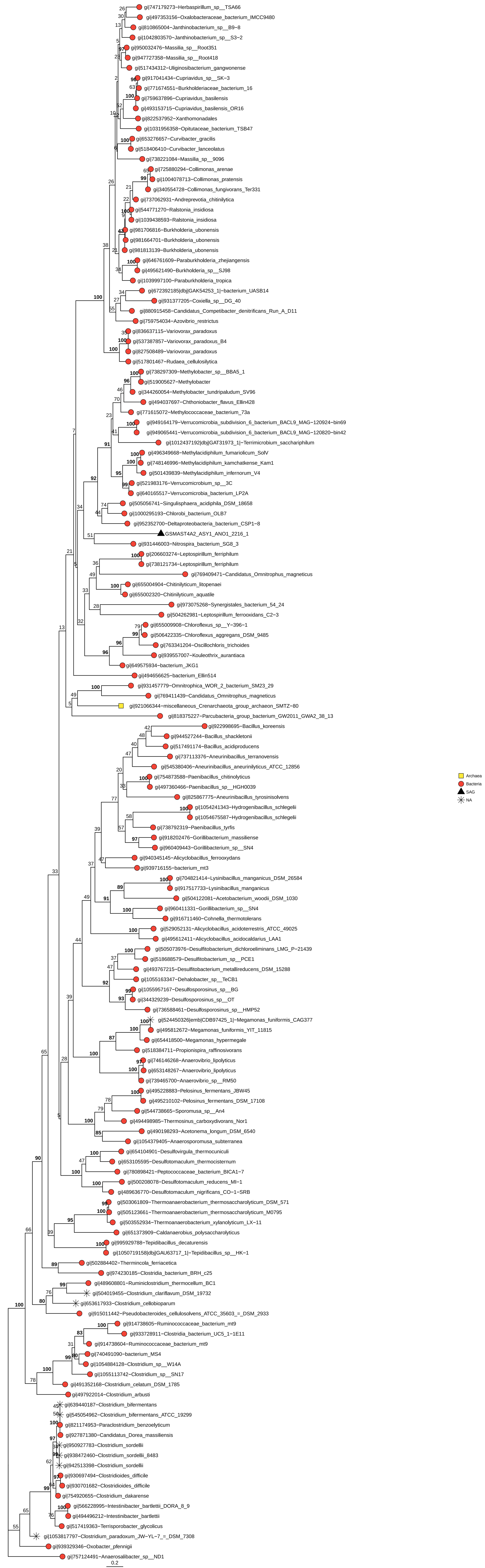
gi|747179273~Herbaspirillum_sp__TSA66
gi|497353156~Oxalobacteraceae_bacterium_IMCC9480
gi|810865004~Janthinobacterium_sp__B9-8
gi|1042803570~Janthinobacterium_sp__S3-2
gi|950032476~Massilia_sp__Root351
gi|947727358~Massilia_sp__Root418
gi|517434312~Uliginosibacterium_gangwonense
gi|917041434~Cupriavidus_sp__SK-3
gi|771674551~Burkholderiaceae_bacterium_16
gi|759637896~Cupriavidus_basilensis
gi|493153715~Cupriavidus_basilensis_OR16
gi|822537952~Xanthomonadales
gi|1031956358~Opitutaceae_bacterium_TSB47
gi|653276657~Curvibacter_gracilis
gi|518406410~Curvibacter_lanceolatus
gi|738221084~Massilia_sp__9096
gi|725880294~Collimonas_arenae
gi|1004078713~Collimonas_pratensis
gi|340554728~Collimonas_fungivorans_Ter331
gi|737062931~Andreprevotia_chitinilytica
gi|544771270~Ralstonia_insidiosa
gi|1039438593~Ralstonia_insidiosa
gi|981706816~Burkholderia_ubonensis
gi|981664701~Burkholderia_ubonensis
gi|981813139~Burkholderia_ubonensis
gi|646761609~Paraburkholderia_zhejiangensis
gi|495621490~Burkholderia_sp__SJ98
gi|1039997100~Paraburkholderia_tropica
gi|672392185|dbj|GAK54253_1|~bacterium_UASB14
gi|931377205~Coxiella_sp__DG_40
gi|880915458~Candidatus_Competibacter_denitrificans_Run_A_D11
gi|759754034~Azovibrio_restrictus
gi|836637115~Variovorax_paradoxus
gi|537387857~Variovorax_paradoxus_B4
gi|827508489~Variovorax_paradoxus
gi|517801467~Rudaea_cellulosilytica
gi|738297309~Methylobacter_sp__BBA5_1
gi|519005627~Methylobacter
gi|344260054~Methylobacter_tundripaludum_SV96
gi|494037697~Chthoniobacter_flavus_Ellin428
gi|771615072~Methylococcaceae_bacterium_73a
gi|949164179~Verrucomicrobia_subdivision_6_bacterium_BACL9_MAG-120924_bin69
gi|949065441~Verrucomicrobia_subdivision_6_bacterium_BACL9_MAG-120820_bin42
gi|1012437192|dbj|GAT31973_1|~Terrimicrobium_sacchariphilum
gi|496349668~Methylacidiphilum_fumariolicum_SolV
gi|748146996~Methylacidiphilum_kamchatkense_Kam1
gi|501439839~Methylacidiphilum_infernorum_V4
gi|521983176~Verrucomicrobium_sp__3C
gi|640165517~Verrucomicrobia_bacterium_LP2A
gi|505056741~Singulisphaera_acidiphila_DSM_18658
gi|1000295193~Chlorobi_bacterium_OLB7
gi|952352700~Deltaproteobacteria_bacterium_CSP1-8
GSMAST4A2_ASY1_ANO1_2216_1
gi|931446003~Nitrospira_bacterium_SG8_3
gi|206603274~Leptospirillum_ferriphilum
gi|738121734~Leptospirillum_ferriphilum
gi|769409471~Candidatus_Omnitrophus_magneticus
gi|655004904~Chitinilyticum_litopenaei
gi|655002320~Chitinilyticum_aquatile
gi|973075268~Synergistales_bacterium_54_24
gi|504262981~Leptospirillum_ferrooxidans_C2-3
gi|655009908~Chloroflexus_sp__Y-396-1
gi|506422335~Chloroflexus_aggregans_DSM_9485
gi|763341204~Oscillochloris_trichoides
gi|939557007~Kouleothrix_aurantiaca
gi|649575934~bacterium_JKG1
gi|494656625~bacterium_Ellin514
gi|931457779~Omnitrophica_WOR_2_bacterium_SM23_29
gi|769411439~Candidatus_Omnitrophus_magneticus
gi|921066344~miscellaneous_Crenarchaeota_group_archaeon_SMTZ-80
gi|818375227~Parcubacteria_group_bacterium_GW2011_GWA2_38_13
gi|922998695~Bacillus_koreensis
gi|944527244~Bacillus_shackletonii
gi|517491174~Bacillus_acidiproducens
gi|737113376~Aneurinibacillus_terranovensis
gi|545380406~Aneurinibacillus_aneurinilyticus_ATCC_12856
gi|754873588~Paenibacillus_chitinolyticus
gi|497360466~Paenibacillus_sp__HGH0039
gi|825867775~Aneurinibacillus_tyrosinisolvens
gi|1054241343~Hydrogenibacillus_schlegelii
gi|1054675587~Hydrogenibacillus_schlegelii
gi|738792319~Paenibacillus_tyrfis
gi|918202476~Gorillibacterium_massiliense
gi|960409443~Gorillibacterium_sp__SN4
gi|940345145~Alicyclobacillus_ferrooxydans
gi|939716155~bacterium_mt3
gi|704821414~Lysinibacillus_manganicus_DSM_26584
gi|917517733~Lysinibacillus_manganicus
gi|504122081~Acetobacterium_woodii_DSM_1030
gi|960411331~Gorillibacterium_sp__SN4
gi|916711460~Cohnella_thermotolerans
gi|529052131~Alicyclobacillus_acidoterrestris_ATCC_49025
gi|495612411~Alicyclobacillus_acidocaldarius_LAA1
gi|505073976~Desulfitobacterium_dichloroeliminans_LMG_P-21439
gi|518688579~Desulfitobacterium_sp__PCE1
gi|493767215~Desulfitobacterium_metallireducens_DSM_15288
gi|1055163347~Dehalobacter_sp__TeCB1
gi|1055957167~Desulfosporosinus_sp__BG
gi|344329239~Desulfosporosinus_sp__OT
gi|736588461~Desulfosporosinus_sp__HMP52
gi|524450326|emb|CDB97425_1|~Megamonas_funiformis_CAG377
gi|495812672~Megamonas_funiformis_YIT_11815
gi|654418500~Megamonas_hypermegale
gi|518384711~Propionispira_raffinosivorans
gi|746146268~Anaerovibrio_lipolyticus
gi|653148267~Anaerovibrio_lipolyticus
gi|739465700~Anaerovibrio_sp__RM50
gi|495228883~Pelosinus_fermentans_JBW45
gi|495210102~Pelosinus_fermentans_DSM_17108
gi|544738665~Sporomusa_sp__An4
gi|494498985~Thermosinus_carboxydivorans_Nor1
gi|490198293~Acetonema_longum_DSM_6540
gi|1054379405~Anaerosporomusa_subterranea
gi|654104901~Desulfovirgula_thermocuniculi
gi|653105595~Desulfotomaculum_thermocisternum
gi|780898421~Peptococcaceae_bacterium_BICA1-7
gi|500208070~Desulfotomaculum_reducens_MI-1
gi|489636770~Desulfotomaculum_nigrificans_CO-1-SRB
gi|503061809~Thermoanaerobacterium_thermosaccharolyticum_DSM_571
gi|505123661~Thermoanaerobacterium_thermosaccharolyticum_M0795
gi|503552934~Thermoanaerobacterium_xylanolyticum_LX-11
gi|651373909~Caldanaerobius_polysaccharolyticus
gi|995929788~Tepidibacillus_decaturensis
gi|1050719158|dbj|GAU63717_1|~Tepidibacillus_sp__HK-1
gi|502884402~Thermincola_ferriacetica
gi|974230185~Clostridia_bacterium_BRH_c25
gi|489608801~Ruminiclostridium_thermocellum_BC1
gi|504019455~Clostridium_clariflavum_DSM_19732
gi|653617933~Clostridium_cellobioparum
gi|915011442~Pseudobacteroides_cellulosolvens_ATCC_35603_=_DSM_2933
gi|914738605~Ruminococcaceae_bacterium_mt9
gi|933728911~Clostridia_bacterium_UC5_1-1E11
gi|914738604~Ruminococcaceae_bacterium_mt9
gi|740491090~bacterium_MS4
gi|1054884128~Clostridium_sp__W14A
gi|1055113742~Clostridium_sp__SN17
gi|491352168~Clostridium_celatum_DSM_1785
gi|497922014~Clostridium_arbusti
gi|639440187~Clostridium_bifermentans
gi|545054962~Clostridium_bifermentans_ATCC_19299
gi|821174953~Paraclostridium_benzoelyticum
gi|927871380~Candidatus_Dorea_massiliensis
gi|950927783~Clostridium_sordellii
gi|938472460~Clostridium_sordellii_8483
gi|942513398~Clostridium_sordellii
gi|930697494~Clostridioides_difficile
gi|930701682~Clostridioides_difficile
gi|754920655~Clostridium_dakarense
gi|566228995~Intestinibacter_bartlettii_DORA_8_9
gi|494496212~Intestinibacter_bartlettii
gi|517419363~Terrisporobacter_glycolicus
gi|1053817797~Clostridium_paradoxum_JW-YL-7_=_DSM_7308
gi|939329346~Oxobacter_pfennigii
gi|757124491~Anaerosalibacter_sp__ND1
0.2
Archaea
Bacteria
SAG
NA

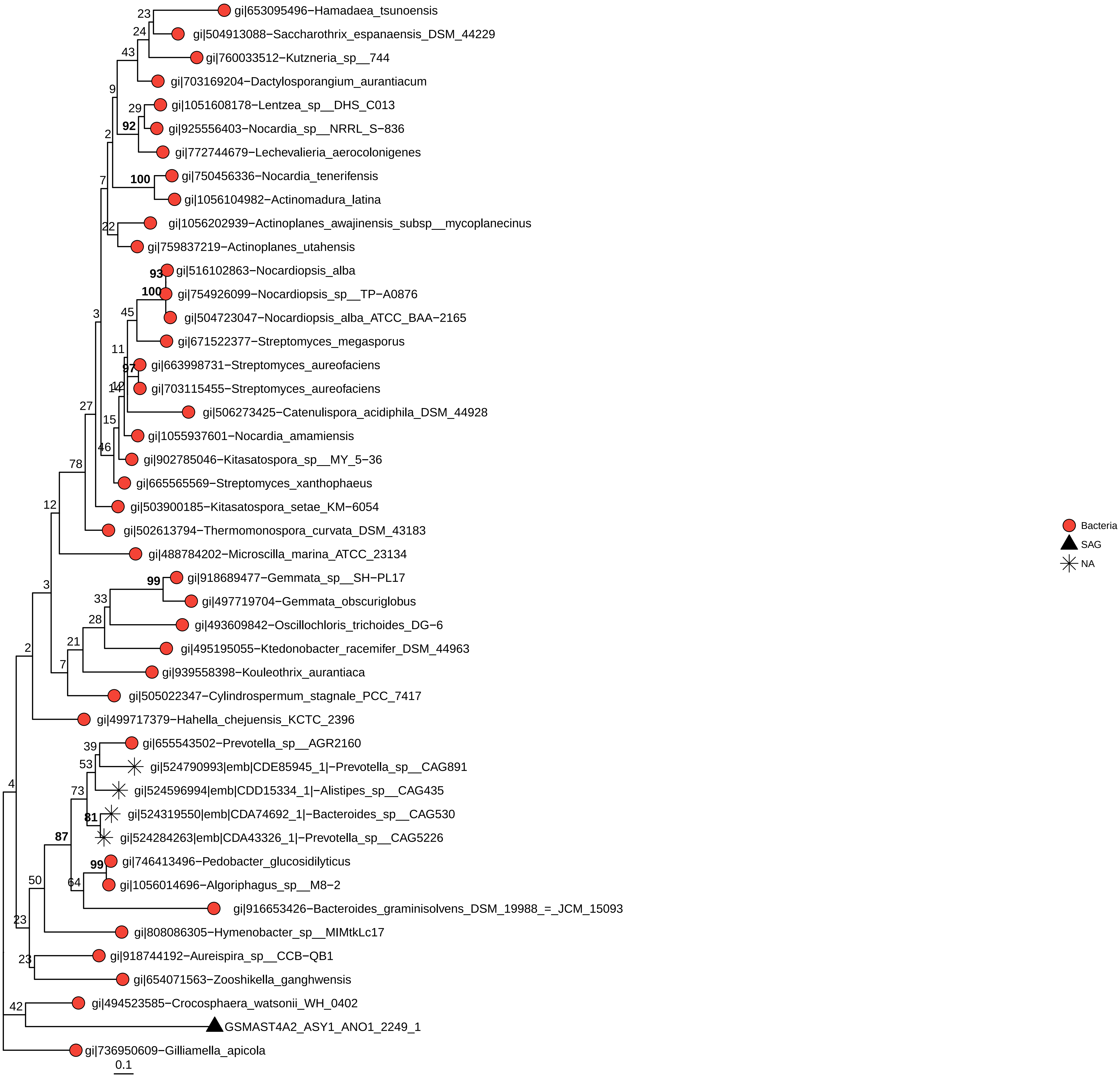
gi|653095496−Hamadaea_tsunoensis
gi|504913088−Saccharothrix_espanaensis_DSM_44229
gi|760033512−Kutzneria_sp__744
gi|703169204−Dactylosporangium_aurantiacum
gi|1051608178−Lentzea_sp__DHS_C013
gi|925556403−Nocardia_sp__NRRL_S−836
gi|772744679−Lechevalieria_aerocolonigenes
gi|750456336−Nocardia_tenerifensis
gi|1056104982−Actinomadura_latina
gi|1056202939−Actinoplanes_awajinensis_subsp__mycoplanecinus
gi|759837219−Actinoplanes_utahensis
gi|516102863−Nocardiopsis_alba
gi|754926099−Nocardiopsis_sp__TP−A0876
gi|504723047−Nocardiopsis_alba_ATCC_BAA−2165
gi|671522377−Streptomyces_megasporus
gi|663998731−Streptomyces_aureofaciens
gi|703115455−Streptomyces_aureofaciens
gi|506273425−Catenulispora_acidiphila_DSM_44928
gi|1055937601−Nocardia_amamiensis
gi|902785046−Kitasatospora_sp__MY_5−36
gi|665565569−Streptomyces_xanthophaeus
gi|503900185−Kitasatospora_setae_KM−6054
gi|502613794−Thermomonospora_curvata_DSM_43183
gi|488784202−Microscilla_marina_ATCC_23134
gi|918689477−Gemmata_sp__SH−PL17
gi|497719704−Gemmata_obscuriglobus
gi|493609842−Oscillochloris_trichoides_DG−6
gi|495195055−Ktedonobacter_racemifer_DSM_44963
gi|939558398−Kouleothrix_aurantiaca
gi|505022347−Cylindrospermum_stagnale_PCC_7417
gi|499717379−Hahella_chejuensis_KCTC_2396
gi|655543502−Prevotella_sp__AGR2160
gi|524790993|emb|CDE85945_1|−Prevotella_sp__CAG891
gi|524596994|emb|CDD15334_1|−Alistipes_sp__CAG435
gi|524319550|emb|CDA74692_1|−Bacteroides_sp__CAG530
gi|524284263|emb|CDA43326_1|−Prevotella_sp__CAG5226
gi|746413496−Pedobacter_glucosidilyticus
gi|1056014696−Algoriphagus_sp__M8−2
gi|916653426−Bacteroides_graminisolvens_DSM_19988_=_JCM_15093
gi|808086305−Hymenobacter_sp__MIMtkLc17
gi|918744192−Aureispira_sp__CCB−QB1
gi|654071563−Zooshikella_ganghwensis
gi|494523585−Crocosphaera_watsonii_WH_0402
GSMAST4A2_ASY1_ANO1_2249_1
gi|736950609−Gilliamella_apicola
23
24
43
9
29
92
2
7
100
22
93
100
45
11
97
14
12
15
46
27
3
78
12
3
99
33
28
21
7
2
39
53
73
81
87
99
64
50
4
23
23
42
0.1
Bacteria
SAG
NA

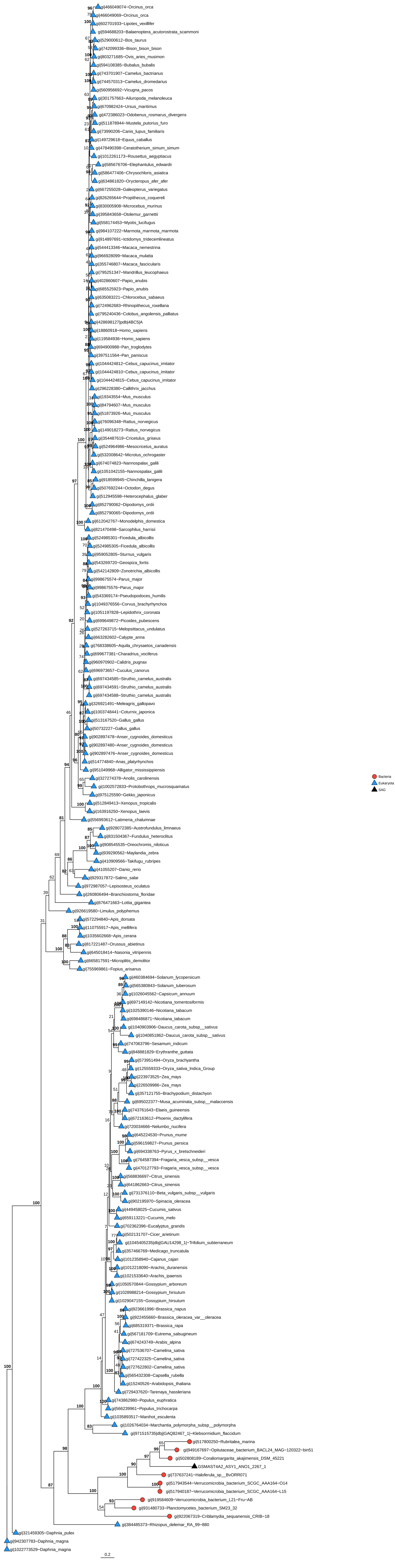
gi|466049074~Orcinus_orca
gi|466049069~Orcinus_orca
gi|602701933~Lipotes_vexillifer
gi|594688203~Balaenoptera_acutorostrata_scammoni
gi|529000612~Bos_taurus
gi|742099336~Bison_bison_bison
gi|803271685~Ovis_aries_musimon
gi|594108385~Bubalus_bubalis
gi|743701907~Camelus_bactrianus
gi|744570313~Camelus_dromedarius
gi|560956692~Vicugna_pacos
gi|301757663~Ailuropoda_melanoleuca
gi|670892424~Ursus_maritimus
gi|472386023~Odobenus_rosmarus_divergens
gi|511878944~Mustela_putorius_furo
gi|73990206~Canis_lupus_familiaris
gi|149729618~Equus_caballus
gi|478490398~Ceratotherium_simum_simum
gi|1012261173~Rousettus_aegyptiacus
gi|585676706~Elephantulus_edwardii
gi|586477406~Chrysochloris_asiatica
gi|634861820~Orycteropus_afer_afer
gi|667255028~Galeopterus_variegatus
gi|826265644~Propithecus_coquereli
gi|830005908~Microcebus_murinus
gi|395843658~Otolemur_garnettii
gi|558174453~Myotis_lucifugus
gi|984107222~Marmota_marmota_marmota
gi|914897691~Ictidomys_tridecemlineatus
gi|544413346~Macaca_nemestrina
gi|966928099~Macaca_mulatta
gi|355746807~Macaca_fascicularis
gi|795251347~Mandrillus_leucophaeus
gi|402860607~Papio_anubis
gi|685525923~Papio_anubis
gi|635083221~Chlorocebus_sabaeus
gi|724962683~Rhinopithecus_roxellana
gi|795240436~Colobus_angolensis_palliatus
gi|428698127|pdb|4BC5|A
gi|18860918~Homo_sapiens
gi|119584936~Homo_sapiens
gi|694900988~Pan_troglodytes
gi|397511564~Pan_paniscus
gi|1044424812~Cebus_capucinus_imitator
gi|1044424810~Cebus_capucinus_imitator
gi|1044424815~Cebus_capucinus_imitator
gi|296228380~Callithrix_jacchus
gi|19343554~Mus_musculus
gi|84794607~Mus_musculus
gi|51873926~Mus_musculus
gi|76096348~Rattus_norvegicus
gi|149018273~Rattus_norvegicus
gi|354487619~Cricetulus_griseus
gi|524964986~Mesocricetus_auratus
gi|532008642~Microtus_ochrogaster
gi|674074823~Nannospalax_galili
gi|1051042155~Nannospalax_galili
gi|918599945~Chinchilla_lanigera
gi|507692244~Octodon_degus
gi|512945598~Heterocephalus_glaber
gi|852790062~Dipodomys_ordii
gi|852790065~Dipodomys_ordii
gi|12042767~Monodelphis_domestica
gi|821470498~Sarcophilus_harrisii
gi|524985301~Ficedula_albicollis
gi|524985305~Ficedula_albicollis
gi|959052805~Sturnus_vulgaris
gi|543269720~Geospiza_fortis
gi|542142809~Zonotrichia_albicollis
gi|998675574~Parus_major
gi|998675576~Parus_major
gi|543369174~Pseudopodoces_humilis
gi|1049376556~Corvus_brachyrhynchos
gi|1051197828~Lepidothrix_coronata
gi|699649872~Picoides_pubescens
gi|527263715~Melopsittacus_undulatus
gi|663282602~Calypte_anna
gi|768338605~Aquila_chrysaetos_canadensis
gi|699677381~Charadrius_vociferus
gi|960970902~Calidris_pugnax
gi|696973657~Cuculus_canorus
gi|697434585~Struthio_camelus_australis
gi|697434591~Struthio_camelus_australis
gi|697434588~Struthio_camelus_australis
gi|326921491~Meleagris_gallopavo
gi|1003748441~Coturnix_japonica
gi|513167520~Gallus_gallus
gi|50732227~Gallus_gallus
gi|902897478~Anser_cygnoides_domesticus
gi|902897480~Anser_cygnoides_domesticus
gi|902897476~Anser_cygnoides_domesticus
gi|514774840~Anas_platyrhynchos
gi|951049968~Alligator_mississippiensis
gi|327274378~Anolis_carolinensis
gi|1002572833~Protobothrops_mucrosquamatus
gi|975125590~Gekko_japonicus
gi|512849413~Xenopus_tropicalis
gi|163916250~Xenopus_laevis
gi|556993612~Latimeria_chalumnae
gi|928072385~Austrofundulus_limnaeus
gi|831504367~Fundulus_heteroclitus
gi|908545535~Oreochromis_niloticus
gi|939290562~Maylandia_zebra
gi|410909566~Takifugu_rubripes
gi|41055207~Danio_rerio
gi|929317872~Salmo_salar
gi|972987057~Lepisosteus_oculatus
gi|260806494~Branchiostoma_floridae
gi|676471663~Lottia_gigantea
gi|926619580~Limulus_polyphemus
gi|572294840~Apis_dorsata
gi|110755917~Apis_mellifera
gi|1035602668~Apis_cerana
gi|817221487~Orussus_abietinus
gi|645018414~Nasonia_vitripennis
gi|665817591~Microplitis_demolitor
gi|755969861~Fopius_arisanus
gi|460384694~Solanum_lycopersicum
gi|565380843~Solanum_tuberosum
gi|1026045562~Capsicum_annuum
gi|697149142~Nicotiana_tomentosiformis
gi|1025390146~Nicotiana_tabacum
gi|698486871~Nicotiana_tabacum
gi|1040903906~Daucus_carota_subsp__sativus
gi|1040851862~Daucus_carota_subsp__sativus
gi|747063796~Sesamum_indicum
gi|848881829~Erythranthe_guttata
gi|573951494~Oryza_brachyantha
gi|125559333~Oryza_sativa_Indica_Group
gi|223973525~Zea_mays
gi|226509986~Zea_mays
gi|357121755~Brachypodium_distachyon
gi|695022377~Musa_acuminata_subsp__malaccensis
gi|743761643~Elaeis_guineensis
gi|672163612~Phoenix_dactylifera
gi|720034666~Nelumbo_nucifera
gi|645224530~Prunus_mume
gi|596159827~Prunus_persica
gi|694338763~Pyrus_x_bretschneideri
gi|764587394~Fragaria_vesca_subsp__vesca
gi|470127793~Fragaria_vesca_subsp__vesca
gi|568836697~Citrus_sinensis
gi|641862663~Citrus_sinensis
gi|731376110~Beta_vulgaris_subsp__vulgaris
gi|902195970~Spinacia_oleracea
gi|449458025~Cucumis_sativus
gi|659113221~Cucumis_melo
gi|702362396~Eucalyptus_grandis
gi|502131707~Cicer_arietinum
gi|1045405235|dbj|GAU14298_1|~Trifolium_subterraneum
gi|357466769~Medicago_truncatula
gi|1012358940~Cajanus_cajan
gi|1012218090~Arachis_duranensis
gi|1021533640~Arachis_ipaensis
gi|1050570844~Gossypium_arboreum
gi|1028988214~Gossypium_hirsutum
gi|1029047155~Gossypium_hirsutum
gi|923661996~Brassica_napus
gi|922455660~Brassica_oleracea_var__oleracea
gi|685319371~Brassica_rapa
gi|567181709~Eutrema_salsugineum
gi|674243749~Arabis_alpina
gi|727536707~Camelina_sativa
gi|727422325~Camelina_sativa
gi|727622802~Camelina_sativa
gi|565432308~Capsella_rubella
gi|15240526~Arabidopsis_thaliana
gi|729437620~Tarenaya_hassleriana
gi|743862980~Populus_euphratica
gi|566239961~Populus_trichocarpa
gi|1035893517~Manihot_esculenta
gi|1026764034~Marchantia_polymorpha_subsp__polymorpha
gi|971515735|dbj|GAQ82467_1|~Klebsormidium_flaccidum
gi|517800250~Rubritalea_marina
gi|949167697~Opitutaceae_bacterium_BACL24_MAG-120322-bin51
gi|502808189~Coraliomargarita_akajimensis_DSM_45221
GSMAST4A2_ASY1_ANO1_2267_1
gi|737637241~Haloferula_sp__BvORR071
gi|517943544~Verrucomicrobia_bacterium_SCGC_AAA164-O14
gi|517940187~Verrucomicrobia_bacterium_SCGC_AAA164-L15
gi|919584609~Verrucomicrobia_bacterium_L21-Fru-AB
gi|931480733~Planctomycetes_bacterium_SM23_32
gi|922067319~Cribblamydia_sequanensis_CRIB-18
gi|384485373~Rhizopus_delemar_RA_99-880
gi|321459305~Daphnia_pulex
gi|942307783~Daphnia_magna
gi|1022773529~Daphnia_magna
0.2
Bacteria
Eukaryota
SAG

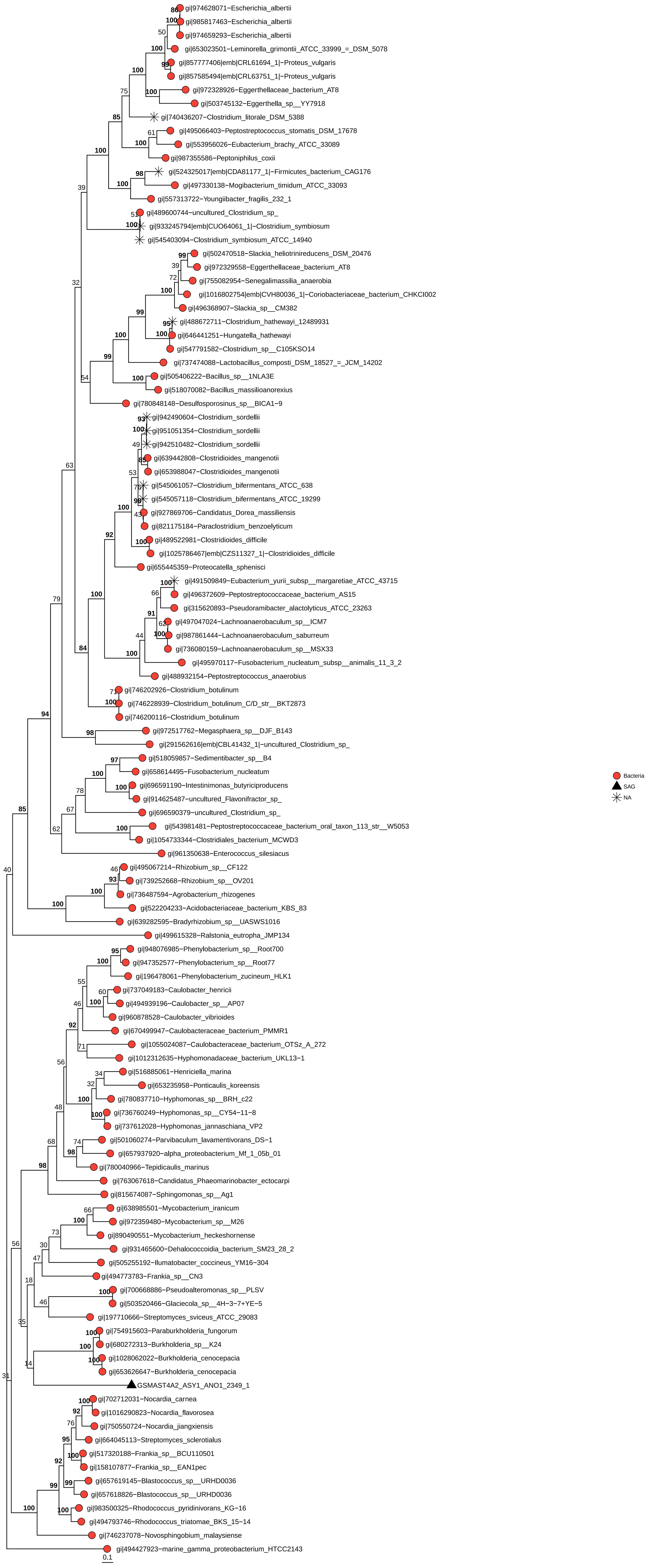
gi|974628071−Escherichia_albertii
gi|985817463−Escherichia_albertii
gi|974659293−Escherichia_albertii
gi|653023501−Leminorella_grimontii_ATCC_33999_=_DSM_5078
gi|857777406|emb|CRL61694_1|−Proteus_vulgaris
gi|857585494|emb|CRL63751_1|−Proteus_vulgaris
gi|972328926−Eggerthellaceae_bacterium_AT8
gi|503745132−Eggerthella_sp__YY7918
gi|740436207−Clostridium_litorale_DSM_5388
gi|495066403−Peptostreptococcus_stomatis_DSM_17678
gi|553956026−Eubacterium_brachy_ATCC_33089
gi|987355586−Peptoniphilus_coxii
gi|524325017|emb|CDA81177_1|−Firmicutes_bacterium_CAG176
gi|497330138−Mogibacterium_timidum_ATCC_33093
gi|557313722−Youngiibacter_fragilis_232_1
gi|489600744−uncultured_Clostridium_sp_
gi|933245794|emb|CUO64061_1|−Clostridium_symbiosum
gi|545403094−Clostridium_symbiosum_ATCC_14940
gi|502470518−Slackia_heliotrinireducens_DSM_20476
gi|972329558−Eggerthellaceae_bacterium_AT8
gi|755082954−Senegalimassilia_anaerobia
gi|1016802754|emb|CVH80036_1|−Coriobacteriaceae_bacterium_CHKCI002
gi|496368907−Slackia_sp__CM382
gi|488672711−Clostridium_hathewayi_12489931
gi|646441251−Hungatella_hathewayi
gi|547791582−Clostridium_sp__C105KSO14
gi|737474088−Lactobacillus_composti_DSM_18527_=_JCM_14202
gi|505406222−Bacillus_sp__1NLA3E
gi|518070082−Bacillus_massilioanorexius
gi|780848148−Desulfosporosinus_sp__BICA1−9
gi|942490604−Clostridium_sordellii
gi|951051354−Clostridium_sordellii
gi|942510482−Clostridium_sordellii
gi|639442808−Clostridioides_mangenotii
gi|653988047−Clostridioides_mangenotii
gi|545061057−Clostridium_bifermentans_ATCC_638
gi|545057118−Clostridium_bifermentans_ATCC_19299
gi|927869706−Candidatus_Dorea_massiliensis
gi|821175184−Paraclostridium_benzoelyticum
gi|489522981−Clostridioides_difficile
gi|1025786467|emb|CZS11327_1|−Clostridioides_difficile
gi|655445359−Proteocatella_sphenisci
gi|491509849−Eubacterium_yurii_subsp__margaretiae_ATCC_43715
gi|496372609−Peptostreptococcaceae_bacterium_AS15
gi|315620893−Pseudoramibacter_alactolyticus_ATCC_23263
gi|497047024−Lachnoanaerobaculum_sp__ICM7
gi|987861444−Lachnoanaerobaculum_saburreum
gi|736080159−Lachnoanaerobaculum_sp__MSX33
gi|495970117−Fusobacterium_nucleatum_subsp__animalis_11_3_2
gi|488932154−Peptostreptococcus_anaerobius
gi|746202926−Clostridium_botulinum
gi|746228939−Clostridium_botulinum_C/D_str__BKT2873
gi|746200116−Clostridium_botulinum
gi|972517762−Megasphaera_sp__DJF_B143
gi|291562616|emb|CBL41432_1|−uncultured_Clostridium_sp_
gi|518059857−Sedimentibacter_sp__B4
gi|658614495−Fusobacterium_nucleatum
gi|696591190−Intestinimonas_butyriciproducens
gi|914625487−uncultured_Flavonifractor_sp_
gi|696590379−uncultured_Clostridium_sp_
gi|543981481−Peptostreptococcaceae_bacterium_oral_taxon_113_str__W5053
gi|1054733344−Clostridiales_bacterium_MCWD3
gi|961350638−Enterococcus_silesiacus
gi|495067214−Rhizobium_sp__CF122
gi|739252668−Rhizobium_sp__OV201
gi|736487594−Agrobacterium_rhizogenes
gi|522204233−Acidobacteriaceae_bacterium_KBS_83
gi|639282595−Bradyrhizobium_sp__UASWS1016
gi|499615328−Ralstonia_eutropha_JMP134
gi|948076985−Phenylobacterium_sp__Root700
gi|947352577−Phenylobacterium_sp__Root77
gi|196478061−Phenylobacterium_zucineum_HLK1
gi|737049183−Caulobacter_henricii
gi|494939196−Caulobacter_sp__AP07
gi|960878528−Caulobacter_vibrioides
gi|670499947−Caulobacteraceae_bacterium_PMMR1
gi|1055024087−Caulobacteraceae_bacterium_OTSz_A_272
gi|1012312635−Hyphomonadaceae_bacterium_UKL13−1
gi|516885061−Henriciella_marina
gi|653235958−Ponticaulis_koreensis
gi|780837710−Hyphomonas_sp__BRH_c22
gi|736760249−Hyphomonas_sp__CY54−11−8
gi|737612028−Hyphomonas_jannaschiana_VP2
gi|501060274−Parvibaculum_lavamentivorans_DS−1
gi|657937920−alpha_proteobacterium_Mf_1_05b_01
gi|780040966−Tepidicaulis_marinus
gi|763067618−Candidatus_Phaeomarinobacter_ectocarpi
gi|815674087−Sphingomonas_sp__Ag1
gi|638985501−Mycobacterium_iranicum
gi|972359480−Mycobacterium_sp__M26
gi|890490551−Mycobacterium_heckeshornense
gi|931465600−Dehalococcoidia_bacterium_SM23_28_2
gi|505255192−Ilumatobacter_coccineus_YM16−304
gi|494773783−Frankia_sp__CN3
gi|700668886−Pseudoalteromonas_sp__PLSV
gi|503520466−Glaciecola_sp__4H−3−7+YE−5
gi|197710666−Streptomyces_sviceus_ATCC_29083
gi|754915603−Paraburkholderia_fungorum
gi|680272313−Burkholderia_sp__K24
gi|1028062022−Burkholderia_cenocepacia
gi|653626647−Burkholderia_cenocepacia
GSMAST4A2_ASY1_ANO1_2349_1
gi|702712031−Nocardia_carnea
gi|1016290823−Nocardia_flavorosea
gi|750550724−Nocardia_jiangxiensis
gi|664045113−Streptomyces_sclerotialus
gi|517320188−Frankia_sp__BCU110501
gi|158107877−Frankia_sp__EAN1pec
gi|657619145−Blastococcus_sp__URHD0036
gi|657618826−Blastococcus_sp__URHD0036
gi|983500325−Rhodococcus_pyridinivorans_KG−16
gi|494793746−Rhodococcus_triatomae_BKS_15−14
gi|746237078−Novosphingobium_malaysiense
gi|494427923−marine_gamma_proteobacterium_HTCC2143
0.1
Bacteria
SAG
NA

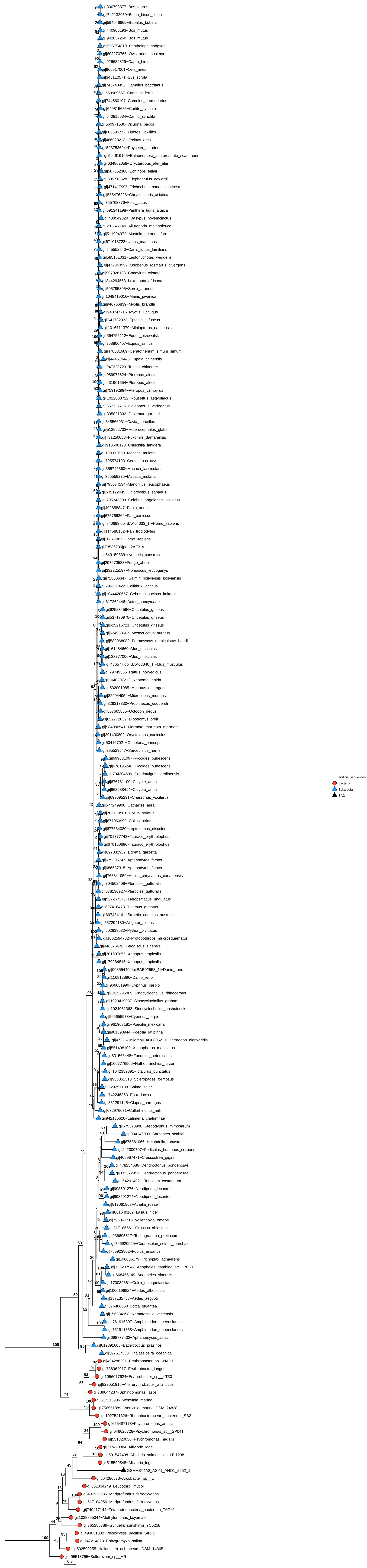
artificial sequences
Bacteria
Eukaryota
SAG
0.3

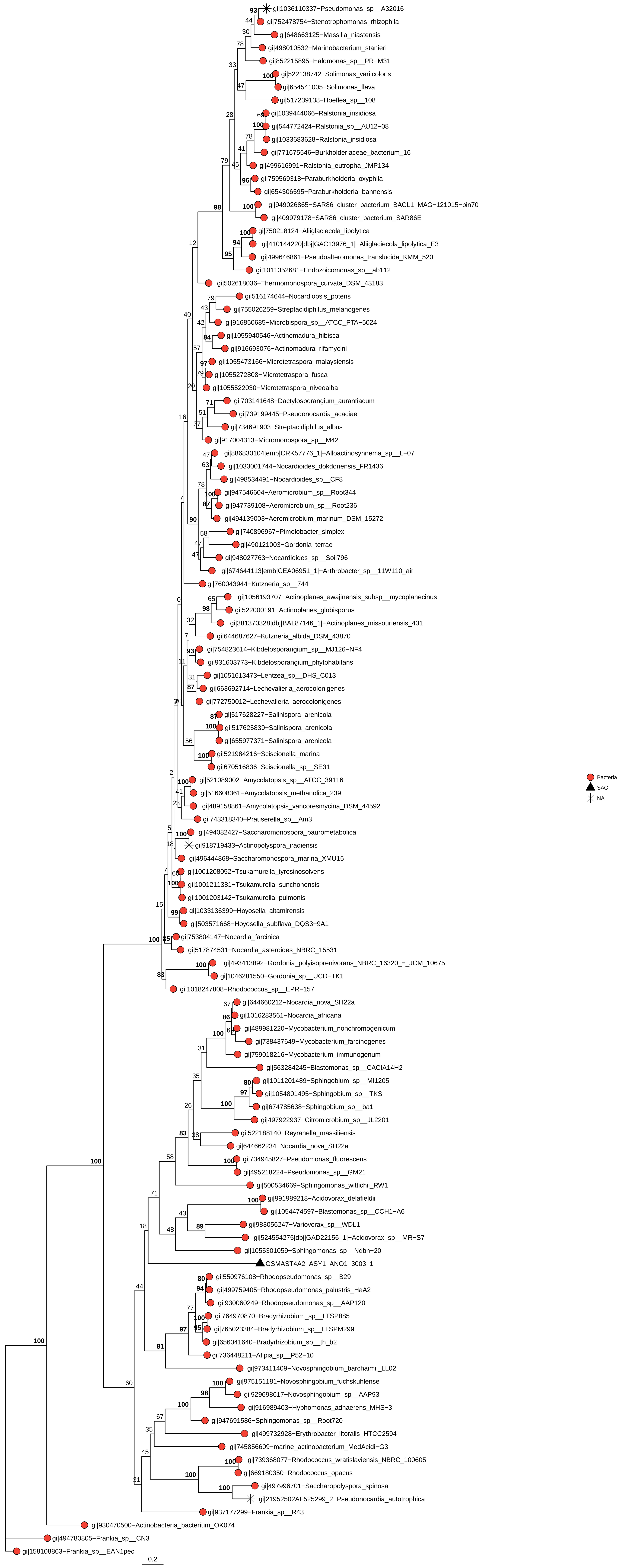

gi|1036110337−Pseudomonas_sp__A32016
gi|752478754−Stenotrophomonas_rhizophila
gi|648663125−Massilia_niastensis
gi|498010532−Marinobacterium_stanieri
gi|852215895−Halomonas_sp__PR−M31
gi|522138742−Solimonas_variicoloris
gi|654541005−Solimonas_flava
gi|517239138−Hoeflea_sp__108
gi|1039444066−Ralstonia_insidiosa
gi|544772424−Ralstonia_sp__AU12−08
gi|1033683628−Ralstonia_insidiosa
gi|771675546−Burkholderiaceae_bacterium_16
gi|499616991−Ralstonia_eutropha_JMP134
gi|759569318−Paraburkholderia_oxyphila
gi|654306595−Paraburkholderia_bannensis
gi|949026865−SAR86_cluster_bacterium_BACL1_MAG−121015−bin70
gi|409979178−SAR86_cluster_bacterium_SAR86E
gi|750218124−Aliiglaciecola_lipolytica
gi|410144220|dbj|GAC13976_1|−Aliiglaciecola_lipolytica_E3
gi|499646861−Pseudoalteromonas_translucida_KMM_520
gi|1011352681−Endozoicomonas_sp__ab112
gi|502618036−Thermomonospora_curvata_DSM_43183
gi|516174644−Nocardiopsis_potens
gi|755026259−Streptacidiphilus_melanogenes
gi|916850685−Microbispora_sp__ATCC_PTA−5024
gi|1055940546−Actinomadura_hibisca
gi|916693076−Actinomadura_rifamycini
gi|1055473166−Microtetraspora_malaysiensis
gi|1055272808−Microtetraspora_fusca
gi|1055522030−Microtetraspora_niveoalba
gi|703141648−Dactylosporangium_aurantiacum
gi|739199445−Pseudonocardia_acaciae
gi|734691903−Streptacidiphilus_albus
gi|917004313−Micromonospora_sp__M42
gi|886830104|emb|CRK57776_1|−Alloactinosynnema_sp__L−07
gi|1033001744−Nocardioides_dokdonensis_FR1436
gi|498534491−Nocardioides_sp__CF8
gi|947546604−Aeromicrobium_sp__Root344
gi|947739108−Aeromicrobium_sp__Root236
gi|494139003−Aeromicrobium_marinum_DSM_15272
gi|740896967−Pimelobacter_simplex
gi|490121003−Gordonia_terrae
gi|948027763−Nocardioides_sp__Soil796
gi|674644113|emb|CEA06951_1|−Arthrobacter_sp__11W110_air
gi|760043944−Kutzneria_sp__744
gi|1056193707−Actinoplanes_awajinensis_subsp__mycoplanecinus
gi|522000191−Actinoplanes_globisporus
gi|381370328|dbj|BAL87146_1|−Actinoplanes_missouriensis_431
gi|644687627−Kutzneria_albida_DSM_43870
gi|754823614−Kibdelosporangium_sp__MJ126−NF4
gi|931603773−Kibdelosporangium_phytohabitans
gi|1051613473−Lentzea_sp__DHS_C013
gi|663692714−Lechevalieria_aerocolonigenes
gi|772750012−Lechevalieria_aerocolonigenes
gi|517628227−Salinispora_arenicola
gi|517625839−Salinispora_arenicola
gi|655977371−Salinispora_arenicola
gi|521984216−Sciscionella_marina
gi|670516836−Sciscionella_sp__SE31
gi|521089002−Amycolatopsis_sp__ATCC_39116
gi|516608361−Amycolatopsis_methanolica_239
gi|489158861−Amycolatopsis_vancoresmycina_DSM_44592
gi|743318340−Prauserella_sp__Am3
gi|494082427−Saccharomonospora_paurometabolica
gi|918719433−Actinopolyspora_iraqiensis
gi|496444868−Saccharomonospora_marina_XMU15
gi|1001208052−Tsukamurella_tyrosinosolvens
gi|1001211381−Tsukamurella_sunchonensis
gi|1001203142−Tsukamurella_pulmonis
gi|1033136399−Hoyosella_altamirensis
gi|503571668−Hoyosella_subflava_DQS3−9A1
gi|753804147−Nocardia_farcinica
gi|517874531−Nocardia_asteroides_NBRC_15531
gi|493413892−Gordonia_polyisoprenivorans_NBRC_16320_=_JCM_10675
gi|1046281550−Gordonia_sp__UCD−TK1
gi|1018247808−Rhodococcus_sp__EPR−157
gi|644660212−Nocardia_nova_SH22a
gi|1016283561−Nocardia_africana
gi|489981220−Mycobacterium_nonchromogenicum
gi|738437649−Mycobacterium_farcinogenes
gi|759018216−Mycobacterium_immunogenum
gi|563284245−Blastomonas_sp__CACIA14H2
gi|1011201489−Sphingobium_sp__MI1205
gi|1054801495−Sphingobium_sp__TKS
gi|674785638−Sphingobium_sp__ba1
gi|497922937−Citromicrobium_sp__JL2201
gi|522188140−Reyranella_massiliensis
gi|644662234−Nocardia_nova_SH22a
gi|734945827−Pseudomonas_fluorescens
gi|495218224−Pseudomonas_sp__GM21
gi|500534669−Sphingomonas_wittichii_RW1
gi|991989218−Acidovorax_delafieldii
gi|1054474597−Blastomonas_sp__CCH1−A6
gi|983056247−Variovorax_sp__WDL1
gi|524554275|dbj|GAD22156_1|−Acidovorax_sp__MR−S7
gi|1055301059−Sphingomonas_sp__Ndbn−20
GSMAST4A2_ASY1_ANO1_3003_1
gi|550976108−Rhodopseudomonas_sp__B29
gi|499759405−Rhodopseudomonas_palustris_HaA2
gi|930060249−Rhodopseudomonas_sp__AAP120
gi|764970870−Bradyrhizobium_sp__LTSP885
gi|765023384−Bradyrhizobium_sp__LTSPM299
gi|656041640−Bradyrhizobium_sp__th_b2
gi|736448211−Afipia_sp__P52−10
gi|973411409−Novosphingobium_barchaimii_LL02
gi|975151181−Novosphingobium_fuchskuhlense
gi|929698617−Novosphingobium_sp__AAP93
gi|916989403−Hyphomonas_adhaerens_MHS−3
gi|947691586−Sphingomonas_sp__Root720
gi|499732928−Erythrobacter_litoralis_HTCC2594
gi|745856609−marine_actinobacterium_MedAcidi−G3
gi|739368077−Rhodococcus_wratislaviensis_NBRC_100605
gi|669180350−Rhodococcus_opacus
gi|497996701−Saccharopolyspora_spinosa
gi|21952502AF525299_2−Pseudonocardia_autotrophica
gi|937177299−Frankia_sp__R43
gi|930470500−Actinobacteria_bacterium_OK074
gi|494780805−Frankia_sp__CN3
gi|158108863−Frankia_sp__EAN1pec
0.2
Bacteria
SAG
NA

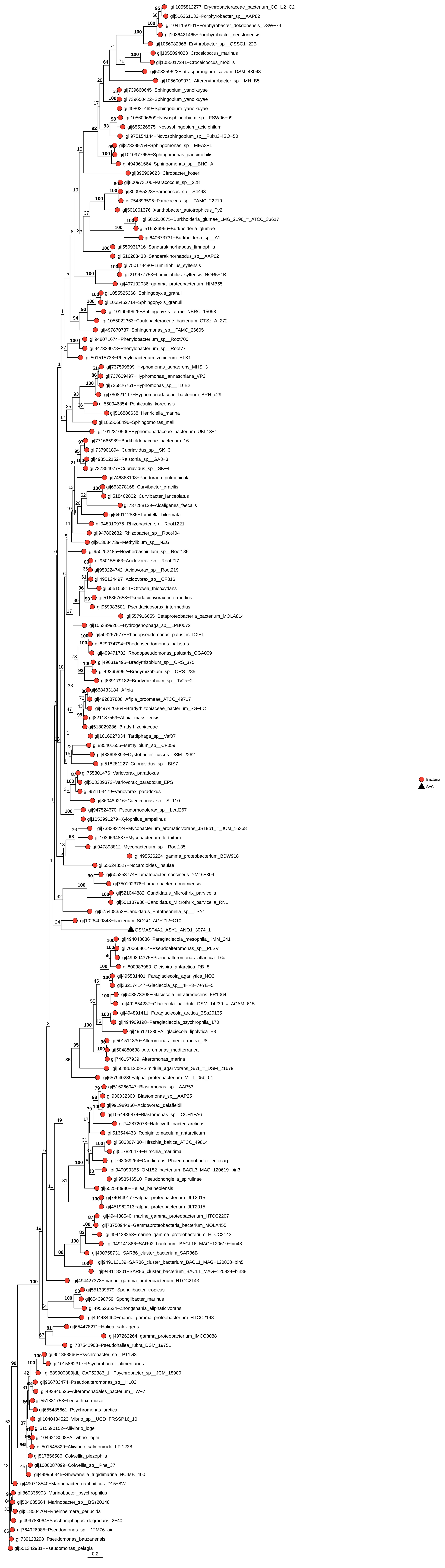
gi|1055812277~Erythrobacteraceae_bacterium_CCH12~C2
gi|516261133~Porphyrobacter_sp__AAP82
gi|1041150101~Porphyrobacter_dokdonensis_DSW~74
gi|1036421465~Porphyrobacter_neustonensis
gi|1056082868~Erythrobacter_sp__QSSC1~22B
gi|1055094023~Croceicoccus_marinus
gi|1055017241~Croceicoccus_mobilis
gi|503259622~Intrasporangium_calvum_DSM_43043
gi|1056009071~Altererythrobacter_sp__MH~B5
gi|739660645~Sphingobium_yanoikuyae
gi|739650422~Sphingobium_yanoikuyae
gi|498021469~Sphingobium_yanoikuyae
gi|1056096609~Novosphingobium_sp__FSW06~99
gi|655226575~Novosphingobium_acidiphilum
gi|975154144~Novosphingobium_sp__Fuku2~ISO~50
gi|873289754~Sphingomonas_sp__MEA3~1
gi|1010977655~Sphingomonas_paucimobilis
gi|494961664~Sphingomonas_sp__BHC~A
gi|895909623~Citrobacter_koseri
gi|800973106~Paracoccus_sp__228
gi|800955328~Paracoccus_sp__S4493
gi|754893595~Paracoccus_sp__PAMC_22219
gi|501061376~Xanthobacter_autotrophicus_Py2
gi|502210675~Burkholderia_glumae_LMG_2196_=_ATCC_33617
gi|516536966~Burkholderia_glumae
gi|640673731~Burkholderia_sp__A1
gi|550931716~Sandarakinorhabdus_limnophila
gi|516263433~Sandarakinorhabdus_sp__AAP62
gi|750178480~Luminiphilus_syltensis
gi|219677753~Luminiphilus_syltensis_NOR5~1B
gi|497102036~gamma_proteobacterium_HIMB55
gi|1055525368~Sphingopyxis_granuli
gi|1055452714~Sphingopyxis_granuli
gi|1016049925~Sphingopyxis_terrae_NBRC_15098
gi|1055022363~Caulobacteraceae_bacterium_OTSz_A_272
gi|497870787~Sphingomonas_sp__PAMC_26605
gi|948071674~Phenylobacterium_sp__Root700
gi|947329078~Phenylobacterium_sp__Root77
gi|501515738~Phenylobacterium_zucineum_HLK1
gi|737599599~Hyphomonas_adhaerens_MHS~3
gi|737609497~Hyphomonas_jannaschiana_VP2
gi|736826761~Hyphomonas_sp__T16B2
gi|780821117~Hyphomonadaceae_bacterium_BRH_c29
gi|550946854~Ponticaulis_koreensis
gi|516886638~Henriciella_marina
gi|1055068496~Sphingomonas_mali
gi|1012310506~Hyphomonadaceae_bacterium_UKL13~1
gi|771665989~Burkholderiaceae_bacterium_16
gi|737901894~Cupriavidus_sp__SK~3
gi|498512152~Ralstonia_sp__GA3~3
gi|737854077~Cupriavidus_sp__SK~4
gi|746368193~Pandoraea_pulmonicola
gi|653278168~Curvibacter_gracilis
gi|518402802~Curvibacter_lanceolatus
gi|737288139~Alcaligenes_faecalis
gi|640112885~Tomitella_biformata
gi|948010976~Rhizobacter_sp__Root1221
gi|947802632~Rhizobacter_sp__Root404
gi|913634739~Methylibium_sp__NZG
gi|950252485~Noviherbaspirillum_sp__Root189
gi|950155963~Acidovorax_sp__Root217
gi|950224742~Acidovorax_sp__Root219
gi|495124497~Acidovorax_sp__CF316
gi|655156811~Ottowia_thiooxydans
gi|516367658~Pseudacidovorax_intermedius
gi|969983601~Pseudacidovorax_intermedius
gi|557916655~Betaproteobacteria_bacterium_MOLA814
gi|1053899201~Hydrogenophaga_sp__LPB0072
gi|503267677~Rhodopseudomonas_palustris_DX~1
gi|829074794~Rhodopseudomonas_palustris
gi|499471782~Rhodopseudomonas_palustris_CGA009
gi|496319495~Bradyrhizobium_sp__ORS_375
gi|493659992~Bradyrhizobium_sp__ORS_285
gi|639179182~Bradyrhizobium_sp__Tv2a~2
gi|658433184~Afipia
gi|492887808~Afipia_broomeae_ATCC_49717
gi|497420364~Bradyrhizobiaceae_bacterium_SG~6C
gi|821187559~Afipia_massiliensis
gi|518029286~Bradyrhizobiaceae
gi|1016927034~Tardiphaga_sp__Vaf07
gi|835401655~Methylibium_sp__CF059
gi|488698393~Cystobacter_fuscus_DSM_2262
gi|518281227~Cupriavidus_sp__BIS7
gi|755801476~Variovorax_paradoxus
gi|503309372~Variovorax_paradoxus_EPS
gi|951103479~Variovorax_paradoxus
gi|860489216~Caenimonas_sp__SL110
gi|947524670~Pseudorhodoferax_sp__Leaf267
gi|1053991279~Xylophilus_ampelinus
gi|738392724~Mycobacterium_aromaticivorans_JS19b1_=_JCM_16368
gi|1039594837~Mycobacterium_fortuitum
gi|947898812~Mycobacterium_sp__Root135
gi|495526224~gamma_proteobacterium_BDW918
gi|655248527~Nocardioides_insulae
gi|505253774~Ilumatobacter_coccineus_YM16~304
gi|750192376~Ilumatobacter_nonamiensis
gi|521044882~Candidatus_Microthrix_parvicella
gi|501187936~Candidatus_Microthrix_parvicella_RN1
gi|575408352~Candidatus_Entotheonella_sp__TSY1
gi|1028409348~bacterium_SCGC_AG~212~C10
GSMAST4A2_ASY1_ANO1_3074_1
gi|494048686~Paraglaciecola_mesophila_KMM_241
gi|700668614~Pseudoalteromonas_sp__PLSV
gi|499894375~Pseudoalteromonas_atlantica_T6c
gi|800983980~Oleispira_antarctica_RB~8
gi|495581401~Paraglaciecola_agarilytica_NO2
gi|332174147~Glaciecola_sp__4H~3~7+YE~5
gi|503873208~Glaciecola_nitratireducens_FR1064
gi|492854237~Glaciecola_pallidula_DSM_14239_=_ACAM_615
gi|494891411~Paraglaciecola_arctica_BSs20135
gi|494909198~Paraglaciecola_psychrophila_170
gi|496121235~Aliiglaciecola_lipolytica_E3
gi|501511330~Alteromonas_mediterranea_U8
gi|504880638~Alteromonas_mediterranea
gi|746157939~Alteromonas_marina
gi|504861203~Simiduia_agarivorans_SA1_=_DSM_21679
gi|657940239~alpha_proteobacterium_Mf_1_05b_01
gi|516266947~Blastomonas_sp__AAP53
gi|930032300~Blastomonas_sp__AAP25
gi|991989150~Acidovorax_delafieldii
gi|1055485874~Blastomonas_sp__CCH1~A6
gi|742872078~Halocynthiibacter_arcticus
gi|516544433~Robiginitomaculum_antarcticum
gi|506307430~Hirschia_baltica_ATCC_49814
gi|517826474~Hirschia_maritima
gi|763069264~Candidatus_Phaeomarinobacter_ectocarpi
gi|949090355~OM182_bacterium_BACL3_MAG~120619~bin3
gi|953546510~Pseudohongiella_spirulinae
gi|652548980~Hellea_balneolensis
gi|740449177~alpha_proteobacterium_JLT2015
gi|451962013~alpha_proteobacterium_JLT2015
gi|494438540~marine_gamma_proteobacterium_HTCC2207
gi|737509449~Gammaproteobacteria_bacterium_MOLA455
gi|494433253~marine_gamma_proteobacterium_HTCC2143
gi|949141866~SAR92_bacterium_BACL16_MAG~120619~bin48
gi|400758731~SAR86_cluster_bacterium_SAR86B
gi|949113139~SAR86_cluster_bacterium_BACL1_MAG~120828~bin5
gi|949118201~SAR86_cluster_bacterium_BACL1_MAG~120924~bin88
gi|494427373~marine_gamma_proteobacterium_HTCC2143
gi|551339579~Spongiibacter_tropicus
gi|654398759~Spongiibacter_marinus
gi|495523534~Zhongshania_aliphaticivorans
gi|494434450~marine_gamma_proteobacterium_HTCC2148
gi|654478271~Haliea_salexigens
gi|497262264~gamma_proteobacterium_IMCC3088
gi|737542903~Pseudohaliea_rubra_DSM_19751
gi|951383866~Psychrobacter_sp__P11G3
gi|1015862317~Psychrobacter_alimentarius
gi|589900389|dbj|GAF52383_1|~Psychrobacter_sp__JCM_18900
gi|966783474~Pseudoalteromonas_sp__H103
gi|493846526~Alteromonadales_bacterium_TW~7
gi|551331753~Leucothrix_mucor
gi|655485661~Psychromonas_arctica
gi|1040434523~Vibrio_sp__UCD~FRSSP16_10
gi|515590152~Aliivibrio_logei
gi|1046218008~Aliivibrio_logei
gi|501545829~Aliivibrio_salmonicida_LFI1238
gi|517856586~Colwellia_piezophila
gi|1000087099~Colwellia_sp__Phe_37
gi|499956345~Shewanella_frigidimarina_NCIMB_400
gi|490718540~Marinobacter_nanhaiticus_D15~8W
gi|860336903~Marinobacter_psychrophilus
gi|504685564~Marinobacter_sp__BSs20148
gi|518504704~Rheinheimera_perlucida
gi|499788064~Saccharophagus_degradans_2~40
gi|764926985~Pseudomonas_sp__12M76_air
gi|739123298~Pseudomonas_bauzanensis
gi|551342931~Pseudomonas_pelagia
0.2
Bacteria
SAG

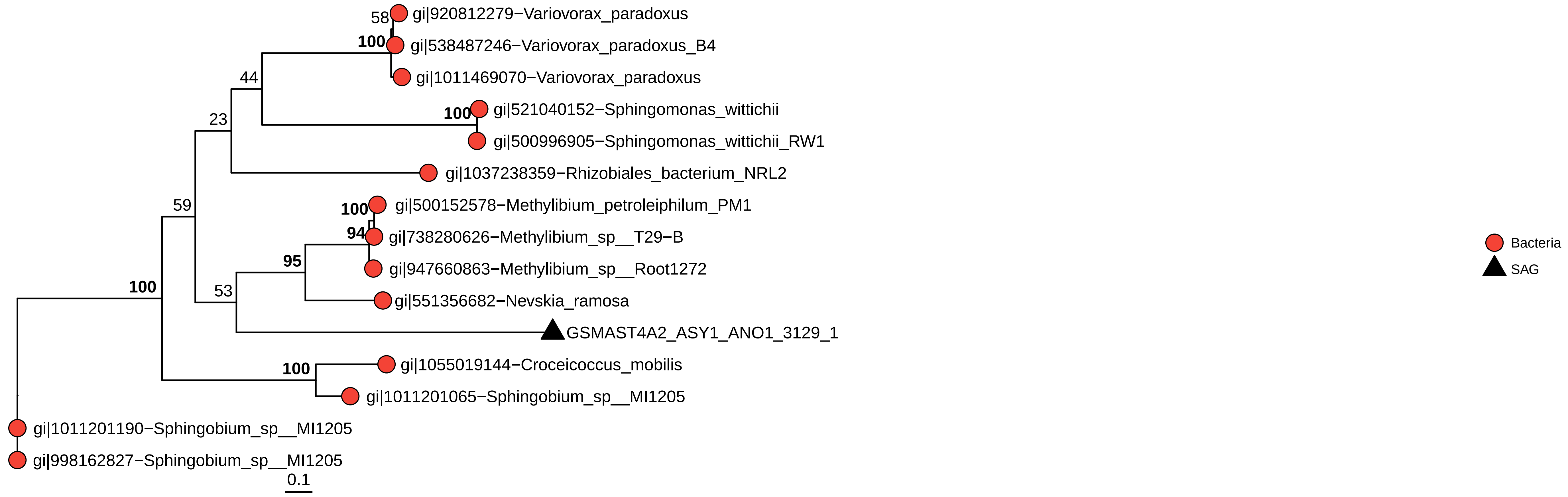
gi|920812279−Variovorax_paradoxus
gi|538487246−Variovorax_paradoxus_B4
gi|1011469070−Variovorax_paradoxus
gi|521040152−Sphingomonas_wittichii
gi|500996905−Sphingomonas_wittichii_RW1
gi|1037238359−Rhizobiales_bacterium_NRL2
gi|500152578−Methylibium_petroleiphilum_PM1
gi|738280626−Methylibium_sp__T29−B
gi|947660863−Methylibium_sp__Root1272
gi|551356682−Nevskia_ramosa
GSMAST4A2_ASY1_ANO1_3129_1
gi|1055019144−Croceicoccus_mobilis
gi|1011201065−Sphingobium_sp__MI1205
gi|1011201190−Sphingobium_sp__MI1205
gi|998162827−Sphingobium_sp__MI1205
58
100
44
23
100
59
100
94
95
100
53
100
0.1
Bacteria
SAG

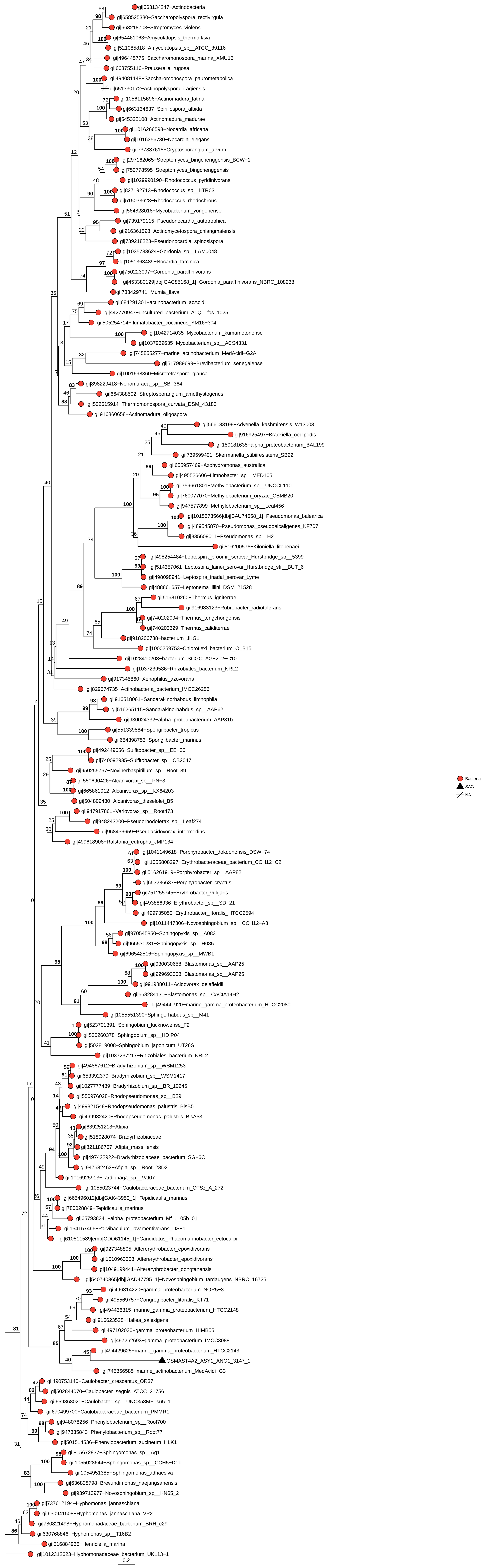

gi|663134247~Actinobacteria
gi|658525380~Saccharopolyspora_rectivirgula
gi|663218703~Streptomyces_violens
gi|654461063~Amycolatopsis_thermoflava
gi|521085818~Amycolatopsis_sp__ATCC_39116
gi|496445775~Saccharomonospora_marina_XMU15
gi|663755116~Prauserella_rugosa
gi|494081148~Saccharomonospora_paurometabolica
gi|651330172~Actinopolyspora_iraqiensis
gi|1056115696~Actinomadura_latina
gi|663134637~Spirillospora_albida
gi|545322108~Actinomadura_madurae
gi|1016266593~Nocardia_africana
gi|1016356730~Nocardia_elegans
gi|737887615~Cryptosporangium_arvum
gi|297162065~Streptomyces_bingchenggensis_BCW−1
gi|759778595~Streptomyces_bingchenggensis
gi|1029990190~Rhodococcus_pyridinivorans
gi|827192713~Rhodococcus_sp__IITR03
gi|515033628~Rhodococcus_rhodochrous
gi|564828018~Mycobacterium_yongonense
gi|739179115~Pseudonocardia_autotrophica
gi|916361598~Actinomycetospora_chiangmaiensis
gi|739218223~Pseudonocardia_spinosispora
gi|1035733624~Gordonia_sp__LAM0048
gi|1051363489~Nocardia_farcinica
gi|750223097~Gordonia_paraffinivorans
gi|453380129|dbj|GAC85168_1|~Gordonia_paraffinivorans_NBRC_108238
gi|733429741~Mumia_flava
gi|684291301~actinobacterium_acAcidi
gi|442770947~uncultured_bacterium_A1Q1_fos_1025
gi|505254714~Ilumatobacter_coccineus_YM16−304
gi|1042714035~Mycobacterium_kumamotonense
gi|1037939635~Mycobacterium_sp__ACS4331
gi|745855277~marine_actinobacterium_MedAcidi−G2A
gi|517989699~Brevibacterium_senegalense
gi|1001698360~Microtetraspora_glauca
gi|898229418~Nonomuraea_sp__SBT364
gi|664388502~Streptosporangium_amethystogenes
gi|502615914~Thermomonospora_curvata_DSM_43183
gi|916860658~Actinomadura_oligospora
gi|566133199~Advenella_kashmirensis_W13003
gi|916925497~Brackiella_oedipodis
gi|159181635~alpha_proteobacterium_BAL199
gi|739599401~Skermanella_stibiiresistens_SB22
gi|655957469~Azohydromonas_australica
gi|495526606~Limnobacter_sp__MED105
gi|759661801~Methylobacterium_sp__UNCCL110
gi|760077070~Methylobacterium_oryzae_CBMB20
gi|947577899~Methylobacterium_sp__Leaf456
gi|1015573566|dbj|BAU74658_1|~Pseudomonas_balearica
gi|489545870~Pseudomonas_pseudoalcaligenes_KF707
gi|835609011~Pseudomonas_sp__H2
gi|816200576~Kiloniella_litopenaei
gi|498254484~Leptospira_broomii_serovar_Hurstbridge_str__5399
gi|514357061~Leptospira_fainei_serovar_Hurstbridge_str__BUT_6
gi|498098941~Leptospira_inadai_serovar_Lyme
gi|488861657~Leptonema_illini_DSM_21528
gi|516810260~Thermus_igniterrae
gi|916983123~Rubrobacter_radiotolerans
gi|740202094~Thermus_tengchongensis
gi|740203329~Thermus_caliditerrae
gi|918206738~bacterium_JKG1
gi|1000259753~Chloroflexi_bacterium_OLB15
gi|1028410203~bacterium_SCGC_AG−212−C10
gi|1037239586~Rhizobiales_bacterium_NRL2
gi|917345860~Xenophilus_azovorans
gi|829574735~Actinobacteria_bacterium_IMCC26256
gi|916518061~Sandarakinorhabdus_limnophila
gi|516265115~Sandarakinorhabdus_sp__AAP62
gi|930024332~alpha_proteobacterium_AAP81b
gi|551339584~Spongiibacter_tropicus
gi|654398753~Spongiibacter_marinus
gi|492449656~Sulfitobacter_sp__EE−36
gi|740092935~Sulfitobacter_sp__CB2047
gi|950255767~Noviherbaspirillum_sp__Root189
gi|550690426~Alcanivorax_sp__PN−3
gi|665861012~Alcanivorax_sp__KX64203
gi|504809430~Alcanivorax_dieselolei_B5
gi|947917861~Variovorax_sp__Root473
gi|948243200~Pseudorhodoferax_sp__Leaf274
gi|968436659~Pseudacidovorax_intermedius
gi|499618908~Ralstonia_eutropha_JMP134
gi|1041149618~Porphyrobacter_dokdonensis_DSW−74
gi|1055808297~Erythrobacteraceae_bacterium_CCH12−C2
gi|516261919~Porphyrobacter_sp__AAP82
gi|653236637~Porphyrobacter_cryptus
gi|751255745~Erythrobacter_vulgaris
gi|493886936~Erythrobacter_sp__SD−21
gi|499735050~Erythrobacter_litoralis_HTCC2594
gi|1011447306~Novosphingobium_sp__CCH12−A3
gi|970545850~Sphingopyxis_sp__A083
gi|966531231~Sphingopyxis_sp__H085
gi|696542516~Sphingopyxis_sp__MWB1
gi|930030658~Blastomonas_sp__AAP25
gi|929693308~Blastomonas_sp__AAP25
gi|991988011~Acidovorax_delafieldii
gi|563284131~Blastomonas_sp__CACIA14H2
gi|494441920~marine_gamma_proteobacterium_HTCC2080
gi|1055551390~Sphingorhabdus_sp__M41
gi|523701391~Sphingobium_lucknowense_F2
gi|530260378~Sphingobium_sp__HDIP04
gi|502819008~Sphingobium_japonicum_UT26S
gi|1037237217~Rhizobiales_bacterium_NRL2
gi|494867612~Bradyrhizobium_sp__WSM1253
gi|653392379~Bradyrhizobium_sp__WSM1417
gi|1027777489~Bradyrhizobium_sp__BR_10245
gi|550976028~Rhodopseudomonas_sp__B29
gi|499821548~Rhodopseudomonas_palustris_BisB5
gi|499982420~Rhodopseudomonas_palustris_BisA53
gi|639251213~Afipia
gi|518028074~Bradyrhizobiaceae
gi|821186767~Afipia_massiliensis
gi|497422922~Bradyrhizobiaceae_bacterium_SG−6C
gi|947632463~Afipia_sp__Root123D2
gi|1016925913~Tardiphaga_sp__Vaf07
gi|1055023744~Caulobacteraceae_bacterium_OTSz_A_272
gi|665496012|dbj|GAK43950_1|~Tepidicaulis_marinus
gi|780028849~Tepidicaulis_marinus
gi|657938341~alpha_proteobacterium_Mf_1_05b_01
gi|154157466~Parvibaculum_lavamentivorans_DS−1
gi|610511589|emb|CDO61145_1|~Candidatus_Phaeomarinobacter_ectocarpi
gi|927348805~Altererythrobacter_epoxidivorans
gi|1010963308~Altererythrobacter_epoxidivorans
gi|1049199441~Altererythrobacter_dongtanensis
gi|540740365|dbj|GAD47795_1|~Novosphingobium_tardaugens_NBRC_16725
gi|496314220~gamma_proteobacterium_NOR5−3
gi|495569757~Congregibacter_litoralis_KT71
gi|494436315~marine_gamma_proteobacterium_HTCC2148
gi|916623528~Haliea_salexigens
gi|497102030~gamma_proteobacterium_HIMB55
gi|497262693~gamma_proteobacterium_IMCC3088
gi|494429625~marine_gamma_proteobacterium_HTCC2143
GSMAST4A2_ASY1_ANO1_3147_1
gi|745856585~marine_actinobacterium_MedAcidi−G3
gi|490753140~Caulobacter_crescentus_OR37
gi|502844070~Caulobacter_segnis_ATCC_21756
gi|659868021~Caulobacter_sp__UNC358MFTsu5_1
gi|670499700~Caulobacteraceae_bacterium_PMMR1
gi|948078256~Phenylobacterium_sp__Root700
gi|947335843~Phenylobacterium_sp__Root77
gi|501514536~Phenylobacterium_zucineum_HLK1
gi|815672837~Sphingomonas_sp__Ag1
gi|1055028644~Sphingomonas_sp__CCH5−D11
gi|1054951385~Sphingomonas_adhaesiva
gi|636828798~Brevundimonas_naejangsanensis
gi|939713977~Novosphingobium_sp__KN65_2
gi|737612194~Hyphomonas_jannaschiana
gi|630941508~Hyphomonas_jannaschiana_VP2
gi|780821498~Hyphomonadaceae_bacterium_BRH_c29
gi|630768846~Hyphomonas_sp__T16B2
gi|516884936~Henriciella_marina
gi|1012312623~Hyphomonadaceae_bacterium_UKL13−1
0.2
Bacteria
SAG
NA

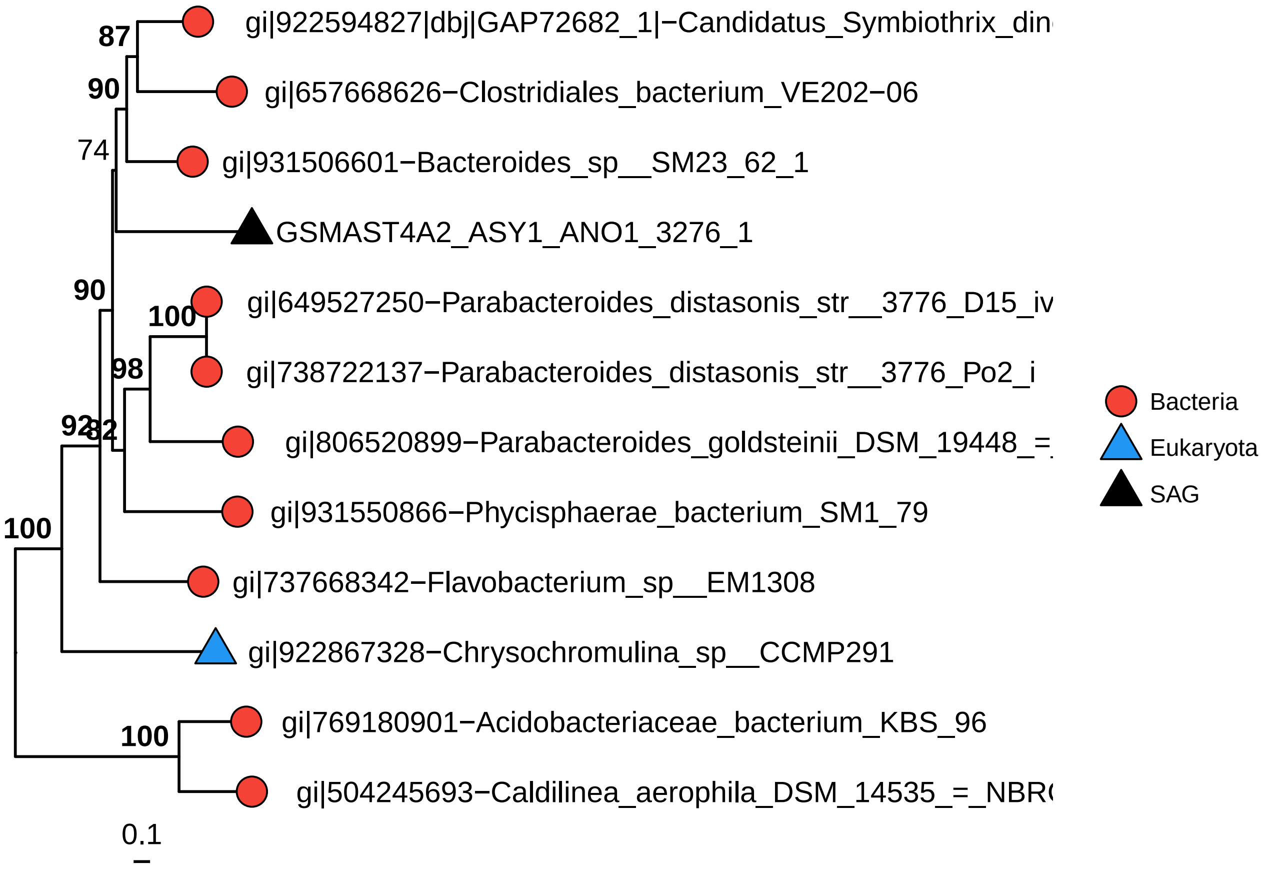

87
90
74
90
100
98
92
82
100
100
gi|922594827|dbj|GAP72682_1|−Candidatus_Symbiothrix_din
gi|657668626−Clostridiales_bacterium_VE202−06
gi|931506601−Bacteroides_sp__SM23_62_1
GSMAST4A2_ASY1_ANO1_3276_1
gi|649527250−Parabacteroides_distasonis_str__3776_D15_iv
gi|738722137−Parabacteroides_distasonis_str__3776_Po2_i
gi|806520899−Parabacteroides_goldsteinii_DSM_19448_=
gi|931550866−Phycisphaerae_bacterium_SM1_79
gi|737668342−Flavobacterium_sp__EM1308
gi|922867328−Chrysochromulina_sp__CCMP291
gi|769180901−Acidobacteriaceae_bacterium_KBS_96
gi|504245693−Caldilinea_aerophila_DSM_14535_=_NBR
0.1
Bacteria
Eukaryota
SAG

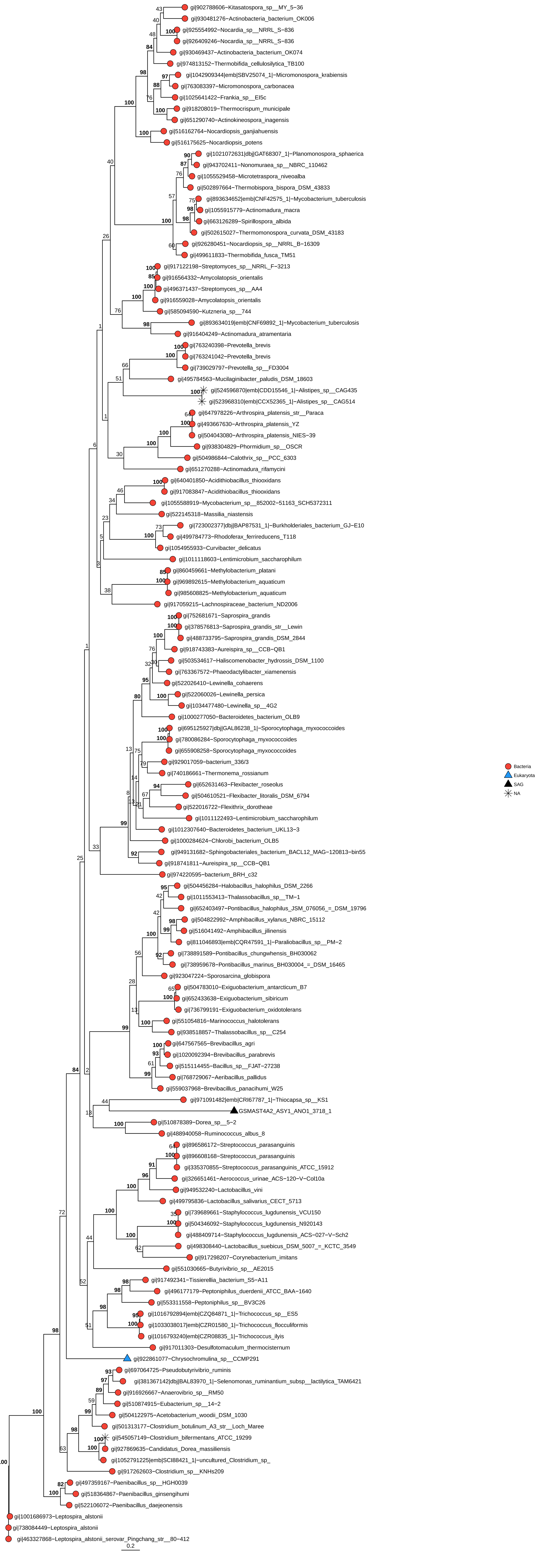
gi|902788606~Kitasatospora_sp__MY_5~36
gi|930481276~Actinobacteria_bacterium_OK006
gi|925554992~Nocardia_sp__NRRL_S~836
gi|926409246~Nocardia_sp__NRRL_S~836
gi|930469437~Actinobacteria_bacterium_OK074
gi|974813152~Thermobifida_cellulosilytica_TB100
gi|1042909344|emb|SBV25074_1|~Micromonospora_krabiensis
gi|763083397~Micromonospora_carbonacea
gi|1025641422~Frankia_sp__EI5c
gi|918208019~Thermocrispum_municipale
gi|651290740~Actinokineospora_inagensis
gi|516162764~Nocardiopsis_ganjiahuensis
gi|516175625~Nocardiopsis_potens
gi|1021072631|dbj|GAT68307_1|~Planomonospora_sphaerica
gi|943702411~Nonomuraea_sp__NBRC_110462
gi|1055529458~Microtetraspora_niveoalba
gi|502897664~Thermobispora_bispora_DSM_43833
gi|893634652|emb|CNF42575_1|~Mycobacterium_tuberculosis
gi|1055915779~Actinomadura_macra
gi|663126289~Spirillospora_albida
gi|502615027~Thermomonospora_curvata_DSM_43183
gi|926280451~Nocardiopsis_sp__NRRL_B~16309
gi|499611833~Thermobifida_fusca_TM51
gi|917122198~Streptomyces_sp__NRRL_F~3213
gi|916564332~Amycolatopsis_orientalis
gi|496371437~Streptomyces_sp__AA4
gi|916559028~Amycolatopsis_orientalis
gi|585094590~Kutzneria_sp__744
gi|893634019|emb|CNF69892_1|~Mycobacterium_tuberculosis
gi|916404249~Actinomadura_atramentaria
gi|763240398~Prevotella_brevis
gi|763241042~Prevotella_brevis
gi|739029797~Prevotella_sp__FD3004
gi|495784563~Mucilaginibacter_paludis_DSM_18603
gi|524596870|emb|CDD15546_1|~Alistipes_sp__CAG435
gi|523968310|emb|CCX52365_1|~Alistipes_sp__CAG514
gi|647978226~Arthrospira_platensis_str__Paraca
gi|493667630~Arthrospira_platensis_YZ
gi|504043080~Arthrospira_platensis_NIES~39
gi|938304829~Phormidium_sp__OSCR
gi|504986844~Calothrix_sp__PCC_6303
gi|651270288~Actinomadura_rifamycini
gi|640401850~Acidithiobacillus_thiooxidans
gi|917083847~Acidithiobacillus_thiooxidans
gi|1055588919~Mycobacterium_sp__852002~51163_SCH5372311
gi|522145318~Massilia_niastensis
gi|723002377|dbj|BAP87531_1|~Burkholderiales_bacterium_GJ~E10
gi|499784773~Rhodoferax_ferrireducens_T118
gi|1054955933~Curvibacter_delicatus
gi|1011118603~Lentimicrobium_saccharophilum
gi|860459661~Methylobacterium_platani
gi|969892615~Methylobacterium_aquaticum
gi|985608825~Methylobacterium_aquaticum
gi|917059215~Lachnospiraceae_bacterium_ND2006
gi|752681671~Saprospira_grandis
gi|378576813~Saprospira_grandis_str__Lewin
gi|488733795~Saprospira_grandis_DSM_2844
gi|918743383~Aureispira_sp__CCB~QB1
gi|503534617~Haliscomenobacter_hydrossis_DSM_1100
gi|763367572~Phaeodactylibacter_xiamenensis
gi|522026410~Lewinella_cohaerens
gi|522060026~Lewinella_persica
gi|1034477480~Lewinella_sp__4G2
gi|1000277050~Bacteroidetes_bacterium_OLB9
gi|695125927|dbj|GAL86238_1|~Sporocytophaga_myxococcoides
gi|780086284~Sporocytophaga_myxococcoides
gi|655908258~Sporocytophaga_myxococcoides
gi|929017059~bacterium_336/3
gi|740186661~Thermonema_rossianum
gi|652631463~Flexibacter_roseolus
gi|504610521~Flexibacter_litoralis_DSM_6794
gi|522016722~Flexithrix_dorotheae
gi|1011122493~Lentimicrobium_saccharophilum
gi|1012307640~Bacteroidetes_bacterium_UKL13~3
gi|1000284624~Chlorobi_bacterium_OLB5
gi|949131682~Sphingobacteriales_bacterium_BACL12_MAG~120813~bin55
gi|918741811~Aureispira_sp__CCB~QB1
gi|974220595~bacterium_BRH_c32
gi|504456284~Halobacillus_halophilus_DSM_2266
gi|1011553413~Thalassobacillus_sp__TM~1
gi|652403497~Pontibacillus_halophilus_JSM_076056_=_DSM_19796
gi|504822992~Amphibacillus_xylanus_NBRC_15112
gi|516041492~Amphibacillus_jilinensis
gi|811046893|emb|CQR47591_1|~Paraliobacillus_sp__PM~2
gi|738891589~Pontibacillus_chungwhensis_BH030062
gi|738959678~Pontibacillus_marinus_BH030004_=_DSM_16465
gi|923047224~Sporosarcina_globispora
gi|504783010~Exiguobacterium_antarcticum_B7
gi|652433638~Exiguobacterium_sibiricum
gi|736799191~Exiguobacterium_oxidotolerans
gi|551054816~Marinococcus_halotolerans
gi|938518857~Thalassobacillus_sp__C254
gi|647567565~Brevibacillus_agri
gi|1020092394~Brevibacillus_parabrevis
gi|515114455~Bacillus_sp__FJAT~27238
gi|768729067~Aeribacillus_pallidus
gi|559037968~Brevibacillus_panacihumi_W25
gi|971091482|emb|CRI67787_1|~Thiocapsa_sp__KS1
GSMAST4A2_ASY1_ANO1_3718_1
gi|510878389~Dorea_sp__5~2
gi|488940058~Ruminococcus_albus_8
gi|896586172~Streptococcus_parasanguinis
gi|896608168~Streptococcus_parasanguinis
gi|335370855~Streptococcus_parasanguinis_ATCC_15912
gi|326651461~Aerococcus_urinae_ACS~120~V~Col10a
gi|949532240~Lactobacillus_vini
gi|499795836~Lactobacillus_salivarius_CECT_5713
gi|739689661~Staphylococcus_lugdunensis_VCU150
gi|504346092~Staphylococcus_lugdunensis_N920143
gi|488409714~Staphylococcus_lugdunensis_ACS~027~V~Sch2
gi|498308440~Lactobacillus_suebicus_DSM_5007_=_KCTC_3549
gi|917298207~Corynebacterium_imitans
gi|551030665~Butyrivibrio_sp__AE2015
gi|917492341~Tissierella_bacterium_S5~A11
gi|496177179~Peptoniphilus_duerdenii_ATCC_BAA~1640
gi|553311558~Peptoniphilus_sp__BV3C26
gi|1016792894|emb|CZQ84871_1|~Trichococcus_sp__ES5
gi|1033038017|emb|CZR01580_1|~Trichococcus_flocculiformis
gi|1016793240|emb|CZR08835_1|~Trichococcus_ilyis
gi|917011303~Desulfotomaculum_thermocisternum
gi|922861077~Chrysochromulina_sp__CCMP291
gi|697064725~Pseudobutyrivibrio_ruminis
gi|381367142|dbj|BAL83970_1|~Selenomonas_ruminantium_subsp__lactilytica_TAM6421
gi|916926667~Anaerovibrio_sp__RM50
gi|510874915~Eubacterium_sp__14~2
gi|504122975~Acetobacterium_woodii_DSM_1030
gi|501313177~Clostridium_botulinum_A3_str__Loch_Maree
gi|545057149~Clostridium_bifermentans_ATCC_19299
gi|927869635~Candidatus_Dorea_massiliensis
gi|1052791225|emb|SCI88421_1|~uncultured_Clostridium_sp_
gi|917262603~Clostridium_sp__KNHs209
gi|497359167~Paenibacillus_sp__HGH0039
gi|518364867~Paenibacillus_ginsengihumi
gi|522106072~Paenibacillus_daejeonensis
gi|1001686973~Leptospira_alstonii
gi|738084449~Leptospira_alstonii
gi|463327868~Leptospira_alstonii_serovar_Pingchang_str__80~412
0.2
Bacteria
Eukaryota
SAG
NA

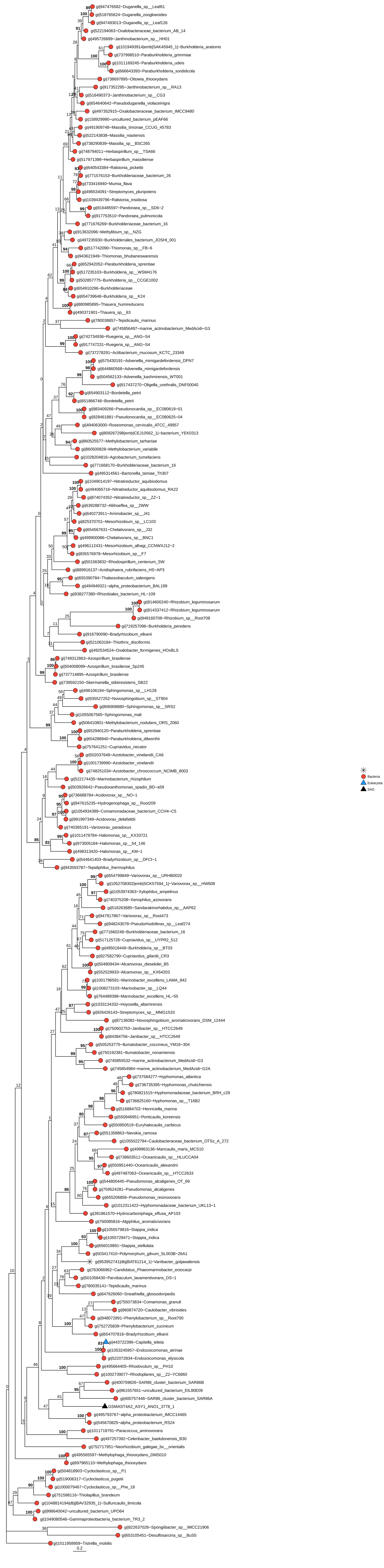

Bacteria
Eukaryota
SAG
0.2

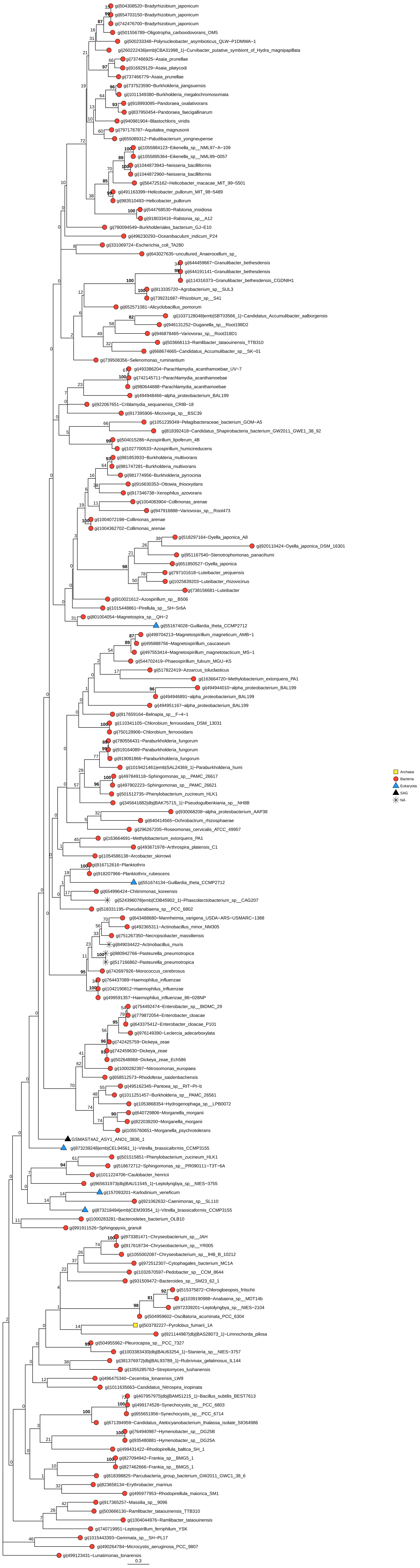

Archaea
Bacteria
Eukaryota
SAG
NA
gi|504308520~Bradyrhizobium_japonicum
gi|654703150~Bradyrhizobium_japonicum
gi|742476700~Bradyrhizobium_japonicum
gi|501556789~Oligotropha_carboxidovorans_OM5
gi|500233348~Polynucleobacter_asymbioticus_QLW~P1DMWA~1
gi|260222436|emb|CBA31998_1|~Curvibacter_putative_symbiont_of_Hydra_magnipapillata
gi|737466925~Asaia_prunellae
gi|916929129~Asaia_platycodi
gi|737466779~Asaia_prunellae
gi|737523590~Burkholderia_jiangsuensis
gi|1011349380~Burkholderia_megalochromosomata
gi|918993095~Pandoraea_oxalativorans
gi|837950454~Pandoraea_faecigallinarum
gi|940981904~Blastochloris_viridis
gi|797176787~Aquitalea_magnusonii
gi|655089312~Paludibacterium_yongneupense
gi|1055884123~Eikenella_sp__NML97~A~109
gi|1055895364~Eikenella_sp__NML99~0057
gi|1044873943~Neisseria_bacilliformis
gi|1044872960~Neisseria_bacilliformis
gi|564725162~Helicobacter_macacae_MIT_99~5501
gi|491163399~Helicobacter_pullorum_MIT_98~5489
gi|983510493~Helicobacter_pullorum
gi|544768530~Ralstonia_insidiosa
gi|918033416~Ralstonia_sp__A12
gi|780094549~Burkholderiales_bacterium_GJ~E10
gi|496230293~Oceanibaculum_indicum_P24
gi|331069724~Escherichia_coli_TA280
gi|643027635~uncultured_Anaerocellum_sp_
gi|644459667~Granulibacter_bethesdensis
gi|644191141~Granulibacter_bethesdensis
gi|114316373~Granulibacter_bethesdensis_CGDNIH1
gi|913335720~Agrobacterium_sp__SUL3
gi|739231687~Rhizobium_sp__S41
gi|652571081~Alicyclobacillus_pomorum
gi|1037128048|emb|SBT03566_1|~Candidatus_Accumulibacter_aalborgensis
gi|946131252~Duganella_sp__Root198D2
gi|946878465~Variovorax_sp__Root318D1
gi|503666113~Ramlibacter_tataouinensis_TTB310
gi|668674665~Candidatus_Accumulibacter_sp__SK~01
gi|739508356~Selenomonas_ruminantium
gi|493386204~Parachlamydia_acanthamoebae_UV~7
gi|742145711~Parachlamydia_acanthamoebae
gi|980644688~Parachlamydia_acanthamoebae
gi|494948466~alpha_proteobacterium_BAL199
gi|922067651~Criblamydia_sequanensis_CRIB~18
gi|917395906~Microvirga_sp__BSC39
gi|1051239349~Pelagibacteraceae_bacterium_GOM~A5
gi|818392418~Candidatus_Shapirobacteria_bacterium_GW2011_GWE1_38_92
gi|504015286~Azospirillum_lipoferum_4B
gi|1027700533~Azospirillum_humicireducens
gi|981853933~Burkholderia_multivorans
gi|981747281~Burkholderia_multivorans
gi|981774956~Burkholderia_pyrrocinia
gi|916630353~Ottowia_thiooxydans
gi|917346738~Xenophilus_azovorans
gi|1004083904~Collimonas_arenae
gi|947916888~Variovorax_sp__Root473
gi|1004072198~Collimonas_arenae
gi|1004362702~Collimonas_arenae
gi|518297164~Dyella_japonica_A8
gi|920110424~Dyella_japonica_DSM_16301
gi|951167540~Stenotrophomonas_panacihumi
gi|651850527~Dyella_japonica
gi|797101618~Luteibacter_yeojuensis
gi|1025839203~Luteibacter_rhizovicinus
gi|738156681~Luteibacter
gi|910021612~Azospirillum_sp__B506
gi|1015448861~Pirellula_sp__SH~Sr6A
gi|801004054~Magnetospira_sp__QH~2
gi|551674028~Guillardia_theta_CCMP2712
gi|499704213~Magnetospirillum_magneticum_AMB~1
gi|495888756~Magnetospirillum_caucaseum
gi|497553414~Magnetospirillum_magnetotacticum_MS~1
gi|544702419~Phaeospirillum_fulvum_MGU~K5
gi|517822419~Azoarcus_toluclasticus
gi|163664720~Methylobacterium_extorquens_PA1
gi|494944010~alpha_proteobacterium_BAL199
gi|494946891~alpha_proteobacterium_BAL199
gi|494951167~alpha_proteobacterium_BAL199
gi|917659164~Belnapia_sp__F~4~1
gi|110341105~Chlorobium_ferrooxidans_DSM_13031
gi|750128906~Chlorobium_ferrooxidans
gi|780556431~Paraburkholderia_fungorum
gi|919164089~Paraburkholderia_fungorum
gi|919091866~Paraburkholderia_fungorum
gi|1019421461|emb|SAL24369_1|~Paraburkholderia_humi
gi|497849118~Sphingomonas_sp__PAMC_26617
gi|497902223~Sphingomonas_sp__PAMC_26621
gi|501512735~Phenylobacterium_zucineum_HLK1
gi|345641882|dbj|BAK75715_1|~Pseudogulbenkiania_sp__NH8B
gi|930068208~alpha_proteobacterium_AAP38
gi|640414565~Ochrobactrum_rhizosphaerae
gi|296267205~Roseomonas_cervicalis_ATCC_49957
gi|163664691~Methylobacterium_extorquens_PA1
gi|493671978~Arthrospira_platensis_C1
gi|1054586138~Arcobacter_skirrowii
gi|916712616~Planktothrix
gi|918207966~Planktothrix_rubescens
gi|551674134~Guillardia_theta_CCMP2712
gi|654996424~Chitinimonas_koreensis
gi|524396078|emb|CDB45902_1|~Phascolarctobacterium_sp__CAG207
gi|518331195~Pseudanabaena_sp__PCC_6802
gi|643488680~Mannheimia_varigena_USDA~ARS~USMARC~1388
gi|492365311~Actinobacillus_minor_NM305
gi|751267350~Necropsobacter_massiliensis
gi|849034422~Actinobacillus_muris
gi|980942766~Pasteurella_pneumotropica
gi|517662166~Pasteurella_pneumotropica
gi|742697926~Moraxella_cerebrosus
gi|764437089~Haemophilus_influenzae
gi|1042190812~Haemophilus_influenzae
gi|499591357~Haemophilus_influenzae_86~028NP
gi|754492474~Enterobacter_sp__BIDMC_29
gi|779872054~Enterobacter_cloacae
gi|643375412~Enterobacter_cloacae_P101
gi|976149390~Leclercia_adecarboxylata
gi|742425759~Dickeya_zeae
gi|742459630~Dickeya_zeae
gi|502648968~Dickeya_zeae_Ech586
gi|1000282397~Nitrosomonas_europaea
gi|658512573~Rhodoferax_saidenbachensis
gi|495162345~Pantoea_sp__RIT~PI~b
gi|1011251457~Burkholderia_sp__PAMC_26561
gi|1053868354~Hydrogenophaga_sp__LPB0072
gi|640729806~Morganella_morganii
gi|822038200~Morganella_morganii
gi|1055760651~Morganella_psychrotolerans
GSMAST4A2_ASY1_ANO1_3836_1
gi|873239248|emb|CEL94561_1|~Vitrella_brassicaformis_CCMP3155
gi|501515851~Phenylobacterium_zucineum_HLK1
gi|518672712~Sphingomonas_sp__PR090111~T3T~6A
gi|1011224706~Caulobacter_henricii
gi|965631973|dbj|BAU11545_1|~Leptolyngbya_sp__NIES~3755
gi|157093201~Karlodinium_veneficum
gi|921062632~Caenimonas_sp__SL110
gi|873218494|emb|CEM39354_1|~Vitrella_brassicaformis_CCMP3155
gi|1000283281~Bacteroidetes_bacterium_OLB10
gi|991911526~Sphingopyxis_granuli
gi|973381471~Chryseobacterium_sp__JAH
gi|917618734~Chryseobacterium_sp__YR005
gi|1055002087~Chryseobacterium_sp__IHB_B_10212
gi|972512307~Cytophagales_bacterium_MC1A
gi|1032670597~Pedobacter_sp__CCM_8644
gi|931509472~Bacteroides_sp__SM23_62_1
gi|515375872~Chlorogloeopsis_fritschii
gi|1039190988~Anabaena_sp__MDT14b
gi|972339201~Leptolyngbya_sp__NIES~2104
gi|504959602~Oscillatoria_acuminata_PCC_6304
gi|503792227~Pyrolobus_fumarii_1A
gi|921144987|dbj|BAS28073_1|~Limnochorda_pilosa
gi|504955962~Pleurocapsa_sp__PCC_7327
gi|1003383430|dbj|BAU63254_1|~Stanieria_sp__NIES~3757
gi|381376972|dbj|BAL93789_1|~Rubrivivax_gelatinosus_IL144
gi|1055285763~Streptomyces_lushanensis
gi|496475340~Cecembia_lonarensis_LW9
gi|1011635663~Candidatus_Nitrospira_inopinata
gi|407957975|dbj|BAM51215_1|~Bacillus_subtilis_BEST7613
gi|499174528~Synechocystis_sp__PCC_6803
gi|655651956~Synechocystis_sp__PCC_6714
gi|671394959~Candidatus_Atelocyanobacterium_thalassa_isolate_SIO64986
gi|764940987~Hymenobacter_sp__DG25B
gi|935480881~Hymenobacter_sp__DG25A
gi|499431422~Rhodopirellula_baltica_SH_1
gi|827094942~Frankia_sp__BMG5_1
gi|827462666~Frankia_sp__BMG5_1
gi|818398825~Parcubacteria_group_bacterium_GW2011_GWC1_38_6
gi|823658134~Erythrobacter_marinus
gi|495977953~Rhodopirellula_maiorica_SM1
gi|917365257~Massilia_sp__9096
gi|503666130~Ramlibacter_tataouinensis_TTB310
gi|1004044976~Ramlibacter_tataouinensis
gi|740719951~Leptospirillum_ferriphilum_YSK
gi|1015443393~Gemmata_sp__SH~PL17
gi|490264784~Microcystis_aeruginosa_PCC_9807
gi|499123431~Lunatimonas_lonarensis
0.3

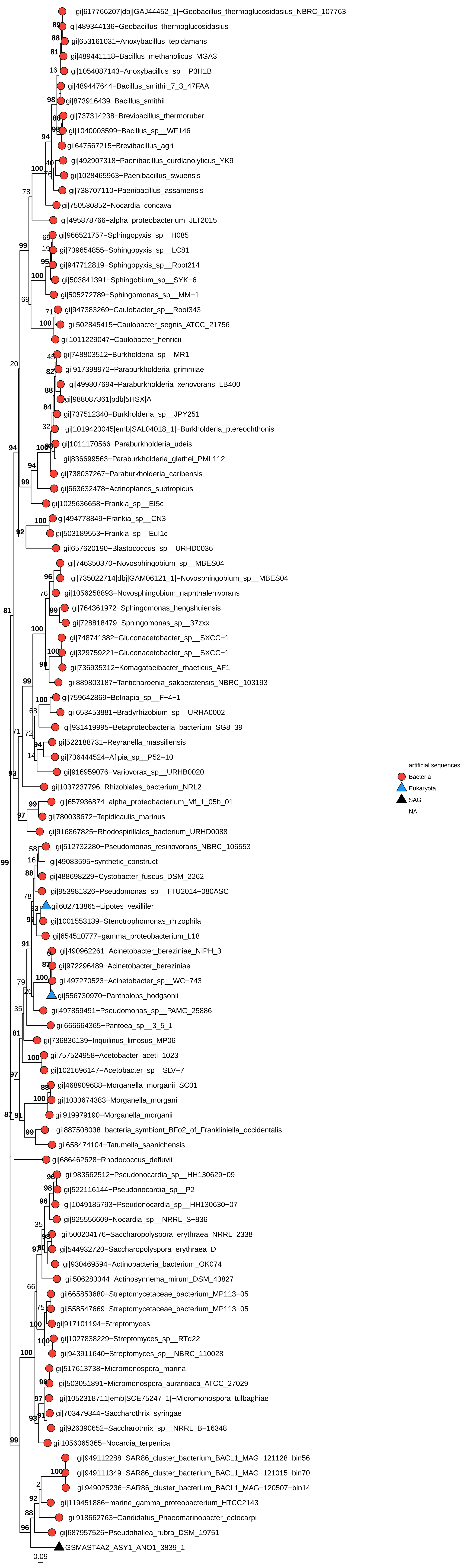
gi|617766207|dbj|GAJ44452_1|−Geobacillus_thermoglucosidasius_NBRC_107763
gi|489344136−Geobacillus_thermoglucosidasius
gi|653161031−Anoxybacillus_tepidamans
gi|489441118−Bacillus_methanolicus_MGA3
gi|1054087143−Anoxybacillus_sp__P3H1B
gi|489447644−Bacillus_smithii_7_3_47FAA
gi|873916439−Bacillus_smithii
gi|737314238−Brevibacillus_thermoruber
gi|1040003599−Bacillus_sp__WF146
gi|647567215−Brevibacillus_agri
gi|492907318−Paenibacillus_curdlanolyticus_YK9
gi|1028465963−Paenibacillus_swuensis
gi|738707110−Paenibacillus_assamensis
gi|750530852−Nocardia_concava
gi|495878766−alpha_proteobacterium_JLT2015
gi|966521757−Sphingopyxis_sp__H085
gi|739654855−Sphingopyxis_sp__LC81
gi|947712819−Sphingopyxis_sp__Root214
gi|503841391−Sphingobium_sp__SYK−6
gi|505272789−Sphingomonas_sp__MM−1
gi|947383269−Caulobacter_sp__Root343
gi|502845415−Caulobacter_segnis_ATCC_21756
gi|1011229047−Caulobacter_henricii
gi|748803512−Burkholderia_sp__MR1
gi|917398972−Paraburkholderia_grimmiae
gi|499807694−Paraburkholderia_xenovorans_LB400
gi|988087361|pdb|5HSX|A
gi|737512340−Burkholderia_sp__JPY251
gi|1019423045|emb|SAL04018_1|−Burkholderia_ptereochthonis
gi|1011170566−Paraburkholderia_udeis
gi|836699563−Paraburkholderia_glathei_PML112
gi|738037267−Paraburkholderia_caribensis
gi|663632478−Actinoplanes_subtropicus
gi|1025636658−Frankia_sp__EI5c
gi|494778849−Frankia_sp__CN3
gi|503189553−Frankia_sp__EuI1c
gi|657620190−Blastococcus_sp__URHD0036
gi|746350370−Novosphingobium_sp__MBES04
gi|735022714|dbj|GAM06121_1|−Novosphingobium_sp__MBES04
gi|1056258893−Novosphingobium_naphthalenivorans
gi|764361972−Sphingomonas_hengshuiensis
gi|728818479−Sphingomonas_sp__37zxx
gi|748741382−Gluconacetobacter_sp__SXCC−1
gi|329759221−Gluconacetobacter_sp__SXCC−1
gi|736935312−Komagataeibacter_rhaeticus_AF1
gi|889803187−Tanticharoenia_sakaeratensis_NBRC_103193
gi|759642869−Belnapia_sp__F−4−1
gi|653453881−Bradyrhizobium_sp__URHA0002
gi|931419995−Betaproteobacteria_bacterium_SG8_39
gi|522188731−Reyranella_massiliensis
gi|736444524−Afipia_sp__P52−10
gi|916959076−Variovorax_sp__URHB0020
gi|1037237796−Rhizobiales_bacterium_NRL2
gi|657936874−alpha_proteobacterium_Mf_1_05b_01
gi|780038672−Tepidicaulis_marinus
gi|916867825−Rhodospirillales_bacterium_URHD0088
gi|512732280−Pseudomonas_resinovorans_NBRC_106553
gi|49083595−synthetic_construct
gi|488698229−Cystobacter_fuscus_DSM_2262
gi|953981326−Pseudomonas_sp__TTU2014−080ASC
gi|602713865−Lipotes_vexillifer
gi|1001553139−Stenotrophomonas_rhizophila
gi|654510777−gamma_proteobacterium_L18
gi|490962261−Acinetobacter_bereziniae_NIPH_3
gi|972296489−Acinetobacter_bereziniae
gi|497270523−Acinetobacter_sp__WC−743
gi|556730970−Pantholops_hodgsonii
gi|497859491−Pseudomonas_sp__PAMC_25886
gi|666664365−Pantoea_sp__3_5_1
gi|736836139−Inquilinus_limosus_MP06
gi|757524958−Acetobacter_aceti_1023
gi|1021696147−Acetobacter_sp__SLV−7
gi|468909688−Morganella_morganii_SC01
gi|1033674383−Morganella_morganii
gi|919979190−Morganella_morganii
gi|887508038−bacteria_symbiont_BFo2_of_Frankliniella_occidentalis
gi|658474104−Tatumella_saanichensis
gi|686462628−Rhodococcus_defluvii
gi|983562512−Pseudonocardia_sp__HH130629−09
gi|522116144−Pseudonocardia_sp__P2
gi|1049185793−Pseudonocardia_sp__HH130630−07
gi|925556609−Nocardia_sp__NRRL_S−836
gi|500204176−Saccharopolyspora_erythraea_NRRL_2338
gi|544932720−Saccharopolyspora_erythraea_D
gi|930469594−Actinobacteria_bacterium_OK074
gi|506283344−Actinosynnema_mirum_DSM_43827
gi|665853680−Streptomycetaceae_bacterium_MP113−05
gi|558547669−Streptomycetaceae_bacterium_MP113−05
gi|917101194−Streptomyces
gi|1027838229−Streptomyces_sp__RTd22
gi|943911640−Streptomyces_sp__NBRC_110028
gi|517613738−Micromonospora_marina
gi|503051891−Micromonospora_aurantiaca_ATCC_27029
gi|1052318711|emb|SCE75247_1|−Micromonospora_tulbaghiae
gi|703479344−Saccharothrix_syringae
gi|926390652−Saccharothrix_sp__NRRL_B−16348
gi|1056065365−Nocardia_terpenica
gi|949112288−SAR86_cluster_bacterium_BACL1_MAG−121128−bin56
gi|949111349−SAR86_cluster_bacterium_BACL1_MAG−121015−bin70
gi|949025236−SAR86_cluster_bacterium_BACL1_MAG−120507−bin14
gi|119451886−marine_gamma_proteobacterium_HTCC2143
gi|918662763−Candidatus_Phaeomarinobacter_ectocarpi
gi|687957526−Pseudohaliea_rubra_DSM_19751
GSMAST4A2_ASY1_ANO1_3839_1
0.09
artificial sequences
Bacteria
Eukaryota
SAG
NA

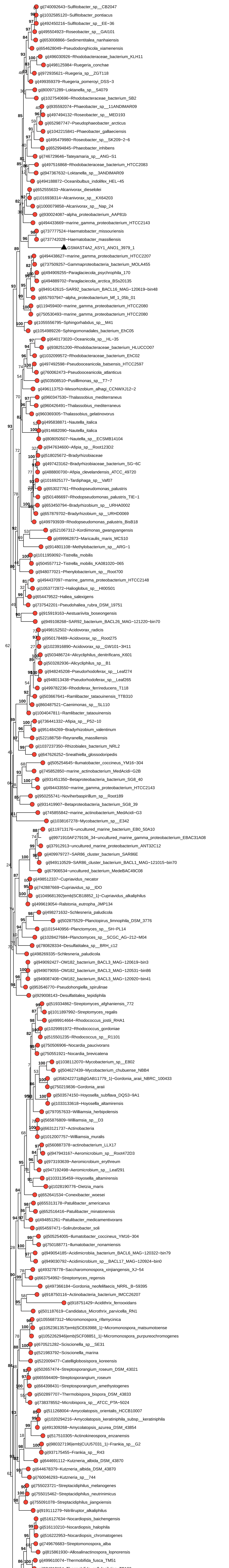
Bacteria
SAG
NA
0.1

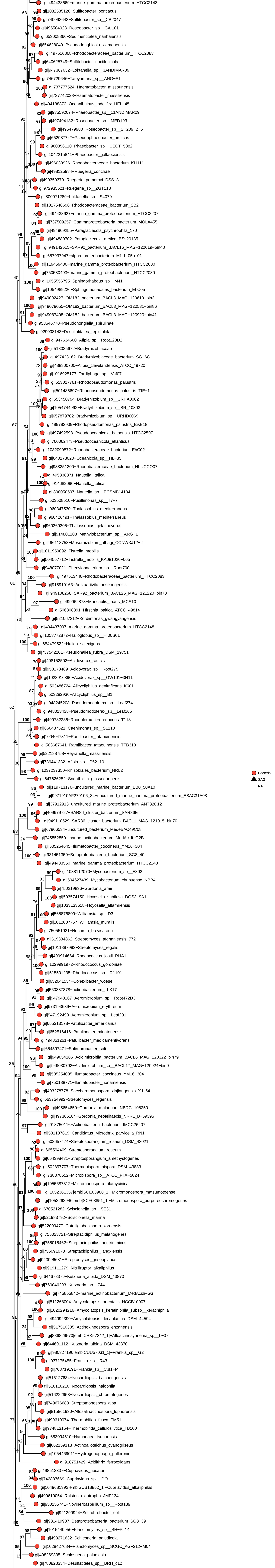

Bacteria
SAG
NA
0.1

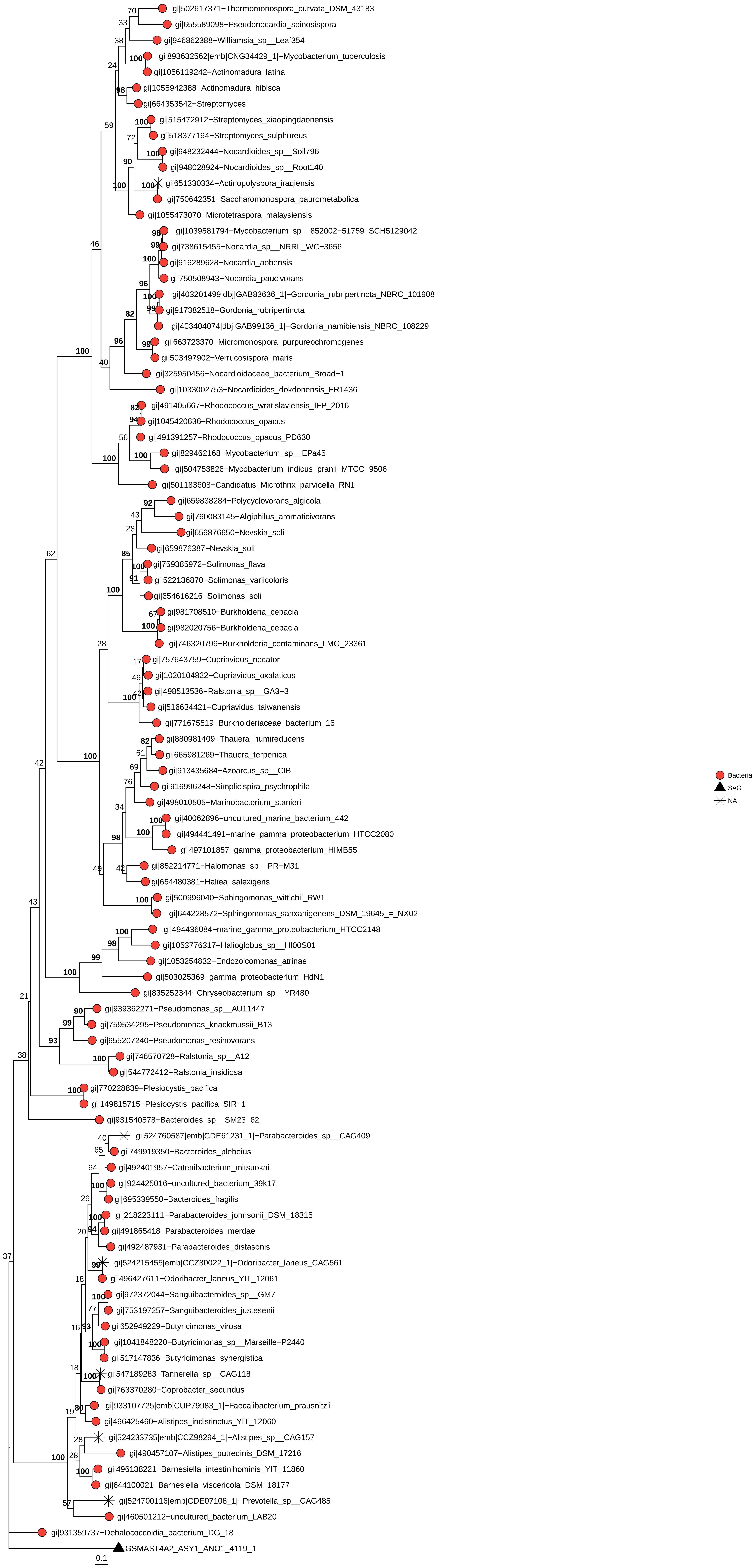
gi|502617371−Thermomonospora_curvata_DSM_43183
gi|655589098−Pseudonocardia_spinosispora
gi|946862388−Williamsia_sp__Leaf354
gi|893632562|emb|CNG34429_1|−Mycobacterium_tuberculosis
gi|1056119242−Actinomadura_latina
gi|1055942388−Actinomadura_hibisca
gi|664353542−Streptomyces
gi|515472912−Streptomyces_xiaopingdaonensis
gi|518377194−Streptomyces_sulphureus
gi|948232444−Nocardioides_sp__Soil796
gi|948028924−Nocardioides_sp__Root140
gi|651330334−Actinopolyspora_iraqiensis
gi|750642351−Saccharomonospora_paurometabolica
gi|1055473070−Microtetraspora_malaysiensis
gi|1039581794−Mycobacterium_sp__852002−51759_SCH5129042
gi|738615455−Nocardia_sp__NRRL_WC−3656
gi|916289628−Nocardia_aobensis
gi|750508943−Nocardia_paucivorans
gi|403201499|dbj|GAB83636_1|−Gordonia_rubripertincta_NBRC_101908
gi|917382518−Gordonia_rubripertincta
gi|403404074|dbj|GAB99136_1|−Gordonia_namibiensis_NBRC_108229
gi|663723370−Micromonospora_purpureochromogenes
gi|503497902−Verrucosispora_maris
gi|325950456−Nocardioidaceae_bacterium_Broad−1
gi|1033002753−Nocardioides_dokdonensis_FR1436
gi|491405667−Rhodococcus_wratislaviensis_IFP_2016
gi|1045420636−Rhodococcus_opacus
gi|491391257−Rhodococcus_opacus_PD630
gi|829462168−Mycobacterium_sp__EPa45
gi|504753826−Mycobacterium_indicus_pranii_MTCC_9506
gi|501183608−Candidatus_Microthrix_parvicella_RN1
gi|659838284−Polycyclovorans_algicola
gi|760083145−Algiphilus_aromaticivorans
gi|659876650−Nevskia_soli
gi|659876387−Nevskia_soli
gi|759385972−Solimonas_flava
gi|522136870−Solimonas_variicoloris
gi|654616216−Solimonas_soli
gi|981708510−Burkholderia_cepacia
gi|982020756−Burkholderia_cepacia
gi|746320799−Burkholderia_contaminans_LMG_23361
gi|757643759−Cupriavidus_necator
gi|1020104822−Cupriavidus_oxalaticus
gi|498513536−Ralstonia_sp__GA3−3
gi|516634421−Cupriavidus_taiwanensis
gi|771675519−Burkholderiaceae_bacterium_16
gi|880981409−Thauera_humireducens
gi|665981269−Thauera_terpenica
gi|913435684−Azoarcus_sp__CIB
gi|916996248−Simplicispira_psychrophila
gi|498010505−Marinobacterium_stanieri
gi|40062896−uncultured_marine_bacterium_442
gi|494441491−marine_gamma_proteobacterium_HTCC2080
gi|497101857−gamma_proteobacterium_HIMB55
gi|852214771−Halomonas_sp__PR−M31
gi|654480381−Haliea_salexigens
gi|500996040−Sphingomonas_wittichii_RW1
gi|644228572−Sphingomonas_sanxanigenens_DSM_19645_=_NX02
gi|494436084−marine_gamma_proteobacterium_HTCC2148
gi|1053776317−Halioglobus_sp__HI00S01
gi|1053254832−Endozoicomonas_atrinae
gi|503025369−gamma_proteobacterium_HdN1
gi|835252344−Chryseobacterium_sp__YR480
gi|939362271−Pseudomonas_sp__AU11447
gi|759534295−Pseudomonas_knackmussii_B13
gi|655207240−Pseudomonas_resinovorans
gi|746570728−Ralstonia_sp__A12
gi|544772412−Ralstonia_insidiosa
gi|770228839−Plesiocystis_pacifica
gi|149815715−Plesiocystis_pacifica_SIR−1
gi|931540578−Bacteroides_sp__SM23_62
gi|524760587|emb|CDE61231_1|−Parabacteroides_sp__CAG409
gi|749919350−Bacteroides_plebeius
gi|492401957−Catenibacterium_mitsuokai
gi|924425016−uncultured_bacterium_39k17
gi|695339550−Bacteroides_fragilis
gi|218223111−Parabacteroides_johnsonii_DSM_18315
gi|491865418−Parabacteroides_merdae
gi|492487931−Parabacteroides_distasonis
gi|524215455|emb|CCZ80022_1|−Odoribacter_laneus_CAG561
gi|496427611−Odoribacter_laneus_YIT_12061
gi|972372044−Sanguibacteroides_sp__GM7
gi|753197257−Sanguibacteroides_justesenii
gi|652949229−Butyricimonas_virosa
gi|1041848220−Butyricimonas_sp__Marseille−P2440
gi|517147836−Butyricimonas_synergistica
gi|547189283−Tannerella_sp__CAG118
gi|763370280−Coprobacter_secundus
gi|933107725|emb|CUP79983_1|−Faecalibacterium_prausnitzii
gi|496425460−Alistipes_indistinctus_YIT_12060
gi|524233735|emb|CCZ98294_1|−Alistipes_sp__CAG157
gi|490457107−Alistipes_putredinis_DSM_17216
gi|496138221−Barnesiella_intestinihominis_YIT_11860
gi|644100021−Barnesiella_viscericola_DSM_18177
gi|524700116|emb|CDE07108_1|−Prevotella_sp__CAG485
gi|460501212−uncultured_bacterium_LAB20
gi|931359737−Dehalococcoidia_bacterium_DG_18
GSMAST4A2_ASY1_ANO1_4119_1
0.1
Bacteria
SAG
NA

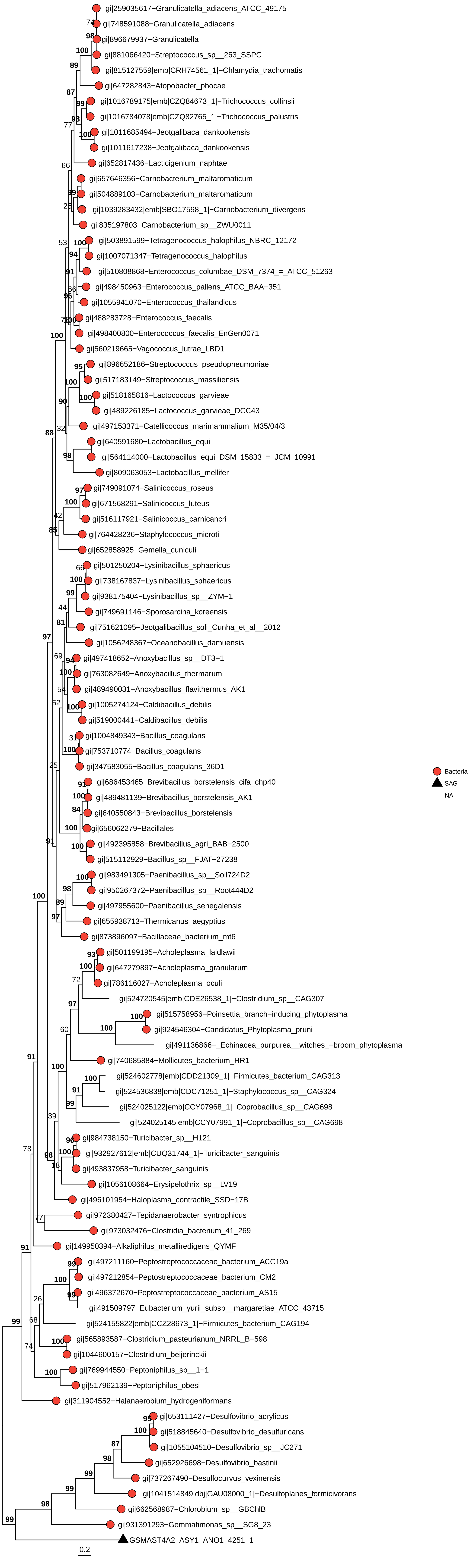
gi|259035617−Granulicatella_adiacens_ATCC_49175
gi|748591088−Granulicatella_adiacens
gi|896679937−Granulicatella
gi|881066420−Streptococcus_sp__263_SSPC
gi|815127559|emb|CRH74561_1|−Chlamydia_trachomatis
gi|647282843−Atopobacter_phocae
gi|1016789175|emb|CZQ84673_1|−Trichococcus_collinsii
gi|1016784078|emb|CZQ82765_1|−Trichococcus_palustris
gi|1011685494−Jeotgalibaca_dankookensis
gi|1011617238−Jeotgalibaca_dankookensis
gi|652817436−Lacticigenium_naphtae
gi|657646356−Carnobacterium_maltaromaticum
gi|504889103−Carnobacterium_maltaromaticum
gi|1039283432|emb|SBO17598_1|−Carnobacterium_divergens
gi|835197803−Carnobacterium_sp__ZWU0011
gi|503891599−Tetragenococcus_halophilus_NBRC_12172
gi|1007071347−Tetragenococcus_halophilus
gi|510808868−Enterococcus_columbae_DSM_7374_=_ATCC_51263
gi|498450963−Enterococcus_pallens_ATCC_BAA−351
gi|1055941070−Enterococcus_thailandicus
gi|488283728−Enterococcus_faecalis
gi|498400800−Enterococcus_faecalis_EnGen0071
gi|560219665−Vagococcus_lutrae_LBD1
gi|896652186−Streptococcus_pseudopneumoniae
gi|517183149−Streptococcus_massiliensis
gi|518165816−Lactococcus_garvieae
gi|489226185−Lactococcus_garvieae_DCC43
gi|497153371−Catellicoccus_marimammalium_M35/04/3
gi|640591680−Lactobacillus_equi
gi|564114000−Lactobacillus_equi_DSM_15833_=_JCM_10991
gi|809063053−Lactobacillus_mellifer
gi|749091074−Salinicoccus_roseus
gi|671568291−Salinicoccus_luteus
gi|516117921−Salinicoccus_carnicancri
gi|764428236−Staphylococcus_microti
gi|652858925−Gemella_cuniculi
gi|501250204−Lysinibacillus_sphaericus
gi|738167837−Lysinibacillus_sphaericus
gi|938175404−Lysinibacillus_sp__ZYM−1
gi|749691146−Sporosarcina_koreensis
gi|751621095−Jeotgalibacillus_soli_Cunha_et_al__2012
gi|1056248367−Oceanobacillus_damuensis
gi|497418652−Anoxybacillus_sp__DT3−1
gi|763082649−Anoxybacillus_thermarum
gi|489490031−Anoxybacillus_flavithermus_AK1
gi|1005274124−Caldibacillus_debilis
gi|519000441−Caldibacillus_debilis
gi|1004849343−Bacillus_coagulans
gi|753710774−Bacillus_coagulans
gi|347583055−Bacillus_coagulans_36D1
gi|686453465−Brevibacillus_borstelensis_cifa_chp40
gi|489481139−Brevibacillus_borstelensis_AK1
gi|640550843−Brevibacillus_borstelensis
gi|656062279−Bacillales
gi|492395858−Brevibacillus_agri_BAB−2500
gi|515112929−Bacillus_sp__FJAT−27238
gi|983491305−Paenibacillus_sp__Soil724D2
gi|950267372−Paenibacillus_sp__Root444D2
gi|497955600−Paenibacillus_senegalensis
gi|655938713−Thermicanus_aegyptius
gi|873896097−Bacillaceae_bacterium_mt6
gi|501199195−Acholeplasma_laidlawii
gi|647279897−Acholeplasma_granularum
gi|786116027−Acholeplasma_oculi
gi|524720545|emb|CDE26538_1|−Clostridium_sp__CAG307
gi|515758956−Poinsettia_branch−inducing_phytoplasma
gi|924546304−Candidatus_Phytoplasma_pruni
gi|491136866−_Echinacea_purpurea__witches_−broom_phytoplasma
gi|740685884−Mollicutes_bacterium_HR1
gi|524602778|emb|CDD21309_1|−Firmicutes_bacterium_CAG313
gi|524536838|emb|CDC71251_1|−Staphylococcus_sp__CAG324
gi|524025122|emb|CCY07968_1|−Coprobacillus_sp__CAG698
gi|524025145|emb|CCY07991_1|−Coprobacillus_sp__CAG698
gi|984738150−Turicibacter_sp__H121
gi|932927612|emb|CUQ31744_1|−Turicibacter_sanguinis
gi|493837958−Turicibacter_sanguinis
gi|1056108664−Erysipelothrix_sp__LV19
gi|496101954−Haloplasma_contractile_SSD−17B
gi|972380427−Tepidanaerobacter_syntrophicus
gi|973032476−Clostridia_bacterium_41_269
gi|149950394−Alkaliphilus_metalliredigens_QYMF
gi|497211160−Peptostreptococcaceae_bacterium_ACC19a
gi|497212854−Peptostreptococcaceae_bacterium_CM2
gi|496372670−Peptostreptococcaceae_bacterium_AS15
gi|491509797−Eubacterium_yurii_subsp__margaretiae_ATCC_43715
gi|524155822|emb|CCZ28673_1|−Firmicutes_bacterium_CAG194
gi|565893587−Clostridium_pasteurianum_NRRL_B−598
gi|1044600157−Clostridium_beijerinckii
gi|769944550−Peptoniphilus_sp__1−1
gi|517962139−Peptoniphilus_obesi
gi|311904552−Halanaerobium_hydrogeniformans
gi|653111427−Desulfovibrio_acrylicus
gi|518845640−Desulfovibrio_desulfuricans
gi|1055104510−Desulfovibrio_sp__JC271
gi|652926698−Desulfovibrio_bastinii
gi|737267490−Desulfocurvus_vexinensis
gi|1041514849|dbj|GAU08000_1|−Desulfoplanes_formicivorans
gi|662568987−Chlorobium_sp__GBChlB
gi|931391293−Gemmatimonas_sp__SG8_23
GSMAST4A2_ASY1_ANO1_4251_1
0.2
Bacteria
SAG
NA

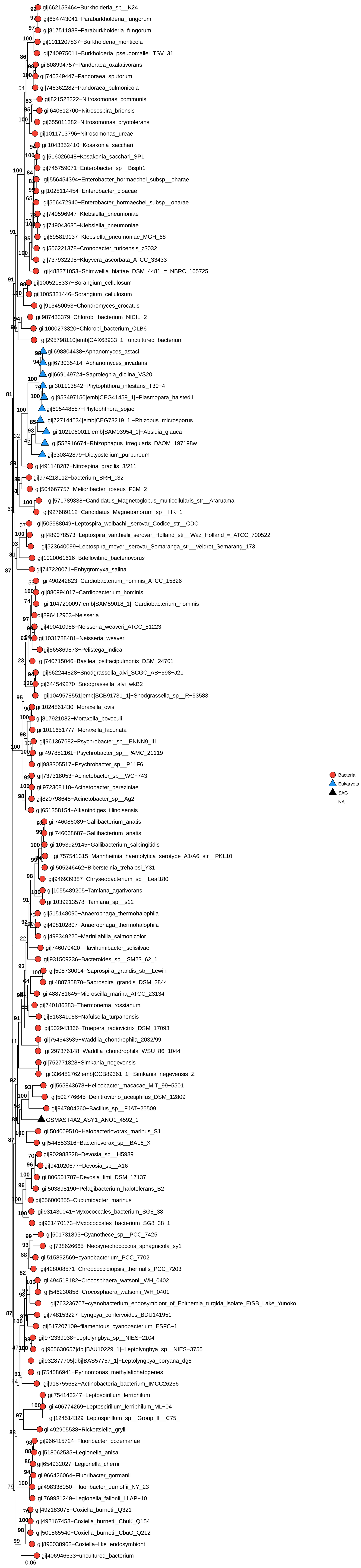

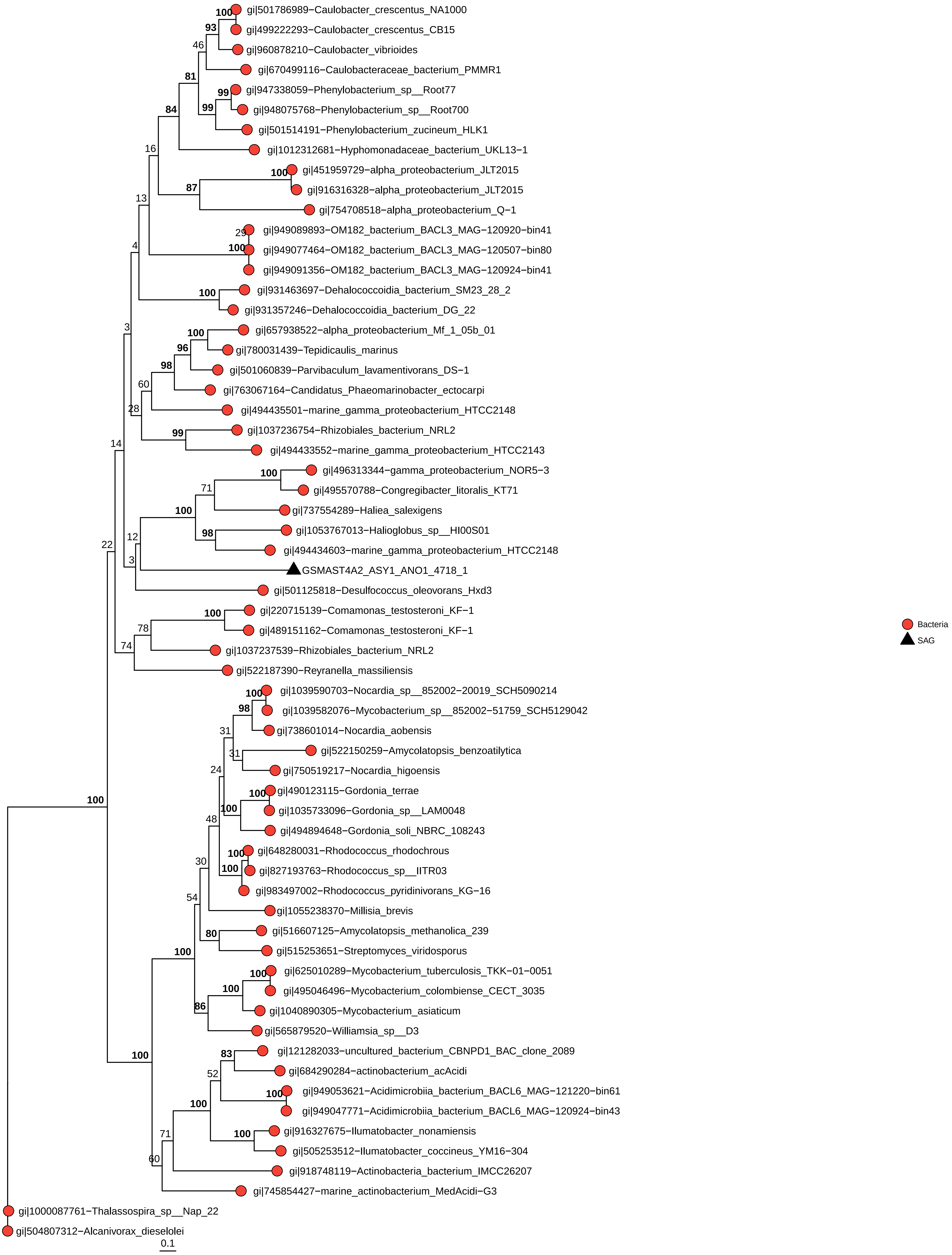

gi|501786989−Caulobacter_crescentus_NA1000
gi|499222293−Caulobacter_crescentus_CB15
gi|960878210−Caulobacter_vibrioides
gi|670499116−Caulobacteraceae_bacterium_PMMR1
gi|947338059−Phenylobacterium_sp__Root77
gi|948075768−Phenylobacterium_sp__Root700
gi|501514191−Phenylobacterium_zucineum_HLK1
gi|1012312681−Hyphomonadaceae_bacterium_UKL13−1
gi|451959729−alpha_proteobacterium_JLT2015
gi|916316328−alpha_proteobacterium_JLT2015
gi|754708518−alpha_proteobacterium_Q−1
gi|949089893−OM182_bacterium_BACL3_MAG−120920−bin41
gi|949077464−OM182_bacterium_BACL3_MAG−120507−bin80
gi|949091356−OM182_bacterium_BACL3_MAG−120924−bin41
gi|931463697−Dehalococcoidia_bacterium_SM23_28_2
gi|931357246−Dehalococcoidia_bacterium_DG_22
gi|657938522−alpha_proteobacterium_Mf_1_05b_01
gi|780031439−Tepidicaulis_marinus
gi|501060839−Parvibaculum_lavamentivorans_DS−1
gi|763067164−Candidatus_Phaeomarinobacter_ectocarpi
gi|494435501−marine_gamma_proteobacterium_HTCC2148
gi|1037236754−Rhizobiales_bacterium_NRL2
gi|494433552−marine_gamma_proteobacterium_HTCC2143
gi|496313344−gamma_proteobacterium_NOR5−3
gi|495570788−Congregibacter_litoralis_KT71
gi|737554289−Haliea_salexigens
gi|1053767013−Halioglobus_sp__HI00S01
gi|494434603−marine_gamma_proteobacterium_HTCC2148
GSMAST4A2_ASY1_ANO1_4718_1
gi|501125818−Desulfococcus_oleovorans_Hxd3
gi|220715139−Comamonas_testosteroni_KF−1
gi|489151162−Comamonas_testosteroni_KF−1
gi|1037237539−Rhizobiales_bacterium_NRL2
gi|522187390−Reyranella_massiliensis
gi|1039590703−Nocardia_sp__852002−20019_SCH5090214
gi|1039582076−Mycobacterium_sp__852002−51759_SCH5129042
gi|738601014−Nocardia_aobensis
gi|522150259−Amycolatopsis_benzoatilytica
gi|750519217−Nocardia_higoensis
gi|490123115−Gordonia_terrae
gi|1035733096−Gordonia_sp__LAM0048
gi|494894648−Gordonia_soli_NBRC_108243
gi|648280031−Rhodococcus_rhodochrous
gi|827193763−Rhodococcus_sp__IITR03
gi|983497002−Rhodococcus_pyridinivorans_KG−16
gi|1055238370−Millisia_brevis
gi|516607125−Amycolatopsis_methanolica_239
gi|515253651−Streptomyces_viridosporus
gi|625010289−Mycobacterium_tuberculosis_TKK−01−0051
gi|495046496−Mycobacterium_colombiense_CECT_3035
gi|1040890305−Mycobacterium_asiaticum
gi|565879520−Williamsia_sp__D3
gi|121282033−uncultured_bacterium_CBNPD1_BAC_clone_2089
gi|684290284−actinobacterium_acAcidi
gi|949053621−Acidimicrobiia_bacterium_BACL6_MAG−121220−bin61
gi|949047771−Acidimicrobiia_bacterium_BACL6_MAG−120924−bin43
gi|916327675−Ilumatobacter_nonamiensis
gi|505253512−Ilumatobacter_coccineus_YM16−304
gi|918748119−Actinobacteria_bacterium_IMCC26207
gi|745854427−marine_actinobacterium_MedAcidi−G3
gi|1000087761−Thalassospira_sp__Nap_22
gi|504807312−Alcanivorax_dieselolei
0.1
Bacteria
SAG

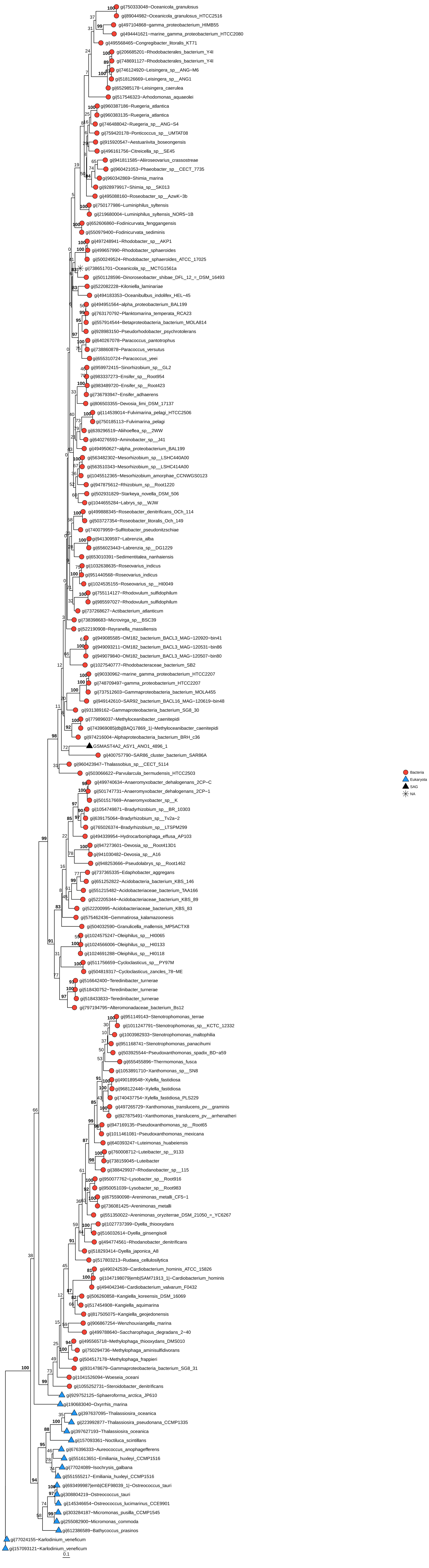

gi|750333048~Oceanicola_granulosus
gi|89044982~Oceanicola_granulosus_HTCC2516
gi|497104868~gamma_proteobacterium_HIMB55
gi|494441621~marine_gamma_proteobacterium_HTCC2080
gi|495568465~Congregibacter_litoralis_KT71
gi|206685201~Rhodobacterales_bacterium_Y4I
gi|748691127~Rhodobacterales_bacterium_Y4I
gi|746124920~Leisingera_sp__ANG~M6
gi|518126669~Leisingera_sp__ANG1
gi|652985178~Leisingera_caerulea
gi|517546323~Arhodomonas_aquaeolei
gi|960387186~Ruegeria_atlantica
gi|960383135~Ruegeria_atlantica
gi|746488042~Ruegeria_sp__ANG~S4
gi|759420178~Ponticoccus_sp__UMTAT08
gi|915920547~Aestuariivita_boseongensis
gi|496161756~Citreicella_sp__SE45
gi|941811585~Aliiroseovarius_crassostreae
gi|960421053~Phaeobacter_sp__CECT_7735
gi|960342869~Shimia_marina
gi|928979917~Shimia_sp__SK013
gi|495088160~Roseobacter_sp__AzwK~3b
gi|750177986~Luminiphilus_syltensis
gi|219680004~Luminiphilus_syltensis_NOR5~1B
gi|652606860~Fodinicurvata_fenggangensis
gi|550979400~Fodinicurvata_sediminis
gi|497248941~Rhodobacter_sp__AKP1
gi|499657990~Rhodobacter_sphaeroides
gi|500249524~Rhodobacter_sphaeroides_ATCC_17025
gi|738651701~Oceanicola_sp__MCTG1561a
gi|501128596~Dinoroseobacter_shibae_DFL_12_=_DSM_16493
gi|522082228~Kiloniella_laminariae
gi|494183353~Oceanibulbus_indolifex_HEL~45
gi|494951564~alpha_proteobacterium_BAL199
gi|763170792~Planktomarina_temperata_RCA23
gi|557914544~Betaproteobacteria_bacterium_MOLA814
gi|928983150~Pseudorhodobacter_psychrotolerans
gi|640267078~Paracoccus_pantotrophus
gi|738860878~Paracoccus_versutus
gi|655310724~Paracoccus_yeei
gi|959972415~Sinorhizobium_sp__GL2
gi|983337273~Ensifer_sp__Root954
gi|983489720~Ensifer_sp__Root423
gi|736793947~Ensifer_adhaerens
gi|806503355~Devosia_limi_DSM_17137
gi|114539014~Fulvimarina_pelagi_HTCC2506
gi|750185113~Fulvimarina_pelagi
gi|639296519~Aliihoeflea_sp__2WW
gi|640276593~Aminobacter_sp__J41
gi|494950627~alpha_proteobacterium_BAL199
gi|563482302~Mesorhizobium_sp__LSHC440A00
gi|563510343~Mesorhizobium_sp__LSHC414A00
gi|1045512365~Mesorhizobium_amorphae_CCNWGS0123
gi|947875612~Rhizobium_sp__Root1220
gi|502931829~Starkeya_novella_DSM_506
gi|1044655284~Labrys_sp__WJW
gi|499888345~Roseobacter_denitrificans_OCh_114
gi|503727354~Roseobacter_litoralis_Och_149
gi|740079959~Sulfitobacter_pseudonitzschiae
gi|941309597~Labrenzia_alba
gi|656023443~Labrenzia_sp__DG1229
gi|653010391~Sedimentitalea_nanhaiensis
gi|1032638635~Roseovarius_indicus
gi|951440568~Roseovarius_indicus
gi|1024535155~Roseovarius_sp__HI0049
gi|755114127~Rhodovulum_sulfidophilum
gi|985597027~Rhodovulum_sulfidophilum
gi|737268627~Actibacterium_atlanticum
gi|738398683~Microvirga_sp__BSC39
gi|522190908~Reyranella_massiliensis
gi|949085585~OM182_bacterium_BACL3_MAG~120920~bin41
gi|949093211~OM182_bacterium_BACL3_MAG~120531~bin86
gi|949079840~OM182_bacterium_BACL3_MAG~120619~bin80
gi|1027540777~Rhodobacteraceae_bacterium_SB2
gi|90330962~marine_gamma_proteobacterium_HTCC2207
gi|748709497~gamma_proteobacterium_HTCC2207
gi|737512603~Gammaproteobacteria_bacterium_MOLA455
gi|949142610~SAR92_bacterium_BACL16_MAG~120619~bin48
gi|931389162~Gammaproteobacteria_bacterium_SG8_30
gi|779896037~Methyloceanibacter_caenitepidi
gi|743969085|dbj|BAQ17869_1|~Methyloceanibacter_caenitepidi
gi|974216004~Alphaproteobacteria_bacterium_BRH_c36
GSMAST4A2_ASY1_ANO1_4896_1
gi|400757790~SAR86_cluster_bacterium_SAR86A
gi|960423947~Thalassobius_sp__CECT_5114
gi|503066622~Parvularcula_bermudensis_HTCC2503
gi|499740634~Anaeromyxobacter_dehalogenans_2CP~C
gi|501747731~Anaeromyxobacter_dehalogenans_2CP~1
gi|501517669~Anaeromyxobacter_sp__K
gi|1054749871~Bradyrhizobium_sp__BR_10303
gi|639175064~Bradyrhizobium_sp__Tv2a~2
gi|765026374~Bradyrhizobium_sp__LTSPM299
gi|494339954~Hydrocarboniphaga_effusa_AP103
gi|947273601~Devosia_sp__Root413D1
gi|941030482~Devosia_sp__A16
gi|948253666~Pseudolabrys_sp__Root1462
gi|737365335~Edaphobacter_aggregans
gi|651252822~Acidobacteria_bacterium_KBS_146
gi|551215482~Acidobacteriaceae_bacterium_TAA166
gi|522205344~Acidobacteriaceae_bacterium_KBS_89
gi|522200995~Acidobacteriaceae_bacterium_KBS_83
gi|575462436~Gemmatirosa_kalamazoonesis
gi|504032590~Granulicella_mallensis_MP5ACTX8
gi|1024575247~Oleiphilus_sp__HI0065
gi|1024566006~Oleiphilus_sp__HI0133
gi|1024691288~Oleiphilus_sp__HI0118
gi|511756659~Cycloclasticus_sp__PY97M
gi|504819317~Cycloclasticus_zancles_78~ME
gi|516642400~Teredinibacter_turnerae
gi|518430752~Teredinibacter_turnerae
gi|518433833~Teredinibacter_turnerae
gi|797194795~Alteromonadaceae_bacterium_Bs12
gi|951149143~Stenotrophomonas_terrae
gi|1011247791~Stenotrophomonas_sp__KCTC_12332
gi|1003982933~Stenotrophomonas_maltophilia
gi|951168741~Stenotrophomonas_panacihumi
gi|503925544~Pseudoxanthomonas_spadix_BD~a59
gi|655455896~Thermomonas_fusca
gi|1053891710~Xanthomonas_sp__SN8
gi|490189548~Xylella_fastidiosa
gi|968122446~Xylella_fastidiosa
gi|740437754~Xylella_fastidiosa_PLS229
gi|497265729~Xanthomonas_translucens_pv__graminis
gi|927875491~Xanthomonas_translucens_pv__arrhenatheri
gi|947169135~Pseudoxanthomonas_sp__Root65
gi|1011461081~Pseudoxanthomonas_mexicana
gi|640393247~Luteimonas_huabeiensis
gi|760008712~Luteibacter_sp__9133
gi|738159045~Luteibacter
gi|388429937~Rhodanobacter_sp__115
gi|950077762~Lysobacter_sp__Root916
gi|950051039~Lysobacter_sp__Root983
gi|675590098~Arenimonas_metalli_CF5~1
gi|736081425~Arenimonas_metalli
gi|551350022~Arenimonas_oryziterrae_DSM_21050_=_YC6267
gi|1027737399~Dyella_thiooxydans
gi|516032614~Dyella_ginsengisoli
gi|494774561~Rhodanobacter_denitrificans
gi|518293414~Dyella_japonica_A8
gi|517803213~Rudaea_cellulosilytica
gi|490242539~Cardiobacterium_hominis_ATCC_15826
gi|1047198079|emb|SAM71913_1|~Cardiobacterium_hominis
gi|494042346~Cardiobacterium_valvarum_F0432
gi|506260858~Kangiella_koreensis_DSM_16069
gi|517454908~Kangiella_aquimarina
gi|817505075~Kangiella_geojedonensis
gi|906867254~Wenzhouxiangella_marina
gi|499788640~Saccharophagus_degradans_2~40
gi|495565718~Methylophaga_thiooxydans_DMS010
gi|750294736~Methylophaga_aminisulfidivorans
gi|504517178~Methylophaga_frappieri
gi|931478679~Gammaproteobacteria_bacterium_SG8_31
gi|1041526094~Woeseia_oceani
gi|1055252731~Steroidobacter_denitrificans
gi|929752125~Sphaeroforma_arctica_JP610
gi|190683040~Oxyrrhis_marina
gi|397637095~Thalassiosira_oceanica
gi|223992877~Thalassiosira_pseudonana_CCMP1335
gi|397627193~Thalassiosira_oceanica
gi|157093361~Noctiluca_scintillans
gi|676396333~Aureococcus_anophagefferens
gi|551613651~Emiliania_huxleyi_CCMP1516
gi|77024089~Isochrysis_galbana
gi|551555217~Emiliania_huxleyi_CCMP1516
gi|693499987|emb|CEF98039_1|~Ostreococcus_tauri
gi|308804219~Ostreococcus_tauri
gi|145346654~Ostreococcus_lucimarinus_CCE9901
gi|303284187~Micromonas_pusilla_CCMP1545
gi|255082900~Micromonas_commoda
gi|612386589~Bathycoccus_prasinos
gi|77024155~Karlodinium_veneficum
gi|157093121~Karlodinium_veneficum
0.1
Bacteria
Eukaryota
SAG
NA

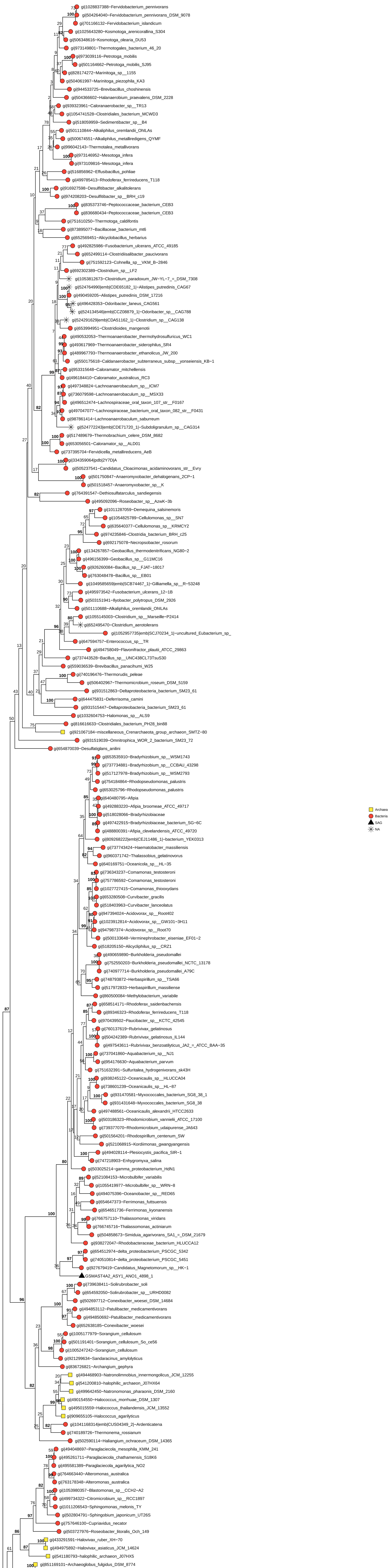
Archaea
Bacteria
SAG
NA
0.1

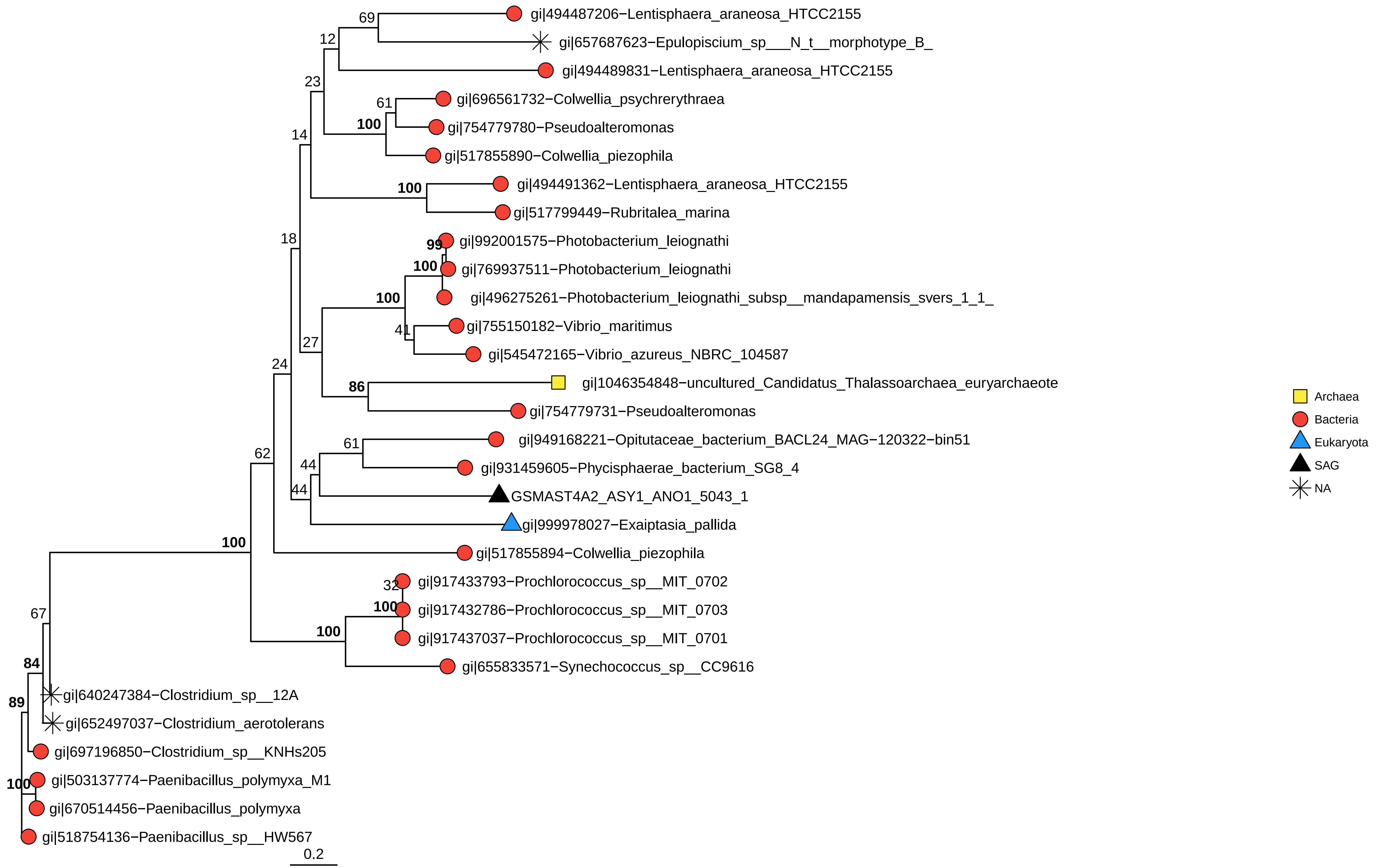

gi|494487206−Lentisphaera_araneosa_HTCC2155
gi|657687623−Epulopiscium_sp___N_t__morphotype_B_
gi|494489831−Lentisphaera_araneosa_HTCC2155
gi|696561732−Colwellia_psychrerythraea
gi|754779780−Pseudoalteromonas
gi|517855890−Colwellia_piezophila
gi|494491362−Lentisphaera_araneosa_HTCC2155
gi|517799449−Rubritalea_marina
gi|992001575−Photobacterium_leiognathi
gi|769937511−Photobacterium_leiognathi
gi|496275261−Photobacterium_leiognathi_subsp__mandapamensis_svers_1_1_
gi|755150182−Vibrio_maritimus
gi|545472165−Vibrio_azureus_NBRC_104587
gi|1046354848−uncultured_Candidatus_Thalassoarchaea_euryarchaeote
gi|754779731−Pseudoalteromonas
gi|949168221−Opitutaceae_bacterium_BACL24_MAG−120322−bin51
gi|931459605−Phycisphaerae_bacterium_SG8_4
GSMAST4A2_ASY1_ANO1_5043_1
gi|999978027−Exaiptasia_pallida
gi|517855894−Colwellia_piezophila
gi|917433793−Prochlorococcus_sp__MIT_0702
gi|917432786−Prochlorococcus_sp__MIT_0703
gi|917437037−Prochlorococcus_sp__MIT_0701
gi|655833571−Synechococcus_sp__CC9616
gi|640247384−Clostridium_sp__12A
gi|652497037−Clostridium_aerotolerans
gi|697196850−Clostridium_sp__KNHs205
gi|503137774−Paenibacillus_polymyxa_M1
gi|670514456−Paenibacillus_polymyxa
gi|518754136−Paenibacillus_sp__HW567
69
12
23
61
100
14
100
18
99
100
100
41
27
86
24
61
44
44
62
100
32
100
100
67
84
89
100
0.2
Archaea
Bacteria
Eukaryota
SAG
NA

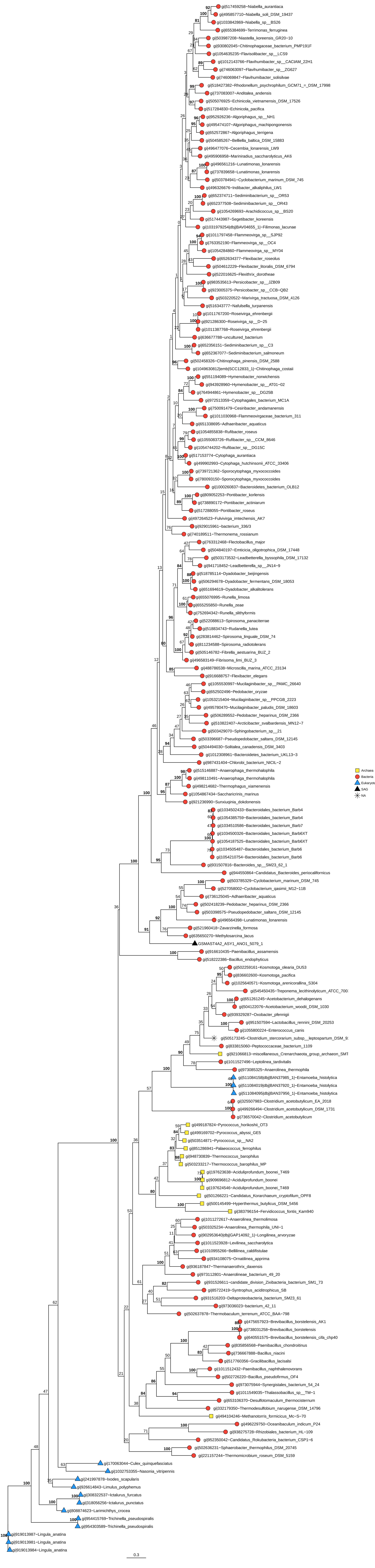
Archaea
Bacteria
Eukaryota
SAG
NA
0.3

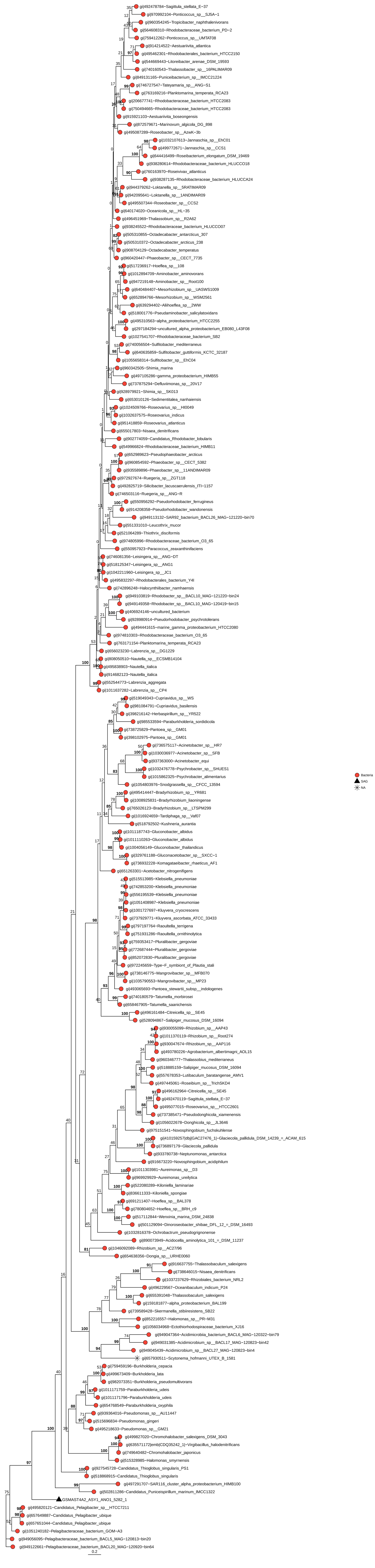

Bacteria
SAG
NA
0.2

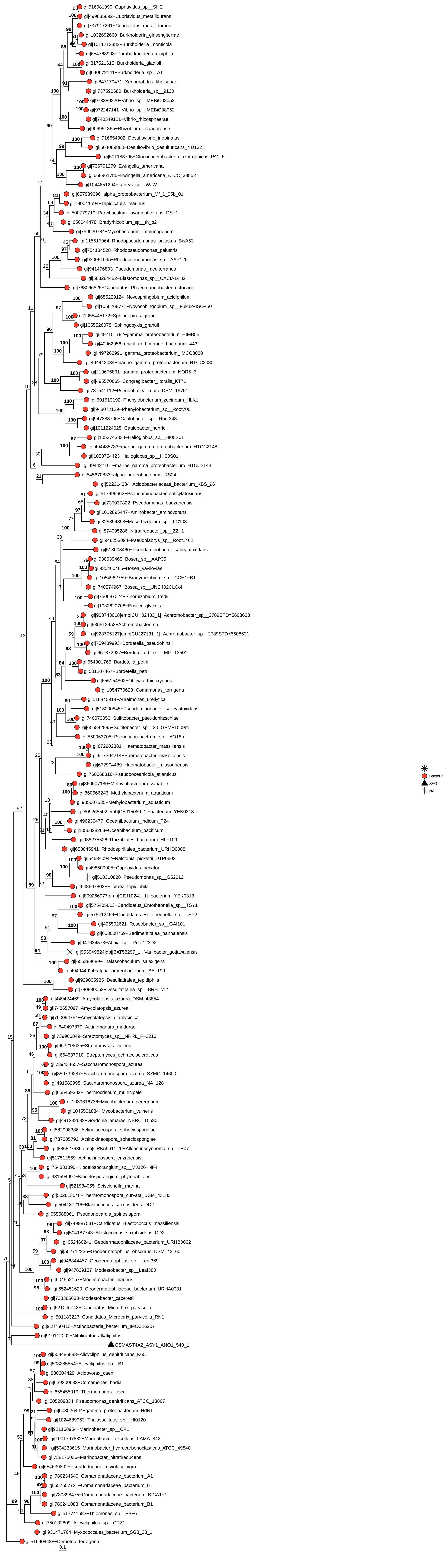
gi|516081990~Cupriavidus_sp__SHE
gi|499835892~Cupriavidus_metallidurans
gi|737917261~Cupriavidus_metallidurans
gi|1032692660~Burkholderia_ginsengiterrae
gi|1011212382~Burkholderia_monticola
gi|654768908~Paraburkholderia_oxyphila
gi|817521615~Burkholderia_gladioli
gi|640672141~Burkholderia_sp__A1
gi|847179471~Xenorhabdus_khoisanae
gi|737590680~Burkholderia_sp__9120
gi|973380220~Vibrio_sp__MEBiC08052
gi|972247141~Vibrio_sp__MEBiC08052
gi|740349121~Vibrio_rhizosphaerae
gi|906951865~Rhizobium_ecuadorense
gi|916654002~Desulfovibrio_inopinatus
gi|504089880~Desulfovibrio_desulfuricans_ND132
gi|501183795~Gluconacetobacter_diazotrophicus_PA1_5
gi|736791279~Ewingella_americana
gi|668961785~Ewingella_americana_ATCC_33852
gi|1044651294~Labrys_sp__WJW
gi|657939096~alpha_proteobacterium_Mf_1_05b_01
gi|780041594~Tepidicaulis_marinus
gi|500779719~Parvibaculum_lavamentivorans_DS~1
gi|656044478~Bradyrhizobium_sp__th_b2
gi|759020784~Mycobacterium_immunogenum
gi|115517964~Rhodopseudomonas_palustris_BisA53
gi|754184528~Rhodopseudomonas_palustris
gi|930061095~Rhodopseudomonas_sp__AAP120
gi|941476803~Pseudomonas_mediterranea
gi|563284482~Blastomonas_sp__CACIA14H2
gi|763066825~Candidatus_Phaeomarinobacter_ectocarpi
gi|655229124~Novosphingobium_acidiphilum
gi|1056268771~Novosphingobium_sp__Fuku2~ISO~50
gi|1055445172~Sphingopyxis_granuli
gi|1055526078~Sphingopyxis_granuli
gi|497101792~gamma_proteobacterium_HIMB55
gi|40062956~uncultured_marine_bacterium_443
gi|497262991~gamma_proteobacterium_IMCC3088
gi|494442034~marine_gamma_proteobacterium_HTCC2080
gi|219676891~gamma_proteobacterium_NOR5~3
gi|495570665~Congregibacter_litoralis_KT71
gi|737541112~Pseudohaliea_rubra_DSM_19751
gi|501513192~Phenylobacterium_zucineum_HLK1
gi|948072129~Phenylobacterium_sp__Root700
gi|947388706~Caulobacter_sp__Root343
gi|1011224025~Caulobacter_henricii
gi|1053743334~Halioglobus_sp__HI00S01
gi|494435733~marine_gamma_proteobacterium_HTCC2148
gi|1053754423~Halioglobus_sp__HI00S01
gi|494427161~marine_gamma_proteobacterium_HTCC2143
gi|545670833~alpha_proteobacterium_RS24
gi|522214384~Acidobacteriaceae_bacterium_KBS_96
gi|517999662~Pseudaminobacter_salicylatoxidans
gi|737037822~Pseudomonas_bauzanensis
gi|1012895447~Aminobacter_aminovorans
gi|825394898~Mesorhizobium_sp__LC103
gi|874095286~Nitratireductor_sp__ZZ~1
gi|948253094~Pseudolabrys_sp__Root1462
gi|518003460~Pseudaminobacter_salicylatoxidans
gi|930039465~Bosea_sp__AAP35
gi|930460465~Bosea_vaviloviae
gi|1054962759~Bradyrhizobium_sp__CCH1~B1
gi|740574967~Bosea_sp__UNC402CLCol
gi|750687024~Sinorhizobium_fredii
gi|1032620708~Ensifer_glycinis
gi|928743018|emb|CUK02433_1|~Achromobacter_sp__2789STDY5608633
gi|935512452~Achromobacter_sp_
gi|928775127|emb|CUJ27131_1|~Achromobacter_sp__2789STDY5608621
gi|759489993~Bordetella_pseudohinzii
gi|657872927~Bordetella_hinzii_LMG_13501
gi|654901765~Bordetella_petrii
gi|501207467~Bordetella_petrii
gi|655154802~Ottowia_thiooxydans
gi|1054770628~Comamonas_terrigena
gi|518840914~Aureimonas_ureilytica
gi|518000845~Pseudaminobacter_salicylatoxidans
gi|740073050~Sulfitobacter_pseudonitzschiae
gi|655842895~Sulfitobacter_sp__20_GPM~1509m
gi|550963705~Pseudochrobactrum_sp__AO18b
gi|672902381~Haematobacter_massiliensis
gi|917304214~Haematobacter_massiliensis
gi|672904489~Haematobacter_missouriensis
gi|760068816~Pseudooceanicola_atlanticus
gi|860507180~Methylobacterium_variabile
gi|860566246~Methylobacterium_aquaticum
gi|985607535~Methylobacterium_aquaticum
gi|809265502|emb|CEJ15089_1|~bacterium_YEK0313
gi|496230477~Oceanibaculum_indicum_P24
gi|1056028263~Oceanibaculum_pacificum
gi|938275526~Rhizobiales_bacterium_HL~109
gi|653045941~Rhodospirillales_bacterium_URHD0088
gi|546340942~Ralstonia_pickettii_DTP0602
gi|498509905~Cupriavidus_necator
gi|510310828~Pseudomonas_sp__G52012
gi|648607802~Elioraea_tepidiphila
gi|809266977|emb|CEJ10241_1|~bacterium_YEK0313
gi|575405613~Candidatus_Entotheonella_sp__TSY1
gi|575412454~Candidatus_Entotheonella_sp__TSY2
gi|495502621~Roseobacter_sp__GAI101
gi|653008769~Sedimentitalea_nanhaiensis
gi|947634573~Afipia_sp__Root123D2
gi|953949824|dbj|BAT58297_1|~Variibacter_gotjawalensis
gi|655389689~Thalassobaculum_salexigens
gi|494944924~alpha_proteobacterium_BAL199
gi|929005935~Desulfatitalea_tepidiphila
gi|780830053~Desulfatitalea_sp__BRH_c12
gi|449424469~Amycolatopsis_azurea_DSM_43854
gi|748657097~Amycolatopsis_azurea
gi|760094754~Amycolatopsis_rifamycinica
gi|640497879~Actinomadura_madurae
gi|739966849~Streptomyces_sp__NRRL_F~3213
gi|663218635~Streptomyces_violens
gi|664537010~Streptomyces_ochraceiscleroticus
gi|739434657~Saccharomonospora_azurea
gi|359739287~Saccharomonospora_azurea_SZMC_14600
gi|491582998~Saccharomonospora_azurea_NA~128
gi|655469382~Thermocrispum_municipale
gi|1039616736~Mycobacterium_peregrinum
gi|1045501834~Mycobacterium_vulneris
gi|491332682~Gordonia_amarae_NBRC_15530
gi|582998386~Actinokineospora_spheciospongiae
gi|737305792~Actinokineospora_spheciospongiae
gi|886827939|emb|CRK55611_1|~Alloactinosynnema_sp__L~07
gi|517512859~Actinokineospora_enzanensis
gi|754831890~Kibdelosporangium_sp__MJ126~NF4
gi|931594997~Kibdelosporangium_phytohabitans
gi|521984055~Sciscionella_marina
gi|502613548~Thermomonospora_curvata_DSM_43183
gi|504187216~Blastococcus_saxobsidens_DD2
gi|655588061~Pseudonocardia_spinosispora
gi|749987531~Candidatus_Blastococcus_massiliensis
gi|504187743~Blastococcus_saxobsidens_DD2
gi|652460241~Geodermatophilaceae_bacterium_URHB0062
gi|502712235~Geodermatophilus_obscurus_DSM_43160
gi|946844457~Geodermatophilus_sp__Leaf369
gi|947629137~Modestobacter_sp__Leaf380
gi|504552157~Modestobacter_marinus
gi|652451620~Geodermatophilaceae_bacterium_URHA0031
gi|738385633~Modestobacter_caceresii
gi|521046743~Candidatus_Microthrix_parvicella
gi|501183227~Candidatus_Microthrix_parvicella_RN1
gi|918750413~Actinobacteria_bacterium_IMCC26207
gi|919112002~Nitriliruptor_alkaliphilus
GSMAST4A2_ASY1_ANO1_540_1
gi|503486883~Alicycliphilus_denitrificans_K601
gi|503285554~Alicycliphilus_sp__B1
gi|930604429~Acidovorax_caeni
gi|639200633~Comamonas_badia
gi|655455019~Thermomonas_fusca
gi|505289834~Pseudomonas_denitrificans_ATCC_13867
gi|503026444~gamma_proteobacterium_HdN1
gi|1024689963~Thalassolituus_sp__HI0120
gi|921168954~Marinobacter_sp__CP1
gi|1001797882~Marinobacter_excellens_LAMA_842
gi|504233615~Marinobacter_hydrocarbonoclasticus_ATCC_49840
gi|738176036~Marinobacter_nitratireducens
gi|654639802~Pseudoduganella_violaceinigra
gi|780234640~Comamonadaceae_bacterium_A1
gi|657657721~Comamonadaceae_bacterium_H1
gi|780898475~Comamonadaceae_bacterium_BICA1~1
gi|780241083~Comamonadaceae_bacterium_B1
gi|517741683~Thiomonas_sp__FB~6
gi|750132809~Alicycliphilus_sp__CRZ1
gi|931471764~Myxococcales_bacterium_SG8_38_1
gi|516904438~Demetria_terragena
0.1
Bacteria
SAG
NA

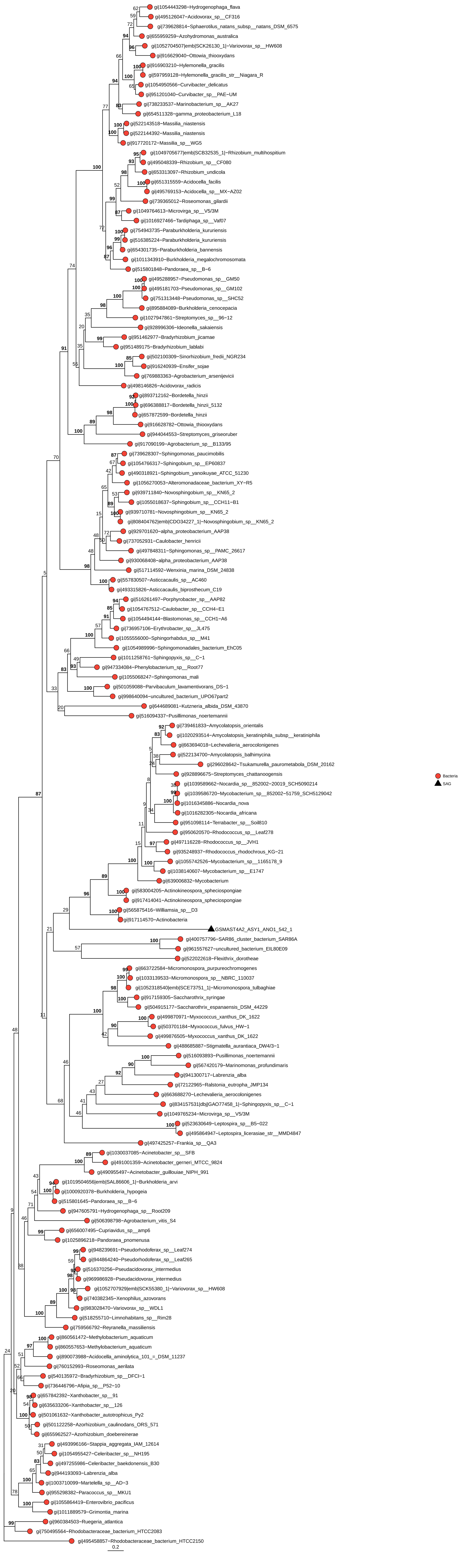

gi|1054443298~Hydrogenophaga_flava
gi|495126047~Acidovorax_sp__CF316
gi|739628814~Sphaerotilus_natans_subsp__natans_DSM_6575
gi|655959259~Azohydromonas_australica
gi|1052704507|emb|SCK26130_1|~Variovorax_sp__HW608
gi|916629040~Ottowia_thiooxydans
gi|916903210~Hylemonella_gracilis
gi|597959128~Hylemonella_gracilis_str__Niagara_R
gi|1054950566~Curvibacter_delicatus
gi|951201040~Curvibacter_sp__PAE~UM
gi|738233537~Marinobacterium_sp__AK27
gi|654511328~gamma_proteobacterium_L18
gi|522143518~Massilia_niastensis
gi|522144392~Massilia_niastensis
gi|917720172~Massilia_sp__WG5
gi|1049705677|emb|SCB32535_1|~Rhizobium_multihospitium
gi|495048339~Rhizobium_sp__CF080
gi|653313097~Rhizobium_undicola
gi|651315559~Acidocella_facilis
gi|495769153~Acidocella_sp__MX~AZ02
gi|739365012~Roseomonas_gilardii
gi|1049764613~Microvirga_sp__V5/3M
gi|1016927466~Tardiphaga_sp__Vaf07
gi|754943735~Paraburkholderia_kururiensis
gi|516385224~Paraburkholderia_kururiensis
gi|654301735~Paraburkholderia_bannensis
gi|1011343910~Burkholderia_megalochromosomata
gi|515801848~Pandoraea_sp__B~6
gi|495288957~Pseudomonas_sp__GM50
gi|495181703~Pseudomonas_sp__GM102
gi|751313448~Pseudomonas_sp__SHC52
gi|895884089~Burkholderia_cenocepacia
gi|1027947861~Streptomyces_sp__96~12
gi|928996306~Ideonella_sakaiensis
gi|951462977~Bradyrhizobium_jicamae
gi|951489175~Bradyrhizobium_lablabi
gi|502100309~Sinorhizobium_fredii_NGR234
gi|916240939~Ensifer_sojae
gi|769883363~Agrobacterium_arsenijevici
gi|498146826~Acidovorax_radicis
gi|893712162~Bordetella_hinzii
gi|696388817~Bordetella_hinzii_5132
gi|657872599~Bordetella_hinzii
gi|916628782~Ottowia_thiooxydans
gi|944044553~Streptomyces_griseoruber
gi|917090199~Agrobacterium_sp__B133/95
gi|739628307~Sphingomonas_paucimobilis
gi|1054766317~Sphingobium_sp__EP60837
gi|490318921~Sphingobium_yanoikuyae_ATCC_51230
gi|1056270053~Alteromonadaceae_bacterium_XY~R5
gi|939711840~Novosphingobium_sp__KN65_2
gi|1055018637~Sphingobium_sp__CCH11~B1
gi|939710781~Novosphingobium_sp__KN65_2
gi|808404762|emb|CDO34227_1|~Novosphingobium_sp__KN65_2
gi|929701620~alpha_proteobacterium_AAP38
gi|737052931~Caulobacter_henricii
gi|497848311~Sphingomonas_sp__PAMC_26617
gi|930068408~alpha_proteobacterium_AAP38
gi|517114592~Wenxinia_marina_DSM_24838
gi|557830507~Asticcacaulis_sp__AC460
gi|493315826~Asticcacaulis_biprosthecum_C19
gi|516261497~Porphyrobacter_sp__AAP82
gi|1054767512~Caulobacter_sp__CCH4~E1
gi|1054494144~Blastomonas_sp__CCH1~A6
gi|736957106~Erythrobacter_sp__JL475
gi|1055556000~Sphingorhabdus_sp__M41
gi|1054989996~Sphingomonadales_bacterium_EhC05
gi|1011258761~Sphingopyxis_sp__C~1
gi|947334084~Phenylobacterium_sp__Root77
gi|1055068247~Sphingomonas_mali
gi|501059088~Parvibaculum_lavamentivorans_DS~1
gi|998640094~uncultured_bacterium_UPO67part2
gi|644689081~Kutzneria_albida_DSM_43870
gi|516094337~Pusillimonas_noertemannii
gi|739461833~Amycolatopsis_orientalis
gi|1020293514~Amycolatopsis_keratiniphila_subsp__keratiniphila
gi|663694018~Lechevalieria_aerocolonigenes
gi|522134700~Amycolatopsis_balhimycina
gi|296028642~Tsukamurella_paurometabola_DSM_20162
gi|928896675~Streptomyces_chattanoogensis
gi|1039589662~Nocardia_sp__852002~20019_SCH5090214
gi|1039586720~Mycobacterium_sp__852002~51759_SCH5129042
gi|1016345886~Nocardia_nova
gi|1016282305~Nocardia_africana
gi|951098114~Terrabacter_sp__Soil810
gi|950620570~Rhodococcus_sp__Leaf278
gi|497116228~Rhodococcus_sp__JVH1
gi|935248937~Rhodococcus_rhodochrous_KG~21
gi|1055742526~Mycobacterium_sp__1165178_9
gi|1038140607~Mycobacterium_sp__E1747
gi|639006832~Mycobacterium
gi|583004205~Actinokineospora_spheciospongiae
gi|917414041~Actinokineospora_spheciospongiae
gi|565875416~Williamsia_sp__D3
gi|917114570~Actinobacteria
GSMAST4A2_ASY1_ANO1_542_1
gi|400757796~SAR86_cluster_bacterium_SAR86A
gi|961557627~uncultured_bacterium_EIL80E09
gi|522022618~Flexithrix_dorotheae
gi|663722584~Micromonospora_purpureochromogenes
gi|1033139533~Micromonospora_sp__NBRC_110037
gi|1052318540|emb|SCE73751_1|~Micromonospora_tulbaghiae
gi|917159305~Saccharothrix_syringae
gi|504915177~Saccharothrix_espanaensis_DSM_44229
gi|499870971~Myxococcus_xanthus_DK_1622
gi|503701184~Myxococcus_fulvus_HW~1
gi|499876505~Myxococcus_xanthus_DK_1622
gi|488685887~Stigmatella_aurantiaca_DW4/3~1
gi|516093893~Pusillimonas_noertemannii
gi|567420179~Marinomonas_profundimaris
gi|941300717~Labrenzia_alba
gi|72122965~Ralstonia_eutropha_JMP134
gi|663688270~Lechevalieria_aerocolonigenes
gi|834157531|dbj|GAO77458_1|~Sphingopyxis_sp__C~1
gi|1049765234~Microvirga_sp__V5/3M
gi|523630649~Leptospira_sp__B5~022
gi|495864947~Leptospira_licerasiae_str__MMD4847
gi|497425257~Frankia_sp__QA3
gi|1030037085~Acinetobacter_sp__SFB
gi|491001359~Acinetobacter_gerneri_MTCC_9824
gi|490955497~Acinetobacter_guillouiae_NIPH_991
gi|1019504656|emb|SAL86606_1|~Burkholderia_arvi
gi|1000920378~Burkholderia_hypogeia
gi|515801645~Pandoraea_sp__B~6
gi|947605791~Hydrogenophaga_sp__Root209
gi|506398798~Agrobacterium_vitis_S4
gi|656007495~Cupriavidus_sp__amp6
gi|1025896218~Pandoraea_pnomenusa
gi|948239691~Pseudorhodoferax_sp__Leaf274
gi|944864240~Pseudorhodoferax_sp__Leaf265
gi|516370256~Pseudacidovorax_intermedius
gi|969986928~Pseudacidovorax_intermedius
gi|1052707929|emb|SCK55380_1|~Variovorax_sp__HW608
gi|740382345~Xenophilus_azovorans
gi|983028470~Variovorax_sp__WDL1
gi|518255710~Limnohabitans_sp__Rim28
gi|759566792~Reyranella_massiliensis
gi|860561472~Methylobacterium_aquaticum
gi|860557563~Methylobacterium_aquaticum
gi|890073988~Acidocella_aminolytica_101_=_DSM_11237
gi|760152993~Roseomonas_aerilata
gi|540135972~Bradyrhizobium_sp__DFCI~1
gi|736446796~Afipia_sp__P52~10
gi|657842392~Xanthobacter_sp__91
gi|635633206~Xanthobacter_sp__126
gi|501061632~Xanthobacter_autotrophicus_Py2
gi|501122258~Azorhizobium_caulinodans_ORS_571
gi|655962527~Azorhizobium_doebereinerae
gi|493996166~Stappia_aggregata_IAM_12614
gi|1054955427~Celeribacter_sp__NH195
gi|497255986~Celeribacter_baekdonensis_B30
gi|944193093~Labrenzia_alba
gi|1003710099~Martelella_sp__AD~3
gi|955298382~Paracoccus_sp__MKU1
gi|1055864419~Enterovibrio_pacificus
gi|1011889579~Grimontia_marina
gi|960384503~Ruegeria_atlantica
gi|750495564~Rhodobacteraceae_bacterium_HTCC2083
gi|495458857~Rhodobacteraceae_bacterium_HTCC2150
0.2
Bacteria
SAG

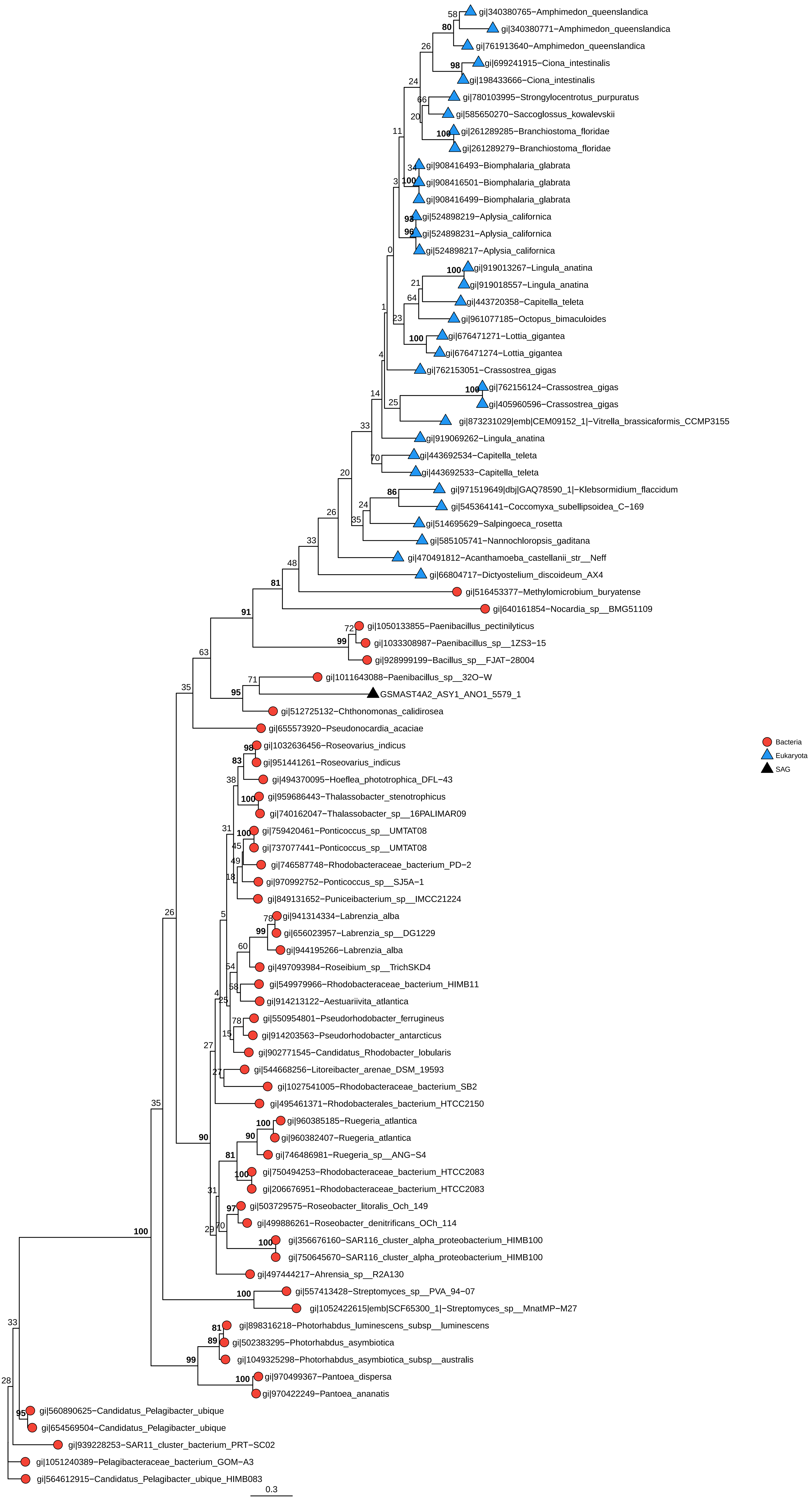
gi|340380765−Amphimedon_queenslandica
gi|340380771−Amphimedon_queenslandica
gi|761913640−Amphimedon_queenslandica
gi|699241915−Ciona_intestinalis
gi|198433666−Ciona_intestinalis
gi|780103995−Strongylocentrotus_purpuratus
gi|585650270−Saccoglossus_kowalevskii
gi|261289285−Branchiostoma_floridae
gi|261289279−Branchiostoma_floridae
gi|908416493−Biomphalaria_glabrata
gi|908416501−Biomphalaria_glabrata
gi|908416499−Biomphalaria_glabrata
gi|524898219−Aplysia_californica
gi|524898231−Aplysia_californica
gi|524898217−Aplysia_californica
gi|919013267−Lingula_anatina
gi|919018557−Lingula_anatina
gi|443720358−Capitella_teleta
gi|961077185−Octopus_bimaculoides
gi|676471271−Lottia_gigantea
gi|676471274−Lottia_gigantea
gi|762153051−Crassostrea_gigas
gi|762156124−Crassostrea_gigas
gi|405960596−Crassostrea_gigas
gi|873231029|emb|CEM09152_1|−Vitrella_brassicaformis_CCMP3155
gi|919069262−Lingula_anatina
gi|443692534−Capitella_teleta
gi|443692533−Capitella_teleta
gi|971519649|dbj|GAQ78590_1|−Klebsormidium_flaccidum
gi|545364141−Coccomyxa_subellipsoidea_C−169
gi|514695629−Salpingoeca_rosetta
gi|585105741−Nannochloropsis_gaditana
gi|470491812−Acanthamoeba_castellanii_str__Neff
gi|66804717−Dictyostelium_discoideum_AX4
gi|516453377−Methylomicrobium_buryatense
gi|640161854−Nocardia_sp__BMG51109
gi|1050133855−Paenibacillus_pectinilyticus
gi|1033308987−Paenibacillus_sp__1ZS3−15
gi|928999199−Bacillus_sp__FJAT−28004
gi|1011643088−Paenibacillus_sp__32O−W
GSMAST4A2_ASY1_ANO1_5579_1
gi|512725132−Chthonomonas_calidirosea
gi|655573920−Pseudonocardia_acaciae
gi|1032636456−Roseovarius_indicus
gi|951441261−Roseovarius_indicus
gi|494370095−Hoeflea_phototrophica_DFL−43
gi|959686443−Thalassobacter_stenotrophicus
gi|740162047−Thalassobacter_sp__16PALIMAR09
gi|759420461−Ponticoccus_sp__UMTAT08
gi|737077441−Ponticoccus_sp__UMTAT08
gi|746587748−Rhodobacteraceae_bacterium_PD−2
gi|970992752−Ponticoccus_sp__SJ5A−1
gi|849131652−Puniceibacterium_sp__IMCC21224
gi|941314334−Labrenzia_alba
gi|656023957−Labrenzia_sp__DG1229
gi|944195266−Labrenzia_alba
gi|497093984−Roseibium_sp__TrichSKD4
gi|549979966−Rhodobacteraceae_bacterium_HIMB11
gi|914213122−Aestuariivita_atlantica
gi|550954801−Pseudorhodobacter_ferrugineus
gi|914203563−Pseudorhodobacter_antarcticus
gi|902771545−Candidatus_Rhodobacter_lobularis
gi|544668256−Litoreibacter_arenae_DSM_19593
gi|1027541005−Rhodobacteraceae_bacterium_SB2
gi|495461371−Rhodobacterales_bacterium_HTCC2150
gi|960385185−Ruegeria_atlantica
gi|960382407−Ruegeria_atlantica
gi|746486981−Ruegeria_sp__ANG−S4
gi|750494253−Rhodobacteraceae_bacterium_HTCC2083
gi|206676951−Rhodobacteraceae_bacterium_HTCC2083
gi|503729575−Roseobacter_litoralis_Och_149
gi|499886261−Roseobacter_denitrificans_OCh_114
gi|356676160−SAR116_cluster_alpha_proteobacterium_HIMB100
gi|750645670−SAR116_cluster_alpha_proteobacterium_HIMB100
gi|497444217−Ahrensia_sp__R2A130
gi|557413428−Streptomyces_sp__PVA_94−07
gi|1052422615|emb|SCF65300_1|−Streptomyces_sp__MnatMP−M27
gi|898316218−Photorhabdus_luminescens_subsp__luminescens
gi|502383295−Photorhabdus_asymbiotica
gi|1049325298−Photorhabdus_asymbiotica_subsp__australis
gi|970499367−Pantoea_dispersa
gi|970422249−Pantoea_ananatis
gi|560890625−Candidatus_Pelagibacter_ubique
gi|654569504−Candidatus_Pelagibacter_ubique
gi|939228253−SAR11_cluster_bacterium_PRT−SC02
gi|1051240389−Pelagibacteraceae_bacterium_GOM−A3
gi|564612915−Candidatus_Pelagibacter_ubique_HIMB083
0.3
Bacteria
Eukaryota
SAG

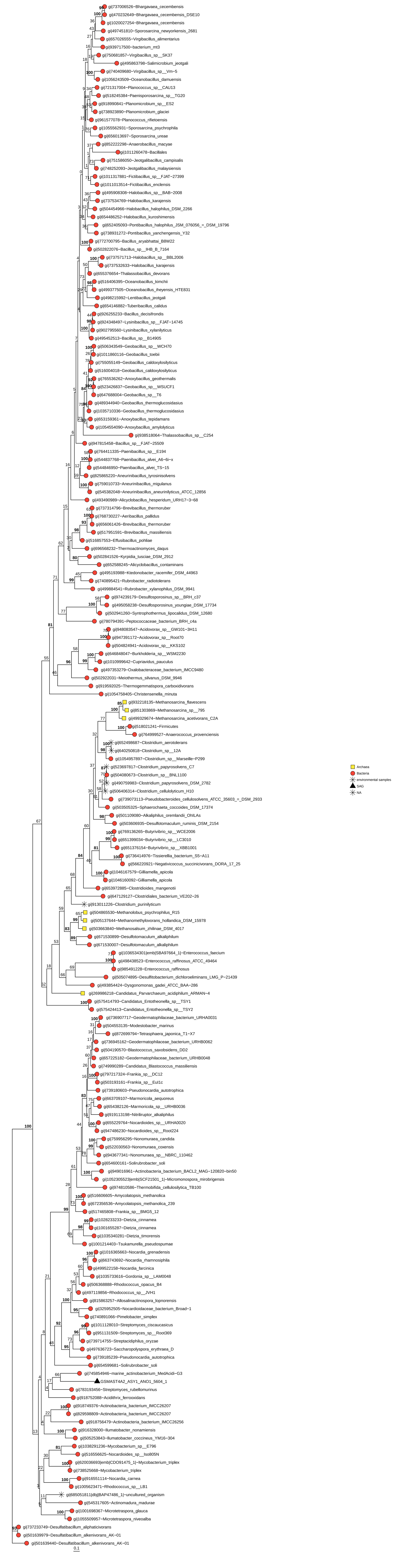

Archaea
Bacteria
environmental samples
SAG
NA
0.1

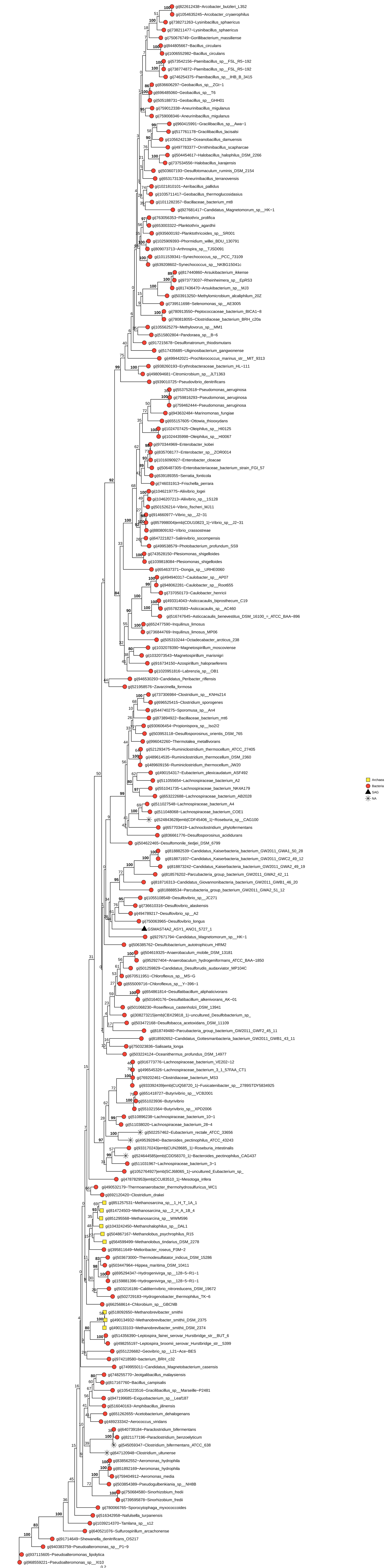

Archaea
Bacteria
SAG
NA
0.2

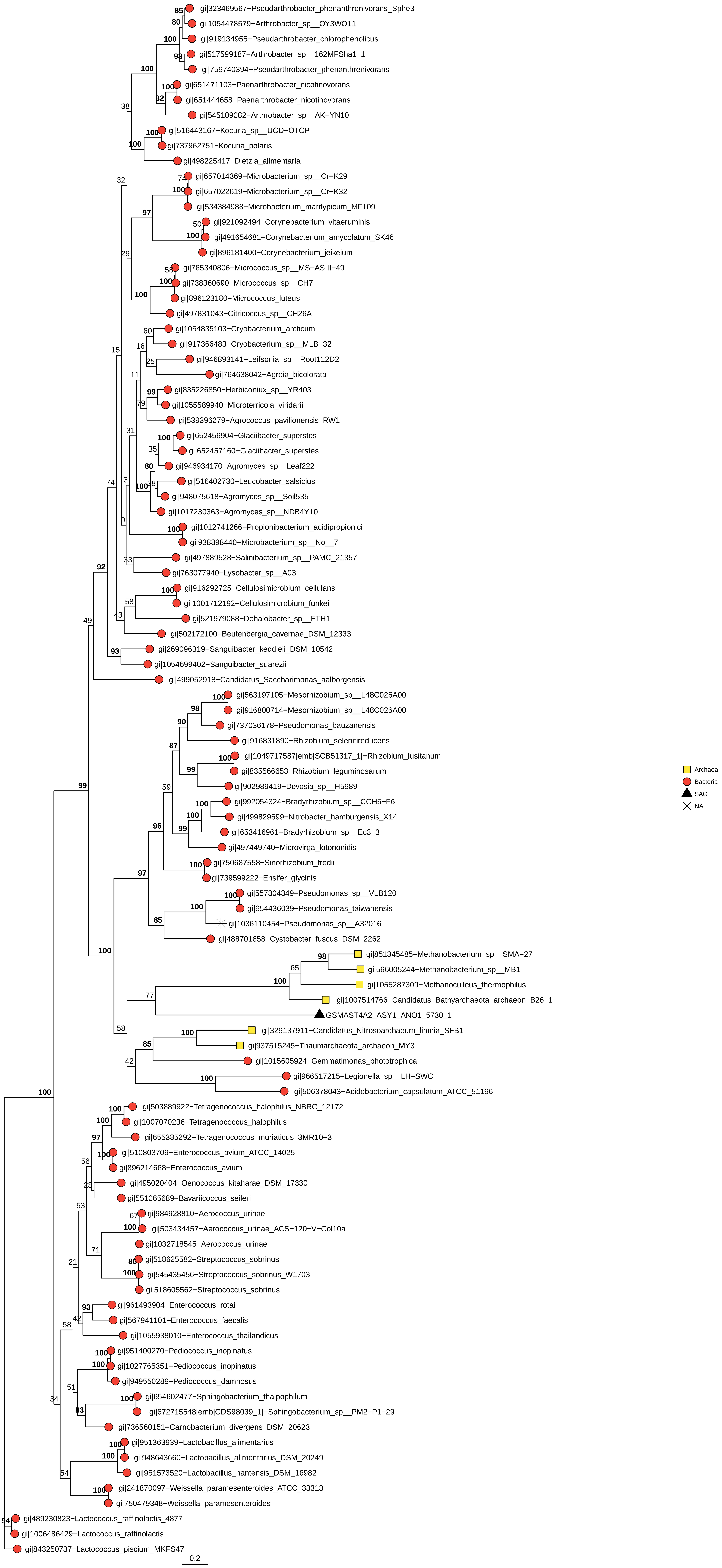
gi|323469567−Pseudarthrobacter_phenanthrenivorans_Sphe3
gi|1054478579−Arthrobacter_sp__OY3WO11
gi|919134955−Pseudarthrobacter_chlorophenolicus
gi|517599187−Arthrobacter_sp__162MFSha1_1
gi|759740394−Pseudarthrobacter_phenanthrenivorans
gi|651471103−Paenarthrobacter_nicotinovorans
gi|651444658−Paenarthrobacter_nicotinovorans
gi|545109082−Arthrobacter_sp__AK−YN10
gi|516443167−Kocuria_sp__UCD−OTCP
gi|737962751−Kocuria_polaris
gi|498225417−Dietzia_alimentaria
gi|657014369−Microbacterium_sp__Cr−K29
gi|657022619−Microbacterium_sp__Cr−K32
gi|534384988−Microbacterium_maritypicum_MF109
gi|921092494−Corynebacterium_vitaeruminis
gi|491654681−Corynebacterium_amycolatum_SK46
gi|896181400−Corynebacterium_jeikeium
gi|765340806−Micrococcus_sp__MS−ASIII−49
gi|738360690−Micrococcus_sp__CH7
gi|896123180−Micrococcus_luteus
gi|497831043−Citricoccus_sp__CH26A
gi|1054835103−Cryobacterium_arcticum
gi|917366483−Cryobacterium_sp__MLB−32
gi|946893141−Leifsonia_sp__Root112D2
gi|764638042−Agreia_bicolorata
gi|835226850−Herbiconiux_sp__YR403
gi|1055589940−Microterricola_viridarii
gi|539396279−Agrococcus_pavilionensis_RW1
gi|652456904−Glaciibacter_superstes
gi|652457160−Glaciibacter_superstes
gi|946934170−Agromyces_sp__Leaf222
gi|516402730−Leucobacter_salsicius
gi|948075618−Agromyces_sp__Soil535
gi|1017230363−Agromyces_sp__NDB4Y10
gi|1012741266−Propionibacterium_acidipropionici
gi|938898440−Microbacterium_sp__No__7
gi|497889528−Salinibacterium_sp__PAMC_21357
gi|763077940−Lysobacter_sp__A03
gi|916292725−Cellulosimicrobium_cellulans
gi|1001712192−Cellulosimicrobium_funkei
gi|521979088−Dehalobacter_sp__FTH1
gi|502172100−Beutenbergia_cavernae_DSM_12333
gi|269096319−Sanguibacter_keddieii_DSM_10542
gi|1054699402−Sanguibacter_suarezii
gi|499052918−Candidatus_Saccharimonas_aalborgensis
gi|563197105−Mesorhizobium_sp__L48C026A00
gi|916800714−Mesorhizobium_sp__L48C026A00
gi|737036178−Pseudomonas_bauzanensis
gi|916831890−Rhizobium_selenitireducens
gi|1049717587|emb|SCB51317_1|−Rhizobium_lusitanum
gi|835566653−Rhizobium_leguminosarum
gi|902989419−Devosia_sp__H5989
gi|992054324−Bradyrhizobium_sp__CCH5−F6
gi|499829699−Nitrobacter_hamburgensis_X14
gi|653416961−Bradyrhizobium_sp__Ec3_3
gi|497449740−Microvirga_lotononidis
gi|750687558−Sinorhizobium_fredii
gi|739599222−Ensifer_glycinis
gi|557304349−Pseudomonas_sp__VLB120
gi|654436039−Pseudomonas_taiwanensis
gi|1036110454−Pseudomonas_sp__A32016
gi|488701658−Cystobacter_fuscus_DSM_2262
gi|851345485−Methanobacterium_sp__SMA−27
gi|566005244−Methanobacterium_sp__MB1
gi|1055287309−Methanoculleus_thermophilus
gi|1007514766−Candidatus_Bathyarchaeota_archaeon_B26−1
GSMAST4A2_ASY1_ANO1_5730_1
gi|329137911−Candidatus_Nitrosoarchaeum_limnia_SFB1
gi|937515245−Thaumarchaeota_archaeon_MY3
gi|1015605924−Gemmatimonas_phototrophica
gi|966517215−Legionella_sp__LH−SWC
gi|506378043−Acidobacterium_capsulatum_ATCC_51196
gi|503889922−Tetragenococcus_halophilus_NBRC_12172
gi|1007070236−Tetragenococcus_halophilus
gi|655385292−Tetragenococcus_muriaticus_3MR10−3
gi|510803709−Enterococcus_avium_ATCC_14025
gi|896214668−Enterococcus_avium
gi|495020404−Oenococcus_kitaharae_DSM_17330
gi|551065689−Bavariicoccus_seileri
gi|984928810−Aerococcus_urinae
gi|503434457−Aerococcus_urinae_ACS−120−V−Col10a
gi|1032718545−Aerococcus_urinae
gi|518625582−Streptococcus_sobrinus
gi|545435456−Streptococcus_sobrinus_W1703
gi|518605562−Streptococcus_sobrinus
gi|961493904−Enterococcus_rotai
gi|567941101−Enterococcus_faecalis
gi|1055938010−Enterococcus_thailandicus
gi|951400270−Pediococcus_inopinatus
gi|1027765351−Pediococcus_inopinatus
gi|949550289−Pediococcus_damnosus
gi|654602477−Sphingobacterium_thalpophilum
gi|672715548|emb|CDS98039_1|−Sphingobacterium_sp__PM2−P1−29
gi|736560151−Carnobacterium_divergens_DSM_20623
gi|951363939−Lactobacillus_alimentarius
gi|948643660−Lactobacillus_alimentarius_DSM_20249
gi|951573520−Lactobacillus_nantensis_DSM_16982
gi|241870097−Weissella_paramesenteroides_ATCC_33313
gi|750479348−Weissella_paramesenteroides
gi|489230823−Lactococcus_raffinolactis_4877
gi|1006486429−Lactococcus_raffinolactis
gi|843250737−Lactococcus_piscium_MKFS47
0.2
Archaea
Bacteria
SAG
NA

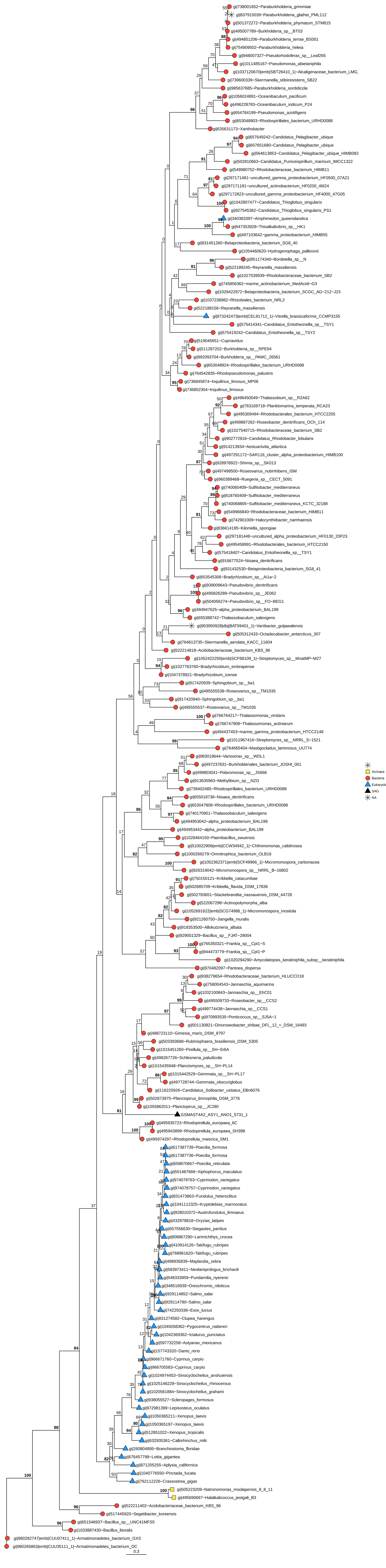
gi|738001652~Paraburkholderia_grimmiae
gi|837915039~Paraburkholderia_glathei_PML112
gi|501372272~Paraburkholderia_phymatum_STM815
gi|495007789~Burkholderia_sp__BT03
gi|494851206~Paraburkholderia_terrae_BS001
gi|754909502~Paraburkholderia_heleia
gi|948007327~Pseudorhodoferax_sp__Leaf265
gi|1011485167~Pseudomonas_abietaniphila
gi|1037120670|emb|SBT26410_1|~Alcaligenaceae_bacterium_LMG_
gi|739600339~Skermanella_stibiiresistens_SB22
gi|985637685~Paraburkholderia_sordidicola
gi|1056024891~Oceanibaculum_pacificum
gi|496228783~Oceanibaculum_indicum_P24
gi|654784199~Pseudomonas_azotifigens
gi|653048903~Rhodospirillales_bacterium_URHD0088
gi|635631173~Xanthobacter
gi|657649242~Candidatus_Pelagibacter_ubique
gi|657651680~Candidatus_Pelagibacter_ubique
gi|564613853~Candidatus_Pelagibacter_ubique_HIMB083
gi|502810663~Candidatus_Puniceispirillum_marinum_IMCC1322
gi|549980752~Rhodobacteraceae_bacterium_HIMB11
gi|297171481~uncultured_gamma_proteobacterium_HF0500_07A21
gi|297171181~uncultured_actinobacterium_HF0200_46I24
gi|297172823~uncultured_gamma_proteobacterium_HF4000_47G05
gi|1042807477~Candidatus_Thioglobus_singularis
gi|927545382~Candidatus_Thioglobus_singularis_PS1
gi|340383397~Amphimedon_queenslandica
gi|647353928~Thioalkalivibrio_sp__HK1
gi|497103642~gamma_proteobacterium_HIMB55
gi|931451260~Betaproteobacteria_bacterium_SG8_40
gi|1054460620~Hydrogenophaga_palleronii
gi|915174340~Bordetella_sp__N
gi|522189245~Reyranella_massiliensis
gi|1027539939~Rhodobacteraceae_bacterium_SB2
gi|745856362~marine_actinobacterium_MedAcidi~G3
gi|1028422972~Betaproteobacteria_bacterium_SCGC_AG~212~J23
gi|1037238982~Rhizobiales_bacterium_NRL2
gi|522188156~Reyranella_massiliensis
gi|873242473|emb|CEL91712_1|~Vitrella_brassicaformis_CCMP3155
gi|575414341~Candidatus_Entotheonella_sp__TSY1
gi|575419242~Candidatus_Entotheonella_sp__TSY2
gi|519045651~Cupriavidus
gi|511287202~Burkholderia_sp__RPE64
gi|992093704~Burkholderia_sp__PAMC_26561
gi|653048924~Rhodospirillales_bacterium_URHD0088
gi|764542835~Rhodopseudomonas_palustris
gi|736845874~Inquilinus_limosus_MP06
gi|736852304~Inquilinus_limosus
gi|496450049~Thalassobium_sp__R2A62
gi|763169718~Planktomarina_temperata_RCA23
gi|495309494~Rhodobacterales_bacterium_HTCC2255
gi|499887262~Roseobacter_denitrificans_OCh_114
gi|1027540715~Rhodobacteraceae_bacterium_SB2
gi|902772916~Candidatus_Rhodobacter_lobularis
gi|914213934~Aestuariivita_atlantica
gi|497291172~SAR116_cluster_alpha_proteobacterium_HIMB100
gi|928978922~Shimia_sp__SK013
gi|497499500~Roseovarius_nubinhibens_ISM
gi|960389468~Ruegeria_sp__CECT_5091
gi|740060409~Sulfitobacter_mediterraneus
gi|618760409~Sulfitobacter_mediterraneus
gi|740068805~Sulfitobacter_mediterraneus_KCTC_32188
gi|549966840~Rhodobacteraceae_bacterium_HIMB11
gi|742901009~Halocynthiibacter_namhaensis
gi|836614195~Kiloniella_spongiae
gi|297181448~uncultured_alpha_proteobacterium_HF0130_20P23
gi|495458991~Rhodobacterales_bacterium_HTCC2150
gi|575418407~Candidatus_Entotheonella_sp__TSY1
gi|916677524~Nisaea_denitrificans
gi|931432530~Betaproteobacteria_bacterium_SG8_41
gi|653545306~Bradyrhizobium_sp__Ai1a~2
gi|939009643~Pseudovibrio_denitrificans
gi|495826289~Pseudovibrio_sp__JE062
gi|504056274~Pseudovibrio_sp__FO~BEG1
gi|494947625~alpha_proteobacterium_BAL199
gi|655388742~Thalassobaculum_salexigens
gi|953950928|dbj|BAT59401_1|~Varibacter_gotjawalensis
gi|505312433~Octadecabacter_antarcticus_307
gi|764613735~Skermanella_aerolata_KACC_11604
gi|522214818~Acidobacteriaceae_bacterium_KBS_96
gi|1052422250|emb|SCF68109_1|~Streptomyces_sp__MnatMP~M27
gi|1027763760~Bradyrhizobium_embrapense
gi|1047378921~Bradyrhizobium_icense
gi|917420939~Sphingobium_sp__ba1
gi|495555538~Roseovarius_sp__TM1035
gi|917420940~Sphingobium_sp__ba1
gi|495555537~Roseovarius_sp__TM1035
gi|766764217~Thalassomonas_viridans
gi|766747908~Thalassomonas_actiniarum
gi|494437453~marine_gamma_proteobacterium_HTCC2148
gi|1011967416~Streptomyces_sp__NRRL_S~1521
gi|764665404~Mastigocladus_laminosus_UU774
gi|983019644~Variovorax_sp__WDL1
gi|497237831~Burkholderiales_bacterium_JOSHI_001
gi|499803041~Polaromonas_sp__JS666
gi|913635963~Methylibium_sp__NZG
gi|739402485~Rhodospirillales_bacterium_URHD0088
gi|655018736~Nisaea_denitrificans
gi|653047808~Rhodospirillales_bacterium_URHD0088
gi|740170951~Thalassobaculum_salexigens
gi|494953042~alpha_proteobacterium_BAL199
gi|494953442~alpha_proteobacterium_BAL199
gi|1028464150~Paenibacillus_swuensis
gi|510022906|emb|CCW34942_1|~Chthonomonas_calidirosea
gi|1000269279~Omnitrophica_bacterium_OLB16
gi|1052362371|emb|SCF49966_1|~Micromonospora_carbonacea
gi|926319042~Micromonospora_sp__NRRL_B~16802
gi|750150121~Kribbella_catacumbae
gi|502685709~Kribbella_flavida_DSM_17836
gi|502783651~Stackebrandtia_nassauensis_DSM_44728
gi|522067298~Actinopolymorpha_alba
gi|1052691622|emb|SCG74986_1|~Micromonospora_inositola
gi|921260750~Jiangella_muralis
gi|918353500~Allokutzneria_albata
gi|929001329~Bacillus_sp__FJAT~28004
gi|765350321~Frankia_sp__CpI1~S
gi|944473779~Frankia_sp__CpI1~P
gi|1020294290~Amycolatopsis_keratiniphila_subsp__keratiniphila
gi|970482097~Pantoea_dispersa
gi|938279654~Rhodobacteraceae_bacterium_HLUCCO18
gi|758004543~Jannaschia_aquimarina
gi|1032100843~Jannaschia_sp__EhC01
gi|495509733~Roseobacter_sp__CCS2
gi|499774438~Jannaschia_sp__CCS1
gi|970993535~Ponticoccus_sp__SJ5A~1
gi|501130821~Dinoroseobacter_shibae_DFL_12_=_DSM_16493
gi|488723110~Gimesia_maris_DSM_8797
gi|503393688~Rubinisphaera_brasiliensis_DSM_5305
gi|1015451260~Pirellula_sp__SH~Sr6A
gi|498267726~Schlesneria_paludicola
gi|1015435948~Planctomyces_sp__SH~PL14
gi|1015442529~Gemmata_sp__SH~PL17
gi|497728744~Gemmata_obscuriglobus
gi|116225926~Candidatus_Solibacter_usitatus_Ellin6076
gi|502873975~Planctopirus_limnophila_DSM_3776
gi|1055862011~Planctopirus_sp__JC280
GSMAST4A2_ASY1_ANO1_5731_1
gi|495935723~Rhodopirellula_europaea_6C
gi|495943899~Rhodopirellula_europaea_SH398
gi|495974297~Rhodopirellula_maiorica_SM1
gi|617387739~Poecilia_formosa
gi|617387736~Poecilia_formosa
gi|658870667~Poecilia_reticulata
gi|551487666~Xiphophorus_maculatus
gi|974078763~Cyprinodon_variegatus
gi|974078757~Cyprinodon_variegatus
gi|831473863~Fundulus_heteroclitus
gi|1041112325~Kryptolebias_marmoratus
gi|928010372~Austrofundulus_limnaeus
gi|432878818~Oryzias_latipes
gi|657556630~Stegastes_partitus
gi|808867290~Larimichthys_crocea
gi|410914126~Takifugu_rubripes
gi|768961620~Takifugu_rubripes
gi|498935839~Maylandia_zebra
gi|583973411~Neolamprologus_brichardi
gi|548333959~Pundamilia_nyererei
gi|348516939~Oreochromis_niloticus
gi|929114852~Salmo_salar
gi|929114780~Salmo_salar
gi|742250336~Esox_lucius
gi|831274582~Clupea_harengus
gi|1049268362~Pygocentrus_nattereri
gi|1042369362~Ictalurus_punctatus
gi|597732258~Astyanax_mexicanus
gi|157743320~Danio_rerio
gi|966671760~Cyprinus_carpio
gi|966705583~Cyprinus_carpio
gi|1024974453~Sinocyclocheilus_anshuiensis
gi|1025146228~Sinocyclocheilus_rhinocerous
gi|1020561884~Sinocyclocheilus_grahami
gi|938055527~Scleropages_formosus
gi|972981399~Lepisosteus_oculatus
gi|1050365211~Xenopus_laevis
gi|1050365197~Xenopus_laevis
gi|512851022~Xenopus_tropicalis
gi|632935361~Callorhinchus_milii
gi|260804895~Branchiostoma_floridae
gi|676457798~Lottia_gigantea
gi|871205255~Aplysia_californica
gi|1040776550~Pinctada_fucata
gi|762112226~Crassostrea_gigas
gi|505223209~Natronomonas_moolapensis_8_8_11
gi|495690697~Halalkalicoccus_jeotgali_B3
gi|522211402~Acidobacteriaceae_bacterium_KBS_96
gi|517445920~Segetibacter_koreensis
gi|651546937~Bacillus_sp__UNC41MFS5
gi|1033887430~Bacillus_litoralis
gi|980282747|emb|CUU07411_1|~Armatimonadetes_bacterium_GXS
gi|980265863|emb|CUU35111_1|~Armatimonadetes_bacterium_DC
0.3
Archaea
Bacteria
Eukaryota
SAG
NA

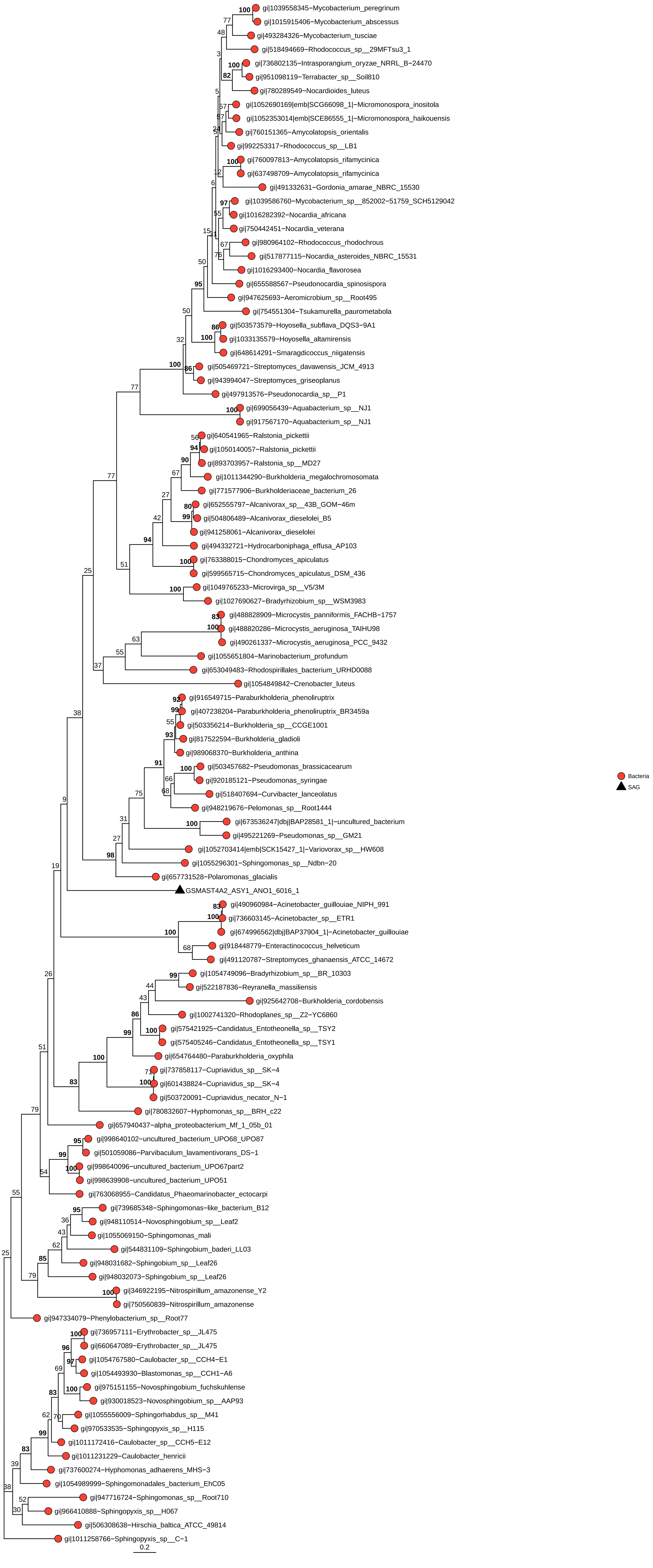
gi|1039558345−Mycobacterium_peregrinum
gi|1015915406−Mycobacterium_abscessus
gi|493284326−Mycobacterium_tusciae
gi|518494669−Rhodococcus_sp__29MFTsu3_1
gi|736802135−Intrasporangium_oryzae_NRRL_B−24470
gi|951098119−Terrabacter_sp__Soil810
gi|780289549−Nocardioides_luteus
gi|1052690169|emb|SCG66098_1|−Micromonospora_inositola
gi|1052353014|emb|SCE86555_1|−Micromonospora_haikouensis
gi|760151365−Amycolatopsis_orientalis
gi|992253317−Rhodococcus_sp__LB1
gi|760097813−Amycolatopsis_rifamycinica
gi|637498709−Amycolatopsis_rifamycinica
gi|491332631−Gordonia_amarae_NBRC_15530
gi|1039586760−Mycobacterium_sp__852002−51759_SCH5129042
gi|1016282392−Nocardia_africana
gi|750442451−Nocardia_veterana
gi|980964102−Rhodococcus_rhodochrous
gi|517877115−Nocardia_asteroides_NBRC_15531
gi|1016293400−Nocardia_flavorosea
gi|655588567−Pseudonocardia_spinosispora
gi|947625693−Aeromicrobium_sp__Root495
gi|754551304−Tsukamurella_paurometabola
gi|503573579−Hoyosella_subflava_DQS3−9A1
gi|1033135579−Hoyosella_altamirensis
gi|648614291−Smaragdicoccus_niigatensis
gi|505469721−Streptomyces_davawensis_JCM_4913
gi|943994047−Streptomyces_griseoplanus
gi|497913576−Pseudonocardia_sp__P1
gi|699056439−Aquabacterium_sp__NJ1
gi|917567170−Aquabacterium_sp__NJ1
gi|640541965−Ralstonia_pickettii
gi|1050140057−Ralstonia_pickettii
gi|893703957−Ralstonia_sp__MD27
gi|1011344290−Burkholderia_megalochromosomata
gi|771577906−Burkholderiaceae_bacterium_26
gi|652555797−Alcanivorax_sp__43B_GOM−46m
gi|504806489−Alcanivorax_dieselolei_B5
gi|941258061−Alcanivorax_dieselolei
gi|494332721−Hydrocarboniphaga_effusa_AP103
gi|763388015−Chondromyces_apiculatus
gi|599565715−Chondromyces_apiculatus_DSM_436
gi|1049765233−Microvirga_sp__V5/3M
gi|1027690627−Bradyrhizobium_sp__WSM3983
gi|488828909−Microcystis_panniformis_FACHB−1757
gi|488820286−Microcystis_aeruginosa_TAIHU98
gi|490261337−Microcystis_aeruginosa_PCC_9432
gi|1055651804−Marinobacterium_profundum
gi|653049483−Rhodospirillales_bacterium_URHD0088
gi|1054849842−Crenobacter_luteus
gi|916549715−Paraburkholderia_phenoliruptrix
gi|407238204−Paraburkholderia_phenoliruptrix_BR3459a
gi|503356214−Burkholderia_sp__CCGE1001
gi|817522594−Burkholderia_gladioli
gi|989068370−Burkholderia_anthina
gi|503457682−Pseudomonas_brassicacearum
gi|920185121−Pseudomonas_syringae
gi|518407694−Curvibacter_lanceolatus
gi|948219676−Pelomonas_sp__Root1444
gi|673536247|dbj|BAP28581_1|−uncultured_bacterium
gi|495221269−Pseudomonas_sp__GM21
gi|1052703414|emb|SCK15427_1|−Variovorax_sp__HW608
gi|1055296301−Sphingomonas_sp__Ndbn−20
gi|657731528−Polaromonas_glacialis
GSMAST4A2_ASY1_ANO1_6016_1
gi|490960984−Acinetobacter_guillouiae_NIPH_991
gi|736603145−Acinetobacter_sp__ETR1
gi|674996562|dbj|BAP37904_1|−Acinetobacter_guillouiae
gi|918448779−Enteractinococcus_helveticum
gi|491120787−Streptomyces_ghanaensis_ATCC_14672
gi|1054749096−Bradyrhizobium_sp__BR_10303
gi|522187836−Reyranella_massiliensis
gi|925642708−Burkholderia_cordobensis
gi|1002741320−Rhodoplanes_sp__Z2−YC6860
gi|575421925−Candidatus_Entotheonella_sp__TSY2
gi|575405246−Candidatus_Entotheonella_sp__TSY1
gi|654764480−Paraburkholderia_oxyphila
gi|737858117−Cupriavidus_sp__SK−4
gi|601438824−Cupriavidus_sp__SK−4
gi|503720091−Cupriavidus_necator_N−1
gi|780832607−Hyphomonas_sp__BRH_c22
gi|657940437−alpha_proteobacterium_Mf_1_05b_01
gi|998640102−uncultured_bacterium_UPO68_UPO87
gi|501059086−Parvibaculum_lavamentivorans_DS−1
gi|998640096−uncultured_bacterium_UPO67part2
gi|998639908−uncultured_bacterium_UPO51
gi|763068955−Candidatus_Phaeomarinobacter_ectocarpi
gi|739685348−Sphingomonas−like_bacterium_B12
gi|948110514−Novosphingobium_sp__Leaf2
gi|1055069150−Sphingomonas_mali
gi|544831109−Sphingobium_baderi_LL03
gi|948031682−Sphingobium_sp__Leaf26
gi|948032073−Sphingobium_sp__Leaf26
gi|346922195−Nitrospirillum_amazonense_Y2
gi|750560839−Nitrospirillum_amazonense
gi|947334079−Phenylobacterium_sp__Root77
gi|736957111−Erythrobacter_sp__JL475
gi|660647089−Erythrobacter_sp__JL475
gi|1054767580−Caulobacter_sp__CCH4−E1
gi|1054493930−Blastomonas_sp__CCH1−A6
gi|975151155−Novosphingobium_fuchskuhlense
gi|930018523−Novosphingobium_sp__AAP93
gi|1055556009−Sphingorhabdus_sp__M41
gi|970533535−Sphingopyxis_sp__H115
gi|1011172416−Caulobacter_sp__CCH5−E12
gi|1011231229−Caulobacter_henricii
gi|737600274−Hyphomonas_adhaerens_MHS−3
gi|1054989999−Sphingomonadales_bacterium_EhC05
gi|947716724−Sphingomonas_sp__Root710
gi|966410888−Sphingopyxis_sp__H067
gi|506308638−Hirschia_baltica_ATCC_49814
gi|1011258766−Sphingopyxis_sp__C−1
0.2
Bacteria
SAG

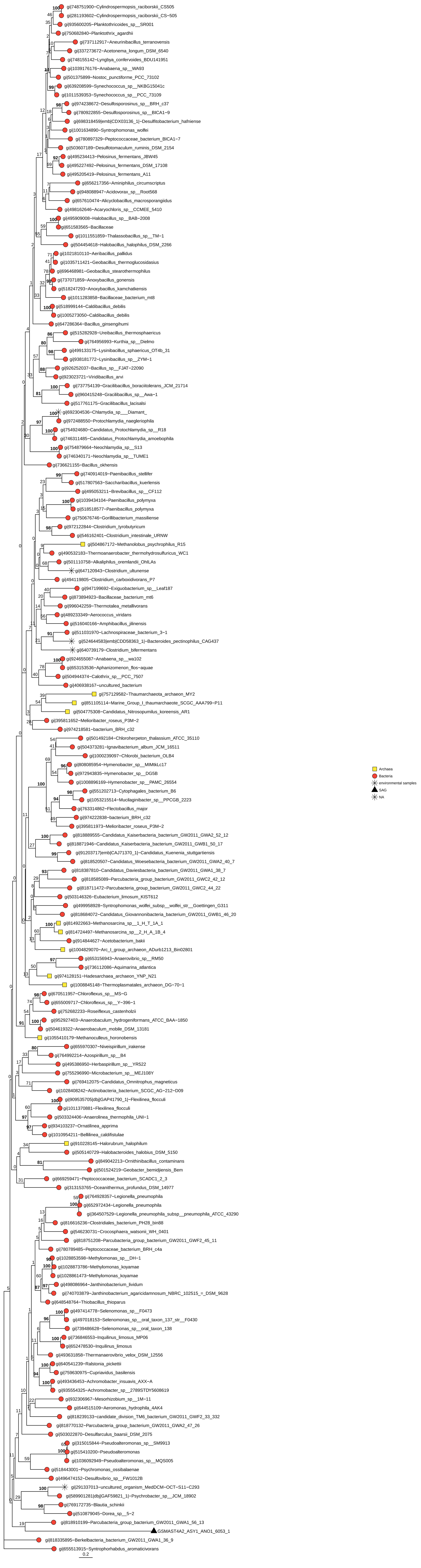

Archaea
Bacteria
environmental samples
SAG
NA
0.2

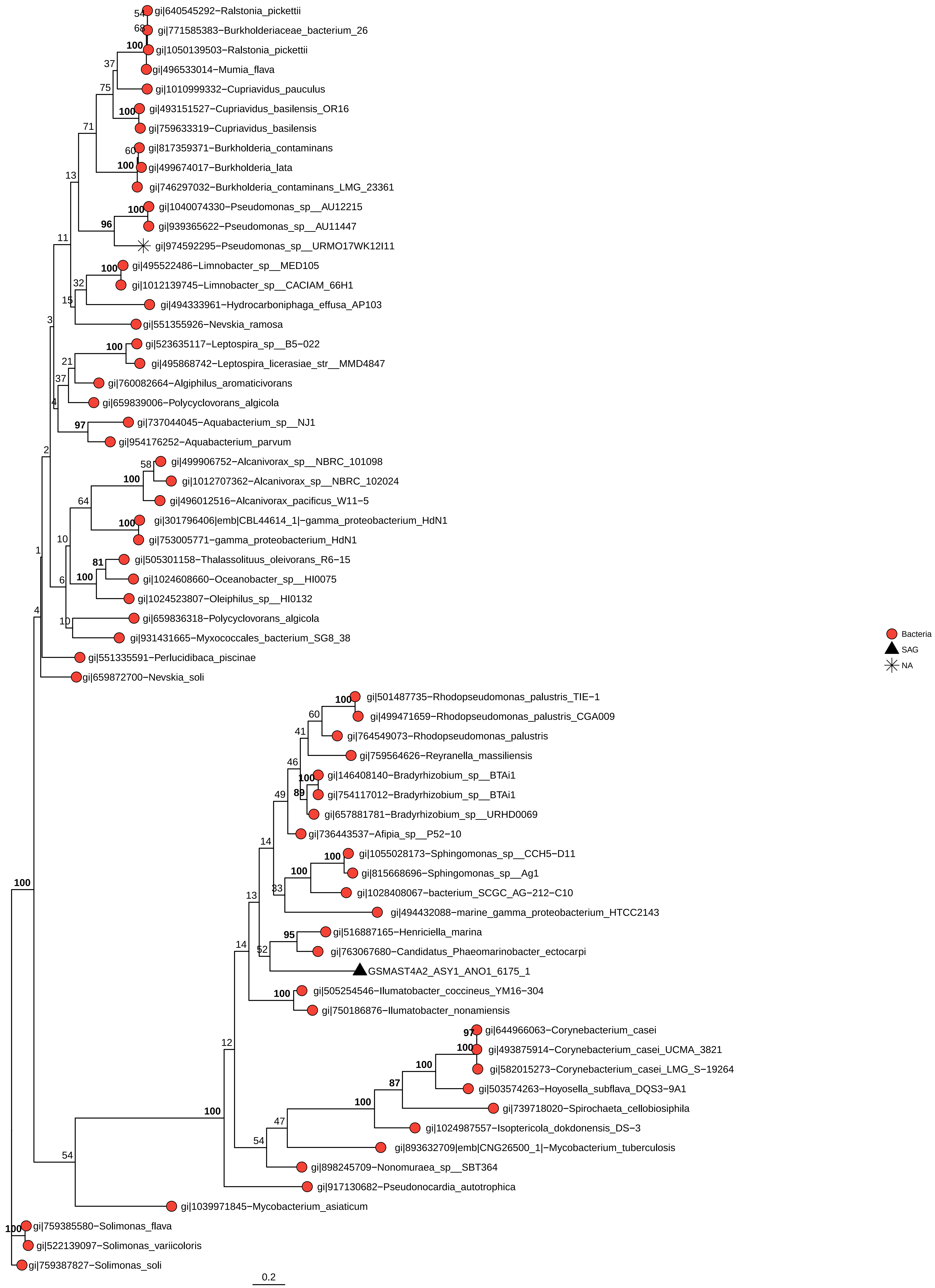
gi|640545292−Ralstonia_pickettii
gi|771585383−Burkholderiaceae_bacterium_26
gi|1050139503−Ralstonia_pickettii
gi|496533014−Mumia_flava
gi|1010999332−Cupriavidus_pauculus
gi|493151527−Cupriavidus_basilensis_OR16
gi|759633319−Cupriavidus_basilensis
gi|817359371−Burkholderia_contaminans
gi|499674017−Burkholderia_lata
gi|746297032−Burkholderia_contaminans_LMG_23361
gi|1040074330−Pseudomonas_sp__AU12215
gi|939365622−Pseudomonas_sp__AU11447
gi|974592295−Pseudomonas_sp__URMO17WK12I11
gi|495522486−Limnobacter_sp__MED105
gi|1012139745−Limnobacter_sp__CACIAM_66H1
gi|494333961−Hydrocarboniphaga_effusa_AP103
gi|551355926−Nevskia_ramosa
gi|523635117−Leptospira_sp__B5−022
gi|495868742−Leptospira_licerasiae_str__MMD4847
gi|760082664−Algiphilus_aromaticivorans
gi|659839006−Polycyclovorans_algicola
gi|737044045−Aquabacterium_sp__NJ1
gi|954176252−Aquabacterium_parvum
gi|499906752−Alcanivorax_sp__NBRC_101098
gi|1012707362−Alcanivorax_sp__NBRC_102024
gi|496012516−Alcanivorax_pacificus_W11−5
gi|301796406|emb|CBL44614_1|−gamma_proteobacterium_HdN1
gi|753005771−gamma_proteobacterium_HdN1
gi|505301158−Thalassolituus_oleivorans_R6−15
gi|1024608660−Oceanobacter_sp__HI0075
gi|1024523807−Oleiphilus_sp__HI0132
gi|659836318−Polycyclovorans_algicola
gi|931431665−Myxococcales_bacterium_SG8_38
gi|551335591−Perlucidibaca_piscinae
gi|659872700−Nevskia_soli
gi|501487735−Rhodopseudomonas_palustris_TIE−1
gi|499471659−Rhodopseudomonas_palustris_CGA009
gi|764549073−Rhodopseudomonas_palustris
gi|759564626−Reyranella_massiliensis
gi|146408140−Bradyrhizobium_sp__BTAi1
gi|754117012−Bradyrhizobium_sp__BTAi1
gi|657881781−Bradyrhizobium_sp__URHD0069
gi|736443537−Afipia_sp__P52−10
gi|1055028173−Sphingomonas_sp__CCH5−D11
gi|815668696−Sphingomonas_sp__Ag1
gi|1028408067−bacterium_SCGC_AG−212−C10
gi|494432088−marine_gamma_proteobacterium_HTCC2143
gi|516887165−Henriciella_marina
gi|763067680−Candidatus_Phaeomarinobacter_ectocarpi
GSMAST4A2_ASY1_ANO1_6175_1
gi|505254546−Ilumatobacter_coccineus_YM16−304
gi|750186876−Ilumatobacter_nonamiensis
gi|644966063−Corynebacterium_casei
gi|493875914−Corynebacterium_casei_UCMA_3821
gi|582015273−Corynebacterium_casei_LMG_S−19264
gi|503574263−Hoyosella_subflava_DQS3−9A1
gi|739718020−Spirochaeta_cellobiosiphila
gi|1024987557−Isoptericola_dokdonensis_DS−3
gi|893632709|emb|CNG26500_1|−Mycobacterium_tuberculosis
gi|898245709−Nonomuraea_sp__SBT364
gi|917130682−Pseudonocardia_autotrophica
gi|1039971845−Mycobacterium_asiaticum
gi|759385580−Solimonas_flava
gi|522139097−Solimonas_variicoloris
gi|759387827−Solimonas_soli
0.2
Bacteria
SAG
NA

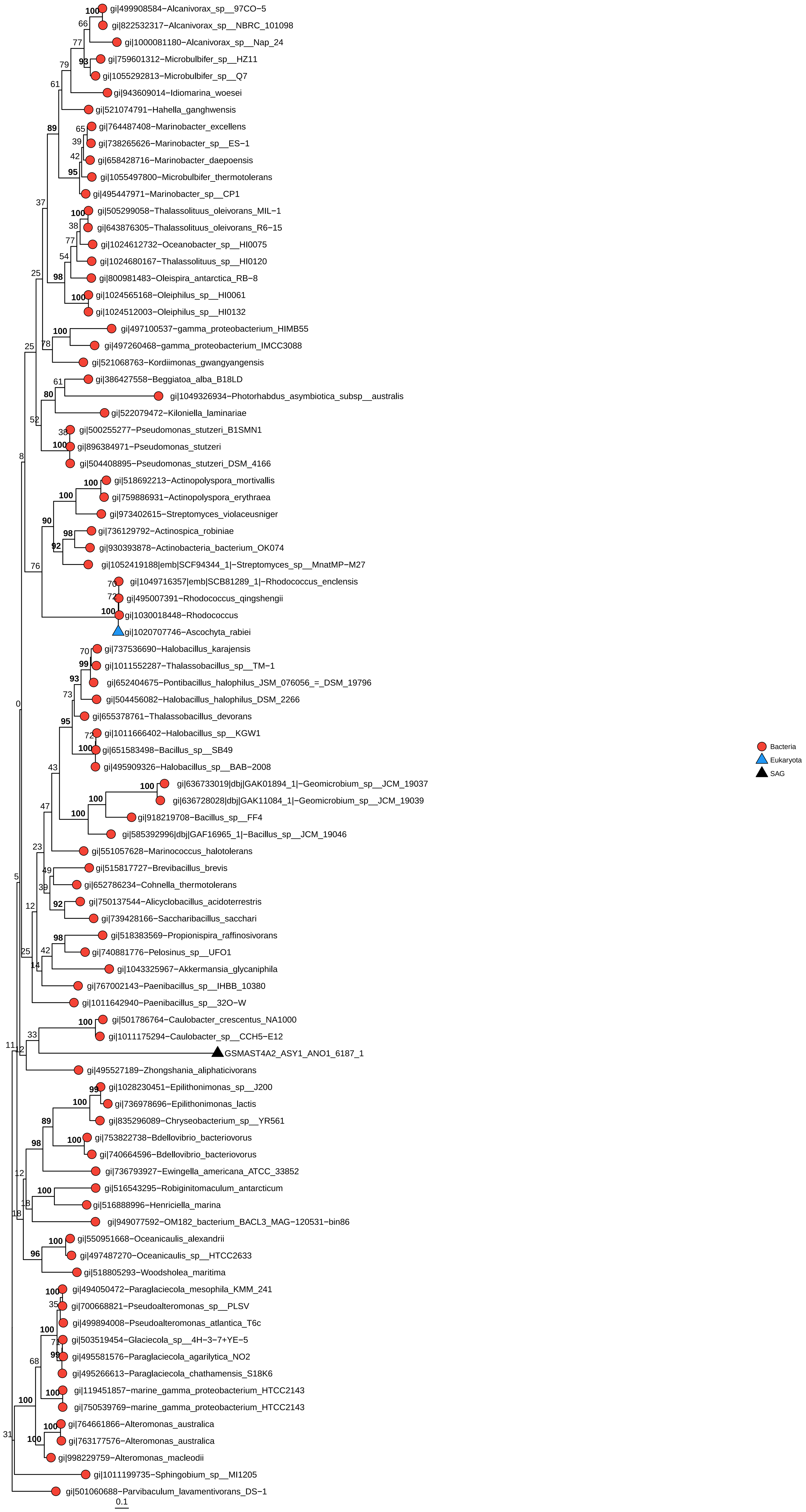
gi|499908584−Alcanivorax_sp__97CO−5
gi|822532317−Alcanivorax_sp__NBRC_101098
gi|1000081180−Alcanivorax_sp__Nap_24
gi|759601312−Microbulbifer_sp__HZ11
gi|1055292813−Microbulbifer_sp__Q7
gi|943609014−Idiomarina_woesei
gi|521074791−Hahella_ganghwensis
gi|764487408−Marinobacter_excellens
gi|738265626−Marinobacter_sp__ES−1
gi|658428716−Marinobacter_daepoensis
gi|1055497800−Microbulbifer_thermotolerans
gi|495447971−Marinobacter_sp__CP1
gi|505299058−Thalassolituus_oleivorans_MIL−1
gi|643876305−Thalassolituus_oleivorans_R6−15
gi|1024612732−Oceanobacter_sp__HI0075
gi|1024680167−Thalassolituus_sp__HI0120
gi|800981483−Oleispira_antarctica_RB−8
gi|1024565168−Oleiphilus_sp__HI0061
gi|1024512003−Oleiphilus_sp__HI0132
gi|497100537−gamma_proteobacterium_HIMB55
gi|497260468−gamma_proteobacterium_IMCC3088
gi|521068763−Kordiimonas_gwangyangensis
gi|386427558−Beggiatoa_alba_B18LD
gi|1049326934−Photorhabdus_asymbiotica_subsp__australis
gi|522079472−Kiloniella_laminariae
gi|500255277−Pseudomonas_stutzeri_B1SMN1
gi|896384971−Pseudomonas_stutzeri
gi|504408895−Pseudomonas_stutzeri_DSM_4166
gi|518692213−Actinopolyspora_mortivallis
gi|759886931−Actinopolyspora_erythraea
gi|973402615−Streptomyces_violaceusniger
gi|736129792−Actinospica_robiniae
gi|930393878−Actinobacteria_bacterium_OK074
gi|1052419188|emb|SCF94344_1|−Streptomyces_sp__MnatMP−M27
gi|1049716357|emb|SCB81289_1|−Rhodococcus_enclensis
gi|495007391−Rhodococcus_qingshengii
gi|1030018448−Rhodococcus
gi|1020707746−Ascochyta_rabiei
gi|737536690−Halobacillus_karajensis
gi|1011552287−Thalassobacillus_sp__TM−1
gi|652404675−Pontibacillus_halophilus_JSM_076056_=_DSM_19796
gi|504456082−Halobacillus_halophilus_DSM_2266
gi|655378761−Thalassobacillus_devorans
gi|1011666402−Halobacillus_sp__KGW1
gi|651583498−Bacillus_sp__SB49
gi|495909326−Halobacillus_sp__BAB−2008
gi|636733019|dbj|GAK01894_1|−Geomicrobium_sp__JCM_19037
gi|636728028|dbj|GAK11084_1|−Geomicrobium_sp__JCM_19039
gi|918219708−Bacillus_sp__FF4
gi|585392996|dbj|GAF16965_1|−Bacillus_sp__JCM_19046
gi|551057628−Marinococcus_halotolerans
gi|515817727−Brevibacillus_brevis
gi|652786234−Cohnella_thermotolerans
gi|750137544−Alicyclobacillus_acidoterrestris
gi|739428166−Saccharibacillus_sacchari
gi|518383569−Propionispira_raffinosivorans
gi|740881776−Pelosinus_sp__UFO1
gi|1043325967−Akkermansia_glycaniphila
gi|767002143−Paenibacillus_sp__IHBB_10380
gi|1011642940−Paenibacillus_sp__32O−W
gi|501786764−Caulobacter_crescentus_NA1000
gi|1011175294−Caulobacter_sp__CCH5−E12
GSMAST4A2_ASY1_ANO1_6187_1
gi|495527189−Zhongshania_aliphaticivorans
gi|1028230451−Epilithonimonas_sp__J200
gi|736978696−Epilithonimonas_lactis
gi|835296089−Chryseobacterium_sp__YR561
gi|753822738−Bdellovibrio_bacteriovorus
gi|740664596−Bdellovibrio_bacteriovorus
gi|736793927−Ewingella_americana_ATCC_33852
gi|516543295−Robiginitomaculum_antarcticum
gi|516888996−Henriciella_marina
gi|949077592−OM182_bacterium_BACL3_MAG−120531−bin86
gi|550951668−Oceanicaulis_alexandrii
gi|497487270−Oceanicaulis_sp__HTCC2633
gi|518805293−Woodsholea_maritima
gi|494050472−Paraglaciecola_mesophila_KMM_241
gi|700668821−Pseudoalteromonas_sp__PLSV
gi|499894008−Pseudoalteromonas_atlantica_T6c
gi|503519454−Glaciecola_sp__4H−3−7+YE−5
gi|495581576−Paraglaciecola_agarilytica_NO2
gi|495266613−Paraglaciecola_chathamensis_S18K6
gi|119451857−marine_gamma_proteobacterium_HTCC2143
gi|750539769−marine_gamma_proteobacterium_HTCC2143
gi|764661866−Alteromonas_australica
gi|763177576−Alteromonas_australica
gi|998229759−Alteromonas_macleodii
gi|1011199735−Sphingobium_sp__MI1205
gi|501060688−Parvibaculum_lavamentivorans_DS−1
0.1
Bacteria
Eukaryota
SAG

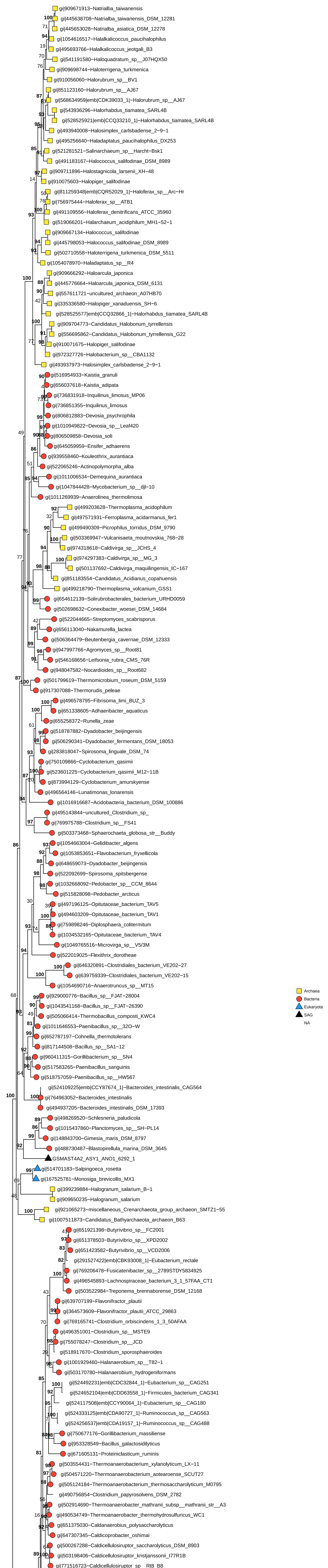
Archaea
Bacteria
Eukaryota
SAG
NA

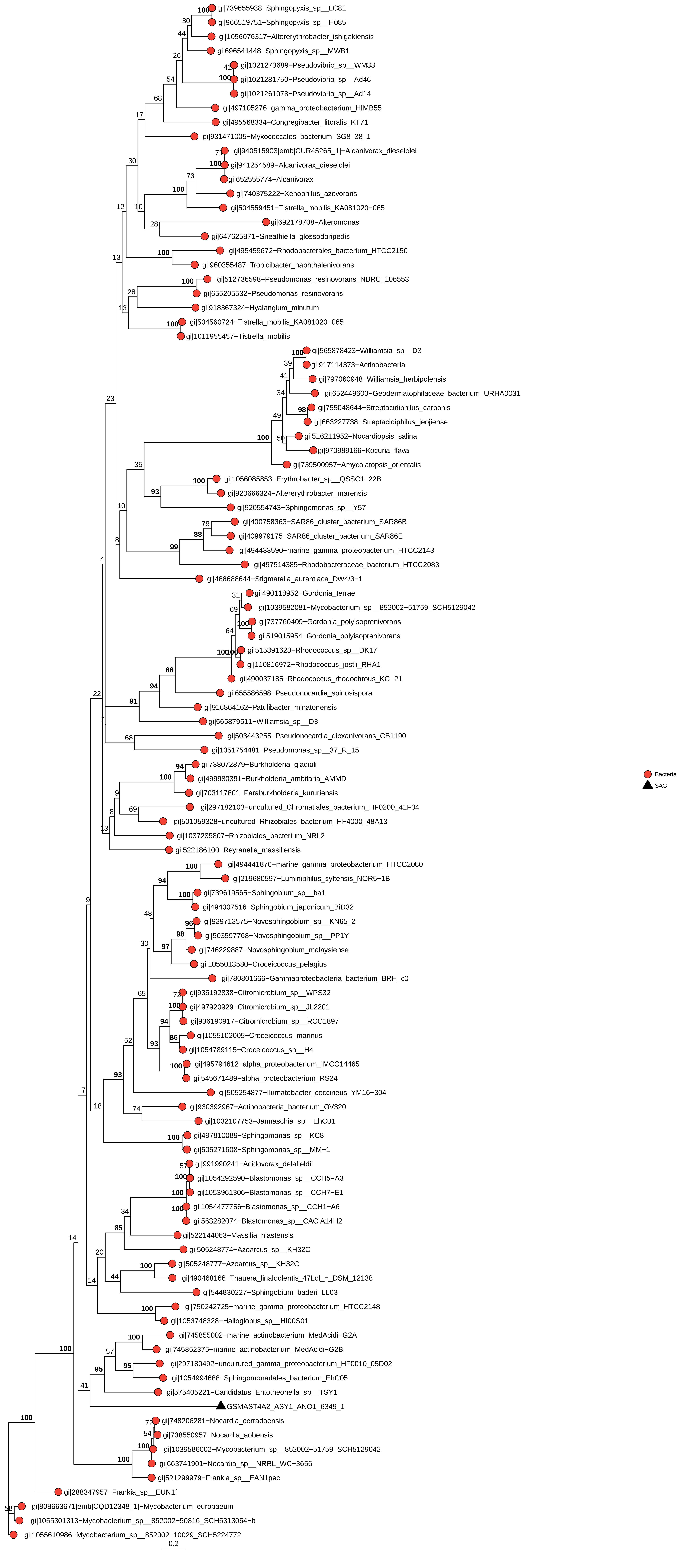
gi|739655938–Sphingopyxis_sp__LC81
gi|966519751–Sphingopyxis_sp__H085
gi|1056076317–Altererythrobacter_ishigakiensis
gi|696541448–Sphingopyxis_sp__MWB1
gi|1021273689–Pseudovibrio_sp__WM33
gi|1021281750–Pseudovibrio_sp__Ad46
gi|1021261078–Pseudovibrio_sp__Ad14
gi|497105276–gamma_proteobacterium_HIMB55
gi|495568334–Congregibacter_litoralis_KT71
gi|931471005–Myxococcales_bacterium_SG8_38_1
gi|940515903|emb|CUR45265_1|–Alcanivorax_dieselolei
gi|941254589–Alcanivorax_dieselolei
gi|652555774–Alcanivorax
gi|740375222–Xenophilus_azovorans
gi|504559451–Tistrella_mobilis_KA081020–065
gi|692178708–Alteromonas
gi|647625871–Sneathiella_glossodoripedis
gi|495459672–Rhodobacterales_bacterium_HTCC2150
gi|960355487–Tropicibacter_naphthalenivorans
gi|512736598–Pseudomonas_resinovorans_NBRC_106553
gi|655205532–Pseudomonas_resinovorans
gi|918367324–Hyalangium_minutum
gi|504560724–Tistrella_mobilis_KA081020–065
gi|1011955457–Tistrella_mobilis
gi|565878423–Williamsia_sp__D3
gi|917114373–Actinobacteria
gi|797060948–Williamsia_herbipolensis
gi|652449600–Geodermatophilaceae_bacterium_URHA0031
gi|755048644–Streptacidiphilus_carbonis
gi|663227738–Streptacidiphilus_jeojiense
gi|516211952–Nocardiopsis_salina
gi|970989166–Kocuria_flava
gi|739500957–Amycolatopsis_orientalis
gi|1056085853–Erythrobacter_sp__QSSC1–22B
gi|920666324–Altererythrobacter_marensis
gi|920554743–Sphingomonas_sp__Y57
gi|400758363–SAR86_cluster_bacterium_SAR86B
gi|409979175–SAR86_cluster_bacterium_SAR86E
gi|494433590–marine_gamma_proteobacterium_HTCC2143
gi|497514385–Rhodobacteraceae_bacterium_HTCC2083
gi|488688644–Stigmatella_aurantiaca_DW4/3–1
gi|490118952–Gordonia_terrae
gi|1039582081–Mycobacterium_sp__852002–51759_SCH5129042
gi|737760409–Gordonia_polyisoprenivorans
gi|519015954–Gordonia_polyisoprenivorans
gi|515391623–Rhodococcus_sp__DK17
gi|110816972–Rhodococcus_jostii_RHA1
gi|490037185–Rhodococcus_rhodochrous_KG–21
gi|655586598–Pseudonocardia_spinosispora
gi|916864162–Patulibacter_minatonensis
gi|565879511–Williamsia_sp__D3
gi|503443255–Pseudonocardia_dioxanivorans_CB1190
gi|1051754481–Pseudomonas_sp__37_R_15
gi|738072879–Burkholderia_gladioli
gi|499980391–Burkholderia_ambifaria_AMMD
gi|703117801–Paraburkholderia_kururiensis
gi|297182103–uncultured_Chromatiales_bacterium_HF0200_41F04
gi|501059328–uncultured_Rhizobiales_bacterium_HF4000_48A13
gi|1037239807–Rhizobiales_bacterium_NRL2
gi|522186100–Reyranella_massiliensis
gi|494441876–marine_gamma_proteobacterium_HTCC2080
gi|219680597–Luminiphilus_syltensis_NOR5–1B
gi|739619565–Sphingobium_sp__ba1
gi|494007516–Sphingobium_japonicum_BiD32
gi|939713575–Novosphingobium_sp__KN65_2
gi|503597768–Novosphingobium_sp__PP1Y
gi|746229887–Novosphingobium_malaysiense
gi|1055013580–Croceicoccus_pelagius
gi|780801666–Gammaproteobacteria_bacterium_BRH_c0
gi|936192838–Citromicrobium_sp__WPS32
gi|497920929–Citromicrobium_sp__JL2201
gi|936190917–Citromicrobium_sp__RCC1897
gi|1055102005–Croceicoccus_marinus
gi|1054789115–Croceicoccus_sp__H4
gi|495794612–alpha_proteobacterium_IMCC14465
gi|545671489–alpha_proteobacterium_RS24
gi|505254877–Ilumatobacter_coccineus_YM16–304
gi|930392967–Actinobacteria_bacterium_OV320
gi|1032107753–Jannaschia_sp__EhC01
gi|497810089–Sphingomonas_sp__KC8
gi|505271608–Sphingomonas_sp__MM–1
gi|991990241–Acidovorax_delafieldii
gi|1054292590–Blastomonas_sp__CCH5–A3
gi|1053961306–Blastomonas_sp__CCH7–E1
gi|1054477756–Blastomonas_sp__CCH1–A6
gi|563282074–Blastomonas_sp__CACIA14H2
gi|522144063–Massilia_niastensis
gi|505248774–Azoarcus_sp__KH32C
gi|505248777–Azoarcus_sp__KH32C
gi|490468166–Thauera_linaloolentis_47Lol_=_DSM_12138
gi|544830227–Sphingobium_baderi_LL03
gi|750242725–marine_gamma_proteobacterium_HTCC2148
gi|1053748328–Halioglobus_sp__HI00S01
gi|745855002–marine_actinobacterium_MedAcidi–G2A
gi|745852375–marine_actinobacterium_MedAcidi–G2B
gi|297180492–uncultured_gamma_proteobacterium_HF0010_05D02
gi|1054994688–Sphingomonadales_bacterium_EhC05
gi|575405221–Candidatus_Entotheonella_sp__TSY1
GSMAST4A2_ASY1_ANO1_6349_1
gi|748206281–Nocardia_cerradoensis
gi|738550957–Nocardia_aobensis
gi|1039586002–Mycobacterium_sp__852002–51759_SCH5129042
gi|663741901–Nocardia_sp__NRRL_WC–3656
gi|521299979–Frankia_sp__EAN1pec
gi|288347957–Frankia_sp__EUN1f
gi|808663671|emb|CQD12348_1|–Mycobacterium_europaeum
gi|1055301313–Mycobacterium_sp__852002–50816_SCH5313054–b
gi|1055610986–Mycobacterium_sp__852002–10029_SCH5224772
0.2
Bacteria
SAG

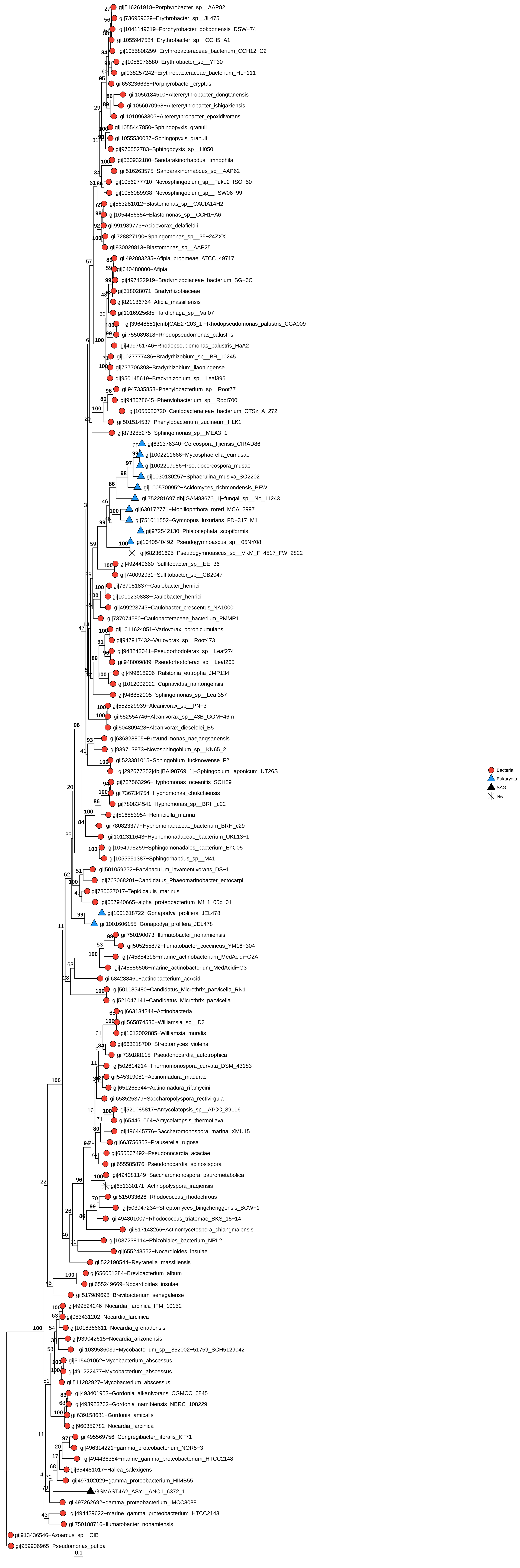

Bacteria
Eukaryota
SAG
NA
0.1

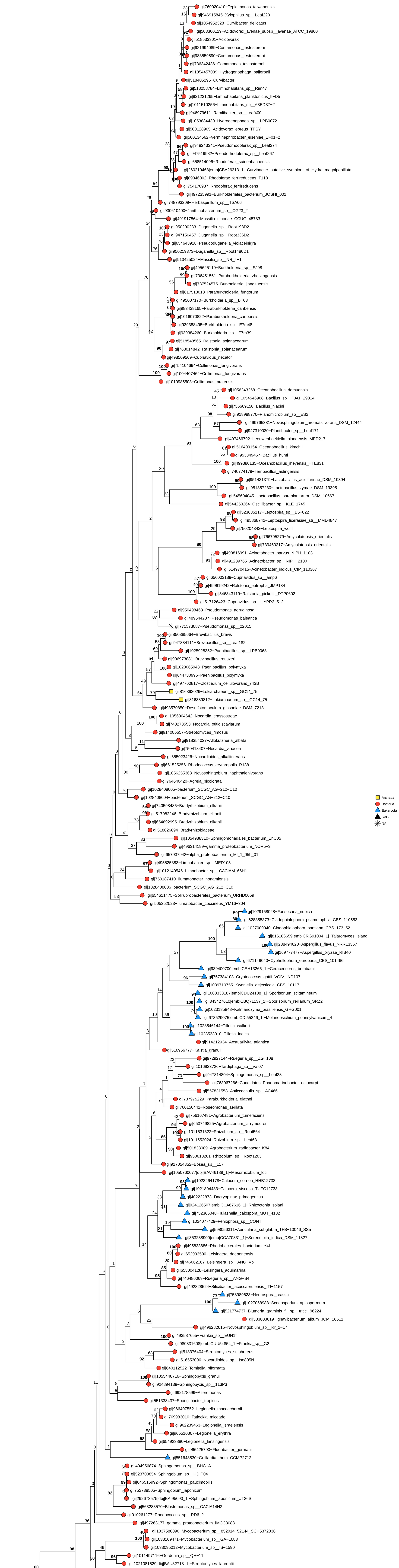

Archaea
Bacteria
Eukaryota
SAG
NA
0.3

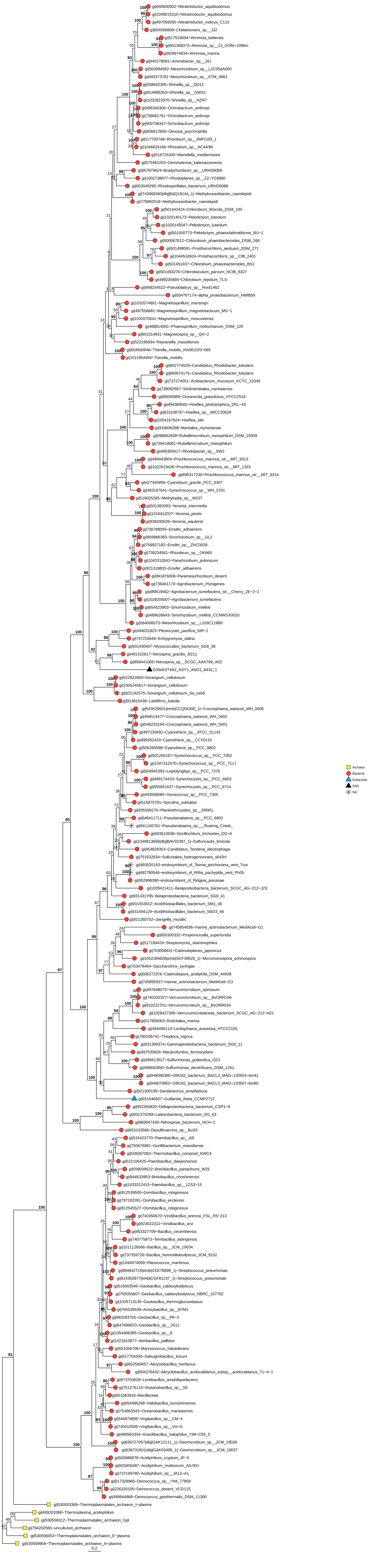
Archaea
Bacteria
Eukaryota
SAG
NA
0.2

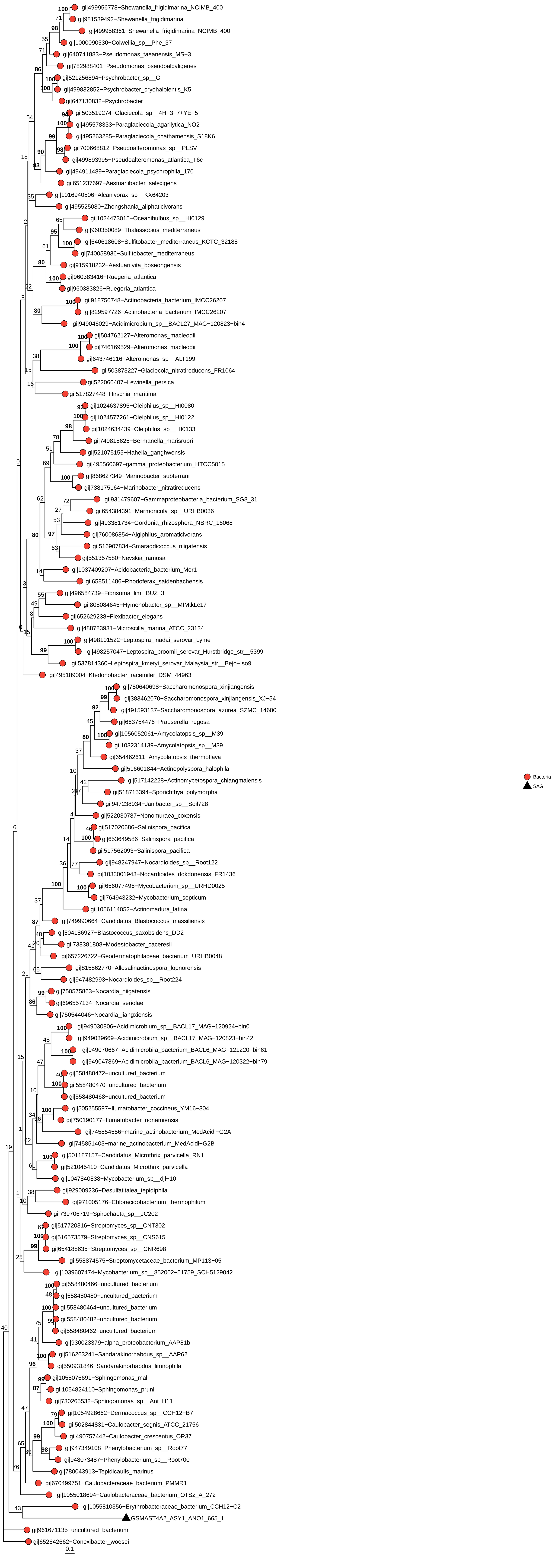
gi|499956778−Shewanella_frigidimarina_NCIMB_400
gi|981539492−Shewanella_frigidimarina
gi|499958361−Shewanella_frigidimarina_NCIMB_400
gi|1000090530−Colwellia_sp__Phe_37
gi|640741883−Pseudomonas_taeanensis_MS−3
gi|782988401−Pseudomonas_pseudoalcaligenes
gi|521256894−Psychrobacter_sp__G
gi|499832852−Psychrobacter_cryohalolentis_K5
gi|647130832−Psychrobacter
gi|503519274−Glaciecola_sp__4H−3−7+YE−5
gi|495578333−Paraglaciecola_agarilytica_NO2
gi|495263285−Paraglaciecola_chathamensis_S18K6
gi|700668812−Pseudoalteromonas_sp__PLSV
gi|499893995−Pseudoalteromonas_atlantica_T6c
gi|494911489−Paraglaciecola_psychrophila_170
gi|651237697−Aestuariibacter_salexigens
gi|1016940506−Alcanivorax_sp__KX64203
gi|495525080−Zhongshania_aliphaticivorans
gi|1024473015−Oceanibulbus_sp__HI0129
gi|960350089−Thalassobius_mediterraneus
gi|640618608−Sulfitobacter_mediterraneus_KCTC_32188
gi|740058936−Sulfitobacter_mediterraneus
gi|915918232−Aestuariivita_boseongensis
gi|960383416−Ruegeria_atlantica
gi|960383826−Ruegeria_atlantica
gi|918750748−Actinobacteria_bacterium_IMCC26207
gi|829597726−Actinobacteria_bacterium_IMCC26207
gi|949046029−Acidimicrobium_sp__BACL27_MAG−120823−bin4
gi|504762127−Alteromonas_macleodii
gi|746169529−Alteromonas_macleodii
gi|643746116−Alteromonas_sp__ALT199
gi|503873227−Glaciecola_nitratireducens_FR1064
gi|522060407−Lewinella_persica
gi|517827448−Hirschia_maritima
gi|1024637895−Oleiphilus_sp__HI0080
gi|1024577261−Oleiphilus_sp__HI0122
gi|1024634439−Oleiphilus_sp__HI0133
gi|749818625−Bermanella_marisrubri
gi|521075155−Hahella_ganghwensis
gi|495560697−gamma_proteobacterium_HTCC5015
gi|868627349−Marinobacter_subterrani
gi|738175164−Marinobacter_nitratireducens
gi|931479607−Gammaproteobacteria_bacterium_SG8_31
gi|654384391−Marmoricola_sp__URHB0036
gi|493381734−Gordonia_rhizosphera_NBRC_16068
gi|760086854−Algiphilus_aromaticivorans
gi|516907834−Smaragdicoccus_niigatensis
gi|551357580−Nevskia_ramosa
gi|1037409207−Acidobacteria_bacterium_Mor1
gi|658511486−Rhodoferax_saidenbachensis
gi|496584739−Fibrisoma_limi_BUZ_3
gi|808084645−Hymenobacter_sp__MIMtkLc17
gi|652629238−Flexibacter_elegans
gi|488783931−Microscilla_marina_ATCC_23134
gi|498101522−Leptospira_inadai_serovar_Lyme
gi|498257047−Leptospira_broomii_serovar_Hurstbridge_str__5399
gi|537814360−Leptospira_kmetyi_serovar_Malaysia_str__Bejo−Iso9
gi|495189004−Ktedonobacter_racemifer_DSM_44963
gi|750640698−Saccharomonospora_xinjiangensis
gi|383462070−Saccharomonospora_xinjiangensis_XJ−54
gi|491593137−Saccharomonospora_azurea_SZMC_14600
gi|663754476−Prauserella_rugosa
gi|1056052061−Amycolatopsis_sp__M39
gi|1032314139−Amycolatopsis_sp__M39
gi|654462611−Amycolatopsis_thermoflava
gi|516601844−Actinopolyspora_halophila
gi|517142228−Actinomycetospora_chiangmaiensis
gi|518715394−Sporichthya_polymorpha
gi|947238934−Janibacter_sp__Soil728
gi|522030787−Nonomuraea_coxensis
gi|517020686−Salinispora_pacifica
gi|653649586−Salinispora_pacifica
gi|517562093−Salinispora_pacifica
gi|948247947−Nocardioides_sp__Root122
gi|1033001943−Nocardioides_dokdonensis_FR1436
gi|656077496−Mycobacterium_sp__URHD0025
gi|764943232−Mycobacterium_septicum
gi|1056114052−Actinomadura_latina
gi|749990664−Candidatus_Blastococcus_massiliensis
gi|504186927−Blastococcus_saxobsidens_DD2
gi|738381808−Modestobacter_caceresii
gi|657226722−Geodermatophilaceae_bacterium_URHB0048
gi|815862770−Allosalinactinospora_lopnorensis
gi|947482993−Nocardioides_sp__Root224
gi|750575863−Nocardia_niigatensis
gi|696557134−Nocardia_seriolae
gi|750544046−Nocardia_jiangxiensis
gi|949030806−Acidimicrobium_sp__BACL17_MAG−120924−bin0
gi|949039669−Acidimicrobium_sp__BACL17_MAG−120823−bin42
gi|949070667−Acidimicrobiia_bacterium_BACL6_MAG−121220−bin61
gi|949047869−Acidimicrobiia_bacterium_BACL6_MAG−120322−bin79
gi|558480472−uncultured_bacterium
gi|558480470−uncultured_bacterium
gi|558480468−uncultured_bacterium
gi|505255597−Ilumatobacter_coccineus_YM16−304
gi|750190177−Ilumatobacter_nonamiensis
gi|745854556−marine_actinobacterium_MedAcidi−G2A
gi|745851403−marine_actinobacterium_MedAcidi−G2B
gi|501187157−Candidatus_Microthrix_parvicella_RN1
gi|521045410−Candidatus_Microthrix_parvicella
gi|1047840838−Mycobacterium_sp__djl−10
gi|929009236−Desulfatitalea_tepidiphila
gi|971005176−Chloracidobacterium_thermophilum
gi|739706719−Spirochaeta_sp__JC202
gi|517720316−Streptomyces_sp__CNT302
gi|516573579−Streptomyces_sp__CNS615
gi|654188635−Streptomyces_sp__CNR698
gi|558874575−Streptomycetaceae_bacterium_MP113−05
gi|1039607474−Mycobacterium_sp__852002−51759_SCH5129042
gi|558480466−uncultured_bacterium
gi|558480480−uncultured_bacterium
gi|558480464−uncultured_bacterium
gi|558480482−uncultured_bacterium
gi|558480462−uncultured_bacterium
gi|930023379−alpha_proteobacterium_AAP81b
gi|516263241−Sandarakinorhabdus_sp__AAP62
gi|550931846−Sandarakinorhabdus_limnophila
gi|1055076691−Sphingomonas_mali
gi|1054824110−Sphingomonas_pruni
gi|730265532−Sphingomonas_sp__Ant_H11
gi|1054928662−Dermacoccus_sp__CCH12−B7
gi|502844831−Caulobacter_segnis_ATCC_21756
gi|490757442−Caulobacter_crescentus_OR37
gi|947349108−Phenylobacterium_sp__Root77
gi|948073487−Phenylobacterium_sp__Root700
gi|780043913−Tepidicaulis_marinus
gi|670499751−Caulobacteraceae_bacterium_PMMR1
gi|1055018694−Caulobacteraceae_bacterium_OTSz_A_272
gi|1055810356−Erythrobacteraceae_bacterium_CCH12−C2
GSMAST4A2_ASY1_ANO1_665_1
gi|961671135−uncultured_bacterium
gi|652642662−Conexibacter_woesei
0.1
Bacteria
SAG

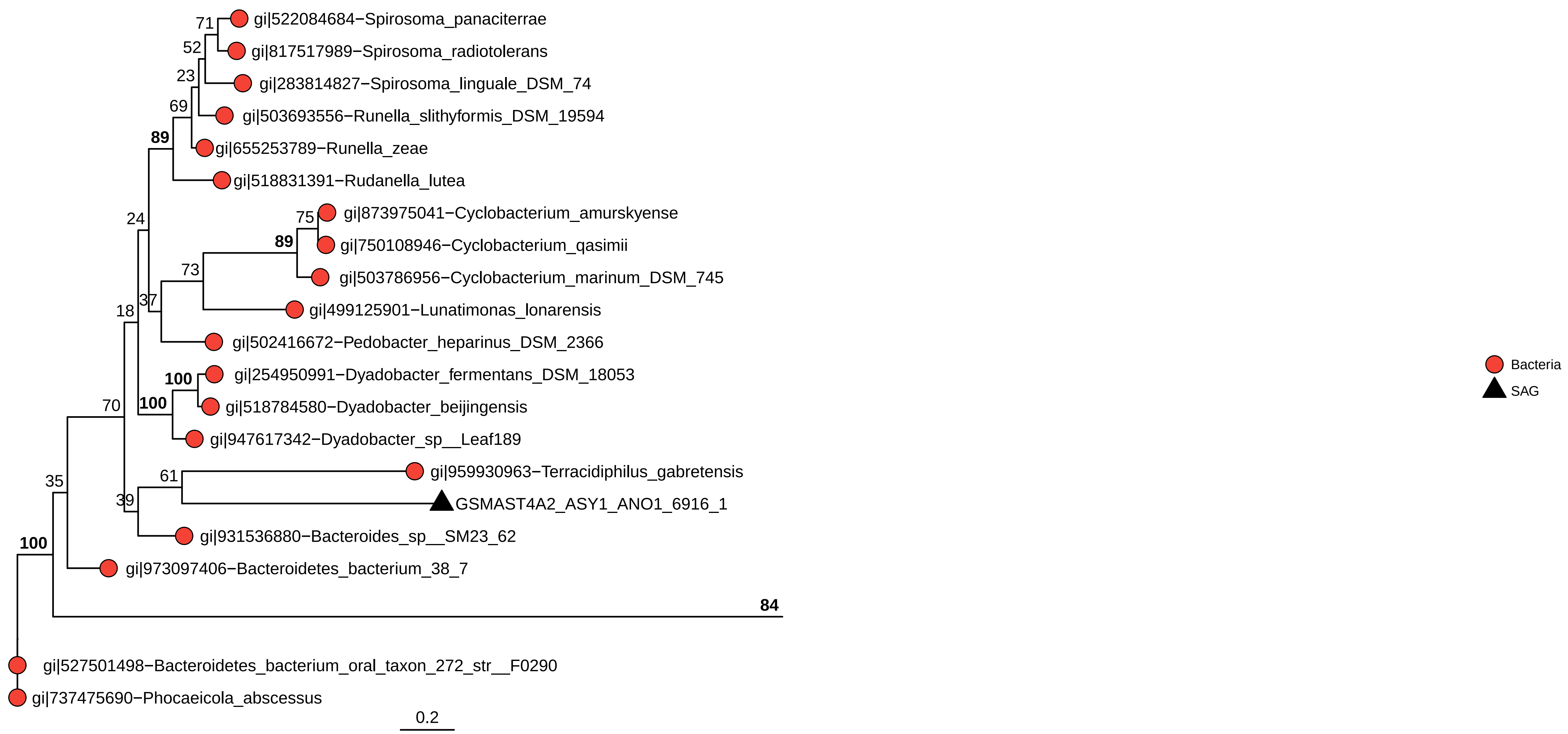
gi|522084684−Spirosoma_panaciterrae
gi|817517989−Spirosoma_radiotolerans
gi|283814827−Spirosoma_linguale_DSM_74
gi|503693556−Runella_slithyformis_DSM_19594
gi|655253789−Runella_zeae
gi|518831391−Rudanella_lutea
gi|873975041−Cyclobacterium_amurskyense
gi|750108946−Cyclobacterium_qasimii
gi|503786956−Cyclobacterium_marinum_DSM_745
gi|499125901−Lunatimonas_lonarensis
gi|502416672−Pedobacter_heparinus_DSM_2366
gi|254950991−Dyadobacter_fermentans_DSM_18053
gi|518784580−Dyadobacter_beijingensis
gi|947617342−Dyadobacter_sp__Leaf189
gi|959930963−Terracidiphilus_gabretensis
GSMAST4A2_ASY1_ANO1_6916_1
gi|931536880−Bacteroides_sp__SM23_62
gi|973097406−Bacteroidetes_bacterium_38_7
gi|527501498−Bacteroidetes_bacterium_oral_taxon_272_str__F0290
gi|737475690−Phocaeicola_abscessus
71
52
23
69
89
24
75
89
73
37
18
100
100
70
61
39
35
100
84
0.2
Bacteria
SAG

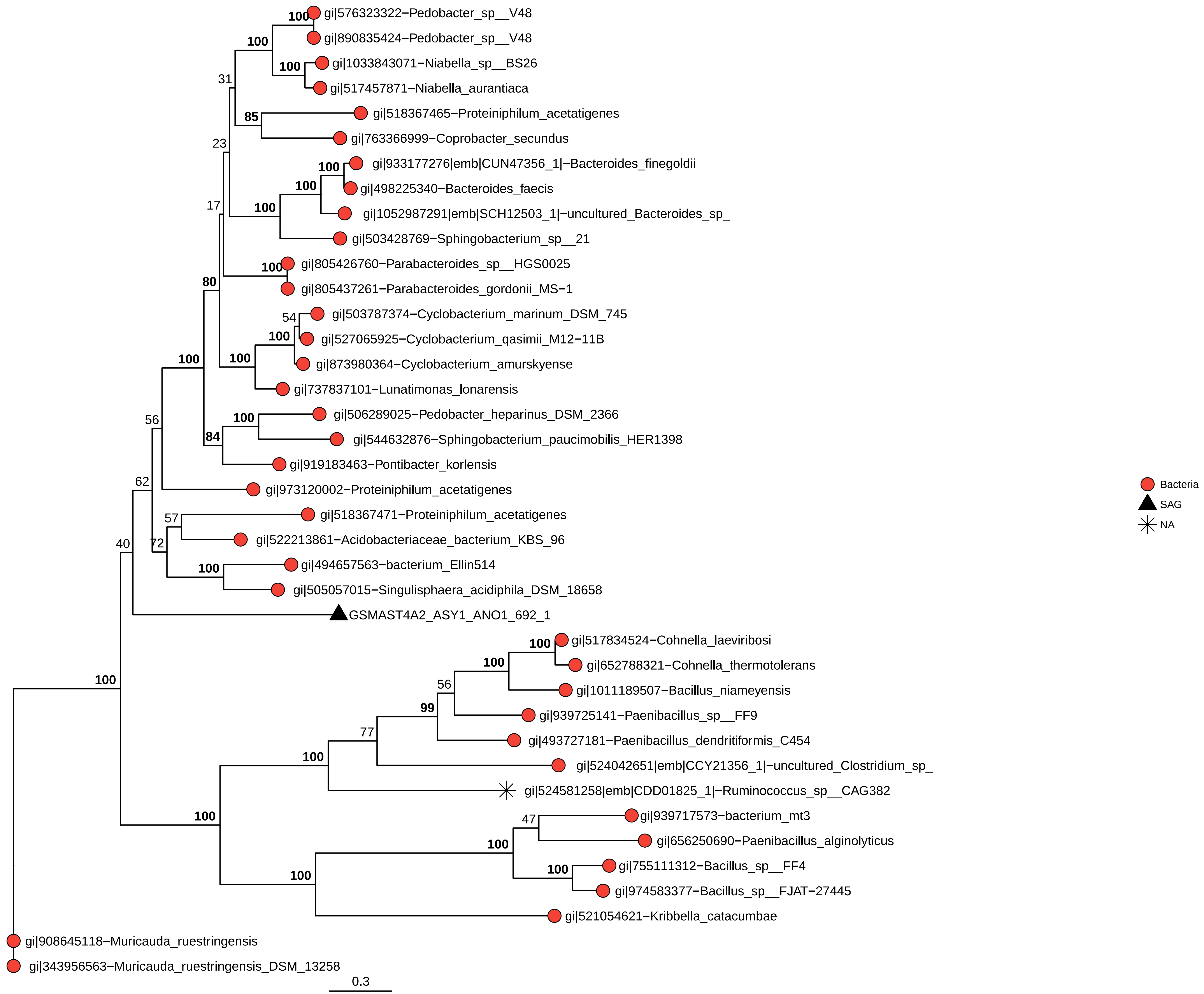
gi|576323322−Pedobacter_sp__V48
gi|890835424−Pedobacter_sp__V48
gi|1033843071−Niabella_sp__BS26
gi|517457871−Niabella_aurantiaca
gi|518367465−Proteiniphilum_acetatigenes
gi|763366999−Coprobacter_secundus
gi|933177276|emb|CUN47356_1|−Bacteroides_finegoldii
gi|498225340−Bacteroides_faecis
gi|1052987291|emb|SCH12503_1|−uncultured_Bacteroides_sp_
gi|503428769−Sphingobacterium_sp__21
gi|805426760−Parabacteroides_sp__HGS0025
gi|805437261−Parabacteroides_gordonii_MS−1
gi|503787374−Cyclobacterium_marinum_DSM_745
gi|527065925−Cyclobacterium_qasimii_M12−11B
gi|873980364−Cyclobacterium_amurskyense
gi|737837101−Lunatimonas_lonarensis
gi|506289025−Pedobacter_heparinus_DSM_2366
gi|544632876−Sphingobacterium_paucimobilis_HER1398
gi|919183463−Pontibacter_korlensis
gi|973120002−Proteiniphilum_acetatigenes
gi|518367471−Proteiniphilum_acetatigenes
gi|522213861−Acidobacteriaceae_bacterium_KBS_96
gi|494657563−bacterium_Ellin514
gi|505057015−Singulisphaera_acidiphila_DSM_18658
GSMAST4A2_ASY1_ANO1_692_1
gi|517834524−Cohnella_laeviribosi
gi|652788321−Cohnella_thermotolerans
gi|1011189507−Bacillus_niameyensis
gi|939725141−Paenibacillus_sp__FF9
gi|493727181−Paenibacillus_dendritiformis_C454
gi|524042651|emb|CCY21356_1|−uncultured_Clostridium_sp_
gi|524581258|emb|CDD01825_1|−Ruminococcus_sp__CAG382
gi|939717573−bacterium_mt3
gi|656250690−Paenibacillus_alginolyticus
gi|755111312−Bacillus_sp__FF4
gi|974583377−Bacillus_sp__FJAT−27445
gi|521054621−Kribbella_catacumbae
gi|908645118−Muricauda_ruestringensis
gi|343956563−Muricauda_ruestringensis_DSM_13258
0.3
Bacteria
SAG
NA

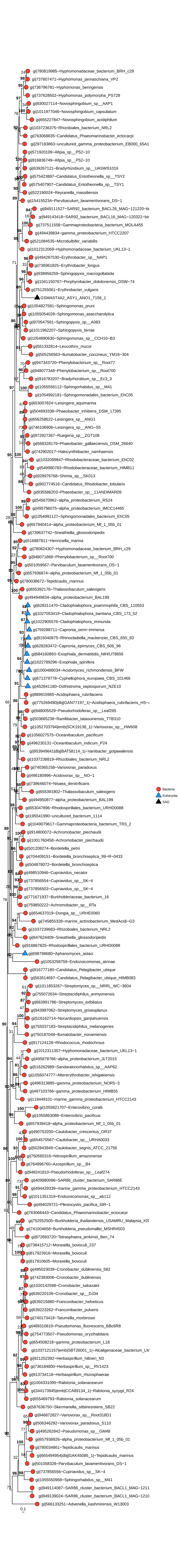
Bacteria
Eukaryota
SAG
gi|780819985~Hyphomonadaceae_bacterium_BRH_c29
gi|737607471~Hyphomonas_jannaschiana_VP2
gi|736796781~Hyphomonas_beringensis
gi|737628502~Hyphomonas_polymorpha_PS728
gi|930027114~Novosphingobium_sp__AAP1
gi|1011797046~Novosphingobium_capsulatum
gi|655227847~Novosphingobium_acidiphilum
gi|1037236375~Rhizobiales_bacterium_NRL2
gi|763068835~Candidatus_Phaeomarinobacter_ectocarpi
gi|297183863~uncultured_gamma_proteobacterium_EB000_65A11
gi|571920109~Afipia_sp__P52~10
gi|916836749~Afipia_sp__P52~10
gi|639267121~Bradyrhizobium_sp__UASWS1016
gi|575423897~Candidatus_Entotheonella_sp__TSY2
gi|575407907~Candidatus_Entotheonella_sp__TSY1
gi|522190024~Reyranella_massiliensis
gi|154155234~Parvibaculum_lavamentivorans_DS~1
gi|949111527~SAR92_bacterium_BACL26_MAG~121220~bin70
gi|949143418~SAR92_bacterium_BACL16_MAG~120322~bin99
gi|737511558~Gammaproteobacteria_bacterium_MOLA455
gi|494439834~gamma_proteobacterium_HTCC2207
gi|521084535~Microbulbifer_variabilis
gi|1012312069~Hyphomonadaceae_bacterium_UKL13~1
gi|494287530~Erythrobacter_sp__NAP1
gi|736961825~Erythrobacter_longus
gi|938956259~Sphingopyxis_macrogoltabida
gi|1041150767~Porphyrobacter_dokdonensis_DSW~74
gi|751255061~Erythrobacter_vulgaris
GSMAST4A2_ASY1_ANO1_7156_1
gi|1054827581~Sphingomonas_pruni
gi|1055054028~Sphingomonas_asaccharolytica
gi|970547561~Sphingopyxis_sp__A083
gi|1011962207~Sphingopyxis_terrae
gi|1054890630~Sphingomonas_sp__CCH10~B3
gi|551332814~Leucothrix_mucor
gi|505256563~Illumatobacter_coccineus_YM16~304
gi|947343720~Phenylobacterium_sp__Root77
gi|948077348~Phenylobacterium_sp__Root700
gi|916783207~Bradyrhizobium_sp__Ec3_3
gi|1055556112~Sphingorhabdus_sp__M41
gi|1054992181~Sphingomonadales_bacterium_EhC05
gi|653007824~Leisingera_aquimarina
gi|504693338~Phaeobacter_inhibens_DSM_17395
gi|656258622~Leisingera_sp__ANG1
gi|746106906~Leisingera_sp__ANG~S5
gi|972927367~Ruegeria_sp__ZGT108
gi|568328179~Phaeobacter_gallaeciensis_DSM_26640
gi|742902017~Halocynthiibacter_namhaensis
gi|1032099847~Rhodobacteraceae_bacterium_EhC02
gi|549980783~Rhodobacteraceae_bacterium_HIMB11
gi|928976768~Shimia_sp__SK013
gi|902774516~Candidatus_Rhodobacter_lobularis
gi|935586203~Phaeobacter_sp__11ANDIMAR09
gi|545670962~alpha_proteobacterium_RS24
gi|495796075~alpha_proteobacterium_IMCC14465
gi|1054991127~Sphingomonadales_bacterium_EhC05
gi|657940414~alpha_proteobacterium_Mf_1_05b_01
gi|739637742~Sneathiella_glossodoripedis
gi|516887811~Henriciella_marina
gi|780824307~Hyphomonadaceae_bacterium_BRH_c29
gi|948071868~Phenylobacterium_sp__Root700
gi|501059567~Parvibaculum_lavamentivorans_DS~1
gi|657936874~alpha_proteobacterium_Mf_1_05b_01
gi|780038672~Tepidicaulis_marinus
gi|655392178~Thalassobaculum_salexigens
gi|494949834~alpha_proteobacterium_BAL199
gi|628311470~Cladophialophora_psammophila_CBS_110553
gi|1027053419~Cladophialophora_bantiana_CBS_173_52
gi|1022905576~Cladophialophora_immunda
gi|759288711~Capronia_semi~immersa
gi|915040875~Rhinocladiella_mackenziei_CBS_650_93
gi|628283472~Capronia_epimyces_CBS_606_96
gi|684160893~Exophiala_dermatitidis_NIH/UT8656
gi|1022789296~Exophiala_spinifera
gi|1000488034~Acidomyces_richmondensis_BFW
gi|671379778~Cyphellophora_europaea_CBS_101466
gi|452841180~Dothistroma_septosporum_NZE10
gi|889915965~Acidiphilaea_rubrifaciens
gi|775269490|dbj|GAN77197_1|~Acidisphaera_rubrifaciens_HS~AP3
gi|948005529~Pseudorhodoferax_sp__Leaf265
gi|503665238~Ramlibacter_tataouinensis_TTB310
gi|1052703794|emb|SCK19138_1|~Variovorax_sp__HW608
gi|1056027575~Oceanibaculum_pacificum
gi|496230131~Oceanibaculum_indicum_P24
gi|953949641|dbj|BAT58114_1|~Variibacter_gotjawalensis
gi|1037238819~Rhizobiales_bacterium_NRL2
gi|740365158~Variovorax_paradoxus
gi|496189996~Acidovorax_sp__NO~1
gi|738646074~Nisaea_denitrificans
gi|655391802~Thalassobaculum_salexigens
gi|494950877~alpha_proteobacterium_BAL199
gi|653047896~Rhodospirillales_bacterium_URHD0088
gi|195541990~uncultured_bacterium_1114
gi|1049079617~Gammaproteobacteria_bacterium_TR3_2
gi|914800072~Achromobacter_piechaudii
gi|1001760458~Achromobacter_piechaudii
gi|501208274~Bordetella_petrii
gi|704409151~Bordetella_bronchiseptica_99~R~0433
gi|504878072~Bordetella_bronchiseptica
gi|498510946~Cupriavidus_necator
gi|737856554~Cupriavidus_sp__SK~4
gi|737856503~Cupriavidus_sp__SK~4
gi|771671937~Burkholderiaceae_bacterium_16
gi|759850222~Achromobacter_sp__RTa
gi|654637019~Dongia_sp__URHE0060
gi|745855328~marine_actinobacterium_MedAcidi~G3
gi|1037239683~Rhizobiales_bacterium_NRL2
gi|647624409~Sneathiella_glossodoripedis
gi|916867825~Rhodospirillales_bacterium_URHD0088
gi|698798680~Aphanomyces_astaci
gi|1053258759~Endozoicomonas_atrinae
gi|739917180~Candidatus_Pelagibacter_ubique
gi|563514697~Candidatus_Pelagibacter_ubique_HIMB083
gi|1011853267~Streptomyces_sp__NRRL_WC~3604
gi|755072634~Streptacidiphilus_anmyonensis
gi|663991786~Streptomyces_exfoliatus
gi|943997062~Streptomyces_griseoplanus
gi|516162714~Nocardiopsis_ganjiahuensis
gi|755037183~Streptacidiphilus_melanogenes
gi|750187048~Illumatobacter_nonamiensis
gi|971124128~Rhodococcus_rhodochrous
gi|1012311357~Hyphomonadaceae_bacterium_UKL13~1
gi|495878766~alpha_proteobacterium_JLT2015
gi|516262989~Sandarakinorhabdus_sp__AAP62
gi|1056074377~Altererythrobacter_ishigakiensis
gi|496313885~gamma_proteobacterium_NOR5~3
gi|497103766~gamma_proteobacterium_HIMB55
gi|119449101~marine_gamma_proteobacterium_HTCC2143
gi|1055821707~Enterovibrio_coralii
gi|1055863088~Enterovibrio_pacificus
gi|657939418~alpha_proteobacterium_Mf_1_05b_01
gi|490753200~Caulobacter_crescentus_OR37
gi|654570567~Caulobacter_sp__URHA0033
gi|502843949~Caulobacter_segnis_ATCC_21756
gi|750565316~Nitrospirillum_amazonense
gi|764996760~Azospirillum_sp__B4
gi|948241810~Pseudorhodoferax_sp__Leaf274
gi|409980096~SAR86_cluster_bacterium_SAR86E
gi|494429339~marine_gamma_proteobacterium_HTCC2143
gi|1011351309~Endozoicomonas_sp__ab112
gi|494029721~Plesiocystis_pacifica_SIR~1
gi|763066443~Candidatus_Phaeomarinobacter_ectocarpi
gi|752552505~Burkholderia_thailandensis_USAMRU_Malaysia_#20
gi|741004658~Burkholderia_pseudomallei_MSHR4503
gi|872693720~Tetrasphaera_jenkinsii_Ben_74
gi|738415712~Moraxella_bovoculi_237
gi|817923916~Moraxella_bovoculi
gi|817910605~Moraxella_bovoculi
gi|495023039~Cronobacter_dublinensis_582
gi|742393006~Cronobacter_dublinensis
gi|1020142598~Cronobacter_sakazakii
gi|639220109~Cronobacter_sp__DJ34
gi|639215880~Franconibacter_helveticus
gi|639223262~Franconibacter_pulveris
gi|740173418~Tatumella_morbirosei
gi|489310819~Pseudomonas_fluorescens_BBc6R8
gi|754773507~Pseudomonas_oryzihabitans
gi|654508218~gamma_proteobacterium_L18
gi|1037121157|emb|SBT26001_1|~Alcaligenaceae_bacterium_LMG_22012
gi|921252392~Herbaspirillum_hiltneri_N3
gi|736184800~Herbaspirillum_sp__RV1423
gi|913734118~Herbaspirillum_rhizosphaerae
gi|1004331095~Ralstonia_solanacearum
gi|344173945|emb|CCA89134_1|~Ralstonia_syzygii_R24
gi|655469793~Ralstonia_solanacearum
gi|587636750~Skermanella_stibiiresistens_SB22
gi|946872827~Variovorax_sp__Root318D1
gi|506346292~Variovorax_paradoxus_S110
gi|495262842~Pseudomonas_sp__GM48
gi|657938926~alpha_proteobacterium_Mf_1_05b_01
gi|780034861~Tepidicaulis_marinus
gi|665494954|dbj|GAK45085_1|~Tepidicaulis_marinus
gi|501058328~Parvibaculum_lavamentivorans_DS~1
gi|737856556~Cupriavidus_sp__SK~4
gi|1055550968~Sphingorhabdus_sp__M41
gi|949114087~SAR86_cluster_bacterium_BACL1_MAG~121105~bin34
gi|949139024~SAR86_cluster_bacterium_BACL1_MAG~121001~bin56
gi|566133251~Advenella_kashmirensis_W13003
0.1

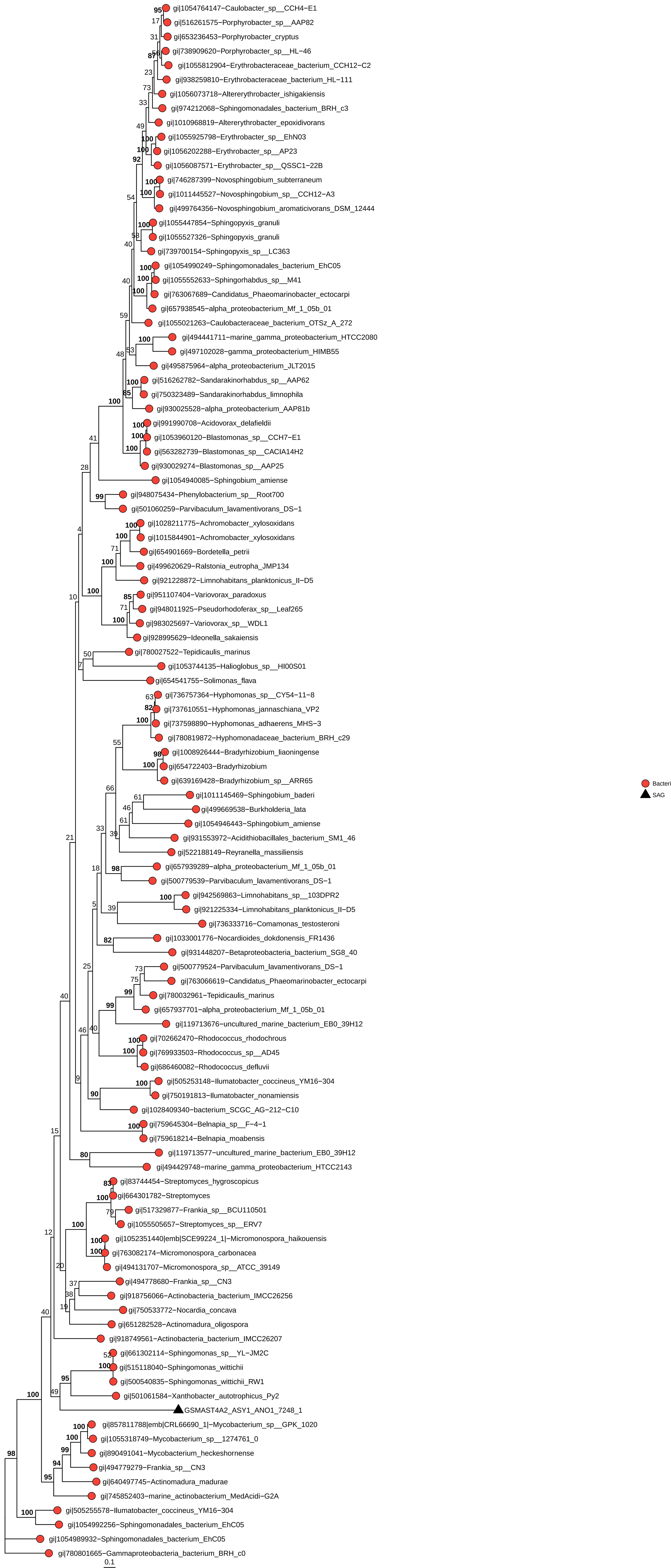
gi|1054764147−Caulobacter_sp__CCH4−E1
gi|516261575−Porphyrobacter_sp__AAP82
gi|653236453−Porphyrobacter_cryptus
gi|738909620−Porphyrobacter_sp__HL−46
gi|1055812904−Erythrobacteraceae_bacterium_CCH12−C2
gi|938259810−Erythrobacteraceae_bacterium_HL−111
gi|1056073718−Altererythrobacter_ishigakiensis
gi|974212068−Sphingomonadales_bacterium_BRH_c3
gi|1010968819−Altererythrobacter_epoxidivorans
gi|1055925798−Erythrobacter_sp__EhN03
gi|1056202288−Erythrobacter_sp__AP23
gi|1056087571−Erythrobacter_sp__QSSC1−22B
gi|746287399−Novosphingobium_subterraneum
gi|1011445527−Novosphingobium_sp__CCH12−A3
gi|499764356−Novosphingobium_aromaticivorans_DSM_12444
gi|1055447854−Sphingopyxis_granuli
gi|1055527326−Sphingopyxis_granuli
gi|739700154−Sphingopyxis_sp__LC363
gi|1054990249−Sphingomonadales_bacterium_EhC05
gi|1055552633−Sphingorhabdus_sp__M41
gi|763067689−Candidatus_Phaeomarinobacter_ectocarpi
gi|657938545−alpha_proteobacterium_Mf_1_05b_01
gi|1055021263−Caulobacteraceae_bacterium_OTSz_A_272
gi|494441711−marine_gamma_proteobacterium_HTCC2080
gi|497102028−gamma_proteobacterium_HIMB55
gi|495875964−alpha_proteobacterium_JLT2015
gi|516262782−Sandarakinorhabdus_sp__AAP62
gi|750323489−Sandarakinorhabdus_limnophila
gi|930025528−alpha_proteobacterium_AAP81b
gi|991990708−Acidovorax_delafieldii
gi|1053960120−Blastomonas_sp__CCH7−E1
gi|563282739−Blastomonas_sp__CACIA14H2
gi|930029274−Blastomonas_sp__AAP25
gi|1054940085−Sphingobium_amiense
gi|948075434−Phenylobacterium_sp__Root700
gi|501060259−Parvibaculum_lavamentivorans_DS−1
gi|1028211775−Achromobacter_xylosoxidans
gi|1015844901−Achromobacter_xylosoxidans
gi|654901669−Bordetella_petrii
gi|499620629−Ralstonia_eutropha_JMP134
gi|921228872−Limnohabitans_planktonicus_II−D5
gi|951107404−Variovorax_paradoxus
gi|948011925−Pseudorhodoferax_sp__Leaf265
gi|983025697−Variovorax_sp__WDL1
gi|928995629−Ideonella_sakaiensis
gi|780027522−Tepidicaulis_marinus
gi|1053744135−Halioglobus_sp__HI00S01
gi|654541755−Solimonas_flava
gi|736757364−Hyphomonas_sp__CY54−11−8
gi|737610551−Hyphomonas_jannaschiana_VP2
gi|737598890−Hyphomonas_adhaerens_MHS−3
gi|780819872−Hyphomonadaceae_bacterium_BRH_c29
gi|1008926444−Bradyrhizobium_liaoningense
gi|654722403−Bradyrhizobium
gi|639169428−Bradyrhizobium_sp__ARR65
gi|1011145469−Sphingobium_baderi
gi|499669538−Burkholderia_lata
gi|1054946443−Sphingobium_amiense
gi|931553972−Acidithiobacillales_bacterium_SM1_46
gi|522188149−Reyranella_massiliensis
gi|657939289−alpha_proteobacterium_Mf_1_05b_01
gi|500779539−Parvibaculum_lavamentivorans_DS−1
gi|942569863−Limnohabitans_sp__103DPR2
gi|921225334−Limnohabitans_planktonicus_II−D5
gi|736333716−Comamonas_testosteroni
gi|1033001776−Nocardioides_dokdonensis_FR1436
gi|931448207−Betaproteobacteria_bacterium_SG8_40
gi|500779524−Parvibaculum_lavamentivorans_DS−1
gi|763066619−Candidatus_Phaeomarinobacter_ectocarpi
gi|780032961−Tepidicaulis_marinus
gi|657937701−alpha_proteobacterium_Mf_1_05b_01
gi|119713676−uncultured_marine_bacterium_EB0_39H12
gi|702662470−Rhodococcus_rhodochrous
gi|769933503−Rhodococcus_sp__AD45
gi|686460082−Rhodococcus_defluvii
gi|505253148−Ilumatobacter_coccineus_YM16−304
gi|750191813−Ilumatobacter_nonamiensis
gi|1028409340−bacterium_SCGC_AG−212−C10
gi|759645304−Belnapia_sp__F−4−1
gi|759618214−Belnapia_moabensis
gi|119713577−uncultured_marine_bacterium_EB0_39H12
gi|494429748−marine_gamma_proteobacterium_HTCC2143
gi|83744454−Streptomyces_hygroscopicus
gi|664301782−Streptomyces
gi|517329877−Frankia_sp__BCU110501
gi|1055505657−Streptomyces_sp__ERV7
gi|1052351440|emb|SCE99224_1|−Micromonospora_haikouensis
gi|763082174−Micromonospora_carbonacea
gi|494131707−Micromonospora_sp__ATCC_39149
gi|494778680−Frankia_sp__CN3
gi|918756066−Actinobacteria_bacterium_IMCC26256
gi|750533772−Nocardia_concava
gi|651282528−Actinomadura_oligospora
gi|918749561−Actinobacteria_bacterium_IMCC26207
gi|661302114−Sphingomonas_sp__YL−JM2C
gi|515118040−Sphingomonas_wittichii
gi|500540835−Sphingomonas_wittichii_RW1
gi|501061584−Xanthobacter_autotrophicus_Py2
GSMAST4A2_ASY1_ANO1_7248_1
gi|857811788|emb|CRL66690_1|−Mycobacterium_sp__GPK_1020
gi|1055318749−Mycobacterium_sp__1274761_0
gi|890491041−Mycobacterium_heckeshornense
gi|494779279−Frankia_sp__CN3
gi|640497745−Actinomadura_madurae
gi|745852403−marine_actinobacterium_MedAcidi−G2A
gi|505255578−Ilumatobacter_coccineus_YM16−304
gi|1054992256−Sphingomonadales_bacterium_EhC05
gi|1054989932−Sphingomonadales_bacterium_EhC05
gi|780801665−Gammaproteobacteria_bacterium_BRH_c0
0.1
Bacteria
SAG

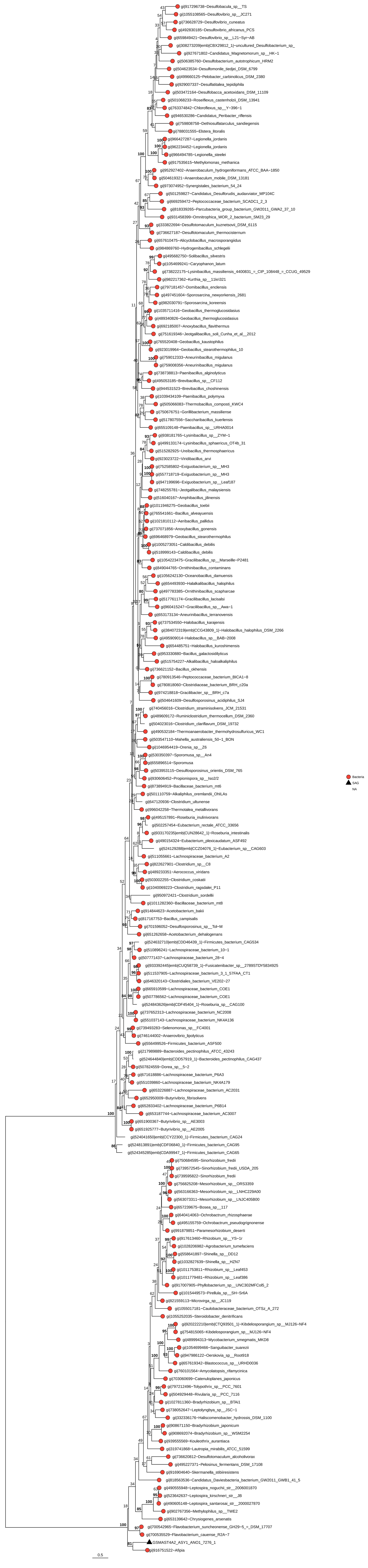
Bacteria
SAG
NA
0.5

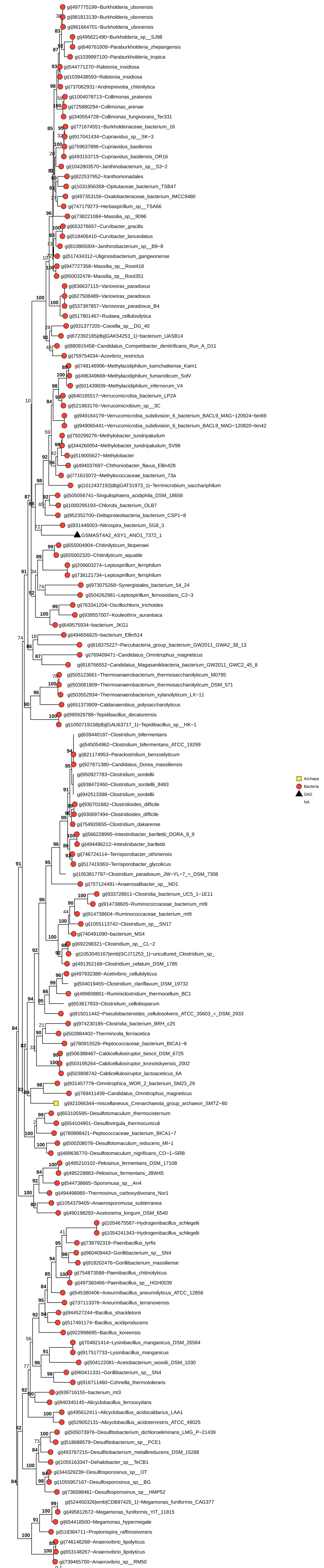
gi|497775199~Burkholderia_ubonensis
gi|981813139~Burkholderia_ubonensis
gi|981664701~Burkholderia_ubonensis
gi|495621490~Paraburkholderia_sp__SJ98
gi|646761609~Paraburkholderia_zhejiangensis
gi|1039997100~Paraburkholderia_tropica
gi|544771270~Ralstonia_insidiosa
gi|1039438593~Ralstonia_insidiosa
gi|737062931~Andreprevotia_chitinilytica
gi|1004078713~Collimonas_pratensis
gi|725880294~Collimonas_arenae
gi|340554728~Collimonas_fungivorans_Ter331
gi|771674551~Burkholderiaceae_bacterium_16
gi|917041434~Cupriavidus_sp__SK-3
gi|759637896~Cupriavidus_basilensis
gi|493153715~Cupriavidus_basilensis_OR16
gi|1042803570~Janthinobacterium_sp__S3-2
gi|822537952~Xanthomonadales
gi|1031956358~Opitutaceae_bacterium_TSB47
gi|497353156~Oxalobacteraceae_bacterium_IMCC9480
gi|747179273~Herbaspirillum_sp__TSA66
gi|738221084~Massilia_sp__9096
gi|653276657~Curvibacter_gracilis
gi|518406410~Curvibacter_lanceolatus
gi|810865004~Janthinobacterium_sp__B9-8
gi|517434312~Uliginosibacterium_gangwonense
gi|947727358~Massilia_sp__Root418
gi|950032476~Massilia_sp__Root351
gi|836637115~Variovorax_paradoxus
gi|827508489~Variovorax_paradoxus
gi|537387857~Variovorax_paradoxus_B4
gi|517801467~Rudaea_cellulosilytica
gi|931377205~Coxiella_sp__DG_40
gi|672392185|dbj|GAK54253_1|~bacterium_UASB14
gi|880915458~Candidatus_Competibacter_denitrificans_Run_A_D11
gi|759754034~Azovibrio_restrictus
gi|748146996~Methylacidiphilum_kamchatkense_Kam1
gi|496349668~Methylacidiphilum_fumariolicum_SolV
gi|501439839~Methylacidiphilum_infernorum_V4
gi|640165517~Verrucomicrobia_bacterium_LP2A
gi|521983176~Verrucomicrobium_sp__3C
gi|949164179~Verrucomicrobia_subdivision_6_bacterium_BACL9_MAG-120924-bin69
gi|949065441~Verrucomicrobia_subdivision_6_bacterium_BACL9_MAG-120820-bin42
gi|750299276~Methylobacter_tundripaludum
gi|344260054~Methylobacter_tundripaludum_SV96
gi|519005627~Methylobacter
gi|494037697~Chthoniobacter_flavus_Ellin428
gi|771615072~Methylococcaceae_bacterium_73a
gi|1012437192|dbj|GAT31973_1|~Terrimicrobium_sacchariphilum
gi|505056741~Singulisphaera_acidiphila_DSM_18658
gi|1000295193~Chlorobi_bacterium_OLB7
gi|952352700~Deltaproteobacteria_bacterium_CSP1-8
gi|931446003~Nitrospira_bacterium_SG8_3
GSMAST4A2_ASY1_ANO1_7372_1
gi|655004904~Chitinilyticum_litopenaei
gi|655002320~Chitinilyticum_aquatile
gi|206603274~Leptospirillum_ferriphilum
gi|738121734~Leptospirillum_ferriphilum
gi|973075268~Synergistales_bacterium_54_24
gi|504262981~Leptospirillum_ferrooxidans_C2-3
gi|763341204~Oscillochloris_trichoides
gi|939557007~Kouleothrix_aurantiaca
gi|649575934~bacterium_JKG1
gi|494656625~bacterium_Ellin514
gi|818375227~Parcubacteria_group_bacterium_GW2011_GWA2_38_13
gi|769409471~Candidatus_Omnitrophus_magneticus
gi|818766552~Candidatus_Magasanikbacteria_bacterium_GW2011_GWC2_45_8
gi|505123661~Thermoanaerobacterium_thermosaccharolyticum_M0795
gi|503061809~Thermoanaerobacterium_thermosaccharolyticum_DSM_571
gi|503552934~Thermoanaerobacterium_xylanolyticum_LX-11
gi|651373909~Caldanaerobius_polysaccharolyticus
gi|995929788~Tepidibacillus_decaturensis
gi|1050719158|dbj|GAJ63717_1|~Tepidibacillus_sp__HK-1
gi|639440187~Clostridium_bifermentans
gi|545054962~Clostridium_bifermentans_ATCC_19299
gi|821174953~Paraclostridium_benzoelyticum
gi|927871380~Candidatus_Dorea_massiliensis
gi|950927783~Clostridium_sordellii
gi|938472460~Clostridium_sordellii_8483
gi|942513398~Clostridium_sordellii
gi|930701682~Clostridioides_difficile
gi|930697494~Clostridioides_difficile
gi|754920655~Clostridium_dakarense
gi|566228995~Intestinibacter_bartlettii_DORA_8_9
gi|494496212~Intestinibacter_bartlettii
gi|746724114~Terrisporobacter_othiniensis
gi|517419363~Terrisporobacter_glycolicus
gi|1053817797~Clostridium_paradoxum_JW-YL-7_=_DSM_7308
gi|757124491~Anaerosalibacter_sp__ND1
gi|933728911~Clostridia_bacterium_UC5_1-1E11
gi|914738605~Ruminococcaceae_bacterium_mt9
gi|914738604~Ruminococcaceae_bacterium_mt9
gi|1055113742~Clostridium_sp__SN17
gi|740491090~bacterium_MS4
gi|692298321~Clostridium_sp__CL-2
gi|1053045167|emb|SCJ71253_1|~uncultured_Clostridium_sp_
gi|491352168~Clostridium_celatum_DSM_1785
gi|497932388~Acetivibrio_cellulolyticus
gi|504019455~Clostridium_clariflavum_DSM_19732
gi|489608801~Ruminiclostridium_thermocellum_BC1
gi|653617933~Clostridium_cellobioparum
gi|915011442~Pseudobacteroides_cellulosolvens_ATCC_35603_=_DSM_2933
gi|974230185~Clostridia_bacterium_BRH_c25
gi|502884402~Thermincola_ferriacetica
gi|780915526~Peptococcaceae_bacterium_BICA1-8
gi|506388467~Caldicellulosiruptor_bescii_DSM_6725
gi|503195264~Caldicellulosiruptor_kronotskyensis_2002
gi|503808742~Caldicellulosiruptor_lactoaceticus_6A
gi|931457779~Omnitrophica_WOR_2_bacterium_SM23_29
gi|769411439~Candidatus_Omnitrophus_magneticus
gi|921066344~miscellaneous_Crenarchaeota_group_archaeon_SMTZ-80
gi|653105595~Desulfotomaculum_thermocisternum
gi|654104901~Desulfovirgula_thermocuniculi
gi|780898421~Peptococcaceae_bacterium_BICA1-7
gi|500208078~Desulfotomaculum_reducens_MI-1
gi|489636770~Desulfotomaculum_nigrificans_CO-1-SRB
gi|495210102~Pelosinus_fermentans_DSM_17108
gi|495228883~Pelosinus_fermentans_JBW45
gi|544738665~Sporomusa_sp__An4
gi|494498985~Thermosinus_carboxydivorans_Nor1
gi|1054379405~Anaerosporomusa_subterranea
gi|490198293~Acetonema_longum_DSM_6540
gi|1054675587~Hydrogenibacillus_schlegelii
gi|1054241343~Hydrogenibacillus_schlegelii
gi|738792319~Paenibacillus_tyrfis
gi|960409443~Gorillibacterium_sp__SN4
gi|918202476~Gorillibacterium_massiliense
gi|754873588~Paenibacillus_chitinolyticus
gi|497360466~Paenibacillus_sp__HGH0039
gi|545380406~Aneurinibacillus_aneurinilyticus_ATCC_12856
gi|737113376~Aneurinibacillus_terranovensis
gi|944527244~Bacillus_shackletonii
gi|517491174~Bacillus_acidiproducens
gi|922998695~Bacillus_koreensis
gi|704821414~Lysinibacillus_manganicus_DSM_26584
gi|917517733~Lysinibacillus_manganicus
gi|504122081~Acetobacterium_woodii_DSM_1030
gi|960411331~Gorillibacterium_sp__SN4
gi|916711460~Cohnella_thermotolerans
gi|939716155~bacterium_mt3
gi|940345145~Alicyclobacillus_ferrooxydans
gi|495612411~Alicyclobacillus_acidocaldarius_LAA1
gi|529052131~Alicyclobacillus_acidoterrestris_ATCC_49025
gi|505073976~Desulfitobacterium_dichloroeliminans_LMG_P-21439
gi|518688579~Desulfitobacterium_sp__PCE1
gi|493767215~Desulfitobacterium_metallireducens_DSM_15288
gi|1055163347~Dehalobacter_sp__TeCB1
gi|344329239~Desulfosporosinus_sp__OT
gi|1055957167~Desulfosporosinus_sp__BG
gi|736588461~Desulfosporosinus_sp__HMP52
gi|524450326|emb|CDB97425_1|~Megamonas_funiformis_CAG377
gi|495812672~Megamonas_funiformis_YIT_11815
gi|654418500~Megamonas_hypermegale
gi|518384711~Propionispira_raffinosivorans
gi|746146268~Anaerovibrio_lipolyticus
gi|653148267~Anaerovibrio_lipolyticus
gi|739450765~Anaerovibrio_sp__RM50
0.1
Archaea
Bacteria
SAG
NA

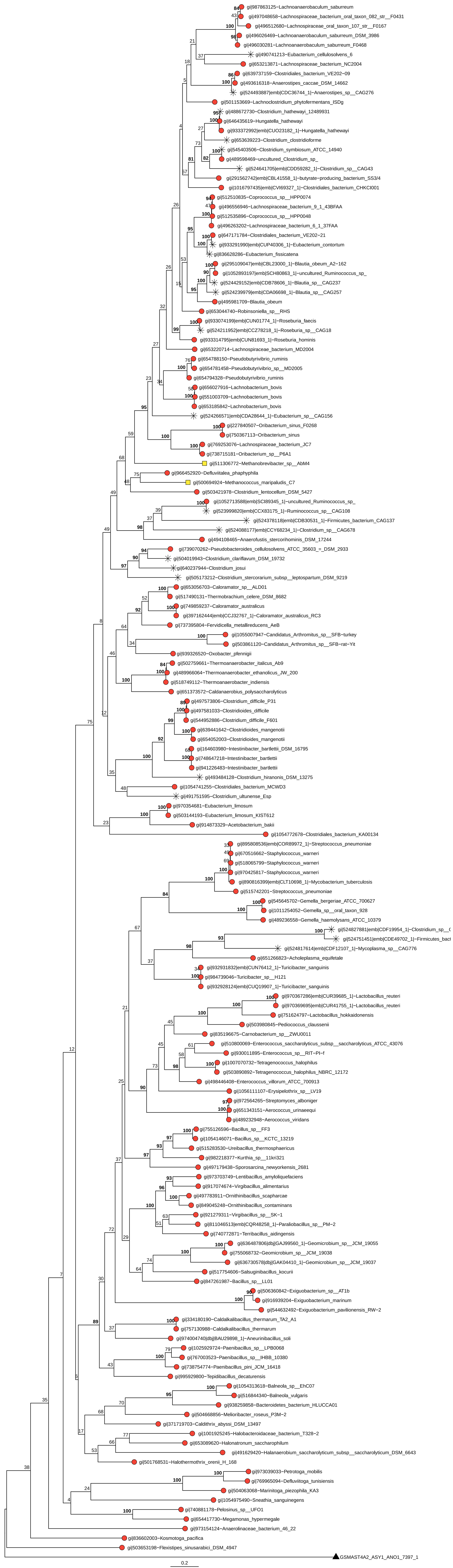
gi|987863125~Lachnoanaerobaculum_saburreum
gi|497048658~Lachnospiraceae_bacterium_oral_taxon_082_str__F0431
gi|496512680~Lachnospiraceae_bacterium_oral_taxon_107_str__F0167
gi|496026469~Lachnoanaerobaculum_saburreum_DSM_3986
gi|496030281~Lachnoanaerobaculum_saburreum_F0468
gi|490741213~Eubacterium_cellulosolvens_6
gi|653213871~Lachnospiraceae_bacterium_NC2004
gi|639737159~Clostridiales_bacterium_VE202-09
gi|493616318~Anaerostipes_caccae_DSM_14662
gi|524493887|emb|CDC36744_1|~Anaerostipes_sp__CAG276
gi|501153669~Lachnoclostridium_phytofermentans_ISDg
gi|488672730~Clostridium_hathewayi_12489931
gi|646435619~Hungatella_hathewayi
gi|933372992|emb|CUO23182_1|~Hungatella_hathewayi
gi|653639223~Clostridium_clostridioforme
gi|545403506~Clostridium_symbiosum_ATCC_14940
gi|489598469~uncultured_Clostridium_sp_
gi|524641705|emb|CDD59282_1|~Clostridium_sp__CAG43
gi|291562742|emb|CBL41558_1|~butyrate-producing_bacterium_SS3/4
gi|1016797435|emb|CVI69327_1|~Clostridiales_bacterium_CHKCI001
gi|512510835~Coprococcus_sp__HPP0074
gi|496556946~Lachnospiraceae_bacterium_9_1_43BFAA
gi|512535896~Coprococcus_sp__HPP0048
gi|496263202~Lachnospiraceae_bacterium_6_1_37FAA
gi|647171784~Clostridiales_bacterium_VE202-21
gi|933291990|emb|CUP40306_1|~Eubacterium_contortum
gi|836628286~Eubacterium_fissicatena
gi|295109047|emb|CBL23000_1|~Blautia_obeum_A2-162
gi|1052893197|emb|SCH80863_1|~uncultured_Ruminococcus_sp_
gi|524429152|emb|CDB78606_1|~Blautia_sp__CAG237
gi|524239979|emb|CDA06698_1|~Blautia_sp__CAG257
gi|495981709~Blautia_obeum
gi|653044740~Robinsoniella_sp__RHS
gi|933074199|emb|CUN01774_1|~Roseburia_faecis
gi|524211952|emb|CCZ78218_1|~Roseburia_sp__CAG18
gi|933314795|emb|CUN81693_1|~Roseburia_hominis
gi|653220714~Lachnospiraceae_bacterium_MD2004
gi|654788150~Pseudobutyrivibrio_ruminis
gi|654781458~Pseudobutyrivibrio_sp__MD2005
gi|654794328~Pseudobutyrivibrio_ruminis
gi|656027916~Lachnobacterium_bovis
gi|551003709~Lachnobacterium_bovis
gi|653185842~Lachnobacterium_bovis
gi|524266571|emb|CDA28644_1|~Eubacterium_sp__CAG156
gi|227840507~Oribacterium_sinus_F0268
gi|750367113~Oribacterium_sinus
gi|769253076~Lachnospiraceae_bacterium_JC7
gi|738715181~Oribacterium_sp__P6A1
gi|511306772~Methanobrevibacter_sp__AbM4
gi|966452920~Defluviitalea_phaphyphila
gi|500694924~Methanococcus_maripaludis_C7
gi|503421978~Clostridium_lentocellum_DSM_5427
gi|1052713588|emb|SCI89345_1|~uncultured_Ruminococcus_sp_
gi|523999820|emb|CCX83175_1|~Ruminococcus_sp__CAG108
gi|524378118|emb|CDB30531_1|~Firmicutes_bacterium_CAG137
gi|524088177|emb|CCY68234_1|~Clostridium_sp__CAG678
gi|494108465~Anaerofustis_stercorihominis_DSM_17244
gi|739070262~Pseudobacteroides_cellulosolvens_ATCC_35603_=_DSM_2933
gi|504019943~Clostridium_clariflavum_DSM_19732
gi|640237944~Clostridium_josui
gi|505173212~Clostridium_stercorarium_subsp__leptospartum_DSM_9219
gi|653056703~Caloramator_sp__ALD01
gi|517490131~Thermobrachium_celere_DSM_8682
gi|749859237~Caloramator_australicus
gi|397162444|emb|CCJ32767_1|~Caloramator_australicus_RC3
gi|737395804~Fervidicella_metallireducens_AeB
gi|1055007947~Candidatus_Arthromitus_sp__SFB-turkey
gi|503861120~Candidatus_Arthromitus_sp__SFB-rat-Yit
gi|939326520~Oxobacter_pfennigii
gi|502759661~Thermoanaerobacter_italicus_Ab9
gi|489966064~Thermoanaerobacter_ethanolicus_JW_200
gi|518749112~Thermoanaerobacter_indiensis
gi|651373572~Caldanaerobius_polysaccharolyticus
gi|497573806~Clostridium_difficile_P31
gi|497581033~Clostridioides_difficile
gi|544952886~Clostridium_difficile_F601
gi|639441642~Clostridioides_mangenotii
gi|654052003~Clostridioides_mangenotii
gi|164603980~Intestinibacter_bartlettii_DSM_16795
gi|748647218~Intestinibacter_bartlettii
gi|941226483~Intestinibacter_bartlettii
gi|493484128~Clostridium_hiranonis_DSM_13275
gi|1054741255~Clostridiales_bacterium_MCWD3
gi|491751595~Clostridium_ultunense_Esp
gi|970354681~Eubacterium_limosum
gi|503144193~Eubacterium_limosum_KIST612
gi|914873329~Acetobacterium_bakii
gi|1054772678~Clostridiales_bacterium_KA00134
gi|895808536|emb|COR89972_1|~Streptococcus_pneumoniae
gi|670516662~Staphylococcus_warneri
gi|518065799~Staphylococcus_warneri
gi|970425817~Staphylococcus_warneri
gi|890816399|emb|CLT10698_1|~Mycobacterium_tuberculosis
gi|515742201~Streptococcus_pneumoniae
gi|545645702~Gemella_bergeriae_ATCC_700627
gi|1011254052~Gemella_sp__oral_taxon_928
gi|489236558~Gemella_haemolysans_ATCC_10379
gi|524827881|emb|CDF19954_1|~Clostridium_sp__C
gi|524751451|emb|CDE49702_1|~Firmicutes_bact
gi|524817614|emb|CDF12107_1|~Mycoplasma_sp__CAG776
gi|651266823~Acholeplasma_equifetale
gi|932931832|emb|CUN76412_1|~Turicibacter_sanguinis
gi|984739046~Turicibacter_sp__H121
gi|932928124|emb|CUQ19907_1|~Turicibacter_sanguinis
gi|970367286|emb|CUR39685_1|~Lactobacillus_reuteri
gi|970369695|emb|CUR41755_1|~Lactobacillus_reuteri
gi|751624797~Lactobacillus_hokkaidonensis
gi|503980845~Pediococcus_claussenii
gi|835196675~Carnobacterium_sp__ZWU0011
gi|510800069~Enterococcus_saccharolyticus_subsp__saccharolyticus_ATCC_43076
gi|930011895~Enterococcus_sp__RIT-PI-f
gi|1007070732~Tetragenococcus_halophilus
gi|503890892~Tetragenococcus_halophilus_NBRC_12172
gi|498446408~Enterococcus_villorum_ATCC_700913
gi|1056111107~Erysipelothrix_sp__LV19
gi|972564265~Streptomyces_alboniger
gi|651343151~Aerococcus_urinaeequi
gi|489232948~Aerococcus_viridans
gi|755126596~Bacillus_sp__FF3
gi|1054146071~Bacillus_sp__KCTC_13219
gi|515283530~Ureibacillus_thermosphaericus
gi|982218377~Kurthia_sp__11kri321
gi|497179438~Sporosarcina_newyorkensis_2681
gi|973703749~Lentibacillus_amyloliquefaciens
gi|917074674~Virgibacillus_alimentarius
gi|497783911~Ornithinibacillus_scapharcae
gi|849045248~Ornithinibacillus_contaminans
gi|921279311~Virgibacillus_sp__SK-1
gi|811046513|emb|CQR48258_1|~Paraliobacillus_sp__PM-2
gi|740772871~Terribacillus_aidingensis
gi|636487806|dbj|GAJ99560_1|~Geomicrobium_sp__JCM_19055
gi|755068732~Geomicrobium_sp__JCM_19038
gi|636730578|dbj|GAK04410_1|~Geomicrobium_sp__JCM_19037
gi|517754606~Salsuginibacillus_kocurii
gi|847261987~Bacillus_sp__LL01
gi|506360842~Exiguobacterium_sp__AT1b
gi|916939204~Exiguobacterium_marinum
gi|544632492~Exiguobacterium_pavilionensis_RW-2
gi|334180190~Caldalkalibacillus_thermarum_TA2_A1
gi|757130988~Caldalkalibacillus_thermarum
gi|974004740|dbj|BAU29898_1|~Aneurinibacillus_soli
gi|1025929724~Paenibacillus_sp__LPB0068
gi|767003523~Paenibacillus_sp__IHBB_10380
gi|738754774~Paenibacillus_pini_JCM_16418
gi|995929800~Tepidibacillus_decaturensis
gi|1054313618~Balneola_sp__EhC07
gi|516844340~Balneola_vulgaris
gi|938259858~Bacteroidetes_bacterium_HLUCCA01
gi|504668856~Melioribacter_roseus_P3M-2
gi|371719703~Caldithrix_abyssi_DSM_13497
gi|1001925245~Halobacteroidaceae_bacterium_T328-2
gi|653089620~Halonatronum_saccharophilum
gi|491629420~Halanaerobium_saccharolyticum_subsp__saccharolyticum_DSM_6643
gi|501768531~Halothermothrix_orenii_H_168
gi|973039033~Petrotoga_mobilis
gi|769965094~Defluviitoga_tunisiensis
gi|504063068~Marinitoga_piezophila_KA3
gi|1054975490~Sneathia_sanguinegens
gi|740881178~Pelosinus_sp__UFO1
gi|654417730~Megamonas_hypermegale
gi|973154124~Anaerolinaceae_bacterium_46_22
gi|836602003~Kosmotoga_pacifica
gi|503653198~Flexistipes_sinusarabici_DSM_4947
GSMAST4A2_ASY1_ANO1_7397_1
0.2

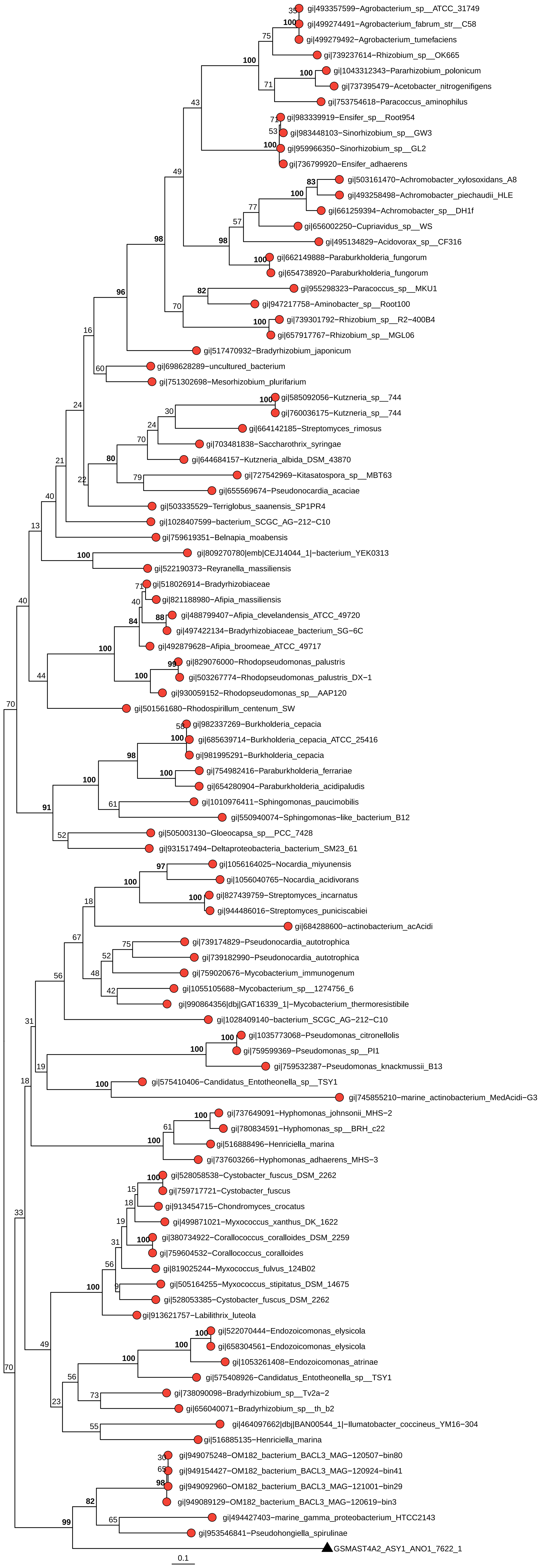

gi|493357599−Agrobacterium_sp__ATCC_31749
gi|499274491−Agrobacterium_fabrum_str__C58
gi|499279492−Agrobacterium_tumefaciens
gi|739237614−Rhizobium_sp__OK665
gi|1043312343−Pararhizobium_polonicum
gi|737395479−Acetobacter_nitrogenifigens
gi|753754618−Paracoccus_aminophilus
gi|983339919−Ensifer_sp__Root954
gi|983448103−Sinorhizobium_sp__GW3
gi|959966350−Sinorhizobium_sp__GL2
gi|736799920−Ensifer_adhaerens
gi|503161470−Achromobacter_xylosoxidans_A8
gi|493258498−Achromobacter_piechaudii_HLE
gi|661259394−Achromobacter_sp__DH1f
gi|656002250−Cupriavidus_sp__WS
gi|495134829−Acidovorax_sp__CF316
gi|662149888−Paraburkholderia_fungorum
gi|654738920−Paraburkholderia_fungorum
gi|955298323−Paracoccus_sp__MKU1
gi|947217758−Aminobacter_sp__Root100
gi|739301792−Rhizobium_sp__R2−400B4
gi|657917767−Rhizobium_sp__MGL06
gi|517470932−Bradyrhizobium_japonicum
gi|698628289−uncultured_bacterium
gi|751302698−Mesorhizobium_plurifarium
gi|585092056−Kutzneria_sp__744
gi|760036175−Kutzneria_sp__744
gi|664142185−Streptomyces_rimosus
gi|703481838−Saccharothrix_syringae
gi|644684157−Kutzneria_albida_DSM_43870
gi|727542969−Kitasatospora_sp__MBT63
gi|655569674−Pseudonocardia_acaciae
gi|503335529−Terriglobus_saanensis_SP1PR4
gi|1028407599−bacterium_SCGC_AG−212−C10
gi|759619351−Belnapia_moabensis
gi|809270780|emb|CEJ14044_1|−bacterium_YEK0313
gi|522190373−Reyranella_massiliensis
gi|518026914−Bradyrhizobiaceae
gi|821188980−Afipia_massiliensis
gi|488799407−Afipia_clevelandensis_ATCC_49720
gi|497422134−Bradyrhizobiaceae_bacterium_SG−6C
gi|492879628−Afipia_broomeae_ATCC_49717
gi|829076000−Rhodopseudomonas_palustris
gi|503267774−Rhodopseudomonas_palustris_DX−1
gi|930059152−Rhodopseudomonas_sp__AAP120
gi|501561680−Rhodospirillum_centenum_SW
gi|982337269−Burkholderia_cepacia
gi|685639714−Burkholderia_cepacia_ATCC_25416
gi|981995291−Burkholderia_cepacia
gi|754982416−Paraburkholderia_ferrariae
gi|654280904−Paraburkholderia_acidipaludis
gi|1010976411−Sphingomonas_paucimobilis
gi|550940074−Sphingomonas−like_bacterium_B12
gi|505003130−Gloeocapsa_sp__PCC_7428
gi|931517494−Deltaproteobacteria_bacterium_SM23_61
gi|1056164025−Nocardia_miyunensis
gi|1056040765−Nocardia_acidivorans
gi|827439759−Streptomyces_incarnatus
gi|944486016−Streptomyces_puniciscabiei
gi|684288600−actinobacterium_acAcidi
gi|739174829−Pseudonocardia_autotrophica
gi|739182990−Pseudonocardia_autotrophica
gi|759020676−Mycobacterium_immunogenum
gi|1055105688−Mycobacterium_sp__1274756_6
gi|990864356|dbj|GAT16339_1|−Mycobacterium_thermoresistibile
gi|1028409140−bacterium_SCGC_AG−212−C10
gi|1035773068−Pseudomonas_citronellolis
gi|759599369−Pseudomonas_sp__PI1
gi|759532387−Pseudomonas_knackmussii_B13
gi|575410406−Candidatus_Entotheonella_sp__TSY1
gi|745855210−marine_actinobacterium_MedAcidi−G3
gi|737649091−Hyphomonas_johnsonii_MHS−2
gi|780834591−Hyphomonas_sp__BRH_c22
gi|516888496−Henriciella_marina
gi|737603266−Hyphomonas_adhaerens_MHS−3
gi|528058538−Cystobacter_fuscus_DSM_2262
gi|759717721−Cystobacter_fuscus
gi|913454715−Chondromyces_crocatus
gi|499871021−Myxococcus_xanthus_DK_1622
gi|380734922−Corallococcus_coralloides_DSM_2259
gi|759604532−Corallococcus_coralloides
gi|819025244−Myxococcus_fulvus_124B02
gi|505164255−Myxococcus_stipitatus_DSM_14675
gi|528053385−Cystobacter_fuscus_DSM_2262
gi|913621757−Labilithrix_luteola
gi|522070444−Endozoicomonas_elysicola
gi|658304561−Endozoicomonas_elysicola
gi|1053261408−Endozoicomonas_atrinae
gi|575408926−Candidatus_Entotheonella_sp__TSY1
gi|738090098−Bradyrhizobium_sp__Tv2a−2
gi|656040071−Bradyrhizobium_sp__th_b2
gi|464097662|dbj|BAN00544_1|−Ilumatobacter_coccineus_YM16−304
gi|516885135−Henriciella_marina
gi|949075248−OM182_bacterium_BACL3_MAG−120507−bin80
gi|949154427−OM182_bacterium_BACL3_MAG−120924−bin41
gi|949092960−OM182_bacterium_BACL3_MAG−121001−bin29
gi|949089129−OM182_bacterium_BACL3_MAG−120619−bin3
gi|494427403−marine_gamma_proteobacterium_HTCC2143
gi|953546841−Pseudohongiella_spirulinae
GSMAST4A2_ASY1_ANO1_7622_1
0.1

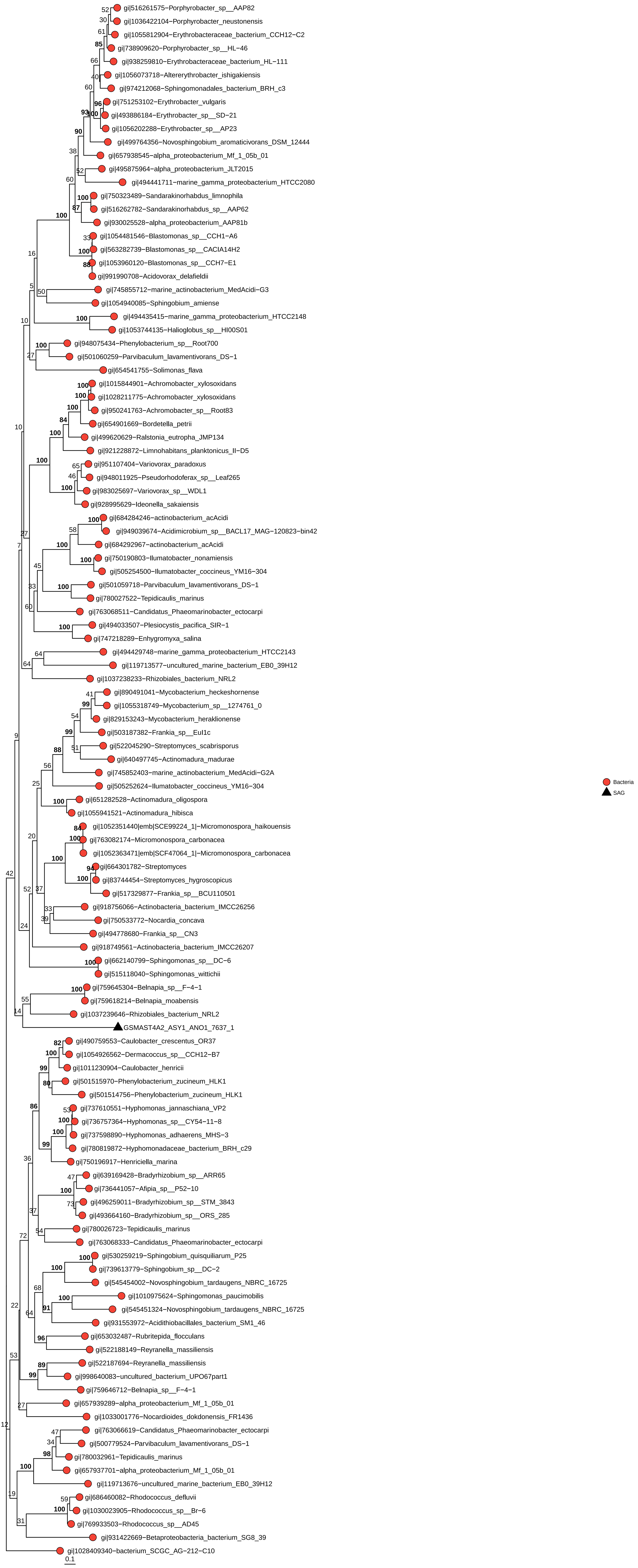

gi|516261575−Porphyrobacter_sp__AAP82
gi|1036422104−Porphyrobacter_neustonensis
gi|1055812904−Erythrobacteraceae_bacterium_CCH12−C2
gi|738909620−Porphyrobacter_sp__HL−46
gi|938259810−Erythrobacteraceae_bacterium_HL−111
gi|1056073718−Altererythrobacter_ishigakiensis
gi|974212068−Sphingomonadales_bacterium_BRH_c3
gi|751253102−Erythrobacter_vulgaris
gi|493886184−Erythrobacter_sp__SD−21
gi|1056202288−Erythrobacter_sp__AP23
gi|499764356−Novosphingobium_aromaticivorans_DSM_12444
gi|657938545−alpha_proteobacterium_Mf_1_05b_01
gi|495875964−alpha_proteobacterium_JLT2015
gi|494441711−marine_gamma_proteobacterium_HTCC2080
gi|750323489−Sandarakinorhabdus_limnophila
gi|516262782−Sandarakinorhabdus_sp__AAP62
gi|930025528−alpha_proteobacterium_AAP81b
gi|1054481546−Blastomonas_sp__CCH1−A6
gi|563282739−Blastomonas_sp__CACIA14H2
gi|1053960120−Blastomonas_sp__CCH7−E1
gi|991990708−Acidovorax_delafieldii
gi|745855712−marine_actinobacterium_MedAcidi−G3
gi|1054940085−Sphingobium_amiense
gi|494435415−marine_gamma_proteobacterium_HTCC2148
gi|1053744135−Halioglobus_sp__HI00S01
gi|948075434−Phenylobacterium_sp__Root700
gi|501060259−Parvibaculum_lavamentivorans_DS−1
gi|654541755−Solimonas_flava
gi|1015844901−Achromobacter_xylosoxidans
gi|1028211775−Achromobacter_xylosoxidans
gi|950241763−Achromobacter_sp__Root83
gi|654901669−Bordetella_petrii
gi|499620629−Ralstonia_eutropha_JMP134
gi|921228872−Limnohabitans_planktonicus_II−D5
gi|951107404−Variovorax_paradoxus
gi|948011925−Pseudorhodoferax_sp__Leaf265
gi|983025697−Variovorax_sp__WDL1
gi|928995629−Ideonella_sakaiensis
gi|684284246−actinobacterium_acAcidi
gi|949039674−Acidimicrobium_sp__BACL17_MAG−120823−bin42
gi|684292967−actinobacterium_acAcidi
gi|750190803−Ilumatobacter_nonamiensis
gi|505254500−Ilumatobacter_coccineus_YM16−304
gi|501059718−Parvibaculum_lavamentivorans_DS−1
gi|780027522−Tepidicaulis_marinus
gi|763068511−Candidatus_Phaeomarinobacter_ectocarpi
gi|494033507−Plesiocystis_pacifica_SIR−1
gi|747218289−Enhygromyxa_salina
gi|494429748−marine_gamma_proteobacterium_HTCC2143
gi|119713577−uncultured_marine_bacterium_EB0_39H12
gi|1037238233−Rhizobiales_bacterium_NRL2
gi|890491041−Mycobacterium_heckeshornense
gi|1055318749−Mycobacterium_sp__1274761_0
gi|829153243−Mycobacterium_heraklionense
gi|503187382−Frankia_sp__EuI1c
gi|522045290−Streptomyces_scabrisporus
gi|640497745−Actinomadura_madurae
gi|745852403−marine_actinobacterium_MedAcidi−G2A
gi|505252624−Ilumatobacter_coccineus_YM16−304
gi|651282528−Actinomadura_oligospora
gi|1055941521−Actinomadura_hibisca
gi|1052351440|emb|SCE99224_1|−Micromonospora_haikouensis
gi|763082174−Micromonospora_carbonacea
gi|1052363471|emb|SCF47064_1|−Micromonospora_carbonacea
gi|664301782−Streptomyces
gi|83744454−Streptomyces_hygroscopicus
gi|517329877−Frankia_sp__BCU110501
gi|918756066−Actinobacteria_bacterium_IMCC26256
gi|750533772−Nocardia_concava
gi|494778680−Frankia_sp__CN3
gi|918749561−Actinobacteria_bacterium_IMCC26207
gi|662140799−Sphingomonas_sp__DC−6
gi|515118040−Sphingomonas_wittichii
gi|759645304−Belnapia_sp__F−4−1
gi|759618214−Belnapia_moabensis
gi|1037239646−Rhizobiales_bacterium_NRL2
GSMAST4A2_ASY1_ANO1_7637_1
gi|490759553−Caulobacter_crescentus_OR37
gi|1054926562−Dermacoccus_sp__CCH12−B7
gi|1011230904−Caulobacter_henricii
gi|501515970−Phenylobacterium_zucineum_HLK1
gi|501514756−Phenylobacterium_zucineum_HLK1
gi|737610551−Hyphomonas_jannaschiana_VP2
gi|736757364−Hyphomonas_sp__CY54−11−8
gi|737598890−Hyphomonas_adhaerens_MHS−3
gi|780819872−Hyphomonadaceae_bacterium_BRH_c29
gi|750196917−Henriciella_marina
gi|639169428−Bradyrhizobium_sp__ARR65
gi|736441057−Afipia_sp__P52−10
gi|496259011−Bradyrhizobium_sp__STM_3843
gi|493664160−Bradyrhizobium_sp__ORS_285
gi|780026723−Tepidicaulis_marinus
gi|763068333−Candidatus_Phaeomarinobacter_ectocarpi
gi|530259219−Sphingobium_quisquiliarum_P25
gi|739613779−Sphingobium_sp__DC−2
gi|545454002−Novosphingobium_tardaugens_NBRC_16725
gi|1010975624−Sphingomonas_paucimobilis
gi|545451324−Novosphingobium_tardaugens_NBRC_16725
gi|931553972−Acidithiobacillales_bacterium_SM1_46
gi|653032487−Rubritepida_flocculans
gi|522188149−Reyranella_massiliensis
gi|522187694−Reyranella_massiliensis
gi|998640083−uncultured_bacterium_UPO67part1
gi|759646712−Belnapia_sp__F−4−1
gi|657939289−alpha_proteobacterium_Mf_1_05b_01
gi|1033001776−Nocardioides_dokdonensis_FR1436
gi|763066619−Candidatus_Phaeomarinobacter_ectocarpi
gi|500779524−Parvibaculum_lavamentivorans_DS−1
gi|780032961−Tepidicaulis_marinus
gi|657937701−alpha_proteobacterium_Mf_1_05b_01
gi|119713676−uncultured_marine_bacterium_EB0_39H12
gi|686460082−Rhodococcus_defluvii
gi|1030023905−Rhodococcus_sp__Br−6
gi|769933503−Rhodococcus_sp__AD45
gi|931422669−Betaproteobacteria_bacterium_SG8_39
gi|1028409340−bacterium_SCGC_AG−212−C10
0.1
Bacteria
SAG

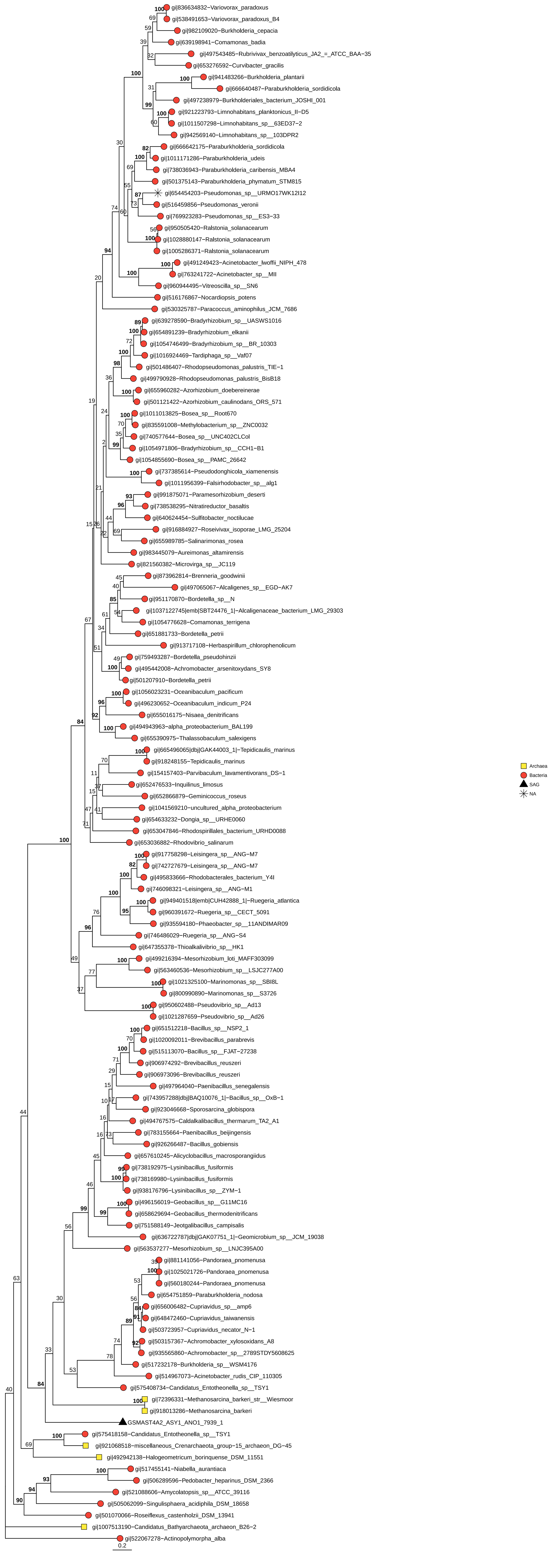
gi|836634832−Variovorax_paradoxus
gi|538491653−Variovorax_paradoxus_B4
gi|982109020−Burkholderia_cepacia
gi|639198941−Comamonas_badia
gi|497543485−Rubrivivax_benzoatilyticus_JA2_=_ATCC_BAA−35
gi|653276592−Curvibacter_gracilis
gi|941483266−Burkholderia_plantarii
gi|666640487−Paraburkholderia_sordidicola
gi|497238979−Burkholderiales_bacterium_JOSHI_001
gi|921223793−Limnohabitans_planktonicus_II−D5
gi|1011507298−Limnohabitans_sp__63ED37−2
gi|942569140−Limnohabitans_sp__103DPR2
gi|666642175−Paraburkholderia_sordidicola
gi|1011171286−Paraburkholderia_udeis
gi|738036943−Paraburkholderia_caribensis_MBA4
gi|501375143−Paraburkholderia_phymatum_STM815
gi|654454203−Pseudomonas_sp__URMO17WK12I12
gi|516459856−Pseudomonas_veronii
gi|769923283−Pseudomonas_sp__ES3−33
gi|950505420−Ralstonia_solanacearum
gi|1028880147−Ralstonia_solanacearum
gi|1005286371−Ralstonia_solanacearum
gi|491249423−Acinetobacter_lwoffii_NIPH_478
gi|763241722−Acinetobacter_sp__MII
gi|960944495−Vitreoscilla_sp__SN6
gi|516176867−Nocardiopsis_potens
gi|530325787−Paracoccus_aminophilus_JCM_7686
gi|639278590−Bradyrhizobium_sp__UASWS1016
gi|654891239−Bradyrhizobium_elkanii
gi|1054746499−Bradyrhizobium_sp__BR_10303
gi|1016924469−Tardiphaga_sp__Vaf07
gi|501486407−Rhodopseudomonas_palustris_TIE−1
gi|499790928−Rhodopseudomonas_palustris_BisB18
gi|655960282−Azorhizobium_doebereinerae
gi|501121422−Azorhizobium_caulinodans_ORS_571
gi|1011013825−Bosea_sp__Root670
gi|835591008−Methylobacterium_sp__ZNC0032
gi|740577644−Bosea_sp__UNC402CLCol
gi|1054971806−Bradyrhizobium_sp__CCH1−B1
gi|1054855690−Bosea_sp__PAMC_26642
gi|737385614−Pseudodonghicola_xiamenensis
gi|1011956399−Falsirhodobacter_sp__alg1
gi|991875071−Paramesorhizobium_deserti
gi|738538295−Nitratireductor_basaltis
gi|640624454−Sulfitobacter_noctilucae
gi|916884927−Roseivivax_isoporae_LMG_25204
gi|655989785−Salinarimonas_rosea
gi|983445079−Aureimonas_altamirensis
gi|821560382−Microvirga_sp__JC119
gi|873962814−Brenneria_goodwinii
gi|497065067−Alcaligenes_sp__EGD−AK7
gi|951170870−Bordetella_sp__N
gi|1037122745|emb|SBT24476_1|−Alcaligenaceae_bacterium_LMG_29303
gi|1054776628−Comamonas_terrigena
gi|651881733−Bordetella_petrii
gi|913717108−Herbaspirillum_chlorophenolicum
gi|759493287−Bordetella_pseudohinzii
gi|495442008−Achromobacter_arsenitoxydans_SY8
gi|501207910−Bordetella_petrii
gi|1056023231−Oceanibaculum_pacificum
gi|496230652−Oceanibaculum_indicum_P24
gi|655016175−Nisaea_denitrificans
gi|494943963−alpha_proteobacterium_BAL199
gi|655390975−Thalassobaculum_salexigens
gi|665496065|dbj|GAK44003_1|−Tepidicaulis_marinus
gi|918248155−Tepidicaulis_marinus
gi|154157403−Parvibaculum_lavamentivorans_DS−1
gi|652476533−Inquilinus_limosus
gi|652866879−Geminicoccus_roseus
gi|1041569210−uncultured_alpha_proteobacterium
gi|654633232−Dongia_sp__URHE0060
gi|653047846−Rhodospirillales_bacterium_URHD0088
gi|653036882−Rhodovibrio_salinarum
gi|917758298−Leisingera_sp__ANG−M7
gi|742727679−Leisingera_sp__ANG−M7
gi|495833666−Rhodobacterales_bacterium_Y4I
gi|746098321−Leisingera_sp__ANG−M1
gi|949401518|emb|CUH42888_1|−Ruegeria_atlantica
gi|960391672−Ruegeria_sp__CECT_5091
gi|935594180−Phaeobacter_sp__11ANDIMAR09
gi|746486029−Ruegeria_sp__ANG−S4
gi|647355378−Thioalkalivibrio_sp__HK1
gi|499216394−Mesorhizobium_loti_MAFF303099
gi|563460536−Mesorhizobium_sp__LSJC277A00
gi|1021325100−Marinomonas_sp__SBI8L
gi|800990890−Marinomonas_sp__S3726
gi|950602488−Pseudovibrio_sp__Ad13
gi|1021287659−Pseudovibrio_sp__Ad26
gi|651512218−Bacillus_sp__NSP2_1
gi|1020092011−Brevibacillus_parabrevis
gi|515113070−Bacillus_sp__FJAT−27238
gi|906974292−Brevibacillus_reuszeri
gi|906973096−Brevibacillus_reuszeri
gi|497964040−Paenibacillus_senegalensis
gi|743957288|dbj|BAQ10076_1|−Bacillus_sp__OxB−1
gi|923046668−Sporosarcina_globispora
gi|494767575−Caldalkalibacillus_thermarum_TA2_A1
gi|783155664−Paenibacillus_beijingensis
gi|926266487−Bacillus_gobiensis
gi|657610245−Alicyclobacillus_macrosporangiidus
gi|738192975−Lysinibacillus_fusiformis
gi|738169980−Lysinibacillus_fusiformis
gi|938176796−Lysinibacillus_sp__ZYM−1
gi|496156019−Geobacillus_sp__G11MC16
gi|658629694−Geobacillus_thermodenitrificans
gi|751588149−Jeotgalibacillus_campisalis
gi|636722787|dbj|GAK07751_1|−Geomicrobium_sp__JCM_19038
gi|563537277−Mesorhizobium_sp__LNJC395A00
gi|881141056−Pandoraea_pnomenusa
gi|1025021726−Pandoraea_pnomenusa
gi|560180244−Pandoraea_pnomenusa
gi|654751859−Paraburkholderia_nodosa
gi|656006482−Cupriavidus_sp__amp6
gi|648472460−Cupriavidus_taiwanensis
gi|503723957−Cupriavidus_necator_N−1
gi|503157367−Achromobacter_xylosoxidans_A8
gi|935565860−Achromobacter_sp__2789STDY5608625
gi|517232178−Burkholderia_sp__WSM4176
gi|514967073−Acinetobacter_rudis_CIP_110305
gi|575408734−Candidatus_Entotheonella_sp__TSY1
gi|72396331−Methanosarcina_barkeri_str__Wiesmoor
gi|918013286−Methanosarcina_barkeri
GSMAST4A2_ASY1_ANO1_7939_1
gi|575418158−Candidatus_Entotheonella_sp__TSY1
gi|921068518−miscellaneous_Crenarchaeota_group−15_archaeon_DG−45
gi|492942138−Halogeometricum_borinquense_DSM_11551
gi|517455141−Niabella_aurantiaca
gi|506289596−Pedobacter_heparinus_DSM_2366
gi|521088606−Amycolatopsis_sp__ATCC_39116
gi|505062099−Singulisphaera_acidiphila_DSM_18658
gi|501070066−Roseiflexus_castenholzii_DSM_13941
gi|1007513190−Candidatus_Bathyarchaeota_archaeon_B26−2
gi|522067278−Actinopolymorpha_alba
0.2
Archaea
Bacteria
SAG
NA

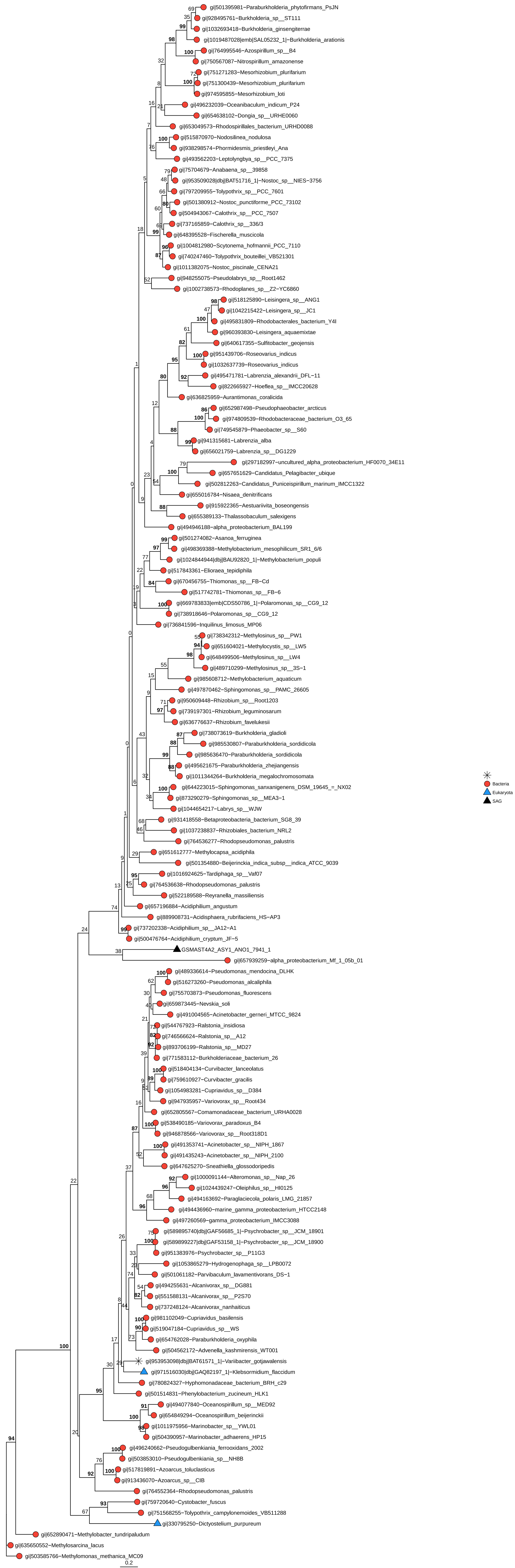

gi|501395981–Paraburkholderia_phytofirmans_PsJN
gi|928495761–Burkholderia_sp__ST111
gi|1032693418–Burkholderia_ginsengiterrae
gi|1019487028|emb|SAL05232_1|–Burkholderia_arationis
gi|764995546–Azospirillum_sp__B4
gi|750567087–Nitrospirillum_amazonense
gi|751271283–Mesorhizobium_plurifarium
gi|751300439–Mesorhizobium_plurifarium
gi|974595855–Mesorhizobium_loti
gi|496232039–Oceanibaculum_indicum_P24
gi|654638102–Dongia_sp__URHE0060
gi|653049573–Rhodospirillales_bacterium_URHD0088
gi|515870970–Nodosilinea_nodulosa
gi|938298574–Phormidesmis_priestleyi_Ana
gi|493562203–Leptolyngbya_sp__PCC_7375
gi|75704679–Anabaena_sp__39858
gi|953509028|dbj|BAT51716_1|–Nostoc_sp__NIES–3756
gi|797209955–Tolypothrix_sp__PCC_7601
gi|501380912–Nostoc_punctiforme_PCC_73102
gi|504943067–Calothrix_sp__PCC_7507
gi|737165859–Calothrix_sp__336/3
gi|648395528–Fischerella_muscicola
gi|1004812980–Scytonema_hofmannii_PCC_7110
gi|740247460–Tolypothrix_bouteillei_VB521301
gi|1011382075–Nostoc_piscinale_CENA21
gi|948255075–Pseudolabrys_sp__Root1462
gi|1002738573–Rhodoplanes_sp__Z2–YC6860
gi|518125890–Leisingera_sp__ANG1
gi|1042215422–Leisingera_sp__JC1
gi|495831809–Rhodobacterales_bacterium_Y4I
gi|960393830–Leisingera_aquaemixtae
gi|640617355–Sulfitobacter_geojensis
gi|951439706–Roseovarius_indicus
gi|1032637739–Roseovarius_indicus
gi|495471781–Labrenzia_alexandrii_DFL–11
gi|822665927–Hoeflea_sp__IMCC20628
gi|636825959–Aurantimonas_coralicida
gi|652987498–Pseudophaeobacter_arcticus
gi|974809539–Rhodobacteraceae_bacterium_O3_65
gi|749545879–Phaeobacter_sp__S60
gi|941315681–Labrenzia_alba
gi|656021759–Labrenzia_sp__DG1229
gi|297182997–uncultured_alpha_proteobacterium_HF0070_34E11
gi|657651629–Candidatus_Pelagibacter_ubique
gi|502812263–Candidatus_Puniceispirillum_marinum_IMCC1322
gi|655016784–Nisaea_denitrificans
gi|915922365–Aestuariivita_boseongensis
gi|655389133–Thalassobaculum_salexigens
gi|494946188–alpha_proteobacterium_BAL199
gi|501274082–Asanoa_ferruginea
gi|498369388–Methylobacterium_mesophilicum_SR1_6/6
gi|1024844944|dbj|BAU92820_1|–Methylobacterium_populi
gi|517843361–Elioraea_tepidiphila
gi|670456755–Thiomonas_sp__FB–Cd
gi|517742781–Thiomonas_sp__FB–6
gi|669783833|emb|CDS50786_1|–Polaromonas_sp__CG9_12
gi|738918646–Polaromonas_sp__CG9_12
gi|736841596–Inquilinus_limosus_MP06
gi|738342312–Methylosinus_sp__PW1
gi|651604021–Methylocystis_sp__LW5
gi|648499506–Methylosinus_sp__LW4
gi|489710299–Methylosinus_sp__3S–1
gi|985608712–Methylobacterium_aquaticum
gi|497870462–Sphingomonas_sp__PAMC_26605
gi|950609448–Rhizobium_sp__Root1203
gi|739197301–Rhizobium_leguminosarum
gi|636776637–Rhizobium_favelukesii
gi|738073619–Burkholderia_gladioli
gi|985530807–Paraburkholderia_sordidicola
gi|985636470–Paraburkholderia_sordidicola
gi|495621675–Paraburkholderia_zhejiangensis
gi|1011344264–Burkholderia_megalochromosomata
gi|644223015–Sphingomonas_sanxanigenens_DSM_19645_=_NX02
gi|873290279–Sphingomonas_sp__MEA3–1
gi|1044654217–Labrys_sp__WJW
gi|931418558–Betaproteobacteria_bacterium_SG8_39
gi|1037238837–Rhizobiales_bacterium_NRL2
gi|764536277–Rhodopseudomonas_palustris
gi|651612777–Methylocapsa_acidiphila
gi|501354880–Beijerinckia_indica_subsp__indica_ATCC_9039
gi|1016924625–Tardiphaga_sp__Vaf07
gi|764536638–Rhodopseudomonas_palustris
gi|522189588–Reyranella_massiliensis
gi|657196884–Acidiphilium_angustum
gi|889908731–Acidisphaera_rubrifaciens_HS–AP3
gi|737202338–Acidiphilium_sp__JA12–A1
gi|500476764–Acidiphilium_cryptum_JF–5
GSMAST4A2_ASY1_ANO1_7941_1
gi|657939259–alpha_proteobacterium_Mf_1_05b_01
gi|489336614–Pseudomonas_mendocina_DLHK
gi|516273260–Pseudomonas_alcaliphila
gi|755703873–Pseudomonas_fluorescens
gi|659873445–Nevskia_soli
gi|491004565–Acinetobacter_gerneri_MTCC_9824
gi|544767923–Ralstonia_insidiosa
gi|746566624–Ralstonia_sp__A12
gi|893706199–Ralstonia_sp__MD27
gi|771583112–Burkholderiaceae_bacterium_26
gi|518404134–Curvibacter_lanceolatus
gi|759610927–Curvibacter_gracilis
gi|1054983281–Cupriavidus_sp__D384
gi|947935957–Variovorax_sp__Root434
gi|652805567–Comamonadaceae_bacterium_URHA0028
gi|538490185–Variovorax_paradoxus_B4
gi|946878566–Variovorax_sp__Root318D1
gi|491353741–Acinetobacter_sp__NIPH_1867
gi|491435243–Acinetobacter_sp__NIPH_2100
gi|647625270–Sneathiella_glossodoripedis
gi|1000091144–Alteromonas_sp__Nap_26
gi|1024439247–Oleiphilus_sp__HI0125
gi|494163692–Paraglaciecola_polaris_LMG_21857
gi|494436960–marine_gamma_proteobacterium_HTCC2148
gi|497260569–gamma_proteobacterium_IMCC3088
gi|589895740|dbj|GAF56685_1|–Psychrobacter_sp__JCM_18901
gi|589899227|dbj|GAF53158_1|–Psychrobacter_sp__JCM_18900
gi|951383976–Psychrobacter_sp__P11G3
gi|1053865279–Hydrogenophaga_sp__LPB0072
gi|501061182–Parvibaculum_lavamentivorans_DS–1
gi|494255631–Alcanivorax_sp__DG881
gi|551588131–Alcanivorax_sp__P2S70
gi|737248124–Alcanivorax_nanhaiticus
gi|981102049–Cupriavidus_basilensis
gi|519047184–Cupriavidus_sp__WS
gi|654762028–Paraburkholderia_oxyphila
gi|504562172–Advenella_kashmirensis_WT001
gi|953953098|dbj|BAT61571_1|–Variibacter_gotjawalensis
gi|971516030|dbj|GAQ82197_1|–Klebsormidium_flaccidum
gi|780824327–Hyphomonadaceae_bacterium_BRH_c29
gi|501514831–Phenylobacterium_zucineum_HLK1
gi|494077840–Oceanospirillum_sp__MED92
gi|654849294–Oceanospirillum_beijerinckii
gi|1011975956–Marinobacter_sp__YWL01
gi|504390957–Marinobacter_adhaerens_HP15
gi|496240662–Pseudogulbenkiania_ferrooxidans_2002
gi|503853010–Pseudogulbenkiania_sp__NH8B
gi|517819891–Azoarcus_toluclasticus
gi|913436070–Azoarcus_sp__CIB
gi|764552364–Rhodopseudomonas_palustris
gi|759720640–Cystobacter_fuscus
gi|751568255–Tolypothrix_campylonemoides_VB511288
gi|330795250–Dictyostelium_purpureum
gi|652890471–Methylobacter_tundripaludum
gi|635650552–Methylosarcina_lacus
gi|503585766–Methylomonas_methanica_MC09
0.2
Bacteria
Eukaryota
SAG

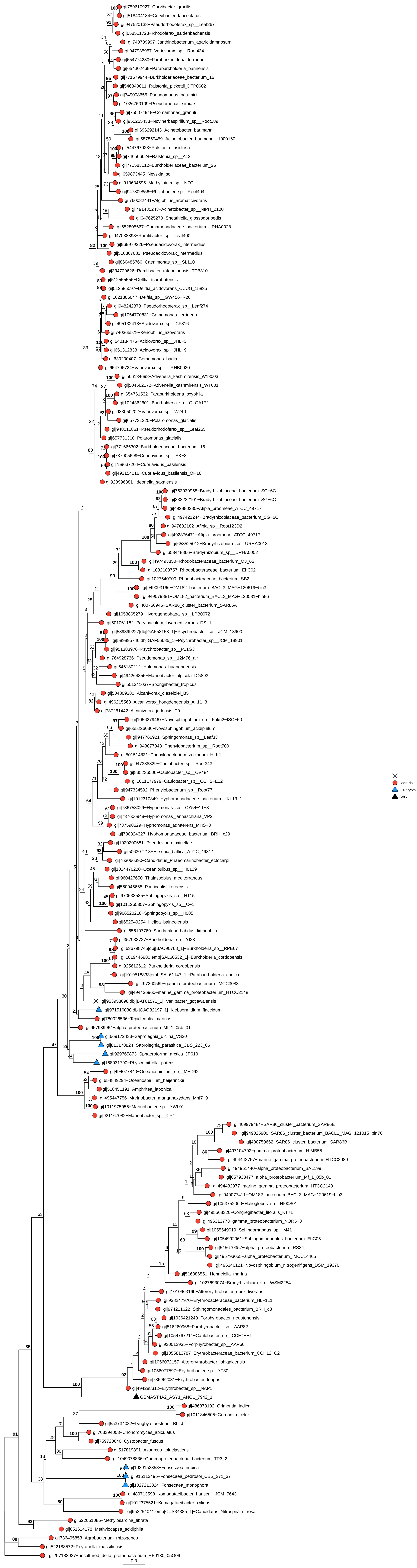
gi|759610927−Curvibacter_gracilis
gi|518404134−Curvibacter_lanceolatus
gi|947520138−Pseudorhodoferax_sp__Leaf267
gi|658511723−Rhodoferax_saidenbachensis
gi|740709997−Janthinobacterium_agaricidamnosum
gi|947935957−Variovorax_sp__Root434
gi|654774280−Paraburkholderia_ferrariae
gi|654302469−Paraburkholderia_bannensis
gi|771679944−Burkholderiaceae_bacterium_16
gi|546340811−Ralstonia_pickettii_DTP0602
gi|749008655−Pseudomonas_batumici
gi|1026750109−Pseudomonas_simiae
gi|755074948−Comamonas_granuli
gi|950255438−Noviherbaspirillum_sp__Root189
gi|696292143−Acinetobacter_baumannii
gi|587859459−Acinetobacter_baumannii_1000160
gi|544767923−Ralstonia_insidiosa
gi|746566624−Ralstonia_sp__A12
gi|771583112−Burkholderiaceae_bacterium_26
gi|659873445−Nevskia_soli
gi|913634595−Methylibium_sp__NZG
gi|947809856−Rhizobacter_sp__Root404
gi|760082441−Algiphilus_aromaticivorans
gi|491435243−Acinetobacter_sp__NIPH_2100
gi|647625270−Sneathiella_glossodoripedis
gi|652805567−Comamonadaceae_bacterium_URHA0028
gi|947038393−Ramlibacter_sp__Leaf400
gi|969979326−Pseudacidovorax_intermedius
gi|516367083−Pseudacidovorax_intermedius
gi|860485766−Caenimonas_sp__SL110
gi|334729626−Ramlibacter_tataouinensis_TTB310
gi|512555556−Delftia_tsuruhatensis
gi|512585097−Delftia_acidovorans_CCUG_15835
gi|1021306047−Delftia_sp__GW456−R20
gi|948242878−Pseudorhodoferax_sp__Leaf274
gi|1054770831−Comamonas_terrigena
gi|495132413−Acidovorax_sp__CF316
gi|740365579−Xenophilus_azovorans
gi|640184476−Acidovorax_sp__JHL−3
gi|651312838−Acidovorax_sp__JHL−9
gi|639200407−Comamonas_badia
gi|654796724−Variovorax_sp__URHB0020
gi|566134698−Advenella_kashmirensis_W13003
gi|504562172−Advenella_kashmirensis_WT001
gi|654761532−Paraburkholderia_oxyphila
gi|1024362601−Burkholderia_sp__OLGA172
gi|983050202−Variovorax_sp__WDL1
gi|657731325−Polaromonas_glacialis
gi|948011861−Pseudorhodoferax_sp__Leaf265
gi|657731310−Polaromonas_glacialis
gi|771665302−Burkholderiaceae_bacterium_16
gi|737905699−Cupriavidus_sp__SK−3
gi|759637204−Cupriavidus_basilensis
gi|493154016−Cupriavidus_basilensis_OR16
gi|928996381−Ideonella_sakaiensis
gi|763039958−Bradyrhizobiaceae_bacterium_SG−6C
gi|338232101−Bradyrhizobiaceae_bacterium_SG−6C
gi|492880380−Afipia_broomeae_ATCC_49717
gi|497421244−Bradyrhizobiaceae_bacterium_SG−6C
gi|947632182−Afipia_sp__Root123D2
gi|492876471−Afipia_broomeae_ATCC_49717
gi|653525012−Bradyrhizobium_sp__URHA0013
gi|653448866−Bradyrhizobium_sp__URHA0002
gi|497493850−Rhodobacteraceae_bacterium_O3_65
gi|1032100757−Rhodobacteraceae_bacterium_EhC02
gi|1027540700−Rhodobacteraceae_bacterium_SB2
gi|949093166−OM182_bacterium_BACL3_MAG−120619−bin3
gi|949079881−OM182_bacterium_BACL3_MAG−120531−bin86
gi|400756946−SAR86_cluster_bacterium_SAR86A
gi|1053865279−Hydrogenophaga_sp__LPB0072
gi|501061182−Parvibaculum_lavamentivorans_DS−1
gi|589899227|dbj|GAF53158_1|−Psychrobacter_sp__JCM_18900
gi|589895740|dbj|GAF56685_1|−Psychrobacter_sp__JCM_18901
gi|951383976−Psychrobacter_sp__P11G3
gi|764928736−Pseudomonas_sp__12M76_air
gi|546180212−Halomonas_huangheensis
gi|494264855−Marinobacter_algicola_DG893
gi|551341037−Spongiibacter_tropicus
gi|504809380−Alcanivorax_dieselolei_B5
gi|496215563−Alcanivorax_hongdengensis_A−11−3
gi|737261442−Alcanivorax_jadensis_T9
gi|1056279467−Novosphingobium_sp__Fuku2−ISO−50
gi|655226036−Novosphingobium_acidiphilum
gi|947766921−Sphingomonas_sp__Leaf33
gi|948077048−Phenylobacterium_sp__Root700
gi|501514831−Phenylobacterium_zucineum_HLK1
gi|947388829−Caulobacter_sp__Root343
gi|835236506−Caulobacter_sp__OV484
gi|1011177979−Caulobacter_sp__CCH5−E12
gi|947334592−Phenylobacterium_sp__Root77
gi|1012310849−Hyphomonadaceae_bacterium_UKL13−1
gi|736758029−Hyphomonas_sp__CY54−11−8
gi|737606948−Hyphomonas_jannaschiana_VP2
gi|737598529−Hyphomonas_adhaerens_MHS−3
gi|780824327−Hyphomonadaceae_bacterium_BRH_c29
gi|1020200681−Pseudovibrio_axinellae
gi|506307218−Hirschia_baltica_ATCC_49814
gi|763066390−Candidatus_Phaeomarinobacter_ectocarpi
gi|1024476220−Oceanibulbus_sp__HI0129
gi|960427650−Thalassobius_mediterraneus
gi|550945665−Ponticaulis_koreensis
gi|970533585−Sphingopyxis_sp__H115
gi|1011265357−Sphingopyxis_sp__C−1
gi|966520218−Sphingopyxis_sp__H085
gi|652549254−Hellea_balneolensis
gi|656107760−Sandarakinorhabdus_limnophila
gi|357938727−Burkholderia_sp__YI23
gi|636798745|dbj|BAO90768_1|−Burkholderia_sp__RPE67
gi|1019446980|emb|SAL60532_1|−Burkholderia_cordobensis
gi|925612612−Burkholderia_cordobensis
gi|1019518833|emb|SAL61147_1|−Paraburkholderia_choica
gi|497260569−gamma_proteobacterium_IMCC3088
gi|494436960−marine_gamma_proteobacterium_HTCC2148
gi|953953098|dbj|BAT61571_1|−Variibacter_gotjawalensis
gi|971516030|dbj|GAQ82197_1|−Klebsormidium_flaccidum
gi|780026536−Tepidicaulis_marinus
gi|657939964−alpha_proteobacterium_Mf_1_05b_01
gi|669172433−Saprolegnia_diclina_VS20
gi|813178824−Saprolegnia_parasitica_CBS_223_65
gi|929765873−Sphaeroforma_arctica_JP610
gi|168031790−Physcomitrella_patens
gi|494077840−Oceanospirillum_sp__MED92
gi|654849294−Oceanospirillum_beijerinckii
gi|518451191−Amphritea_japonica
gi|495447756−Marinobacter_manganoxydans_MnI7−9
gi|1011975956−Marinobacter_sp__YWL01
gi|921167082−Marinobacter_sp__CP1
gi|409979484−SAR86_cluster_bacterium_SAR86E
gi|949025900−SAR86_cluster_bacterium_BACL1_MAG−121015−bin70
gi|400759662−SAR86_cluster_bacterium_SAR86B
gi|497104792−gamma_proteobacterium_HIMB55
gi|494442767−marine_gamma_proteobacterium_HTCC2080
gi|494951440−alpha_proteobacterium_BAL199
gi|657938477−alpha_proteobacterium_Mf_1_05b_01
gi|494432977−marine_gamma_proteobacterium_HTCC2143
gi|949077411−OM182_bacterium_BACL3_MAG−120619−bin3
gi|1053752060−Halioglobus_sp__HI00S01
gi|495568320−Congregibacter_litoralis_KT71
gi|496313773−gamma_proteobacterium_NOR5−3
gi|1055549019−Sphingorhabdus_sp__M41
gi|1054992061−Sphingomonadales_bacterium_EhC05
gi|545670357−alpha_proteobacterium_RS24
gi|495793055−alpha_proteobacterium_IMCC14465
gi|495346121−Novosphingobium_nitrogenifigens_DSM_19370
gi|516886551−Henriciella_marina
gi|1027693074−Bradyrhizobium_sp__WSM2254
gi|1010963169−Altererythrobacter_epoxidivorans
gi|938247970−Erythrobacteraceae_bacterium_HL−111
gi|974211622−Sphingomonadales_bacterium_BRH_c3
gi|1036421249−Porphyrobacter_neustonensis
gi|516260968−Porphyrobacter_sp__AAP82
gi|1054767211−Caulobacter_sp__CCH4−E1
gi|930012935−Porphyrobacter_sp__AAP60
gi|1055813787−Erythrobacteraceae_bacterium_CCH12−C2
gi|1056072157−Altererythrobacter_ishigakiensis
gi|1056077597−Erythrobacter_sp__YT30
gi|736962031−Erythrobacter_longus
gi|494288312−Erythrobacter_sp__NAP1
GSMAST4A2_ASY1_ANO1_7942_1
gi|486373102−Grimontia_indica
gi|1011846505−Grimontia_celer
gi|553734082−Lyngbya_aestuarii_BL_J
gi|763394003−Chondromyces_apiculatus
gi|759720640−Cystobacter_fuscus
gi|517819891−Azoarcus_toluclasticus
gi|1049078836−Gammaproteobacteria_bacterium_TR3_2
gi|1029152358−Fonsecaea_nubica
gi|915113495−Fonsecaea_pedrosoi_CBS_271_37
gi|1027213824−Fonsecaea_monophora
gi|489713598−Komagataeibacter_hansenii_JCM_7643
gi|1012375521−Komagataeibacter_xylinus
gi|953254041|emb|CUS34385_1|−Candidatus_Nitrospira_nitrosa
gi|522051086−Methylosarcina_fibrata
gi|651614178−Methylocapsa_acidiphila
gi|736495853−Agrobacterium_rhizogenes
gi|522188572−Reyranella_massiliensis
gi|297183037−uncultured_delta_proteobacterium_HF0130_05G09
0.3
Bacteria
Eukaryota
SAG

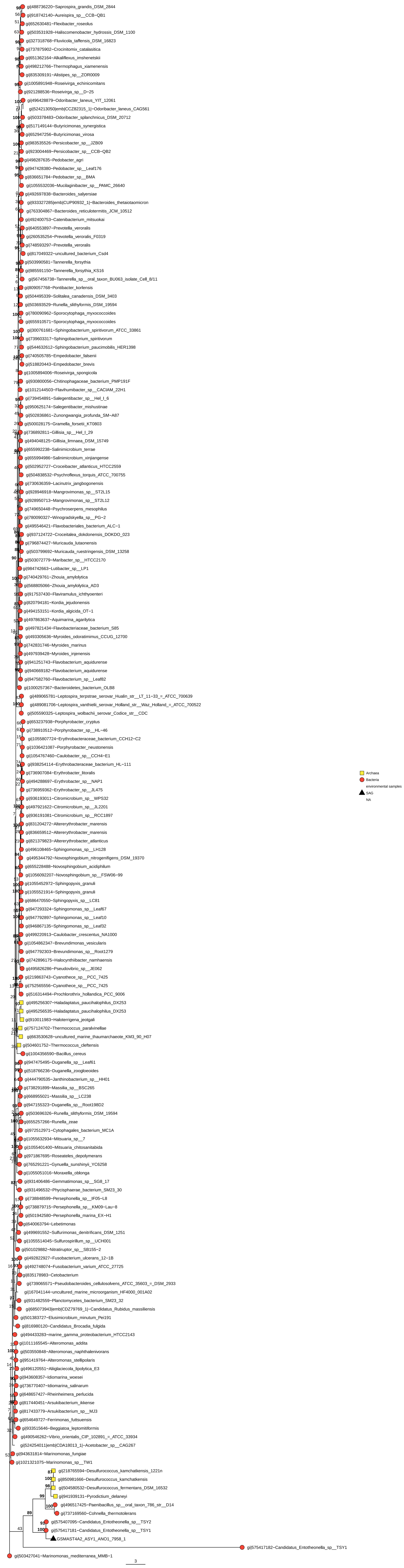

gi|488736220~Saprospira_grandis_DSM_2844
gi|918742140~Aureispira_sp__CCB~QB1
gi|652630481~Flexibacter_roseolus
gi|503531928~Haliscomenobacter_hydrossis_DSM_1100
gi|327318768~Fluviicola_taffensis_DSM_16823
gi|737875902~Crocinitomix_catalasitica
gi|651362164~Alkaliflexus_imshenetskii
gi|498212766~Thermophagus_xiamenensis
gi|835309191~Alistipes_sp__ZOR0009
gi|1005891948~Roseivirga_echinicomitans
gi|921288536~Roseivirga_sp__D~25
gi|496428879~Odoribacter_laneus_YIT_12061
gi|524213050|emb|CCZ82315_1|~Odoribacter_laneus_CAG561
gi|503378483~Odoribacter_splanchnicus_DSM_20712
gi|517149144~Butyricimonas_synergistica
gi|652947256~Butyricimonas_virosa
gi|983535526~Persicobacter_sp__JZB09
gi|923004469~Persicobacter_sp__CCB~QB2
gi|498287635~Pedobacter_agri
gi|947428380~Pedobacter_sp__Leaf176
gi|836651784~Pedobacter_sp__BMA
gi|1055532036~Mucilaginibacter_sp__PAMC_26640
gi|492697838~Bacteroides_salyersiae
gi|933327285|emb|CUP90932_1|~Bacteroides_thetaiotaomicron
gi|763304867~Bacteroides_reticulotermitis_JCM_10512
gi|492400753~Catenibacterium_mitsuokai
gi|640553897~Prevotella_veroralis
gi|260535254~Prevotella_veroralis_F0319
gi|748592397~Prevotella_veroralis
gi|81049322~uncultured_bacterium_Csd4
gi|503990581~Tannerella_forsythia
gi|985591150~Tannerella_forsythia_KS16
gi|567456738~Tannerella_sp__oral_taxon_BU063_isolate_Cell_8/11
gi|809057768~Pontibacter_korlensis
gi|504495339~Solitalea_canadensis_DSM_3403
gi|503693529~Runella_slithyformis_DSM_19594
gi|780090962~Sporocytophaga_myxococcoides
gi|655910571~Sporocytophaga_myxococcoides
gi|300761681~Sphingobacterium_spiritivorum_ATCC_33861
gi|739603317~Sphingobacterium_spiritivorum
gi|544632612~Sphingobacterium_paucimobilis_HER1398
gi|740505785~Empedobacter_falsenii
gi|518820443~Empedobacter_brevis
gi|1005894006~Roseivirga_spongicola
gi|930800056~Chitinophagaceae_bacterium_PMP191F
gi|1012144503~Flavihumibacter_sp__CACIAM_22H1
gi|739454891~Salegentibacter_sp__Hel_I_6
gi|950625174~Salegentibacter_mishustinae
gi|502836861~Zunongwangia_profunda_SM~A87
gi|500028175~Gramella_forsetii_KT0803
gi|736892811~Gillisia_sp__Hel_I_29
gi|494048125~Gillisia_limnaea_DSM_15749
gi|655992238~Salinimicrobium_terrae
gi|655994986~Salinimicrobium_xinjiangense
gi|502952727~Croceibacter_atlanticus_HTCC2559
gi|504838532~Psychroflexus_torquis_ATCC_700755
gi|730636359~Lacinutrix_jangbogonensis
gi|928946918~Mangrovimonas_sp__ST2L15
gi|928950713~Mangrovimonas_sp__ST2L12
gi|749650448~Psychroserpens_mesophilus
gi|780090327~Winogradskyella_sp__PG~2
gi|495546421~Flavobacteriales_bacterium_ALC~1
gi|937124722~Croceitalea_dokdonensis_DOKDO_023
gi|796874427~Muricauda_lutaonensis
gi|503799692~Muricauda_ruestringensis_DSM_13258
gi|503072779~Maribacter_sp__HTCC2170
gi|984742663~Lutibacter_sp__LP1
gi|740429761~Zhouia_amylolytica
gi|568805066~Zhouia_amylolytica_AD3
gi|917537430~Flaviramulus_ichthyoenteri
gi|820794181~Kordia_jejudonensis
gi|494153151~Kordia_algicida_OT~1
gi|497863637~Aquimarina_agarilytica
gi|497821434~Flavobacteriaceae_bacterium_S85
gi|493305636~Myroides_odoratimimus_CCUG_12700
gi|742831746~Myroides_marinus
gi|497939428~Myroides_injenensis
gi|941251743~Flavobacterium_aquidurense
gi|940669182~Flavobacterium_aquidurense
gi|947582760~Flavobacterium_sp__Leaf82
gi|1000257367~Bacteroidetes_bacterium_OLB8
gi|489065781~Leptospira_terpstrae_serovar_Hualin_str__LT_11~33_=_ATCC_700639
gi|489081706~Leptospira_vanthielii_serovar_Holland_str__Waz_Holland_=_ATCC_700522
gi|505590325~Leptospira_wolbachii_serovar_Codice_str__CDC
gi|653237938~Porphyrobacter_cryptus
gi|738910512~Porphyrobacter_sp__HL~46
gi|1055807724~Erythrobacteraceae_bacterium_CCH12~C2
gi|1036421087~Porphyrobacter_neustonensis
gi|1054767460~Caulobacter_sp__CCH4~E1
gi|938254114~Erythrobacteraceae_bacterium_HL~111
gi|736907084~Erythrobacter_litoralis
gi|494288697~Erythrobacter_sp__NAP1
gi|736959362~Erythrobacter_sp__JL475
gi|936193011~Citromicrobium_sp__WPS32
gi|497921622~Citromicrobium_sp__JL2201
gi|936191081~Citromicrobium_sp__RCC1897
gi|831204272~Altererythrobacter_marensis
gi|836659512~Altererythrobacter_marensis
gi|821379823~Altererythrobacter_atlanticus
gi|496108465~Sphingomonas_sp__LH128
gi|495344792~Novosphingobium_nitrogenifigens_DSM_19370
gi|655228488~Novosphingobium_acidiphilum
gi|1056092207~Novosphingobium_sp__FSW06~99
gi|1055452972~Sphingopyxis_granuli
gi|1055521914~Sphingopyxis_granuli
gi|686470550~Sphingopyxis_sp__LC81
gi|947293324~Sphingomonas_sp__Leaf67
gi|947792897~Sphingomonas_sp__Leaf10
gi|946867135~Sphingomonas_sp__Leaf32
gi|499220913~Caulobacter_crescentus_NA1000
gi|1054862347~Brevundimonas_vesicularis
gi|947792303~Brevundimonas_sp__Root1279
gi|742896175~Halocynthiibacter_namhaensis
gi|495826286~Pseudovibrio_sp__JE062
gi|219863743~Cyanothece_sp__PCC_7425
gi|752565556~Cyanothece_sp__PCC_7425
gi|516314494~Prochlorothrix_hollandica_PCC_9006
gi|495256307~Haladaptatus_paucihalophilus_DX253
gi|495256535~Haladaptatus_paucihalophilus_DX253
gi|910011983~Haloterrigena_jeotgali
gi|757124702~Thermococcus_paralvinellae
gi|663530628~uncultured_marine_thaumarchaeote_KM3_90_H07
gi|504601752~Thermococcus_cleftensis
gi|1004356590~Bacillus_cereus
gi|947475495~Duganella_sp__Leaf61
gi|518766236~Duganella_zoogloeoides
gi|444790535~Janthinobacterium_sp__HH01
gi|738291899~Massilia_sp__BSC265
gi|668955021~Massilia_sp__LC238
gi|947155323~Duganella_sp__Root198D2
gi|503696326~Runella_slithyformis_DSM_19594
gi|655257266~Runella_zeae
gi|972512971~Cytophagales_bacterium_MC1A
gi|1055632934~Mitsuaria_sp__7
gi|1055401400~Mitsuaria_chitosanitabida
gi|971867695~Roseateles_depolymerans
gi|765291221~Gynuella_sunshinyii_YC6258
gi|1055051016~Moraxella_oblonga
gi|931406486~Gemmatimonas_sp__SG8_17
gi|931496532~Phycisphaerae_bacterium_SM23_30
gi|738848599~Persephonella_sp__IF05~L8
gi|738879715~Persephonella_sp__KM09~Lau~8
gi|501942580~Persephonella_marina_EX~H1
gi|640063794~Lebetimonas
gi|499691552~Sulfurimonas_denitrificans_DSM_1251
gi|1055514045~Sulfurospirillum_sp__UCH001
gi|501029882~Nitratiruptor_sp__SB155~2
gi|492822927~Fusobacterium_ulcerans_12~1B
gi|492748074~Fusobacterium_varium_ATCC_27725
gi|835178983~Cetobacterium
gi|739065571~Pseudobacteroides_cellulosolvens_ATCC_35603_=_DSM_2933
gi|167041144~uncultured_marine_microorganism_HF4000_001A02
gi|931482559~Planctomycetes_bacterium_SM23_32
gi|685073943|emb|CDZ79769_1|~Candidatus_Rubidus_massiliensis
gi|501383727~Elusimicrobium_minutum_Pei191
gi|816980120~Candidatus_Brocadia_fulgida
gi|494433283~marine_gamma_proteobacterium_HTCC2143
gi|1011165545~Alteromonas_addita
gi|503550848~Alteromonas_naphthalenivorans
gi|951419764~Alteromonas_stellipolaris
gi|496120551~Aliiglaciecola_lipolytica_E3
gi|943608357~Idiomarina_woesei
gi|736770407~Idiomarina_salinarum
gi|648657427~Rheinheimera_perlucida
gi|817440451~Arsukibacterium_ikkense
gi|817433779~Arsukibacterium_sp__MJ3
gi|654649727~Ferrimonas_futtsuensis
gi|933515646~Beggiatoa_leptomitiformis
gi|490546262~Vibrio_orientalis_CIP_102891_=_ATCC_33934
gi|524254011|emb|CDA18013_1|~Acetobacter_sp__CAG267
gi|943631814~Marinomonas_fungiae
gi|1021321075~Marinomonas_sp__TW1
gi|218765594~Desulfurococcus_kamchatkensis_1221n
gi|850981666~Desulfurococcus_kamchatkensis
gi|504580532~Desulfurococcus_fermentans_DSM_16532
gi|941939131~Pyrodictium_delaneyi
gi|496517425~Paenibacillus_sp__oral_taxon_786_str__D14
gi|737169560~Cohnella_thermotolerans
gi|575407095~Candidatus_Entotheonella_sp__TSY2
gi|575417181~Candidatus_Entotheonella_sp__TSY1
GSMAST4A2_ASY1_ANO1_7958_1
gi|575417182~Candidatus_Entotheonella_sp__TSY1
gi|503427041~Marinomonas_mediterranea_MMB~1
3
Archaea
Bacteria
environmental samples
SAG
NA

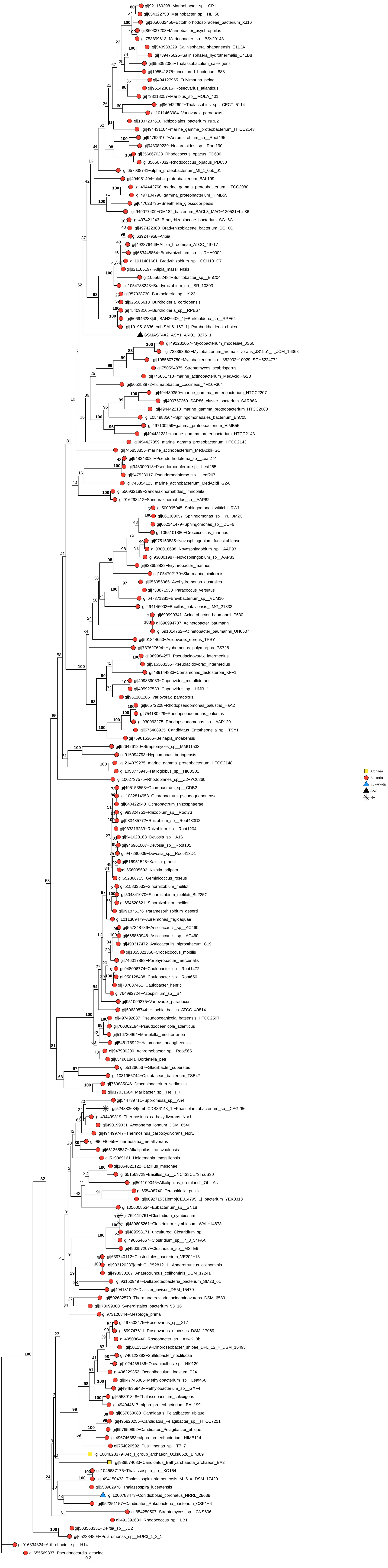
Archaea
Bacteria
Eukaryota
SAG
NA
0.2

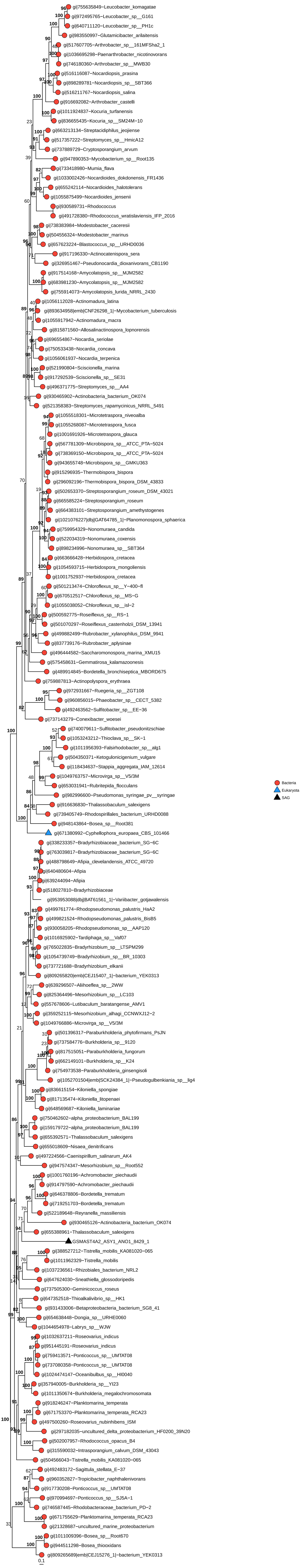

gi|755635849~Leucobacter_komagatae
gi|972495765~Leucobacter_sp__G161
gi|640711120~Leucobacter_sp__PH1c
gi|983550997~Glutamicibacter_arilaitensis
gi|517607705~Arthrobacter_sp__161MFSha2_1
gi|1036695298~Paenarthrobacter_nicotinovorans
gi|746180360~Arthrobacter_sp__MWB30
gi|516116087~Nocardiopsis_prasina
gi|898289781~Nocardiopsis_sp__SBT366
gi|516211767~Nocardiopsis_salina
gi|916692082~Arthrobacter_castelli
gi|1011924837~Kocuria_turfanensis
gi|836655435~Kocuria_sp_SM24M~10
gi|663213134~Streptacidiphilus_jeojiense
gi|517357222~Streptomyces_sp__HmicA12
gi|737889729~Cryptosporangium_arvum
gi|947890353~Mycobacterium_sp__Root135
gi|733418980~Mumia_flava
gi|1033002426~Nocardioides_dokdonensis_FR1436
gi|655242114~Nocardioides_halotolerans
gi|1055875499~Nocardioides_jensenii
gi|930589731~Rhodococcus
gi|491728380~Rhodococcus_wratislaviensis_IFP_2016
gi|738383984~Modestobacter_caceresii
gi|504556324~Modestobacter_marinus
gi|657623224~Blastococcus_sp__URHD0036
gi|917196330~Actinocatenispora_sera
gi|326951467~Pseudonocardia_dioxanivorans_CB1190
gi|917514168~Amycolatopsis_sp__MJM2582
gi|683981230~Amycolatopsis_sp__MJM2582
gi|755914073~Amycolatopsis_lurida_NRRL_2430
gi|1056112028~Actinomadura_latina
gi|893634958|emb|CNF26298_1|~Mycobacterium_tuberculosis
gi|1055917942~Actinomadura_macra
gi|815871560~Allosalinactinospora_lopnorensis
gi|696554867~Nocardia_seriolae
gi|750533438~Nocardia_concava
gi|1056061937~Nocardia_terpenica
gi|521990804~Sciscionella_marina
gi|917292539~Sciscionella_sp__SE31
gi|496371775~Streptomyces_sp__AA4
gi|930465902~Actinobacteria_bacterium_OK074
gi|521358383~Streptomyces_rapamycinicus_NRRL_5491
gi|1055518301~Microtetraspora_niveoalba
gi|1055268087~Microtetraspora_fusca
gi|1001691926~Microtetraspora_glauca
gi|567781309~Microbispora_sp__ATCC_PTA~5024
gi|738369150~Microbispora_sp__ATCC_PTA~5024
gi|943655748~Microbispora_sp__GMKU363
gi|915296935~Thermobispora_bispora
gi|296092196~Thermobispora_bispora_DSM_43833
gi|502653370~Streptosporangium_roseum_DSM_43021
gi|665585224~Streptosporangium_roseum
gi|664383101~Streptosporangium_amethystogenes
gi|1021076227|dbj|GAT64785_1|~Planomonospora_sphaerica
gi|759954329~Nonomuraea_candida
gi|522034319~Nonomuraea_coxensis
gi|898234996~Nonomuraea_sp__SBT364
gi|663666428~Herbidospora_cretacea
gi|1054593715~Herbidospora_mongoliensis
gi|1001752937~Herbidospora_cretacea
gi|501213474~Chloroflexus_sp__Y~400~fl
gi|670512517~Chloroflexus_sp__MS~G
gi|1055038052~Chloroflexus_sp__isl~2
gi|500592775~Roseiflexus_sp__RS~1
gi|501070297~Roseiflexus_castenholzii_DSM_13941
gi|499882499~Rubrobacter_xylanophilus_DSM_9941
gi|837739176~Rubrobacter_aplysinae
gi|496444582~Saccharomonospora_marina_XMU15
gi|575458631~Gemmatirosa_kalamazoonesis
gi|489914845~Bordetella_bronchiseptica_MBORD675
gi|759887813~Actinopolyspora_erythraea
gi|972931667~Ruegeria_sp__ZGT108
gi|960856015~Phaeobacter_sp__CECT_5382
gi|492463562~Sulfitobacter_sp__EE~36
gi|737143279~Conexibacter_woesei
gi|740079611~Sulfitobacter_pseudonitzschiae
gi|1053243212~Thioclava_sp__SK~1
gi|1011956393~Falsirhodobacter_sp__alg1
gi|504350371~Ketogulonicigenium_vulgare
gi|118434637~Stappia_aggregata_IAM_12614
gi|1049763757~Microvirga_sp__V5/3M
gi|653031941~Rubritepida_flocculans
gi|982996600~Pseudomonas_syringae_pv__syringae
gi|916636830~Thalassobaculum_salexigens
gi|739405749~Rhodospirillales_bacterium_URHD0088
gi|948143864~Bosea_sp__Root381
gi|671380992~Cyphellophora_europaea_CBS_101466
gi|338233357~Bradyrhizobiaceae_bacterium_SG~6C
gi|763039817~Bradyrhizobiaceae_bacterium_SG~6C
gi|488798649~Afipia_clevelandensis_ATCC_49720
gi|640480604~Afipia
gi|639244094~Afipia
gi|518027810~Bradyrhizobiaceae
gi|953953088|dbj|BAT61561_1|~Variibacter_gotjawalensis
gi|497761774~Rhodopseudomonas_palustris_HaA2
gi|499821524~Rhodopseudomonas_palustris_BisB5
gi|930058205~Rhodopseudomonas_sp__AAP120
gi|1016925902~Tardiphaga_sp__Vaf07
gi|765022835~Bradyrhizobium_sp__LTSPM299
gi|1054739749~Bradyrhizobium_sp__BR_10303
gi|737721688~Bradyrhizobium_elkanii
gi|809265820|emb|CEJ15407_1|~bacterium_YEK0313
gi|639296507~Aliihoeflea_sp__2WW
gi|825364496~Mesorhizobium_sp__LC103
gi|557678606~Lutibaculum_baratangense_AMV1
gi|359252115~Mesorhizobium_alhagi_CCNWXJ12~2
gi|1049766886~Microvirga_sp__V5/3M
gi|501396317~Paraburkholderia_phytofirmans_PsJN
gi|737584776~Burkholderia_sp__9120
gi|817515051~Paraburkholderia_fungorum
gi|662149101~Burkholderia_sp__K24
gi|754973538~Paraburkholderia_ginsengisoli
gi|1052701504|emb|SCK24384_1|~Pseudogulbenkiania_sp__lig4
gi|836615154~Kiloniella_spongiae
gi|817135474~Kiloniella_litopenaei
gi|648569687~Kiloniella_laminariae
gi|750462602~alpha_proteobacterium_BAL199
gi|159179722~alpha_proteobacterium_BAL199
gi|655392571~Thalassobaculum_salexigens
gi|655018609~Nisaea_denitrificans
gi|497224566~Caenispirillum_salinarum_AK4
gi|947574347~Mesorhizobium_sp__Root552
gi|1001760196~Achromobacter_piechaudii
gi|914797590~Achromobacter_piechaudii
gi|646378806~Bordetella_trematum
gi|719251703~Bordetella_trematum
gi|522189648~Reyranella_massiliensis
gi|930465126~Actinobacteria_bacterium_OK074
gi|655388961~Thalassobaculum_salexigens
GSMAST4A2_ASY1_ANO1_8429_1
gi|388527212~Tistrella_mobilis_KA081020~065
gi|1011962329~Tistrella_mobilis
gi|1037236561~Rhizobiales_bacterium_NRL2
gi|647624030~Sneathiella_glossodoripedis
gi|737505300~Geminicoccus_roseus
gi|647352518~Thioalkalivibrio_sp__HK1
gi|931433006~Betaproteobacteria_bacterium_SG8_41
gi|654638448~Dongia_sp__URHE0060
gi|1044654978~Labrys_sp__WJW
gi|1032637211~Roseovarius_indicus
gi|951445191~Roseovarius_indicus
gi|759413571~Ponticoccus_sp__UMTAT08
gi|737080358~Ponticoccus_sp__UMTAT08
gi|1024474147~Oceanibulbus_sp__HI0040
gi|357940005~Burkholderia_sp__YI23
gi|1011350674~Burkholderia_megalochromosomata
gi|918246247~Planktomarina_temperata
gi|671753370~Planktomarina_temperata_RCA23
gi|497500260~Roseovarius_nubinhibens_ISM
gi|297182035~uncultured_delta_proteobacterium_HF0200_39N20
gi|502007957~Rhodococcus_opacus_B4
gi|315590032~Intrasporangium_calvum_DSM_43043
gi|504566043~Tistrella_mobilis_KA081020~065
gi|492483172~Sagittula_stellata_E~37
gi|960352827~Tropicibacter_naphthalenivorans
gi|917730208~Ponticoccus_sp__UMTAT08
gi|970994697~Ponticoccus_sp__SJ5A~1
gi|746587445~Rhodobacteraceae_bacterium_PD~2
gi|671755629~Planktomarina_temperata_RCA23
gi|21328687~uncultured_marine_proteobacterium
gi|1011009396~Bosea_sp__Root670
gi|944511298~Bosea_thiooxidans
gi|809265689|emb|CEJ15276_1|~bacterium_YEK0313
0.1
Bacteria
Eukaryota
SAG

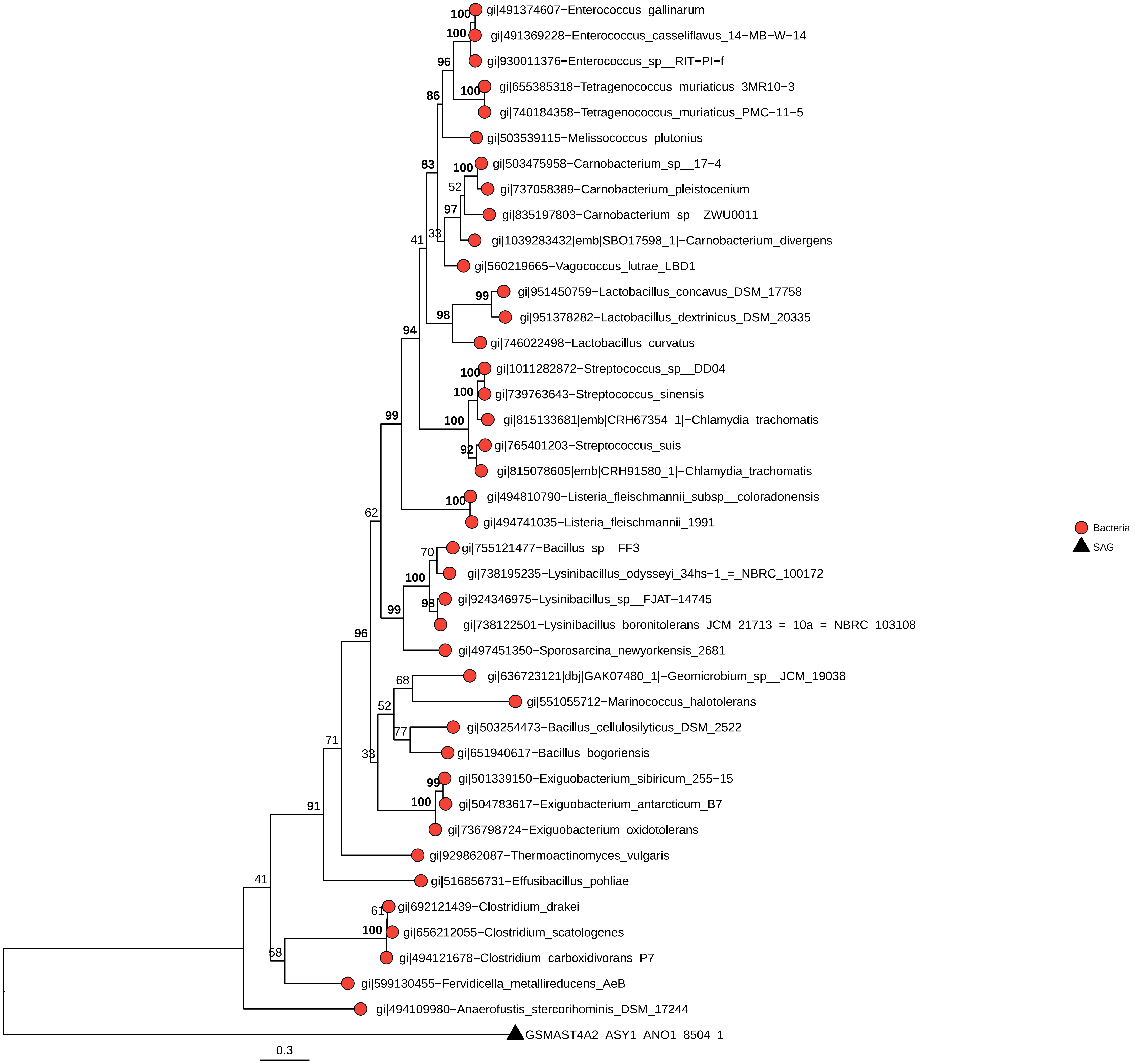
gi|491374607−Enterococcus_gallinarum
gi|491369228−Enterococcus_casseliflavus_14−MB−W−14
gi|930011376−Enterococcus_sp__RIT−PI−f
gi|655385318−Tetragenococcus_muriaticus_3MR10−3
gi|740184358−Tetragenococcus_muriaticus_PMC−11−5
gi|503539115−Melissococcus_plutonius
gi|503475958−Carnobacterium_sp__17−4
gi|737058389−Carnobacterium_pleistocenium
gi|835197803−Carnobacterium_sp__ZWU0011
gi|1039283432|emb|SBO17598_1|−Carnobacterium_divergens
gi|560219665−Vagococcus_lutrae_LBD1
gi|951450759−Lactobacillus_concavus_DSM_17758
gi|951378282−Lactobacillus_dextrinicus_DSM_20335
gi|746022498−Lactobacillus_curvatus
gi|1011282872−Streptococcus_sp__DD04
gi|739763643−Streptococcus_sinensis
gi|815133681|emb|CRH67354_1|−Chlamydia_trachomatis
gi|765401203−Streptococcus_suis
gi|815078605|emb|CRH91580_1|−Chlamydia_trachomatis
gi|494810790−Listeria_fleischmannii_subsp__coloradonensis
gi|494741035−Listeria_fleischmannii_1991
gi|755121477−Bacillus_sp__FF3
gi|738195235−Lysinibacillus_odysseyi_34hs−1_=_NBRC_100172
gi|924346975−Lysinibacillus_sp__FJAT−14745
gi|738122501−Lysinibacillus_boronitolerans_JCM_21713_=_10a_=_NBRC_103108
gi|497451350−Sporosarcina_newyorkensis_2681
gi|636723121|dbj|GAK07480_1|−Geomicrobium_sp__JCM_19038
gi|551055712−Marinococcus_halotolerans
gi|503254473−Bacillus_cellulosilyticus_DSM_2522
gi|651940617−Bacillus_bogoriensis
gi|501339150−Exiguobacterium_sibiricum_255−15
gi|504783617−Exiguobacterium_antarcticum_B7
gi|736798724−Exiguobacterium_oxidotolerans
gi|929862087−Thermoactinomyces_vulgaris
gi|516856731−Effusibacillus_pohliae
gi|692121439−Clostridium_drakei
gi|656212055−Clostridium_scatologenes
gi|494121678−Clostridium_carboxidivorans_P7
gi|599130455−Fervidicella_metallireducens_AeB
gi|494109980−Anaerofustis_stercorihominis_DSM_17244
GSMAST4A2_ASY1_ANO1_8504_1
Bacteria
SAG
0.3

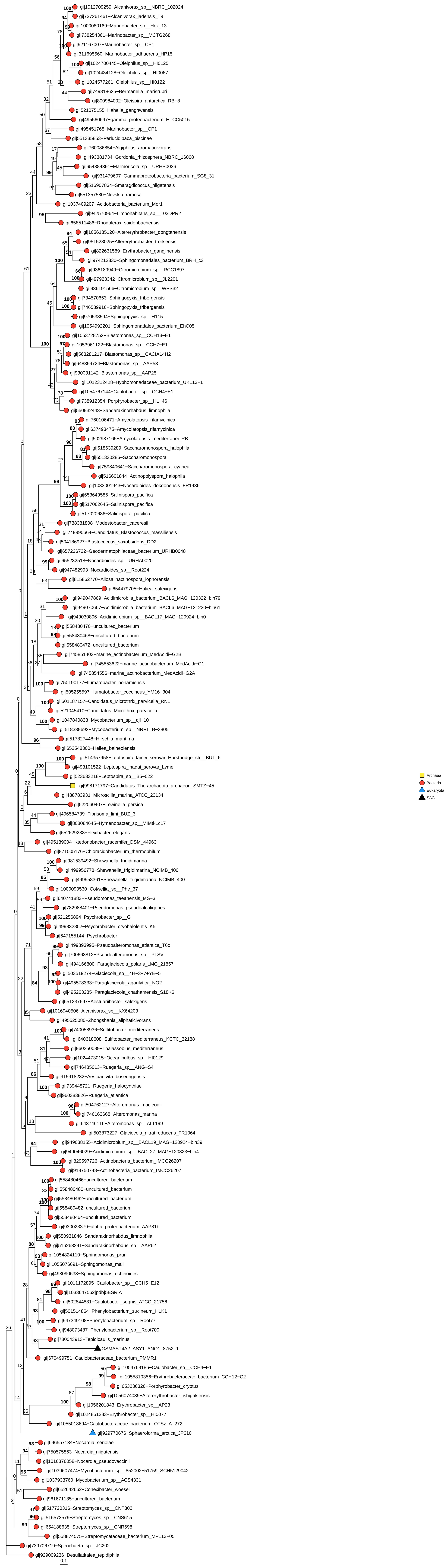
gi|1012709259~Alcanivorax_sp__NBRC_102024
gi|737261461~Alcanivorax_jadensis_T9
gi|1000080169~Marinobacter_sp__Hex_13
gi|738254361~Marinobacter_sp__MCTG268
gi|921167007~Marinobacter_sp__CP1
gi|311695560~Marinobacter_adhaerens_HP15
gi|1024700445~Oleiphilus_sp__HI0125
gi|1024434128~Oleiphilus_sp__HI0067
gi|1024577261~Oleiphilus_sp__HI0122
gi|749818625~Bermanella_marisrubri
gi|800984060~Oleispira_antarctica_RB~8
gi|521075155~Hahella_ganghwensis
gi|495560697~gamma_proteobacterium_HTCC5015
gi|495451768~Marinobacter_sp__CP1
gi|551335853~Perlucidibaca_piscinae
gi|760086854~Algiphilus_aromaticivorans
gi|493381734~Gordonia_rhizosphera_NBRC_16068
gi|654384391~Marmoricola_sp__URHB0036
gi|931479607~Gammaproteobacteria_bacterium_SG8_31
gi|516907834~Smaragdicoccus_niigatensis
gi|551357580~Nevskia_ramosa
gi|1037409207~Acidobacteria_bacterium_Mor1
gi|942570964~Limnohabitans_sp__103DPR2
gi|658511486~Rhodoferax_saidenbachensis
gi|1056185120~Altererythrobacter_dongtanensis
gi|951528025~Altererythrobacter_troitsensis
gi|822631589~Erythrobacter_gangjinensis
gi|974212330~Sphingomonadales_bacterium_BRH_c3
gi|936189949~Citromicrobium_sp__RCC1897
gi|497923342~Citromicrobium_sp__JL2201
gi|936191566~Citromicrobium_sp__WPS32
gi|734570653~Sphingopyxis_fribergensis
gi|746539916~Sphingopyxis_fribergensis
gi|970533594~Sphingopyxis_sp__H115
gi|1054992201~Sphingomonadales_bacterium_EhC05
gi|1053728752~Blastomonas_sp__CCH13~E1
gi|1053961122~Blastomonas_sp__CCH7~E1
gi|563281217~Blastomonas_sp__CACIA14H2
gi|648399724~Blastomonas_sp__AAP53
gi|930031142~Blastomonas_sp__AAP25
gi|1012312428~Hyphomonadaceae_bacterium_UKL13~1
gi|1054767144~Caulobacter_sp__CCH4~E1
gi|738912354~Porphyrobacter_sp__HL~46
gi|550932443~Sandarakinorhabdus_limnophila
gi|760106471~Amycolatopsis_rifamycinica
gi|637493475~Amycolatopsis_rifamycinica
gi|502987165~Amycolatopsis_mediterranei_RB
gi|518639289~Saccharomonospora_halophila
gi|651330286~Saccharomonospora
gi|759840641~Saccharomonospora_cyanea
gi|516601844~Actinopolyspora_halophila
gi|1033001943~Nocardioides_dokdonensis_FR1436
gi|653649586~Salinispora_pacifica
gi|517062645~Salinispora_pacifica
gi|517020686~Salinispora_pacifica
gi|738381808~Modestobacter_caceresii
gi|749990664~Candidatus_Blastococcus_massiliensis
gi|504186927~Blastococcus_saxobsidens_DD2
gi|657226722~Geodermatophilaceae_bacterium_URHB0048
gi|655232518~Nocardioides_sp__URHA0020
gi|947482993~Nocardioides_sp__Root224
gi|815862770~Allosalinactinospora_lopnorensis
gi|654479705~Haliea_salexigens
gi|949047869~Acidimicrobiia_bacterium_BACL6_MAG~120322~bin79
gi|949070667~Acidimicrobiia_bacterium_BACL6_MAG~121220~bin61
gi|949030806~Acidimicrobium_sp__BACL17_MAG~120924~bin0
gi|558480470~uncultured_bacterium
gi|558480468~uncultured_bacterium
gi|558480472~uncultured_bacterium
gi|745851403~marine_actinobacterium_MedAcidi~G2B
gi|745853622~marine_actinobacterium_MedAcidi~G1
gi|745854556~marine_actinobacterium_MedAcidi~G2A
gi|750190177~Ilumatobacter_nonamiensis
gi|505255597~Ilumatobacter_coccineus_YM16~304
gi|501187157~Candidatus_Microthrix_parvicella_RN1
gi|521045410~Candidatus_Microthrix_parvicella
gi|1047840838~Mycobacterium_sp__djl~10
gi|518339692~Mycobacterium_sp__NRRL_B~3805
gi|517827448~Hirschia_maritima
gi|652548300~Hellea_balneolensis
gi|514357958~Leptospira_fainei_serovar_Hurstbridge_str__BUT_6
gi|498101522~Leptospira_inadai_serovar_Lyme
gi|523633218~Leptospira_sp__B5~022
gi|998171797~Candidatus_Thorarchaeota_archaeon_SMTZ~45
gi|488783931~Microscilla_marina_ATCC_23134
gi|522060407~Lewinella_persica
gi|496584739~Fibrisoma_limi_BUZ_3
gi|808084645~Hymenobacter_sp__MIMtkLc17
gi|652629238~Flexibacter_elegans
gi|495189004~Ktedonobacter_racemifer_DSM_44963
gi|971005176~Chloracidobacterium_thermophilum
gi|981539492~Shewanella_frigidimarina
gi|499956778~Shewanella_frigidimarina_NCIMB_400
gi|499958361~Shewanella_frigidimarina_NCIMB_400
gi|1000090530~Colwellia_sp__Phe_37
gi|640741883~Pseudomonas_taeanensis_MS~3
gi|782988401~Pseudomonas_pseudoalcaligenes
gi|521256894~Psychrobacter_sp__G
gi|499832852~Psychrobacter_cryohalolentis_K5
gi|647155144~Psychrobacter
gi|499893995~Pseudoalteromonas_atlantica_T6c
gi|700668812~Pseudoalteromonas_sp__PLSV
gi|494166800~Paraglaciecola_polaris_LMG_21857
gi|503519274~Glaciecola_sp__4H~3~7+YE~5
gi|495578333~Paraglaciecola_agarilytica_NO2
gi|495263285~Paraglaciecola_chathamensis_S18K6
gi|651237697~Aestuariibacter_salexigens
gi|1016940506~Alcanivorax_sp__KX64203
gi|495525080~Zhongshania_aliphaticivorans
gi|740058936~Sulfitobacter_mediterraneus
gi|640618608~Sulfitobacter_mediterraneus_KCTC_32188
gi|960350089~Thalassobius_mediterraneus
gi|1024473015~Oceanibulbus_sp__HI0129
gi|746485013~Ruegeria_sp__ANG~S4
gi|915918232~Aestuariivita_boseongensis
gi|739448721~Ruegeria_halocynthiae
gi|960383826~Ruegeria_atlantica
gi|504762127~Alteromonas_macleodii
gi|746163668~Alteromonas_marina
gi|643746116~Alteromonas_sp__ALT199
gi|503873227~Glaciecola_nitratireducens_FR1064
gi|949038155~Acidimicrobium_sp__BACL19_MAG~120924~bin39
gi|949046029~Acidimicrobium_sp__BACL27_MAG~120823~bin4
gi|829597726~Actinobacteria_bacterium_IMCC26207
gi|918750748~Actinobacteria_bacterium_IMCC26207
gi|558480466~uncultured_bacterium
gi|558480480~uncultured_bacterium
gi|558480462~uncultured_bacterium
gi|558480482~uncultured_bacterium
gi|558480464~uncultured_bacterium
gi|930023379~alpha_proteobacterium_AAP81b
gi|550931846~Sandarakinorhabdus_limnophila
gi|516263241~Sandarakinorhabdus_sp__AAP62
gi|1054824110~Sphingomonas_pruni
gi|1055076691~Sphingomonas_mali
gi|498090633~Sphingomonas_echinoides
gi|1011172895~Caulobacter_sp__CCH5~E12
gi|1033647562|pdb|5ESR|A
gi|502844831~Caulobacter_segnis_ATCC_21756
gi|501514864~Phenylobacterium_zucineum_HLK1
gi|947349108~Phenylobacterium_sp__Root77
gi|948073487~Phenylobacterium_sp__Root700
gi|780043913~Tepidicaulis_marinus
GSMAST4A2_ASY1_ANO1_8752_1
gi|670499751~Caulobacteraceae_bacterium_PMMR1
gi|1054769186~Caulobacter_sp__CCH4~E1
gi|1055810356~Erythrobacteraceae_bacterium_CCH12~C2
gi|653236326~Porphyrobacter_cryptus
gi|1056074039~Altererythrobacter_ishigakiensis
gi|1056201843~Erythrobacter_sp__AP23
gi|1024851283~Erythrobacter_sp__HI0077
gi|1055018694~Caulobacteraceae_bacterium_OTSz_A_272
gi|929770676~Sphaeroforma_arctica_JP610
gi|696557134~Nocardia_seriolae
gi|750575863~Nocardia_niigatensis
gi|1016376058~Nocardia_pseudovaccinii
gi|1039607474~Mycobacterium_sp__852002~51759_SCH5129042
gi|1037933760~Mycobacterium_sp__ACS4331
gi|652642662~Conexibacter_woesei
gi|961671135~uncultured_bacterium
gi|517720316~Streptomyces_sp__CNT302
gi|516573579~Streptomyces_sp__CNS615
gi|654188635~Streptomyces_sp__CNR698
gi|558874575~Streptomycetaceae_bacterium_MP113~05
gi|739706719~Spirochaeta_sp__JC202
gi|929009236~Desulfatitalea_tepidiphila
0.1
Archaea
Bacteria
Eukaryota
SAG

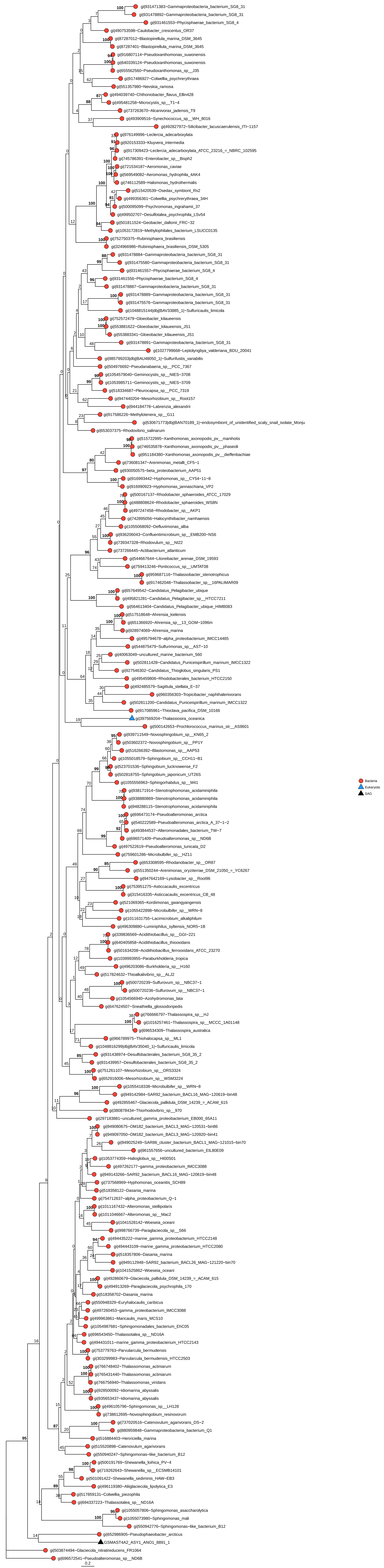

Bacteria
Eukaryota
SAG
0.2

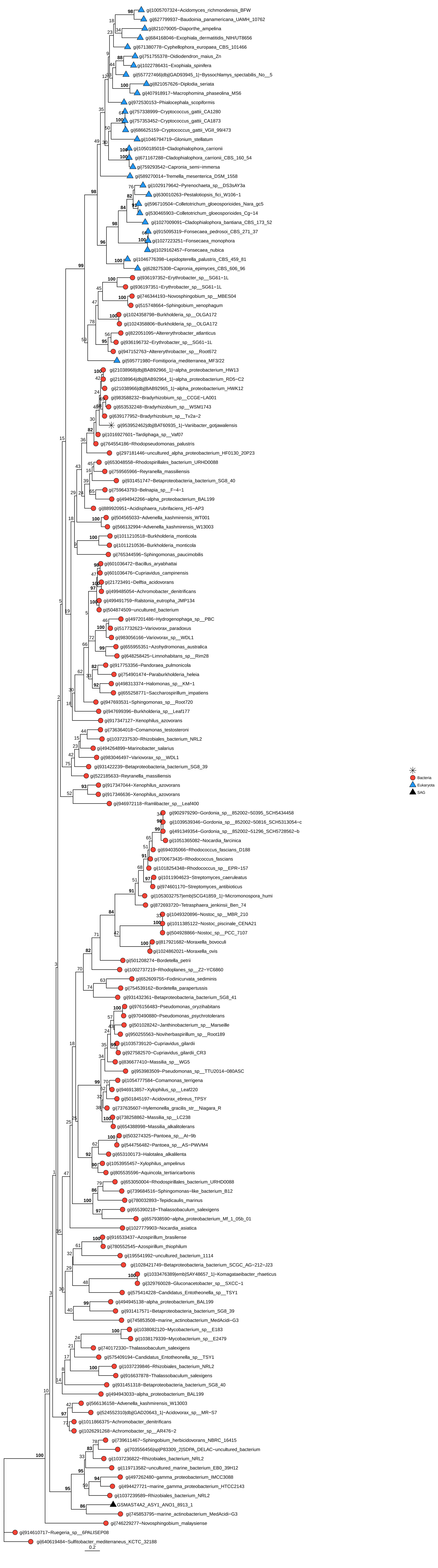

Bacteria
Eukaryota
SAG
0.2

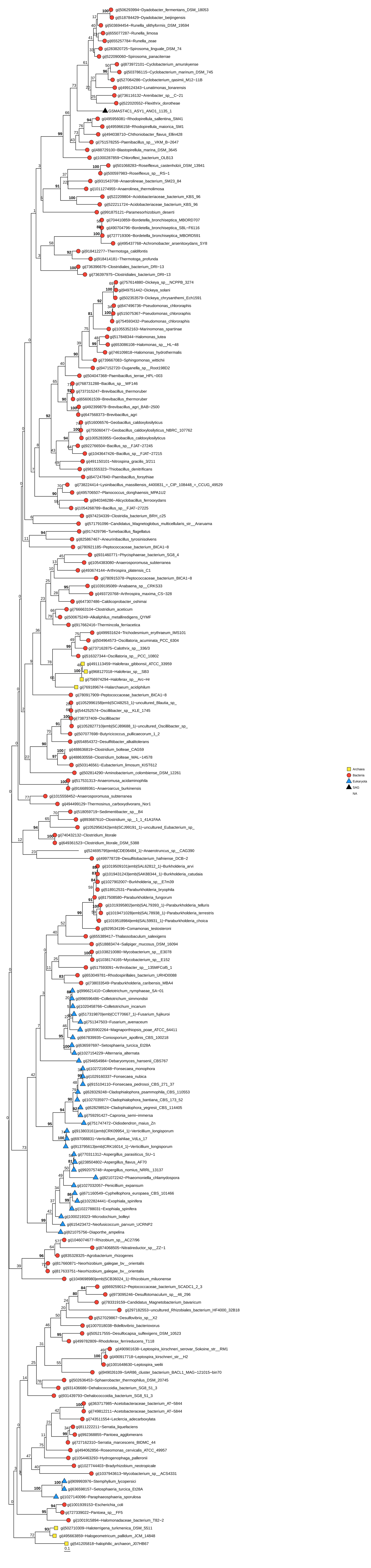
Archaea
Bacteria
Eukaryota
SAG
NA
0.1

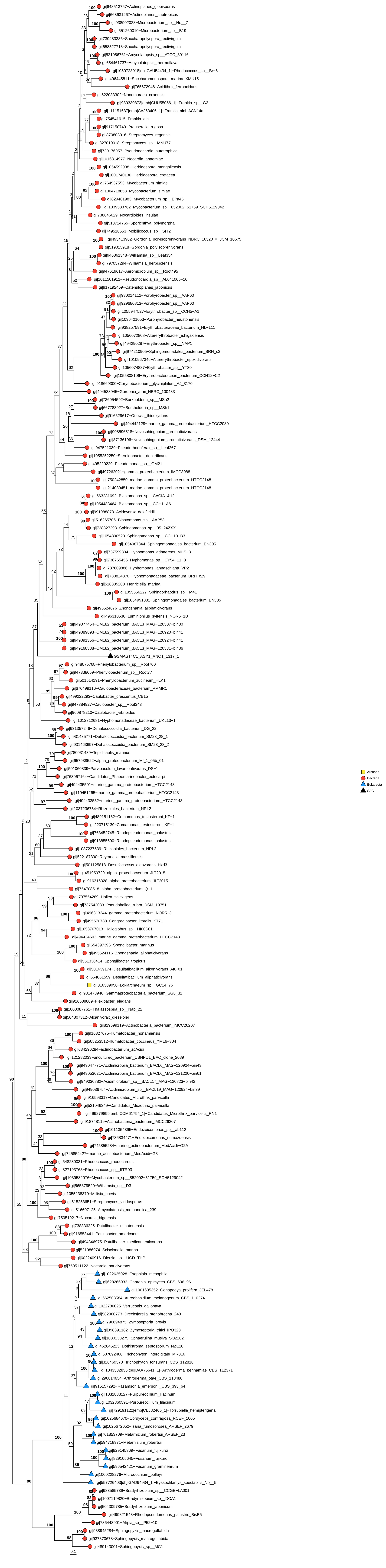

Archaea
Bacteria
Eukaryota
SAG
0.1

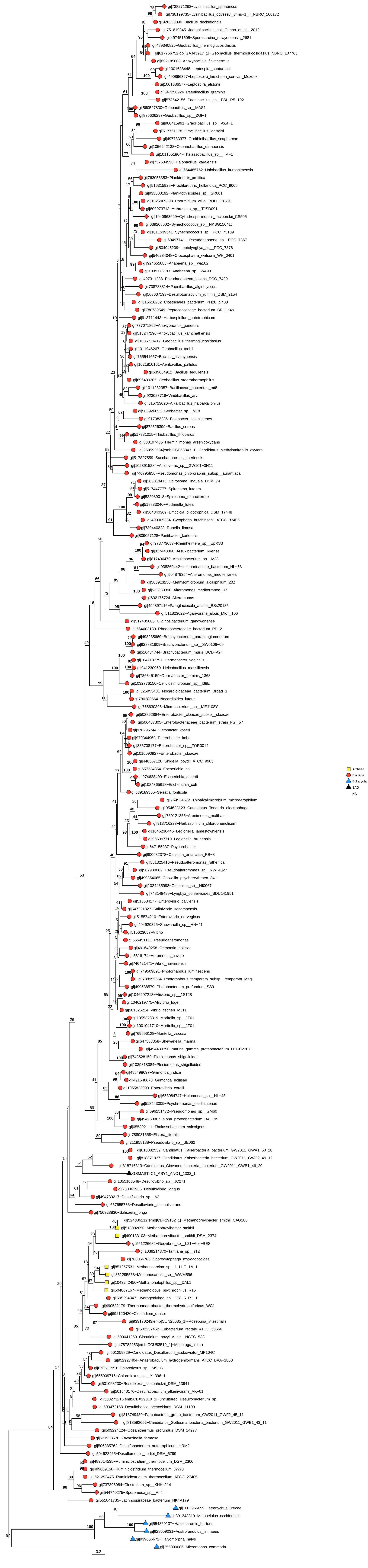
Archaea
Bacteria
Eukaryota
SAG
NA
gi|738271263~Lysinibacillus_sphaericus
gi|738199735~Lysinibacillus_odysseyi_34hs-1_=_NBRC_100172
gi|926258090~Bacillus_decisifrondis
gi|751619345~Jeotgalibacillus_soli_Cunha_et_al._2012
gi|497451605~Sporosarcina_newyorkensis_2681
gi|489340825~Geobacillus_thermoglucosidasius
gi|617766752|dbj|GAJ43917_1|~Geobacillus_thermoglucosidasius_NBRC_107763
gi|692185008~Anoxybacillus_flavithermus
gi|1001638448~Leptospira_santarosai
gi|490896327~Leptospira_kirschneri_serovar_Mozdok
gi|1001686577~Leptospira_alstonii
gi|647258924~Paenibacillus_graminis
gi|573542156~Paenibacillus_sp__FSL_R5-192
gi|560527830~Geobacillus_sp__MAS1
gi|836606297~Geobacillus_sp__ZGt-1
gi|960415991~Gracilibacillus_sp__Awa-1
gi|517761178~Gracilibacillus_lacisalsi
gi|497783377~Ornithinibacillus_scapharcae
gi|1056242139~Oceanobacillus_damuensis
gi|1011551864~Thalassobacillus_sp__TM-1
gi|737534556~Halobacillus_karajensis
gi|654485752~Halobacillus_kuroshimensis
gi|763056353~Planktothrix_prolifica
gi|516315929~Prochlorothrix_hollandica_PCC_9006
gi|935600192~Planktothricoides_sp__SR001
gi|1025909393~Phormidium_willei_BDU_130791
gi|809073713~Arthrospira_sp__TJSD091
gi|1040963629~Cylindrospermopsis_raciborskii_CS505
gi|639208602~Synechococcus_sp__NKBG15041c
gi|1011539341~Synechococcus_sp__PCC_73109
gi|504977411~Pseudanabaena_sp__PCC_7367
gi|504945209~Leptolyngbya_sp__PCC_7376
gi|546234048~Crocosphaera_watsonii_WH_0401
gi|924655083~Anabaena_sp__wa102
gi|1039176183~Anabaena_sp__WA93
gi|497311288~Pseudanabaena_biceps_PCC_7429
gi|738738814~Paenibacillus_alginolyticus
gi|503607193~Desulfotomaculum_ruminis_DSM_2154
gi|816616232~Clostridiales_bacterium_PH28_bin88
gi|780789549~Peptococcaceae_bacterium_BRH_c4a
gi|913711443~Herbaspirillum_autotrophicum
gi|737071866~Anoxybacillus_gonensis
gi|518247290~Anoxybacillus_kamchatkensis
gi|1035711417~Geobacillus_thermoglucosidasius
gi|1011946267~Geobacillus_toebii
gi|765541657~Bacillus_alveayuensis
gi|1021810101~Aeribacillus_pallidus
gi|639654912~Bacillus_tequilensis
gi|696489305~Geobacillus_stearothermophilus
gi|1011282357~Bacillaceae_bacterium_mt8
gi|923023718~Viridibacillus_arvi
gi|515753020~Alkalibacillus_haloalkaliphilus
gi|505926055~Geobacter_sp__M18
gi|917083286~Pelobacter_seleniigenes
gi|872526399~Bacillus_cereus
gi|517331015~Thiobacillus_thioparus
gi|500197435~Herminiimonas_arsenicoxydans
gi|258592534|emb|CBE68843_1|~Candidatus_Methylomirabilis_oxyfera
gi|517807559~Saccharibacillus_kuerlensis
gi|1023915284~Acidovorax_sp__GW101-3H11
gi|740795856~Pseudomonas_chlororaphis_subsp__aurantiaca
gi|283818415~Spirosoma_linguale_DSM_74
gi|517447777~Spirosoma_luteum
gi|522089018~Spirosoma_panaciterrae
gi|518833046~Rudanella_lutea
gi|504840369~Emticicia_oligotrophica_DSM_17448
gi|499905384~Cytophaga_hutchinsonii_ATCC_33406
gi|739440323~Runella_limosa
gi|809057128~Pontibacter_korlensis
gi|973773037~Rheinheimera_sp__EpRS3
gi|817440860~Arsukibacterium_ikkense
gi|817436470~Arsukibacterium_sp__MJ3
gi|938269442~Idiomarinaceae_bacterium_HL-53
gi|504879354~Alteromonas_mediterranea
gi|503913250~Methylomicrobium_alcaliphilum_20Z
gi|522830398~Alteromonas_mediterranea_U7
gi|692175724~Alteromonas
gi|494897116~Paraglaciecola_arctica_BSs20135
gi|511823622~Agarivorans_albus_MKT_106
gi|517435685~Uliginosibacterium_gangwonense
gi|564603180~Rhodobacteraceae_bacterium_PD-2
gi|498235669~Brachybacterium_paraconglomeratum
gi|928881609~Brachybacterium_sp__SW0106-09
gi|516434744~Brachybacterium_muris_UCD-AY4
gi|1042187797~Dermabacter_vaginalis
gi|941230960~Helcobacillus_massiliensis
gi|736345159~Dermabacter_hominis_1368
gi|1032776150~Cellulosimicrobium_sp__I38E
gi|325953401~Nocardioidaceae_bacterium_Broad-1
gi|780288564~Nocardioides_luteus
gi|755630396~Microbacterium_sp__MEJ108Y
gi|502862884~Enterobacter_cloacae_subsp__cloacae
gi|506487305~Enterobacteriaceae_bacterium_strain_FGI_57
gi|970295744~Citrobacter_koseri
gi|970344969~Enterobacter_kobei
gi|835708177~Enterobacter_sp__ZOR0014
gi|1016090927~Enterobacter_cloacae
gi|446567128~Shigella_boydii_ATCC_9905
gi|657334354~Escherichia_coli
gi|974628409~Escherichia_albertii
gi|1024365618~Escherichia_coli
gi|639189355~Serratia_fonticola
gi|764534672~Thioalkalimicrobium_microaerophilum
gi|954628123~Candidatus_Tenderia_electrophaga
gi|760121355~Arenimonas_malthae
gi|913716223~Herbaspirillum_chlorophenolicum
gi|1046230446~Legionella_jamestowniensis
gi|966397710~Legionella_brunensis
gi|647155937~Psychrobacter
gi|800982378~Oleispira_antarctica_RB-8
gi|551325410~Pseudoalteromonas_ruthenica
gi|567930062~Pseudoalteromonas_sp__NW_4327
gi|499354065~Colwellia_psychrerythraea_34H
gi|1024435998~Oleiphilus_sp__HI0067
gi|748148499~Lyngbya_confervoides_BDU141951
gi|515584177~Enterovibrio_calviensis
gi|647221827~Salinivibrio_socompensis
gi|515574210~Enterovibrio_norvegicus
gi|494920325~Shewanella_sp__HN-41
gi|515623057~Vibrio
gi|655451111~Pseudoalteromonas
gi|491649258~Grimontia_hollisae
gi|5616174~Aeromonas_caviae
gi|746421471~Vibrio_navarrensis
gi|749509891~Photorhabdus_luminescens
gi|738955564~Photorhabdus_temperata_subsp__temperata_Meg1
gi|499538579~Photobacterium_profundum_SS9
gi|1046207213~Aliivibrio_sp__1S128
gi|1046219775~Aliivibrio_logei
gi|501526214~Vibrio_fischeri_MJ11
gi|1055378319~Moritella_sp__JT01
gi|1001041710~Moritella_sp__JT01
gi|769996128~Moritella_viscosa
gi|647533358~Shewanella_marina
gi|494439390~marine_gamma_proteobacterium_HTCC2207
gi|743528150~Plesiomonas_shigelloides
gi|1039818084~Plesiomonas_shigelloides
gi|488498697~Grimontia_indica
gi|491648678~Grimontia_hollisae
gi|1055823009~Enterovibrio_coralii
gi|653084747~Halomonas_sp__HL-48
gi|518443005~Psychromonas_ossibalaenae
gi|696251472~Pseudomonas_sp__GM60
gi|494950967~alpha_proteobacterium_BAL199
gi|655392111~Thalassobaculum_salexigens
gi|778031558~Elstera_litoralis
gi|211958188~Pseudovibrio_sp__JE062
gi|818882539~Candidatus_Kaiserbacteria_bacterium_GW2011_GWA1_50_28
gi|818871937~Candidatus_Kaiserbacteria_bacterium_GW2011_GWC2_49_12
gi|818716313~Candidatus_Giovannonibacteria_bacterium_GW2011_GWB1_46_20
GSMAST4C1_ASY1_ANO1_1333_1
gi|1055108548~Desulfovibrio_sp__JC271
gi|750063965~Desulfovibrio_longus
gi|494789217~Desulfovibrio_sp__A2
gi|657655783~Desulfovibrio_alcoholivorans
gi|750323836~Salisaeta_longa
gi|524836212|emb|CDF29152_1|~Methanobrevibacter_smithii_CAG186
gi|518092650~Methanobrevibacter_smithii
gi|490133103~Methanobrevibacter_smithii_DSM_2374
gi|555122682~Geovibrio_sp__L21-Ace-BES
gi|1039214370~Tamlana_sp__s12
gi|780066765~Sporocytophaga_myxococcoides
gi|851257531~Methanosarcina_sp__1_H_T_1A_1
gi|851295568~Methanosarcina_sp__WWM596
gi|1043242450~Methanohalophilus_sp__DAL1
gi|504867167~Methanolobus_psychrophilus_R15
gi|695294347~Hydrogenivirga_sp__128-5-R1-1
gi|490532179~Thermoanaerobacter_thermohydrosulfuricus_WC1
gi|692120420~Clostridium_drakei
gi|933170243|emb|CUN28685_1|~Roseburia_intestinalis
gi|502257462~Eubacterium_rectale_ATCC_33656
gi|500041250~Clostridium_novyi_A_str__NCTC_538
gi|478782953|emb|CCU83510_1|~Mesotoga_infera
gi|501259829~Candidatus_Desulforudis_audaxviator_MP104C
gi|952927404~Anaerobaculum_hydrogeniformans_ATCC_BAA-1850
gi|670511951~Chloroflexus_sp__MS-G
gi|655009716~Chloroflexus_sp__Y-396-1
gi|501068230~Roseiflexus_castenholzii_DSM_13941
gi|501640176~Desulfatibacillum_alkenivorans_AK-01
gi|308273215|emb|CBX29818_1|~uncultured_Desulfobacterium_sp_
gi|503472168~Desulfobacca_acetoxidans_DSM_11109
gi|818749480~Parcubacteria_group_bacterium_GW2011_GWF2_45_11
gi|818592652~Candidatus_Gottesmanbacteria_bacterium_GW2011_GWB1_43_11
gi|503224124~Oceanithermus_profundus_DSM_14977
gi|521958576~Zavarzinella_formosa
gi|506385762~Desulfobacterium_autotrophicum_HRM2
gi|504622465~Desulfomonile_tiedjei_DSM_6799
gi|489614535~Ruminiclostridium_thermocellum_DSM_2360
gi|489609156~Ruminiclostridium_thermocellum_JW20
gi|521293475~Ruminiclostridium_thermocellum_ATCC_27405
gi|737306984~Clostridium_sp__KNHs214
gi|544740275~Sporomusa_sp__An4
gi|551041735~Lachnospiraceae_bacterium_NK4A179
gi|1005966699~Tetranychus_urticae
gi|391343819~Metaseiulus_occidentalis
gi|554869137~Haplochromis_burtoni
gi|928059031~Austrofundulus_limnaeus
gi|939656672~Halyomorpha_halys
gi|255090086~Micromonas_commoda
0.2

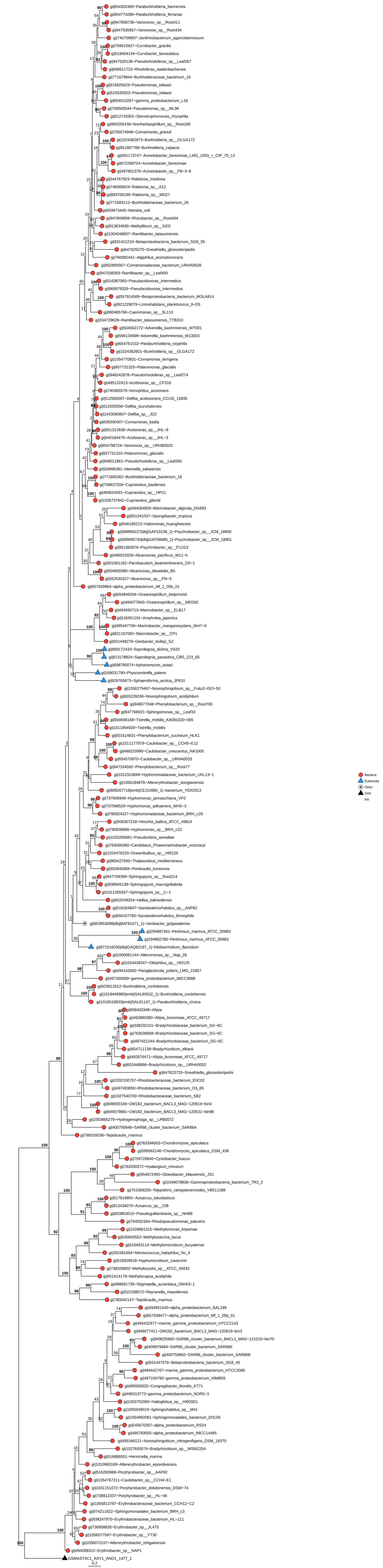
Bacteria
Eukaryota
Other
SAG
NA
0.2

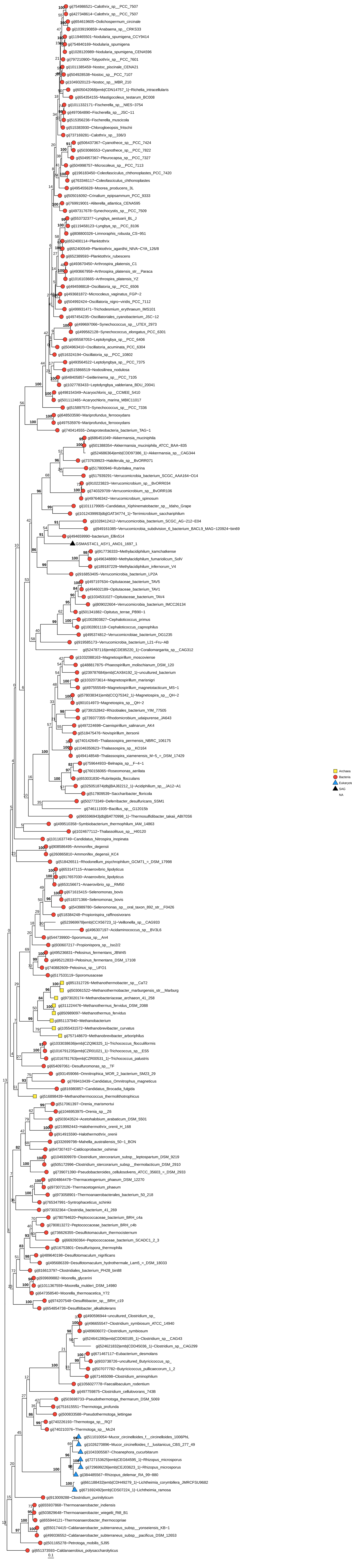

gi|754986521~Calothrix_sp__PCC_7507
gi|427348614~Calothrix_sp__PCC_7507
gi|654619605~Dolichospermum_circinale
gi|1039190859~Anabaena_sp__CRKS33
gi|119465501~Nodularia_spumigena_CCY9414
gi|754840169~Nodularia_spumigena
gi|1028120989~Nodularia_spumigena_CENA596
gi|797210900~Tolypothrix_sp__PCC_7601
gi|1011385459~Nostoc_piscinale_CENA21
gi|504928538~Nostoc_sp__PCC_7107
gi|1049320123~Nostoc_sp__MBR_210
gi|505042068|emb|CDN14757_1|~Richelia_intracellularis
gi|654354155~Mastigocoleus_testarum_BC008
gi|1011332171~Fischerella_sp__NIES~3754
gi|497064890~Fischerella_sp__JSC~11
gi|515356236~Fischerella_muscicola
gi|515383930~Chlorogloeopsis_fritschii
gi|737169281~Calothrix_sp__336/3
gi|506437367~Cyanothece_sp__PCC_7424
gi|503086553~Cyanothece_sp__PCC_7822
gi|504957367~Pleurocapsa_sp__PCC_7327
gi|504998757~Microcoleus_sp__PCC_7113
gi|916183450~Coleofasciculus_chthonoplastes_PCC_7420
gi|763346117~Coleofasciculus_chthonoplastes
gi|495455628~Moorea_producens_3L
gi|505016092~Crinalium_epipsammum_PCC_9333
gi|769919001~Alterella_atlantica_CENA595
gi|497317678~Synechocystis_sp__PCC_7509
gi|553732377~Lyngbya_aestuarii_BL_J
gi|119458123~Lyngbya_sp__PCC_8106
gi|808800326~Limnoraphis_robusta_CS~951
gi|652400114~Planktothrix
gi|652400549~Planktothrix_agardhii_NIVA~CYA_126/8
gi|652389593~Planktothrix_rubescens
gi|493670450~Arthrospira_platensis_C1
gi|493667958~Arthrospira_platensis_str__Paraca
gi|1016103665~Arthrospira_platensis_YZ
gi|494598818~Oscillatoria_sp__PCC_6506
gi|493681872~Microcoleus_vaginatus_FGP~2
gi|504992424~Oscillatoria_nigro~viridis_PCC_7112
gi|499931471~Trichodesmium_erythraeum_IMS101
gi|497454235~Oscillatoriales_cyanobacterium_JSC~12
gi|499697066~Synechococcus_sp__UTEX_2973
gi|499562128~Synechococcus_elongatus_PCC_6301
gi|495587053~Leptolyngbya_sp__PCC_6406
gi|504963410~Oscillatoria_acuminata_PCC_6304
gi|516324194~Oscillatoria_sp__PCC_10802
gi|495564522~Leptolyngbya_sp__PCC_7375
gi|515866519~Nodosilinea_nodulosa
gi|648405857~Geitlerinema_sp__PCC_7105
gi|1027783433~Leptolyngbya_valderiana_BDU_20041
gi|498154349~Acaryochloris_sp__CCMEE_5410
gi|501112465~Acaryochloris_marina_MBIC11017
gi|515897573~Synechococcus_sp__PCC_7336
gi|648503590~Mariprofundus_ferrooxydans
gi|497535976~Mariprofundus_ferrooxydans
gi|740414555~Zetaproteobacteria_bacterium_TAG~1
gi|586451049~Akkermansia_muciniphila
gi|501388354~Akkermansia_muciniphila_ATCC_BAA~835
gi|524686364|emb|CDD97386_1|~Akkermansia_sp__CAG344
gi|737639923~Haloferula_sp__BvORR071
gi|517800946~Rubritalea_marina
gi|517839291~Verrucomicrobia_bacterium_SCGC_AAA164~O14
gi|910223823~Verrucomicrobium_sp__BvORR034
gi|740329709~Verrucomicrobium_sp__BvORR106
gi|497646342~Verrucomicrobium_spinosum
gi|1011179905~Candidatus_Xiphinematobacter_sp__Idaho_Grape
gi|1012439993|dbj|GAT34774_1|~Terrimicrobium_sacchariphilum
gi|1028412412~Verrucomicrobia_bacterium_SCGC_AG~212~E04
gi|949161085~Verrucomicrobia_subdivision_6_bacterium_BACL9_MAG~120924~bin69
gi|494659990~bacterium_Ellin514
GSMAST4C1_ASY1_ANO1_1697_1
gi|917736333~Methylacidiphilum_kamchatkense
gi|496348890~Methylacidiphilum_fumariolicum_SolV
gi|189187229~Methylacidiphilum_infernorum_V4
gi|916853405~Verrucomicrobia_bacterium_LP2A
gi|497197634~Opitutaceae_bacterium_TAV5
gi|494602189~Opitutaceae_bacterium_TAV1
gi|1034531027~Opitutaceae_bacterium_TAV4
gi|809022604~Verrucomicrobia_bacterium_IMCC26134
gi|501341882~Opitutus_terrae_PB90~1
gi|1002803827~Cephaloticoccus_primus
gi|1002801118~Cephaloticoccus_capnophilus
gi|495374812~Verrucomicrobiae_bacterium_DG1235
gi|919585173~Verrucomicrobia_bacterium_L21~Fru~AB
gi|524787116|emb|CDE85220_1|~Coraliomargarita_sp__CAG312
gi|1032088163~Magnetospirillum_moscoviense
gi|488817875~Phaeospirillum_molischianum_DSM_120
gi|239787684|emb|CAX84192_1|~uncultured_bacterium
gi|1032073614~Magnetospirillum_marisnigri
gi|497555549~Magnetospirillum_magnetotacticum_MS~1
gi|578038341|emb|CCQ75342_1|~Magnetospira_sp__QH~2
gi|801014973~Magnetospira_sp__QH~2
gi|739152842~Rhizobiales_bacterium_YIM_77505
gi|739377355~Rhodomicrobium_udaipurense_JA643
gi|497224698~Caenispirillum_salinarum_AK4
gi|518475476~Novispirillum_itersonii
gi|740142645~Thalassospira_permensis_NBRC_106175
gi|1046350623~Thalassospira_sp__KO164
gi|494148548~Thalassospira_xiamenensis_M~5_=_DSM_17429
gi|759644933~Belnapia_sp__F~4~1
gi|760156065~Roseomonas_aerilata
gi|653031830~Rubritepida_flocculans
gi|325051874|dbj|BAJ82212_1|~Acidiphilium_sp__JA12~A1
gi|517809539~Saccharibacter_floricola
gi|502773349~Deferribacter_desulfuricans_SSM1
gi|746111935~Bacillus_sp__G1(2015)b
gi|965596943|dbj|BAT70998_1|~Thermosulfidibacter_takaii_ABI70S6
gi|499510358~Symbiobacterium_thermophilum_IAM_14863
gi|1024677112~Thalassolituus_sp__HI0120
gi|1011637749~Candidatus_Nitrospira_inopinata
gi|908586495~Ammonifex_degensii
gi|260865810~Ammonifex_degensii_KC4
gi|518426511~Rhodoferax_psychrophilum_GCM71_=_DSM_17998
gi|653147115~Anaerovibrio_lipolyticus
gi|917657030~Anaerovibrio_lipolyticus
gi|653156671~Anaerovibrio_sp__RM50
gi|671615415~Selenomonas_bovis
gi|518371366~Selenomonas_bovis
gi|543989780~Selenomonas_sp__oral_taxon_892_str__F0426
gi|518384248~Propionispira_raffinosivorans
gi|523969978|emb|CCZ56723_1|~Veillonella_sp__CAG933
gi|496307197~Acidaminococcus_sp__BV3L6
gi|544739900~Sporomusa_sp__An4
gi|930607217~Propionispora_sp__Iso2/2
gi|495236831~Pelosinus_fermentans_JBW45
gi|495212833~Pelosinus_fermentans_DSM_17108
gi|740882609~Pelosinus_sp__UFO1
gi|517533119~Sporomusaceae
gi|651312726~Methanothermobacter_sp__CaT2
gi|503061522~Methanothermobacter_marburgensis_str__Marburg
gi|973020174~Methanobacteriaceae_archaeon_41_258
gi|311224476~Methanothermus_fervidus_DSM_2088
gi|850989097~Methanothermus_fervidus
gi|851137940~Methanobacterium
gi|1055431572~Methanobrevibacter_curvatus
gi|757148670~Methanobrevibacter_arboriphilus
gi|1030836636|emb|CZQ96325_1|~Trichococcus_flocculiformis
gi|1016791235|emb|CZR01021_1|~Trichococcus_sp__ES5
gi|1016781763|emb|CZR00531_1|~Trichococcus_palustris
gi|654097061~Desulfuromonas_sp__TF
gi|931459006~Omnitrophica_WOR_2_bacterium_SM23_29
gi|769410439~Candidatus_Omnitrophus_magneticus
gi|816980857~Candidatus_Brocadia_fulgida
gi|516898439~Methanothermococcus_thermolithotrophicus
gi|517061397~Orenia_marismortui
gi|1046953975~Orenia_sp__Z6
gi|503043524~Acetohalobium_arabaticum_DSM_5501
gi|219992443~Halothermothrix_orenii_H_168
gi|914915590~Halothermothrix_orenii
gi|332699798~Mahella_australiensis_50~1_BON
gi|647307437~Caldicoprobacter_oshimai
gi|1049309978~Clostridium_stercorarium_subsp__leptospartum_DSM_9219
gi|505172996~Clostridium_stercorarium_subsp__thermolacticum_DSM_2910
gi|739071390~Pseudobacteroides_cellulosolvens_ATCC_35603_=_DSM_2933
gi|504864478~Thermacetogenium_phaeum_DSM_12270
gi|973072126~Thermacetogenium_phaeum
gi|973058901~Thermoanaerobacterales_bacterium_50_218
gi|765347991~Syntrophaceticus_schinkii
gi|973032364~Clostridia_bacterium_41_269
gi|780794620~Peptococcaceae_bacterium_BRH_c4a
gi|780813272~Peptococcaceae_bacterium_BRH_c4b
gi|736626355~Desulfotomaculum_thermocisternum
gi|669260364~Peptococcaceae_bacterium_SCADC1_2_3
gi|515753801~Desulfurispora_thermophila
gi|489640198~Desulfotomaculum_nigrificans
gi|495686339~Desulfotomaculum_hydrothermale_Lam5_=_DSM_18033
gi|816613797~Clostridiales_bacterium_PH28_bin88
gi|939699882~Moorella_glycerini
gi|1011367559~Moorella_mulderi_DSM_14980
gi|647358540~Moorella_thermoacetica_Y72
gi|974207548~Desulfitobacter_sp__BRH_c19
gi|654854738~Desulfitibacter_alkalitolerans
gi|490596944~uncultured_Clostridium_sp_
gi|496655547~Clostridium_symbiosum_ATCC_14940
gi|489606072~Clostridium_symbiosum
gi|524641280|emb|CDD60185_1|~Clostridium_sp__CAG43
gi|524621832|emb|CDD45036_1|~Clostridium_sp__CAG299
gi|671467117~Eubacterium_desmolans
gi|933738726~uncultured_Butyricicoccus_sp_
gi|507077782~Butyricicoccus_pullicaecorum_1_2
gi|671465098~Clostridium_aminophilum
gi|1056027778~Faecalibaculum_rodentium
gi|497759875~Clostridium_cellulovorans_743B
gi|503698733~Pseudothermotoga_thermarum_DSM_5069
gi|751615551~Thermotoga_profunda
gi|500633588~Pseudothermotoga_lettingae
gi|740226193~Thermotoga_sp__RQ7
gi|740210376~Thermotoga_sp__Mc24
gi|511010054~Mucor_circinelloides_f__circinelloides_1006PhL
gi|1026270896~Mucor_circinelloides_f__lusitanicus_CBS_277_49
gi|1043305587~Choanephora_cucurbitarum
gi|727153625|emb|CEG66595_1|~Rhizopus_microsporus
gi|729699226|emb|CEJ03623_1|~Rhizopus_microsporus
gi|384485567~Rhizopus_delemar_RA_99~880
gi|661188432|emb|CDH49279_1|~Lichtheimia_corymbifera_JMRC:FSU:9682
gi|671692492|emb|CDS07224_1|~Lichtheimia_ramosa
gi|913009288~Clostridium_purinilyticum
gi|655937868~Thermoanaerobacter_indiensis
gi|503829648~Thermoanaerobacter_wiegelii_Rt8_B1
gi|655944121~Thermoanaerobacter_thermocopriae
gi|550174415~Caldanaerobacter_subterraneus_subsp__yonseiensis_KB~1
gi|499336552~Caldanaerobacter_subterraneus_subsp__pacificus_DSM_12653
gi|501165278~Petrotoga_mobilis_SJ95
gi|651373593~Caldanaerobius_polysaccharolyticus
0.1
Archaea
Bacteria
Eukaryota
SAG
NA

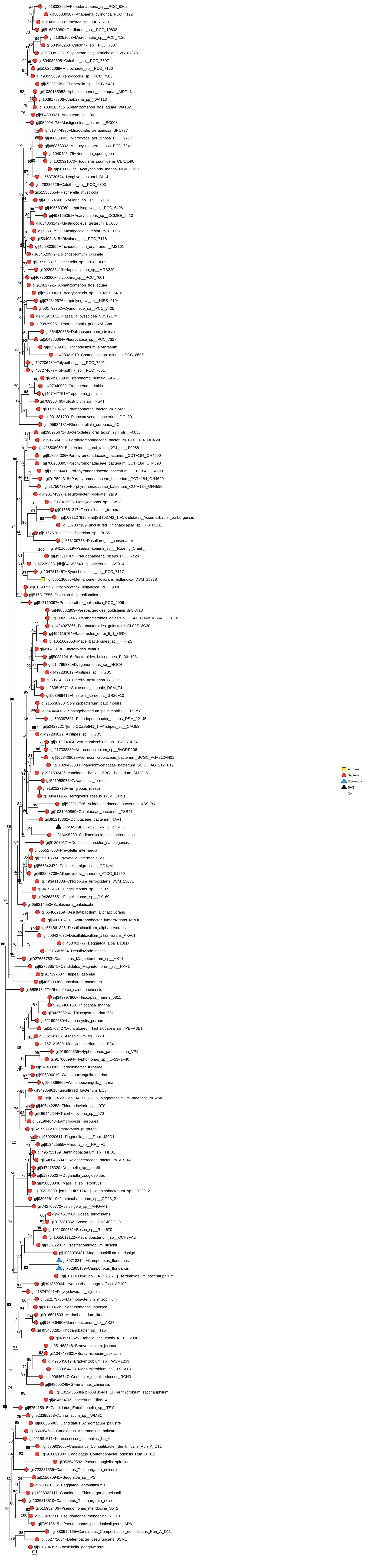

Archaea
Bacteria
Eukaryota
SAG
NA
gi|518328969~Pseudanabaena_sp__PCC_6802
gi|505030307~Anabaena_cylindrica_PCC_7122
gi|1049320837~Nostoc_sp__MBR_210
gi|516328980~Oscillatoria_sp__PCC_10802
gi|516251693~Microchaete_sp__PCC_7126
gi|504940263~Calothrix_sp__PCC_7507
gi|889981322~Scytonema_tolypothrichoides_VB-61278
gi|504939395~Calothrix_sp__PCC_7507
gi|516252069~Microchaete_sp__PCC_7126
gi|493555089~Xenococcus_sp__PCC_7305
gi|655321581~Fischerella_sp__PCC_9431
gi|1039185952~Aphanizomenon_flos-aquae_MDT14a
gi|1039178706~Anabaena_sp__WA113
gi|1039203415~Aphanizomenon_flos-aquae_WA102
gi|504890931~Anabaena_sp__90
gi|959924173~Mastigocoleus_testarum_BC008
gi|513474335~Microcystis_aeruginosa_SPC777
gi|488850402~Microcystis_aeruginosa_PCC_9717
gi|488863392~Microcystis_aeruginosa_PCC_7941
gi|1040495475~Nodularia_spumigena
gi|1020321070~Nodularia_spumigena_CENA596
gi|501117195~Acaryochloris_marina_MBIC11017
gi|553730574~Lyngbya_aestuarii_BL_J
gi|428235526~Calothrix_sp__PCC_6303
gi|515353554~Fischerella_muscicola
gi|427374508~Rivularia_sp__PCC_7116
gi|495583760~Leptolyngbya_sp__PCC_6406
gi|498165351~Acaryochloris_sp__CCMEE_5410
gi|654353142~Mastigocoleus_testarum_BC008
gi|738313556~Mastigocoleus_testarum_BC008
gi|504934815~Rivularia_sp__PCC_7116
gi|499930955~Trichodesmium_erythraeum_IMS101
gi|654620972~Dolichospermum_circinale
gi|737153377~Fischerella_sp__PCC_9605
gi|922988413~Hapalosiphon_sp__MRB220
gi|407266260~Tolypothrix_sp__PCC_7601
gi|916817225~Aphanizomenon_flos-aquae
gi|657199631~Acaryochloris_sp__CCMEE_5410
gi|972342976~Leptolyngbya_sp__NIES-2104
gi|501732352~Cyanothece_sp__PCC_7425
gi|746971638~Hassallia_byssoidea_VB512170
gi|938299251~Phormidesmis_priestleyi_Ana
gi|654620865~Dolichospermum_circinale
gi|504956064~Pleurocapsa_sp__PCC_7327
gi|825989312~Trichodesmium_erythraeum
gi|428021910~Chamaesiphon_minutus_PCC_6605
gi|797206430~Tolypothrix_sp__PCC_7601
gi|407274677~Tolypothrix_sp__PCC_7601
gi|505820949~Treponema_primitia_ZAS-2
gi|497940002~Treponema_primitia
gi|497947751~Treponema_primitia
gi|769360460~Clostridium_sp__FS41
gi|931504752~Phycisphaerae_bacterium_SM23_33
gi|931381703~Planctomycetes_bacterium_DG_20
gi|495934181~Rhodopirellula_europaea_6C
gi|298275671~Bacteroidetes_oral_taxon_274_str__F0058
gi|917504255~Porphyromonadaceae_bacterium_COT-184_OH4590
gi|496508950~Bacteroidetes_oral_taxon_274_str__F0058
gi|917504336~Porphyromonadaceae_bacterium_COT-184_OH4590
gi|700230395~Porphyromonadaceae_bacterium_COT-184_OH4590
gi|917504460~Porphyromonadaceae_bacterium_COT-184_OH4590
gi|917504318~Porphyromonadaceae_bacterium_COT-184_OH4590
gi|917504335~Porphyromonadaceae_bacterium_COT-184_OH4590
gi|490174327~Desulfobulbus_postgatei_2ac9
gi|917063525~Methylomonas_sp__LW13
gi|916651217~Teredinibacter_turnerae
gi|1037127915|emb|SBT03742_1|~Candidatus_Accumulibacter_aalborgensis
gi|557037109~uncultured_Thiohalocapsa_sp__PB-PSB1
gi|916767612~Desulfosarcina_sp__BuS5
gi|653109753~Desulforegula_conservatrix
gi|941165519~Pseudanabaena_sp___Roaring_Creek_
gi|497314469~Pseudanabaena_biceps_PCC_7429
gi|672393931|dbj|GAK53549_1|~bacterium_UASB14
gi|1047311457~Synechococcus_sp__PCC_7117
gi|505136685~Methanomethylovorans_hollandica_DSM_15978
gi|815937157~Prochlorothrix_hollandica_PCC_9006
gi|516317555~Prochlorothrix_hollandica
gi|817119397~Prochlorothrix_hollandica_PCC_9006
gi|498502803~Parabacteroides_goldsteinii_dnLKV18
gi|806522446~Parabacteroides_goldsteinii_DSM_19448_=_WAL_12034
gi|949271366~Parabacteroides_goldsteinii_CL02T12C30
gi|495115764~Bacteroides_dorei_5_1_36/D4
gi|1051602053~Macellibacteroides_sp__HH-ZS
gi|490430136~Bacteroides_ovatus
gi|503312416~Bacteroides_helcogenes_P_36-108
gi|914765832~Dysgonomonas_sp__HGC4
gi|497283619~Alistipes_sp__HGB5
gi|505142583~Fibrella_aestuarina_BUZ_2
gi|283816071~Spirosoma_linguale_DSM_74
gi|503989412~Niastella_koreensis_GR20-10
gi|916538980~Sphingobacterium_paucimobilis
gi|543404182~Sphingobacterium_paucimobilis_HER1398
gi|503397501~Pseudopedobacter_saltans_DSM_12145
gi|524232217|emb|CCZ95641_1|~Alistipes_sp__CAG53
gi|497283822~Alistipes_sp__HGB5
gi|910224964~Verrucomicrobium_sp__BvORR034
gi|917338889~Verrucomicrobium_sp__BvORR106
gi|1028429029~Verrucomicrobiaceae_bacterium_SCGC_AG-212-N21
gi|1028425684~Planctomycetaceae_bacterium_SCGC_AG-212-F19
gi|915324626~candidate_division_BRC1_bacterium_SM23_51
gi|521958978~Zavarzinella_formosa
gi|918022716~Terriglobus_roseus
gi|390411966~Terriglobus_roseus_DSM_18391
gi|522211726~Acidobacteriaceae_bacterium_KBS_96
gi|1031959869~Opitutaceae_bacterium_TSB47
gi|391218362~Opitutaceae_bacterium_TAV1
GSMAST4C1_ASY1_ANO1_2334_1
gi|916695239~Sedimenticola_selenatireducens
gi|918570171~Dethiosulfatarculus_sandiegensis
gi|655527353~Prevotella_intermedia
gi|771513684~Prevotella_intermedia_ZT
gi|565842472~Prevotella_nigrescens_CC14M
gi|493298708~Alloprevotella_tannerae_ATCC_51259
gi|493411303~Chlorobium_ferrooxidans_DSM_13031
gi|941934531~Flagellimonas_sp__DK169
gi|942687551~Flagellimonas_sp__DK169
gi|656314950~Schlesneria_paludicola
gi|654862169~Desulfatibacillum_aliphaticivorans
gi|500016710~Syntrophobacter_fumaroxidans_MPOB
gi|654861325~Desulfatibacillum_aliphaticivorans
gi|506427672~Desulfatibacillum_alkenivorans_AK-01
gi|488761777~Beggiatoa_alba_B18LD
gi|916687634~Desulfovibrio_bastinii
gi|927685783~Candidatus_Magnetomorum_sp__HK-1
gi|927688575~Candidatus_Magnetomorum_sp__HK-1
gi|917297687~Hippea_jasoniae
gi|406893363~uncultured_bacterium
gi|658513427~Rhodoferax_saidenbachensis
gi|343797860~Thiocapsa_marina_5811
gi|915460153~Thiocapsa_marina
gi|343798260~Thiocapsa_marina_5811
gi|521992630~Lamprocystis_purpurea
gi|557034275~uncultured_Thiohalocapsa_sp__PB-PSB1
gi|502743662~Azospirillum_sp__B510
gi|757121689~Methyloceanibacter_sp__B34
gi|916990635~Hyphomonas_jannaschiana_VP2
gi|917000994~Hyphomonas_sp__L-53-1-40
gi|518433905~Teredinibacter_turnerae
gi|906396019~Wenzhouxiangella_marina
gi|906866662~Wenzhouxiangella_marina
gi|349858618~uncultured_bacterium_EC5
gi|829456531|dbj|BAE50517_1|~Magnetospirillum_magneticum_AMB-1
gi|496442253~Thiorhodovibrio_sp__970
gi|496442244~Thiorhodovibrio_sp__970
gi|521994648~Lamprocystis_purpurea
gi|521997123~Lamprocystis_purpurea
gi|950220611~Duganella_sp__Root1480D1
gi|913425555~Massilia_sp__NR_4-1
gi|495723169~Janthinobacterium_sp__HH01
gi|648643604~Oxalobacteraceae_bacterium_AB_14
gi|947475320~Duganella_sp__Leaf61
gi|518765237~Duganella_zoogloeoides
gi|950030336~Massilia_sp__Root351
gi|955158091|emb|CUI09124_1|~Janthinobacterium_sp__CG23_2
gi|930616119~Janthinobacterium_sp__CG23_2
gi|742700770~Leisingera_sp__ANG-M1
gi|944510904~Bosea_thiooxidans
gi|917381362~Bosea_sp__UNC402CLCol
gi|1011009581~Bosea_sp__Root670
gi|1055612122~Methylobacterium_sp__CCH7-A2
gi|933872817~Prosthecomicrobium_hirschii
gi|1032076431~Magnetospirillum_marisnigri
gi|307188144~Camponotus_floridanus
gi|752865109~Camponotus_floridanus
gi|1012439045|dbj|GAT33826_1|~Terrimicrobium_sacchariphilum
gi|391859964~Hydrocarboniphaga_effusa_AP103
gi|918247901~Polycyclovorans_algicola
gi|522173735~Marinobacterium_rhizophilum
gi|916614699~Neptunomonas_japonica
gi|916691503~Marinobacterium_litorale
gi|917086490~Marinobacterium_sp__AK27
gi|495483281~Rhodanobacter_sp__115
gi|499719625~Hahella_chejuensis_KCTC_2396
gi|951463348~Bradyrhizobium_jicamae
gi|1047433601~Bradyrhizobium_paxllaeri
gi|497545019~Bradyrhizobium_sp__WSM1253
gi|639054458~Marinimicrobium_sp__LS-A18
gi|490646747~Geobacter_metallireducens_RCH3
gi|648595249~Gilvimarinus_chinensis
gi|1012436638|dbj|GAT35441_1|~Terrimicrobium_sacchariphilum
gi|494654769~bacterium_Ellin514
gi|575415915~Candidatus_Entotheonella_sp__TSY1
gi|923395253~Achromatium_sp__WMS1
gi|860366983~Candidatus_Achromatium_palustre
gi|860364617~Candidatus_Achromatium_palustre
gi|291581611~Nitrosococcus_halophilus_Nc_4
gi|880903605~Candidatus_Competibacter_denitrificans_Run_A_D11
gi|916891000~Candidatus_Contendobacter_odensis_Run_B_J11
gi|953549532~Pseudohongiella_spirulinae
gi|723287539~Candidatus_Thiomargarita_nelsonii
gi|152070941~Beggiatoa_sp__PS
gi|933516302~Beggiatoa_leptomitiformis
gi|1026322111~Candidatus_Thiomargarita_nelsonii
gi|1026315810~Candidatus_Thiomargarita_nelsonii
gi|515932406~Pseudomonas_mendocina_S5_2
gi|503482711~Pseudomonas_mendocina_NK-01
gi|739135151~Pseudomonas_pseudoalcaligenes_AD6
gi|880919165~Candidatus_Competibacter_denitrificans_Run_A_D11
gi|502772064~Deferribacter_desulfuricans_SSM1
gi|916704397~Zooshikella_ganghwensis
0.1

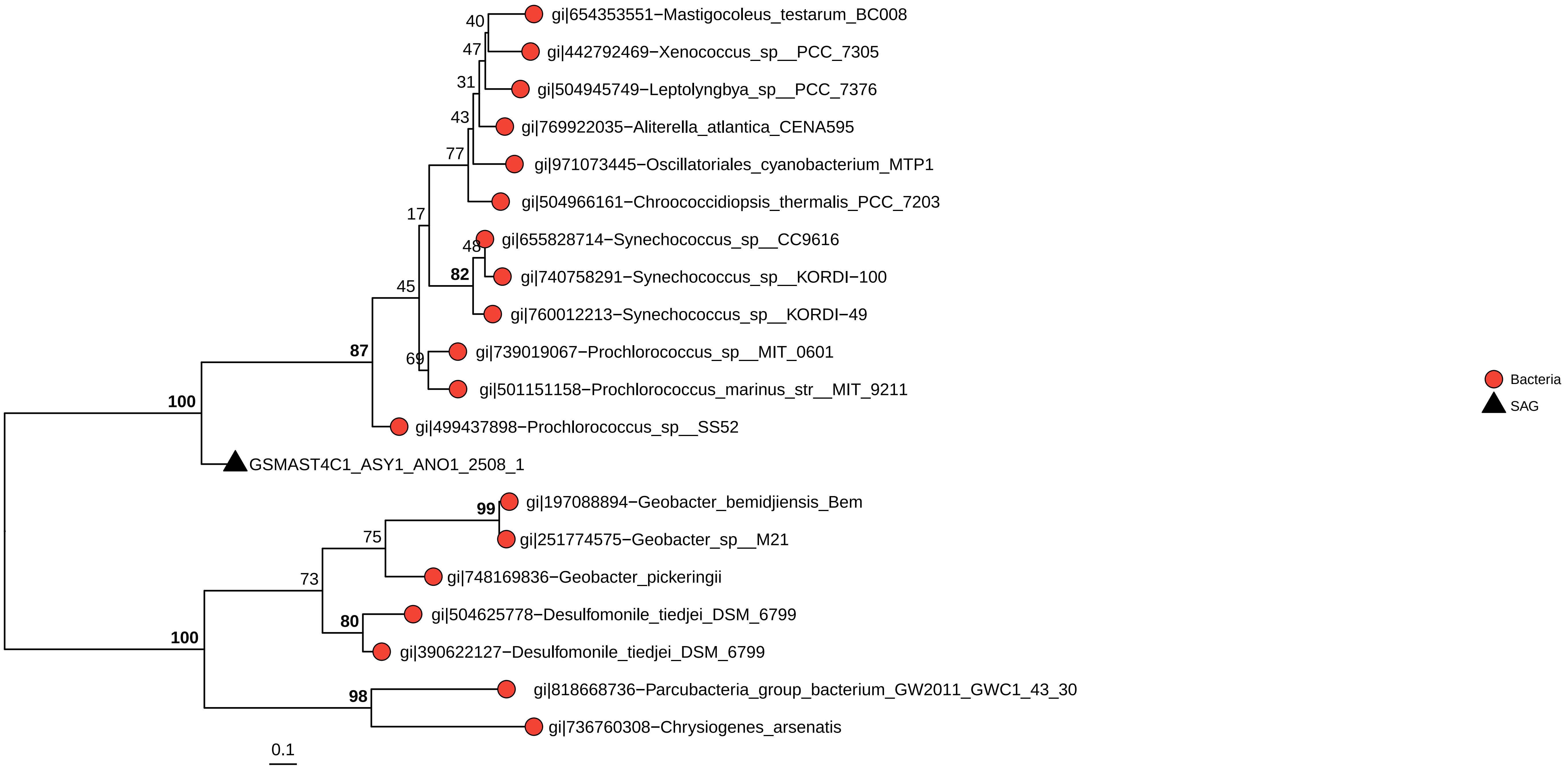

gi|654353551−Mastigocoleus_testarum_BC008
gi|442792469−Xenococcus_sp__PCC_7305
gi|504945749−Leptolyngbya_sp__PCC_7376
gi|769922035−Aliterella_atlantica_CENA595
gi|971073445−Oscillatoriales_cyanobacterium_MTP1
gi|504966161−Chroococcidiopsis_thermalis_PCC_7203
gi|655828714−Synechococcus_sp__CC9616
gi|740758291−Synechococcus_sp__KORDI−100
gi|760012213−Synechococcus_sp__KORDI−49
gi|739019067−Prochlorococcus_sp__MIT_0601
gi|501151158−Prochlorococcus_marinus_str__MIT_9211
gi|499437898−Prochlorococcus_sp__SS52
GSMAST4C1_ASY1_ANO1_2508_1
gi|197088894−Geobacter_bemidjiensis_Bem
gi|251774575−Geobacter_sp__M21
gi|748169836−Geobacter_pickeringii
gi|504625778−Desulfomonile_tiedjei_DSM_6799
gi|390622127−Desulfomonile_tiedjei_DSM_6799
gi|818668736−Parcubacteria_group_bacterium_GW2011_GWC1_43_30
gi|736760308−Chrysiogenes_arsenatis
40
47
31
43
77
17
48
82
45
69
87
100
99
75
73
80
100
98
0.1
Bacteria
SAG

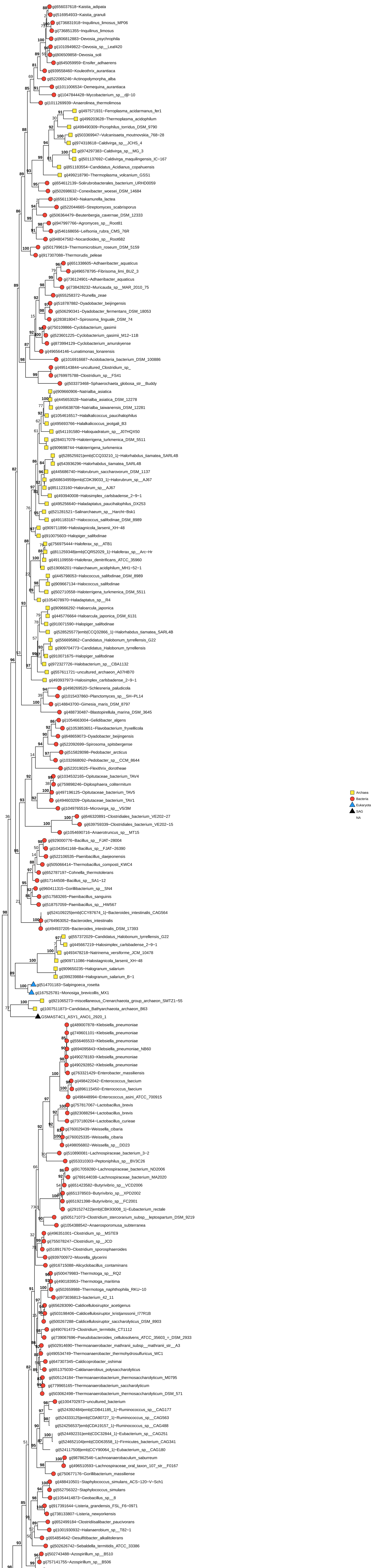
Archaea
Bacteria
Eukaryota
SAG
NA
0.1

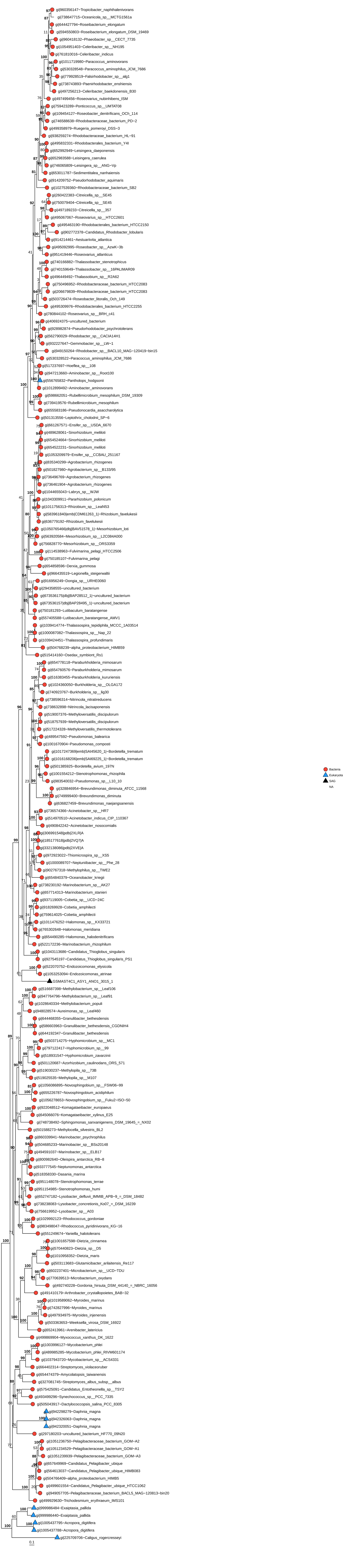
Bacteria
Eukaryota
SAG
NA
0.1

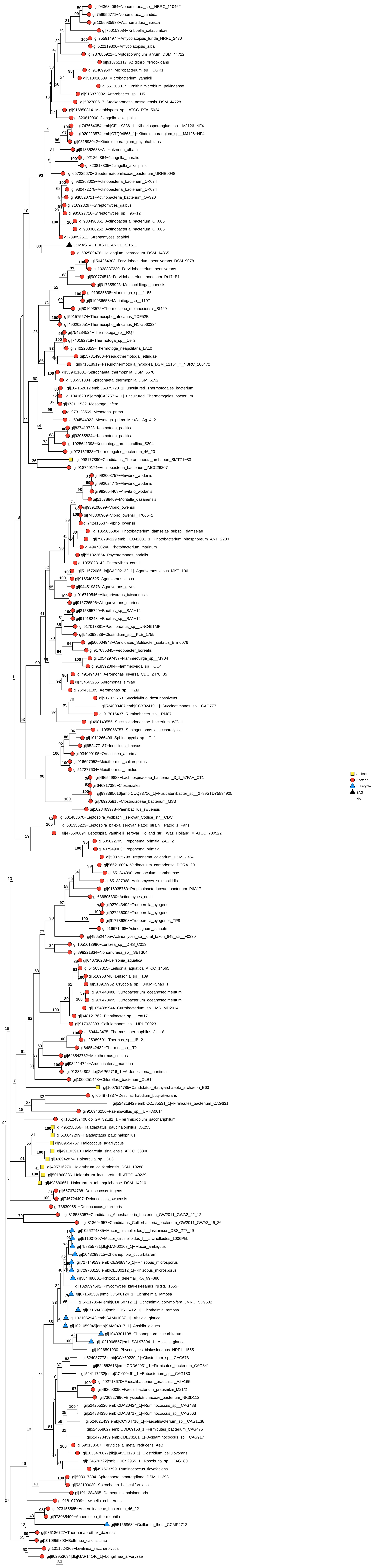
Archaea
Bacteria
Eukaryota
SAG
NA
0.1

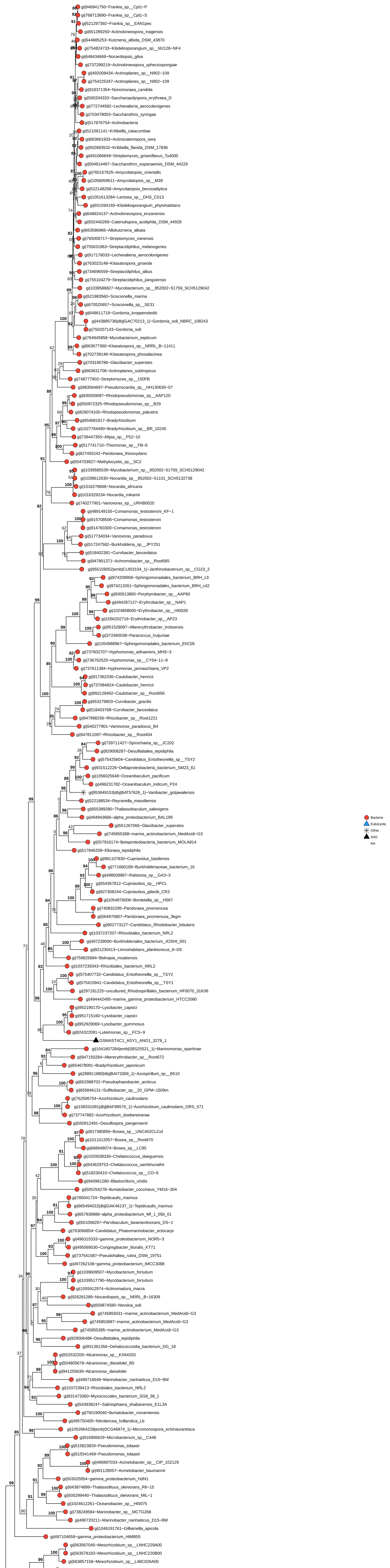

Bacteria
Eukaryota
Other
SAG
NA
0.1

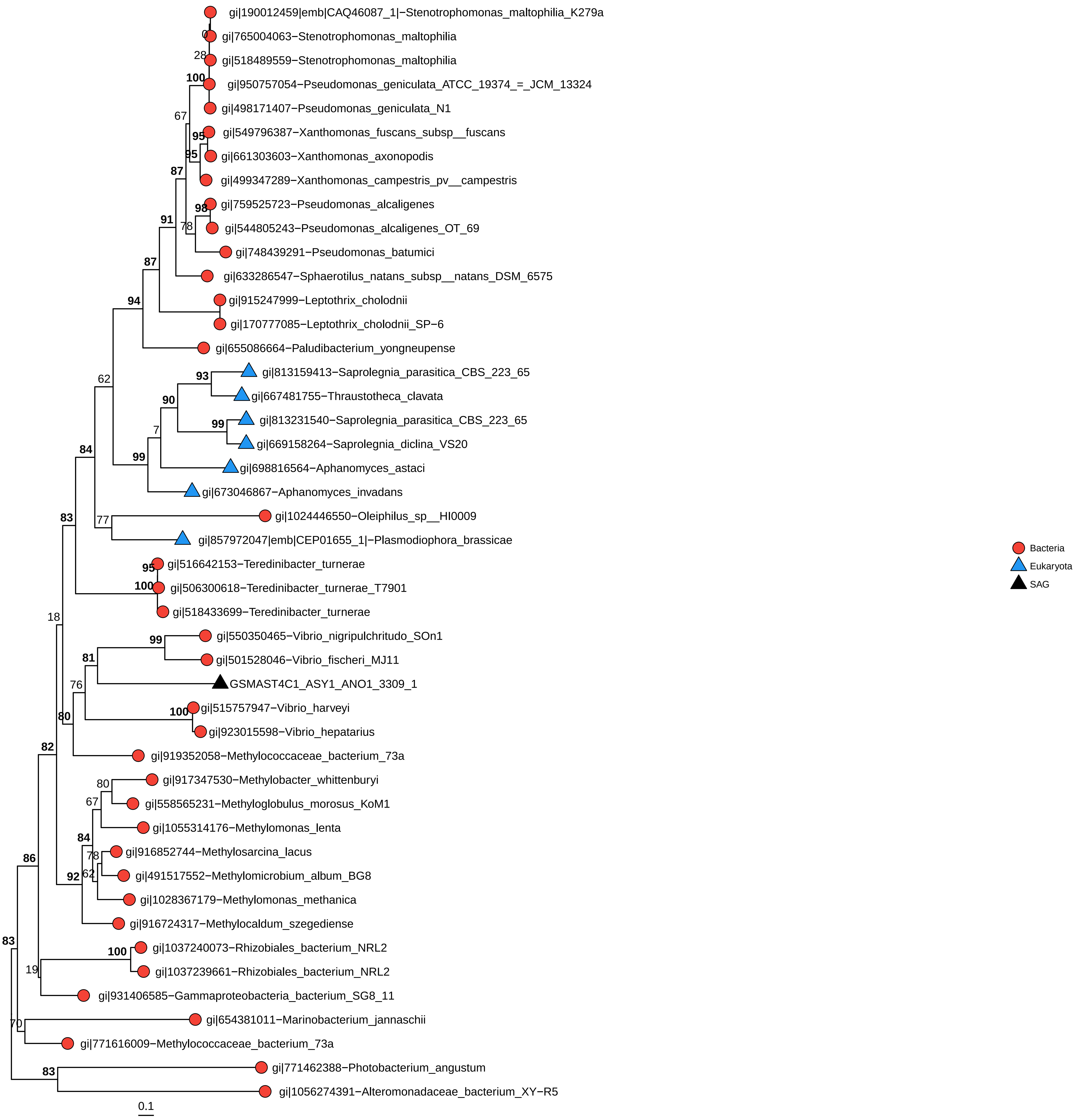
gi|190012459|emb|CAQ46087_1|−Stenotrophomonas_maltophilia_K279a
gi|765004063−Stenotrophomonas_maltophilia
gi|518489559−Stenotrophomonas_maltophilia
gi|950757054−Pseudomonas_geniculata_ATCC_19374_=_JCM_13324
gi|498171407−Pseudomonas_geniculata_N1
gi|549796387−Xanthomonas_fuscans_subsp__fuscans
gi|661303603−Xanthomonas_axonopodis
gi|499347289−Xanthomonas_campestris_pv__campestris
gi|759525723−Pseudomonas_alcaligenes
gi|544805243−Pseudomonas_alcaligenes_OT_69
gi|748439291−Pseudomonas_batumici
gi|633286547−Sphaerotilus_natans_subsp__natans_DSM_6575
gi|915247999−Leptothrix_cholodnii
gi|170777085−Leptothrix_cholodnii_SP−6
gi|655086664−Paludibacterium_yongneupense
gi|813159413−Saprolegnia_parasitica_CBS_223_65
gi|667481755−Thraustotheca_clavata
gi|813231540−Saprolegnia_parasitica_CBS_223_65
gi|669158264−Saprolegnia_diclina_VS20
gi|698816564−Aphanomyces_astaci
gi|673046867−Aphanomyces_invadans
gi|1024446550−Oleiphilus_sp__HI0009
gi|857972047|emb|CEP01655_1|−Plasmodiophora_brassicae
gi|516642153−Teredinibacter_turnerae
gi|506300618−Teredinibacter_turnerae_T7901
gi|518433699−Teredinibacter_turnerae
gi|550350465−Vibrio_nigripulchritudo_SOn1
gi|501528046−Vibrio_fischeri_MJ11
GSMAST4C1_ASY1_ANO1_3309_1
gi|515757947−Vibrio_harveyi
gi|923015598−Vibrio_hepatarius
gi|919352058−Methylococcaceae_bacterium_73a
gi|917347530−Methylobacter_whittenburyi
gi|558565231−Methyloglobulus_morosus_KoM1
gi|1055314176−Methylomonas_lenta
gi|916852744−Methylosarcina_lacus
gi|491517552−Methylomicrobium_album_BG8
gi|1028367179−Methylomonas_methanica
gi|916724317−Methylocaldum_szegediense
gi|1037240073−Rhizobiales_bacterium_NRL2
gi|1037239661−Rhizobiales_bacterium_NRL2
gi|931406585−Gammaproteobacteria_bacterium_SG8_11
gi|654381011−Marinobacterium_jannaschii
gi|771616009−Methylococcaceae_bacterium_73a
gi|771462388−Photobacterium_angustum
gi|1056274391−Alteromonadaceae_bacterium_XY−R5
0.1
Bacteria
Eukaryota
SAG

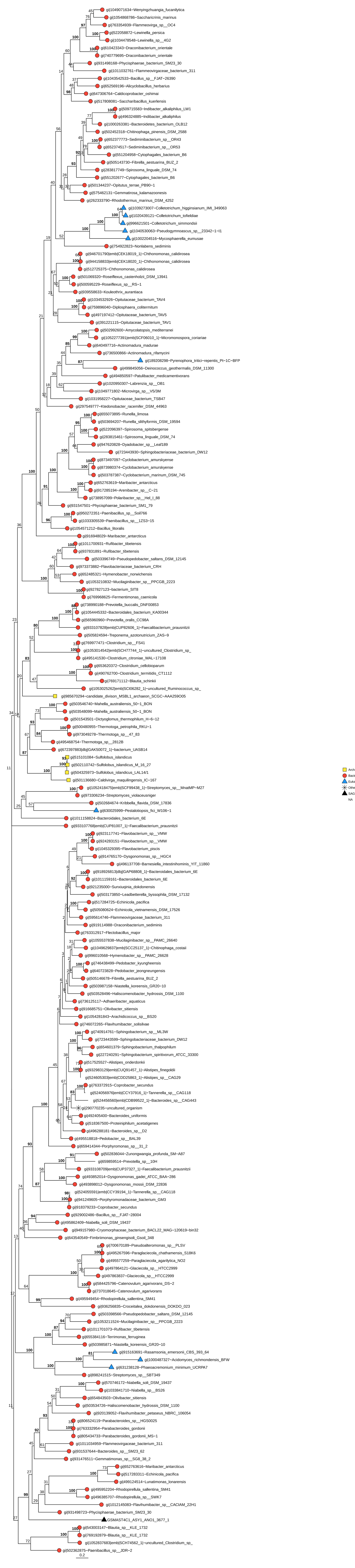
Archaea
Bacteria
Eukaryota
Other
SAG
NA
0.2

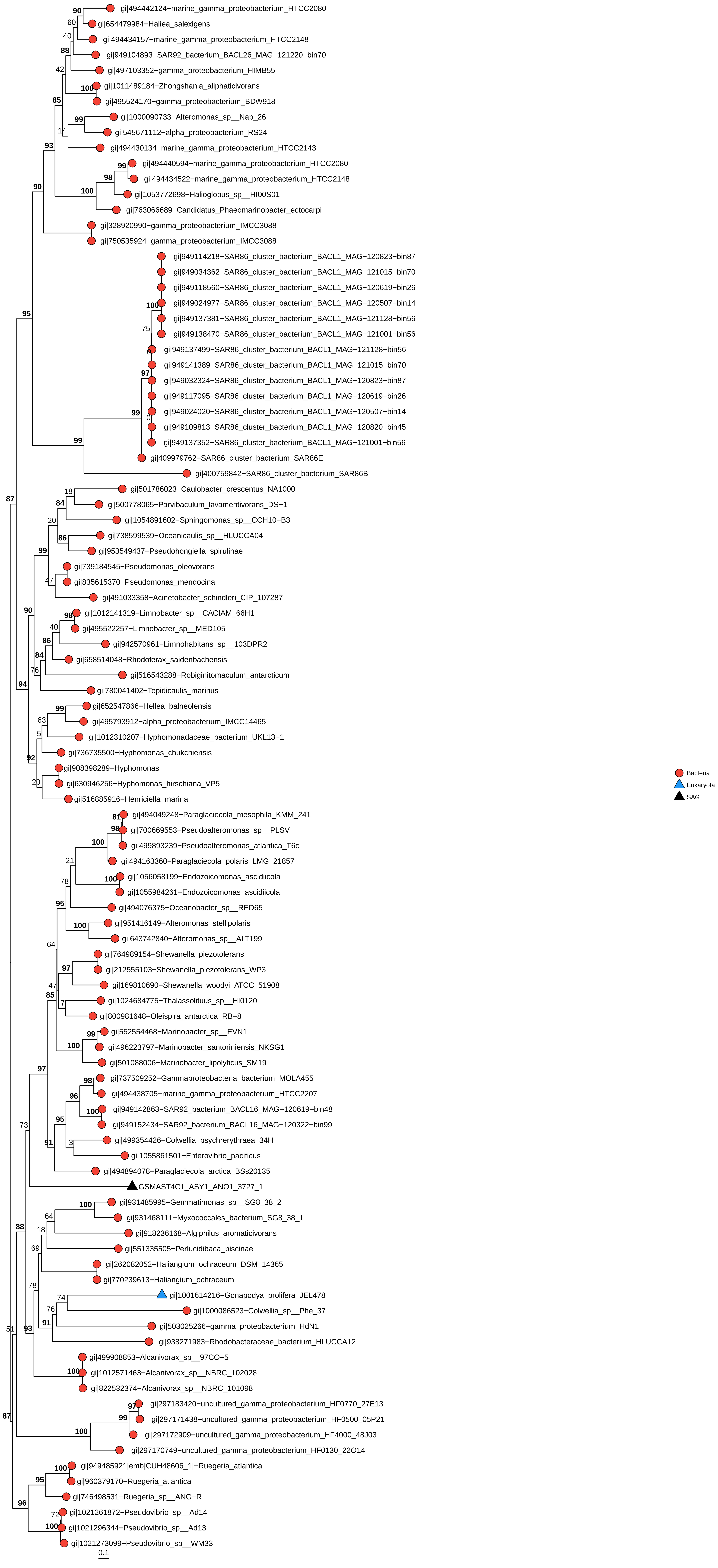
gi|494442124−marine_gamma_proteobacterium_HTCC2080
gi|654479984−Haliea_salexigens
gi|494434157−marine_gamma_proteobacterium_HTCC2148
gi|949104893−SAR92_bacterium_BACL26_MAG−121220−bin70
gi|497103352−gamma_proteobacterium_HIMB55
gi|1011489184−Zhongshania_aliphaticivorans
gi|495524170−gamma_proteobacterium_BDW918
gi|1000090733−Alteromonas_sp__Nap_26
gi|545671112−alpha_proteobacterium_RS24
gi|494430134−marine_gamma_proteobacterium_HTCC2143
gi|494440594−marine_gamma_proteobacterium_HTCC2080
gi|494434522−marine_gamma_proteobacterium_HTCC2148
gi|1053772698−Halioglobus_sp__HI00S01
gi|763066689−Candidatus_Phaeomarinobacter_ectocarpi
gi|328920990−gamma_proteobacterium_IMCC3088
gi|750535924−gamma_proteobacterium_IMCC3088
gi|949114218−SAR86_cluster_bacterium_BACL1_MAG−120823−bin87
gi|949034362−SAR86_cluster_bacterium_BACL1_MAG−121015−bin70
gi|949118560−SAR86_cluster_bacterium_BACL1_MAG−120619−bin26
gi|949024977−SAR86_cluster_bacterium_BACL1_MAG−120507−bin14
gi|949137381−SAR86_cluster_bacterium_BACL1_MAG−121128−bin56
gi|949138470−SAR86_cluster_bacterium_BACL1_MAG−121001−bin56
gi|949137499−SAR86_cluster_bacterium_BACL1_MAG−121128−bin56
gi|949141389−SAR86_cluster_bacterium_BACL1_MAG−121015−bin70
gi|949032324−SAR86_cluster_bacterium_BACL1_MAG−120823−bin87
gi|949117095−SAR86_cluster_bacterium_BACL1_MAG−120619−bin26
gi|949024020−SAR86_cluster_bacterium_BACL1_MAG−120507−bin14
gi|949109813−SAR86_cluster_bacterium_BACL1_MAG−120820−bin45
gi|949137352−SAR86_cluster_bacterium_BACL1_MAG−121001−bin56
gi|409979762−SAR86_cluster_bacterium_SAR86E
gi|400759842−SAR86_cluster_bacterium_SAR86B
gi|501786023−Caulobacter_crescentus_NA1000
gi|500778065−Parvibaculum_lavamentivorans_DS−1
gi|1054891602−Sphingomonas_sp__CCH10−B3
gi|738599539−Oceanicaulis_sp__HLUCCA04
gi|953549437−Pseudohongiella_spirulinae
gi|739184545−Pseudomonas_oleovorans
gi|835615370−Pseudomonas_mendocina
gi|491033358−Acinetobacter_schindleri_CIP_107287
gi|1012141319−Limnobacter_sp__CACIAM_66H1
gi|495522257−Limnobacter_sp__MED105
gi|942570961−Limnohabitans_sp__103DPR2
gi|658514048−Rhodoferax_saidenbachensis
gi|516543288−Robiginitomaculum_antarcticum
gi|780041402−Tepidicaulis_marinus
gi|652547866−Hellea_balneolensis
gi|495793912−alpha_proteobacterium_IMCC14465
gi|1012310207−Hyphomonadaceae_bacterium_UKL13−1
gi|736735500−Hyphomonas_chukchiensis
gi|908398289−Hyphomonas
gi|630946256−Hyphomonas_hirschiana_VP5
gi|516885916−Henriciella_marina
gi|494049248−Paraglaciecola_mesophila_KMM_241
gi|700669553−Pseudoalteromonas_sp__PLSV
gi|499893239−Pseudoalteromonas_atlantica_T6c
gi|494163360−Paraglaciecola_polaris_LMG_21857
gi|1056058199−Endozoicomonas_ascidiicola
gi|1055984261−Endozoicomonas_ascidiicola
gi|494076375−Oceanobacter_sp__RED65
gi|951416149−Alteromonas_stellipolaris
gi|643742840−Alteromonas_sp__ALT199
gi|764989154−Shewanella_piezotolerans
gi|212555103−Shewanella_piezotolerans_WP3
gi|169810690−Shewanella_woodyi_ATCC_51908
gi|1024684775−Thalassolituus_sp__HI0120
gi|800981648−Oleispira_antarctica_RB−8
gi|552554468−Marinobacter_sp__EVN1
gi|496223797−Marinobacter_santoriniensis_NKSG1
gi|501088006−Marinobacter_lipolyticus_SM19
gi|737509252−Gammaproteobacteria_bacterium_MOLA455
gi|494438705−marine_gamma_proteobacterium_HTCC2207
gi|949142863−SAR92_bacterium_BACL16_MAG−120619−bin48
gi|949152434−SAR92_bacterium_BACL16_MAG−120322−bin99
gi|499354426−Colwellia_psychrerythraea_34H
gi|1055861501−Enterovibrio_pacificus
gi|494894078−Paraglaciecola_arctica_BSs20135
GSMAST4C1_ASY1_ANO1_3727_1
gi|931485995−Gemmatimonas_sp__SG8_38_2
gi|931468111−Myxococcales_bacterium_SG8_38_1
gi|918236168−Algiphilus_aromaticivorans
gi|551335505−Perlucidibaca_piscinae
gi|262082052−Haliangium_ochraceum_DSM_14365
gi|770239613−Haliangium_ochraceum
gi|1001614216−Gonapodya_prolifera_JEL478
gi|1000086523−Colwellia_sp__Phe_37
gi|503025266−gamma_proteobacterium_HdN1
gi|938271983−Rhodobacteraceae_bacterium_HLUCCA12
gi|499908853−Alcanivorax_sp__97CO−5
gi|1012571463−Alcanivorax_sp__NBRC_102028
gi|822532374−Alcanivorax_sp__NBRC_101098
gi|297183420−uncultured_gamma_proteobacterium_HF0770_27E13
gi|297171438−uncultured_gamma_proteobacterium_HF0500_05P21
gi|297172909−uncultured_gamma_proteobacterium_HF4000_48J03
gi|297170749−uncultured_gamma_proteobacterium_HF0130_22O14
gi|949485921|emb|CUH48606_1|−Ruegeria_atlantica
gi|960379170−Ruegeria_atlantica
gi|746498531−Ruegeria_sp__ANG−R
gi|1021261872−Pseudovibrio_sp__Ad14
gi|1021296344−Pseudovibrio_sp__Ad13
gi|1021273099−Pseudovibrio_sp__WM33
0.1
Bacteria
Eukaryota
SAG

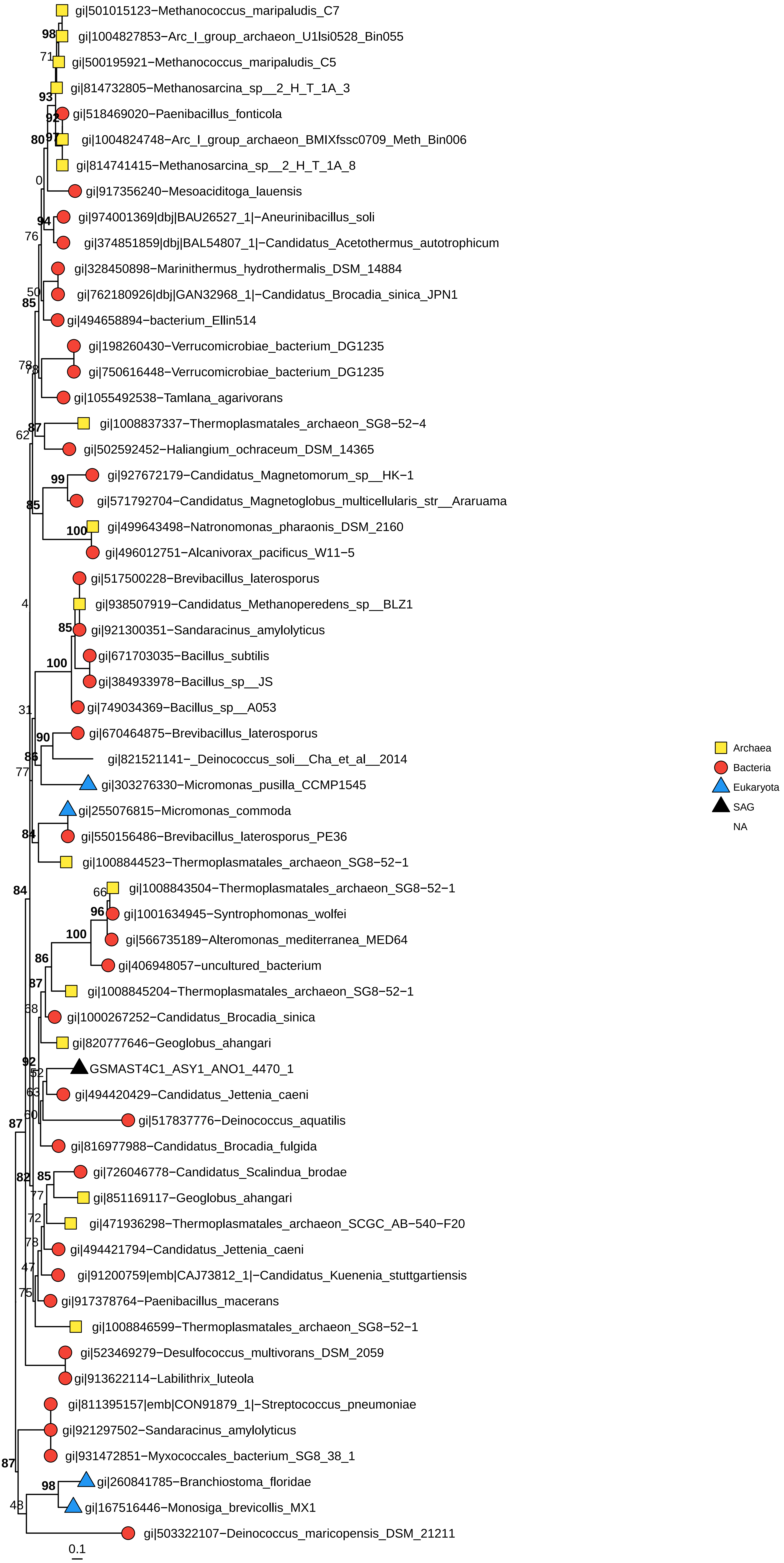
gi|501015123−Methanococcus_maripaludis_C7
gi|1004827853−Arc_I_group_archaeon_U1lsi0528_Bin055
gi|500195921−Methanococcus_maripaludis_C5
gi|814732805−Methanosarcina_sp__2_H_T_1A_3
gi|518469020−Paenibacillus_fonticola
gi|1004824748−Arc_I_group_archaeon_BMIXfssc0709_Meth_Bin006
gi|814741415−Methanosarcina_sp__2_H_T_1A_8
gi|917356240−Mesoaciditoga_lauensis
gi|974001369|dbj|BAU26527_1|−Aneurinibacillus_soli
gi|374851859|dbj|BAL54807_1|−Candidatus_Acetothermus_autotrophicum
gi|328450898−Marinithermus_hydrothermalis_DSM_14884
gi|762180926|dbj|GAN32968_1|−Candidatus_Brocadia_sinica_JPN1
gi|494658894−bacterium_Ellin514
gi|198260430−Verrucomicrobiae_bacterium_DG1235
gi|750616448−Verrucomicrobiae_bacterium_DG1235
gi|1055492538−Tamlana_agarivorans
gi|1008837337−Thermoplasmatales_archaeon_SG8−52−4
gi|502592452−Haliangium_ochraceum_DSM_14365
gi|927672179−Candidatus_Magnetomorum_sp__HK−1
gi|571792704−Candidatus_Magnetoglobus_multicellularis_str__Araruama
gi|499643498−Natronomonas_pharaonis_DSM_2160
gi|496012751−Alcanivorax_pacificus_W11−5
gi|517500228−Brevibacillus_laterosporus
gi|938507919−Candidatus_Methanoperedens_sp__BLZ1
gi|921300351−Sandaracinus_amylolyticus
gi|671703035−Bacillus_subtilis
gi|384933978−Bacillus_sp__JS
gi|749034369−Bacillus_sp__A053
gi|670464875−Brevibacillus_laterosporus
gi|821521141−_Deinococcus_soli__Cha_et_al__2014
gi|303276330−Micromonas_pusilla_CCMP1545
gi|255076815−Micromonas_commoda
gi|550156486−Brevibacillus_laterosporus_PE36
gi|1008844523−Thermoplasmatales_archaeon_SG8−52−1
gi|1008843504−Thermoplasmatales_archaeon_SG8−52−1
gi|1001634945−Syntrophomonas_wolfei
gi|566735189−Alteromonas_mediterranea_MED64
gi|406948057−uncultured_bacterium
gi|1008845204−Thermoplasmatales_archaeon_SG8−52−1
gi|1000267252−Candidatus_Brocadia_sinica
gi|820777646−Geoglobus_ahangari
GSMAST4C1_ASY1_ANO1_4470_1
gi|494420429−Candidatus_Jettenia_caeni
gi|517837776−Deinococcus_aquatilis
gi|816977988−Candidatus_Brocadia_fulgida
gi|726046778−Candidatus_Scalindua_brodae
gi|851169117−Geoglobus_ahangari
gi|471936298−Thermoplasmatales_archaeon_SCGC_AB−540−F20
gi|494421794−Candidatus_Jettenia_caeni
gi|91200759|emb|CAJ73812_1|−Candidatus_Kuenenia_stuttgartiensis
gi|917378764−Paenibacillus_macerans
gi|1008846599−Thermoplasmatales_archaeon_SG8−52−1
gi|523469279−Desulfococcus_multivorans_DSM_2059
gi|913622114−Labilithrix_luteola
gi|811395157|emb|CON91879_1|−Streptococcus_pneumoniae
gi|921297502−Sandaracinus_amylolyticus
gi|931472851−Myxococcales_bacterium_SG8_38_1
gi|260841785−Branchiostoma_floridae
gi|167516446−Monosiga_brevicollis_MX1
gi|503322107−Deinococcus_maricopensis_DSM_21211
0.1
Archaea
Bacteria
Eukaryota
SAG
NA

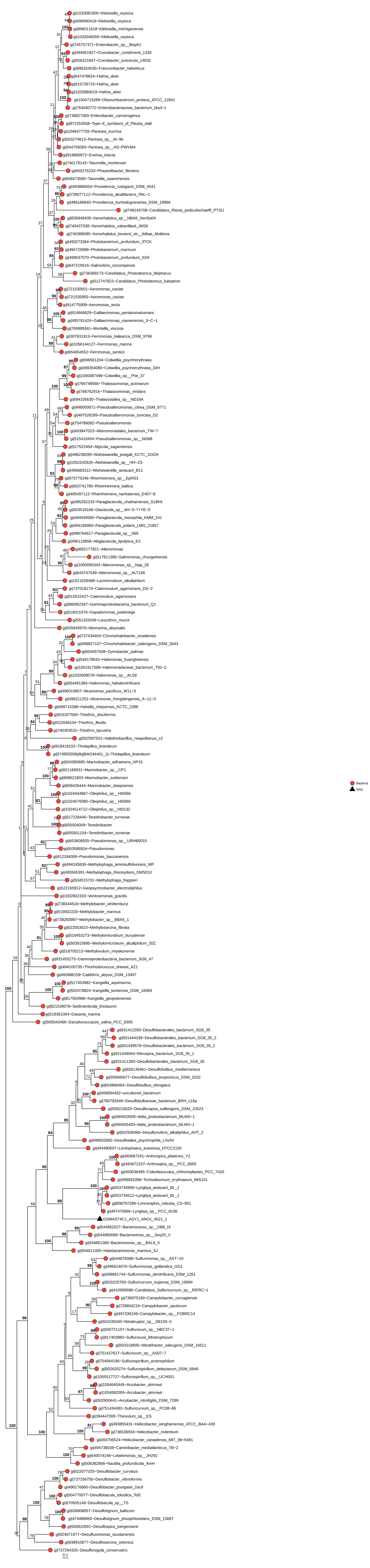
Bacteria
SAG
0.1

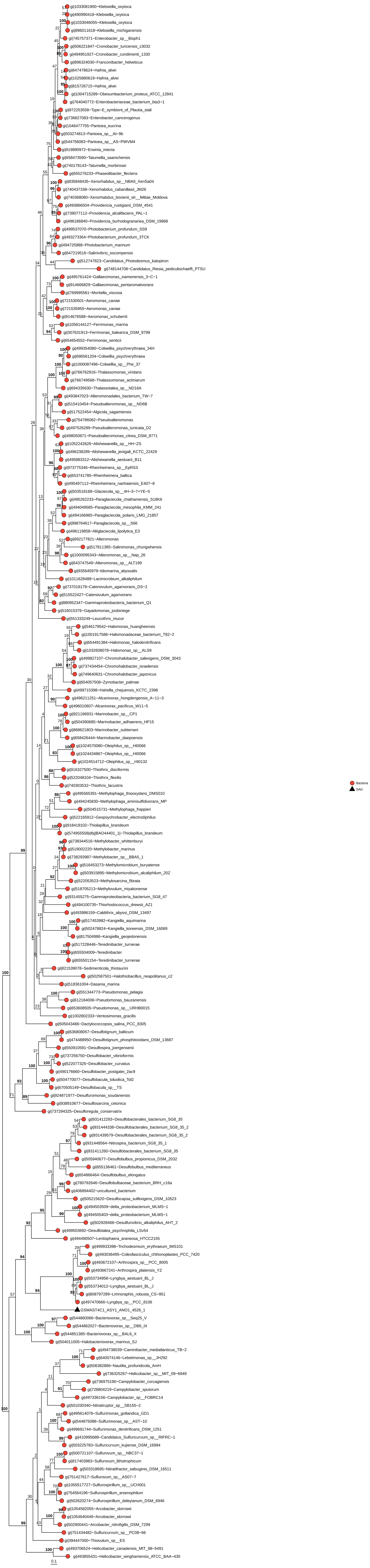
Bacteria
SAG
0.1

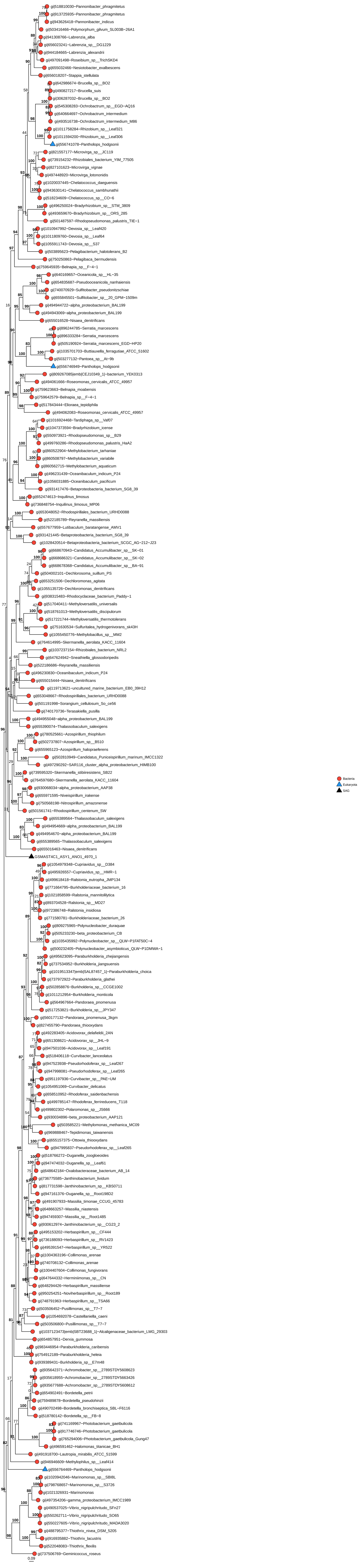
Bacteria
Eukaryota
SAG
0.09

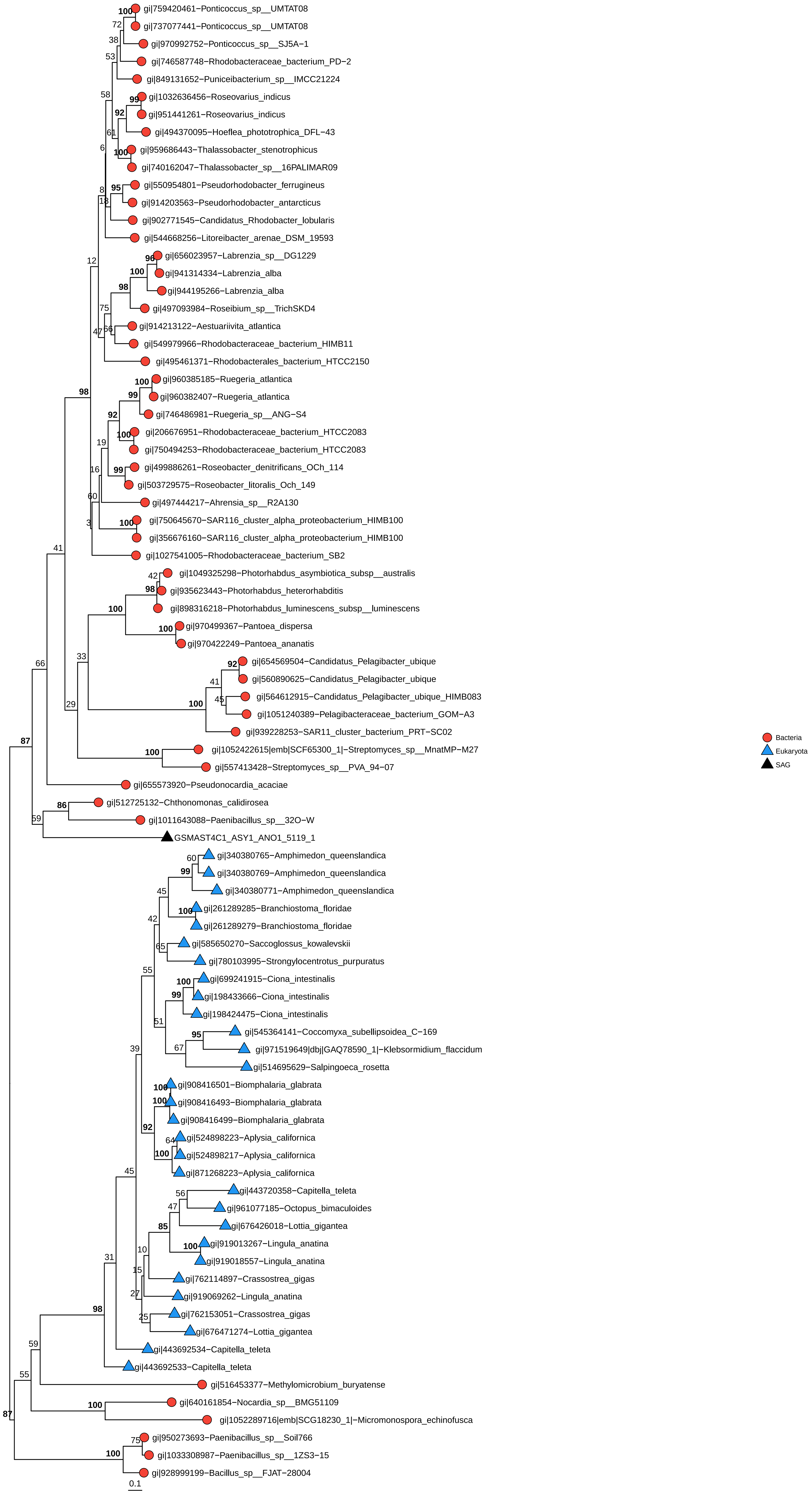
gi|759420461−Ponticoccus_sp__UMTAT08
gi|737077441−Ponticoccus_sp__UMTAT08
gi|970992752−Ponticoccus_sp__SJ5A−1
gi|746587748−Rhodobacteraceae_bacterium_PD−2
gi|849131652−Puniceibacterium_sp__IMCC21224
gi|1032636456−Roseovarius_indicus
gi|951441261−Roseovarius_indicus
gi|494370095−Hoeflea_phototrophica_DFL−43
gi|959686443−Thalassobacter_stenotrophicus
gi|740162047−Thalassobacter_sp__16PALIMAR09
gi|550954801−Pseudorhodobacter_ferrugineus
gi|914203563−Pseudorhodobacter_antarcticus
gi|902771545−Candidatus_Rhodobacter_lobularis
gi|544668256−Litoreibacter_arenae_DSM_19593
gi|656023957−Labrenzia_sp__DG1229
gi|941314334−Labrenzia_alba
gi|944195266−Labrenzia_alba
gi|497093984−Roseibium_sp__TrichSKD4
gi|914213122−Aestuariivita_atlantica
gi|549979966−Rhodobacteraceae_bacterium_HIMB11
gi|495461371−Rhodobacterales_bacterium_HTCC2150
gi|960385185−Ruegeria_atlantica
gi|960382407−Ruegeria_atlantica
gi|746486981−Ruegeria_sp__ANG−S4
gi|206676951−Rhodobacteraceae_bacterium_HTCC2083
gi|750494253−Rhodobacteraceae_bacterium_HTCC2083
gi|499886261−Roseobacter_denitrificans_OCh_114
gi|503729575−Roseobacter_litoralis_Och_149
gi|497444217−Ahrensia_sp__R2A130
gi|750645670−SAR116_cluster_alpha_proteobacterium_HIMB100
gi|356676160−SAR116_cluster_alpha_proteobacterium_HIMB100
gi|1027541005−Rhodobacteraceae_bacterium_SB2
gi|1049325298−Photorhabdus_asymbiotica_subsp__australis
gi|935623443−Photorhabdus_heterorhabditis
gi|898316218−Photorhabdus_luminescens_subsp__luminescens
gi|970499367−Pantoea_dispersa
gi|970422249−Pantoea_ananatis
gi|654569504−Candidatus_Pelagibacter_ubique
gi|560890625−Candidatus_Pelagibacter_ubique
gi|564612915−Candidatus_Pelagibacter_ubique_HIMB083
gi|1051240389−Pelagibacteraceae_bacterium_GOM−A3
gi|939228253−SAR11_cluster_bacterium_PRT−SC02
gi|1052422615|emb|SCF65300_1|−Streptomyces_sp__MnatMP−M27
gi|557413428−Streptomyces_sp__PVA_94−07
gi|655573920−Pseudonocardia_acaciae
gi|512725132−Chthonomonas_calidirosea
gi|1011643088−Paenibacillus_sp__32O−W
GSMAST4C1_ASY1_ANO1_5119_1
gi|340380765−Amphimedon_queenslandica
gi|340380769−Amphimedon_queenslandica
gi|340380771−Amphimedon_queenslandica
gi|261289285−Branchiostoma_floridae
gi|261289279−Branchiostoma_floridae
gi|585650270−Saccoglossus_kowalevskii
gi|780103995−Strongylocentrotus_purpuratus
gi|699241915−Ciona_intestinalis
gi|198433666−Ciona_intestinalis
gi|198424475−Ciona_intestinalis
gi|545364141−Coccomyxa_subellipsoidea_C−169
gi|971519649|dbj|GAQ78590_1|−Klebsormidium_flaccidum
gi|514695629−Salpingoeca_rosetta
gi|908416501−Biomphalaria_glabrata
gi|908416493−Biomphalaria_glabrata
gi|908416499−Biomphalaria_glabrata
gi|524898223−Aplysia_californica
gi|524898217−Aplysia_californica
gi|871268223−Aplysia_californica
gi|443720358−Capitella_teleta
gi|961077185−Octopus_bimaculoides
gi|676426018−Lottia_gigantea
gi|919013267−Lingula_anatina
gi|919018557−Lingula_anatina
gi|762114897−Crassostrea_gigas
gi|919069262−Lingula_anatina
gi|762153051−Crassostrea_gigas
gi|676471274−Lottia_gigantea
gi|443692534−Capitella_teleta
gi|443692533−Capitella_teleta
gi|516453377−Methylomicrobium_buryatense
gi|640161854−Nocardia_sp__BMG51109
gi|1052289716|emb|SCG18230_1|−Micromonospora_echinofusca
gi|950273693−Paenibacillus_sp__Soil766
gi|1033308987−Paenibacillus_sp__1ZS3−15
gi|928999199−Bacillus_sp__FJAT−28004
0.1
Bacteria
Eukaryota
SAG

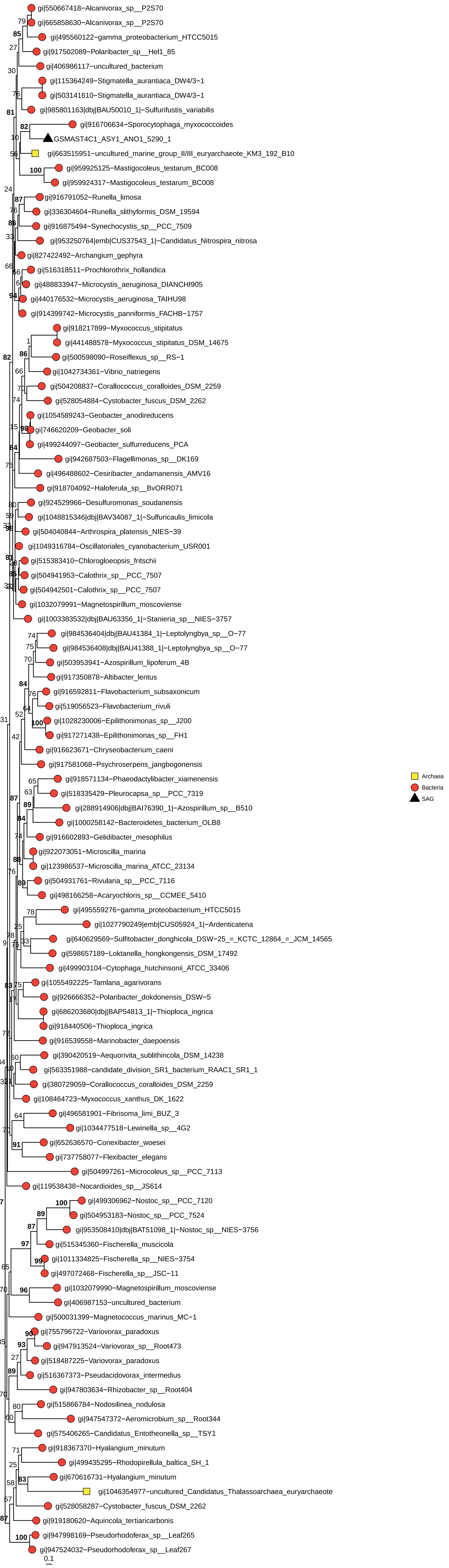
gi|550667418-Alcanivorax_sp__P2S70
gi|665858630-Alcanivorax_sp__P2S70
gi|495560122-gamma_proteobacterium_HTCC5015
gi|917502089-Polaribacter_sp__Hel1_85
gi|406986117-uncultured_bacterium
gi|115364249-Stigmatella_aurantiaca_DW4/3-1
gi|503141610-Stigmatella_aurantiaca_DW4/3-1
gi|985801163|dbj|BAU50010_1|-Sulfurifustis_variabilis
gi|916706634-Sporocytophaga_myxococcoides
GSMAST4C1_ASY1_ANO1_5290_1
gi|663515951-uncultured_marine_group_II/III_euryarchaeote_KM3_192_B10
gi|959925125-Mastigocoleus_testarum_BC008
gi|959924317-Mastigocoleus_testarum_BC008
gi|916791052-Runella_limosa
gi|336304604-Runella_slithyformis_DSM_19594
gi|916875494-Synechocystis_sp__PCC_7509
gi|953250764|emb|CUS37543_1|-Candidatus_Nitrospira_nitrosa
gi|827422492-Archangium_gephyra
gi|516318511-Prochlorothrix_hollandica
gi|488833947-Microcystis_aeruginosa_DIANCHI905
gi|440176532-Microcystis_aeruginosa_TAIHU98
gi|914399742-Microcystis_panniformis_FACHB-1757
gi|918217899-Myxococcus_stipitatus
gi|441488578-Myxococcus_stipitatus_DSM_14675
gi|500598090-Roseiflexus_sp__RS-1
gi|1042734361-Vibrio_natriegens
gi|504208837-Corallococcus_coralloides_DSM_2259
gi|528054884-Cystobacter_fuscus_DSM_2262
gi|1054589243-Geobacter_anodireducens
gi|746620209-Geobacter_soli
gi|499244097-Geobacter_sulfurreducens_PCA
gi|942687503-Flagellimonas_sp__DK169
gi|496488602-Cesiribacter_andamanensis_AMV16
gi|918704092-Haloferula_sp__BvORR071
gi|924529966-Desulfuromonas_soudanensis
gi|1048815346|dbj|BAV34087_1|-Sulfuricaulis_limicola
gi|504040844-Arthrospira_platensis_NIES-39
gi|1049316784-Oscillatoriales_cyanobacterium_USR001
gi|515383410-Chlorogloeopsis_fritschii
gi|504941953-Calothrix_sp__PCC_7507
gi|504942501-Calothrix_sp__PCC_7507
gi|1032079991-Magnetospirillum_moscoviense
gi|1003383532|dbj|BAU63356_1|-Stanieria_sp__NIES-3757
gi|984536404|dbj|BAU41384_1|-Leptolyngbya_sp__O-77
gi|984536408|dbj|BAU41388_1|-Leptolyngbya_sp__O-77
gi|503953941-Azospirillum_lipoferum_4B
gi|917350878-Altibacter_lentus
gi|916592811-Flavobacterium_subsaxonicum
gi|519056523-Flavobacterium_rivuli
gi|1028230006-Epilithonimonas_sp__J200
gi|917271438-Epilithonimonas_sp__FH1
gi|916623671-Chryseobacterium_caeni
gi|917581068-Psychroserpens_jangbogonensis
gi|918571134-Phaeodactylibacter_xiamenensis
gi|518335429-Pleurocapsa_sp__PCC_7319
gi|288914906|dbj|BAI76390_1|-Azospirillum_sp__B510
gi|1000258142-Bacteroidetes_bacterium_OLB8
gi|916602893-Gelidibacter_mesophilus
gi|922073051-Microscilla_marina
gi|123986537-Microscilla_marina_ATCC_23134
gi|504931761-Rivularia_sp__PCC_7116
gi|498166258-Acaryochloris_sp__CCMEE_5410
gi|495559276-gamma_proteobacterium_HTCC5015
gi|1027790249|emb|CUS05924_1|-Ardenticatena
gi|640629569-Sulfitobacter_donghicola_DSW-25_=_KCTC_12864_=_JCM_14565
gi|598657189-Loktanella_hongkongensis_DSM_17492
gi|499903104-Cytophaga_hutchinsonii_ATCC_33406
gi|1055492225-Tamlana_agarivorans
gi|926666352-Polaribacter_dokdonensis_DSW-5
gi|686203680|dbj|BAP54813_1|-Thioploca_ingrica
gi|918440506-Thioploca_ingrica
gi|916539558-Marinobacter_daepoensis
gi|390420519-Aequorivita_sublithincola_DSM_14238
gi|563351988-candidate_division_SR1_bacterium_RAAC1_SR1_1
gi|380729059-Corallococcus_coralloides_DSM_2259
gi|108464723-Myxococcus_xanthus_DK_1622
gi|496581901-Fibrisoma_limi_BUZ_3
gi|1034477518-Lewinella_sp__4G2
gi|652636570-Conexibacter_woesei
gi|737758077-Flexibacter_elegans
gi|504997261-Microcoleus_sp__PCC_7113
gi|119538438-Nocardioides_sp__JS614
gi|499306962-Nostoc_sp__PCC_7120
gi|504953183-Nostoc_sp__PCC_7524
gi|953508410|dbj|BAT51098_1|-Nostoc_sp__NIES-3756
gi|515345360-Fischerella_muscicola
gi|1011334825-Fischerella_sp__NIES-3754
gi|497072468-Fischerella_sp__JSC-11
gi|1032079990-Magnetospirillum_moscoviense
gi|406987153-uncultured_bacterium
gi|500031399-Magnetococcus_marinus_MC-1
gi|755796722-Variovorax_paradoxus
gi|947913524-Variovorax_sp__Root473
gi|518487225-Variovorax_paradoxus
gi|516367373-Pseudacidovorax_intermedius
gi|947803634-Rhizobacter_sp__Root404
gi|515866784-Nodosilinea_nodulosa
gi|947547372-Aeromicrobium_sp__Root344
gi|575406265-Candidatus_Entotheonella_sp__TSY1
gi|918367370-Hyalangium_minutum
gi|499435295-Rhodopirellula_baltica_SH_1
gi|670616731-Hyalangium_minutum
gi|1046354977-uncultured_Candidatus_Thalassoarchaea_euryarchaeote
gi|528058287-Cystobacter_fuscus_DSM_2262
gi|919180620-Aquincola_tertiaricarbonis
gi|947998169-Pseudorhodoferax_sp__Leaf265
gi|947524032-Pseudorhodoferax_sp__Leaf267
0.1
Archaea
Bacteria
SAG

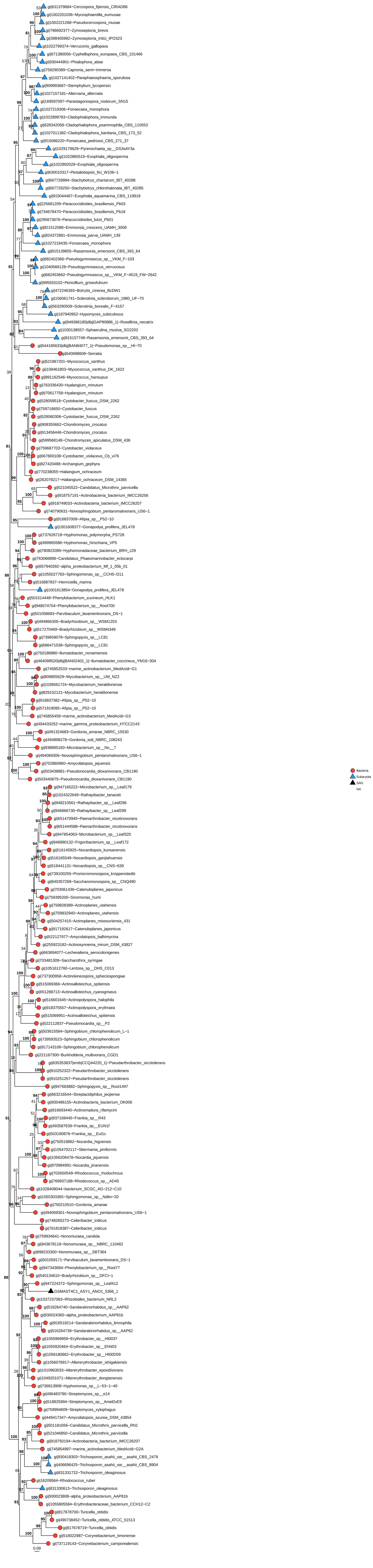
gi|631379684~Cercospora_fijiensis_CIRAD86
gi|1002201036~Mycosphaerella_eumusae
gi|1002221268~Pseudocercospora_musae
gi|796692377~Zymoseptoria_brevis
gi|398405992~Zymoseptoria_tritici_IPO323
gi|1022799374~Verruconis_gallopava
gi|671380056~Cyphellophora_europaea_CBS_101466
gi|930444901~Phialophora_attae
gi|759290389~Capronia_semi~immersa
gi|1027141402~Paraphaeosphaeria_sporulosa
gi|909993687~Stemphylium_lycopersici
gi|1027157181~Alternaria_alternata
gi|169597097~Parastagonospora_nodorum_SN15
gi|1027219306~Fonsecaea_monophora
gi|1022899783~Cladophialophora_immunda
gi|628342058~Cladophialophora_psammophila_CBS_110553
gi|1027011382~Cladophialophora_bantiana_CBS_173_52
gi|915086220~Fonsecaea_pedrosoi_CBS_271_37
gi|1029179629~Pyrenochaeta_sp__DS3sAY3a
gi|1022865515~Exophiala_oligosperma
gi|1022892029~Exophiala_oligosperma
gi|630010317~Pestalotiopsis_fici_W106~1
gi|667729994~Stachybotrys_chartarum_IBT_40288
gi|667729250~Stachybotrys_chlorohalonata_IBT_40285
gi|915044467~Exophiala_aquamarina_CBS_119918
gi|225681209~Paracoccidioides_brasiliensis_Pb03
gi|734678470~Paracoccidioides_brasiliensis_Pb18
gi|295673676~Paracoccidioides_lutzii_Pb01
gi|821512088~Emmonsia_crescens_UAMH_3008
gi|824372881~Emmonsia_parva_UAMH_139
gi|1027218435~Fonsecaea_monophora
gi|915139855~Rasamsonia_emersonii_CBS_393_64
gi|682402366~Pseudogymnoascus_sp__VKM_F~103
gi|1040566128~Pseudogymnoascus_verrucosus
gi|682453662~Pseudogymnoascus_sp__VKM_F~4519_FW~2642
gi|995933152~Penicillium_griseofulvum
gi|472246393~Botrytis_cinerea_BcDW1
gi|156061741~Sclerotinia_sclerotiorum_1980_UF~70
gi|563290509~Sclerotinia_borealis_F~4157
gi|187940952~Hypomyces_subiculosus
gi|949386185|dbj|GAP90886_1|~Rosellinia_necatrix
gi|1030138557~Sphaerulina_musiva_SO2202
gi|915157748~Rasamsonia_emersonii_CBS_393_64
gi|544185633|dbj|BAN84077_1|~Pseudomonas_sp__HI~70
gi|640698609~Serratia
gi|521967201~Myxococcus_xanthus
gi|108461803~Myxococcus_xanthus_DK_1622
gi|891162546~Myxococcus_hansupus
gi|763336430~Hyalangium_minutum
gi|670617758~Hyalangium_minutum
gi|528058518~Cystobacter_fuscus_DSM_2262
gi|759716650~Cystobacter_fuscus
gi|528060306~Cystobacter_fuscus_DSM_2262
gi|908355662~Chondromyces_crocatus
gi|913456446~Chondromyces_crocatus
gi|599568148~Chondromyces_apiculatus_DSM_436
gi|759687703~Cystobacter_violaceus
gi|667800108~Cystobacter_violaceus_Cb_vi76
gi|827420488~Archangium_gephyra
gi|770238055~Haliangium_ochraceum
gi|262078217~Haliangium_ochraceum_DSM_14365
gi|521045522~Candidatus_Microthrix_parvicella
gi|918757191~Actinobacteria_bacterium_IMCC26256
gi|918749033~Actinobacteria_bacterium_IMCC26207
gi|740790631~Novosphingobium_pentaromativorans_US6~1
gi|916837009~Afipia_sp__P52~10
gi|1001608377~Gonapodya_prolifera_JEL478
gi|737626718~Hyphomonas_polymorpha_PS728
gi|499965586~Hyphomonas_hirschiana_VP5
gi|780823395~Hyphomonadaceae_bacterium_BRH_c29
gi|763066896~Candidatus_Phaeomarinobacter_ectocarpi
gi|657940392~alpha_proteobacterium_Mf_1_05b_01
gi|1055027783~Sphingomonas_sp__CCH5~D11
gi|516887837~Henriciella_marina
gi|1001613854~Gonapodya_prolifera_JEL478
gi|501514448~Phenylobacterium_zucineum_HLK1
gi|948074704~Phenylobacterium_sp__Root700
gi|501058683~Parvibaculum_lavamentivorans_DS~1
gi|494866305~Bradyrhizobium_sp__WSM1253
gi|517270469~Bradyrhizobium_sp__WSM4349
gi|739659078~Sphingopyxis_sp__LC81
gi|686471538~Sphingopyxis_sp__LC81
gi|750186880~Illumatobacter_nonamiensis
gi|464099520|dbj|BAN02402_1|~Illumatobacter_coccineus_YM16~304
gi|745852349~marine_actinobacterium_MedAcidi~G1
gi|808805629~Mycobacterium_sp__UM_NZ2
gi|1039561724~Mycobacterium_heraklionense
gi|829152121~Mycobacterium_heraklionense
gi|916837382~Afipia_sp__P52~10
gi|571919095~Afipia_sp__P52~10
gi|745855459~marine_actinobacterium_MedAcidi~G3
gi|494433252~marine_gamma_proteobacterium_HTCC2143
gi|491324683~Gordonia_amarae_NBRC_15530
gi|494898278~Gordonia_soli_NBRC_108243
gi|938895183~Microbacterium_sp__No__7
gi|494069306~Novosphingobium_pentaromativorans_US6~1
gi|702860960~Amycolatopsis_jejuensis
gi|503438881~Pseudonocardia_dioxanivorans_CB1190
gi|503440675~Pseudonocardia_dioxanivorans_CB1190
gi|947165222~Microbacterium_sp__Leaf179
gi|1024322649~Rathayibacter_tanaceti
gi|948210561~Rathayibacter_sp__Leaf296
gi|946866730~Rathayibacter_sp__Leaf299
gi|651470945~Paenarthrobacter_nicotinovorans
gi|651444588~Paenarthrobacter_nicotinovorans
gi|947854063~Microbacterium_sp__Leaf320
gi|946890132~Frigoribacterium_sp__Leaf172
gi|516145925~Nocardiopsis_kunsanensis
gi|516165549~Nocardiopsis_ganjiahuensis
gi|518441131~Nocardiopsis_sp__CNS~639
gi|739100255~Promicromonospora_kroppenstedtii
gi|640357269~Saccharomonospora_sp__CNQ490
gi|703061436~Catenuloplanes_japonicus
gi|759395200~Sinomonas_humi
gi|759828389~Actinoplanes_utahensis
gi|759832940~Actinoplanes_utahensis
gi|504257415~Actinoplanes_missouriensis_431
gi|917192617~Catenuloplanes_japonicus
gi|522127977~Amycolatopsis_balhimycina
gi|255923182~Actinosynnema_mirum_DSM_43827
gi|663694077~Lechevalieria_aerocolonigenes
gi|703481309~Saccharothrix_syringae
gi|1051612760~Lentzea_sp__DHS_C013
gi|737300958~Actinokineospora_spheciospongiae
gi|515069366~Actinoalloteichus_spitiensis
gi|651288713~Actinoalloteichus_cyanogriseus
gi|516601645~Actinopolyspora_halophila
gi|918375557~Actinopolyspora_erythraea
gi|515069951~Actinoalloteichus_spitiensis
gi|522112837~Pseudonocardia_sp__P2
gi|503615584~Sphingobium_chlorophenolicum_L~1
gi|739593523~Sphingobium_chlorophenolicum
gi|917143106~Sphingobium_chlorophenolicum
gi|221167300~Burkholderia_multivorans_CGD1
gi|635353837|emb|CCQ44220_1|~Pseudarthrobacter_siccitolerans
gi|910252322~Pseudarthrobacter_siccitolerans
gi|910251257~Pseudarthrobacter_siccitolerans
gi|947683882~Sphingopyxis_sp__Root1497
gi|663216544~Streptacidiphilus_jeojiense
gi|930486155~Actinobacteria_bacterium_OK006
gi|916693440~Actinomadura_rifamycini
gi|937169445~Frankia_sp__R43
gi|493587639~Frankia_sp__EUN1f
gi|503190876~Frankia_sp__EuI1c
gi|750518862~Nocardia_higoensis
gi|1054702117~Skermania_piniformis
gi|1056206478~Nocardia_jejuensis
gi|970984991~Nocardia_jinanensis
gi|702650549~Rhodococcus_rhodochrous
gi|769937188~Rhodococcus_sp__AD45
gi|1028408044~bacterium_SCGC_AG~212~C10
gi|1055303565~Sphingomonas_sp__Ndbn~20
gi|750210510~Gordonia_amarae
gi|494069301~Novosphingobium_pentaromativorans_US6~1
gi|748265273~Celeribacter_indicus
gi|761818387~Celeribacter_indicus
gi|759934641~Nonomuraea_candida
gi|943678118~Nonomuraea_sp__NBRC_110462
gi|898233300~Nonomuraea_sp__SBT364
gi|501059171~Parvibaculum_lavamentivorans_DS~1
gi|947343684~Phenylobacterium_sp__Root77
gi|540134610~Bradyrhizobium_sp__DFCI~1
gi|947224372~Sphingomonas_sp__Leaf412
GSMAST4C1_ASY1_ANO1_5366_1
gi|1037237083~Rhizobiales_bacterium_NRL2
gi|516264740~Sandarakinorhabdus_sp__AAP62
gi|930024365~alpha_proteobacterium_AAP81b
gi|916518214~Sandarakinorhabdus_limnophila
gi|516264739~Sandarakinorhabdus_sp__AAP62
gi|1055969959~Erythrobacter_sp__HI0037
gi|1055920464~Erythrobacter_sp__EhN03
gi|1056180662~Erythrobacter_sp__HI00D59
gi|1056076917~Altererythrobacter_ishigakiensis
gi|1010963033~Altererythrobacter_epoxidivorans
gi|1049201071~Altererythrobacter_dongtanensis
gi|736813908~Hyphomonas_sp__L~53~1~40
gi|496483790~Streptomyces_sp__e14
gi|518825994~Streptomyces_sp__Amel2xE9
gi|759994609~Streptomyces_xylophagus
gi|449417347~Amycolatopsis_azurea_DSM_43854
gi|501181656~Candidatus_Microthrix_parvicella_RN1
gi|521046850~Candidatus_Microthrix_parvicella
gi|918750194~Actinobacteria_bacterium_IMCC26207
gi|745854997~marine_actinobacterium_MedAcidi~G2A
gi|930418303~Trichosporon_asahii_var__asahii_CBS_2479
gi|406696425~Trichosporon_asahii_var__asahii_CBS_8904
gi|831331722~Trichosporon_oleaginosus
gi|16209564~Rhodococcus_ruber
gi|831330613~Trichosporon_oleaginosus
gi|930023808~alpha_proteobacterium_AAP81b
gi|1055805584~Erythrobacteraceae_bacterium_CCH12~C2
gi|817678700~Turicella_otitidis
gi|490738452~Turicella_otitidis_ATCC_51513
gi|817678719~Turicella_otitidis
gi|518022987~Corynebacterium_timonense
gi|737119143~Corynebacterium_camporealensis
0.09
Bacteria
Eukaryota
SAG
NA

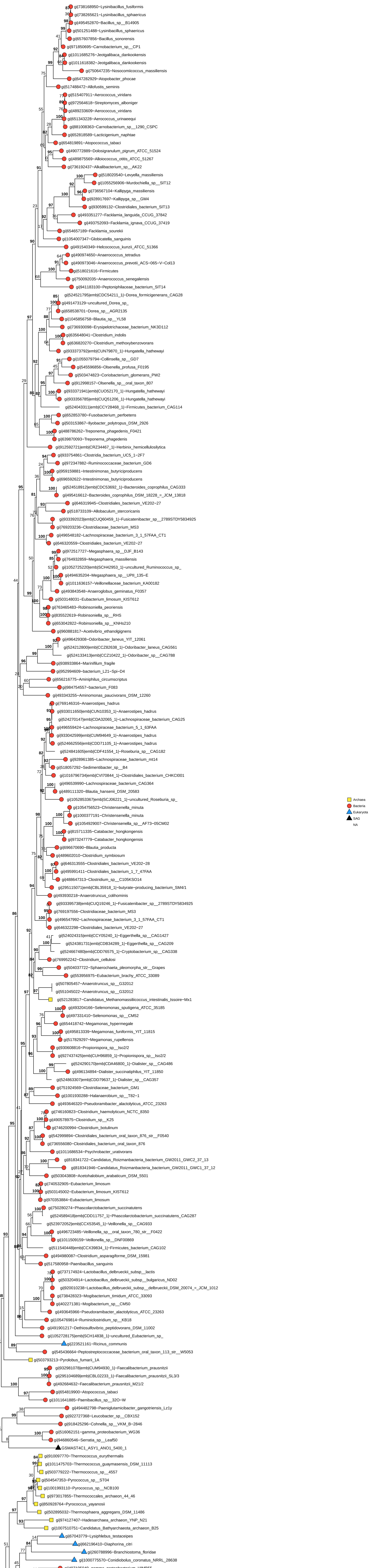
Archaea
Bacteria
Eukaryota
SAG
NA
0.1

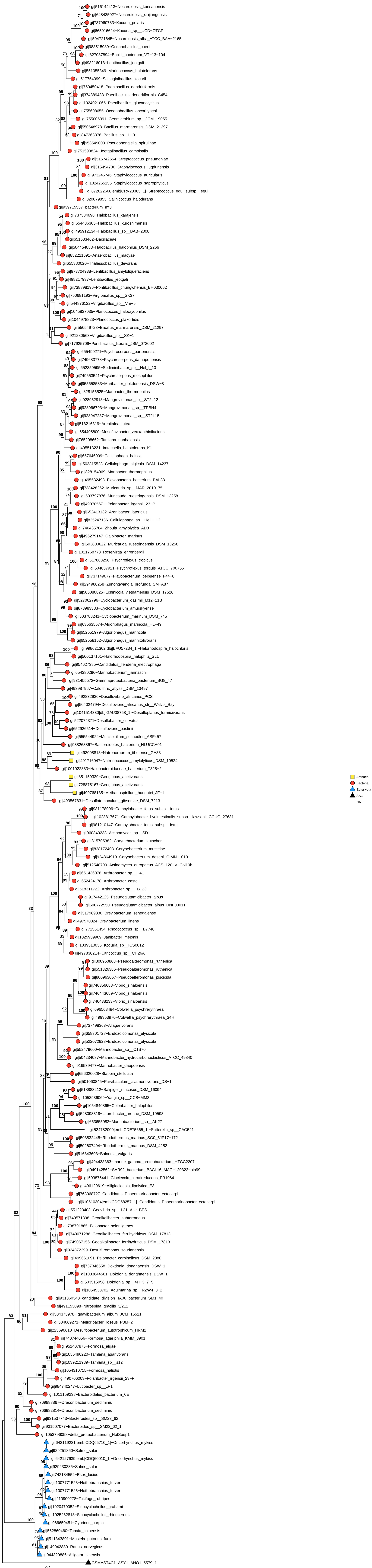
Archaea
Bacteria
Eukaryota
SAG
NA
0.1

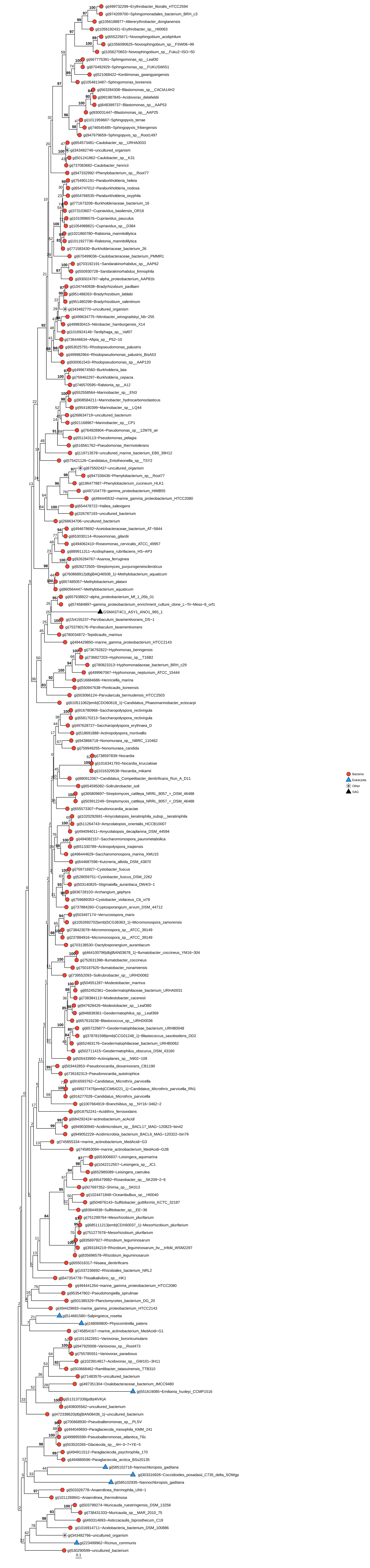
Bacteria
Eukaryota
Other
SAG
0.1

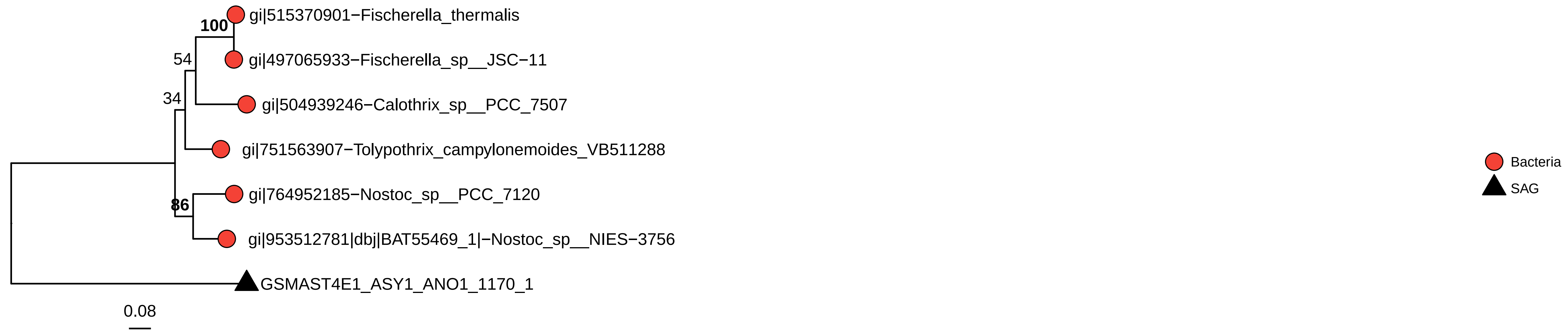

gi|515370901−Fischerella_thermalis
100
gi|497065933−Fischerella_sp__JSC−11
54
gi|504939246−Calothrix_sp__PCC_7507
34
gi|751563907−Tolypothrix_campylonemoides_VB511288
gi|764952185−Nostoc_sp__PCC_7120
86
gi|953512781|dbj|BAT55469_1|−Nostoc_sp__NIES−3756
GSMAST4E1_ASY1_ANO1_1170_1
0.08
Bacteria
SAG

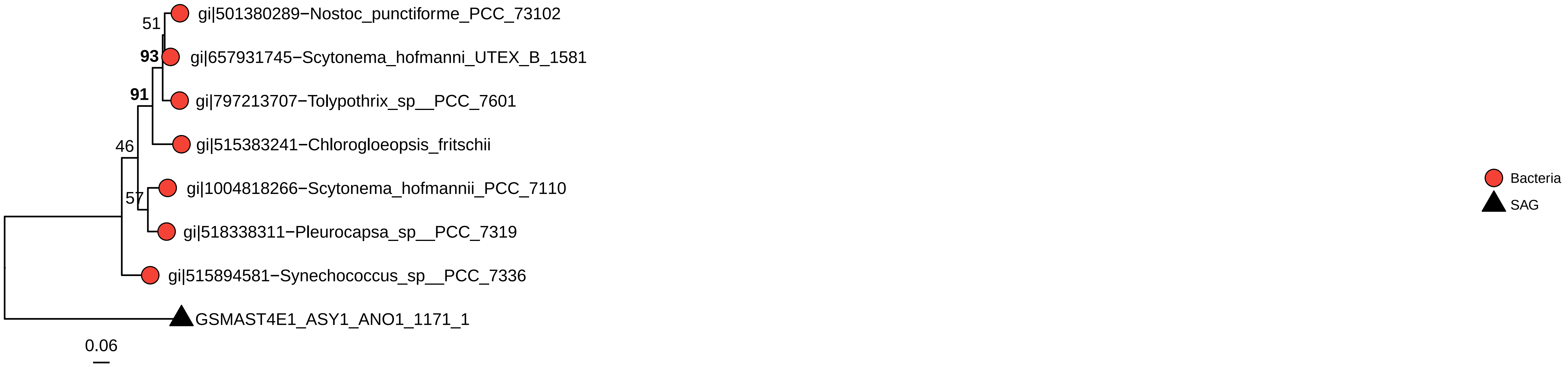
51
gi|501380289−Nostoc_punctiforme_PCC_73102
93
gi|657931745−Scytonema_hofmanni_UTEX_B_1581
91
gi|797213707−Tolypothrix_sp__PCC_7601
46
gi|515383241−Chlorogloeopsis_fritschii
gi|1004818266−Scytonema_hofmannii_PCC_7110
57
gi|518338311−Pleurocapsa_sp__PCC_7319
gi|515894581−Synechococcus_sp__PCC_7336
GSMAST4E1_ASY1_ANO1_1171_1
0.06
Bacteria
SAG

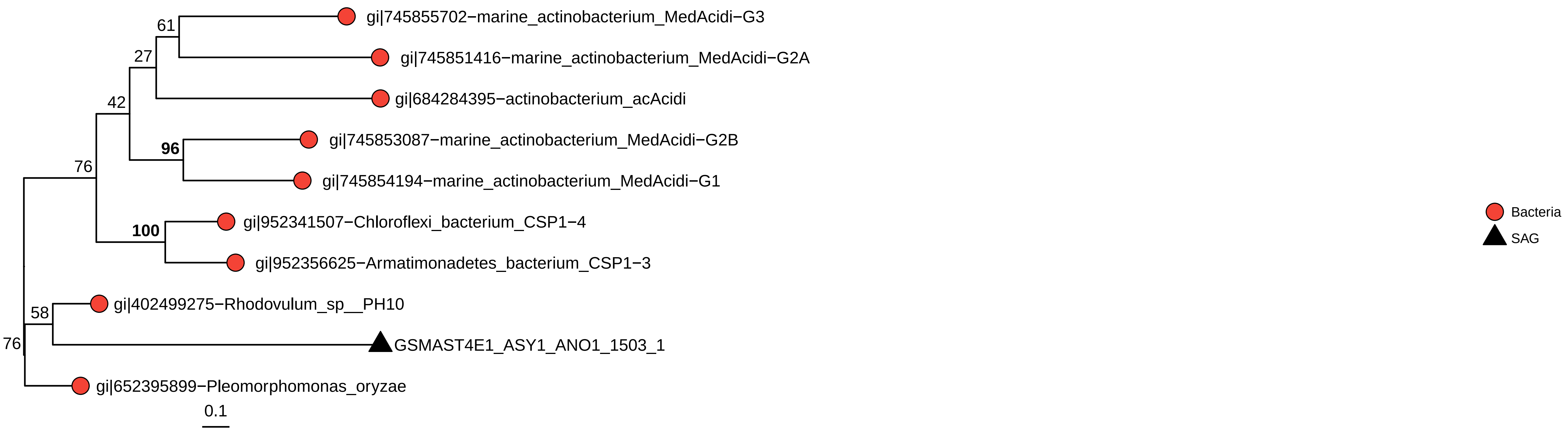
61
gi|745855702−marine_actinobacterium_MedAcidi−G3
27
gi|745851416−marine_actinobacterium_MedAcidi−G2A
42
gi|684284395−actinobacterium_acAcidi
96
gi|745853087−marine_actinobacterium_MedAcidi−G2B
76
gi|745854194−marine_actinobacterium_MedAcidi−G1
100
gi|952341507−Chloroflexi_bacterium_CSP1−4
gi|952356625−Armatimonadetes_bacterium_CSP1−3
58
gi|402499275−Rhodovulum_sp__PH10
76
GSMAST4E1_ASY1_ANO1_1503_1
gi|652395899−Pleomorphomonas_oryzae
0.1
Bacteria
SAG

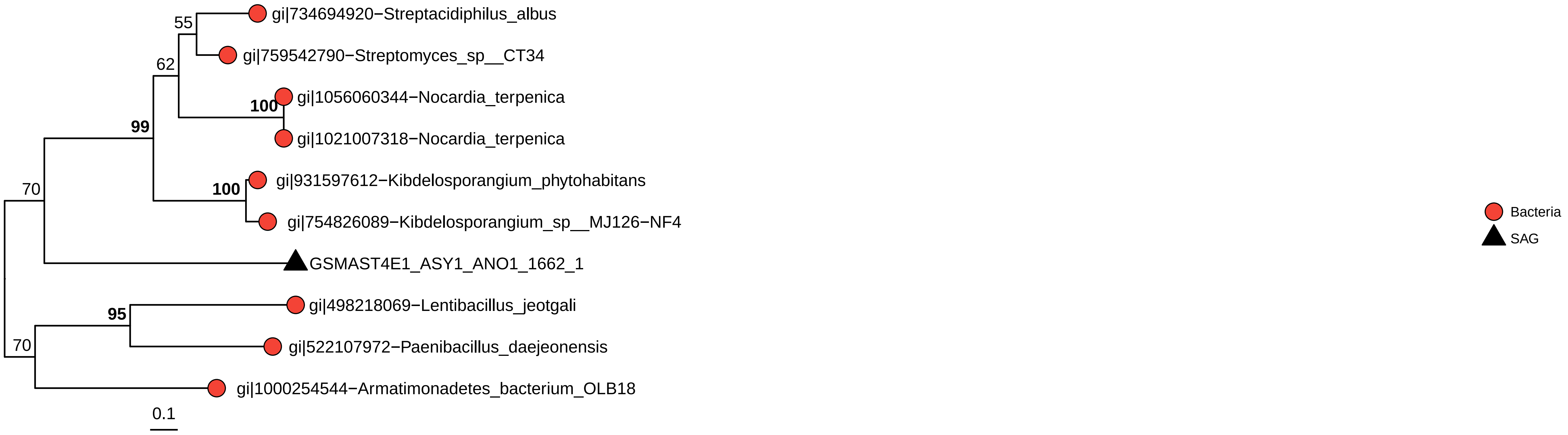
55
62
99
100
100
70
95
70
gi|734694920−Streptacidiphilus_albus
gi|759542790−Streptomyces_sp__CT34
gi|1056060344−Nocardia_terpenica
gi|1021007318−Nocardia_terpenica
gi|931597612−Kibdelosporangium_phytohabitans
gi|754826089−Kibdelosporangium_sp__MJ126−NF4
GSMAST4E1_ASY1_ANO1_1662_1
gi|498218069−Lentibacillus_jeotgali
gi|522107972−Paenibacillus_daejeonensis
gi|1000254544−Armatimonadetes_bacterium_OLB18
0.1
Bacteria
SAG

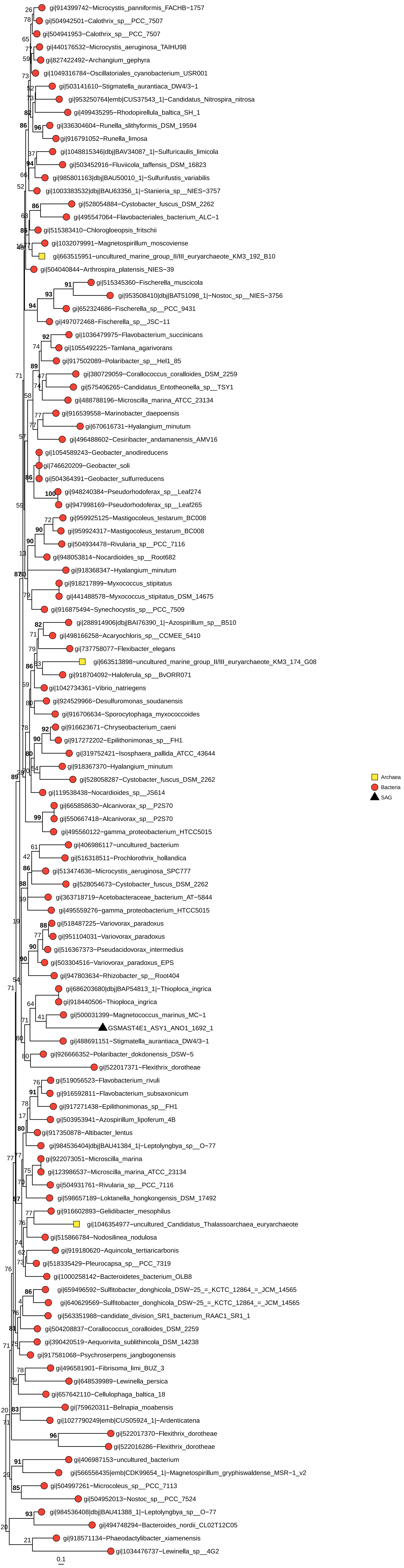
gi|914399742−Microcystis_panniformis_FACHB−1757
gi|504942501−Calothrix_sp__PCC_7507
gi|504941953−Calothrix_sp__PCC_7507
gi|440176532−Microcystis_aeruginosa_TAIHU98
gi|827422492−Archangium_gephyra
gi|1049316784−Oscillatoriales_cyanobacterium_USR001
gi|503141610−Stigmatella_aurantiaca_DW4/3−1
gi|953250764|emb|CUS37543_1|−Candidatus_Nitrospira_nitrosa
gi|499435295−Rhodopirellula_baltica_SH_1
gi|336304604−Runella_slithyformis_DSM_19594
gi|916791052−Runella_limosa
gi|1048815346|dbj|BAV34087_1|−Sulfuricaulis_limicola
gi|503452916−Fluviicola_taffensis_DSM_16823
gi|985801163|dbj|BAU50010_1|−Sulfurifustis_variabilis
gi|1003383532|dbj|BAU63356_1|−Stanieria_sp__NIES−3757
gi|528054884−Cystobacter_fuscus_DSM_2262
gi|495547064−Flavobacteriales_bacterium_ALC−1
gi|515383410−Chlorogloeopsis_fritschii
gi|1032079991−Magnetospirillum_moscoviense
gi|663515951−uncultured_marine_group_II/III_euryarchaeote_KM3_192_B10
gi|504040844−Arthrospira_platensis_NIES−39
gi|515345360−Fischerella_muscicola
gi|953508410|dbj|BAT51098_1|−Nostoc_sp__NIES−3756
gi|652324686−Fischerella_sp__PCC_9431
gi|497072468−Fischerella_sp__JSC−11
gi|1036479975−Flavobacterium_succinicans
gi|1055492225−Tamlana_agarivorans
gi|917502089−Polaribacter_sp__Hel1_85
gi|380729059−Corallococcus_coralloides_DSM_2259
gi|575416265−Candidatus_Entotheonella_sp__TSY1
gi|488788196−Microscilla_marina_ATCC_23134
gi|916539558−Marinobacter_daepoensis
gi|670616731−Hyalangium_minutum
gi|496488602−Cesiribacter_andamanensis_AMV16
gi|1054589243−Geobacter_anodireducens
gi|746620209−Geobacter_soli
gi|504364391−Geobacter_sulfurreducens
gi|948240384−Pseudorhodoferax_sp__Leaf274
gi|947998169−Pseudorhodoferax_sp__Leaf265
gi|959925125−Mastigocoleus_testarum_BC008
gi|959924317−Mastigocoleus_testarum_BC008
gi|504934478−Rivularia_sp__PCC_7116
gi|948053814−Nocardioides_sp__Root682
gi|918368347−Hyalangium_minutum
gi|918217899−Myxococcus_stipitatus
gi|441488578−Myxococcus_stipitatus_DSM_14675
gi|916875494−Synechocystis_sp__PCC_7509
gi|288914906|dbj|BAI76390_1|−Azospirillum_sp__B510
gi|498166258−Acaryochloris_sp__CCMEE_5410
gi|737758077−Flexibacter_elegans
gi|663513898−uncultured_marine_group_II/III_euryarchaeote_KM3_174_G08
gi|918704092−Haloferula_sp__BvORR071
gi|1042734361−Vibrio_natriegens
gi|924529966−Desulfuromonas_soudanensis
gi|916706634−Sporocytophaga_myxococcoides
gi|916623671−Chryseobacterium_caeni
gi|917272202−Epilithonimonas_sp__FH1
gi|319752421−Isosphaera_pallida_ATCC_43644
gi|918367370−Hyalangium_minutum
gi|528058287−Cystobacter_fuscus_DSM_2262
gi|119538438−Nocardioides_sp__JS614
gi|665858630−Alcanivorax_sp__P2S70
gi|550667418−Alcanivorax_sp__P2S70
gi|495560122−gamma_proteobacterium_HTCC5015
gi|406986117−uncultured_bacterium
gi|516318511−Prochlorothrix_hollandica
gi|513474636−Microcystis_aeruginosa_SPC777
gi|528054673−Cystobacter_fuscus_DSM_2262
gi|363718719−Acetobacteraceae_bacterium_AT−5844
gi|495559276−gamma_proteobacterium_HTCC5015
gi|518487225−Variovorax_paradoxus
gi|951104031−Variovorax_paradoxus
gi|516367373−Pseudacidovorax_intermedius
gi|503304516−Variovorax_paradoxus_EPS
gi|947803634−Rhizobacter_sp__Root404
gi|686203680|dbj|BAP54813_1|−Thioploca_ingrica
gi|918440506−Thioploca_ingrica
gi|500031399−Magnetococcus_marinus_MC−1
GSMAST4E1_ASY1_ANO1_1692_1
gi|488691151−Stigmatella_aurantiaca_DW4/3−1
gi|926666352−Polaribacter_dokdonensis_DSW−5
gi|522017371−Flexithrix_dorotheae
gi|519056523−Flavobacterium_rivuli
gi|916592811−Flavobacterium_subsaxonicum
gi|917271438−Epilithonimonas_sp__FH1
gi|503953941−Azospirillum_lipoferum_4B
gi|917350878−Altibacter_lentus
gi|984536404|dbj|BAU41384_1|−Leptolyngbya_sp__O−77
gi|922073051−Microscilla_marina
gi|123986537−Microscilla_marina_ATCC_23134
gi|504931761−Rivularia_sp__PCC_7116
gi|598657189−Loktanella_hongkongensis_DSM_17492
gi|916602893−Gelidibacter_mesophilus
gi|1046354977−uncultured_Candidatus_Thalassoarchaea_euryarchaeote
gi|515866784−Nodosilinea_nodulosa
gi|919180620−Aquincola_tertiaricarbonis
gi|518335429−Pleurocapsa_sp__PCC_7319
gi|1000258142−Bacteroidetes_bacterium_OLB8
gi|659496592−Sulfitobacter_donghicola_DSW−25_=_KCTC_12864_=_JCM_14565
gi|640629569−Sulfitobacter_donghicola_DSW−25_=_KCTC_12864_=_JCM_14565
gi|563351988−candidate_division_SR1_bacterium_RAAC1_SR1_1
gi|504208837−Corallococcus_coralloides_DSM_2259
gi|390420519−Aequorivita_sublithincola_DSM_14238
gi|917581068−Psychroserpens_jangbogonensis
gi|496581901−Fibrisoma_limi_BUZ_3
gi|648539989−Lewinella_persica
gi|657642110−Cellulophaga_baltica_18
gi|759620311−Belnapia_moabensis
gi|1027790249|emb|CUS05924_1|−Ardenticatena
gi|522017370−Flexithrix_dorotheae
gi|522016286−Flexithrix_dorotheae
gi|406987153−uncultured_bacterium
gi|566556435|emb|CDK99654_1|−Magnetospirillum_gryphiswaldense_MSR−1_v2
gi|504997261−Microcoleus_sp__PCC_7113
gi|504952013−Nostoc_sp__PCC_7524
gi|984536408|dbj|BAU41388_1|−Leptolyngbya_sp__O−77
gi|494748294−Bacteroides_nordii_CL02T12C05
gi|918571134−Phaeodactylibacter_xiamenensis
gi|1034476737−Lewinella_sp__4G2
0.1
Archaea
Bacteria
SAG

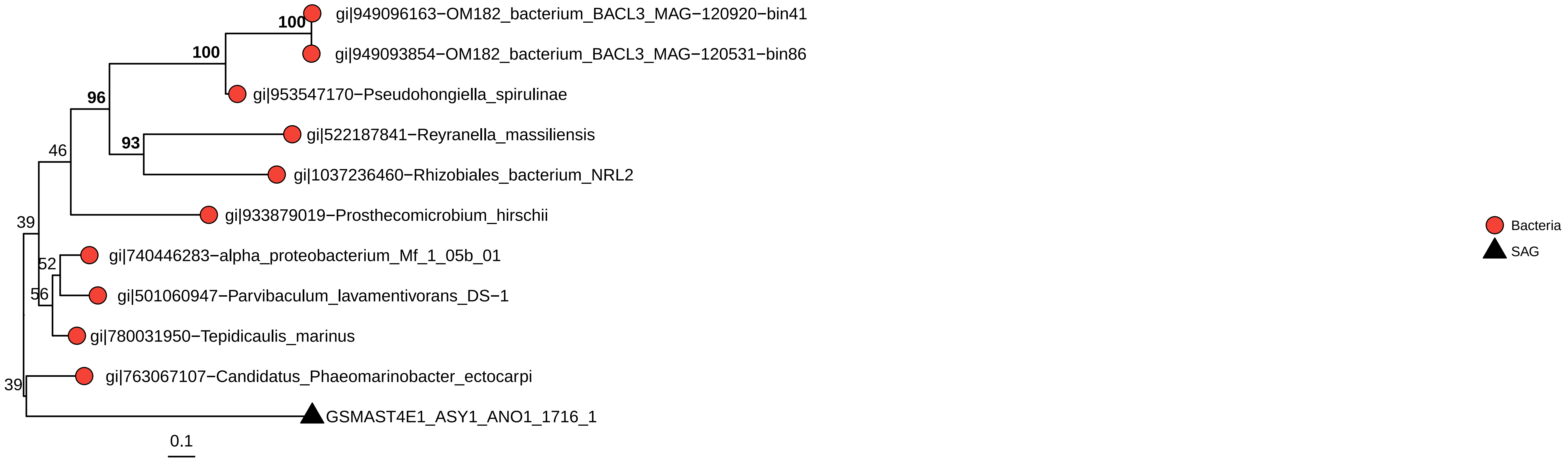

gi|949096163−OM182_bacterium_BACL3_MAG−120920−bin41
gi|949093854−OM182_bacterium_BACL3_MAG−120531−bin86
gi|953547170−Pseudohongiella_spirulinae
gi|522187841−Reyranella_massiliensis
gi|1037236460−Rhizobiales_bacterium_NRL2
gi|933879019−Prosthecomicrobium_hirschii
gi|740446283−alpha_proteobacterium_Mf_1_05b_01
gi|501060947−Parvibaculum_lavamentivorans_DS−1
gi|780031950−Tepidicaulis_marinus
gi|763067107−Candidatus_Phaeomarinobacter_ectocarpi
GSMAST4E1_ASY1_ANO1_1716_1
100
100
96
93
46
39
52
56
39
0.1
Bacteria
SAG

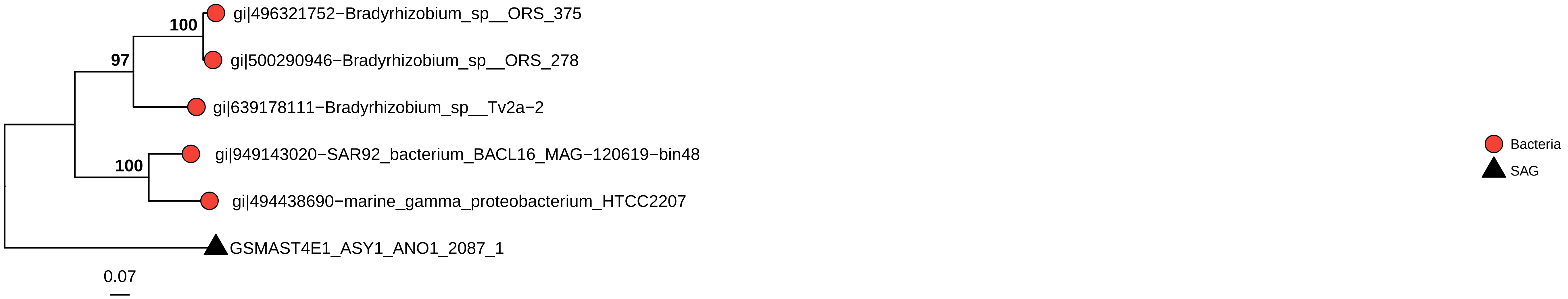
100
97
gi|496321752−Bradyrhizobium_sp__ORS_375
gi|500290946−Bradyrhizobium_sp__ORS_278
gi|639178111−Bradyrhizobium_sp__Tv2a−2
100
gi|949143020−SAR92_bacterium_BACL16_MAG−120619−bin48
gi|494438690−marine_gamma_proteobacterium_HTCC2207
GSMAST4E1_ASY1_ANO1_2087_1
0.07
Bacteria
SAG

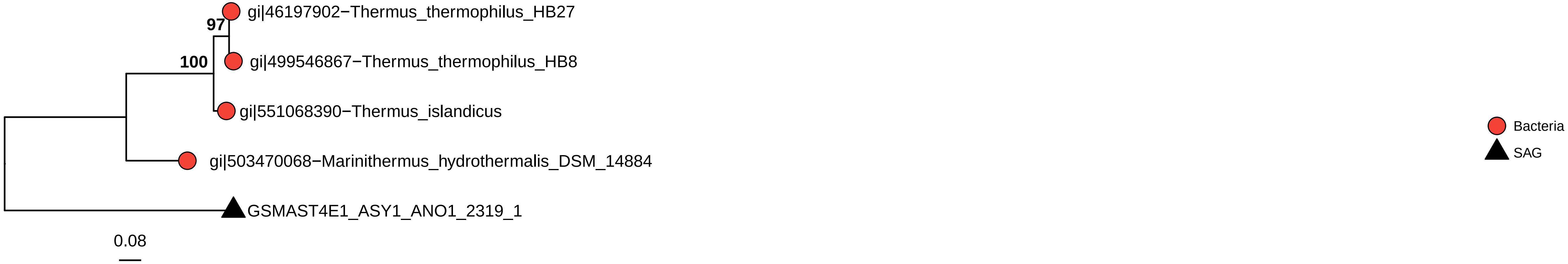
gi|46197902−Thermus_thermophilus_HB27
97
100
gi|499546867−Thermus_thermophilus_HB8
gi|551068390−Thermus_islandicus
gi|503470068−Marinithermus_hydrothermalis_DSM_14884
GSMAST4E1_ASY1_ANO1_2319_1
0.08
Bacteria
SAG

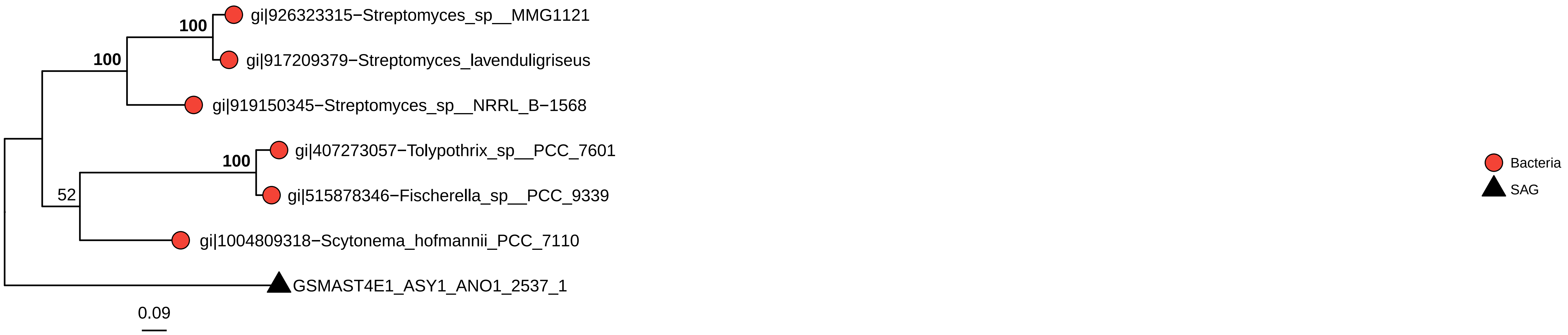
100
100
gi|926323315−Streptomyces_sp__MMG1121
gi|917209379−Streptomyces_lavenduligriseus
gi|919150345−Streptomyces_sp__NRRL_B−1568
100
gi|407273057−Tolypothrix_sp__PCC_7601
52
gi|515878346−Fischerella_sp__PCC_9339
gi|1004809318−Scytonema_hofmannii_PCC_7110
GSMAST4E1_ASY1_ANO1_2537_1
0.09
Bacteria
SAG

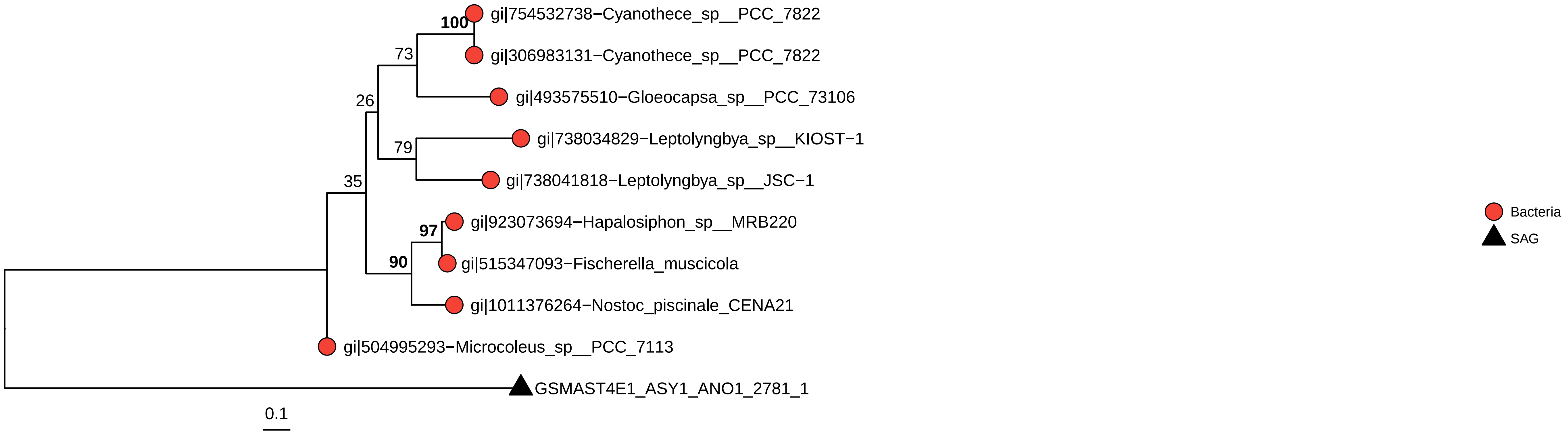

gi|754532738−Cyanothece_sp__PCC_7822
gi|306983131−Cyanothece_sp__PCC_7822
gi|493575510−Gloeocapsa_sp__PCC_73106
gi|738034829−Leptolyngbya_sp__KIOST−1
gi|738041818−Leptolyngbya_sp__JSC−1
gi|923073694−Hapalosiphon_sp__MRB220
gi|515347093−Fischerella_muscicola
gi|1011376264−Nostoc_piscinale_CENA21
gi|504995293−Microcoleus_sp__PCC_7113
GSMAST4E1_ASY1_ANO1_2781_1
100
73
26
79
35
97
90
0.1
Bacteria
SAG

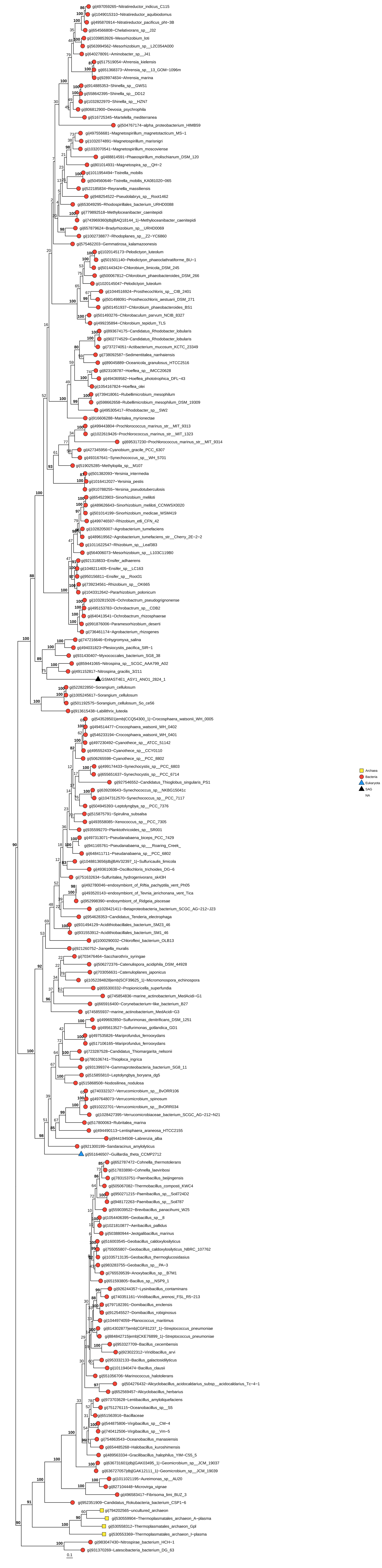
Archaea
Bacteria
Eukaryota
SAG
NA
0.1

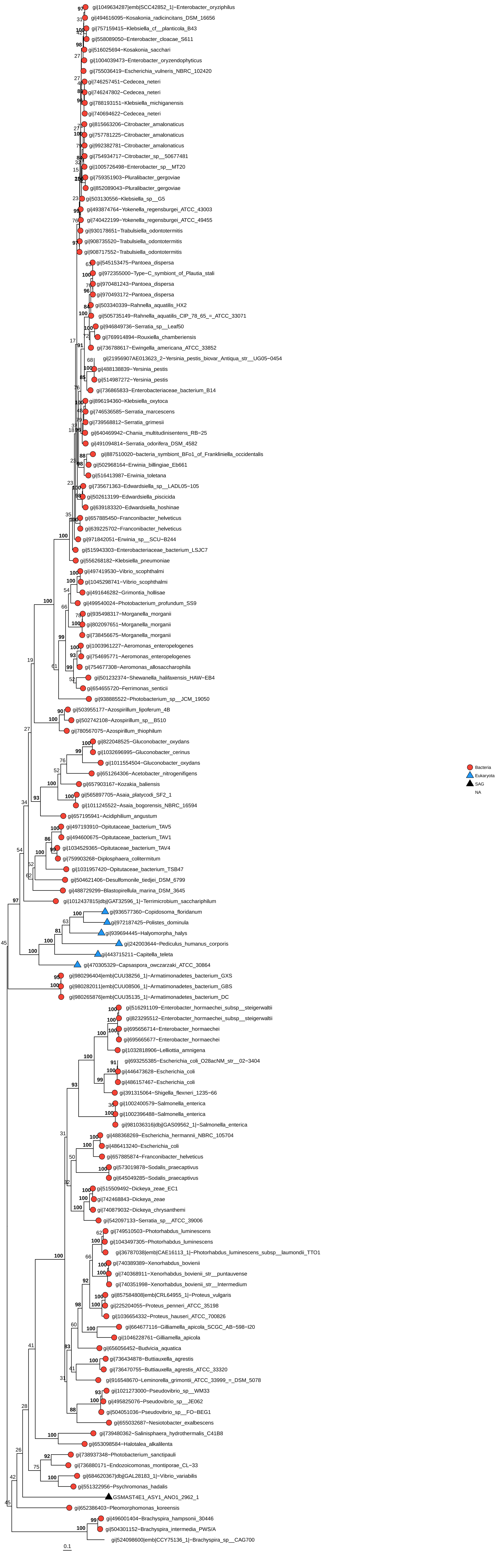
gi|1049634287|emb|SCC42852_1|~Enterobacter_oryziphilus
gi|494616095~Kosakonia_radicincitans_DSM_16656
gi|757159415~Klebsiella_cf__planticola_B43
gi|558089050~Enterobacter_cloacae_S611
gi|516025694~Kosakonia_sacchari
gi|1004039473~Enterobacter_oryzendophyticus
gi|755036419~Escherichia_vulneris_NBRC_102420
gi|746257451~Cedecea_neteri
gi|746247802~Cedecea_neteri
gi|788193151~Klebsiella_michiganensis
gi|740694622~Cedecea_neteri
gi|815663206~Citrobacter_amalonaticus
gi|757781225~Citrobacter_amalonaticus
gi|992382781~Citrobacter_amalonaticus
gi|754934717~Citrobacter_sp__50677481
gi|1005726498~Enterobacter_sp__MT20
gi|759351903~Pluralibacter_gergoviae
gi|852089043~Pluralibacter_gergoviae
gi|503130556~Klebsiella_sp__G5
gi|493874764~Yokenella_regensburgei_ATCC_43003
gi|740422199~Yokenella_regensburgei_ATCC_49455
gi|930178651~Trabulsiella_odontotermitis
gi|908735520~Trabulsiella_odontotermitis
gi|908717552~Trabulsiella_odontotermitis
gi|545153475~Pantoea_dispersa
gi|972355000~Type-C_symbiont_of_Plautia_stali
gi|970481243~Pantoea_dispersa
gi|970493172~Pantoea_dispersa
gi|503340339~Rahnella_aquatilis_HX2
gi|505735149~Rahnella_aquatilis_CIP_78_65_=_ATCC_33071
gi|946849736~Serratia_sp__Leaf50
gi|769914894~Rouxiella_chamberiensis
gi|736788617~Ewingella_americana_ATCC_33852
gi|21956907AE013623_2~Yersinia_pestis_biovar_Antiqua_str__UG05-0454
gi|488138839~Yersinia_pestis
gi|514987272~Yersinia_pestis
gi|736865833~Enterobacteriaceae_bacterium_B14
gi|896194360~Klebsiella_oxytoca
gi|746536585~Serratia_marcescens
gi|739568812~Serratia_grimesii
gi|640469942~Chania_multitudinisentens_RB-25
gi|491094814~Serratia_odorifera_DSM_4582
gi|887510020~bacteria_symbiont_BFo1_of_Frankliniella_occidentalis
gi|502968164~Erwinia_billingiae_Eb661
gi|516413987~Erwinia_toletana
gi|735671363~Edwardsiella_sp__LADL05-105
gi|502613199~Edwardsiella_piscicida
gi|639183320~Edwardsiella_hoshinae
gi|657885450~Franconibacter_helveticus
gi|639225702~Franconibacter_helveticus
gi|971842051~Erwinia_sp__SCU-B244
gi|515943303~Enterobacteriaceae_bacterium_LSJC7
gi|556268182~Klebsiella_pneumoniae
gi|497419530~Vibrio_scophthalmi
gi|1045298741~Vibrio_scophthalmi
gi|491646282~Grimontia_hollisae
gi|499540024~Photobacterium_profundum_SS9
gi|935498317~Morganella_morganii
gi|802097651~Morganella_morganii
gi|738456675~Morganella_morganii
gi|1003961227~Aeromonas_enteropelogenes
gi|754695771~Aeromonas_enteropelogenes
gi|754677308~Aeromonas_allosaccharophila
gi|501232374~Shewanella_halifaxensis_HAW-EB4
gi|654655720~Ferrimonas_senticii
gi|938885522~Photobacterium_sp__JCM_19050
gi|503955177~Azospirillum_lipoferum_4B
gi|502742108~Azospirillum_sp__B510
gi|780567075~Azospirillum_thiophilum
gi|822048525~Gluconobacter_oxydans
gi|1032696995~Gluconobacter_cerinus
gi|1011554504~Gluconobacter_oxydans
gi|651264306~Acetobacter_nitrogenifigens
gi|657903167~Kozakia_baliensis
gi|565897705~Asaia_platycodi_SF2_1
gi|1011245522~Asaia_bogorensis_NBRC_16594
gi|657195941~Acidiphilium_angustum
gi|497193910~Opitutaceae_bacterium_TAV5
gi|494600675~Opitutaceae_bacterium_TAV1
gi|1034529365~Opitutaceae_bacterium_TAV4
gi|759903268~Diplosphaera_colitermitum
gi|1031957420~Opitutaceae_bacterium_TSB47
gi|504621406~Desulfomonile_tiedjei_DSM_6799
gi|488729299~Blastopirellula_marina_DSM_3645
gi|1012437815|dbj|GAT32596_1|~Terrimicrobium_sacchariphilum
gi|936577360~Copidosoma_floridanum
gi|972187425~Polistes_dominula
gi|939694445~Halyomorpha_halys
gi|242003644~Pediculus_humanus_corporis
gi|443715211~Capitella_teleta
gi|470305329~Capsaspora_owczarzaki_ATCC_30864
gi|980296404|emb|CUU38256_1|~Armatimonadetes_bacterium_GXS
gi|980282011|emb|CUU08506_1|~Armatimonadetes_bacterium_GBS
gi|980265876|emb|CUU35135_1|~Armatimonadetes_bacterium_DC
gi|516291109~Enterobacter_hormaechei_subsp__steigerwaltii
gi|823295512~Enterobacter_hormaechei_subsp__steigerwaltii
gi|695656714~Enterobacter_hormaechei
gi|695665677~Enterobacter_hormaechei
gi|1032818906~Lelliottia_amnigena
gi|693255385~Escherichia_coli_O28acNM_str__02-3404
gi|446473628~Escherichia_coli
gi|486157467~Escherichia_coli
gi|391315064~Shigella_flexneri_1235-66
gi|1002400579~Salmonella_enterica
gi|1002396488~Salmonella_enterica
gi|981036316|dbj|GAS09562_1|~Salmonella_enterica
gi|488368269~Escherichia_hermannii_NBRC_105704
gi|486413240~Escherichia_coli
gi|657885874~Franconibacter_helveticus
gi|573019878~Sodalis_praecaptivus
gi|645049285~Sodalis_praecaptivus
gi|515509492~Dickeya_zeae_EC1
gi|742468843~Dickeya_zeae
gi|740879032~Dickeya_chrysanthemi
gi|542097133~Serratia_sp__ATCC_39006
gi|749510503~Photorhabdus_luminescens
gi|1043497305~Photorhabdus_luminescens
gi|36787038|emb|CAE16113_1|~Photorhabdus_luminescens_subsp__laumondii_TTO1
gi|740389389~Xenorhabdus_bovienii
gi|740368911~Xenorhabdus_bovienii_str__puntauvense
gi|740351998~Xenorhabdus_bovienii_str__Intermedium
gi|857584808|emb|CRL64955_1|~Proteus_vulgaris
gi|225204055~Proteus_penneri_ATCC_35198
gi|1036654332~Proteus_hauseri_ATCC_700826
gi|664677116~Gilliamella_apicola_SCGC_AB-598-I20
gi|1046228761~Gilliamella_apicola
gi|656056452~Budvicia_aquatica
gi|736434878~Buttiauxella_agrestis
gi|736470755~Buttiauxella_agrestis_ATCC_33320
gi|916548670~Leminorella_grimontii_ATCC_33999_=_DSM_5078
gi|1021273000~Pseudovibrio_sp__WM33
gi|495825076~Pseudovibrio_sp__JE062
gi|504051036~Pseudovibrio_sp__FO-BEG1
gi|655032687~Nesiotobacter_exalbescens
gi|739480362~Salinisphaera_hydrothermalis_C41B8
gi|653098584~Halotalea_alkalilenta
gi|738937348~Photobacterium_sanctipauli
gi|736880171~Endozoicomonas_montiporae_CL-33
gi|684620367|dbj|GAL28183_1|~Vibrio_variabilis
gi|551322956~Psychromonas_hadalis
GSMAST4E1_ASY1_ANO1_2962_1
gi|652386403~Pleomorphomonas_koreensis
gi|496001404~Brachyspira_hampsonii_30446
gi|504301152~Brachyspira_intermedia_PWS/A
gi|524098600|emb|CCY75136_1|~Brachyspira_sp__CAG700
0.1
Bacteria
Eukaryota
SAG
NA

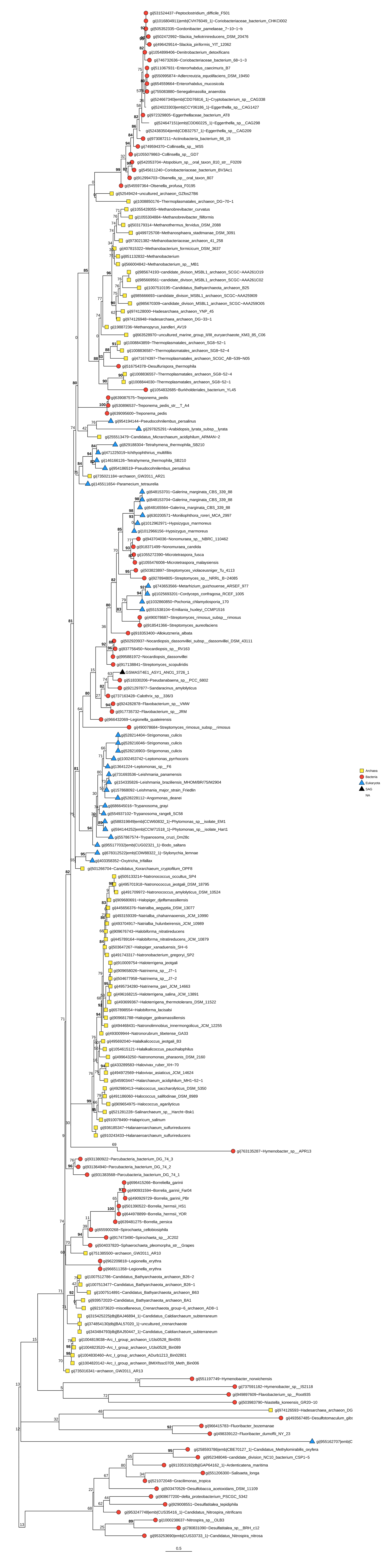

Archaea
Bacteria
Eukaryota
SAG
NA
0.5

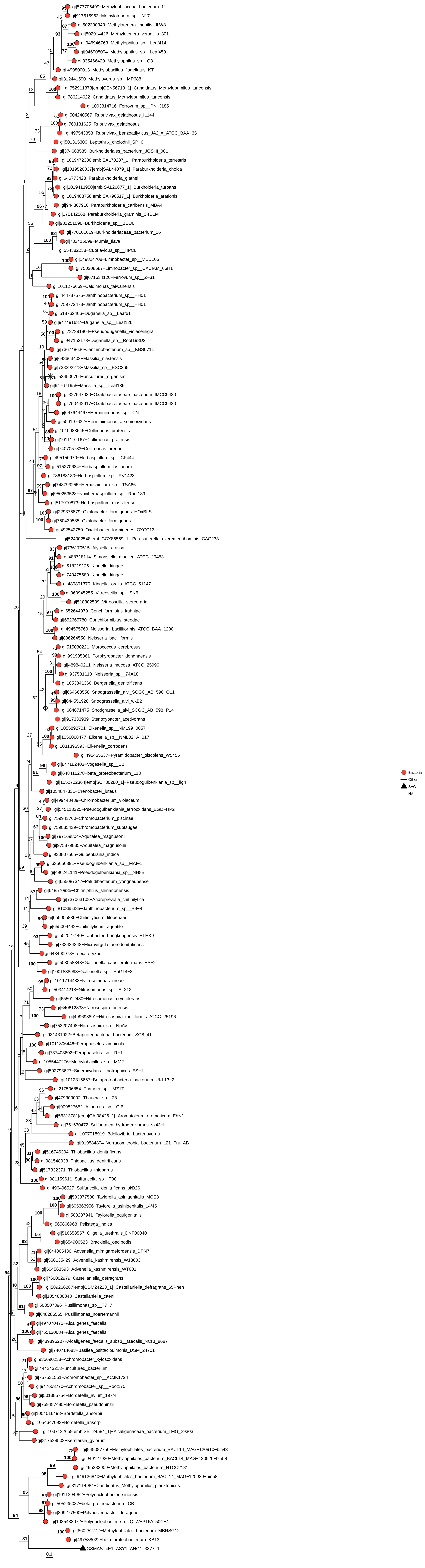
gi|577705499~Methylophilaceae_bacterium_11
gi|917615963~Methylotenera_sp__N17
gi|502390343~Methylotenera_mobilis_JLW8
gi|502914426~Methylotenera_versatilis_301
gi|946946763~Methylophilus_sp__Leaf414
gi|946908094~Methylophilus_sp__Leaf459
gi|835466429~Methylophilus_sp__Q8
gi|499800013~Methylobacillus_flagellatus_KT
gi|312441590~Methylovorus_sp__MP688
gi|752911878|emb|CEN56713_1|~Candidatus_Methylopumilus_turicensis
gi|786214622~Candidatus_Methylopumilus_turicensis
gi|1003314716~Ferrovum_sp__PN~J185
gi|504240567~Rubrivivax_gelatinosus_IL144
gi|760131625~Rubrivivax_gelatinosus
gi|497543853~Rubrivivax_benzoatilyticus_JA2_=_ATCC_BAA~35
gi|501315306~Leptothrix_cholodnii_SP~6
gi|374668535~Burkholderiales_bacterium_JOSHI_001
gi|1019472380|emb|SAL70287_1|~Paraburkholderia_terrestris
gi|1019520037|emb|SAL44079_1|~Paraburkholderia_choica
gi|646773428~Paraburkholderia_glathei
gi|1019413950|emb|SAL26877_1|~Burkholderia_turbans
gi|1019488758|emb|SAK96517_1|~Burkholderia_arationis
gi|944367916~Paraburkholderia_caribensis_MBA4
gi|170142568~Paraburkholderia_graminis_C4D1M
gi|981251096~Burkholderia_sp__BDU6
gi|770101619~Burkholderiaceae_bacterium_16
gi|733416099~Mumia_flava
gi|554382238~Cupriavidus_sp__HPCL
gi|149824708~Limnobacter_sp__MED105
gi|750208687~Limnobacter_sp__CACIAM_66H1
gi|671634120~Ferrovum_sp__Z~31
gi|1011276669~Caldimonas_taiwanensis
gi|444787575~Janthinobacterium_sp__HH01
gi|759772473~Janthinobacterium_sp__HH01
gi|518762406~Duganella_sp__Leaf61
gi|947491687~Duganella_sp__Leaf126
gi|737391804~Pseudoduganella_violaceinigra
gi|947152173~Duganella_sp__Root198D2
gi|736748636~Janthinobacterium_sp__KBS0711
gi|648663403~Massilia_niastensis
gi|738292278~Massilia_sp__BSC265
gi|534500704~uncultured_organism
gi|947671958~Massilia_sp__Leaf139
gi|327547030~Oxalobacteraceae_bacterium_IMCC9480
gi|750442917~Oxalobacteraceae_bacterium_IMCC9480
gi|647644467~Herminiimonas_sp__CN
gi|500197632~Herminiimonas_arsenicoxydans
gi|1010983645~Collimonas_pratensis
gi|1011197167~Collimonas_pratensis
gi|740705783~Collimonas_arenae
gi|495150970~Herbaspirillum_sp__CF444
gi|515270684~Herbaspirillum_lusitanum
gi|736183130~Herbaspirillum_sp__RV1423
gi|748793255~Herbaspirillum_sp__TSA66
gi|950253528~Noviherbaspirillum_sp__Root189
gi|517970873~Herbaspirillum_massiliense
gi|229376879~Oxalobacter_formigenes_HOxBLS
gi|750439585~Oxalobacter_formigenes
gi|492542750~Oxalobacter_formigenes_OXCC13
gi|524002548|emb|CCX86569_1|~Parasutterella_excrementihominis_CAG233
gi|736170515~Alysiella_crassa
gi|488718114~Simonsiella_muelleri_ATCC_29453
gi|518219126~Kingella_kingae
gi|740475680~Kingella_kingae
gi|489891370~Kingella_oralis_ATCC_51147
gi|960945255~Vitreoscilla_sp__SN6
gi|518802539~Vitreoscilla_stercoraria
gi|652644079~Conchiformibius_kuhniae
gi|652665780~Conchiformibius_steedae
gi|494575769~Neisseria_bacilliformis_ATCC_BAA~1200
gi|896264550~Neisseria_bacilliformis
gi|515030221~Morococcus_cerebrosus
gi|991985361~Porphyrobacter_donghaensis
gi|489840211~Neisseria_mucosa_ATCC_25996
gi|937531110~Neisseria_sp__74A18
gi|1053841360~Bergeriella_denitrificans
gi|664668558~Snodgrassella_alvi_SCGC_AB~598~O11
gi|644551928~Snodgrassella_alvi_wkB2
gi|664671475~Snodgrassella_alvi_SCGC_AB~598~P14
gi|917333939~Stenoxybacter_acetivorans
gi|1055892701~Eikenella_sp__NML99~0057
gi|1056068477~Eikenella_sp__NML02~A~017
gi|1031396593~Eikenella_corrodens
gi|496455537~Pyramidobacter_piscolens_W5455
gi|847182403~Vogesella_sp__EB
gi|648416278~beta_proteobacterium_L13
gi|1052702364|emb|SCK30280_1|~Pseudogulbenkiania_sp__lig4
gi|1054847331~Crenobacter_luteus
gi|499448489~Chromobacterium_violaceum
gi|545113325~Pseudogulbenkiania_ferrooxidans_EGD~HP2
gi|759943760~Chromobacterium_piscinae
gi|759885439~Chromobacterium_subtsugae
gi|797169804~Aquitalea_magnusonii
gi|975879835~Aquitalea_magnusonii
gi|930807565~Gulbenkiania_indica
gi|635656391~Pseudogulbenkiania_sp__MAI~1
gi|496241141~Pseudogulbenkiania_sp__NH8B
gi|655087347~Paludibacterium_yongneupense
gi|648570985~Chitiniphilus_shinanonensis
gi|737063108~Andreprevotia_chitinilytica
gi|810865385~Janthinobacterium_sp__B9~8
gi|655005836~Chitinilyticum_litopenaei
gi|655004442~Chitinilyticum_aquatile
gi|502027440~Laribacter_hongkongensis_HLHK9
gi|738434848~Microvirgula_aerodenitrificans
gi|648490978~Leeia_oryzae
gi|503058843~Gallionella_capsiferriformans_ES~2
gi|1001838993~Gallionella_sp__ShG14~8
gi|1011714488~Nitrosomonas_ureae
gi|503414218~Nitrosomonas_sp__AL212
gi|655012430~Nitrosomonas_cryotolerans
gi|640612838~Nitrosospira_briensis
gi|499698891~Nitrosospira_multiformis_ATCC_25196
gi|753207498~Nitrosospira_sp__NpAV
gi|931431922~Betaproteobacteria_bacterium_SG8_41
gi|1011806446~Ferriphaselus_amnicola
gi|737403602~Ferriphaselus_sp__R~1
gi|1055447276~Methylobacillus_sp__MM2
gi|502793627~Sideroxydans_lithotrophicus_ES~1
gi|1012315667~Betaproteobacteria_bacterium_UKL13~2
gi|217506854~Thauera_sp__MZ1T
gi|479303002~Thauera_sp__28
gi|909827652~Azoarcus_sp__CIB
gi|56313781|emb|CAI08426_1|~Aromatoleum_aromaticum_EbN1
gi|751630472~Sulfuritalea_hydrogenivorans_sk43H
gi|1007018919~Bdellovibrio_bacteriovorus
gi|919584804~Verrucomicrobia_bacterium_L21~Fru~AB
gi|516746304~Thiobacillus_denitrificans
gi|981548038~Thiobacillus_denitrificans
gi|517332371~Thiobacillus_thioparus
gi|981159611~Sulfuricella_sp__T08
gi|496496527~Sulfuricella_denitrificans_skB26
gi|503877508~Taylorella_asinigenitalis_MCE3
gi|505363956~Taylorella_asinigenitalis_14/45
gi|503287941~Taylorella_equigenitalis
gi|565866968~Pelistega_indica
gi|516658557~Oligella_urethralis_DNF00040
gi|654906523~Brackiella_oedipodis
gi|644865436~Advenella_mimigardefordensis_DPN7
gi|566135429~Advenella_kashmirensis_W13003
gi|504563593~Advenella_kashmirensis_WT001
gi|760002979~Castellaniella_defragrans
gi|589266287|emb|CDM24223_1|~Castellaniella_defragrans_65Phen
gi|1054686848~Castellaniella_caeni
gi|503507396~Pusillimonas_sp__T7~7
gi|648286565~Pusillimonas_noertemannii
gi|497070472~Alcaligenes_faecalis
gi|755130684~Alcaligenes_faecalis
gi|489896207~Alcaligenes_faecalis_subsp__faecalis_NCIB_8687
gi|740714683~Basilea_psittacipulmonis_DSM_24701
gi|935690238~Achromobacter_xylosoxidans
gi|444243213~uncultured_bacterium
gi|757531551~Achromobacter_sp__KCJK1724
gi|947653770~Achromobacter_sp__Root170
gi|501385754~Bordetella_avium_197N
gi|759487485~Bordetella_pseudohinzii
gi|1054016498~Bordetella_ansorpii
gi|1054647093~Bordetella_ansorpii
gi|1037122659|emb|SBT24584_1|~Alcaligenaceae_bacterium_LMG_29303
gi|817528503~Kerstersia_gyiorum
gi|949087756~Methylophilales_bacterium_BACL14_MAG~120910~bin43
gi|949127920~Methylophilales_bacterium_BACL14_MAG~120920~bin58
gi|495382909~Methylophilales_bacterium_HTCC2181
gi|949126840~Methylophilales_bacterium_BACL14_MAG~120920~bin58
gi|817114984~Candidatus_Methylopumilus_planktonicus
gi|1011394952~Polynucleobacter_sinensis
gi|505235087~beta_proteobacterium_CB
gi|809277500~Polynucleobacter_duraquae
gi|1035438072~Polynucleobacter_sp__QLW~P1FAT50C~4
gi|860252747~Methylophilales_bacterium_MBRSG12
gi|497538022~beta_proteobacterium_KB13
GSMAST4E1_ASY1_ANO1_3877_1
0.1
Bacteria
Other
SAG
NA

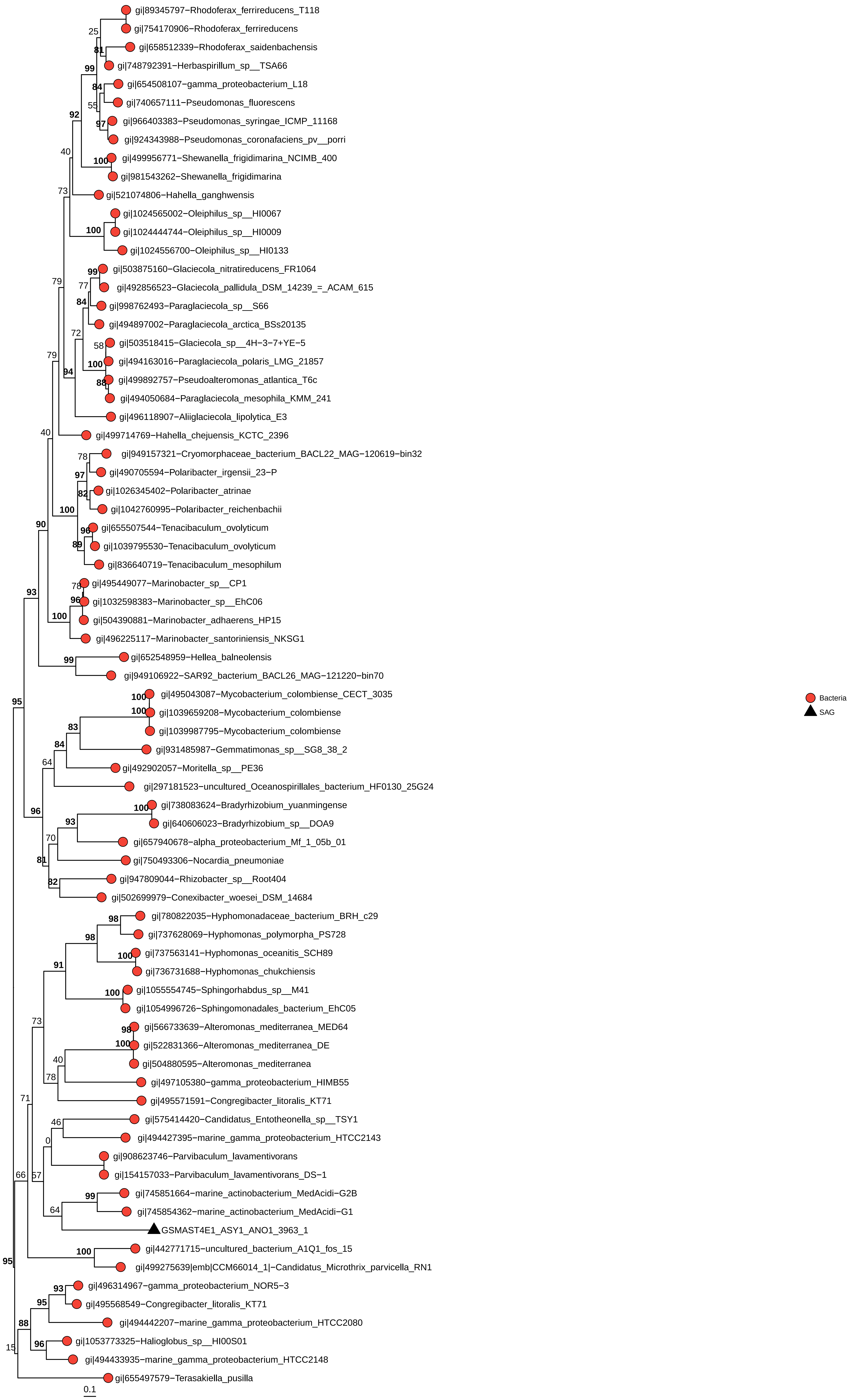
gi|89345797−Rhodoferax_ferrireducens_T118
gi|754170906−Rhodoferax_ferrireducens
gi|658512339−Rhodoferax_saidenbachensis
gi|748792391−Herbaspirillum_sp__TSA66
gi|654508107−gamma_proteobacterium_L18
gi|740657111−Pseudomonas_fluorescens
gi|966403383−Pseudomonas_syringae_ICMP_11168
gi|924343988−Pseudomonas_coronafaciens_pv__porri
gi|499956771−Shewanella_frigidimarina_NCIMB_400
gi|981543262−Shewanella_frigidimarina
gi|521074806−Hahella_ganghwensis
gi|1024565002−Oleiphilus_sp__HI0067
gi|1024444744−Oleiphilus_sp__HI0009
gi|1024556700−Oleiphilus_sp__HI0133
gi|503875160−Glaciecola_nitratireducens_FR1064
gi|492856523−Glaciecola_pallidula_DSM_14239_=_ACAM_615
gi|998762493−Paraglaciecola_sp__S66
gi|494897002−Paraglaciecola_arctica_BSs20135
gi|503518415−Glaciecola_sp__4H−3−7+YE−5
gi|494163016−Paraglaciecola_polaris_LMG_21857
gi|499892757−Pseudoalteromonas_atlantica_T6c
gi|494050684−Paraglaciecola_mesophila_KMM_241
gi|496118907−Aliiglaciecola_lipolytica_E3
gi|499714769−Hahella_chejuensis_KCTC_2396
gi|949157321−Cryomorphaceae_bacterium_BACL22_MAG−120619−bin32
gi|490705594−Polaribacter_irgensii_23−P
gi|1026345402−Polaribacter_atrinae
gi|1042760995−Polaribacter_reichenbachii
gi|655507544−Tenacibaculum_ovolyticum
gi|1039795530−Tenacibaculum_ovolyticum
gi|836640719−Tenacibaculum_mesophilum
gi|495449077−Marinobacter_sp__CP1
gi|1032598383−Marinobacter_sp__EhC06
gi|504390881−Marinobacter_adhaerens_HP15
gi|496225117−Marinobacter_santoriniensis_NKSG1
gi|652548959−Hellea_balneolensis
gi|949106922−SAR92_bacterium_BACL26_MAG−121220−bin70
gi|495043087−Mycobacterium_colombiense_CECT_3035
gi|1039659208−Mycobacterium_colombiense
gi|1039987795−Mycobacterium_colombiense
gi|931485987−Gemmatimonas_sp__SG8_38_2
gi|492902057−Moritella_sp__PE36
gi|297181523−uncultured_Oceanospirillales_bacterium_HF0130_25G24
gi|738083624−Bradyrhizobium_yuanmingense
gi|640606023−Bradyrhizobium_sp__DOA9
gi|657940678−alpha_proteobacterium_Mf_1_05b_01
gi|750493306−Nocardia_pneumoniae
gi|947809044−Rhizobacter_sp__Root404
gi|502699979−Conexibacter_woesei_DSM_14684
gi|780822035−Hyphomonadaceae_bacterium_BRH_c29
gi|737628069−Hyphomonas_polymorpha_PS728
gi|737563141−Hyphomonas_oceanitis_SCH89
gi|736731688−Hyphomonas_chukchiensis
gi|1055554745−Sphingorhabdus_sp__M41
gi|1054996726−Sphingomonadales_bacterium_EhC05
gi|566733639−Alteromonas_mediterranea_MED64
gi|522831366−Alteromonas_mediterranea_DE
gi|504880595−Alteromonas_mediterranea
gi|497105380−gamma_proteobacterium_HIMB55
gi|495571591−Congregibacter_litoralis_KT71
gi|575414420−Candidatus_Entotheonella_sp__TSY1
gi|494427395−marine_gamma_proteobacterium_HTCC2143
gi|908623746−Parvibaculum_lavamentivorans
gi|154157033−Parvibaculum_lavamentivorans_DS−1
gi|745851664−marine_actinobacterium_MedAcidi−G2B
gi|745854362−marine_actinobacterium_MedAcidi−G1
GSMAST4E1_ASY1_ANO1_3963_1
gi|442771715−uncultured_bacterium_A1Q1_fos_15
gi|499275639|emb|CCM66014_1|−Candidatus_Microthrix_parvicella_RN1
gi|496314967−gamma_proteobacterium_NOR5−3
gi|495568549−Congregibacter_litoralis_KT71
gi|494442207−marine_gamma_proteobacterium_HTCC2080
gi|1053773325−Halioglobus_sp__HI00S01
gi|494433935−marine_gamma_proteobacterium_HTCC2148
gi|655497579−Terasakiella_pusilla
0.1
Bacteria
SAG

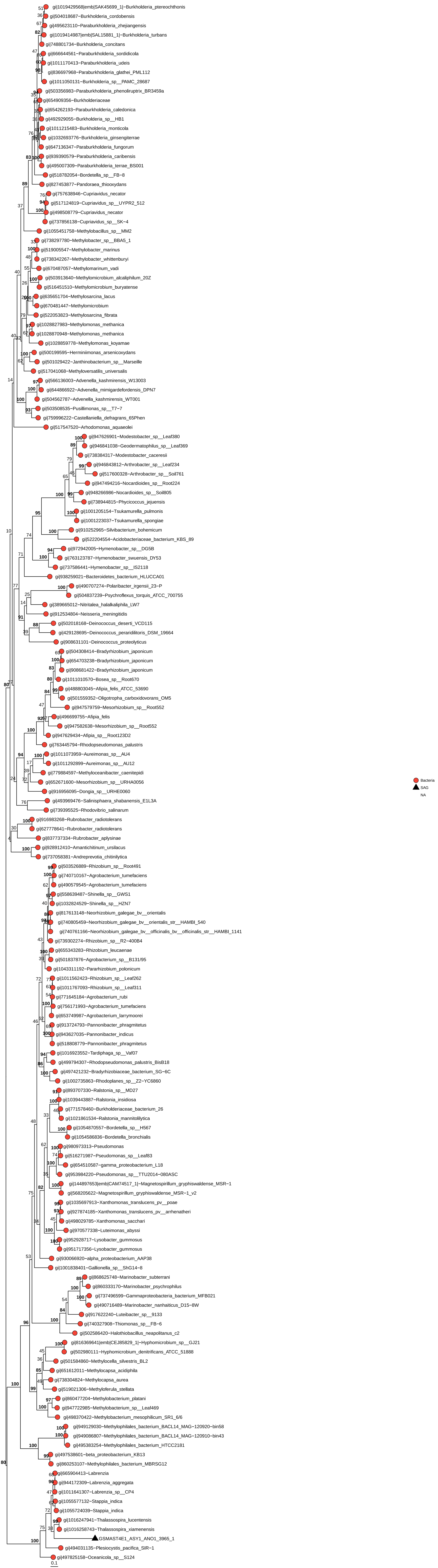
Bacteria
SAG
NA
0.1

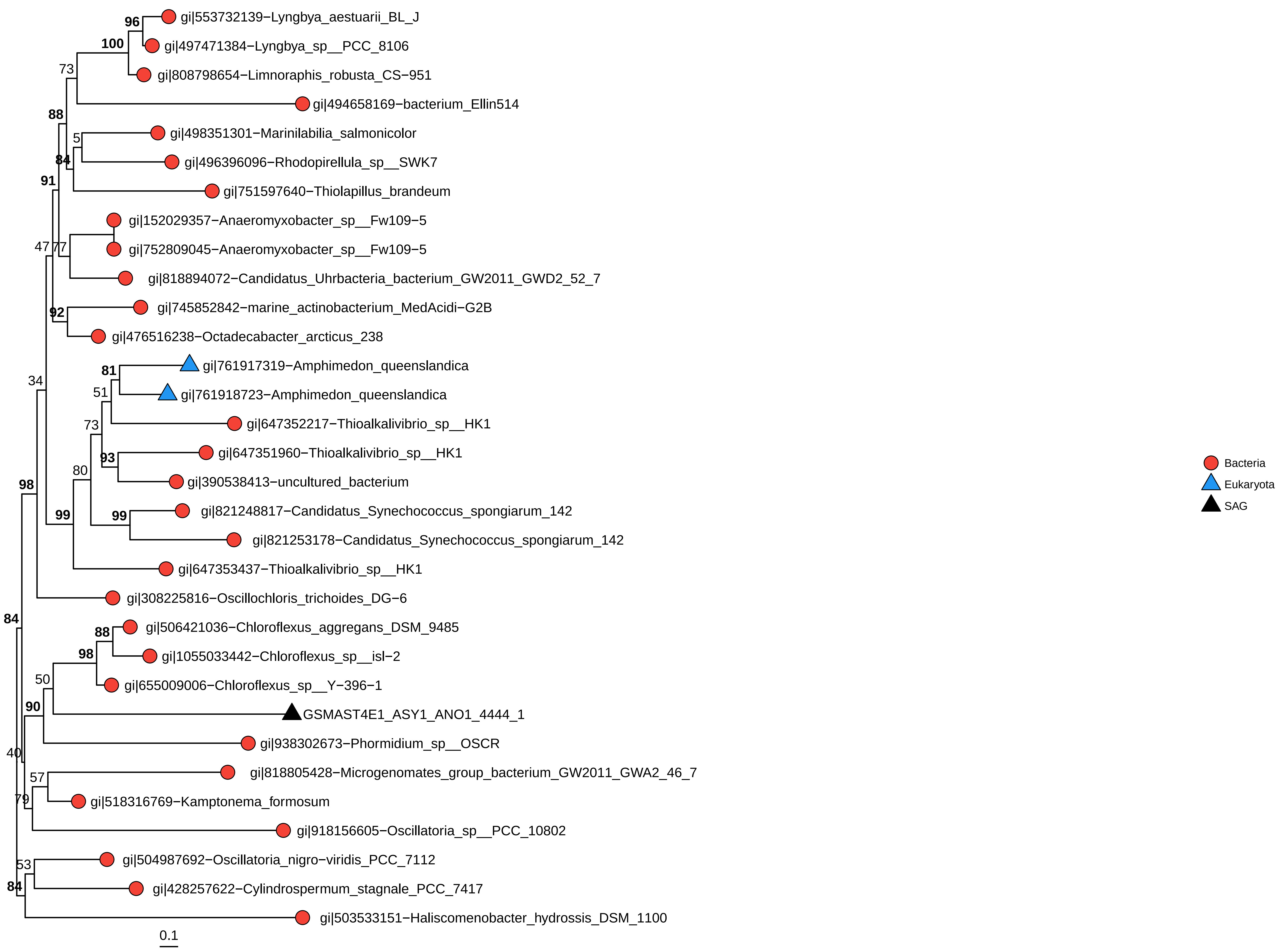
96
100
73
88
5
84
91
47
77
92
81
51
73
93
80
99
99
34
98
84
88
98
50
90
40
57
79
53
84
gi|553732139−Lyngbya_aestuarii_BL_J
gi|497471384−Lyngbya_sp__PCC_8106
gi|808798654−Limnoraphis_robusta_CS−951
gi|494658169−bacterium_Ellin514
gi|498351301−Marinilabilia_salmonicolor
gi|496396096−Rhodopirellula_sp__SWK7
gi|751597640−Thiolapillus_brandeum
gi|152029357−Anaeromyxobacter_sp__Fw109−5
gi|752809045−Anaeromyxobacter_sp__Fw109−5
gi|818894072−Candidatus_Uhrbacteria_bacterium_GW2011_GWD2_52_7
gi|745852842−marine_actinobacterium_MedAcidi−G2B
gi|476516238−Octadecabacter_arcticus_238
gi|761917319−Amphimedon_queenslandica
gi|761918723−Amphimedon_queenslandica
gi|647352217−Thioalkalivibrio_sp__HK1
gi|647351960−Thioalkalivibrio_sp__HK1
gi|390538413−uncultured_bacterium
gi|821248817−Candidatus_Synechococcus_spongiarum_142
gi|821253178−Candidatus_Synechococcus_spongiarum_142
gi|647353437−Thioalkalivibrio_sp__HK1
gi|308225816−Oscillochloris_trichoides_DG−6
gi|506421036−Chloroflexus_aggregans_DSM_9485
gi|1055033442−Chloroflexus_sp__isl−2
gi|655009006−Chloroflexus_sp__Y−396−1
GSMAST4E1_ASY1_ANO1_4444_1
gi|938302673−Phormidium_sp__OSCR
gi|818805428−Microgenomates_group_bacterium_GW2011_GWA2_46_7
gi|518316769−Kamptonema_formosum
gi|918156605−Oscillatoria_sp__PCC_10802
gi|504987692−Oscillatoria_nigro−viridis_PCC_7112
gi|428257622−Cylindrospermum_stagnale_PCC_7417
gi|503533151−Haliscomenobacter_hydrossis_DSM_1100
0.1
Bacteria
Eukaryota
SAG

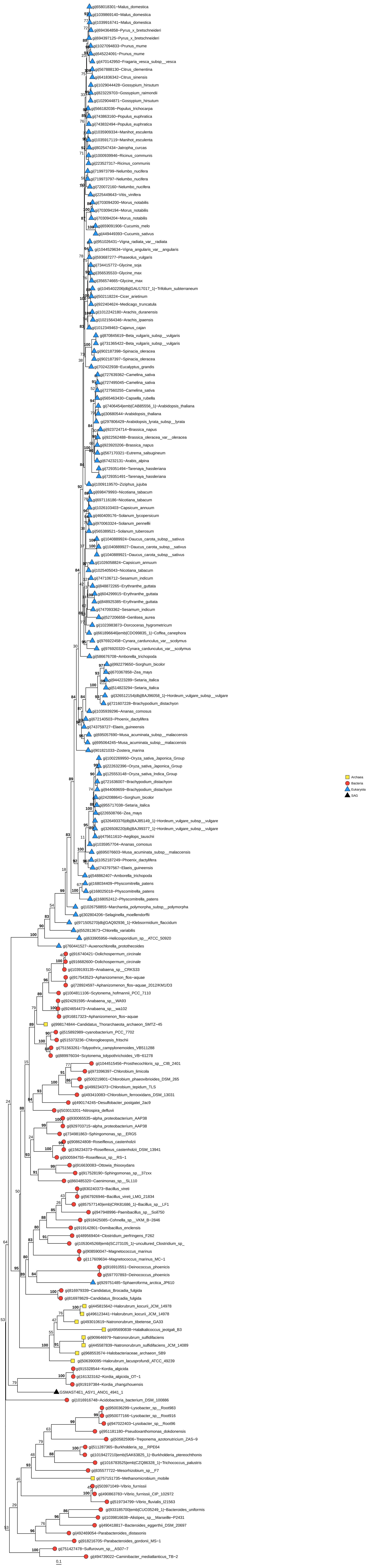

Archaea
Bacteria
Eukaryota
SAG
0.1

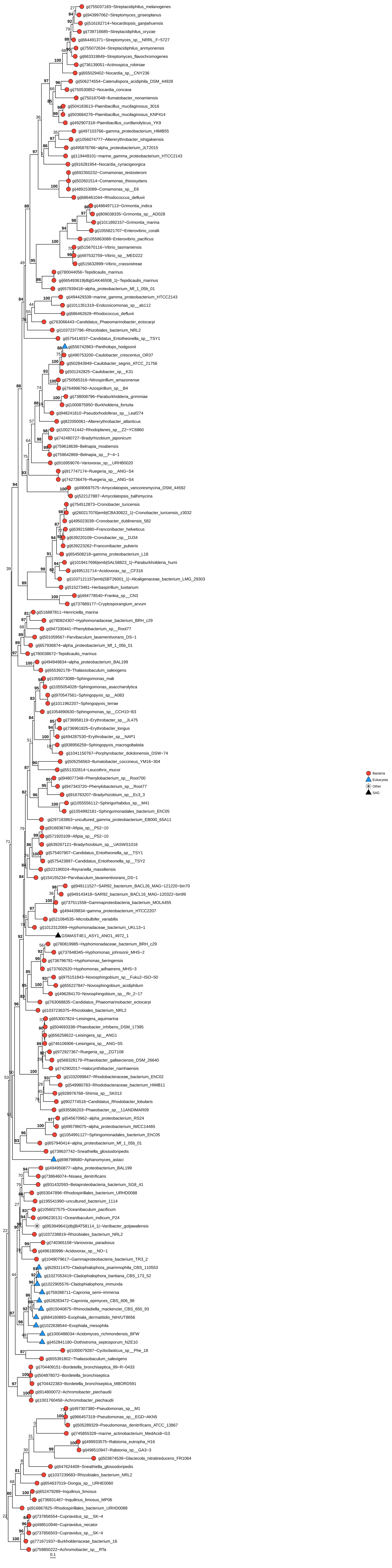
gi|755037183~Streptacidiphilus_melanogenes
gi|943997062~Streptomyces_griseoplanus
gi|516162714~Nocardiopsis_ganjiahuensis
gi|739716685~Streptacidiphilus_oryzae
gi|664491371~Streptomyces_sp__NRRL_F-5727
gi|755072634~Streptacidiphilus_anmyonensis
gi|663319849~Streptomyces_flavochromogenes
gi|736139051~Actinospica_robiniae
gi|655029402~Nocardia_sp__CNY236
gi|506274554~Catenulispora_acidiphila_DSM_44928
gi|750530852~Nocardia_concava
gi|750187048~Ilumatobacter_nonamiensis
gi|504183613~Paenibacillus_mucilaginosus_3016
gi|503684276~Paenibacillus_mucilaginosus_KNP414
gi|492907318~Paenibacillus_curdlanolyticus_YK9
gi|497103766~gamma_proteobacterium_HIMB55
gi|1056074777~Altererythrobacter_ishigakiensis
gi|495878766~alpha_proteobacterium_JLT2015
gi|119449101~marine_gamma_proteobacterium_HTCC2143
gi|916281954~Nocardia_cyriacigeorgica
gi|692350232~Comamonas_testosteroni
gi|502601514~Comamonas_thiooxydans
gi|489153089~Comamonas_sp__E6
gi|686461044~Rhodococcus_defluvii
gi|488497113~Grimontia_indica
gi|809038335~Grimontia_sp__AD028
gi|1011892157~Grimontia_marina
gi|1055821707~Enterovibrio_coralii
gi|1055863088~Enterovibrio_pacificus
gi|515670116~Vibrio_tasmaniensis
gi|497532759~Vibrio_sp__MED222
gi|515632899~Vibrio_crassostreae
gi|780044056~Tepidicaulis_marinus
gi|665493619|dbj|GAK46508_1|~Tepidicaulis_marinus
gi|657939418~alpha_proteobacterium_Mf_1_05b_01
gi|494429339~marine_gamma_proteobacterium_HTCC2143
gi|1011351319~Endozoicomonas_sp__ab112
gi|686462628~Rhodococcus_defluvii
gi|763066443~Candidatus_Phaeomarinobacter_ectocarpi
gi|1037237796~Rhizobiales_bacterium_NRL2
gi|575414037~Candidatus_Entotheonella_sp__TSY1
gi|556742863~Pantholops_hodgsonii
gi|490753200~Caulobacter_crescentus_OR37
gi|502843949~Caulobacter_segnis_ATCC_21756
gi|501242825~Caulobacter_sp__K31
gi|750565316~Nitrospirillum_amazonense
gi|764996760~Azospirillum_sp__B4
gi|738008796~Paraburkholderia_grimmiae
gi|1000875950~Burkholderia_fortuita
gi|948241810~Pseudorhodoferax_sp__Leaf274
gi|822050061~Altererythrobacter_atlanticus
gi|1002741442~Rhodoplanes_sp__Z2-YC6860
gi|742480727~Bradyrhizobium_japonicum
gi|759618639~Belnapia_moabensis
gi|759642869~Belnapia_sp__F-4-1
gi|916959076~Variovorax_sp__URHB0020
gi|917747174~Ruegeria_sp__ANG-S4
gi|742736476~Ruegeria_sp__ANG-S4
gi|490697575~Amycolatopsis_vancoresmycina_DSM_44592
gi|522127887~Amycolatopsis_balhimycina
gi|754512873~Cronobacter_turicensis
gi|260217076|emb|CBA30822_1|~Cronobacter_turicensis_z3032
gi|495023039~Cronobacter_dublinensis_582
gi|639215880~Franconibacter_helveticus
gi|639220109~Cronobacter_sp__DJ34
gi|639223262~Franconibacter_pulveris
gi|654508218~gamma_proteobacterium_L18
gi|1019417696|emb|SAL58823_1|~Paraburkholderia_humi
gi|495131714~Acidovorax_sp__CF316
gi|1037121157|emb|SBT26001_1|~Alcaligenaceae_bacterium_LMG_29303
gi|515273481~Herbaspirillum_lusitanum
gi|497487540~Frankia_sp__CN3
gi|737889177~Cryptosporangium_arvum
gi|516887811~Henriciella_marina
gi|780824307~Hyphomonadaceae_bacterium_BRH_c29
gi|947330441~Phenylobacterium_sp__Root77
gi|501059567~Parvibaculum_lavamentivorans_DS-1
gi|657936874~alpha_proteobacterium_Mf_1_05b_01
gi|780038672~Tepidicaulis_marinus
gi|494949834~alpha_proteobacterium_BAL199
gi|655392178~Thalassobaculum_salexigens
gi|1055073088~Sphingomonas_mali
gi|1055054028~Sphingomonas_asaccharolytica
gi|970547561~Sphingopyxis_sp__A083
gi|1011962207~Sphingopyxis_terrae
gi|1054890630~Sphingomonas_sp__CCH10-B3
gi|736958119~Erythrobacter_sp__JL475
gi|736961825~Erythrobacter_longus
gi|494287530~Erythrobacter_sp__NAP1
gi|938956259~Sphingopyxis_macrogoltabida
gi|1041150767~Porphyrobacter_dokdonensis_DSW-74
gi|505256563~Ilumatobacter_coccineus_YM16-304
gi|551332814~Leucothrix_mucor
gi|948077348~Phenylobacterium_sp__Root700
gi|947343720~Phenylobacterium_sp__Root77
gi|916783207~Bradyrhizobium_sp__Ec3_3
gi|1055556112~Sphingorhabdus_sp__M41
gi|1054992181~Sphingomonadales_bacterium_EhC05
gi|297183863~uncultured_gamma_proteobacterium_EB000_65A11
gi|916836749~Afipia_sp__P52-10
gi|571920109~Afipia_sp__P52-10
gi|639267121~Bradyrhizobium_sp__UASWS1016
gi|575407907~Candidatus_Entotheonella_sp__TSY1
gi|575423897~Candidatus_Entotheonella_sp__TSY2
gi|522190024~Reyranella_massiliensis
gi|154155234~Parvibaculum_lavamentivorans_DS-1
gi|949111527~SAR92_bacterium_BACL26_MAG-121220-bin70
gi|949143418~SAR92_bacterium_BACL16_MAG-120322-bin99
gi|737511558~Gammaproteobacteria_bacterium_MOLA455
gi|494439834~gamma_proteobacterium_HTCC2207
gi|521084535~Microbulbifer_variabilis
gi|1012312069~Hyphomonadaceae_bacterium_UKL13-1
GSMAST4E1_ASY1_ANO1_4972_1
gi|780819985~Hyphomonadaceae_bacterium_BRH_c29
gi|737648345~Hyphomonas_johnsonii_MHS-2
gi|736796781~Hyphomonas_beringensis
gi|737602520~Hyphomonas_adhaerens_MHS-3
gi|975151843~Novosphingobium_sp__Fuku2-ISO-50
gi|655227847~Novosphingobium_acidiphilum
gi|496284170~Novosphingobium_sp__Rr_2-17
gi|763068835~Candidatus_Phaeomarinobacter_ectocarpi
gi|1037236375~Rhizobiales_bacterium_NRL2
gi|653007824~Leisingera_aquimarina
gi|504693338~Phaeobacter_inhibens_DSM_17395
gi|656258622~Leisingera_sp__ANG1
gi|746106906~Leisingera_sp__ANG-S5
gi|972927367~Ruegeria_sp__ZGT108
gi|568328179~Phaeobacter_gallaeciensis_DSM_26640
gi|742902017~Halocynthiibacter_namhaensis
gi|1032099847~Rhodobacteraceae_bacterium_EhC02
gi|549980783~Rhodobacteraceae_bacterium_HIMB11
gi|928976768~Shimia_sp__SK013
gi|902774516~Candidatus_Rhodobacter_lobularis
gi|935586203~Phaeobacter_sp__11ANDIMAR09
gi|545670962~alpha_proteobacterium_RS24
gi|495796075~alpha_proteobacterium_IMCC14465
gi|1054991127~Sphingomonadales_bacterium_EhC05
gi|657940414~alpha_proteobacterium_Mf_1_05b_01
gi|739637742~Sneathiella_glossodoripedis
gi|698798680~Aphanomyces_astaci
gi|494950877~alpha_proteobacterium_BAL199
gi|738646074~Nisaea_denitrificans
gi|931432593~Betaproteobacteria_bacterium_SG8_41
gi|653047896~Rhodospirillales_bacterium_URHD0088
gi|195541990~uncultured_bacterium_1114
gi|1056027575~Oceanibaculum_pacificum
gi|496230131~Oceanibaculum_indicum_P24
gi|953949641|dbj|BAT58114_1|~Variibacter_gotjawalensis
gi|1037238819~Rhizobiales_bacterium_NRL2
gi|740365158~Variovorax_paradoxus
gi|496180996~Acidovorax_sp__NO-1
gi|1049079617~Gammaproteobacteria_bacterium_TR3_2
gi|628311470~Cladophialophora_psammophila_CBS_110553
gi|1027053419~Cladophialophora_bantiana_CBS_173_52
gi|1022905576~Cladophialophora_immunda
gi|759288711~Capronia_semi-immersa
gi|628283472~Capronia_epimyces_CBS_606_96
gi|915040875~Rhinocladiella_mackenziei_CBS_650_93
gi|684160893~Exophiala_dermatitidis_NIH/UT8656
gi|1022638544~Exophiala_mesophila
gi|1000488034~Acidomyces_richmondensis_BFW
gi|452841180~Dothistroma_septosporum_NZE10
gi|1000797287~Cycloclasticus_sp__Phe_18
gi|655391802~Thalassobaculum_salexigens
gi|704409151~Bordetella_bronchiseptica_99-R-0433
gi|504878072~Bordetella_bronchiseptica
gi|704422383~Bordetella_bronchiseptica_MBORD591
gi|914800072~Achromobacter_piechaudii
gi|1001760458~Achromobacter_piechaudii
gi|497307380~Pseudomonas_sp__M1
gi|966457319~Pseudomonas_sp__EGD-AKN5
gi|505289329~Pseudomonas_denitrificans_ATCC_13867
gi|745855328~marine_actinobacterium_MedAcid-G3
gi|499933575~Ralstonia_eutropha_H16
gi|498510947~Ralstonia_sp__GA3-3
gi|503874539~Glaciecola_nitratireducens_FR1064
gi|647624409~Sneathiella_glossodoripedis
gi|1037239683~Rhizobiales_bacterium_NRL2
gi|654637019~Dongia_sp__URHE0060
gi|652479289~Inquilinus_limosus
gi|736831467~Inquilinus_limosus_MP06
gi|916867825~Rhodospirillales_bacterium_URHD0088
gi|737856554~Cupriavidus_sp__SK-4
gi|498510946~Cupriavidus_necator
gi|737856503~Cupriavidus_sp__SK-4
gi|771671937~Burkholderiaceae_bacterium_16
gi|759850222~Achromobacter_sp__RTa
0.1
Bacteria
Eukaryota
Other
SAG

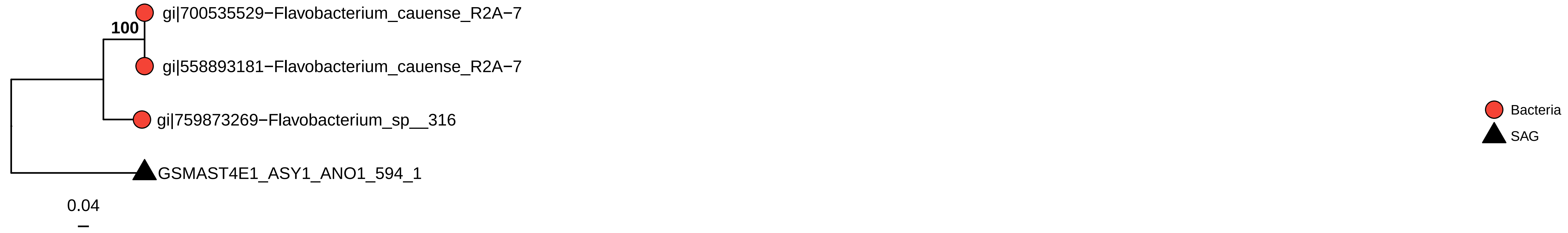
100
gi|700535529−Flavobacterium_cauense_R2A−7
gi|558893181−Flavobacterium_cauense_R2A−7
gi|759873269−Flavobacterium_sp__316
GSMAST4E1_ASY1_ANO1_594_1
0.04
Bacteria
SAG

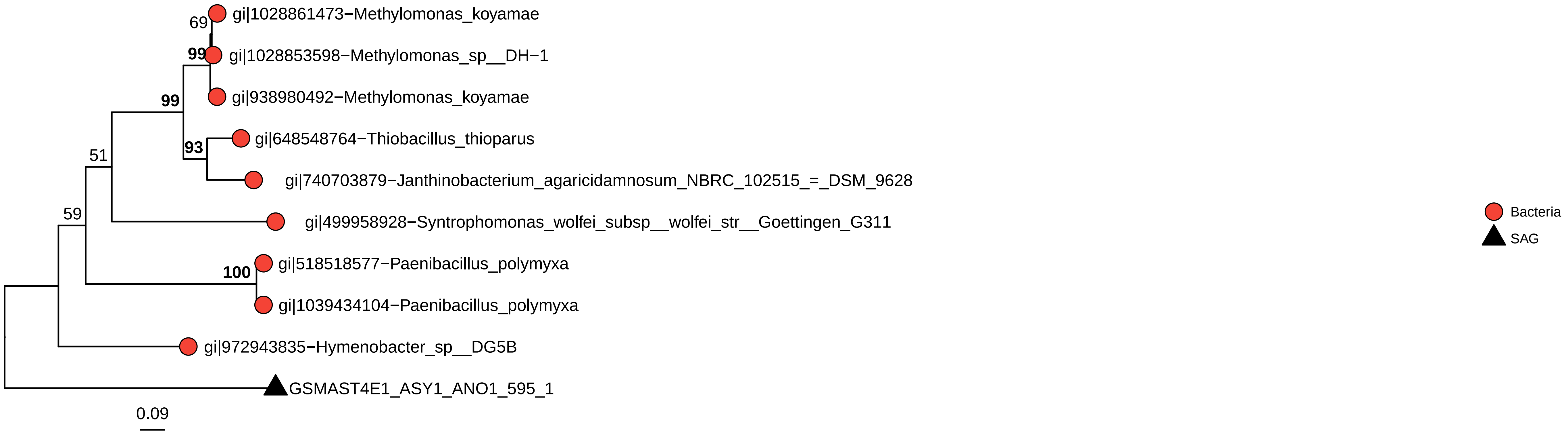
gi|1028861473−Methylomonas_koyamae
gi|1028853598−Methylomonas_sp__DH−1
gi|938980492−Methylomonas_koyamae
gi|648548764−Thiobacillus_thioparus
gi|740703879−Janthinobacterium_agaricidamnosum_NBRC_102515_=_DSM_9628
gi|499958928−Syntrophomonas_wolfei_subsp__wolfei_str__Goettingen_G311
gi|518518577−Paenibacillus_polymyxa
gi|1039434104−Paenibacillus_polymyxa
gi|972943835−Hymenobacter_sp__DG5B
GSMAST4E1_ASY1_ANO1_595_1
69
99
99
93
51
59
100
0.09
Bacteria
SAG

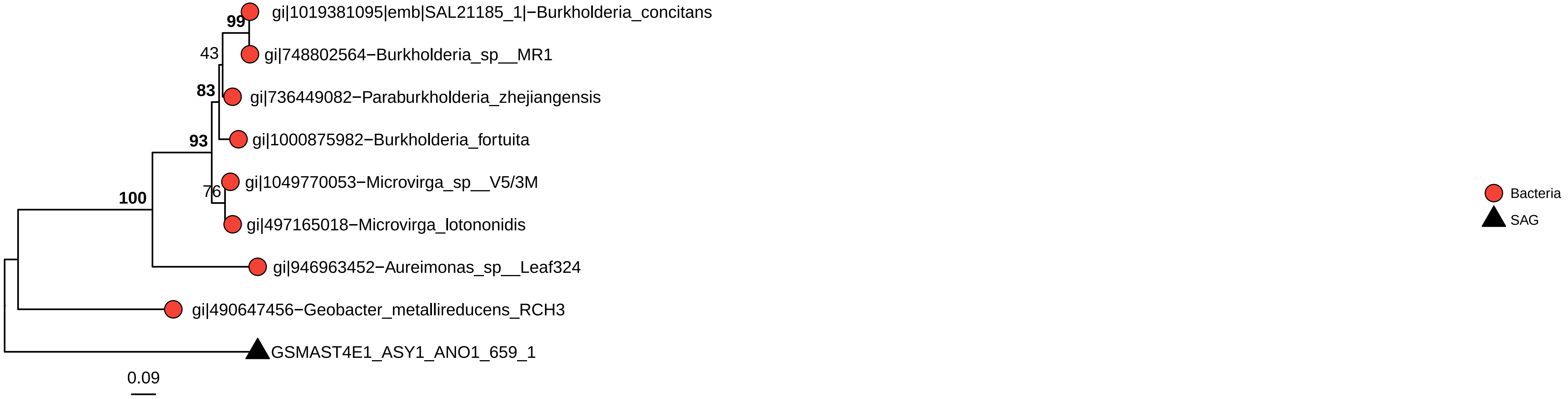
99
gi|1019381095|emb|SAL21185_1|−Burkholderia_concitans
43
gi|748802564−Burkholderia_sp__MR1
83
gi|736449082−Paraburkholderia_zhejiangensis
93
gi|1000875982−Burkholderia_fortuita
gi|1049770053−Microvirga_sp__V5/3M
76
100
gi|497165018−Microvirga_lotononidis
gi|946963452−Aureimonas_sp__Leaf324
gi|490647456−Geobacter_metallireducens_RCH3
GSMAST4E1_ASY1_ANO1_659_1
0.09
Bacteria
SAG

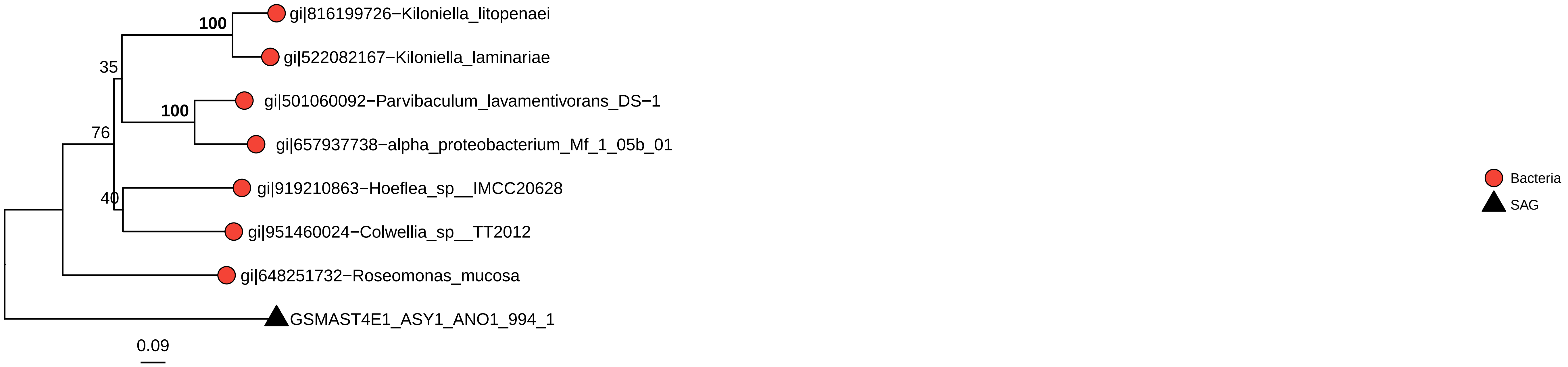

100
gi|816199726−Kiloniella_litopenaei
gi|522082167−Kiloniella_laminariae
35
100
gi|501060092−Parvibaculum_lavamentivorans_DS−1
76
gi|657937738−alpha_proteobacterium_Mf_1_05b_01
40
gi|919210863−Hoeflea_sp__IMCC20628
gi|951460024−Colwellia_sp__TT2012
gi|648251732−Roseomonas_mucosa
GSMAST4E1_ASY1_ANO1_994_1
0.09
Bacteria
SAG

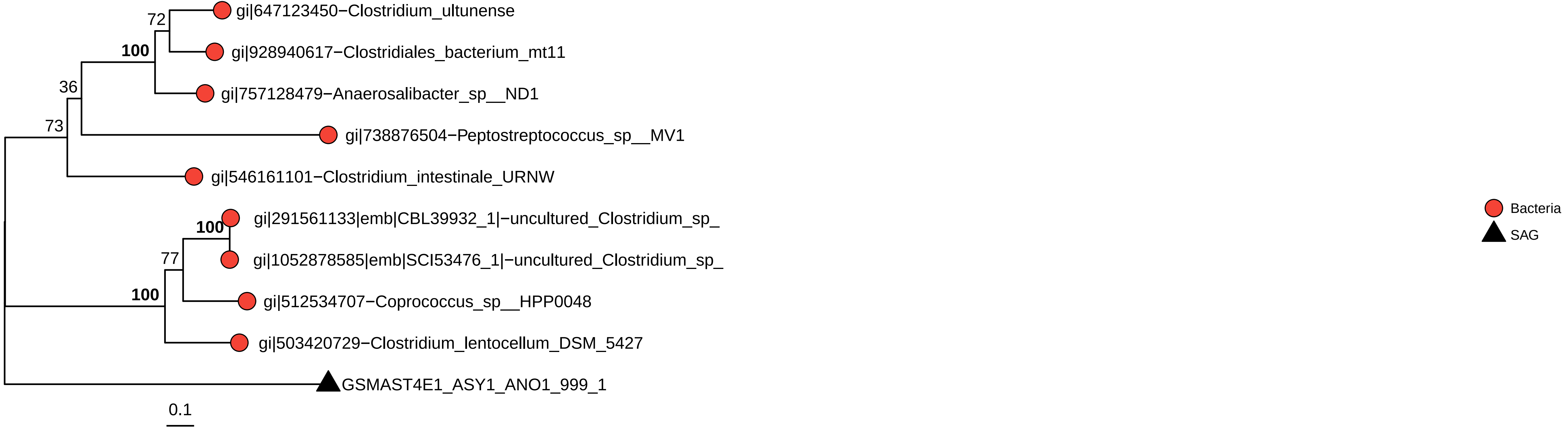
gi|647123450−Clostridium_ultunense
gi|928940617−Clostridiales_bacterium_mt11
gi|757128479−Anaerosalibacter_sp__ND1
gi|738876504−Peptostreptococcus_sp__MV1
gi|546161101−Clostridium_intestinale_URNW
gi|291561133|emb|CBL39932_1|−uncultured_Clostridium_sp_
gi|1052878585|emb|SCI53476_1|−uncultured_Clostridium_sp_
gi|512534707−Coprococcus_sp__HPP0048
gi|503420729−Clostridium_lentocellum_DSM_5427
GSMAST4E1_ASY1_ANO1_999_1
72
100
36
73
100
77
100
0.1
Bacteria
SAG
